# Language-dependent variation in observed mechanistic performance of web-enabled large language models across myeloid–mucosal immune contexts: a blinded expert evaluation

**DOI:** 10.3389/fimmu.2026.1965554

**Published:** 2026-09-15

**Authors:** Jinxi Hu, Tong Mei, Lei Lin, Chengyan Xia, Tian Ma, Penghui Xiang, Wenzhuo Wang, Siqing Kong, Buorui Zhang, Sen Cao, Yutong Ge, Xiaochen Li, Lujie Zhang, Liangjing Xia, Hongliang Duan, Chengla Yi

**Affiliations:** 1 Department of Orthopedics, Tongji Hospital, Tongji Medical College, Huazhong University of Science and Technology, Wuhan, China; 2 Department of Orthopedics, The First Hospital of Hebei Medical University, Shijiazhuang, China; 3 Department of Pharmacy, Shandong First Medical University, Taian, Shandong, China; 4 College of Chinese Medicine, Hong Kong Baptist University, Hong Kong, Hong Kong SAR, China; 5 Faculty of Applied Sciences, Macao Polytechnic University, Macao, Macao SAR, China; 6 Department of Minimally Invasive Oncology, The Third Affiliated Hospital of Shandong First Medical University, Jinan, China; 7 Department of Pharmacy, Panzhihua Central Hospital, Panzhihua, Sichuan, China

**Keywords:** large language models, mechanistic fidelity, mucosal immunity, multilingual evaluation, myeloid cells

## Abstract

**Objective:**

To determine whether observed mechanistic performance of web-enabled large language model (LLM) responses differs across response languages under deployed web conditions and to distinguish such language-associated shifts in relative model performance from stable between-model differences.

**Methods:**

Twenty-four standardized questions spanning intestinal, airway, and urogenital mucosa and four levels of mechanistic complexity were posed to five web-enabled LLMs in English, Simplified Chinese, Traditional Chinese, and Japanese, yielding 480 responses. Twenty senior clinician-experts, assigned to language-specific panels and blinded to model identity, independently evaluated each response using a six-domain Mechanistic Fidelity Score (MFS; 0–24), with two ratings per response. Overall model effects were tested using block-adjusted regression with question-clustered inference. Because expert panels were nested within language, raw cross-language means were treated descriptively, whereas language-dependent relative performance was assessed after within-reviewer standardization. Each standardized value represents performance relative to an individual reviewer’s own scoring distribution rather than an absolute cross-language MFS difference.

**Results:**

All 480 responses received two ratings (960 evaluations). Inter-rater agreement was moderate-to-good (Krippendorff α=0.709; Lin CCC = 0.709). Overall model performance differed substantially (F(4,23)=95.80, P<.001). Kimi K3 (mean MFS 20.65) and ChatGPT 5.6 Sol (20.43) outperformed DeepSeek V4 Flash 0731, ChatGPT 5.6 Luna, and Xiaomi V2.5 Pro Ultra after Holm correction (all P<.001), while differing nonsignificantly from each other (P = .182). Reviewer-standardized scores showed a strong model-by-language interaction in observed relative performance (F(12,191)=44.21, P<.001), whereas no statistically significant model-by-complexity interaction was detected in the present question set (P = .328).

**Conclusion:**

Mechanistic fidelity in the evaluated web-enabled systems was strongly model dependent, while observed relative performance varied across language panels. Because response language was not separable from panel composition and could also influence translation and web retrieval, these findings do not establish an intrinsic causal effect of language on underlying model reasoning. Multilingual biomedical evaluation should therefore test long-form mechanistic explanations directly in the language and deployment setting of intended use rather than infer equivalence from English performance alone.

## 1 Introduction

Mucosal immunity is organized through time-dependent cooperation rather than the isolated activity of a single leukocyte population. In the intestine, macrophage efferocytosis of apoptotic neutrophils can couple inflammatory resolution to epithelial repair (1), while resident eosinophils participate in tissue homeostasis and can shape dendritic-cell behavior (2, 3). In the airway, extracellular DNA and neutrophil extracellular traps may become linked to inflammasome activation and epithelial injury in severe asthma (4). The urinary tract adds another layer of context dependence: prior infection can leave persistent epithelial immune imprinting (5), and bladder tumors can exploit neutrophil and macrophage programs to remodel antitumor immunity (6, 7). Crohn disease provides a complementary example in which a macrophage-centered IL-23/IFN-γ circuit can sustain chronic intestinal inflammation (8). These observations illustrate a basic difficulty of mechanistic immunology: the same myeloid lineage may be protective at one stage, damaging at another, and functionally different across mucosal compartments.

This conditional structure creates a demanding test for large language models. A model can retrieve the names of cytokines, receptors, or cell types without necessarily representing how those elements are connected, when the direction of an interaction changes, or which claims are well established versus context dependent. Multilingual medical LLM development has begun to address linguistic coverage through dedicated corpora and benchmarks (9), multilingual biomedical pretraining and translated evaluation sets (10), and Chinese clinical benchmarks constructed around expert-guided scenarios (11). Work using Chinese licensing questions has likewise shown that performance in a non-English setting cannot be assumed from English-oriented development alone (12). More general multilingual benchmarks have reinforced the value of parallel questions and expert review when testing reasoning across languages (13).

A second shift in medical LLM evaluation concerns the form of the task. Many early benchmarks emphasized multiple-choice examination accuracy, whereas newer resources increasingly use real clinical scenarios, expert-developed checklists, error taxonomies, and long-form reasoning (14–17). This change matters for mechanistic biomedical science because a biologically plausible response can be superficially fluent yet omit a key cell–cell feedback loop, flatten a protective–pathogenic duality, or overstate evidence from a narrow experimental context. Such failures are difficult to capture with answer-key accuracy alone.

We therefore designed a blinded, expert-adjudicated benchmark focused on a specific mechanistic domain: myeloid-cell orchestration across intestinal, airway, and urogenital mucosal immunity. Five web-enabled LLMs answered 24 parallel questions in four languages, with every model–language–question combination generated in an independent session. The primary objective was to compare mechanistic fidelity across models while testing whether relative model performance changed with language. Secondary objectives were to characterize which components of mechanistic reasoning differentiated models, assess whether increasing mechanistic complexity altered model rankings, and quantify agreement between independent expert raters. The study was deliberately framed as an evaluation of generated mechanistic explanations rather than a test of clinical decision making or patient outcomes. The novelty of the present design lies in jointly evaluating long-form causal structure, four parallel languages, web-enabled retrieval, and blinded domain-expert scoring within a single mechanistic benchmark. Although this is not a patient-outcome study, the question has practical significance because multilingual mechanistic explanations may be reused in biomedical education, literature synthesis, hypothesis generation, and clinical-support workflows, where a plausible but context-flattened explanation can misstate biological causality or therapeutic trade-offs.

## 2 Methods

### 2.1 Study design and evaluation framework

We conducted a factorial, cross-language evaluation of web-enabled LLM-generated explanations in myeloid–mucosal immunology. The design crossed 24 standardized mechanistic questions with four languages and five LLMs, producing 480 response units. Each response was evaluated independently by two experts, yielding 960 reviewer-level ratings. The unit of model generation was one model–language–question combination; the unit of descriptive response-level analysis was the mean of the two independent expert ratings for that response. **Figure 1** summarizes the study workflow.

**Figure 1:**
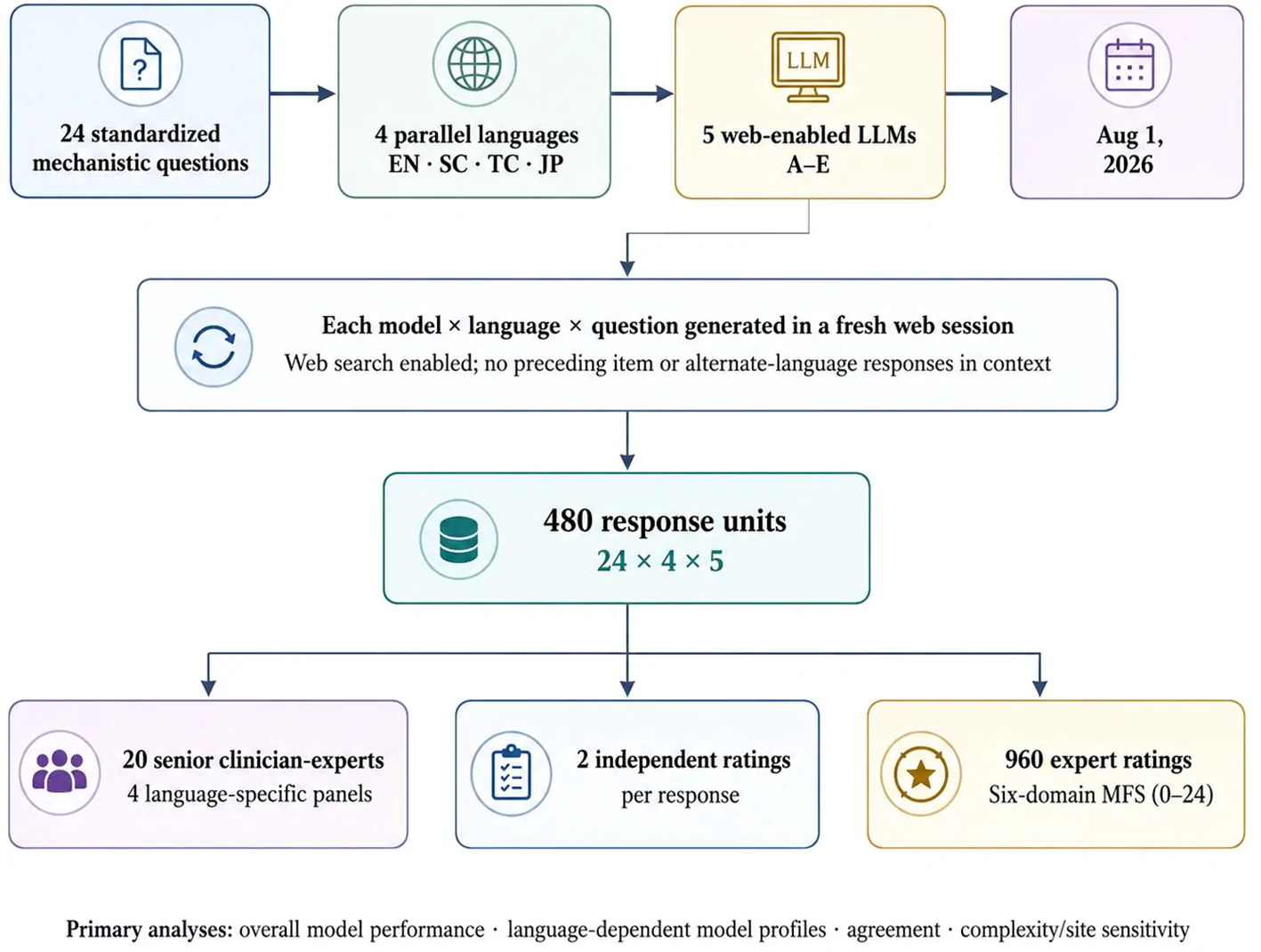
Study design and evaluation workflow. Each of the 480 model–language–question combinations was generated in an independent web session. All responses received two blinded expert ratings using the six-domain Mechanistic Fidelity Score (MFS).

### 2.2 Development of the mechanistic question set

The question set was constructed to sample biologically distinct forms of myeloid reasoning rather than to maximize the number of isolated facts. Twenty-four questions were distributed evenly across four prespecified complexity levels. Level 1 focused on foundational myeloid networks and homeostatic barrier defense; Level 2 required explicit intercellular crosstalk and transition from inflammatory recruitment to resolution; Level 3 integrated disease-specific mechanisms; and Level 4 required translational reasoning about selective intervention, therapeutic trade-offs, or reprogramming of myeloid states. Six questions were assigned to each level. These four levels were prespecified conceptual strata of requested mechanistic integration; they were not empirically validated psychometric levels of item difficulty.

The mucosal design was also balanced: eight questions addressed intestinal mucosa, eight airway mucosa, and eight urogenital mucosa. Across the full set, the requested cell types included neutrophils, eosinophils, mast cells or basophils, monocytes/macrophages, and dendritic cells. Mechanistic prompts included, among other topics, neutrophil extracellular traps, efferocytosis, lipid mediators, metabolic and epigenetic reprogramming, trained immunity, mucosal repair, and tumor-associated myeloid networks.

### 2.3 Language preparation

The biological target of each question was fixed before model testing. Parallel versions were prepared in English, Simplified Chinese, Traditional Chinese, and Japanese with the intention of preserving the same requested cell populations, tissue context, disease state, mechanistic relationships, and translational constraints. Disease names, cytokine nomenclature, and established molecular abbreviations were retained in their conventional scientific forms where appropriate. Equivalence was assessed at the task-content level rather than by requiring literal lexical identity: each version had to preserve the prespecified cell populations, tissue or disease state, requested causal relationships, translational constraints, and assigned complexity category, while allowing conventional scientific phrasing in the target language. The complete set of 24 questions in English, Simplified Chinese, Traditional Chinese, and Japanese is reproduced in **Supplementary Table A1**. The four language sets were then frozen before the generation phase; no response in one language was used to create or revise the response in another language.

### 2.4 LLM response generation

Five models were evaluated: DeepSeek V4 Flash 0731 (Model A), ChatGPT 5.6 Luna (Model B), ChatGPT 5.6 Sol (Model C), Kimi K3 (Model D), and Xiaomi V2.5 Pro Ultra, an internal-test model at the time of evaluation (Model E). All responses were generated on August 1, 2026 through web interfaces, with web search enabled for every model. The study therefore evaluates web-enabled model behavior under the conditions available on that date, rather than closed-book parametric knowledge alone. Because public web interfaces can change without a frozen parameter snapshot, these model labels identify the interfaces available on the study date rather than immutable model weights.

Each individual question was generated in a newly initiated session. For a given model, English, Simplified Chinese, Traditional Chinese, and Japanese responses were produced independently; no preceding question, answer, or alternate-language version of the same item was present in the active conversation context. The standardized question text in the target language was used as the task content. Responses were retained as generated and were not rewritten to harmonize terminology, length, or structure across models. Because generation occurred through web interfaces rather than standardized APIs, sampling parameters that were not exposed by a platform were not forced to common values. Operationally, the user-side standalone prompt consisted of the frozen target-language question text itself; no model-specific preamble, example answer, persona instruction, or additional biological context was appended. Platform-level system prompts, hidden retrieval/ranking logic, and unexposed decoding settings were not observable or controllable and were therefore treated as part of the deployed web system.

### 2.5 Expert panel and eligibility

Twenty experts participated in the blinded evaluation. The English panel included four experts from Hong Kong Baptist University, Monash University, and the University of Hawai‘i. The Simplified Chinese panel included eight experts from Panzhihua Central Hospital, the Third Affiliated Hospital of Shandong First Medical University, and Tongji Hospital, Tongji Medical College, Huazhong University of Science and Technology. The Traditional Chinese panel included six experts from Macao Polytechnic University, the School of Chinese Medicine at Hong Kong Baptist University, and Macau University of Science and Technology. The Japanese panel included two experts from Kunming University of Science and Technology; both had obtained master’s degrees in Japan. Expert scoring was completed from August 2 to August 8, 2026.

A common seniority threshold was used. Every evaluator held clinical physician licensure, a professional rank equivalent to associate-senior level or above, at least 10 years of clinical experience, an h-index greater than 10, and a first- or corresponding-author publication in a relevant field within the preceding three years. Only institutional affiliation and non-identifying qualification thresholds were retained in the analytic archive. **Table 1** summarizes the panel structure.

**Table 1:** Expert-panel structure and rating allocation.

Panel	Experts	Institutions	Questions per expert	Ratings per expert	Total ratings
English	4	Hong Kong Baptist University; Monash University; University of Hawai‘i	12	60	240
Simplified Chinese	8	Panzhihua Central Hospital; Third Affiliated Hospital of Shandong First Medical University; Tongji Hospital, Tongji Medical College, HUST	6	30	240
Traditional Chinese	6	Macao Polytechnic University; School of Chinese Medicine, Hong Kong Baptist University; Macau University of Science and Technology	8	40	240
Japanese	2	Kunming University of Science and Technology	24	120	240

All panels evaluated exactly 240 reviewer-level model responses. Across the full dataset, every language–question–model response therefore received two independent ratings.

### 2.6 Blinding and rating allocation

Experts were informed that the materials were generated by different LLMs but were not given the underlying model identities during scoring. Responses were presented under anonymous model labels. Each expert rated only the questions assigned to that language panel. The allocation scheme was constructed so that every response was scored independently by two experts, while distributing workload across the larger English, Simplified Chinese, and Traditional Chinese panels. Because only two Japanese-language experts were available, both evaluated all 24 Japanese questions.

### 2.7 Mechanistic fidelity score

The primary outcome was a study-specific Mechanistic Fidelity Score (MFS) designed for long-form immunological explanations. Six domains were scored from 0 to 4: factual accuracy, mechanistic completeness, crosstalk integration, tissue/disease specificity, protective–pathogenic duality, and evidence fidelity. Dimension-specific anchors moved from absent, substantially incorrect, or poorly supported performance at the lower end to accurate, comprehensive, context-sensitive, and appropriately qualified performance at the upper end. The six dimensions were summed to produce a total score from 0 to 24.

A common ordinal anchor ladder was applied within each domain: 0 indicated absent, irrelevant, or predominantly incorrect content; 1 indicated limited performance with major errors or omissions; 2 indicated partially correct performance with important mechanistic or contextual gaps; 3 indicated largely correct and context-appropriate performance with only minor omissions or overstatement; and 4 indicated accurate, comprehensive, integrated, and appropriately qualified performance.

The ladder was operationalized by domain. For factual accuracy, scores 1–4 progressed from multiple substantive errors to no substantive factual error; for mechanistic completeness, from addressing only a small fraction of requested processes to covering all major requested processes; for crosstalk integration, from listing cells without correct relationships to coherent reciprocal and sequential interactions; for tissue/disease specificity, from generic or mismatched context to fully context-specific explanation; for protective–pathogenic duality, from a fixed or misleading beneficial/harmful label to an integrated account of state-, time-, and trade-off-dependent roles; and for evidence fidelity, from unsupported or overconfident claims to consistent calibration of established evidence, context dependence, and uncertainty.

Factual accuracy assessed whether named cells, mediators, pathways, and directional biological claims were correct. Mechanistic completeness assessed whether the response addressed the major processes explicitly requested by the question rather than substituting a generic overview. Crosstalk integration focused on whether the model represented reciprocal or sequential relationships among myeloid populations. Tissue/disease specificity assessed contextual fidelity to the named mucosa or disease. Protective–pathogenic duality assessed whether the explanation preserved state- and time-dependent roles rather than assigning a lineage a fixed beneficial or harmful label. Evidence fidelity assessed whether claims were calibrated to the strength and scope of available evidence rather than presented with unwarranted certainty. The six domains were intentionally related but not interchangeable: completeness measured coverage of requested mechanisms, crosstalk measured causal linkage among covered elements, and tissue/disease specificity measured whether those links were correctly contextualized. Reviewers were instructed to score each criterion separately rather than allow weakness in one domain to mechanically determine another.

Experts also recorded whether a response contained a critical mechanistic error using yes, no, or uncertain categories and rated confidence in their own assessment on a five-point scale. These variables were analyzed separately from the MFS and were not used to modify the six component scores.

A critical mechanistic error was defined as a claim that materially reversed or distorted the central causal account—for example, assigning a key effector function to the wrong cell or tissue context, presenting a context-dependent pathway as uniformly directional in a way that changed the conclusion, or proposing near-complete suppression of a host-defense pathway while omitting the requested infection trade-off. Minor omissions, terminology preferences, or absence of a secondary mediator did not by themselves qualify. For illustration, describing persistent NET formation as uniformly tissue protective despite its potential for epithelial injury would meet the threshold if central to the answer, whereas acknowledging both antimicrobial and tissue-damaging NET effects would not. Reviewers could select “uncertain” when the seriousness of a claim or the supporting evidence base was ambiguous.

### 2.8 Data management and quality control

The scoring dataset was assembled using reviewer codes rather than names. The final file contained all 960 expected reviewer-level observations and no missing MFS dimension scores. Range checks required each MFS component to fall between 0 and 4 and confidence to fall between 1 and 5. Language-specific wording for the critical-error item was harmonized to the common categories yes, no, and uncertain before analysis. No imputation was required. The original language-specific response booklets and de-identified reviewer-level ratings were archived separately from direct personal identifiers.

### 2.9 Statistical analysis

Analyses were organized around four prespecified questions: whether models differed in overall mechanistic fidelity; whether their relative performance changed with language; whether between-model contrasts varied across the four prespecified conceptual complexity categories; and whether independent experts showed sufficient agreement to support response-level comparison. Descriptive statistics are reported as means and standard deviations or 95% confidence intervals, as appropriate.

For overall model comparisons, the two independent ratings were first averaged for each response, yielding 480 response-level MFS values. We fitted a fixed-effects regression that compared the five models within the same question–language block. Standard errors were clustered by question to preserve the repeated four-language structure of each biological item. The omnibus model test was followed by all pairwise model contrasts; Holm adjustment controlled family-wise multiplicity. Question-clustered 95% confidence intervals were used for model-level estimates and pairwise differences. This specification is equivalent to including a fixed effect for each question–language block plus model indicators, so every model contrast is made within the same biological item and response language. Because the paired ratings were averaged before this analysis, reviewer-level duplication did not enter the overall model test.

A direct raw comparison of mean MFS across languages would be confounded because each language was scored by a different expert panel. We therefore treated absolute language-specific MFS means as descriptive only. To examine language-associated shifts in observed relative model performance, each reviewer’s MFS values were standardized within reviewer to a mean of zero and a standard deviation of one. A positive z score therefore indicated performance above that reviewer’s own mean, and one z unit represented one reviewer-specific standard deviation rather than one common MFS unit. The interaction model included model and language terms, question fixed effects, and the model-by-language interaction. Standard errors were clustered by reviewer–question block, which grouped the five model scores assigned by the same reviewer to the same question and preserved their within-block dependence. The interaction, rather than a language main effect, was the primary inferential test of whether relative model performance varied across language panels; the standardized values are relative positions, not absolute cross-language effect sizes or a causal estimate of language.

Mechanistic complexity was examined using the four prespecified levels. An analogous block-adjusted interaction model tested whether model rankings changed with complexity. A reviewer-standardized language-by-complexity analysis was treated as secondary because the panels remained language specific. Mucosal-site means were summarized for intestinal, airway, and urogenital questions to identify broad site gradients without overinterpreting small exploratory differences. Complexity was treated as a four-level categorical construct; the model-by-complexity term tested heterogeneity of between-model contrasts across those categories rather than a monotonic “difficulty” effect. As a *post hoc* robustness check, we also repeated the within-panel rank-pattern interpretation after omitting each language panel in turn. This leave-one-language-out check was descriptive and was not treated as a second formal interaction test.

Inter-rater agreement for the total MFS was estimated from the paired ratings for all 480 responses. Interval-scale Krippendorff α and Lin’s concordance correlation coefficient (CCC) were used as concordance measures; Spearman correlation and the mean and median absolute rating difference were reported as complementary descriptors. All tests were two-sided. A corrected P value <.05 was considered statistically significant for planned pairwise contrasts. Analyses were performed in Python 3.13 using pandas 2.2.3, NumPy 2.3.5, SciPy 1.17.0, and statsmodels 0.14.6.

### 2.10 Ethics and consent

The Ethics Committee of Panzhihua Central Hospital granted an exemption from full ethics review for this study. The evaluation involved model-generated scientific text and expert ratings and did not involve patients, patient records, or clinical interventions. All participating experts provided informed consent.

## 3 Results

### 3.1 Dataset completeness and rating behavior

The complete analytic dataset contained 480 model responses and 960 expert ratings from 20 evaluators. Every response had two ratings. Across all reviewer-level observations, the mean MFS was 16.98 (SD 5.68) and mean scoring confidence was 3.83 (SD 1.17) on the five-point confidence scale. No response received a definite critical-mechanistic-error rating. Of the 960 ratings, 906 (94.4%) were marked no critical error and 54 (5.6%) were marked uncertain. Because the definite critical-error category was rare by design and no “yes” ratings occurred, this result should not be interpreted as evidence that the models were equivalently free of consequential shortcomings; graded deficiencies were captured by the six MFS domains and the 54 uncertain critical-error ratings.

Across all language panels, interval Krippendorff α for total MFS was 0.709, Lin CCC was 0.709, and Spearman ρ was 0.807. The median absolute difference between paired ratings was 1 point. Agreement varied by panel. English and Traditional Chinese showed the strongest coefficient-based concordance. Simplified Chinese showed substantially lower agreement. The Japanese panel had a lower concordance coefficient but a median absolute difference of 0 points, reflecting the narrow, near-ceiling score range in that panel.

### 3.2 Overall model performance

Overall MFS differed markedly across the five models (F(4,23)=95.80, P<.001). Kimi K3 (Model D) had the highest mean response-level MFS, 20.65 (95% CI 20.18–21.12), followed closely by ChatGPT 5.6 Sol (Model C), 20.43 (19.96–20.89). ChatGPT 5.6 Luna (Model B), Xiaomi V2.5 Pro Ultra (Model E), and DeepSeek V4 Flash 0731 (Model A) had means of 14.94, 14.60, and 14.27, respectively. Models C and D each exceeded A, B, and E after Holm adjustment (all adjusted P<.001). The 0.22-point difference between D and C was not significant after correction (Holm-adjusted P = .182). Differences among A, B, and E were also not significant after correction in the overall analysis (**Table 2**).

**Table 2:** Overall mechanistic fidelity by model.

Model	Mean MFS (95% CI)	Factual accuracy	Mechanistic completeness	Crosstalk integration	Tissue/disease specificity	Protective–pathogenic duality	Evidence fidelity
A DeepSeek V4 Flash 0731	14.27 (13.76–14.78)	3.11	2.11	1.85	2.71	2.55	1.93
B ChatGPT 5.6 Luna	14.94 (14.63–15.25)	3.02	2.33	1.95	2.94	2.58	2.11
C ChatGPT 5.6 Sol	20.43 (19.96–20.89)	3.86	3.75	3.06	3.64	3.20	2.92
D Kimi K3	20.65 (20.18–21.12)	3.82	3.58	3.00	3.58	3.54	3.14
E Xiaomi V2.5 Pro Ultra	14.60 (14.18–15.03)	2.91	2.19	1.91	2.90	2.47	2.22

Dimension values are means on the 0–4 scale. Model-level MFS confidence intervals were calculated across question clusters after averaging across languages.

Pairwise question-clustered contrasts are shown in **Figure 2**.

**Figure 2:**
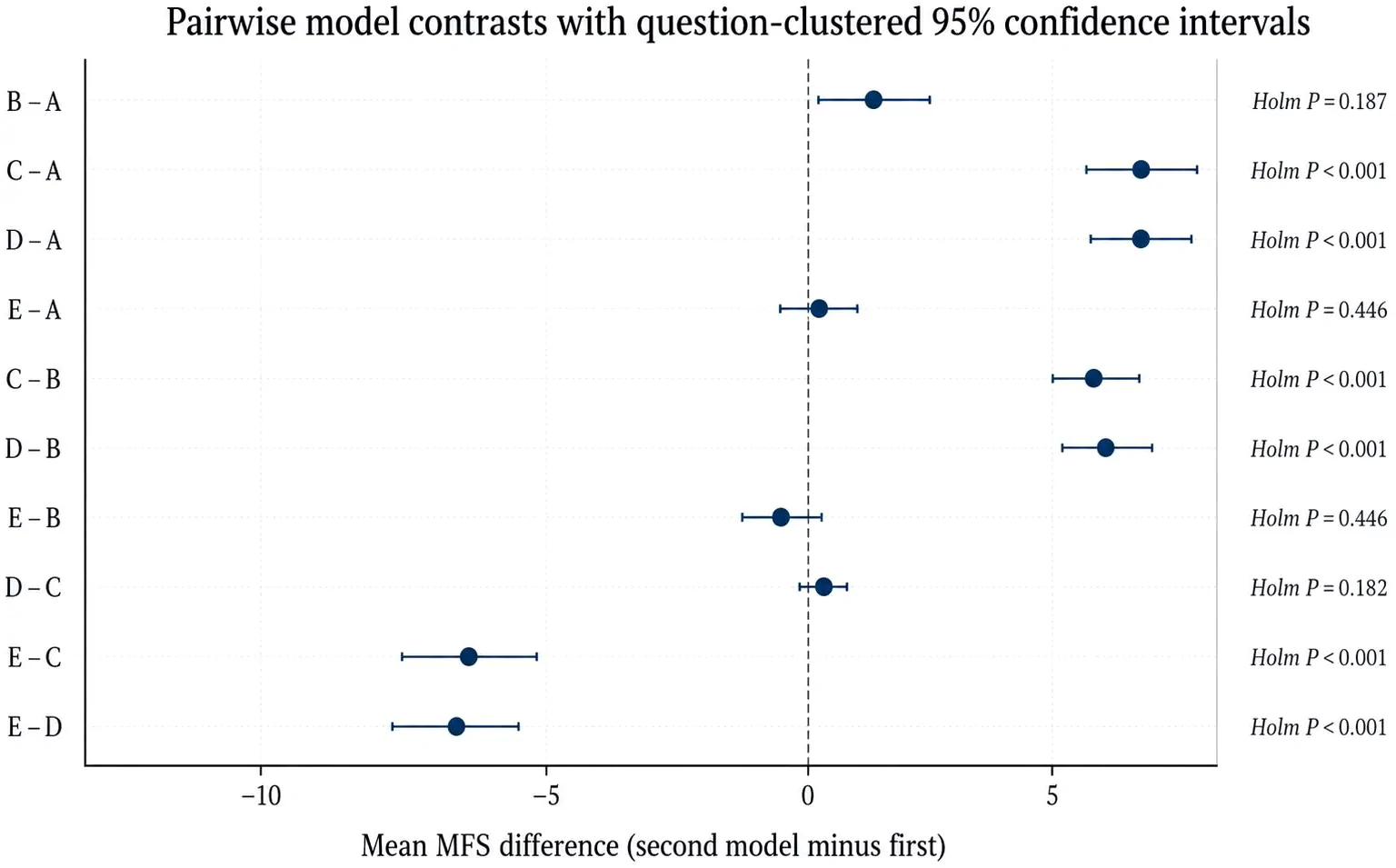
Pairwise model contrasts for total MFS. Differences are expressed as the mean score of the second model minus the first model; horizontal bars show question-clustered 95% confidence intervals. Holm-adjusted P values are shown at right.

### 3.3 Mechanistic-domain profiles

The overall separation between the two leading models and the remaining three was not driven by a single scoring dimension (**Figure 3**). Models C and D had factual-accuracy means above 3.8/4 and substantially higher completeness, crosstalk integration, and tissue/disease specificity than A, B, or E. The most informative differences appeared in dimensions that required relationships rather than terminology alone. Crosstalk integration averaged 3.06 for C and 3.00 for D but remained below 2.0 for A, B, and E. Evidence fidelity was the lowest-scoring dimension for most models and remained below 3.2 even for the leaders.

**Figure 3:**
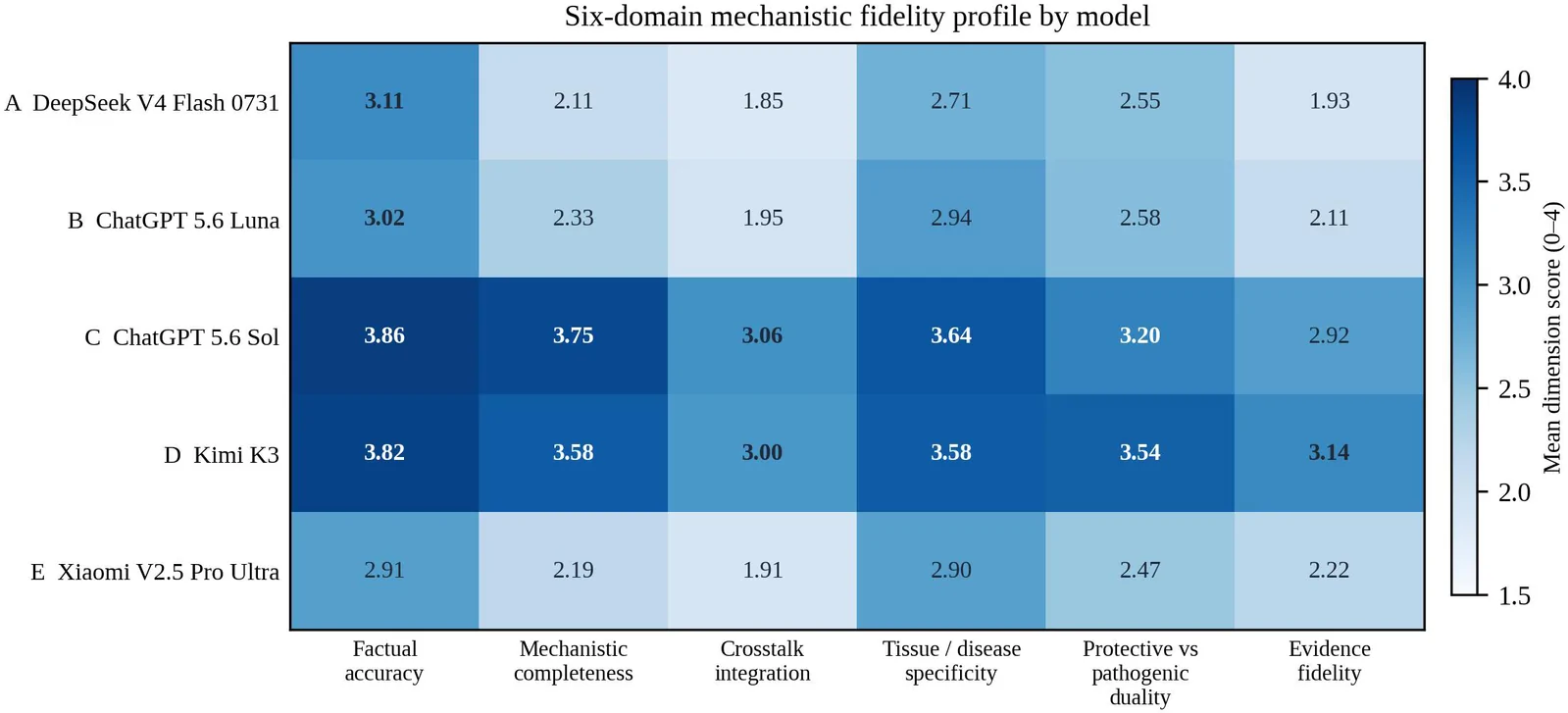
Six-domain mechanistic fidelity score profile by model. Cell values are mean expert scores on the 0–4 scale. Darker shading indicates higher performance.

The two leading models also showed different strengths. Model C scored slightly higher than D for factual accuracy, mechanistic completeness, crosstalk integration, and tissue/disease specificity. Model D scored higher for protective–pathogenic duality and evidence fidelity. These complementary profiles help explain why their total MFS values were close despite different patterns across the six domains.

### 3.4 Language-dependent shifts in observed relative model performance

Raw language-specific MFS values differed substantially, but these absolute means cannot be interpreted as isolated language effects because each language was evaluated by a different expert panel. The raw values are therefore reported to show the observed score distributions, not to claim that one language was intrinsically easier or harder. The Japanese panel, for example, used a narrow range near the upper end of the MFS scale, whereas the Simplified Chinese panel showed much wider between-reviewer dispersion. Interpretation is especially constrained by the Japanese panel’s two-rater, range-restricted structure and by the lower coefficient-based agreement in the Simplified Chinese panel.

The reviewer-standardized analysis examined whether observed model rankings and relative separations changed across language panels. The model-by-language interaction was strong (F(12,191)=44.21, P<.001). Models C and D remained the two highest relative performers in every language panel, but the lower-ranked models did not preserve a stable order. In English, the descriptive order was D>C>A>B>E; in Simplified Chinese it was D>C>B>E>A; in Traditional Chinese it was D>C>E>B>A; and in Japanese C and D were tied at the top, followed by E, B, and A. Thus, the language-dependent result was not a simple uniform gain or loss shared by every model. Instead, the magnitude and direction of relative model differences changed with language; the corresponding language-specific response profiles are summarized in **Table 3**. In the descriptive leave-one-language-out check, omitting any single panel did not eliminate cross-language rank reversals among Models A, B, and E across the remaining three panels, while Models C and D remained the upper tier. Thus, the observed rank instability was not attributable to one panel alone, although this sensitivity check does not remove panel, translation, or retrieval confounding.

**Table 3:** Language-specific response profiles.

Language	Model	Raw MFS mean (95% CI)	Reviewer-standardized mean z	Within-panel rank
English	A DeepSeek V4 Flash 0731	16.69 (15.66–17.72)	0.01	3
English	B ChatGPT 5.6 Luna	12.81 (11.95–13.68)	-0.76	4
English	C ChatGPT 5.6 Sol	22.10 (21.67–22.54)	0.99	2
English	D Kimi K3	22.44 (22.06–22.81)	1.05	1
English	E Xiaomi V2.5 Pro Ultra	9.94 (9.35–10.53)	-1.29	5
Simplified Chinese	A DeepSeek V4 Flash 0731	8.17 (6.75–9.59)	-0.86	5
Simplified Chinese	B ChatGPT 5.6 Luna	11.75 (10.62–12.88)	-0.25	3
Simplified Chinese	C ChatGPT 5.6 Sol	17.71 (16.06–19.36)	0.78	2
Simplified Chinese	D Kimi K3	17.88 (16.24–19.51)	0.82	1
Simplified Chinese	E Xiaomi V2.5 Pro Ultra	10.46 (8.95–11.97)	-0.49	4
Traditional Chinese	A DeepSeek V4 Flash 0731	11.27 (10.53–12.01)	-1.20	5
Traditional Chinese	B ChatGPT 5.6 Luna	13.46 (12.49–14.42)	-0.60	4
Traditional Chinese	C ChatGPT 5.6 Sol	18.92 (18.60–19.24)	0.85	2
Traditional Chinese	D Kimi K3	19.31 (19.08–19.54)	0.95	1
Traditional Chinese	E Xiaomi V2.5 Pro Ultra	15.75 (14.97–16.53)	0.00	3
Japanese	A DeepSeek V4 Flash 0731	20.96 (20.65–21.27)	-1.18	5
Japanese	B ChatGPT 5.6 Luna	21.73 (21.53–21.93)	-0.44	4
Japanese	C ChatGPT 5.6 Sol	22.98 (22.94–23.02)	0.77	1
Japanese	D Kimi K3	22.98 (22.94–23.02)	0.77	2
Japanese	E Xiaomi V2.5 Pro Ultra	22.27 (22.04–22.50)	0.08	3

Raw MFS means are descriptive because expert panels were language-specific. Reviewer-standardized z scores were used for the model-by-language inferential test and indicate each model’s relative position within the scoring distribution of the reviewers who evaluated that language. A z score of +1 corresponds to one reviewer-specific standard deviation above that reviewer’s own mean; these values should not be interpreted as absolute MFS differences between languages or as standardized effects against a common biological gold standard.

The raw and reviewer-standardized profiles are shown together in **Figure 4**.

**Figure 4:**
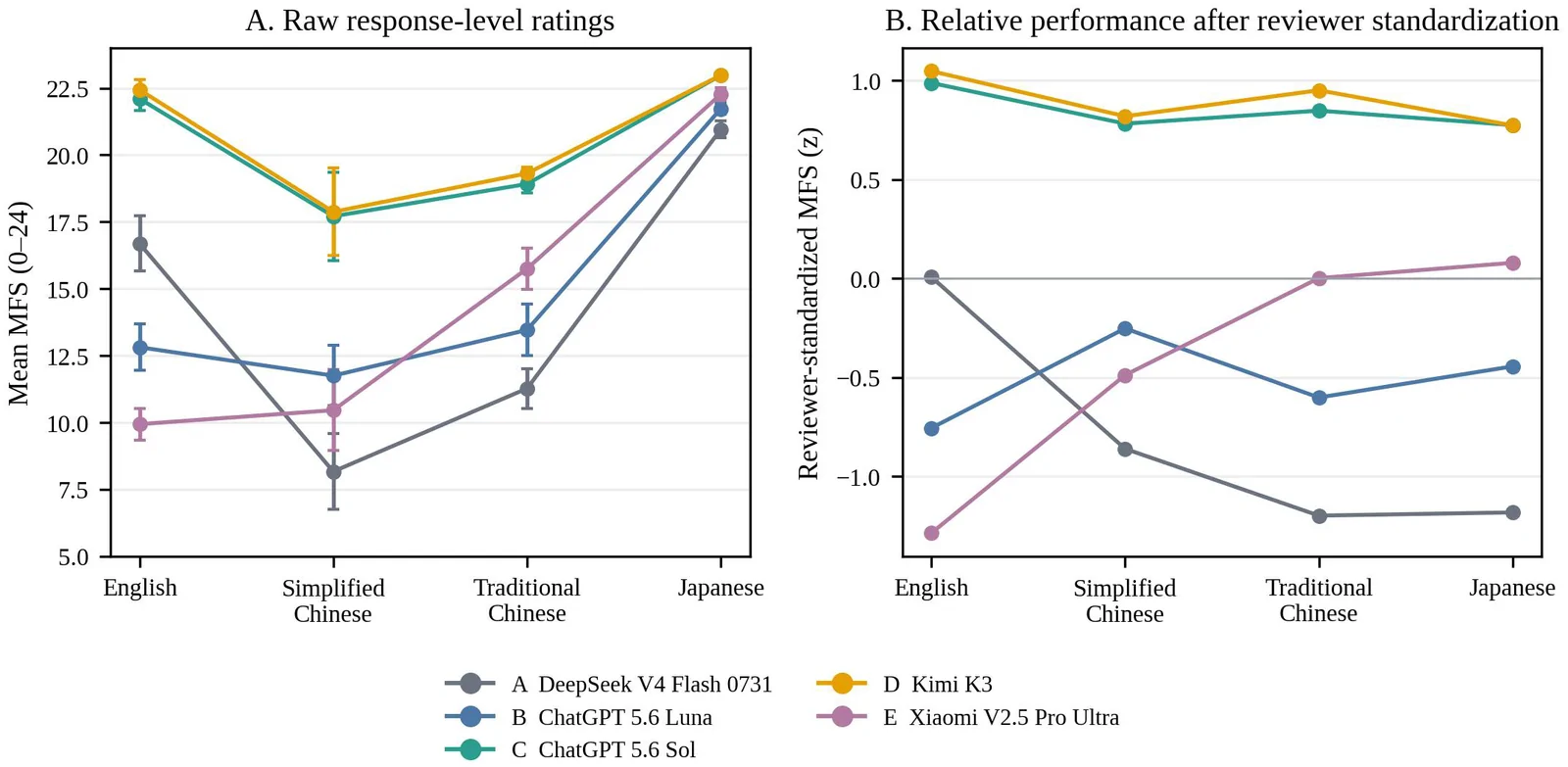
Language-specific model profiles. **(A)** shows raw response-level MFS means with 95% confidence intervals; these absolute values are descriptive because expert panels were nested within language. **(B)** shows reviewer-standardized MFS values used to examine relative model performance across languages.

### 3.5 Question complexity and mucosal context

Mean MFS values were similar across the four prespecified complexity levels: 16.71 for Level 1, 17.22 for Level 2, 16.81 for Level 3, and 17.17 for Level 4. No statistically significant model-by-complexity interaction was detected in the present question set (F(12,191)=1.14, P = 0.328). The trajectories of C and D remained clearly separated from A, B, and E across foundational, crosstalk, disease-integrated, and translational questions (**Figure 5**). A reviewer-standardized language-by-complexity interaction was statistically detectable (F(12,191)=10.32, P<.001), but the within-language standardized shifts across levels were small and did not form a consistent monotonic complexity gradient.

**Figure 5:**
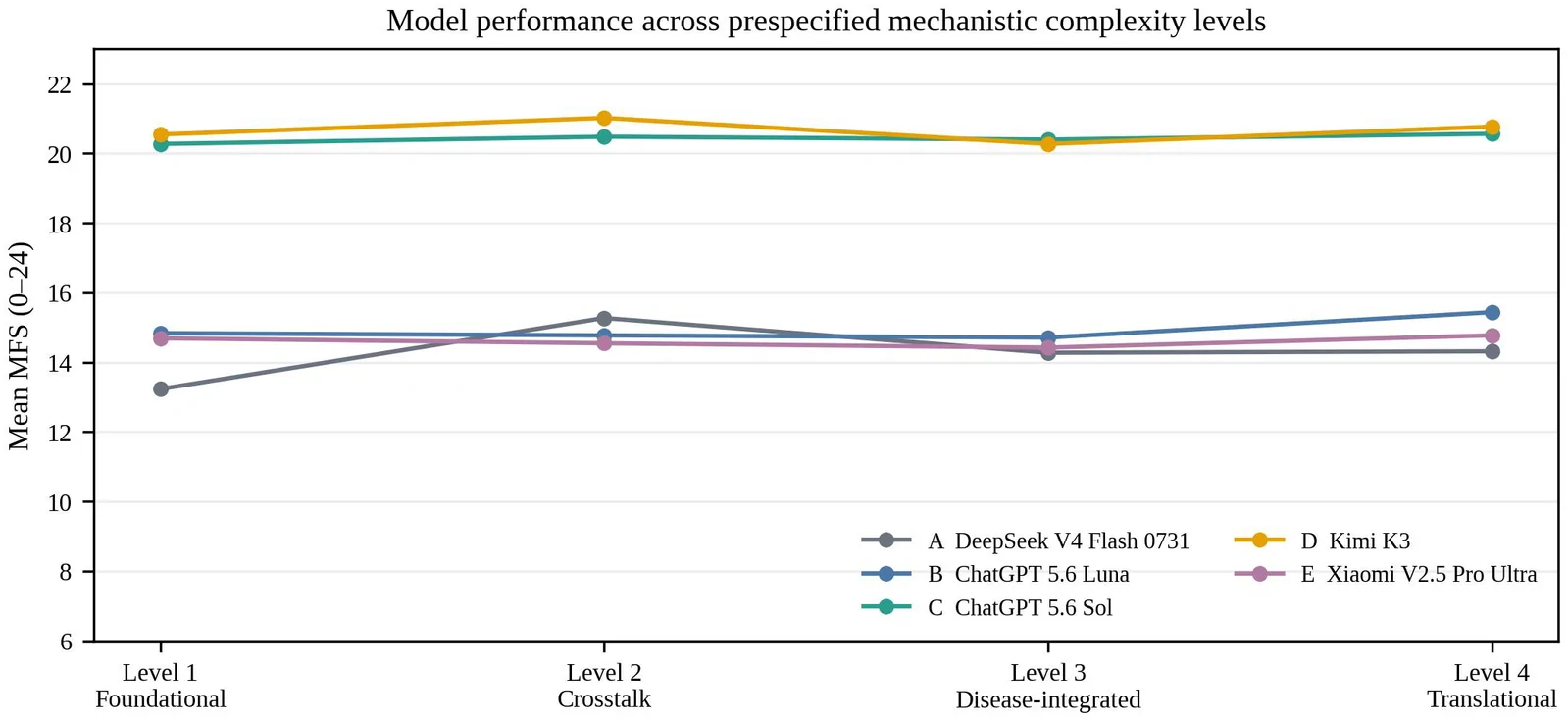
Model performance across the four prespecified mechanistic-complexity levels. Lines are descriptive mean MFS values. The formal model-by-complexity interaction was not significant (P = .328).

There was also no meaningful descriptive gradient by mucosal site. Mean MFS was 16.92 for intestinal, 17.00 for airway, and 17.02 for urogenital questions. The absence of a large site-level separation suggests that, within this question set, model identity explained more of the observed score structure than broad mucosal location.

### 3.6 Inter-rater agreement

Panel-specific concordance coefficients and absolute differences between paired ratings are summarized in **Table 4**.

**Table 4:** Agreement between paired expert ratings of total MFS.

Panel	Paired responses	Krippendorff α	Lin CCC	Spearman ρ	Mean absolute difference	Median absolute difference
Overall	480	0.709	0.709	0.807	2.48	1.0
English	120	0.856	0.856	0.838	2.26	2.0
Simplified Chinese	120	0.305	0.306	0.382	5.15	2.5
Traditional Chinese	120	0.735	0.734	0.739	1.88	1.0
Japanese	120	0.487	0.490	0.573	0.62	0.0

Krippendorff α was calculated for interval-scale MFS values. CCC, concordance correlation coefficient. The low Japanese coefficient estimates should be interpreted together with the very small absolute differences because the panel’s scores were strongly range-restricted.

### 3.7 Prespecified inferential tests

The prespecified omnibus tests for overall model effects and the model-by-language, model-by-complexity, and language-by-complexity terms are summarized in **Table 5**.

**Table 5:** Prespecified omnibus tests.

Analysis	Statistic	df	P value	Interpretation
Overall model effect	F=95.80	4, 23	<.001	Large between-model differences in MFS
Model × language (reviewer-standardized)	F=44.21	12, 191	<.001	Relative model performance changed with language
Model × complexity	F=1.14	12, 191	0.328	No statistically significant interaction detected in this question set
Language × complexity (reviewer-standardized)	F=10.32	12, 191	<.001	Small secondary heterogeneity across complexity levels

## 4 Discussion

### 4.1 Main interpretation

This study evaluated mechanistic explanations rather than exam-style answer selection, and the resulting pattern was not a simple ranking of five chatbots. Two models—ChatGPT 5.6 Sol and Kimi K3—formed a consistently higher-performing tier across all four language panels, while DeepSeek V4 Flash 0731, ChatGPT 5.6 Luna, and Xiaomi V2.5 Pro Ultra showed lower overall MFS values. At the same time, the lower-performing models changed relative position when the language changed. The central result is therefore dual: model identity remained a strong determinant of mechanistic fidelity, while the observed expression of that model advantage varied across language panels under the deployed web conditions.

The second important observation is that factual competence and integrated mechanistic reasoning were not equivalent. Even among the best models, crosstalk integration and evidence fidelity were lower than factual accuracy. Among the lower-scoring models, factual accuracy remained near 3/4 while crosstalk integration was below 2/4. This separation suggests that recognizing a mediator or cell function is substantially easier than organizing multiple correct elements into a time-, tissue-, and disease-specific causal account.

### 4.2 Language as a modifier of observed model-specific mechanistic performance

The language result requires a deliberately narrow interpretation. Because the English, Simplified Chinese, Traditional Chinese, and Japanese outputs were scored by different expert panels, the raw language means combine model behavior with panel-specific scoring severity and scale use. The near-ceiling Japanese scores and broad Simplified Chinese dispersion make that confounding visible rather than theoretical. We therefore did not interpret raw differences as evidence that Japanese output was intrinsically better or Simplified Chinese output intrinsically worse. Instead, the primary language-dependent analysis asked whether the relative position of models changed after each reviewer’s scoring distribution was standardized. Reviewer standardization should be read only as within-reviewer rescaling: a z score of +1 means one reviewer-specific standard deviation above that reviewer’s own mean, not one absolute MFS unit or a common cross-language effect size. It cannot remove panel-specific conceptual calibration or language-specific differences introduced by question rendering and web retrieval. The two-rater, range-restricted Japanese panel and the lower Simplified Chinese concordance therefore warrant particular caution when interpreting the interaction.

That interaction was large. The two strongest models remained strong in all four languages, but the remaining models moved substantially. Xiaomi V2.5 Pro Ultra was the lowest relative performer in English but the third-ranked model in Traditional Chinese and Japanese. DeepSeek V4 Flash 0731 was third in English but fifth in the other three panels. ChatGPT 5.6 Luna moved in the opposite direction relative to DeepSeek in the non-English panels. These shifts argue against treating “multilingual capability” as a single property attached to a model. They are more consistent with a conditional relationship among model, language, retrieved evidence, and the linguistic form in which a mechanistic explanation is constructed. The descriptive leave-one-language-out check yielded the same qualitative conclusion: omitting any single language panel did not eliminate rank reversals among Models A, B, and E across the remaining panels. This reduces concern that one panel alone created the observed instability, but it does not identify response language as the causal source.

Recent multilingual medical work provides a plausible context for this finding without establishing its cause. Multilingual medical pretraining and benchmark construction have documented uneven language coverage and have motivated language-specific evaluation (9–13). More recent expert-annotated benchmarks have likewise found that multilingual clinical error detection and long-form reasoning remain nonuniform (15, 16). Our design extends that problem from clinical questions to basic and translational immunological mechanisms. Because web search was enabled, the observed variation may reflect not only the underlying model but also language-dependent retrieval, source selection, synthesis, and confidence calibration. The present data cannot separate those components. Accordingly, the model-by-language interaction is best interpreted as language-associated variation in observed performance of the combined model–retrieval–interface system as deployed on the study date, not as an isolated property of the underlying model weights.

### 4.3 From factual recall to multicellular reasoning

Myeloid–mucosal biology is well suited to exposing the difference between factual recall and network reasoning. A credible answer must often state not only that a neutrophil produces a protease or that a macrophage performs efferocytosis, but also when that process becomes beneficial, when it becomes damaging, which cell supplies the upstream signal, and how the tissue returns toward homeostasis. The six-domain MFS was designed to make those distinctions visible. The crosstalk and duality dimensions specifically penalized explanations that reduced complex myeloid programs to static “pro-inflammatory” or “anti-inflammatory” labels.

The domain profiles suggest that this design captured information not contained in total fluency. Models C and D were both strong overall, yet they arrived there differently. Model C showed a modest advantage in completeness and tissue-specific organization, whereas Model D had higher protective–pathogenic duality and evidence fidelity. The difference was too small to separate their total MFS after multiplicity correction, but it matters conceptually: two systems can reach similar global scores while offering different kinds of scientific explanation. This is one reason that a single automated similarity metric or one overall correctness label would have been insufficient for the present task.

The nonsignificant model-by-complexity interaction should be interpreted as an absence of a statistically detected interaction in the present question set, not as evidence that model performance is invariant to complexity. We had expected increasingly integrative questions to produce a pronounced divergence, but the descriptive trajectories of the leading models remained relatively stable from foundational questions to translational reasoning. This does not mean Level 4 questions were biologically simple, nor does it validate the four categories as psychometric difficulty levels. Rather, within these 24 prespecified conceptual strata, we did not detect sufficient heterogeneity to conclude that increasing requested integration reordered the models. The result also cautions against assuming that a benchmark becomes more discriminative merely by labeling questions “complex.” The discriminative content of a prompt depends on the specific mechanisms it demands and on the scoring dimensions used to evaluate the answer.

### 4.4 Mucosal context and biological plausibility

The absence of a large intestinal, airway, or urogenital site gradient should not be interpreted as biological equivalence among these tissues. Airway macrophages respond to epithelial alarmin environments such as TSLP and IL-33 (18), bladder tumors contain macrophage states that can suppress antitumor immunity (19), and recurrent urinary infection involves tissue-localized immune memory beyond simple acute neutrophil recruitment (20). These are mechanistically distinct settings. Our site-level result instead indicates that the balanced question set did not produce a broad systematic advantage for one mucosal compartment. Within each site, model identity and language-dependent relative performance remained the more conspicuous sources of variation.

### 4.5 Implications for multilingual biomedical AI evaluation

The practical implication is not that one language should be preferred for biomedical reasoning. It is that an LLM intended for multilingual scientific or clinical-support environments should be evaluated in the language in which the reasoning will actually be consumed. A high English score cannot establish that the same model will preserve the same ranking against competitors in Chinese or Japanese, even when the biological question is parallel. This is especially relevant when the output is not a short answer but an explanation that combines several cell types, uncertain evidence, and trade-offs between host defense and tissue injury.

The study also supports a broader shift in benchmark design. Multiple-choice accuracy remains useful, but mechanistic biomedical evaluation benefits from explicit expert criteria and long-form outputs, as reflected in recent physician-validated and expert-annotated benchmarks (14–17). A useful next generation of benchmarks could combine structured mechanistic checklists, source-level citation auditing, retrieval provenance, and repeated generations. Such designs would help distinguish whether an error arises from missing knowledge, flawed biological synthesis, unstable retrieval, or language-specific expression.

Evaluation for deployment should also extend beyond mechanistic fidelity. The MFS did not score readability, concision, empathy, or audience accessibility, so the present data cannot establish whether greater mechanistic depth trades off against usable communication. For educational or clinical-support workflows, high-fidelity outputs should be treated as AI-generated drafts, paired with source provenance and explicit uncertainty, and subjected to language-matched domain-expert review before they inform high-stakes decisions; specialty-specific comparative work in some fields similarly supports an adjunct rather than replacement role for current LLMs (21). These safeguards are particularly important because the present study did not evaluate clinical outcomes or patient safety.

### 4.6 Strengths and limitations

Several features strengthen the study. The same 24 biological targets were represented in four languages; all five models were evaluated on the same date; web search was enabled for every model; every individual item was generated in a fresh session; and the response set covered three mucosal sites and four prespecified levels of mechanistic complexity. Model identity was blinded during rating. Twenty senior clinician-experts contributed, and every response received two independent evaluations. The MFS separated factual accuracy from completeness, crosstalk, tissue specificity, functional duality, and evidence calibration rather than collapsing all features into a single holistic impression.

The limitations are equally important. First, expert panels were nested within language. Reviewer standardization reduces differences in average severity and scale use, but it cannot eliminate differences in conceptual calibration, specialty emphasis, or interpretation between panels. The model-by-language interaction is therefore evidence of language-associated variation in observed relative performance under this panel-nested design, whereas neither the standardized interaction nor the raw means identify an absolute or intrinsic language effect. Second, the question set contained only 24 items from one mechanistic domain. The findings should not be generalized to all immunology, molecular biology, or clinical reasoning tasks without external replication. The four complexity strata were conceptual categories of requested integration and were not independently validated as psychometric difficulty levels. Third, the study captured five specific model builds on one date. Web-enabled systems evolve quickly, and retrieval results can change even when the underlying model name is unchanged. Search was enabled but source-level retrieval logs were not standardized across platforms, so the experiment evaluates the deployed web-enabled system rather than a controlled common evidence base. Fourth, only one response was generated for each model–language–question combination. Within-model stochastic repeatability was not estimated. Fifth, the MFS was study specific and has not undergone independent psychometric validation outside this benchmark. Although overall agreement was acceptable, the Simplified Chinese panel showed relatively weak coefficient-based agreement, and the Japanese panel contained only two raters and substantial score range restriction. Public web interfaces may additionally apply unobserved system prompts, retrieval-ranking policies, and decoding settings that differ by platform. Future reproducibility studies should, where feasible, pair ecologically valid web-interface testing with frozen API or model snapshots and recorded sampling and retrieval settings. Sixth, question development was investigator-driven and did not include patient, caregiver, trainee, or other end-user co-design. That choice is consistent with a construct-focused mechanistic benchmark, but it limits ecological generalizability to spontaneous real-world questions, which may prioritize different mechanisms, wording, or communication needs. Seventh, the MFS was intentionally a scientific-fidelity instrument and did not assess readability, concision, or audience comprehension; no trade-off between mechanistic quality and accessibility can therefore be inferred from these data. Finally, the evaluation did not use a separately adjudicated gold-standard mechanism checklist or a citation-by-citation external verification process as an independent endpoint. Expert evidence-fidelity ratings capture whether claims were appropriately supported and qualified at the response level, but they do not identify every retrieved source or prove the validity of every literature citation generated by a model. Future work should combine expert scoring with source provenance and explicit mechanistic claim verification. Taken together, these limitations mean that the findings describe the five evaluated web-enabled systems, the present prompts, expert panels, benchmark, and study date; they do not establish a general property of all LLMs or all biomedical reasoning tasks.

## 5 Conclusion

Under these evaluation conditions, mechanistic fidelity was strongly model dependent and observed relative performance varied across language panels. Because response language was inseparable from panel composition and could also alter retrieval and linguistic realization, the study does not isolate an intrinsic causal effect of language on underlying model reasoning. ChatGPT 5.6 Sol and Kimi K3 consistently formed the highest-performing tier, but their strengths were distributed differently across completeness, crosstalk, functional duality, and evidence fidelity. Lower-ranked models did not retain a stable order across English, Simplified Chinese, Traditional Chinese, and Japanese. These findings support language- and deployment-specific evaluation of long-form biomedical explanations and scoring frameworks that test relationships among cells, tissues, disease states, and evidence, while restricting inference to the evaluated systems and benchmark rather than generalizing to all LLMs or biomedical reasoning tasks.

## Data Availability

The original contributions presented in the study are included in the article/**Supplementary Material**. Further inquiries can be directed to the corresponding authors.
