# Supplementary material for: Language-dependent variation in observed mechanistic performance of web-enabled large language models across myeloid–mucosal immune contexts: a blinded expert evaluation
Source: Front Immunol. 2026 Sep 15;17:1965554. doi: 10.3389/fimmu.2026.1965554 (PMC13619594; doi:10.3389/fimmu.2026.1965554)
Supplement: Supplementary file 1 [file DataSheet1.pdf]

Supplementary Appendix Table 1. Complete Multilingual Mechanistic Question Set and Model-Generated Responses Across Five Web-Enabled Large Language Models

Myeloid–Mucosal Immunity

I. Model A

English (EN)

Anonymous Model A | English

Anonymous Model A
English
24 standardized questions
Myeloid–Mucosal Immunity

Q01
Question
At the healthy intestinal mucosa, how do neutrophils contribute to barrier defense while interacting with resident macrophages and dendritic cells, and what mechanisms normally limit neutrophil-driven tissue injury?

Response
In healthy intestine, neutrophils are not abundant residents; they are rapidly recruited when epithelial cells or lamina-propria sentinels detect microbial penetration. Resident macrophages and dendritic cells sense danger through pattern-recognition receptors and, together with epithelium, generate chemokines such as CXCL1/CXCL2/CXCL8 and lipid cues that guide neutrophils across venules and then across epithelium. Once at the threatened surface, neutrophils phagocytose microbes, generate reactive oxygen species, release antimicrobial granule proteins, and can cooperate with macrophages by reducing the microbial burden that those longer-lived phagocytes subsequently clear. Dendritic cells mainly integrate microbial and tissue signals and shape downstream adaptive responses rather than acting as major partners in direct neutrophil killing.

Several layers normally keep this response spatially and temporally constrained. Healthy intestinal macrophages are relatively hyporesponsive to innocuous microbial products and produce IL-10, while epithelial barrier integrity, mucus, antimicrobial peptides, and compartmentalization reduce unnecessary recruitment. Neutrophils that have completed their task undergo apoptosis and are removed by macrophage efferocytosis; this switches macrophages toward IL-10, TGF-beta and pro-resolving lipid programs and limits continued chemokine production. Endogenous antiproteases, antioxidant systems, epithelial repair pathways, and lipid mediators such as lipoxins and resolvins further restrain proteases, oxidants, and continued trafficking. NET formation can be antimicrobial, but in healthy mucosa it must remain limited because extracellular histones, proteases, and oxidants can damage epithelium. Thus barrier defense depends on rapid, local neutrophil activity followed by macrophage-mediated clearance and resolution rather than sustained granulocyte activation.

Q02
Question
At the healthy intestinal mucosa, how do eosinophils contribute to homeostasis and communicate with macrophages or dendritic cells, and how do these roles differ from eosinophil functions during inflammation?

Response
Intestinal eosinophils are unusually abundant even in the absence of overt inflammation. Their homeostatic maintenance depends on tissue cues that include IL-5 and eotaxin-family chemokines, especially CCL11, and they can contribute to epithelial support, extracellular-matrix turnover, and regulation of local humoral immunity. Eosinophils can release cytokines, growth factors and lipid mediators that influence macrophage functional state and dendritic-cell activation; IL-4/IL-13-like signals can favor repair-associated macrophage programs, whereas TGF-beta and other mediators may affect stromal remodeling. Proposed effects on IgA-producing plasma cells and tolerance have substantial experimental support in some models but are not uniformly demonstrated across species and intestinal compartments.

During inflammation, the same cells can become strongly pathogenic. Increased recruitment and survival, often driven by IL-5, eotaxins and type 2 cytokines, raises the local concentration of eosinophil peroxidase, major basic protein, cationic proteins, reactive oxygen species and cysteinyl leukotrienes. These products can injure epithelium, increase permeability, activate or mature dendritic cells, and amplify macrophage cytokine production. Conversely, eosinophils may also contribute to resolution through production of pro-resolving lipid mediators and by supporting repair, so the phenotype is context dependent. The key distinction is therefore not simply the presence of eosinophils, but their activation state, density, tissue localization and the cytokine environment: low-level resident signaling can be compatible with homeostasis, whereas sustained degranulation and type 2 or mixed inflammatory activation can convert the same network into a tissue-damaging one.

Q03
Question
At the healthy respiratory mucosa and during an acute microbial challenge, how do neutrophils coordinate with alveolar or interstitial macrophages and dendritic cells to provide host defense while limiting collateral epithelial injury?

Response
At baseline, alveolar macrophages provide the first phagocytic and regulatory barrier in the distal lung, while interstitial macrophages and dendritic cells survey deeper tissue and relay antigenic information. During an acute microbial challenge, macrophage and epithelial pattern-recognition receptors induce TNF, IL-1-family cytokines, GM-CSF/G-CSF and neutrophil-attracting chemokines such as CXCL1, CXCL2 and CXCL8. Recruited neutrophils then deliver rapid phagocytosis, oxidative killing, degranulation and, when strongly activated, extracellular-trap formation. Dendritic cells capture antigen and migrate to draining lymph nodes, while locally produced cytokines from macrophages and neutrophils tune dendritic-cell maturation and the subsequent T-cell response.

Protection requires that this amplification loop shut down once microbes are controlled. Alveolar macrophages engulf apoptotic neutrophils, a process that suppresses additional pro-inflammatory cytokine output and promotes IL-10, TGF-beta and pro-resolving lipid mediators. Epithelial cells restore tight junctions and mucociliary function, and airway antiproteases and antioxidants buffer neutrophil elastase, myeloperoxidase

products and reactive oxygen species. Resolvins, lipoxins and related mediators reduce further neutrophil trafficking while preserving phagocytic clearance. If efferocytosis is impaired, bacterial products persist, or neutrophils undergo excessive NETosis or necrosis, proteases and histones can directly damage epithelium and perpetuate macrophage activation. Thus effective respiratory defense is a sequence of sentinel activation, short-lived neutrophil escalation, and macrophage-centered resolution rather than a continuously maximal inflammatory response.

Q04

Question

How do eosinophils interact with macrophages and dendritic cells in the airway mucosa under homeostatic conditions and during type 2 inflammation?

Response

Under homeostatic conditions, airway eosinophils are relatively sparse and are shaped by epithelial, stromal and macrophage-derived survival signals. They can exchange cytokines and lipid mediators with macrophages and dendritic cells, but the network is usually low amplitude. Macrophages remove apoptotic granulocytes and help maintain an anti-inflammatory surface environment, while dendritic cells sample inhaled antigen without necessarily driving strong T-cell priming.

Type 2 inflammation changes both cell number and function. Epithelial alarmins such as TSLP, IL-33 and IL-25 activate dendritic cells and type 2 innate lymphoid cells; dendritic cells promote Th2 differentiation, and IL-4/IL-13 induce chemokines and tissue programs that favor eosinophil accumulation. IL-5 supports eosinophil maturation and survival. Activated eosinophils release major basic protein, eosinophil peroxidase, leukotrienes, cytokines and growth factors that can injure epithelium, increase mucus production and modify dendritic-cell activation. In parallel, IL-4/IL-13 drive macrophages toward repair-associated, alternatively activated states that can limit acute injury but also contribute to mucus, fibrosis and remodeling when persistent.

The interaction is therefore bidirectional: dendritic-cell priming and macrophage-derived or epithelial signals shape eosinophils, while eosinophil mediators feed back on those antigen-presenting cells. Resolution depends on allergen withdrawal, loss of survival cytokines, eosinophil apoptosis and macrophage efferocytosis, together with regulatory T-cell and pro-resolving lipid pathways. Which eosinophil-to-dendritic-cell interactions are dominant in human airways remains context dependent, especially across asthma endotypes.

Q05

Question

In the urinary tract mucosa, how do neutrophils coordinate with monocytes or macrophages and dendritic cells during early antibacterial defense, and what mechanisms prevent excessive tissue injury?

Response

In early urinary-tract infection, urothelial cells and resident myeloid cells detect bacterial products through pattern-recognition pathways such as TLR signaling and rapidly produce IL-6, IL-1-family cytokines and neutrophil-attracting chemokines. Neutrophils are then recruited across the bladder microvasculature and urothelium, where they phagocytose bacteria, generate reactive oxygen species and deploy granule antimicrobials. Monocytes enter somewhat later and can differentiate into inflammatory or repair-oriented macrophages; these cells phagocytose bacteria and debris, sustain cytokine production when needed, and then remove dying neutrophils. Dendritic cells capture antigen and provide a bridge to adaptive immunity, although their exact contribution varies with urinary-tract site and infection model.

Excessive neutrophil activity is limited by rapid pathogen clearance, apoptosis and macrophage efferocytosis, which reduces chemokine production and promotes IL-10, TGF-beta and repair programs. Urothelial turnover and regeneration, antioxidant systems, protease inhibitors and restoration of impermeable junctions reduce collateral injury. At the same time, complete suppression of neutrophil recruitment would be harmful because extracellular bacteria can expand quickly in urine and along the mucosal surface. Tissue protection therefore relies on a calibrated sequence: strong early chemotaxis and bacterial killing, followed by removal of spent neutrophils and a macrophage shift from inflammatory signaling toward debris clearance and epithelial repair. Persistent bacterial reservoirs, uncontrolled NET formation, or failed efferocytosis can convert this protective response into chronic mucosal damage.

Q06

Question

In the bladder or lower urogenital mucosa, how can mast-cell mediators influence macrophage and dendritic-cell functions, and how might this network contribute to both host defense and inflammatory symptoms?

Response

Mast cells in the bladder and lower urogenital mucosa can rapidly release histamine, tryptase, TNF, prostaglandins, leukotrienes and other mediators after microbial, epithelial or neuroimmune stimulation. These products influence macrophages and dendritic cells through histamine receptors, protease-activated receptors and cytokine pathways. TNF and lipid mediators can promote leukocyte recruitment and dendritic-cell maturation, whereas the balance of histamine receptor signaling can either enhance or restrain cytokine production depending on receptor expression and local conditions. Mast-cell proteases can also alter extracellular matrix and epithelial junctions, indirectly changing the signals seen by antigen-presenting cells.

This network is protective when it increases vascular access, recruits phagocytes, improves microbial sensing and helps coordinate adaptive immunity. The same mediators can produce symptoms: histamine and eicosanoids increase vascular permeability, tryptase can activate PAR2 on epithelial and sensory-neural elements, and mast-cell products can increase neuropeptides and afferent excitability, contributing to urgency, pain and frequency. Macrophage-derived IL-1, TNF and other mediators can reinforce these vascular and neural effects, creating a feed-forward inflammatory circuit.

Resolution requires loss of the initiating stimulus, stabilization or desensitization of mast cells, clearance of debris and apoptotic cells by macrophages, and restoration of epithelial barrier function. IL-10, TGF-beta and pro-resolving lipid pathways can dampen the circuit. The relative importance of direct mast-cell-to-dendritic-cell signaling versus indirect effects through epithelium and nerves is disease dependent, so mechanisms inferred from infection models should not be assumed to explain every chronic bladder pain syndrome.

Q07

Question

During acute intestinal mucosal inflammation and subsequent resolution, how do neutrophils and macrophages influence each other through recruitment signals, cytokines, efferocytosis, lipid mediators, and tissue-repair programs?

Response

Acute intestinal inflammation is commonly initiated by epithelial and myeloid production of neutrophil-recruiting signals such as CXCL1/CXCL2/CXCL8, complement fragments and leukotriene B4. Activated macrophages produce TNF, IL-1-family cytokines and chemokines that recruit and prime neutrophils. In turn, neutrophil proteases, reactive oxygen species, alarmins and lipid mediators can amplify macrophage activation and recruit additional granulocytes. This positive feedback is useful while microbes are being contained but becomes destructive if it remains active after the initiating threat has fallen.

The transition to resolution is strongly linked to neutrophil apoptosis and macrophage efferocytosis. Engulfment of apoptotic neutrophils through receptors such as MerTK and related pathways suppresses inflammatory transcription and promotes IL-10, TGF-beta, LXR/PPAR-linked metabolic programs and production of specialized pro-resolving mediators. Lipoxin-, resolvin- and protectin-family mediators reduce further neutrophil influx while improving macrophage clearance functions. Macrophages then support tissue repair through growth factors, matrix remodeling, angiogenic signals and communication with epithelial and stromal cells.

Failure at any step can prolong disease: ongoing microbial leakage maintains chemokine production; necrotic rather than apoptotic neutrophil death releases DAMPs; defective efferocytosis leaves proteases and oxidants in the tissue; and inflammatory macrophages may continue to produce TNF and IL-1. Thus neutrophils and macrophages form a time-dependent circuit that first amplifies antimicrobial defense and then, if resolution pathways engage properly, actively converts the inflammatory lesion into a reparative one.

Q08

Question

How can eosinophil-derived mediators alter macrophage polarization and dendritic-cell activation in the intestinal mucosa, and how could these interactions shift the balance among tolerance, tissue repair, and inflammation?

Response

Eosinophils can influence intestinal macrophages through cytokines, lipid mediators, growth factors and granule products. IL-4- and IL-13-like signals can favor macrophage programs associated with scavenging, matrix remodeling and tissue repair, while TGF-beta can promote repair but also fibrosis if persistent. Eosinophil-derived lipid mediators can be either inflammatory or pro-resolving depending on the enzymatic context. Granule proteins and reactive oxygen species, by contrast, can act as danger signals and increase macrophage inflammatory activation when eosinophils degranulate extensively.

Dendritic cells are similarly sensitive to the surrounding eosinophil milieu. Cytokines and granule products can alter dendritic-cell maturation, costimulatory molecule expression and migration, thereby changing whether mucosal antigen presentation favors tolerance or effector T-cell responses. In a low-injury environment, epithelial integrity, retinoic-acid-related mucosal programs, regulatory cytokines and efficient apoptotic-cell clearance favor tolerogenic presentation. During helminth infection, food-allergic responses or other type 2 inflammation, increased eosinophil survival and degranulation can shift the same network toward Th2-biased immunity and barrier dysfunction.

The balance among tolerance, repair and inflammation therefore depends on activation state rather than a fixed eosinophil phenotype. Moderate eosinophil-derived repair signals may support barrier recovery, whereas sustained IL-4/IL-13, TGF-beta or granule release can produce maladaptive remodeling. Some proposed eosinophil effects on macrophage polarization and dendritic-cell instruction are well supported in animal models but are less completely resolved in human intestinal tissue, so causal importance is likely disease- and compartment-specific.

Q09

Question

During neutrophilic airway inflammation, how do neutrophils and macrophages influence each other through cytokines, lipid mediators, neutrophil extracellular traps, efferocytosis, and metabolic signals, and how can this crosstalk determine persistence versus resolution?

Response

In neutrophilic airway inflammation, macrophages and epithelium produce TNF, IL-1, GM-CSF and CXCL8-family chemokines that recruit and prolong the survival of neutrophils. Neutrophils return the signal through proteases, oxidants, leukotriene B4, alarmins and extracellular traps. NET-associated DNA, histones and granule enzymes can activate macrophage pattern-recognition and inflammasome pathways, increasing IL-1-beta and additional neutrophil chemotaxis. This creates a self-reinforcing circuit when microbial products or injured epithelium continue to provide background stimulation.

Metabolism influences the direction of the response. Highly glycolytic, HIF-1alpha-linked macrophage states tend to support inflammatory cytokine production, and metabolites such as succinate can reinforce this program. Neutrophils themselves depend heavily on glycolysis, which allows them to function in hypoxic mucus but can also sustain intense effector activity. In contrast, macrophage transition toward oxidative, lipid-handling and pro-resolving programs supports efferocytosis and repair.

The decisive event for resolution is efficient removal of apoptotic neutrophils. Efferocytosis suppresses inflammatory transcription and promotes IL-10, TGF-beta and specialized pro-resolving mediators, while also reducing the substrate available for secondary necrosis and NET release. Persistent inflammation is favored by delayed neutrophil apoptosis, excessive NETosis, impaired macrophage efferocytosis, ongoing infection or metabolic locking of macrophages in an inflammatory state. Thus the same neutrophil-macrophage crosstalk can be protective early and pathogenic when clearance and metabolic reprogramming fail.

Q10

Question

During allergic airway inflammation, how do mast cells and eosinophils, with basophils where relevant, interact with dendritic cells and macrophages to amplify or restrain type 2 immunity?

Response

Allergic airway inflammation begins when epithelial alarmins and allergen-activated dendritic cells promote type 2 immunity. Dendritic cells drive Th2 differentiation and interact with epithelial cytokines such as TSLP and IL-33. IgE-armed mast cells then respond rapidly to allergen by releasing histamine, PGD2, cysteinyl leukotrienes, TNF and proteases. These mediators increase vascular permeability and bronchoconstriction and help recruit eosinophils. Eosinophil survival is supported by IL-5, while eotaxin-family chemokines guide tissue accumulation; activated eosinophils release granule proteins, leukotrienes, cytokines and growth factors that further damage epithelium and amplify antigen-presenting-cell activation.

Basophils, where they are abundant enough to matter, provide an additional rapid source of IL-4 and can reinforce Th2 differentiation and IgE-associated circuits. Macrophages exposed to IL-4 and IL-13 acquire repair-associated phenotypes that remove debris and can restrain some inflammation, but chronic alternative activation also supports mucus production, fibrosis and remodeling. Mast-cell and eosinophil products can alter macrophage cytokines and dendritic-cell maturation, making the network highly bidirectional.

Restraint comes from regulatory T cells, IL-10, loss of allergen, decline of IL-5 and other survival cues, eosinophil apoptosis, macrophage efferocytosis and pro-resolving lipids. Mast-cell desensitization and restoration of epithelial barrier integrity also reduce amplification. In human asthma, the relative contribution of basophils versus mast cells and eosinophils varies by endotype, so the network should be viewed as modular rather than identical in every patient.

Q11

Question

During acute bacterial urinary tract infection, how do recruited neutrophils and monocyte-derived macrophages cooperate or compete in pathogen clearance, cytokine production, efferocytosis, and mucosal repair?

Response

During acute bacterial UTI, neutrophils provide the fastest high-capacity antimicrobial response. They follow urothelial and macrophage-derived chemokines into the infected mucosa and urinary lumen, where phagocytosis, oxidative killing and granule antimicrobials reduce extracellular bacterial numbers. Recruited monocytes differentiate into macrophages that can also phagocytose bacteria, but they are especially important for sustained cytokine production, clearance of damaged cells and coordination of repair. Both populations can produce TNF, IL-1-family cytokines and chemokines, so cooperation can become amplification if microbial burden remains high.

Competition is possible because the two cell types share chemotactic niches, nutrients and phagocytic targets, and excessive macrophage cytokine output can prolong neutrophil influx. However, a simple competitive model is less well established than a sequential one. As bacterial control improves, neutrophils undergo apoptosis and monocyte-derived or resident macrophages engulf them. Efferocytosis switches macrophages toward IL-10, TGF-beta, lipid-resolving pathways and tissue-repair programs, while urothelial cells regenerate and restore barrier function.

Pathology emerges when neutrophils undergo necrosis or excessive NET formation, when macrophages fail to clear them, or when bacteria persist in intracellular or epithelial reservoirs. In that setting, proteases, oxidants and cytokines cause mucosal injury that can exceed the damage caused directly by the pathogen. Effective defense therefore depends on early neutrophil dominance followed by macrophage-mediated resolution rather than prolonged simultaneous maximal activation of both compartments.

Q12

Question

In chronic bladder mucosal inflammation, how might mast-cell–macrophage crosstalk alter sensory signaling, vascular permeability, epithelial barrier function, and the persistence or resolution of inflammation?

Response

Mast cells and macrophages can form a neuroimmune-vascular circuit in chronically inflamed bladder mucosa. Mast-cell histamine, tryptase, prostaglandins, leukotrienes and nerve growth factor can directly or indirectly increase excitability of sensory afferents. Tryptase acting through protease-activated receptors and histamine acting through neuronal or epithelial receptors can promote neuropeptide release, urgency and pain. At the same time, these mediators increase endothelial permeability, facilitating leukocyte influx and edema.

Macrophages respond to mast-cell products and to epithelial DAMPs by producing TNF, IL-1, IL-6, chemokines and additional lipid mediators. These signals can further activate endothelium and sensory nerves and can weaken epithelial junctions. Barrier leakage then exposes deeper tissue to urinary solutes and microbial products, generating more DAMPs and reinforcing both mast-cell and macrophage activation. This provides a plausible mechanism for persistence even when the initiating insult is no longer dominant.

Resolution requires breaking this positive feedback. Macrophage efferocytosis and transition toward IL-10, TGF-beta and pro-resolving lipid programs can reduce cytokine output and support epithelial repair; restoration of urothelial junctions decreases stimulation by urinary contents. Mast-cell stabilization or loss of activating cues can reduce neurogenic and vascular signaling. The exact importance of this circuit differs among chronic bladder disorders, and mechanistic findings from infection, interstitial cystitis/bladder pain syndrome and experimental injury models are not fully interchangeable.

Q13

Question

In ulcerative colitis, how do neutrophil recruitment, neutrophil extracellular trap formation, neutrophil metabolism, and neutrophil–macrophage crosstalk contribute to mucosal injury versus inflammatory resolution?

Response

In ulcerative colitis, neutrophil influx is a hallmark of active mucosal disease. Epithelial and myeloid production of CXCL8-family chemokines, complement-derived signals and other chemoattractants drives transendothelial and transepithelial migration. Neutrophils kill microbes but also release reactive oxygen species, myeloperoxidase products, elastase and other proteases that can erode epithelium. NETs add extracellular DNA,

histones and granule enzymes; these structures can trap microbes but can also injure epithelial cells, activate macrophages and amplify thrombogenic or inflammatory signaling.

The inflamed colon is hypoxic and metabolically stressed. Neutrophils are strongly glycolytic and remain functional in low oxygen, while lactate, succinate and other local metabolites can shape macrophage and neutrophil activation. Persistent inflammatory macrophages generate TNF, IL-1 and chemokines that recruit additional neutrophils, creating a feed-forward loop. By contrast, when neutrophils undergo apoptosis and are efficiently engulfed, macrophages shift toward IL-10, TGF-beta, lipid-resolving and tissue-repair programs.

Thus neutrophil presence is not intrinsically equivalent to pathology. Controlled recruitment can protect a damaged barrier from invading bacteria, whereas sustained influx, NETosis, secondary necrosis and failed efferocytosis cause collateral injury. Resolution depends on reducing chemotactic drive, clearing apoptotic neutrophils, restoring epithelial integrity and reprogramming macrophages away from inflammatory cytokine production. The relative importance of NETs in human UC is supported by association and mechanistic studies but remains less certain than the central role of neutrophil recruitment itself.

Q14

Question

In Crohn’s disease, how can dysregulated interactions among neutrophils, monocyte-derived macrophages, and dendritic cells alter microbial handling, antigen presentation, inflammatory persistence, and epithelial repair?

Response

Crohn's disease can convert normally coordinated myeloid defense into a chronic inflammatory circuit. Genetic and environmental factors that impair microbial sensing, autophagy or barrier function can reduce efficient handling of translocated microbes. Neutrophils may be recruited inadequately at some early stages or excessively once inflammation is established; either pattern can leave persistent microbial material. Monocytes entering the mucosa differentiate into inflammatory macrophages that produce TNF, IL-1, IL-6 and IL-23, while dendritic cells process antigen and promote Th1/Th17-skewed adaptive responses.

This alters antigen presentation qualitatively as well as quantitatively. Persistent bacterial products keep dendritic cells and macrophages activated, increasing costimulation and IL-12/IL-23-family signaling rather than tolerogenic presentation. Neutrophil proteases, oxidants and DAMPs from damaged tissue further reinforce macrophage and dendritic-cell activation. In turn, cytokines and chemokines from these antigen-presenting cells sustain granulocyte recruitment, producing a self-perpetuating loop.

Repair also suffers. Inflammatory macrophages can inhibit epithelial restitution when TNF, IL-1 and reactive mediators remain high, while defective efferocytosis delays the switch to growth-factor and matrix-remodeling programs. Conversely, appropriately timed macrophage transition toward pro-resolving states supports epithelial and stromal repair. The contribution of individual pathways such as NOD2 or ATG16L1 varies between patients, so Crohn's disease is best understood as convergent failure of microbial containment, inflammatory shutoff and tissue repair rather than one uniform myeloid defect.

Q15

Question

In severe neutrophilic asthma, how do neutrophils, macrophages, and dendritic cells form self-sustaining inflammatory circuits, and what roles may neutrophil extracellular traps, inflammasome signaling, microbial signals, and corticosteroid resistance play?

Response

Severe neutrophilic asthma can be maintained by a circuit linking epithelial injury, macrophages, dendritic cells and neutrophils. Microbial products or damaged epithelium activate TLR and other innate pathways, leading macrophages and epithelial cells to release IL-1, TNF, CXCL8-family chemokines and growth factors that recruit and prolong neutrophil survival. Dendritic cells can reinforce Th17- or mixed inflammatory responses, generating IL-17 that further increases epithelial neutrophil chemokines. Neutrophils then release proteases, oxidants and DAMPs, feeding back onto macrophages and epithelium.

NETs may strengthen this loop because extracellular DNA, histones and granule enzymes can damage epithelium and activate pattern-recognition and inflammasome signaling. Macrophage NLRP3-related activation and IL-1-beta production are mechanistically plausible drivers of continued neutrophilic inflammation, particularly when infection, dysbiosis or particulate exposures persist. Microbial signals may therefore act as both triggers and maintainers, although not all neutrophilic asthma is infection driven.

Corticosteroid resistance can arise at several levels: Th17-dominant pathways are relatively less steroid sensitive; oxidative stress and p38 MAPK signaling can impair glucocorticoid-receptor function; and persistent microbial or inflammasome signaling can continue despite suppression of classic eosinophilic pathways. NET burden and defective macrophage efferocytosis may also maintain steroid-insensitive DAMP signaling. These mechanisms are supported to different degrees across experimental and clinical studies, so a single self-sustaining circuit should not be assumed in every severe neutrophilic asthma phenotype.

Q16

Question

In eosinophilic asthma, how do eosinophils, mast cells, and where relevant basophils interact with macrophages, dendritic cells, and airway epithelium to initiate, amplify, and resolve type 2 inflammation?

Response

Type 2 airway inflammation is commonly initiated when stressed epithelium releases TSLP, IL-33 and IL-25. These alarmins activate dendritic cells and ILC2s; dendritic cells prime Th2 cells, while ILC2s and Th2 cells supply IL-5 and IL-13. IL-5 promotes eosinophil maturation and survival, and eotaxins recruit eosinophils into the airway. Allergen-specific IgE arms mast cells, which rapidly release histamine, PGD2, cysteinyl leukotrienes and proteases after re-exposure. Basophils can add an early source of IL-4 and other type 2 mediators in settings where they are recruited.

The network then amplifies itself. Eosinophil granule proteins and oxidants injure epithelium, generating more alarmins; eosinophil and mast-cell lipid mediators increase bronchoconstriction, permeability and mucus. IL-4/IL-13 program macrophages toward alternatively activated states that clear debris and can support repair, but chronic activation can promote fibrosis and mucus metaplasia. Dendritic cells exposed to an alarmin-rich environment remain efficient at sustaining Th2 memory.

Resolution requires withdrawal or control of allergen, decline of alarmin and IL-5 signals, apoptosis of eosinophils, macrophage efferocytosis, regulatory T-cell activity and specialized pro-resolving mediators. Mast-cell desensitization and epithelial barrier restoration reduce recurrent triggering. Thus the same macrophage and eosinophil repair programs that are beneficial after an acute episode can become remodeling pathways when type 2 signaling is chronic.

Q17

Question

In recurrent urinary tract infection, how can prior infection reshape neutrophil and monocyte or macrophage responses at the bladder mucosa through trained immunity, tolerance, or tissue remodeling, and how strong is the evidence for each mechanism?

Response

Prior UTI can plausibly reshape later myeloid responses through at least three mechanisms. First, trained immunity can leave monocytes or their bone-marrow progenitors with metabolic and epigenetic changes that increase responses to later stimulation; altered chromatin accessibility and histone marks at inflammatory genes are well established in trained-immunity biology generally. Whether this occurs in a durable, bladder-specific form after human UTI is much less certain. Neutrophils are short lived, so persistent training would more likely involve progenitors or tissue cues than long-term memory in the same neutrophil population.

Second, repeated or intense pattern-recognition signaling can produce tolerance, with reduced TNF/IL-1 responses and altered metabolic programs on restimulation. Such dampening may protect tissue but could also delay bacterial clearance. Third, prior infection can remodel the bladder itself: epithelial turnover, stromal changes, altered innervation, vascular remodeling, residual intracellular bacterial reservoirs and persistent changes in chemokine production can all change the recruitment and behavior of neutrophils and monocytes during recurrence.

The evidence is strongest for infection-induced tissue remodeling and persistent epithelial or microbial changes in experimental UTI models. Evidence for innate memory or tolerance affecting subsequent bladder responses is mechanistically credible and supported by animal and ex vivo observations, but direct causal evidence in patients is more limited. Therefore recurrent UTI should not be attributed to a single trained-immunity mechanism; tissue state, bacterial persistence, host hormones, anatomy and prior antimicrobial exposure can all dominate the phenotype.

Q18

Question

In bladder cancer, how do tumor-associated neutrophils and macrophages interact within the mucosal tumor microenvironment to influence antigen presentation, T-cell activity, angiogenesis, invasion, and response to immunotherapy?

Response

Tumor-associated macrophages (TAMs) and neutrophils (TANs) can cooperate to create an immunosuppressive bladder-tumor microenvironment. Tumor- and myeloid-derived CCL2, CSF1, CXCL8 and related cues recruit and retain these cells. TAMs can express IL-10, TGF-beta, arginase activity, PD-L1 and other suppressive programs that reduce T-cell activation and antigen-presenting efficiency. TANs can add reactive oxygen species, arginase, proteases and suppressive mediators, while neutrophil-derived chemokines can recruit additional myeloid cells.

Both cell types can support angiogenesis and invasion. VEGF, MMPs and extracellular-matrix-remodeling enzymes increase vascular growth and tissue penetration, and NET-associated proteases or DNA scaffolds may facilitate tumor-cell adhesion and invasion in some contexts. Suppressive macrophages can also impair dendritic-cell function and reduce effective priming of cytotoxic T cells. Conversely, macrophages and neutrophils are plastic: inflammatory, interferon-responsive or phagocytic states can support antitumor activity, so their presence alone does not define function.

These states can influence immunotherapy. A highly suppressive myeloid compartment may exclude or inhibit T cells and reduce response to checkpoint blockade, whereas treatments that activate innate sensing or repolarize macrophages may improve antigen presentation and T-cell recruitment. In bladder cancer, intravesical immunotherapy additionally changes the mucosal environment directly. The key translational challenge is separating tumor-promoting myeloid states from antimicrobial and tissue-repair functions that remain important in the urinary tract.

Q19

Question

Which mechanistically plausible strategies could suppress pathogenic neutrophil recruitment or neutrophil-extracellular-trap-associated injury in ulcerative colitis while preserving antimicrobial defense and macrophage-mediated repair? Explain predicted benefits, trade-offs, and evidence gaps.

Response

A rational strategy in ulcerative colitis is to reduce excessive neutrophil trafficking or toxic effector release without eliminating the early antimicrobial response. Partial or locally delivered inhibition of CXCR2-family chemotaxis could reduce sustained neutrophil accumulation, but strong systemic blockade risks impaired bacterial defense and delayed wound containment. Targeting upstream epithelial or macrophage chemokine production may offer similar benefits but can be less selective because the same cytokines support broader host defense.

NET-associated injury could be attacked downstream. DNase-based degradation of extracellular DNA may dismantle NET scaffolds, while inhibition of PAD4, neutrophil elastase or myeloperoxidase could reduce NET formation or tissue toxicity. These approaches are mechanistically attractive, but NETs can trap microbes, so aggressive inhibition could increase bacterial dissemination. A complementary approach is to enhance macrophage efferocytosis and specialized pro-resolving mediators, which would shorten neutrophil residence while preserving phagocytic killing and actively support epithelial repair.

The most favorable design is therefore spatially and temporally selective: local colonic delivery, dosing during established inflammatory excess, or biomarkers that identify NET-high/CXCR2-high disease. Major evidence gaps include which neutrophil functions are truly causal in human UC, whether NET inhibition improves outcomes without infection risk, and whether chronic chemokine blockade interferes with mucosal healing. Preserving macrophage repair argues against indiscriminate depletion of myeloid cells and in favor of rebalancing recruitment, death and clearance.

Q20

Question

How could metabolic or epigenetic reprogramming of intestinal macrophages and granulocytes be used to restore mucosal homeostasis in inflammatory bowel disease, and what mechanisms could cause loss of efficacy or unintended immunosuppression?

Response

Metabolic and epigenetic reprogramming could restore intestinal homeostasis by shifting myeloid cells away from persistent inflammatory states rather than simply suppressing them. In macrophages, high glycolytic flux, HIF-1alpha signaling, succinate accumulation and inflammatory chromatin accessibility can support IL-1/TNF production. Promoting oxidative metabolism, mitochondrial fitness, itaconate-NRF2-like antioxidant pathways or PPAR/LXR-associated lipid handling could favor efferocytosis and repair. Epigenetic approaches that alter histone acetylation, methylation or BET-dependent enhancer activity could similarly reduce persistent inflammatory gene programs.

Granulocytes are more difficult targets because mature neutrophils are short lived, but their activation can still be changed by metabolic availability, hypoxia signaling, NADPH-oxidase substrate supply and chromatin-dependent NET programs. Reprogramming progenitors in bone marrow is conceptually possible but carries greater systemic risk. Local intestinal delivery or cell-selective nanoparticles would therefore be preferable to broad systemic manipulation.

Loss of efficacy can occur if ongoing microbial leakage, hypoxia or cytokine exposure continuously re-imposes the inflammatory state. Cellular heterogeneity also means that one metabolic pathway may be beneficial in macrophages but harmful in neutrophils or epithelium. Unintended immunosuppression is a major concern: excessive inhibition of glycolysis, epigenetic transcription or innate sensing could impair bacterial killing, antigen presentation and wound defense. Durable therapy therefore requires state-selective, reversible reprogramming that preserves antimicrobial functions and macrophage efferocytosis rather than globally silencing innate immunity.

Q21

Question

How might biologics that alter type 2 cytokine signaling reshape the eosinophil–mast-cell–macrophage–dendritic-cell network in airway mucosa, beyond simply reducing eosinophil counts?

Response

Biologics that alter type 2 cytokine signaling can rewire the airway myeloid network even when eosinophil counts change only modestly. Blocking IL-5 or its receptor reduces eosinophil maturation, survival and tissue persistence, thereby decreasing eosinophil-derived granule proteins, leukotrienes and cytokines that activate epithelium, macrophages and dendritic cells. Blocking IL-4/IL-13 signaling can reduce eotaxin production, mucus programs, IgE-supporting signals and alternative macrophage activation, so the effect extends to epithelial and macrophage phenotypes as well as eosinophils.

Upstream blockade of epithelial alarmins such as TSLP, and potentially IL-33 pathways, can reduce dendritic-cell conditioning and ILC2 activation before the cascade fully develops. This may secondarily reduce mast-cell recruitment or reactivity, Th2 memory stimulation and macrophage type 2 polarization. Dendritic cells receiving fewer alarmin and IL-4-family signals may present antigen in a less strongly type 2-promoting context, while macrophages may shift from chronic remodeling programs toward more balanced scavenging and repair.

These network effects can alter exacerbation thresholds, mucus production, barrier repair and responsiveness to environmental triggers independently of blood eosinophil count. However, the exact remodeling differs by drug target and asthma endotype. Long-lived mast cells, established tissue remodeling and non-type-2 inflammatory circuits may persist despite successful cytokine blockade, so cell counts alone are an incomplete biomarker of how the mucosal network has changed.

Q22

Question

What myeloid-directed strategies could interrupt neutrophil–macrophage inflammatory circuits in neutrophilic asthma while preserving antibacterial defense, and which proposed mechanisms are clinically established versus preclinical?

Response

The central therapeutic problem in neutrophilic asthma is that neutrophils are important for antibacterial defense. Strategies that simply deplete them are therefore unattractive. More selective approaches include dampening excessive CXCR2-dependent recruitment, limiting macrophage IL-1/TNF-driven chemokine production, improving macrophage efferocytosis, or inhibiting toxic neutrophil outputs such as NETs, elastase and excessive oxidant release. Metabolic or pro-resolving interventions could theoretically shorten the inflammatory phase without blocking phagocytosis.

Clinically, there is no broadly established myeloid-targeted therapy that reliably defines and treats neutrophilic asthma as a single entity. Macrolides have clinical evidence in selected severe asthma populations and can reduce exacerbations, but their effects are pleiotropic and include antimicrobial and immunomodulatory actions rather than a clean neutrophil-macrophage mechanism. CXCR2 antagonism has reached clinical testing and can lower airway neutrophils, yet efficacy and infection/safety trade-offs have limited adoption. Broad IL-1, IL-17 or TNF blockade has not become standard therapy for this phenotype.

More selective inflammasome inhibition, NET/PAD4 targeting, macrophage metabolic reprogramming, specialized pro-resolving mediators and cell-specific delivery remain mainly preclinical or early translational concepts. A successful strategy will likely require endotyping: patients with infection-driven neutrophilia should not receive the same intervention as those with sterile NET- or inflammasome-dominant inflammation. Preserving antibacterial defense therefore depends on targeting pathological persistence and effector excess rather than neutrophil presence itself.

Q23

Question

Could trained-immunity or mucosal-vaccine approaches beneficially reprogram monocytes or macrophages and neutrophils to reduce recurrent urinary tract infection? Explain the proposed mechanisms, potential tissue-specific risks, and current evidence limitations.

Response

Trained-immunity and mucosal-vaccine approaches are plausible ways to reduce recurrent UTI because they could improve the speed and quality of local innate responses before a large bacterial burden develops. A trained-immunity strategy would aim to reprogram monocyte progenitors or

tissue macrophages through metabolic and epigenetic changes so that later bacterial sensing produces faster phagocytosis and appropriately scaled cytokine responses. Neutrophil benefit would likely be indirect, through better progenitor output, chemokine timing and macrophage instruction, because mature neutrophils are short lived.

Mucosal vaccination could add pathogen-specific antibody and T-cell memory while also altering epithelial and myeloid readiness. Ideally, local IgA/IgG and resident lymphocyte responses would reduce bacterial attachment, allowing neutrophils to clear a smaller inoculum and limiting tissue-damaging recruitment. Bacterial-lysate and mucosal immunization concepts provide proof of principle, but formulations, routes and patient populations are heterogeneous and robust mechanistic correlates are still limited.

The risks are tissue specific. Excessive training could increase sterile inflammation, pain, permeability or fibrosis; repeated innate stimulation could instead induce tolerance and blunt bacterial clearance. Broadly heightened myelopoiesis could also have systemic inflammatory effects. Current evidence therefore supports the concept more strongly than a standardized mechanism-based therapy. Human studies need to show durable protection, define whether macrophage or progenitor reprogramming actually occurs in the bladder axis, and demonstrate that recurrent infection falls without increasing chronic inflammatory symptoms.

Q24

Question

How could selective reprogramming of tumor-associated macrophages and neutrophils improve antitumor immunity in bladder cancer while minimizing disruption of protective urinary mucosal immunity? Compare mechanistic approaches and key translational barriers.

Response

Selective reprogramming of tumor-associated macrophages and neutrophils could improve bladder-cancer immunity by converting suppressive myeloid cells into phagocytic, inflammatory or T-cell-supporting states while leaving protective urinary mucosal defense largely intact. For macrophages, candidate mechanisms include blocking CSF1/CSF1R or CCL2-dependent recruitment, inhibiting PI3K-gamma-like suppressive signaling, disrupting CD47-SIRPalpha antiphagocytic checkpoints, or activating innate pathways such as TLR/STING to enhance antigen presentation and inflammatory cytokines. Reprogramming is generally preferable to complete macrophage depletion because macrophages are needed for bacterial clearance and repair.

For neutrophils, CXCR2-directed trafficking blockade could reduce tumor-promoting recruitment, while inhibition of NET formation, neutrophil elastase or TGF-beta-driven suppressive polarization might limit invasion and T-cell suppression. The risk is greater if these interventions are systemic, because urinary infection defense depends heavily on rapid neutrophil recruitment. Intravesical or tumor-targeted delivery is therefore especially attractive in bladder cancer: it may create high local exposure while preserving circulating and uninvolved-mucosal myeloid function.

Major barriers include profound TAM/TAN heterogeneity, plasticity over time, lack of stable human biomarkers that distinguish antitumor from antimicrobial states, and compensatory recruitment through parallel chemokine pathways. Tumor necrosis or barrier damage can also increase infection risk. Combination with checkpoint blockade or intravesical immunotherapy may be synergistic, but sequencing matters because over-suppressing myeloid inflammation could reduce antigen presentation. Translation will require spatial biomarkers and local-delivery strategies that separate tumor-associated myeloid programs from normal urinary mucosal immunity.

Japanese (JP)

Anonymous Model A | 日本語

Anonymous Model A

日本語

24 standardized questions

Myeloid–Mucosal Immunity

Q01

質問

健常な腸管粘膜において、好中球は常在マクロファージおよび樹状細胞と相互作用しながら、どのようにバリア防御に寄与しますか。また、好中球による組織障害を通常どのような機序が抑制していますか。

回答

健常腸管では好中球は恒常的に大量存在する細胞ではなく、上皮バリアを越える微生物や組織損傷が検知されたときに速やかに動員されます。上皮細胞、常在マクロファージ、樹状細胞がパターン認識受容体を介して危険シグナルを感知し、CXCL1、CXCL2、CXCL8 などのケモカインや脂質性走化シグナルを産生します。好中球は感染部位へ移動し、貪食、活性酸素、顆粒内抗菌分子、強い刺激下では細胞外トラップによって微生物量を減らします。マクロファージはより長時間にわたり微生物と細胞残骸を処理し、樹状細胞は抗原を取り込み、後続の適応免疫を調節します。

組織障害を抑えるには、炎症を適切な場所と時間に限定する仕組みが必要です。健常腸管マクロファージは無害な微生物刺激に対して比較的低反応性で、IL-10 などを産生します。粘液、抗菌ペプチド、上皮のタイトジャンクションも不要な好中球動員を減らします。役割を終えた好中球はアポトーシスに入り、マクロファージにエフェロサイトーシスされることで、IL-10、TGF-β、リポキシンやレゾルビンなどの収束プログラムが誘導されます。さらに抗プロテアーゼ、抗酸化系、上皮修復がエラスターゼや活性酸素の副次的障害を抑えます。したがって防御の本質は、短時間の局所的な好中球応答と、その後のマクロファージ主導の消炎･修復です。

Q02

質問

健常な腸管粘膜において、好酸球はどのように恒常性の維持に寄与し、マクロファージまたは樹状細胞と情報交換しますか。また、これらの役割は炎症時の好酸球機能とどのように異なりますか。

回答

好酸球は健常腸管粘膜にも比較的多く存在し、その維持には IL-5 や CCL11 などの好酸球関連シグナルが関与します。恒常状態では、好酸球はサイトカイン、脂質メディエーター、増殖因子を介して上皮維持、細胞外マトリックス調節、局所体液性免疫に寄与し得ます。IL-4/IL-13 様シグナルはマクロファージを貪食･修復寄りの状態へ傾け、TGF-βは組織修復や基質制御に関与し得ます。好酸球が IgA 産生細胞や免疫寛容を支えるという知見は一部の動物モデルで支持されていますが、種や腸管部位によって一貫しない点があります。

炎症時には同じ細胞が組織障害性に変化します。IL-5、エオタキシン、2 型サイトカインにより好酸球の動員と生存が増えると、好酸球ペルオキシダーゼ、主要塩基性蛋白、陽イオン性顆粒蛋白、活性酸素、システイニルロイコトリエンが大量に放出され、上皮障害や透過性亢進を起こし得ます。これらは樹状細胞の成熟やマクロファージの炎症性サイトカイン産生も促進します。一方で、好酸球は収束性脂質メディエーターや修復因子を産生する場合もあります。したがって、恒常性と炎症の違いは好酸球の存在そのものよりも、細胞数、活性化状態、局在、周囲のサイトカイン環境で決まります。

Q03

質問

健常な気道粘膜および急性の微生物刺激時に、好中球は肺胞または間質マクロファージ、樹状細胞とどのように協調して宿主防御を担いながら、上皮への二次的損傷を抑えますか。

回答

健常な肺では肺胞マクロファージが吸入微生物に対する第一の貪食･制御細胞であり、間質マクロファージや樹状細胞はより深部の組織監視と抗原伝達を担います。急性微生物刺激では、マクロファージと上皮細胞の TLR などが活性化され、TNF、IL-1 系サイトカイン、GM-CSF/G-CSF、CXCL1、CXCL2、CXCL8 などが産生されます。好中球はこれらのシグナルに従って動員され、貪食、酸化的殺菌、脱顆粒、必要に応じて NET 形成により病原体量を迅速に下げます。樹状細胞は抗原を取り込み、所属リンパ節へ移動して適応免疫を開始します。

上皮への二次的損傷を防ぐには、その増幅回路を感染制御後に停止させる必要があります。肺胞マクロファージがアポトーシス好中球をエフェロサイトーシスすると、炎症性サイトカインが減少し、IL-10、TGF-β、収束性脂質メディエーターが増えます。上皮はタイトジャンクションと粘液線毛機能を回復し、気道の抗プロテアーゼや抗酸化系が好中球エラスターゼ、MPO 由来酸化物、活性酸素を緩衝します。エフェロサイトーシス不全、持続感染、過剰な NET 形成があると、ヒストンや顆粒酵素が上皮を傷害し、再びマクロファージを活性化します。したがって、効果的な防御は「感知→一時的な好中球増幅→マクロファージ主導の収束」という時間的な連携です。

Q04

質問

恒常状態および 2 型炎症時の気道粘膜において、好酸球はマクロファージや樹状細胞とどのように相互作用しますか。

回答

恒常状態の気道では好酸球は比較的少なく、上皮、間質、局所髄系細胞からの生存シグナルにより制御されています。この段階では好酸球とマクロファージ、樹状細胞とのクロストークは低強度で、マクロファージはアポトーシス細胞の除去と低炎症環境の維持を担い、樹状細胞は強い T 細胞活性化を起こさずに吸入抗原をサンプリングします。

2 型炎症では、TSLP、IL-33、IL-25 などの上皮アラーミンが樹状細胞や ILC2 を活性化し、樹状細胞は Th2 分化を促進します。IL-4/IL-13 は上皮やマクロファージのプログラムを変え、IL-5 は好酸球の生存を延長します。活性化好酸球は主要塩基性蛋白、好酸球ペルオキシダーゼ、ロイコトリエン、サイトカイン、増殖因子を放出し、上皮障害、粘液増加、樹状細胞活性化を促します。一方、IL-4/IL-13 はマクロファージを修復関連の代替活性化状態へ導きますが、これが慢性化すると粘液化生、線維化、気道リモデリングに寄与します。収束には好酸球アポトーシス、マクロファージによる除去、制御性 T 細胞、収束性脂質メディエーターが重要です。

Q05

質問

尿路粘膜における初期の抗菌防御で、好中球は単球またはマクロファージ、樹状細胞とどのように協調しますか。また、過剰な組織障害を防ぐ機序は何ですか。

回答

尿路の初期細菌防御では、尿路上皮と常在髄系細胞が TLR などのパターン認識経路を介して細菌成分を検知し、IL-6、IL-1 系サイトカインと好中球走化性ケモカインを産生します。好中球は膀胱微小血管と尿路上皮を通過して感染部位へ入り、貪食、活性酸素、顆粒内抗菌分子によって細菌を排除します。単球はやや遅れて流入し、マクロファージへ分化して細菌と組織残骸を処理し、死んだ好中球を除去します。樹状細胞は抗原を取り込み、先天免疫から適応免疫への橋渡しをします。

過剰な障害は、病原体量が低下した後の速やかな収束によって抑えられます。好中球がアポトーシスに入り、マクロファージにエフェロサイトーシスされると、走化因子と炎症性サイトカインが減少し、IL-10、TGF-β、修復プログラムが上昇します。尿路上皮の再生、抗酸化系、プロテアーゼ阻害因子、タイトジャンクション回復も副次的障害を減らします。一方、好中球動員を過度に抑制すると細菌増殖を許すため、理想的なのは強い初期殺菌と、その後のマクロファージ主導の除去･修復への迅速な切り替えです。

Q06
質問
膀胱または下部泌尿生殖器粘膜において、肥満細胞由来メディエーターはマクロファージや樹状細胞の機能にどのような影響を与えますか。また、このネットワークは宿主防御と炎症症状の双方にどのように関与し得ますか。

回答
膀胱や下部泌尿生殖器粘膜の肥満細胞は、微生物、上皮損傷、神経刺激などに反応して、ヒスタミン、トリプターゼ、TNF、プロスタグランジン、ロイコトリエンを急速に放出します。これらはヒスタミン受容体、PAR、サイトカイン経路を介してマクロファージと樹状細胞に作用します。TNF や脂質メディエーターは白血球動員と樹状細胞成熟を促し、肥満細胞プロテアーゼは細胞外マトリックスや上皮接着を変化させることで、抗原提示細胞が受け取る危険シグナルを間接的に増やします。

このネットワークは感染時には血流と血管透過性を高め、貪食細胞を呼び込み、抗原認識を強化するため防御的です。しかし同じメディエーターは症状も引き起こします。ヒスタミンやエイコサノイドは血管漏出を増やし、トリプターゼは PAR2 や感覚神経に作用して疼痛、尿意切迫、頻尿を促進し得ます。マクロファージ由来 IL-1 や TNF はさらに神経･血管反応を強めます。収束には刺激の消失、肥満細胞活性の低下、マクロファージによる残骸除去、IL-10、TGF-β、収束性脂質メディエーターが必要です。慢性膀胱痛と感染ではこの回路の寄与が異なる可能性があります。

Q07
質問
急性の腸管粘膜炎症とその後の炎症収束過程において、好中球とマクロファージは、遊走･動員シグナル、サイトカイン、エフェロサイトーシス、脂質メディエーター、組織修復プログラムを介してどのように相互作用しますか。

回答
急性腸管炎症では、上皮やマクロファージから CXCL1、CXCL2、CXCL8、補体断片、ロイコトリエン B4 などが放出され、好中球が迅速に動員されます。マクロファージは TNF、IL-1、ケモカインを産生して好中球を活性化し、好中球はプロテアーゼ、活性酸素、DAMP、脂質メディエーターを放出してマクロファージをさらに刺激します。この正のフィードバックは微生物封じ込めには有効ですが、脅威が低下した後も続けば粘膜障害を増幅します。

収束への転換点は、好中球のアポトーシスとマクロファージによるエフェロサイトーシスです。MerTK などを介してアポトーシス好中球を取り込むと、マクロファージは炎症性転写を抑え、IL-10、TGF-β、LXR/PPAR 関連代謝、リポキシンやレゾルビンなどを増やします。同時に、上皮修復、血管･基質再構築を支える因子が増えます。好中球が壊死し、微生物漏出が続き、あるいはエフェロサイトーシスが破綻すると、DAMP、顆粒酵素、TNF/IL-1 が残存します。したがって、好中球とマクロファージは初期には共同で防御を増幅し、後期にはマクロファージが炎症を修復へ変換する時間依存的な回路を形成します。

Q08
質問
好酸球由来メディエーターは、腸管粘膜のマクロファージの機能状態や樹状細胞の活性化をどのように変化させますか。また、これらの相互作用は免疫寛容、組織修復、炎症のバランスをどのように変え得ますか。

回答
好酸球はサイトカイン、脂質メディエーター、増殖因子、顆粒成分を介して腸管マクロファージの機能状態を変化させます。IL-4/IL-13 様シグナルは貪食、マトリックス再構築、修復関連プログラムを促し、TGF-βは修復を助ける一方、長期化すれば線維化に寄与し得ます。好酸球由来脂質メディエーターには収束性のものもありますが、大量の顆粒蛋白や活性酸素は危険シグナルとしてマクロファージ炎症活性を高めます。

樹状細胞もこの好酸球環境の影響を受け、成熟、共刺激分子、移動能が変化し、抗原提示が寛容方向かエフェクター T 細胞方向かを左右します。低障害環境では上皮完全性、制御性サイトカイン、効率的な細胞除去が寛容を支えますが、寄生虫感染、食物アレルギー、その他の 2 型炎症では好酸球の増加と脱顆粒が Th2 様反応とバリア障害を強めます。したがって、寛容･修復･炎症のバランスは固定的な好酸球表現型ではなく、活性化強度と持続時間で決まります。人腸管における一部の直接的な好酸球→樹状細胞･マクロファージ作用は、なお疾患や部位に依存します。

Q09
質問
好中球性気道炎症において、好中球とマクロファージは、サイトカイン、脂質メディエーター、好中球細胞外トラップ、エフェロサイトーシス、代謝シグナルを介してどのように相互作用しますか。また、そのクロストークは炎症の持続と収束をどのように左右しますか。

回答
好中球性気道炎症では、マクロファージと上皮が TNF、IL-1、GM-CSF、CXCL8 などを産生し、好中球の動員と生存を促します。好中球はエラスターゼ、活性酸素、ロイコトリエン B4、DAMP、NET を放出し、マクロファージを再刺激します。NET 中の DNA、ヒストン、顆粒酵素はパターン認識系やインフラマソームを活性化し、IL-1βと追加の好中球走化シグナルを増やすため、持続性の自己増幅回路になり得ます。

代謝も炎症の方向を決めます。高解糖、HIF-1α、コハク酸に結びつくマクロファージ状態は炎症性サイトカイン産生を支え、好中球自体も強く解糖に依存するため低酸素の粘液中でも機能できます。収束の鍵はアポトーシス好中球の効率的なエフェロサイトーシスで、これにより IL-10、TGF-β、収束性脂質メディエーターが増え、マクロファージは酸化代謝、脂質処理、修復方向へ移行します。好中球アポトーシス遅延、過剰 NET 形成、持続感染、エフェロサイトーシス不全があると、炎症はより持続しやすくなります。

Q10
質問
アレルギー性気道炎症において、肥満細胞と好酸球、さらに該当する場合には好塩基球は、樹状細胞およびマクロファージとどのように相互作用して 2 型免疫を増幅または抑制しますか。

回答
アレルギー性気道炎症は、上皮アラーミンとアレルゲンを取り込んだ樹状細胞によって 2 型免疫が開始されることで進みます。樹状細胞は Th2 分化を促し、IgE で感作された肥満細胞は再曝露時にヒスタミン、PGD2、システイニルロイコトリエン、TNF、プロテアーゼを放出します。これにより血管透過性、気管支収縮、好酸球動員が増えます。IL-5 は好酸球生存を支え、好酸球顆粒蛋白、ロイコトリエン、サイトカイン、増殖因子は上皮障害と抗原提示細胞活性化をさらに増幅します。

該当する場合、好塩基球は早期 IL-4 供給源となり、Th2/IgE 回路を補強します。IL-4/IL-13 はマクロファージを代替活性化状態へ導き、急性期には清掃と修復を助ける一方、慢性化すると粘液、線維化、リモデリングを促します。抑制には制御性 T 細胞、IL-10、アレルゲン低下、IL-5 などの生存シグナル低下、好酸球アポトーシスとマクロファージによる除去、収束性脂質メディエーターが重要です。好塩基球、肥満細胞、好酸球の相対的寄与は喘息エンドタイプにより異なります。

Q11
質問
急性細菌性尿路感染において、動員された好中球と単球由来マクロファージは、病原体排除、サイトカイン産生、エフェロサイトーシス、粘膜修復の過程でどのように協調または競合しますか。

回答
急性細菌性尿路感染では、好中球が最も迅速な高容量の抗菌効果を担います。尿路上皮やマクロファージ由来ケモカインに従って感染粘膜と尿腔へ移動し、貪食、活性酸素、顆粒内抗菌分子により細菌数を減らします。動員単球から分化したマクロファージも細菌を貪食しますが、持続的なサイトカイン産生、損傷細胞除去、修復調整に特に重要です。両者は TNF、IL-1 系サイトカイン、ケモカインを産生するため、微生物負荷が高い間は協調的防御である一方、炎症を増幅することもあります。

「競合」は同じ走化ニッチ、栄養、貪食標的を共有する点では起こり得ますが、単純な競争モデルよりも時間的な分業の方が支持されています。感染が制御されると好中球はアポトーシスに入り、マクロファージのエフェロサイトーシスにより IL-10、TGF-β、修復プログラムが増えます。好中球の壊死や過剰 NET 形成、マクロファージによる除去不全、上皮内細菌リザーバーの残存があると、プロテアーゼ、活性酸素、サイトカインが粘膜障害を持続させます。したがって、早期好中球優位から後期マクロファージ主導の収束へ切り替わることが重要です。

Q12
質問
慢性膀胱粘膜炎症において、肥満細胞とマクロファージのクロストークは、感覚神経シグナル、血管透過性、上皮バリア機能、そして炎症の持続または収束をどのように変化させ得ますか。

回答
慢性膀胱粘膜炎症では、肥満細胞とマクロファージが神経･血管･免疫の正のフィードバックを形成し得ます。肥満細胞のヒスタミン、トリプターゼ、プロスタグランジン、ロイコトリエン、神経成長因子は感覚神経の興奮性を直接または間接に高めます。トリプターゼ-PAR2 やヒスタミン受容体を介した刺激は神経ペプチド放出、疼痛、尿意切迫、頻尿を促し、同時に内皮透過性を高めて白血球流入と浮腫を増やします。

マクロファージは肥満細胞メディエーターと上皮 DAMP に反応して TNF、IL-1、IL-6、ケモカイン、脂質メディエーターを産生し、血管と感覚神経をさらに刺激し、上皮接着も弱めます。バリアが破綻すると尿中溶質や微生物成分が深部組織へ到達し、回路が持続します。収束にはマクロファージのエフェロサイトーシス、IL-10、TGF-β、収束性脂質プログラム、上皮バリア修復、肥満細胞刺激の低下が必要です。感染、間質性膀胱炎/膀胱痛症候群、実験的損傷では、この回路の重要性が同一とは限りません。

Q13
質問
潰瘍性大腸炎において、好中球の動員、好中球細胞外トラップ形成、好中球代謝、ならびに好中球–マクロファージ間クロストークは、粘膜障害と炎症収束にそれぞれどのように寄与しますか。

回答
潰瘍性大腸炎の活動期では好中球流入が顕著です。上皮と髄系細胞の CXCL8 系ケモカイン、補体シグナルなどにより好中球が血管内皮と上皮を越えて移動します。好中球はバリアを越えた細菌を殺菌しますが、活性酸素、MPO 産物、エラスターゼ、その他の顆粒酵素は上皮を傷害します。NET 中の DNA、ヒストン、顆粒酵素は微生物を捕捉する一方で、上皮障害やマクロファージ活性化を起こし、炎症をさらに増幅し得ます。

炎症性大腸は低酸素･代謝ストレス環境であり、好中球は解糖依存性のためそこで機能を維持できます。コハク酸や乳酸などの代謝物も髄系細胞機能を変えます。炎症性マクロファージが TNF、IL-1、ケモカインを持続産生すると好中球流入が続きますが、好中球がアポトーシスに入り効率よく除去されると、マクロファージは IL-10、TGF-β、収束性脂質、修復プログラムへ移行します。したがって、好中球の存在自体ではなく、長期動員、NET 過剰、二次壊死、エフェロサイトーシス不全が粘膜障害に結びつきます。人 UC での NET の因果的重要性は、好中球動員そのものより不確実です。

Q14
質問
クローン病において、好中球、単球由来マクロファージ、樹状細胞の相互作用の異常は、微生物処理、抗原提示、炎症の持続、上皮修復をどのように変化させますか。

回答
クローン病では、微生物処理、バリア維持、炎症停止の協調が破綻し、髄系防御が慢性炎症回路へ変化します。NOD2 や ATG16L1 に関連する微生物感知･オートファジー異常、上皮バリア障害、持続的な菌体移行などにより微生物成分が残存します。好中球は時期によって初期動員不全または慢性期過剰動員を示し得ます。流入単球は炎症性マクロファージへ分化し TNF、IL-1、IL-6、IL-23 を産生し、樹状細胞は抗原を提示して Th1/Th17 様応答を強めます。

持続する細菌成分はマクロファージと樹状細胞の共刺激と IL-12/IL-23 系を維持し、寛容性の抗原提示を妨げます。好中球プロテアーゼ、活性酸素、組織 DAMP も抗原提示細胞を再刺激します。一方、高い TNF や IL-1、酸化ストレスは上皮再生を阻害し、エフェロサイトーシス不全はマクロファージが増殖因子･基質再構築へ切り替わるのを遅らせます。各経路の重要性は患者間で異なるため、クローン病は単一の髄系欠陥よりも、微生物封じ込め、炎症停止、組織修復の複合的失敗として捉える方が妥当です。

Q15
質問
重症好中球性喘息において、好中球、マクロファージ、樹状細胞はどのように自己持続的な炎症回路を形成しますか。また、好中球細胞外トラップ、インフラマソームシグナル、微生物由来シグナル、ステロイド抵抗性はどのような役割を果たし得ますか。

回答
重症好中球性喘息では、上皮障害、マクロファージ、樹状細胞、好中球が自己持続的な炎症回路を形成し得ます。微生物産物や損傷上皮が TLR などを刺激すると、マクロファージと上皮から IL-1、TNF、CXCL8 系ケモカインや増殖因子が放出され、好中球動員と生存が促進されます。樹状細胞は Th17 または混合型応答を強め、IL-17 がさらに上皮の好中球ケモカイン産生を増やします。好中球はプロテアーゼ、活性酸素、DAMP を放出し、マクロファージと上皮を再刺激します。

NET は DNA、ヒストン、顆粒酵素を通じて上皮を傷害し、パターン認識系やインフラマソームを活性化するため、この回路を増強し得ます。マクロファージの NLRP3 関連シグナルと IL-1β は、持続感染、ディスバイオーシス、粒子曝露がある表現型で特に妥当な機序です。ステロイド抵抗性には、Th17 経路の相対的低感受性、酸化ストレスや p38 MAPK によるグルココルチコイド受容体機能低下、持続する微生物･インフラマソーム刺激が関与し得ます。NET 負荷やエフェロサイトーシス不全もステロイド非感受性 DAMP シグナルを残す可能性がありますが、これらの重みは患者ごとに異なります。

Q16
質問
好酸球性喘息において、好酸球、肥満細胞、さらに該当する場合には好塩基球は、マクロファージ、樹状細胞、気道上皮とどのように相互作用して 2 型炎症を開始･増幅･収束させますか。

回答
好酸球性喘息では、刺激された気道上皮から TSLP、IL-33、IL-25 が放出され、樹状細胞と ILC2 が活性化されます。樹状細胞は Th2 分化を促し、ILC2 と Th2 細胞は IL-5、IL-13 を産生します。IL-5 は好酸球成熟と生存を支え、エオタキシンは気道への移動を促します。アレルゲン特異的 IgE をもつ肥満細胞は再曝露時にヒスタミン、PGD2、システイニルロイコトリエン、プロテアーゼを放出します。必要な状況では好塩基球が早期 IL-4 供給源となります。

増幅期には好酸球顆粒蛋白と活性酸素が上皮を傷害し、さらにアラーミンを放出させます。好酸球･肥満細胞の脂質メディエーターは気管支収縮、血管漏出、粘液を増やします。IL-4/IL-13 はマクロファージを代替活性化状態へ導き、残骸除去と修復には有益ですが、長期化すると線維化や粘液化生に寄与します。収束にはアレルゲンとアラーミンの低下、IL-5 などの生存シグナル低下、好酸球アポトーシス、マクロファージのエフェロサイトーシス、制御性 T 細胞、収束性脂質メディエーターが必要です。急性修復に有益な 2 型プログラムが慢性化するとリモデリングへ転じます。

Q17
質問
反復性尿路感染において、過去の感染は、訓練免疫、免疫寛容、または組織リモデリングを介して膀胱粘膜の好中球および単球 / マクロファージ応答をどのように再構成し得ますか。また、それぞれの機序を支持するエビデンスはどの程度確立されていますか。

回答
過去の尿路感染は少なくとも三つの経路で後続の髄系応答を変え得ます。第一に、訓練免疫では単球や骨髄前駆細胞に代謝･エピジェネティック変化が残り、次の刺激で貪食や炎症遺伝子応答が高まります。この原理自体は訓練免疫研究でよく支持されていますが、人の UTI 後に持続的かつ膀胱特異的な訓練状態が形成されるという直接証拠は限定的です。成熟好中球は短命なので、長期的効果は同じ好中球に記憶されるより、前駆細胞や組織シグナルを介して現れると考える方が妥当です。

第二に、反復する強いパターン認識刺激は耐性を誘導し、再刺激時の TNF や IL-1 反応を弱めることがあります。これは組織保護的ですが、細菌排除を遅らせる可能性があります。第三に、既往感染は上皮再生、間質、血管、神経、基礎ケモカイン産生、上皮内細菌リザーバーなど膀胱組織そのものを再構築します。実験的 UTI では感染後の組織リモデリングと持続的上皮変化の証拠が比較的強く、訓練免疫や耐性の寄与は機序的には妥当でも、人の反復性 UTI での直接因果証拠はまだ弱いといえます。

Q18

質問

膀胱がんにおいて、腫瘍関連好中球と腫瘍関連マクロファージは粘膜腫瘍微小環境内でどのように相互作用し、抗原提示、T 細胞活性、血管新生、浸潤、免疫療法への反応に影響しますか。

回答

膀胱がんの粘膜腫瘍微小環境では、腫瘍関連マクロファージ（TAM）と好中球（TAN）が免疫抑制ネットワークを共同形成し得ます。腫瘍や髄系細胞由来 CCL2、CSF1、CXCL8 などがこれらの細胞を動員･維持します。TAM は IL-10、TGF-β、アルギナーゼ、PD-L1 などを発現し、T 細胞活性化と効率的な抗原提示を低下させます。TAN は活性酸素、アルギナーゼ、プロテアーゼ、その他の抑制性メディエーターを加え、さらに髄系細胞を呼び込むことがあります。

両者は VEGF、MMP、基質分解酵素を通じて血管新生と浸潤を促し、状況によっては NET が腫瘍細胞の接着や浸潤を助ける足場になります。ただし TAM/TAN は可塑的で、インターフェロン応答性や高い貪食性をもつ状態では抗腫瘍作用を示し得ます。抑制性髄系環境が強いと T 細胞浸潤やチェックポイント阻害療法の効果が低下し得ますが、自然免疫感知の活性化やマクロファージ再プログラムは抗原提示を改善する可能性があります。膀胱がんでは膀胱内治療が粘膜免疫を直接変えるため、抗腫瘍効果と正常尿路の抗菌防御･修復を同時に考える必要があります。

Q19

質問

潰瘍性大腸炎において、病原性の好中球動員または好中球細胞外トラップ関連障害を抑えつつ、抗微生物防御とマクロファージによる修復を可能な限り温存するには、どのような機序的に妥当な戦略が考えられますか。予想される利点、トレードオフ、エビデンスの不足を説明してください。

回答

潰瘍性大腸炎では、好中球を全面的に除去するより、過剰な持続動員や最も毒性の高いエフェクター機能を選択的に抑える方が合理的です。CXCR2 依存性走化を部分的または局所的に抑えれば粘膜への好中球蓄積を減らせますが、強い全身阻害は細菌防御を損なう可能性があります。NET 関連障害には、細胞外 DNA の分解、PAD4、好中球エラスターゼ、MPO の阻害によって NET 形成や毒性を抑える戦略が考えられます。ただし NET は微生物捕捉にも働くため、過度な阻害は感染拡大リスクを伴います。

別の方向は、マクロファージのエフェロサイトーシスと収束性脂質メディエーターを強化し、好中球が殺菌後に速やかに退出するよう促すことです。これならマクロファージの修復機能を温存しやすくなります。大腸局所投与、炎症過剰期の短期投与、NET 高値/CXCR2 高値患者の選択などは理論上安全性を高めます。主要な未解決点は、人 UC でどの好中球機能が真に因果的か、NET 阻害が感染を増やさず臨床効果を示すか、慢性走化阻害が粘膜治癒を妨げないかです。髄系細胞の一括除去より「動員･死･除去･修復」の再調整が望まれます。

Q20

質問

炎症性腸疾患において、腸管マクロファージと顆粒球の代謝的またはエピジェネティックなリプログラミングを粘膜恒常性の回復にどのように利用できるでしょうか。また、効果消失や意図しない免疫抑制をもたらし得る機序は何ですか。

回答

代謝･エピジェネティック再プログラムは、自然免疫を全面的に抑えるのではなく、髄系細胞を持続炎症状態から清掃･修復可能な状態へ戻す目的で用いるべきです。マクロファージでは高解糖、HIF-1α、コハク酸蓄積、炎症エンハンサーの開放が IL-1 や TNF 産生を支えます。ミトコンドリア機能、酸化代謝、itaconate-NRF2 様抗酸化経路、PPAR/LXR 関連脂質処理を促すことで、エフェロサイトーシスと修復を高められる可能性があります。ヒストンアセチル化･メチル化や BET 依存エンハンサーを調節する手法も慢性炎症遺伝子を抑制し得ます。

成熟好中球は短命で長期再プログラムが難しい一方、解糖、低酸素応答、NADPH オキシダーゼ基質、NET 関連クロマチン状態はその機能を変えます。骨髄前駆細胞を直接変えると全身リスクが高くなるため、腸管局所または細胞選択的送達が望まれます。持続する微生物漏出、低酸素、サイトカイン曝露は炎症状態を再形成し、効果消失を招きます。また、解糖、転写、自然免疫感知を過度に抑えると細菌殺傷、抗原提示、創傷防御が低下します。したがって、可逆的かつ局所的で、細胞状態に選択的な再プログラムが理想です。

Q21

質問

2 型サイトカインシグナルを変化させる生物学的製剤は、単に好酸球数を減らすだけでなく、気道粘膜の好酸球–肥満細胞–マクロファージ–樹状細胞ネットワークをどのように再構成し得ますか。

回答

2 型サイトカインを標的とする生物学的製剤は、好酸球数を減らすだけでなく、気道髄系ネットワーク全体を変えます。IL-5 またはその受容体を遮断すると好酸球成熟、生存、組織滞留が低下し、顆粒蛋白、ロイコトリエン、サイトカインによる上皮･マクロファージ･樹状細胞への刺激が減ります。IL-4/IL-13 経路を遮断するとエオタキシン、粘液、IgE 支援プログラム、マクロファージの持続的代替活性化が低下するため、上皮やマクロファージの表現型も変わります。

より上流の TSLP、さらには IL-33 経路を抑えると、樹状細胞の条件付けと ILC2 活性化が減り、Th2 記憶、肥満細胞反応、マクロファージ 2 型偏倚まで二次的に弱まります。そのため血中好酸球数が大きく変わらなくても、粘液、バリア修復、環境刺激への反応閾値、細胞間フィードバックは変化し得ます。ただし長寿命の肥満細胞、既存の気道リモデリング、非 2 型炎症は残り得ます。したがって、好酸球数だけでは粘膜ネットワークの再構成を十分に評価できません。

Q22
質問
好中球性喘息において、抗菌防御を維持しながら好中球–マクロファージ炎症回路を遮断するために、どのような骨髄系細胞標的戦略が考えられますか。また、提案されている機序のうち、臨床的に確立しているものと前臨床段階にあるものを区別してください。

回答
好中球性喘息では、好中球が細菌防御に不可欠であるため、単純な好中球除去は望ましくありません。より選択的な戦略として、過剰な CXCR2 依存動員の抑制、マクロファージ IL-1/TNF によるケモカイン回路の低下、エフェロサイトーシス促進、NET、エラスターゼ、過剰活性酸素の選択的制限が考えられます。収束性脂質メディエーターや代謝再プログラムも、貪食を残したまま炎症持続時間を短縮する可能性があります。

臨床的には、「好中球-マクロファージ回路」を直接標的とする標準治療はまだ確立していません。マクロライドは一部の重症喘息で増悪低減の臨床エビデンスがありますが、抗菌作用と免疫調節を併せ持つ多面的治療であり、純粋な髄系標的ではありません。CXCR2 拮抗は臨床試験で気道好中球を減らしますが、効果と感染･安全性のトレードオフにより一般化していません。インフラマソーム阻害、PAD4/NET 標的、マクロファージ代謝再プログラム、収束性メディエーター、細胞特異的送達は主として前臨床または初期トランスレーショナル段階です。感染駆動型と無菌性 NET/インフラマソーム型を分けるエンドタイピングが重要です。

Q23
質問
訓練免疫または粘膜ワクチンのアプローチによって、単球 / マクロファージと好中球を有益にリプログラムし、反復性尿路感染を減らすことは可能でしょうか。想定される機序、組織特異的な潜在的リスク、現時点のエビデンスの限界を説明してください。

回答
訓練免疫と粘膜ワクチンは、細菌量が大きくなる前に局所防御を改善することで反復性 UTI を減らす可能性があります。訓練免疫では、単球前駆細胞や組織マクロファージに代謝･エピジェネティック変化を誘導し、次回感染時の貪食と適度な炎症応答を迅速化することが狙いです。成熟好中球は短命なので、長期的効果は前駆細胞出力、走化タイミング、マクロファージからの指令を介した間接効果と考える方が妥当です。

粘膜ワクチンは病原体特異的抗体と T 細胞記憶を作るだけでなく、上皮と髄系細胞の準備状態も変え得ます。理想的には局所 IgA/IgG や組織常在リンパ球が細菌付着を減らし、好中球が処理すべき菌量を小さくすることで組織障害性動員を抑えます。リスクは、訓練が強すぎることによる無菌炎症、疼痛、透過性亢進、線維化、あるいは反復刺激による耐性化と殺菌低下です。現時点では概念的妥当性の方が標準化された機序治療より強く、持続的予防効果、膀胱軸での実際の再プログラム、慢性炎症症状を増やさないことを人で示す必要があります。

Q24
質問
腫瘍関連マクロファージと好中球を選択的にリプログラムすることで、保護的な尿路粘膜免疫への影響を最小限にしつつ、膀胱がんの抗腫瘍免疫をどのように高められるでしょうか。機序的アプローチと主要なトランスレーショナル上の障壁を比較してください。

回答
腫瘍関連マクロファージと好中球の選択的再プログラムは、免疫抑制性の髄系細胞をより高い貪食性、抗原提示能、T 細胞支援能をもつ状態へ変えつつ、正常尿路の防御を残すことを目標とします。マクロファージでは CSF1/CSF1R や CCL2 依存動員の抑制、PI3Kγ様抑制シグナルの阻害、CD47-SIRPα抗貪食軸の解除、TLR/STING 様自然免疫刺激による抗原提示強化が候補です。完全除去より再プログラムの方が、感染防御と修復を温存しやすいと考えられます。

好中球では CXCR2 による腫瘍内流入を抑えたり、NET、好中球エラスターゼ、TGF-β関連抑制表現型を標的としたりすることで浸潤と T 細胞抑制を弱める可能性があります。ただし全身的に行うと尿路感染防御を損なうため、膀胱内投与や腫瘍標的ナノ送達など局所戦略が特に魅力的です。主要な障壁は TAM/TAN の高度な異質性と可塑性、抗腫瘍状態と抗菌状態を区別する安定なヒトバイオマーカーの不足、代替ケモカイン経路による補償、バリア損傷後の感染リスクです。チェックポイント阻害や膀胱内免疫療法との併用は有望ですが、髄系の抗原提示を過度に抑えない順序設計が必要です。

Simplified Chinese (SC)

Anonymous Model A | 简体中文

Anonymous Model A
简体中文
24 standardized questions
Myeloid–Mucosal Immunity

Q01
问题
在健康肠黏膜中，中性粒细胞如何在与驻留巨噬细胞和树突状细胞相互作用的同时参与屏障防御？哪些机制通常限制中性粒细胞介导的组织损伤？

回答
健康肠黏膜中的中性粒细胞数量通常不高，但当上皮屏障出现微生物穿越时，会被迅速募集。上皮细胞、驻留巨噬细胞和树突状细胞通过模式识别受体感知危险，并产生 CXCL1、CXCL2、CXCL8 等趋化因子以及脂质趋化信号，引导中性粒细胞从血管进入固有层并

跨越上皮。到达感染或微损伤部位后，中性粒细胞通过吞噬、活性氧、颗粒抗菌蛋白以及在强刺激下形成胞外诱捕网来降低微生物负荷；巨噬细胞则负责持续吞噬、清除残骸和整合炎症强度，树突状细胞更多承担抗原采集和后续适应性免疫启动。

限制组织损伤依赖多层制动。健康肠道巨噬细胞对无害菌源刺激相对低反应并产生 IL-10，完整的黏液层、抗菌肽和上皮连接也减少不必要的粒细胞募集。完成任务的中性粒细胞进入凋亡后，由巨噬细胞进行凋亡细胞清除，这会降低 TNF、IL-1 等炎症信号，并促进 IL-10、TGF-β以及脂氧素、消退素等促消退介质。组织中的抗蛋白酶、抗氧化系统和上皮修复程序还可缓冲弹性蛋白酶、髓过氧化物酶和活性氧造成的旁观者损伤。因此，健康屏障防御的核心是“快速、局部、短暂”的中性粒细胞效应，随后由巨噬细胞主导清除和炎症消退，而不是持续高水平的粒细胞激活。

Q02

问题

在健康肠黏膜中，嗜酸性粒细胞如何参与维持稳态并与巨噬细胞或树突状细胞进行通讯？这些作用与炎症状态下嗜酸性粒细胞的功能有何不同？

回答

嗜酸性粒细胞在健康肠黏膜中本来就相对丰富，其维持受到 IL-5 和 CCL11 等嗜酸性粒细胞趋化信号影响。稳态下，它们可通过细胞因子、脂质介质和生长因子参与上皮支持、基质更新和局部体液免疫，并能改变巨噬细胞和树突状细胞的功能状态。例如，IL-4/IL-13 样信号可推动巨噬细胞向清除和修复相关程序偏移，TGF-β则可参与组织修复和基质调节。关于嗜酸性粒细胞支持 IgA 浆细胞或耐受的作用，在部分动物模型中证据较强，但在人类不同肠段并不完全一致。

炎症时，同一类细胞可转为明显的组织损伤来源。IL-5、嗜酸性粒细胞趋化因子和 2 型细胞因子可增加其募集与存活，随后释放嗜酸性粒细胞过氧化物酶、主要碱性蛋白、阳离子颗粒蛋白、活性氧和半胱氨酰白三烯，导致上皮损伤、通透性升高，并可促进树突状细胞成熟和巨噬细胞炎症因子产生。另一方面，部分嗜酸性粒细胞也能产生促消退脂质介质并参与修复。因此，稳态与炎症的差别主要取决于细胞密度、激活状态、组织定位和细胞因子环境，而不是“是否存在嗜酸性粒细胞”这一单一因素。

Q03

问题

在健康呼吸道黏膜以及急性微生物攻击期间，中性粒细胞如何与肺泡或间质巨噬细胞及树突状细胞协同，在发挥宿主防御作用的同时限制对上皮的旁观者损伤？

回答

健康呼吸道中，肺泡巨噬细胞是最早接触吸入微生物的吞噬和调节细胞，间质巨噬细胞与树突状细胞则监测更深部组织并负责抗原传递。急性微生物攻击时，巨噬细胞和上皮细胞通过 TLR 等模式识别通路产生 TNF、IL-1、GM-CSF/G-CSF 以及 CXCL1、CXCL2、CXCL8 等中性粒细胞趋化因子。募集来的中性粒细胞通过吞噬、氧化杀菌、脱颗粒和必要时形成胞外诱捕网迅速降低病原体负荷；树突状细胞同时摄取抗原并迁移至引流淋巴结，启动适应性免疫。

限制附带上皮损伤的关键是及时“关停”这一放大回路。肺泡巨噬细胞清除凋亡中性粒细胞后，炎症因子产生下降，并转向 IL-10、TGF-β及促消退脂质介质。上皮重新建立紧密连接和黏液纤毛清除，气道抗蛋白酶和抗氧化系统抑制中性粒细胞弹性蛋白酶、髓过氧化物酶产物及活性氧。若凋亡细胞清除失败、病原体持续存在或 NET 形成过多，组蛋白和颗粒酶就会直接损伤上皮并再次激活巨噬细胞。因此，有效防御并非持续最大化炎症，而是先快速升级，再由巨噬细胞主导进入消退和修复。

Q04

问题

在稳态条件和 2 型炎症期间，嗜酸性粒细胞如何在气道黏膜中与巨噬细胞和树突状细胞相互作用？

回答

稳态气道中的嗜酸性粒细胞较少，其生存和定位受上皮、基质及局部髓系细胞信号调节。此时嗜酸性粒细胞与巨噬细胞、树突状细胞之间的通讯总体较弱，巨噬细胞主要负责清除凋亡细胞和维持低炎症环境，树突状细胞则在不过度启动 T 细胞的情况下采样吸入抗原。

2 型炎症时，TSLP、IL-33、IL-25 等上皮警报素激活树突状细胞和 ILC2，树突状细胞促进 Th2 分化，IL-4/IL-13 进一步改变上皮和巨噬细胞，IL-5 则延长嗜酸性粒细胞存活。激活的嗜酸性粒细胞释放主要碱性蛋白、嗜酸性粒细胞过氧化物酶、白三烯、细胞因子和生长因子，可损伤上皮、增加黏液并改变树突状细胞激活。与此同时，IL-4/IL-13 驱动巨噬细胞进入修复相关的替代激活状态，急性期可帮助清除和修复，但持续存在时也可促进黏液化生、纤维化和气道重塑。炎症消退依赖嗜酸性粒细胞凋亡、巨噬细胞清除、调节性 T 细胞和促消退脂质信号。

Q05

问题

在尿路黏膜中，早期抗菌防御期间，中性粒细胞如何与单核细胞或巨噬细胞及树突状细胞协同？哪些机制可防止过度组织损伤？

回答

尿路早期细菌感染时，尿路上皮和驻留髓系细胞通过 TLR 等模式识别通路快速产生 IL-6、IL-1 类细胞因子和中性粒细胞趋化因子。中性粒细胞随后穿过膀胱微血管和尿路上皮进入感染部位，通过吞噬、活性氧和颗粒抗菌分子清除细菌。单核细胞随后进入并分化为巨噬细胞，既可继续吞噬细菌，也负责清除受损细胞和死亡中性粒细胞；树突状细胞则摄取抗原并连接先天与适应性免疫。

防止过度损伤主要依靠感染负荷下降后的快速转段。中性粒细胞凋亡并被巨噬细胞进行凋亡细胞清除后，趋化因子和促炎因子下降，IL-10、TGF-β以及修复程序上升。尿路上皮更新、抗氧化体系、蛋白酶抑制剂和紧密连接恢复可进一步减少活性氧与颗粒酶造成的损伤。过度抑制中性粒细胞同样有风险，因为尿液环境中的细菌可迅速扩增。因此，最佳状态是强而短暂的早期杀菌，随后迅速转向巨噬细胞主导的清除和黏膜修复。

Q06

问题

在膀胱或下泌尿生殖道黏膜中，肥大细胞释放的介质如何影响巨噬细胞和树突状细胞的功能？这一网络如何同时参与宿主防御和炎症相关症状？

回答

膀胱或下泌尿生殖道的肥大细胞可在微生物、上皮损伤或神经刺激后迅速释放组胺、胰蛋白酶、TNF、前列腺素和白三烯等介质。这些信号可通过组胺受体、蛋白酶激活受体和细胞因子通路影响巨噬细胞与树突状细胞。TNF 和脂质介质可促进白细胞募集及树突状细胞成熟；肥大细胞蛋白酶还可改变细胞外基质和上皮连接，从而间接改变抗原呈递细胞接收到的危险信号。

该网络在感染时有利于增加血流和血管通透性、加速吞噬细胞募集并强化抗原识别，因此具有宿主防御价值。但同样的介质也可引起症状：组胺和花生四烯酸代谢物增加血管渗漏，胰蛋白酶可激活 PAR2 并影响感觉神经，促进尿急、疼痛和频尿；巨噬细胞产生的 IL-1、TNF 等又可进一步增强神经和血管反应。炎症消退依赖刺激消失、肥大细胞活性下降、巨噬细胞清除残骸以及 IL-10、TGF-β和促消退脂质介质。不同膀胱疾病中这条肥大细胞-巨噬细胞-神经轴的重要性并不完全相同。

Q07

问题

在急性肠黏膜炎症及其后的炎症消退阶段，中性粒细胞与巨噬细胞如何通过募集信号、细胞因子、凋亡细胞清除、脂质介质和组织修复程序相互影响？

回答

急性肠黏膜炎症中，上皮和巨噬细胞产生 CXCL1、CXCL2、CXCL8、补体片段和白三烯 B4 等募集信号，使中性粒细胞快速进入组织。巨噬细胞释放 TNF、IL-1 和趋化因子激活并延长中性粒细胞效应，而中性粒细胞释放的蛋白酶、活性氧、损伤相关分子和脂质介质又可反过来加强巨噬细胞炎症反应。这一正反馈在控制微生物时有益，但如果威胁已经下降仍持续存在，就会转化为黏膜损伤。

炎症从放大转向消退的核心事件是中性粒细胞凋亡和巨噬细胞的凋亡细胞清除。通过 MerTK 等受体吞噬凋亡中性粒细胞后，巨噬细胞会抑制促炎转录，增加 IL-10、TGF-β、LXR/PPAR 相关代谢程序和脂氧素、消退素等促消退介质，同时增强基质重塑、血管和上皮修复信号。若中性粒细胞发生坏死而非凋亡、微生物持续穿越屏障或凋亡细胞清除受损，DAMP、颗粒酶和 TNF/IL-1 就会持续存在。因此，中性粒细胞和巨噬细胞的关系本质上是时间依赖的：早期共同放大防御，后期由巨噬细胞将炎症主动转化为修复。

Q08

问题

嗜酸性粒细胞来源的介质如何改变肠黏膜巨噬细胞的功能状态和树突状细胞的激活？这些相互作用如何推动免疫耐受、组织修复与炎症之间的平衡发生变化？

回答

嗜酸性粒细胞可通过细胞因子、脂质介质、生长因子和颗粒产物改变肠黏膜巨噬细胞状态。IL-4/IL-13 样信号可促进吞噬、基质重塑和修复相关程序，TGF-β可参与修复但持续存在时也可能促纤维化；某些脂质介质具有促消退作用，而大量颗粒蛋白和活性氧则可作为危险信号，推动巨噬细胞进入炎症状态。

树突状细胞同样会受到嗜酸性粒细胞微环境影响。嗜酸性粒细胞来源的细胞因子和颗粒产物可改变树突状细胞成熟、共刺激分子和迁移，从而影响黏膜抗原呈递更偏向耐受还是效应 T 细胞反应。低损伤环境中，完整上皮、调节性细胞因子和有效清除有利于耐受；在寄生虫感染、食物过敏或其他 2 型炎症中，嗜酸性粒细胞数量和脱颗粒增加则会推动 Th2 样反应及屏障破坏。因而，耐受、修复和炎症之间的平衡取决于嗜酸性粒细胞的激活强度和持续时间，而不是一个固定的“促炎”或“抗炎”表型。人类肠道中部分直接因果关系仍具有情境依赖性。

Q09

问题

在中性粒细胞性气道炎症中，中性粒细胞与巨噬细胞如何通过细胞因子、脂质介质、中性粒细胞胞外诱捕网、凋亡细胞清除和代谢信号相互影响？这种串扰如何决定炎症持续还是消退？

回答

中性粒细胞性气道炎症中，巨噬细胞和上皮产生 TNF、IL-1、GM-CSF 及 CXCL8 等信号，促进中性粒细胞募集和存活。中性粒细胞随后释放弹性蛋白酶、活性氧、白三烯 B4、DAMP 和中性粒细胞胞外诱捕网，反过来刺激巨噬细胞。NET 中的 DNA、组蛋白和颗粒酶可激活模式识别及炎症小体通路，增加 IL-1β和新的中性粒细胞趋化信号，从而形成自我维持回路。

代谢状态会决定该回路走向。高糖酵解、HIF-1α和琥珀酸相关的巨噬细胞状态更有利于促炎细胞因子产生，而中性粒细胞本身高度依赖糖酵解，因此即使在缺氧黏液中也能持续发挥效应。炎症消退的关键是凋亡中性粒细胞被巨噬细胞有效清除；这会增加 IL-10、TGF-β和促消退脂质介质，并促使巨噬细胞转向氧化代谢、脂质处理和组织修复。若中性粒细胞凋亡延迟、NET 形成过多、持续感染或巨噬细胞清除功能下降，炎症就更可能长期维持。

Q10

问题

在过敏性气道炎症中，肥大细胞和嗜酸性粒细胞（在相关情境下包括嗜碱性粒细胞）如何与树突状细胞和巨噬细胞相互作用，从而放大或限制 2 型免疫反应？

回答

过敏性气道炎症首先由上皮警报素和过敏原激活的树突状细胞推动 2 型免疫。树突状细胞促进 Th2 分化；IgE 致敏的肥大细胞再遇过敏原时快速释放组胺、PGD2、半胱氨酰白三烯、TNF 和蛋白酶，造成支气管收缩、血管通透性增加并促进嗜酸性粒细胞募集。IL-5 维持嗜酸性粒细胞生存，嗜酸性粒细胞释放颗粒蛋白、白三烯、细胞因子和生长因子，可进一步损伤上皮并增强抗原呈递细胞活化。

在相关情境下，嗜碱性粒细胞可提供早期 IL-4 并加强 Th2 和 IgE 回路。IL-4/IL-13 使巨噬细胞偏向替代激活状态，急性期有助于清除和修复，但持续存在时可促进黏液、纤维化和重塑。限制 2 型免疫依赖调节性 T 细胞、IL-10、过敏原减少、IL-5 等存活信号下降、嗜酸性粒细胞凋亡和巨噬细胞清除，以及促消退脂质介质。不同哮喘内型中嗜碱性粒细胞、肥大细胞和嗜酸性粒细胞的相对权重不同，因此这一网络具有明显的模块化和情境依赖性。

Q11

问题

在急性细菌性尿路感染期间，募集到局部的中性粒细胞和单核细胞来源巨噬细胞如何在病原体清除、细胞因子产生、凋亡细胞清除和黏膜修复过程中协同或竞争？

回答

急性细菌性尿路感染中，中性粒细胞承担最快、最强的直接抗菌效应。它们沿尿路上皮和巨噬细胞产生的趋化梯度进入感染黏膜及尿腔，通过吞噬、活性氧和颗粒抗菌物质迅速降低细菌数量。单核细胞来源巨噬细胞也能吞噬细菌，但更重要的作用包括持续产生细胞因子、清除受损细胞和协调后续修复。两者都可产生 TNF、IL-1 及趋化因子，因此在病原负荷高时既协同杀菌，也可能共同放大炎症。

所谓“竞争”更多表现为共享趋化位点、营养和吞噬靶物，而不是简单互相抑制。感染控制后，中性粒细胞凋亡，巨噬细胞通过凋亡细胞清除将炎症程序切换为 IL-10、TGF-β和修复相关状态，并促进尿路上皮再生。若中性粒细胞发生坏死或过度形成 NET、巨噬细胞清除失败，或细菌在上皮细胞内形成持续储库，蛋白酶、活性氧和细胞因子会持续损伤黏膜。因此更符合证据的模式是：中性粒细胞早期占主导，随后由单核细胞/巨噬细胞承担清除和修复，而不是两类细胞长期同时处于最大激活状态。

Q12

问题

在慢性膀胱黏膜炎症中，肥大细胞与巨噬细胞之间的串扰可能如何改变感觉神经信号、血管通透性、上皮屏障功能以及炎症的持续或消退？

回答

慢性膀胱黏膜炎症中，肥大细胞和巨噬细胞可形成神经-血管-免疫正反馈。肥大细胞释放组胺、胰蛋白酶、前列腺素、白三烯和神经生长因子，可直接或间接提高感觉神经兴奋性；胰蛋白酶经 PAR2、组胺经相应受体还可促进神经肽释放，导致尿急、疼痛和频尿。同时，这些介质增加内皮通透性，使更多白细胞进入组织并形成水肿。

巨噬细胞受到肥大细胞介质和上皮 DAMP 刺激后产生 TNF、IL-1、IL-6、趋化因子和脂质介质，进一步激活血管和感觉神经，并削弱上皮连接。屏障变漏后，尿液中的溶质和微生物成分更容易刺激深层组织，形成持续性回路。炎症消退需要巨噬细胞通过凋亡细胞清除转向 IL-10、TGF-β和促消退脂质程序，同时修复尿路上皮连接并降低肥大细胞刺激。不同慢性膀胱疾病的驱动机制并不完全相同，因此感染模型中的发现不能直接等同于所有膀胱疼痛综合征。

Q13

问题

在溃疡性结肠炎中，中性粒细胞募集、中性粒细胞胞外诱捕网形成、中性粒细胞代谢以及中性粒细胞-巨噬细胞串扰如何分别促进黏膜损伤或炎症消退？

回答

溃疡性结肠炎活动期的典型特征之一是大量中性粒细胞进入黏膜。上皮和髓系细胞产生 CXCL8 等趋化因子、补体信号及其他趋化物，推动中性粒细胞跨内皮和跨上皮迁移。中性粒细胞可杀灭穿越受损屏障的细菌，但其活性氧、髓过氧化物酶产物、弹性蛋白酶和其他颗粒酶也会直接损伤上皮。NET 中的 DNA、组蛋白和颗粒酶可捕获微生物，却也能损伤上皮并激活巨噬细胞，形成额外炎症放大。

炎症结肠处于缺氧和代谢应激环境，中性粒细胞依赖糖酵解仍能持续效应，而琥珀酸、乳酸等代谢物也可改变髓系细胞状态。持续的炎症巨噬细胞产生 TNF、IL-1 和趋化因子，继续募集粒细胞；相反，当中性粒细胞凋亡并被有效清除后，巨噬细胞转向 IL-10、TGF-β、促消退脂质和组织修复程序。因此，中性粒细胞本身并不等于致病：短暂募集有助于抗菌，持续募集、NET 过多、继发坏死和清除失败才更容易造成黏膜损伤。NET 在人类 UC 中的因果贡献仍比“中性粒细胞募集”这一核心机制更不确定。

Q14

问题

在克罗恩病中，中性粒细胞、单核细胞来源巨噬细胞和树突状细胞之间失调的相互作用如何改变微生物处理、抗原呈递、炎症持续以及上皮修复？

回答

克罗恩病中，微生物处理、屏障和髓系细胞关停机制的失调可把正常防御转化为慢性炎症。与 NOD2、ATG16L1 等相关的微生物感知和自噬异常、上皮屏障缺陷或持续菌体穿越，都可能使病原成分长期存在。中性粒细胞在不同阶段可能出现早期募集不足或慢性期过

度募集；单核细胞进入黏膜后形成炎症型巨噬细胞，产生 TNF、IL-1、IL-6 和 IL-23；树突状细胞则持续摄取抗原并推动 Th1/Th17 样适应性反应。

长期存在的细菌成分使巨噬细胞和树突状细胞维持较强共刺激和 IL-12/IL-23 信号，而不是耐受性抗原呈递。中性粒细胞蛋白酶、活性氧和组织 DAMP 又可反过来增强抗原呈递细胞激活，形成持续回路。修复同样受影响：高水平 TNF、IL-1 和氧化应激会阻碍上皮恢复，而凋亡细胞清除不足则延迟巨噬细胞向生长因子和基质重塑程序转变。不同患者的主导通路并不相同，因此克罗恩病更适合被理解为“微生物控制、炎症终止和组织修复”多个环节的汇合性失败，而不是单一髓系细胞缺陷。

Q15
问题
在重度中性粒细胞性哮喘中，中性粒细胞、巨噬细胞和树突状细胞如何形成自我维持的炎症回路？中性粒细胞胞外诱捕网、炎症小体信号、微生物信号以及糖皮质激素抵抗可能分别发挥什么作用？

回答
重度中性粒细胞性哮喘可由上皮损伤、巨噬细胞、树突状细胞和中性粒细胞共同形成自我维持回路。微生物产物或损伤上皮激活 TLR 等先天通路，使巨噬细胞和上皮产生 IL-1、TNF、CXCL8 等趋化因子及生长因子，募集并延长中性粒细胞存活。树突状细胞可加强 Th17 或混合型适应性反应，IL-17 又促使上皮产生更多中性粒细胞趋化因子。中性粒细胞释放的蛋白酶、活性氧和 DAMP 则继续刺激巨噬细胞和上皮。

NET 可能进一步放大该回路，因为其 DNA、组蛋白和颗粒酶可损伤上皮并激活模式识别及炎症小体。巨噬细胞 NLRP3 相关通路及 IL-1β在部分表型中具有机制学合理性，特别是在持续感染、菌群失衡或颗粒暴露存在时。糖皮质激素抵抗可能与 Th17 通路相对低敏感、氧化应激和 p38 MAPK 损伤糖皮质激素受体功能，以及持续微生物/炎症小体信号有关。NET 负荷高和巨噬细胞凋亡细胞清除不足也可能维持类固醇不敏感的 DAMP 刺激。不过这些机制在人类患者中的权重并不一致，不能假定所有中性粒细胞性哮喘都由同一条回路驱动。

Q16
问题
在嗜酸性粒细胞性哮喘中，嗜酸性粒细胞、肥大细胞以及在相关情境下的嗜碱性粒细胞，如何与巨噬细胞、树突状细胞和气道上皮相互作用，从而启动、放大并最终促使 2 型炎症消退？

回答
嗜酸性粒细胞性哮喘通常由受刺激的气道上皮释放 TSLP、IL-33 和 IL-25 开始。警报素激活树突状细胞和 ILC2；树突状细胞促进 Th2 分化，ILC2 和 Th2 产生 IL-5 与 IL-13。IL-5 维持嗜酸性粒细胞成熟和存活，嗜酸性粒细胞趋化因子促进其进入气道。过敏原特异性 IgE 使肥大细胞致敏，再次暴露后肥大细胞迅速释放组胺、PGD2、半胱氨酰白三烯和蛋白酶；在相关情境下，嗜碱性粒细胞可补充早期 IL-4 信号。

放大阶段中，嗜酸性粒细胞颗粒蛋白和活性氧损伤上皮，产生更多警报素；嗜酸性粒细胞和肥大细胞脂质介质加重支气管收缩、血管渗漏和黏液。IL-4/IL-13 使巨噬细胞进入替代激活状态，可帮助清除残骸和修复，但长期存在时促进纤维化和黏液化生。炎症消退需要减少过敏原和警报素、降低 IL-5 等生存信号、嗜酸性粒细胞凋亡、巨噬细胞凋亡细胞清除、调节性 T 细胞和促消退脂质介质。也就是说，急性期有益的修复程序在 2 型信号长期持续时可转为气道重塑。

Q17
问题
在复发性尿路感染中，既往感染如何通过训练性免疫、免疫耐受或组织重塑改变膀胱黏膜中的中性粒细胞和单核细胞/巨噬细胞反应？目前对这些机制各自的证据强度如何？

回答
既往尿路感染可能通过三类机制改变再次感染时的髓系反应。第一，训练性免疫可在单核细胞或骨髓祖细胞中留下代谢和表观遗传改变，使后续刺激时的吞噬和炎症基因响应增强；这一机制在训练性免疫领域总体较为确立，但“人类 UTI 后形成持久、膀胱特异的训练状态”证据仍有限。中性粒细胞寿命很短，因此长期记忆更可能通过祖细胞或组织环境间接传递，而不是由同一批中性粒细胞保存。

第二，反复强烈的模式识别刺激也可能造成耐受，使再次刺激时 TNF、IL-1 等反应下降；这可保护组织，却可能延迟杀菌。第三，既往感染可重塑膀胱本身，包括上皮更新、基质和血管变化、神经重塑、趋化因子基线改变以及潜在的上皮内细菌储库。这些变化会直接影响下一次感染时中性粒细胞和单核细胞的募集。总体上，动物 UTI 模型对感染后组织重塑和持续上皮改变的证据较强；训练性免疫或耐受的机制合理性较高，但在人类复发性 UTI 中的直接因果证据仍相对不足。

Q18
问题
在膀胱癌中，肿瘤相关中性粒细胞和肿瘤相关巨噬细胞如何在黏膜肿瘤微环境中相互作用，并影响抗原呈递、T 细胞活性、血管生成、肿瘤侵袭以及免疫治疗反应？

回答
膀胱癌黏膜肿瘤微环境中，肿瘤相关巨噬细胞和中性粒细胞可共同形成免疫抑制网络。肿瘤及髓系细胞产生 CCL2、CSF1、CXCL8 等信号募集和维持这些细胞。肿瘤相关巨噬细胞可表达 IL-10、TGF-β、精氨酸酶和 PD-L1 等抑制程序，降低 T 细胞激活和有效抗原呈递；肿瘤相关中性粒细胞可补充活性氧、精氨酸酶、蛋白酶及其他抑制因子，并通过趋化因子募集更多髓系细胞。

两类细胞还可通过 VEGF、MMP 和基质重塑酶促进血管生成和侵袭，部分情境下 NET 也可为肿瘤细胞黏附和侵袭提供支架。另一方面，巨噬细胞和中性粒细胞具有高度可塑性，干扰素反应或强吞噬表型也可支持抗肿瘤免疫，因此“细胞数量多”并不等同于“功能一定

促肿瘤”。强免疫抑制性髓系环境可限制 T 细胞进入或削弱检查点抑制治疗，而激活先天感知或重编程巨噬细胞可能改善抗原呈递。膀胱癌的特殊之处在于局部膀胱灌注可直接改变黏膜免疫，因此治疗必须同时考虑抗肿瘤效应与正常尿路抗菌和修复功能。

Q19

问题

在溃疡性结肠炎中，哪些具有机制学合理性的策略可能抑制致病性中性粒细胞募集或中性粒细胞胞外诱捕网相关损伤，同时尽量保留抗微生物防御和巨噬细胞介导的修复？请说明预期获益、潜在权衡及证据缺口。

回答

在 UC 中，更合理的目标不是清除中性粒细胞，而是降低持续性过度募集或最具毒性的效应。部分或局部抑制 CXCR2 依赖的趋化可减少中性粒细胞在黏膜中的长期积聚，但系统性强力阻断可能削弱细菌防御。对 NET 损伤可考虑降解胞外 DNA、抑制 PAD4、弹性蛋白酶或髓过氧化物酶，从而减少 NET 形成或组织毒性；然而 NET 也有捕获微生物的作用，因此过度抑制可能增加感染或细菌扩散风险。

另一条思路是增强巨噬细胞凋亡细胞清除和促消退介质，使中性粒细胞在完成杀菌后更快退出，同时保留巨噬细胞修复。局部结肠递送、在炎症过度阶段短时给药，或仅选择 NET 高/CXCR2 高患者，理论上更有利于控制风险。关键证据缺口包括：人类 UC 中哪些中性粒细胞效应是真正因果驱动；NET 抑制是否能改善黏膜而不增加感染；长期趋化阻断是否会妨碍伤口封闭和上皮修复。因而，相比广泛髓系细胞耗竭，更合理的是重新平衡“募集-死亡-清除-修复”这一时间序列。

Q20

问题

如何利用肠道巨噬细胞和粒细胞的代谢或表观遗传重编程来恢复炎症性肠病中的黏膜稳态？哪些机制可能导致疗效丧失或非预期的免疫抑制？

回答

代谢或表观遗传重编程的目标应是把髓系细胞从持续炎症状态拉回能够清除和修复的状态，而不是全面关闭先天免疫。巨噬细胞中，高糖酵解、HIF-1α、琥珀酸积累及炎症增强子开放可支持 IL-1、TNF 产生；提高线粒体功能、促进氧化代谢、增强 itaconate-NRF2 样抗氧化通路以及 PPAR/LXR 相关脂质处理，可能有利于凋亡细胞清除和修复。通过调节组蛋白乙酰化、甲基化或 BET 依赖的增强子，也可能降低长期炎症基因表达。

粒细胞尤其是成熟中性粒细胞寿命短，直接长期重编程较困难，但其效应仍受糖酵解、缺氧、NADPH 氧化酶底物和 NET 相关染色质状态影响；若直接重塑骨髓祖细胞，则系统性风险更高。疗效可能因持续微生物泄漏、缺氧或细胞因子暴露而丧失，因为这些刺激会重新强迫细胞进入炎症程序。非预期免疫抑制则可能来自过度抑制糖酵解、表观遗传转录或先天感知，导致细菌杀灭、抗原呈递和伤口防御下降。因此，更理想的是局部、可逆、细胞状态选择性的重编程，而不是全身性“静音”髓系免疫。

Q21

问题

能够改变 2 型细胞因子信号的生物制剂，除降低嗜酸性粒细胞数量外，还可能如何重塑气道黏膜中的嗜酸性粒细胞-肥大细胞-巨噬细胞-树突状细胞网络？

回答

改变 2 型细胞因子信号的生物制剂可以在不只“减少嗜酸性粒细胞数量”的层面重塑气道网络。阻断 IL-5 或其受体会降低嗜酸性粒细胞成熟、存活和组织停留，从而减少其颗粒蛋白、白三烯和细胞因子对上皮、巨噬细胞和树突状细胞的持续刺激。阻断 IL-4/IL-13 信号则可降低嗜酸性粒细胞趋化因子、黏液和 IgE 相关程序，并减少巨噬细胞长期替代激活，因此会同时改变上皮和巨噬细胞表型。

更上游的 TSLP，乃至 IL-33 通路阻断，可减少树突状细胞预激活和 ILC2 启动，从网络起点降低 Th2 记忆刺激、肥大细胞反应和巨噬细胞 2 型极化。这样即使血液嗜酸性粒细胞计数变化不大，气道局部的黏液、屏障修复、过敏原阈值和细胞间反馈也可能显著改变。不过不同靶点的网络效应并不相同，长期存活的肥大细胞、既有气道重塑以及非 2 型炎症可在治疗后继续存在。因此，嗜酸性粒细胞计数只能反映网络的一部分，而不能完整代表黏膜免疫已经如何被重塑。

Q22

问题

在中性粒细胞性哮喘中，哪些针对髓系细胞的策略可能中断中性粒细胞-巨噬细胞炎症回路，同时保留抗细菌防御？这些策略所依据的机制中，哪些已有临床证据，哪些仍主要处于临床前阶段？

回答

中性粒细胞性哮喘的治疗难点在于中性粒细胞同时承担重要抗细菌防御，因此单纯耗竭并不理想。更合理的策略包括：降低过度 CXCR2 依赖募集，减弱巨噬细胞 IL-1/TNF 驱动的趋化因子回路，促进巨噬细胞凋亡细胞清除，或选择性限制 NET、弹性蛋白酶和过量氧化产物。促消退脂质介质和代谢重编程理论上也可缩短炎症持续时间，而不完全阻断吞噬和杀菌。

临床上，目前并没有一种广泛确立、专门针对“中性粒细胞-巨噬细胞回路”的标准治疗。大环内酯在部分重症哮喘人群中有降低急性加重的临床证据，但其机制多效，包括抗菌和免疫调节，并非纯髓系靶向。CXCR2 拮抗剂已进入临床研究并能降低气道中性粒细胞，但疗效和感染/安全权衡限制了常规应用。更直接的炎症小体抑制、PAD4/NET 抑制、巨噬细胞代谢重编程、促消退介质及细胞特异递送仍主要处于临床前或早期转化阶段。未来最关键的是内型分层：感染驱动型与无菌 NET/炎症小体驱动型患者不应采用相同策略。

Q23
问题
训练性免疫或黏膜疫苗策略是否可能通过有益地重编程单核细胞/巨噬细胞和中性粒细胞来减少复发性尿路感染？请解释其拟议机制、潜在的组织特异性风险以及当前证据的局限。

回答
训练性免疫和黏膜疫苗都有可能通过提前改善局部防御来降低复发性 UTI。训练性免疫的目标是使单核细胞祖细胞或组织巨噬细胞发生代谢和表观遗传改变，使再次遇到细菌时更快吞噬并产生适度的炎症反应。由于成熟中性粒细胞寿命短，对其长期影响更可能是间接的，例如改变骨髓输出、趋化时序或巨噬细胞对中性粒细胞的指导。

黏膜疫苗则可同时建立病原特异性抗体和 T 细胞记忆，并改变上皮与髓系细胞的“预备状态”。理想情况下，局部 IgA/IgG 和驻留淋巴细胞降低细菌黏附，使中性粒细胞面对更小的菌量，从而减少组织损伤性募集。潜在风险包括训练过强导致无菌炎症、疼痛、通透性增加或纤维化；反复先天刺激也可能反而诱导耐受，削弱杀菌。现阶段证据更支持“概念可行”而非已经建立统一机制治疗。仍需要人体研究证明保护作用持久，确认膀胱轴确实发生了巨噬细胞或祖细胞重编程，并证明复发减少的同时不会增加慢性炎症症状。

Q24
问题
如何通过选择性重编程肿瘤相关巨噬细胞和中性粒细胞来增强膀胱癌的抗肿瘤免疫，同时尽量减少对保护性尿路黏膜免疫的破坏？请比较不同机制策略及其主要转化障碍。

回答
选择性重编程肿瘤相关巨噬细胞和中性粒细胞的目标，是把免疫抑制性细胞转为更强吞噬、抗原呈递和 T 细胞支持状态，而不是把所有髓系细胞清除。巨噬细胞方面，可考虑阻断 CSF1/CSF1R 或 CCL2 募集、抑制 PI3Kγ样免疫抑制信号、解除 CD47-SIRPα“别吃我”轴，或用 TLR/STING 样先天激动方式增强抗原呈递。相比完全耗竭，重编程更有机会保留正常尿路的抗菌和修复功能。

中性粒细胞方面，可通过 CXCR2 限制肿瘤内募集，或抑制 NET、弹性蛋白酶及 TGF-β相关抑制表型，减少侵袭和 T 细胞抑制。但系统性干预会威胁尿路对细菌的快速中性粒细胞防御，因此膀胱灌注、肿瘤靶向纳米递送等局部策略尤其有吸引力。主要转化障碍包括 TAM/TAN 高度异质和可塑、缺乏稳定的人体标志物、替代趋化通路造成补偿性募集，以及治疗导致屏障损伤后增加感染风险。与检查点抑制或局部免疫治疗联用可能协同，但顺序必须避免过度削弱髓系抗原呈递和初始炎症。真正可行的方案需要空间生物标志物和局部递送来区分“肿瘤相关髓系程序”与“正常尿路保护性免疫”。

Traditional Chinese (TC)

Anonymous Model A | 繁體中文
Anonymous Model A
繁體中文
24 standardized questions
Myeloid–Mucosal Immunity

Q01
問題
在健康腸黏膜中，中性粒細胞如何在與駐留巨噬細胞和樹突狀細胞相互作用的同時參與屏障防禦？哪些機制通常限制中性粒細胞介導的組織損傷？

回答
健康腸黏膜中的中性粒細胞數量通常不高，但當上皮屏障出現微生物穿越時，會被迅速募集。上皮細胞、駐留巨噬細胞和樹突狀細胞通過模式識別受體感知危險，並產生 CXCL1、CXCL2、CXCL8 等趨化因子以及脂質趨化信號，引導中性粒細胞從血管進入固有層並跨越上皮。到達感染或微損傷部位後，中性粒細胞通過吞噬、活性氧、顆粒抗菌蛋白以及在強刺激下形成胞外誘捕網來降低微生物負荷；巨噬細胞則負責持續吞噬、清除殘骸和整合炎症強度，樹突狀細胞更多承擔抗原採集和後續適應性免疫啓動。

限制組織損傷依賴多層制動。健康腸道巨噬細胞對無害菌源刺激相對低反應並產生 IL-10，完整的黏液層、抗菌肽和上皮連接也減少不必要的粒細胞募集。完成任務的中性粒細胞進入凋亡後，由巨噬細胞進行凋亡細胞清除，這會降低 TNF、IL-1 等炎症信號，並促進 IL-10、TGF-β以及脂氧素、消退素等促消退介質。組織中的抗蛋白酶、抗氧化系統和上皮修復程序還可緩衝彈性蛋白酶、髓過氧化物酶和活性氧造成的旁觀者損傷。因此，健康屏障防禦的核心是“快速、局部、短暫”的中性粒細胞效應，隨後由巨噬細胞主導清除和炎症消退，而不是持續高水平的粒細胞激活。

Q02
問題
在健康腸黏膜中，嗜酸性粒細胞如何參與維持穩態並與巨噬細胞或樹突狀細胞進行通訊？這些作用與發炎狀態下嗜酸性粒細胞的功能有何不同？

回答
嗜酸性粒細胞在健康腸黏膜中本來就相對豐富，其維持受到 IL-5 和 CCL11 等嗜酸性粒細胞趨化信號影響。穩態下，它們可通過細胞因子、脂質介質和生長因子參與上皮支持、基質更新和局部體液免疫，並能改變巨噬細胞和樹突狀細胞的功能狀態。例如，IL-4/IL-13 樣信號可推動巨噬細胞向清除和修復相關程序偏移，TGF-β則可參與組織修復和基質調節。關於嗜酸性粒細胞支持 IgA 漿細胞或耐受的作用，在部分動物模型中證據較強，但在人類不同腸段並不完全一致。

炎症時，同一類細胞可轉為明顯的組織損傷來源。IL-5、嗜酸性粒細胞趨化因子和 2 型細胞因子可增加其募集與存活，隨後釋放嗜酸性粒細胞過氧化物酶、主要鹼性蛋白、陽離子顆粒蛋白、活性氧和半胱氨酰白三烯，導致上皮損傷、通透性升高，並可促進樹突狀細胞

成熟和巨噬細胞炎症因子產生。另一方面，部分嗜酸性粒細胞也能產生促消退脂質介質並參與修復。因此，穩態與炎症的差別主要取決於細胞密度、激活狀態、組織定位和細胞因子環境，而不是“是否存在嗜酸性粒細胞”這一單一因素。

Q03

問題

在健康呼吸道黏膜以及急性微生物攻擊期間，中性粒細胞如何與肺泡或間質巨噬細胞及樹突狀細胞協同，在發揮宿主防禦作用的同時限制對上皮的附帶損傷？

回答

健康呼吸道中，肺泡巨噬細胞是最早接觸吸入微生物的吞噬和調節細胞，間質巨噬細胞與樹突狀細胞則監測更深部組織並負責抗原傳遞。急性微生物攻擊時，巨噬細胞和上皮細胞通過 TLR 等模式識別通路產生 TNF、IL-1、GM-CSF/G-CSF 以及 CXCL1、CXCL2、CXCL8 等中性粒細胞趨化因子。募集來的中性粒細胞通過吞噬、氧化殺菌、脫顆粒和必要時形成胞外誘捕網迅速降低病原體負荷；樹突狀細胞同時攝取抗原並遷移至引流淋巴結，啓動適應性免疫。

限制附帶上皮損傷的關鍵是及時“關停”這一放大迴路。肺泡巨噬細胞清除凋亡中性粒細胞後，炎症因子產生下降，並轉向 IL-10、TGF-β及促消退脂質介質。上皮重新建立緊密連接和黏液纖毛清除，氣道抗蛋白酶和抗氧化系統抑制中性粒細胞彈性蛋白酶、髓過氧化物酶產物及活性氧。若凋亡細胞清除失敗、病原體持續存在或 NET 形成過多，組蛋白和顆粒酶就會直接損傷上皮並再次激活巨噬細胞。因此，有效防禦並非持續最大化炎症，而是先快速升級，再由巨噬細胞主導進入消退和修復。

Q04

問題

在穩態條件和第 2 型發炎期間，嗜酸性粒細胞如何在氣道黏膜中與巨噬細胞和樹突狀細胞相互作用？

回答

穩態氣道中的嗜酸性粒細胞較少，其生存和定位受上皮、基質及局部髓系細胞信號調節。此時嗜酸性粒細胞與巨噬細胞、樹突狀細胞之間的通訊總體較弱，巨噬細胞主要負責清除凋亡細胞和維持低炎症環境，樹突狀細胞則在不過度啓動 T 細胞的情況下採樣吸入抗原。

2 型炎症時，TSLP、IL-33、IL-25 等上皮警報素激活樹突狀細胞和 ILC2，樹突狀細胞促進 Th2 分化，IL-4/IL-13 進一步改變上皮和巨噬細胞，IL-5 則延長嗜酸性粒細胞存活。激活的嗜酸性粒細胞釋放主要鹼性蛋白、嗜酸性粒細胞過氧化物酶、白三烯、細胞因子和生長因子，可損傷上皮、增加黏液並改變樹突狀細胞激活。與此同時，IL-4/IL-13 驅動巨噬細胞進入修復相關的替代激活狀態，急性期可幫助清除和修復，但持續存在時也可促進黏液化生、纖維化和氣道重塑。炎症消退依賴嗜酸性粒細胞凋亡、巨噬細胞清除、調節性 T 細胞和促消退脂質信號。

Q05

問題

在尿路黏膜中，早期抗菌防禦期間，中性粒細胞如何與單核細胞或巨噬細胞及樹突狀細胞協同？哪些機制可防止過度組織損傷？

回答

尿路早期細菌感染時，尿路上皮和駐留髓系細胞通過 TLR 等模式識別通路快速產生 IL-6、IL-1 類細胞因子和中性粒細胞趨化因子。中性粒細胞隨後穿過膀胱微血管和尿路上皮進入感染部位，通過吞噬、活性氧和顆粒抗菌分子清除細菌。單核細胞隨後進入並分化為巨噬細胞，既可繼續吞噬細菌，也負責清除受損細胞和死亡中性粒細胞；樹突狀細胞則攝取抗原並連接先天與適應性免疫。

防止過度損傷主要依靠感染負荷下降後的快速轉段。中性粒細胞凋亡並被巨噬細胞進行凋亡細胞清除後，趨化因子和促炎因子下降，IL-10、TGF-β以及修復程序上升。尿路上皮更新、抗氧化體系、蛋白酶抑制劑和緊密連接恢復可進一步減少活性氧與顆粒酶造成的損傷。過度抑制中性粒細胞同樣有風險，因為尿液環境中的細菌可迅速擴增。因此，最佳狀態是強而短暫的早期殺菌，隨後迅速轉向巨噬細胞主導的清除和黏膜修復。

Q06

問題

在膀胱或下泌尿生殖道黏膜中，肥大細胞釋放的介質如何影響巨噬細胞和樹突狀細胞的功能？這一網路如何同時參與宿主防禦和發炎相關症狀？

回答

膀胱或下泌尿生殖道的肥大細胞可在微生物、上皮損傷或神經刺激後迅速釋放組胺、胰蛋白酶、TNF、前列腺素和白三烯等介質。這些信號可通過組胺受體、蛋白酶激活受體和細胞因子通路影響巨噬細胞與樹突狀細胞。TNF 和脂質介質可促進白細胞募集及樹突狀細胞成熟；肥大細胞蛋白酶還可改變細胞外基質和上皮連接，從而間接改變抗原呈遞細胞接收到的危險信號。

該網絡在感染時有利於增加血流和血管通透性、加速吞噬細胞募集並強化抗原識別，因此具有宿主防禦價值。但同樣的介質也可引起症狀：組胺和花生四烯酸代謝物增加血管滲漏，胰蛋白酶可激活 PAR2 並影響感覺神經，促進尿急、疼痛和頻尿；巨噬細胞產生的 IL-1、TNF 等又可進一步增強神經和血管反應。炎症消退依賴刺激消失、肥大細胞活性下降、巨噬細胞清除殘骸以及 IL-10、TGF-β和促消退脂質介質。不同膀胱疾病中這條肥大細胞-巨噬細胞-神經軸的重要性並不完全相同。

Q07
問題
在急性腸黏膜發炎及其後的發炎消退階段，中性粒細胞與巨噬細胞如何透過募集訊號、細胞激素、凋亡細胞清除、脂質介質和組織修復程序相互影響？

回答
急性腸黏膜炎症中，上皮和巨噬細胞產生 CXCL1、CXCL2、CXCL8、補體片段和白三烯 B4 等募集信號，使中性粒細胞快速進入組織。巨噬細胞釋放 TNF、IL-1 和趨化因子激活並延長中性粒細胞效應，而中性粒細胞釋放的蛋白酶、活性氧、損傷相關分子和脂質介質又可反過來加強巨噬細胞炎症反應。這一正反饋在控制微生物時有益，但如果威脅已經下降仍持續存在，就會轉化為黏膜損傷。

炎症從放大轉向消退的核心事件是中性粒細胞凋亡和巨噬細胞的凋亡細胞清除。通過 MerTK 等受體吞噬凋亡中性粒細胞後，巨噬細胞會抑制促炎轉錄，增加 IL-10、TGF-β、LXR/PPAR 相關代謝程序和脂氧素、消退素等促消退介質，同時增強基質重塑、血管和上皮修復信號。若中性粒細胞發生壞死而非凋亡、微生物持續穿越屏障或凋亡細胞清除受損，DAMP、顆粒酶和 TNF/IL-1 就會持續存在。因此，中性粒細胞和巨噬細胞的關係本質上是時間依賴的：早期共同放大防禦，後期由巨噬細胞將炎症主動轉化為修復。

Q08
問題
嗜酸性粒細胞來源的介質如何改變腸黏膜巨噬細胞的功能狀態和樹突狀細胞的活化？這些相互作用如何推動免疫耐受、組織修復與發炎之間的平衡發生變化？

回答
嗜酸性粒細胞可通過細胞因子、脂質介質、生長因子和顆粒產物改變腸黏膜巨噬細胞狀態。IL-4/IL-13 樣信號可促進吞噬、基質重塑和修復相關程序，TGF-β可參與修復但持續存在時也可能促纖維化；某些脂質介質具有促消退作用，而大量顆粒蛋白和活性氧則可作為危險信號，推動巨噬細胞進入炎症狀態。

樹突狀細胞同樣會受到嗜酸性粒細胞微環境影響。嗜酸性粒細胞來源的細胞因子和顆粒產物可改變樹突狀細胞成熟、共刺激分子和遷移，從而影響黏膜抗原呈遞更偏向耐受還是效應 T 細胞反應。低損傷環境中，完整上皮、調節性細胞因子和有效清除有利於耐受；在寄生蟲感染、食物過敏或其他 2 型炎症中，嗜酸性粒細胞數量和脫顆粒增加則會推動 Th2 樣反應及屏障破壞。因而，耐受、修復和炎症之間的平衡取決於嗜酸性粒細胞的激活強度和持續時間，而不是一個固定的“促炎”或“抗炎”表型。人類腸道中部分直接因果關係仍具有情境依賴性。

Q09
問題
在中性粒細胞性氣道發炎中，中性粒細胞與巨噬細胞如何透過細胞激素、脂質介質、中性粒細胞胞外誘捕網、凋亡細胞清除和代謝訊號相互影響？這種串擾如何決定發炎持續還是消退？

回答
中性粒細胞性氣道炎症中，巨噬細胞和上皮產生 TNF、IL-1、GM-CSF 及 CXCL8 等信號，促進中性粒細胞募集和存活。中性粒細胞隨後釋放彈性蛋白酶、活性氧、白三烯 B4、DAMP 和中性粒細胞胞外誘捕網，反過來刺激巨噬細胞。NET 中的 DNA、組蛋白和顆粒酶可激活模式識別及炎症小體通路，增加 IL-1β和新的中性粒細胞趨化信號，從而形成自我維持迴路。

代謝狀態會決定該迴路走向。高糖酵解、HIF-1α和琥珀酸相關的巨噬細胞狀態更有利於促炎細胞因子產生，而中性粒細胞本身高度依賴糖酵解，因此即使在缺氧黏液中也能持續發揮效應。炎症消退的關鍵是凋亡中性粒細胞被巨噬細胞有效清除；這會增加 IL-10、TGF-β和促消退脂質介質，並促使巨噬細胞轉向氧化代謝、脂質處理和組織修復。若中性粒細胞凋亡延遲、NET 形成過多、持續感染或巨噬細胞清除功能下降，炎症就更可能長期維持。

Q10
問題
在過敏性氣道發炎中，肥大細胞和嗜酸性粒細胞（在相關情境下包括嗜鹼性粒細胞）如何與樹突狀細胞和巨噬細胞相互作用，從而放大或限制第 2 型免疫反應？

回答
過敏性氣道炎症首先由上皮警報素和過敏原激活的樹突狀細胞推動 2 型免疫。樹突狀細胞促進 Th2 分化；IgE 致敏的肥大細胞再遇過敏原時快速釋放組胺、PGD2、半胱氨酰白三烯、TNF 和蛋白酶，造成支氣管收縮、血管通透性增加並促進嗜酸性粒細胞募集。IL-5 維持嗜酸性粒細胞生存，嗜酸性粒細胞釋放顆粒蛋白、白三烯、細胞因子和生長因子，可進一步損傷上皮並增強抗原呈遞細胞活化。

在相關情境下，嗜鹼性粒細胞可提供早期 IL-4 並加強 Th2 和 IgE 迴路。IL-4/IL-13 使巨噬細胞偏向替代激活狀態，急性期有助於清除和修復，但持續存在時可促進黏液、纖維化和重塑。限制 2 型免疫依賴調節性 T 細胞、IL-10、過敏原減少、IL-5 等存活信號下降、嗜酸性粒細胞凋亡和巨噬細胞清除，以及促消退脂質介質。不同哮喘內型中嗜鹼性粒細胞、肥大細胞和嗜酸性粒細胞的相對權重不同，因此這一網絡具有明顯的模塊化和情境依賴性。

Q11
問題
在急性細菌性尿路感染期間，募集到局部的中性粒細胞和單核細胞來源巨噬細胞如何在病原體清除、細胞激素產生、凋亡細胞清除和黏膜修復過程中協同或競爭？

回答
急性細菌性尿路感染中，中性粒細胞承擔最快、最強的直接抗菌效應。它們沿尿路上皮和巨噬細胞產生的趨化梯度進入感染黏膜及尿腔，通過吞噬、活性氧和顆粒抗菌物質迅速降低細菌數量。單核細胞來源巨噬細胞也能吞噬細菌，但更重要的作用包括持續產生細胞

因子、清除受損細胞和協調後續修復。兩者都可產生 TNF、IL-1 及趨化因子，因此在病原負荷高時既協同殺菌，也可能共同放大炎症。

所謂“競爭”更多表現為共享趨化位點、營養和吞噬靶物，而不是簡單互相抑制。感染控制後，中性粒細胞凋亡，巨噬細胞通過凋亡細胞清除將炎症程序切換為 IL-10、TGF-β和修復相關狀態，並促進尿路上皮再生。若中性粒細胞發生壞死或過度形成 NET、巨噬細胞清除失敗，或細菌在上皮細胞內形成持續儲庫，蛋白酶、活性氧和細胞因子會持續損傷黏膜。因此更符合證據的模式是：中性粒細胞早期佔主導，隨後由單核細胞/巨噬細胞承擔清除和修復，而不是兩類細胞長期同時處於最大激活狀態。

Q12

問題

在慢性膀胱黏膜發炎中，肥大細胞與巨噬細胞之間的串擾可能如何改變感覺神經訊號、血管通透性、上皮屏障功能以及發炎的持續或消退？

回答

慢性膀胱黏膜炎症中，肥大細胞和巨噬細胞可形成神經-血管-免疫正反饋。肥大細胞釋放組胺、胰蛋白酶、前列腺素、白三烯和神經生長因子，可直接或間接提高感覺神經興奮性；胰蛋白酶經 PAR2、組胺經相應受體還可促進神經肽釋放，導致尿急、疼痛和頻尿。同時，這些介質增加內皮通透性，使更多白細胞進入組織並形成水腫。

巨噬細胞受到肥大細胞介質和上皮 DAMP 刺激後產生 TNF、IL-1、IL-6、趨化因子和脂質介質，進一步激活血管和感覺神經，並削弱上皮連接。屏障變漏後，尿液中的溶質和微生物成分更容易刺激深層組織，形成持續性迴路。炎症消退需要巨噬細胞通過凋亡細胞清除轉向 IL-10、TGF-β和促消退脂質程序，同時修復尿路上皮連接並降低肥大細胞刺激。不同慢性膀胱疾病的驅動機制並不完全相同，因此感染模型中的發現不能直接等同於所有膀胱疼痛綜合徵。

Q13

問題

在潰瘍性結腸炎中，中性粒細胞募集、中性粒細胞胞外誘捕網形成、中性粒細胞代謝以及中性粒細胞-巨噬細胞串擾如何分別促進黏膜損傷或發炎消退？

回答

潰瘍性結腸炎活動期的典型特徵之一是大量中性粒細胞進入黏膜。上皮和髓系細胞產生 CXCL8 等趨化因子、補體信號及其他趨化物，推動中性粒細胞跨內皮和跨上皮遷移。中性粒細胞可殺滅穿越受損屏障的細菌，但其活性氧、髓過氧化物酶產物、彈性蛋白酶和其他顆粒酶也會直接損傷上皮。NET 中的 DNA、組蛋白和顆粒酶可捕獲微生物，卻也能損傷上皮並激活巨噬細胞，形成額外炎症放大。

炎癥結腸處於缺氧和代謝應激環境，中性粒細胞依賴糖酵解仍能持續效應，而琥珀酸、乳酸等代謝物也可改變髓系細胞狀態。持續的炎症巨噬細胞產生 TNF、IL-1 和趨化因子，繼續募集粒細胞；相反，當中性粒細胞凋亡並被有效清除後，巨噬細胞轉向 IL-10、TGF-β、促消退脂質和組織修復程序。因此，中性粒細胞本身並不等於致病：短暫募集有助於抗菌，持續募集、NET 過多、繼發壞死和清除失敗才更容易造成黏膜損傷。NET 在人類 UC 中的因果貢獻仍比“中性粒細胞募集”這一核心機制更不確定。

Q14

問題

在克隆氏症中，中性粒細胞、單核細胞來源巨噬細胞和樹突狀細胞之間失調的相互作用如何改變微生物處理、抗原呈遞、發炎持續以及上皮修復？

回答

克隆氏症中，微生物處理、屏障和髓系細胞關停機制的失調可把正常防禦轉化為慢性炎症。與 NOD2、ATG16L1 等相關的微生物感知和自噬異常、上皮屏障缺陷或持續菌體穿越，都可能使病原成分長期存在。中性粒細胞在不同階段可能出現早期募集不足或慢性期過度募集；單核細胞進入黏膜後形成炎症型巨噬細胞，產生 TNF、IL-1、IL-6 和 IL-23；樹突狀細胞則持續攝取抗原並推動 Th1/Th17 樣適應性反應。

長期存在的細菌成分使巨噬細胞和樹突狀細胞維持較強共刺激和 IL-12/IL-23 信號，而不是耐受性抗原呈遞。中性粒細胞蛋白酶、活性氧和組織 DAMP 又可反過來增強抗原呈遞細胞激活，形成持續迴路。修復同樣受影響：高水平 TNF、IL-1 和氧化應激會阻礙上皮恢復，而凋亡細胞清除不足則延遲巨噬細胞向生長因子和基質重塑程序轉變。不同患者的主導通路並不相同，因此克隆氏症更適合被理解為“微生物控制、炎症終止和組織修復”多個環節的匯合性失敗，而不是單一髓系細胞缺陷。

Q15

問題

在重度中性粒細胞性氣喘中，中性粒細胞、巨噬細胞和樹突狀細胞如何形成自我維持的發炎迴路？中性粒細胞胞外誘捕網、發炎小體訊號、微生物訊號以及糖皮質激素抗性可能分別發揮什麼作用？

回答

重度中性粒細胞性哮喘可由上皮損傷、巨噬細胞、樹突狀細胞和中性粒細胞共同形成自我維持迴路。微生物產物或損傷上皮激活 TLR 等先天通路，使巨噬細胞和上皮產生 IL-1、TNF、CXCL8 等趨化因子及生長因子，募集並延長中性粒細胞存活。樹突狀細胞可加強 Th17 或混合型適應性反應，IL-17 又促使上皮產生更多中性粒細胞趨化因子。中性粒細胞釋放的蛋白酶、活性氧和 DAMP 則繼續刺激巨噬細胞和上皮。

NET 可能進一步放大該迴路，因為其 DNA、組蛋白和顆粒酶可損傷上皮並激活模式識別及炎症小體。巨噬細胞 NLRP3 相關通路及 IL-1β在部分表型中具有機制學合理性，特別是在持續感染、菌群失衡或顆粒暴露存在時。糖皮質激素抵抗可能與 Th17 通路相對低敏感、氧化應激和 p38 MAPK 損傷糖皮質激素受體功能，以及持續微生物/炎症小體信號有關。NET 負荷高和巨噬細胞凋亡細胞清除不足也可

能維持類固醇不敏感的 DAMP 刺激。不過這些機制在人類患者中的權重並不一致，不能假定所有中性粒細胞性哮喘都由同一條迴路驅動。

Q16
問題
在嗜酸性粒細胞性氣喘中，嗜酸性粒細胞、肥大細胞以及在相關情境下的嗜鹼性粒細胞，如何與巨噬細胞、樹突狀細胞和氣道上皮相互作用，從而啟動、放大並最終促使第 2 型發炎消退？

回答
嗜酸性粒細胞性哮喘通常由受刺激的氣道上皮釋放 TSLP、IL-33 和 IL-25 開始。警報素激活樹突狀細胞和 ILC2；樹突狀細胞促進 Th2 分化，ILC2 和 Th2 產生 IL-5 與 IL-13。IL-5 維持嗜酸性粒細胞成熟和存活，嗜酸性粒細胞趨化因子促進其進入氣道。過敏原特異性 IgE 使肥大細胞致敏，再次暴露後肥大細胞迅速釋放組胺、PGD2、半胱氨酰白三烯和蛋白酶；在相關情境下，嗜鹼性粒細胞可補充早期 IL-4 信號。

放大階段中，嗜酸性粒細胞顆粒蛋白和活性氧損傷上皮，產生更多警報素；嗜酸性粒細胞和肥大細胞脂質介質加重支氣管收縮、血管滲漏和黏液。IL-4/IL-13 使巨噬細胞進入替代激活狀態，可幫助清除殘骸和修復，但長期存在時促進纖維化和黏液化生。炎症消退需要減少過敏原和警報素、降低 IL-5 等生存信號、嗜酸性粒細胞凋亡、巨噬細胞凋亡細胞清除、調節性 T 細胞和促消退脂質介質。也就是說，急性期有益的修復程序在 2 型信號長期持續時可轉為氣道重塑。

Q17
問題
在復發性尿路感染中，既往感染如何透過訓練性免疫、免疫耐受或組織重塑改變膀胱黏膜中的中性粒細胞和單核細胞/巨噬細胞反應？目前對這些機制各自的證據強度如何？

回答
既往尿路感染可能通過三類機制改變再次感染時的髓系反應。第一，訓練性免疫可在單核細胞或骨髓祖細胞中留下代謝和表觀遺傳改變，使後續刺激時的吞噬和炎症基因響應增強；這一機制在訓練性免疫領域總體較為確立，但“人類 UTI 後形成持久、膀胱特異的訓練狀態”證據仍有限。中性粒細胞壽命很短，因此長期記憶更可能通過祖細胞或組織環境間接傳遞，而不是由同一批中性粒細胞保存。

第二，反復強烈的模式識別刺激也可能造成耐受，使再次刺激時 TNF、IL-1 等反應下降；這可保護組織，卻可能延遲殺菌。第三，既往感染可重塑膀胱本身，包括上皮更新、基質和血管變化、神經重塑、趨化因子基線改變以及潛在的上皮內細菌儲庫。這些變化會直接影響下一次感染時中性粒細胞和單核細胞的募集。總體上，動物 UTI 模型對感染後組織重塑和持續上皮改變的證據較強；訓練性免疫或耐受的機制合理性較高，但在人類復發性 UTI 中的直接因果證據仍相對不足。

Q18
問題
在膀胱癌中，腫瘤相關中性粒細胞和腫瘤相關巨噬細胞如何在黏膜腫瘤微環境中相互作用，並影響抗原呈遞、T 細胞活性、血管新生、腫瘤侵襲以及免疫治療反應？

回答
膀胱癌黏膜腫瘤微環境中，腫瘤相關巨噬細胞和中性粒細胞可共同形成免疫抑制網絡。腫瘤及髓系細胞產生 CCL2、CSF1、CXCL8 等信號募集和維持這些細胞。腫瘤相關巨噬細胞可表達 IL-10、TGF-β、精氨酸酶和 PD-L1 等抑制程序，降低 T 細胞激活和有效抗原呈遞；腫瘤相關中性粒細胞可補充活性氧、精氨酸酶、蛋白酶及其他抑制因子，並通過趨化因子募集更多髓系細胞。

兩類細胞還可通過 VEGF、MMP 和基質重塑酶促進血管生成和侵襲，部分情境下 NET 也可為腫瘤細胞黏附和侵襲提供支架。另一方面，巨噬細胞和中性粒細胞具有高度可塑性，干擾素反應或強吞噬表型也可支持抗腫瘤免疫，因此“細胞數量多”並不等同於“功能一定促腫瘤”。強免疫抑制性髓系環境可限制 T 細胞進入或削弱檢查點抑制治療，而激活先天感知或重編程巨噬細胞可能改善抗原呈遞。膀胱癌的特殊之處在於局部膀胱灌注可直接改變黏膜免疫，因此治療必須同時考慮抗腫瘤效應與正常尿路抗菌和修復功能。

Q19
問題
在潰瘍性結腸炎中，哪些具有機制學合理性的策略可能抑制致病性中性粒細胞募集或中性粒細胞胞外誘捕網相關損傷，同時盡量保留抗微生物防禦和巨噬細胞介導的修復？請說明預期獲益、潛在權衡及證據缺口。

回答
在 UC 中，更合理的目標不是清除中性粒細胞，而是降低持續性過度募集或最具毒性的效應。部分或局部抑制 CXCR2 依賴的趨化可減少中性粒細胞在黏膜中的長期積聚，但系統性強力阻斷可能削弱細菌防禦。對 NET 損傷可考慮降解胞外 DNA、抑制 PAD4、彈性蛋白酶或髓過氧化物酶，從而減少 NET 形成或組織毒性；然而 NET 也有捕獲微生物的作用，因此過度抑制可能增加感染或細菌擴散風險。

另一條思路是增強巨噬細胞凋亡細胞清除和促消退介質，使中性粒細胞在完成殺菌後更快退出，同時保留巨噬細胞修復。局部結腸遞送、在炎症過度階段短時給藥，或僅選擇 NET 高/CXCR2 高患者，理論上更有利於控制風險。關鍵證據缺口包括：人類 UC 中哪些中性粒細胞效應是真正因果驅動；NET 抑制是否能改善黏膜而不增加感染；長期趨化阻斷是否會妨礙傷口封閉和上皮修復。因而，相比廣泛髓系細胞耗竭，更合理的是重新平衡“募集-死亡-清除-修復”這一時間序列。

Q20
問題
如何利用腸道巨噬細胞和粒細胞的代謝或表觀遺傳重編程來恢復發炎性腸病中的黏膜穩態？哪些機制可能導致療效喪失或非預期的免疫抑制？

回答
代謝或表觀遺傳重編程的目標應是把髓系細胞從持續炎症狀態拉回能夠清除和修復的狀態，而不是全面關閉先天免疫。巨噬細胞中，高糖酵解、HIF-1α、琥珀酸積累及炎症增強子開放可支持 IL-1、TNF 產生；提高線粒體功能、促進氧化代謝、增強 itaconate-NRF2 樣抗氧化通路以及 PPAR/LXR 相關脂質處理，可能有利於凋亡細胞清除和修復。通過調節組蛋白乙酰化、甲基化或 BET 依賴的增強子，也可能降低長期炎症基因表達。

粒細胞尤其是成熟中性粒細胞壽命短，直接長期重編程較困難，但其效應仍受糖酵解、缺氧、NADPH 氧化酶底物和 NET 相關染色質狀態影響；若直接重塑骨髓祖細胞，則系統性風險更高。療效可能因持續微生物洩漏、缺氧或細胞因子暴露而喪失，因為這些刺激會重新強迫細胞進入炎症程序。非預期免疫抑制則可能來自過度抑制糖酵解、表觀遺傳轉錄或先天感知，導致細菌殺滅、抗原呈遞和傷口防禦下降。因此，更理想的是局部、可逆、細胞狀態選擇性的重編程，而不是全身性“靜音”髓系免疫。

Q21
問題
能夠改變第 2 型細胞激素訊號的生物製劑，除降低嗜酸性粒細胞數量外，還可能如何重塑氣道黏膜中的嗜酸性粒細胞-肥大細胞-巨噬細胞-樹突狀細胞網路？

回答
改變 2 型細胞因子信號的生物制劑可以在不只“減少嗜酸性粒細胞數量”的層面重塑氣道網絡。阻斷 IL-5 或其受體會降低嗜酸性粒細胞成熟、存活和組織停留，從而減少其顆粒蛋白、白三烯和細胞因子對上皮、巨噬細胞和樹突狀細胞的持續刺激。阻斷 IL-4/IL-13 信號則可降低嗜酸性粒細胞趨化因子、黏液和 IgE 相關程序，並減少巨噬細胞長期替代激活，因此會同時改變上皮和巨噬細胞表型。

更上游的 TSLP，乃至 IL-33 通路阻斷，可減少樹突狀細胞預激活和 ILC2 啓動，從網絡起點降低 Th2 記憶刺激、肥大細胞反應和巨噬細胞 2 型極化。這樣即使血液嗜酸性粒細胞計數變化不大，氣道局部的黏液、屏障修復、過敏原閾值和細胞間反饋也可能顯著改變。不過不同靶點的網絡效應並不相同，長期存活的肥大細胞、既有氣道重塑以及非 2 型炎症可在治療後繼續存在。因此，嗜酸性粒細胞計數只能反映網絡的一部分，而不能完整代表黏膜免疫已經如何被重塑。

Q22
問題
在中性粒細胞性氣喘中，哪些針對髓系細胞的策略可能中斷中性粒細胞-巨噬細胞發炎迴路，同時保留抗細菌防禦？這些策略所依據的機制中，哪些已有臨床證據，哪些仍主要處於臨床前階段？

回答
中性粒細胞性哮喘的治療難點在於中性粒細胞同時承擔重要抗細菌防禦，因此單純耗竭並不理想。更合理的策略包括：降低過度 CXCR2 依賴募集，減弱巨噬細胞 IL-1/TNF 驅動的趨化因子迴路，促進巨噬細胞凋亡細胞清除，或選擇性限制 NET、彈性蛋白酶和過量氧化產物。促消退脂質介質和代謝重編程理論上也可縮短炎症持續時間，而不完全阻斷吞噬和殺菌。

臨床上，目前並沒有一種廣泛確立、專門針對“中性粒細胞-巨噬細胞迴路”的標準治療。大環內酯在部分重症哮喘人群中有降低急性加重的臨床證據，但其機制多效，包括抗菌和免疫調節，並非純髓系靶向。CXCR2 拮抗劑已進入臨床研究並能降低氣道中性粒細胞，但療效和感染/安全權衡限制了常規應用。更直接的炎症小體抑制、PAD4/NET 抑制、巨噬細胞代謝重編程、促消退介質及細胞特異遞送仍主要處於臨床前或早期轉化階段。未來最關鍵的是內型分層：感染驅動型與無菌 NET/炎症小體驅動型患者不應採用相同策略。

Q23
問題
訓練性免疫或黏膜疫苗策略是否可能透過有益地重編程單核細胞/巨噬細胞和中性粒細胞來減少復發性尿路感染？請解釋其擬議機制、潛在的組織特異性風險以及目前證據的侷限。

回答
訓練性免疫和黏膜疫苗都有可能通過提前改善局部防禦來降低復發性 UTI。訓練性免疫的目標是使單核細胞祖細胞或組織巨噬細胞發生代謝和表觀遺傳改變，使再次遇到細菌時更快吞噬並產生適度的炎症反應。由於成熟中性粒細胞壽命短，對其長期影響更可能是間接的，例如改變骨髓輸出、趨化時序或巨噬細胞對中性粒細胞的指導。

黏膜疫苗則可同時建立病原特異性抗體和 T 細胞記憶，並改變上皮與髓系細胞的“預備狀態”。理想情況下，局部 IgA/IgG 和駐留淋巴細胞降低細菌黏附，使中性粒細胞面對更小的菌量，從而減少組織損傷性募集。潛在風險包括訓練過強導致無菌炎症、疼痛、通透性增加或纖維化；反復先天刺激也可能反而誘導耐受，削弱殺菌。現階段證據更支持“概念可行”而非已經建立統一機制治療。仍需要人體研究證明保護作用持久，確認膀胱軸確實發生了巨噬細胞或祖細胞重編程，並證明復發減少的同時不會增加慢性炎症症狀。

Q24

問題

如何透過選擇性重編程腫瘤相關巨噬細胞和中性粒細胞來增強膀胱癌的抗腫瘤免疫，同時盡量減少對保護性尿路黏膜免疫的破壞？請比較不同機制策略及其主要轉譯障礙。

回答

選擇性重編程腫瘤相關巨噬細胞和中性粒細胞的目標，是把免疫抑制性細胞轉為更強吞噬、抗原呈遞和 T 細胞支持狀態，而不是把所有髓系細胞清除。巨噬細胞方面，可考慮阻斷 CSF1/CSF1R 或 CCL2 募集、抑制 PI3Kγ樣免疫抑制信號、解除 CD47-SIRPα“別吃我”軸，或用 TLR/STING 樣先天激動方式增強抗原呈遞。相比完全耗竭，重編程更有機會保留正常尿路的抗菌和修復功能。

中性粒細胞方面，可通過 CXCR2 限制腫瘤內募集，或抑制 NET、彈性蛋白酶及 TGF-β相關抑制表型，減少侵襲和 T 細胞抑制。但系統性干預會威脅尿路對細菌的快速中性粒細胞防禦，因此膀胱灌注、腫瘤靶向納米遞送等局部策略尤其有吸引力。主要轉化障礙包括 TAM/TAN 高度異質和可塑、缺乏穩定的人體標誌物、替代趨化通路造成補償性募集，以及治療導致屏障損傷後增加感染風險。與檢查點抑制或局部免疫治療聯用可能協同，但順序必須避免過度削弱髓系抗原呈遞和初始炎症。真正可行的方案需要空間生物標誌物和局部遞送來區分“腫瘤相關髓系程序”與“正常尿路保護性免疫”。

II. Model B

English (EN)

Model B | English | 24 Questions

Model B

Myeloid–mucosal immunity | Q01–Q24

Q01

Question

At the healthy intestinal mucosa, how do neutrophils contribute to barrier defense while interacting with resident macrophages and dendritic cells, and what mechanisms normally limit neutrophil-driven tissue injury?

Response

In a healthy intestine, neutrophils are present at low numbers in the lamina propria and are recruited rapidly when microbes cross the epithelial barrier. Epithelial cells, resident macrophages, and stromal cells provide chemotactic signals such as CXCL1/CXCL2/CXCL8 and lipid mediators. Recruited neutrophils phagocytose bacteria, generate reactive oxygen species, release antimicrobial granule proteins, and can form extracellular traps when the microbial burden is high. Resident macrophages help by sensing breaches, producing early recruitment signals, and later clearing spent neutrophils. Dendritic cells mainly coordinate antigen sampling and adaptive responses rather than acting as the first bactericidal cell.

The important feature of the healthy mucosa is that this response is spatially and temporally restricted. Intact mucus, epithelial tight junctions, antimicrobial peptides, and rapid epithelial repair reduce the need for prolonged neutrophil activation. Intestinal macrophages are relatively hyporesponsive to harmless luminal stimuli and produce IL-10 and other regulatory signals. Neutrophils undergo apoptosis after completing their task, and macrophage efferocytosis switches the local environment toward IL-10, TGF-beta, pro-resolving lipid mediators, and tissue repair.

Tissue injury is further limited by antiproteases, antioxidant systems, DNases that clear extracellular DNA, and termination of chemokine gradients. When these brakes fail, the same neutrophil tools that kill microbes—proteases, oxidants, and NET-associated histones—can damage crypt epithelium and perpetuate inflammation.

Q02

Question

At the healthy intestinal mucosa, how do eosinophils contribute to homeostasis and communicate with macrophages or dendritic cells, and how do these roles differ from eosinophil functions during inflammation?

Response

Eosinophils are a normal component of the intestinal lamina propria, especially in the small bowel. At baseline they are maintained largely through IL-5 and eotaxin/CCR3 signaling and appear to participate in barrier surveillance, immunoglobulin production, and tissue remodeling. They can release cytokines, growth factors, and lipid mediators without undergoing the explosive degranulation seen in disease. Their communication with macrophages is often biased toward a repair-oriented environment; eosinophil-derived IL-4 and IL-13 can favor alternative macrophage programs, although the strength of this pathway varies by species and tissue. Interactions with dendritic cells may alter antigen presentation and local tolerance, but these mechanisms are less firmly established in humans.

During inflammation, the balance changes. Eosinophils are recruited in larger numbers, survive longer, and release major basic protein, eosinophil peroxidase, cationic proteins, leukotrienes, and inflammatory cytokines. These products can injure epithelium, increase vascular permeability, activate sensory nerves, and modify macrophage or dendritic-cell activation. At the same time, eosinophils can still support repair by producing TGF-beta and growth factors.

Clinically, eosinophils should therefore not be viewed as purely pathogenic. Homeostatic eosinophils may support mucosal integrity, whereas excessive or dysregulated activation can shift the same network toward inflammation and fibrosis. Much of the detailed cell-to-cell circuitry is well supported in animal models but remains context-dependent in human intestine.

Q03

Question

At the healthy respiratory mucosa and during an acute microbial challenge, how do neutrophils coordinate with alveolar or interstitial macrophages and dendritic cells to provide host defense while limiting collateral epithelial injury?

Response

In the healthy lung, alveolar macrophages keep neutrophil recruitment low by clearing inhaled particles and suppressing unnecessary inflammation. During an acute bacterial or fungal challenge, alveolar and interstitial macrophages sense microbial products through pattern-recognition receptors and release TNF, IL-1, CXCL1/CXCL2/CXCL8, and other mediators. These signals recruit neutrophils from the circulation. Dendritic cells sample antigen and help shape later T-cell responses, while also contributing cytokines that can reinforce local antimicrobial activity.

Neutrophils provide rapid killing through phagocytosis, oxidative burst, antimicrobial proteases, and, in selected settings, extracellular traps. Macrophages and neutrophils can amplify each other early: macrophage chemokines recruit neutrophils, and neutrophil-derived danger signals can further activate macrophages. This is useful when microbial burden is high but becomes harmful if prolonged.

Resolution depends on active braking mechanisms. Neutrophils undergo apoptosis and are removed by macrophage efferocytosis, which promotes IL-10, TGF-beta, lipid mediators such as lipoxins/resolvins, and epithelial repair. Airway antioxidants, antiproteases, mucus clearance, and DNases limit oxidant, elastase, and NET-associated injury. The clinical balance is therefore not simply "more neutrophils equals better defense": timely recruitment improves pathogen control, whereas delayed clearance or persistent activation increases epithelial damage and can contribute to acute lung injury or chronic airway inflammation.

Q04

Question

How do eosinophils interact with macrophages and dendritic cells in the airway mucosa under homeostatic conditions and during type 2 inflammation?

Response

Under homeostatic conditions, airway eosinophils are relatively sparse. Alveolar macrophages and airway dendritic cells generally maintain a low-inflammatory environment, and epithelial cells limit exposure to inhaled antigens. Eosinophils can provide low-level cytokines and growth factors, but their contribution to normal airway homeostasis is less prominent than in the gastrointestinal tract.

During type 2 inflammation, epithelial alarmins such as IL-33, TSLP, and IL-25 activate dendritic cells and type 2 innate lymphoid pathways. Dendritic cells promote Th2 differentiation, leading to IL-4, IL-5, and IL-13 production. IL-5 supports eosinophil survival, while eotaxins recruit eosinophils through CCR3. Activated eosinophils then release leukotrienes, granule proteins, cytokines, and growth factors. These products can enhance dendritic-cell activation and can push macrophages toward IL-4/IL-13-responsive repair programs.

This network has both protective and pathogenic aspects. Macrophage repair programs can clear debris and support epithelial restoration, but persistent type 2 signaling can also promote mucus production, smooth-muscle reactivity, and remodeling. Eosinophil granule proteins can directly injure epithelium. Thus, the same macrophage and dendritic-cell interactions that help terminate an acute response can, when repeatedly stimulated, contribute to chronic asthma. The exact importance of direct eosinophil-to-dendritic-cell signaling in human airways remains less certain than the broader cytokine network.

Q05

Question

In the urinary tract mucosa, how do neutrophils coordinate with monocytes or macrophages and dendritic cells during early antibacterial defense, and what mechanisms prevent excessive tissue injury?

Response

During an early bacterial urinary tract infection, urothelial cells and resident myeloid cells detect bacteria through pattern-recognition receptors, including TLR pathways. They rapidly produce IL-6, CXCL8 and related chemokines, leading to a strong neutrophil influx. Neutrophils migrate across the urothelium into the urinary space, where they phagocytose bacteria, generate reactive oxygen species, release antimicrobial proteins, and sometimes form extracellular traps. This luminal migration is important because it places the effector cells directly where the bacterial burden is highest.

Monocytes and macrophages support this response by producing recruitment signals, engulfing bacteria and debris, and later clearing apoptotic neutrophils. Dendritic cells contribute antigen presentation and help connect the innate response to adaptive immunity. There can be overlap and competition: excessive macrophage or neutrophil cytokine production can intensify inflammation without improving bacterial clearance.

Protection from tissue injury relies on rapid bacterial control followed by rapid shutdown. Neutrophil apoptosis and macrophage efferocytosis are central. IL-10, TGF-beta, pro-resolving lipid mediators, epithelial regeneration, and restoration of tight junctions help re-establish the barrier. If neutrophils persist, proteases, oxidants, and NET components can injure urothelium and worsen symptoms. Thus, the desirable response is a short, high-intensity antimicrobial burst followed by efficient clearance and repair rather than prolonged neutrophilic inflammation.

Q06

Question

In the bladder or lower urogenital mucosa, how can mast-cell mediators influence macrophage and dendritic-cell functions, and how might this network contribute to both host defense and inflammatory symptoms?

Response

Mast cells sit close to blood vessels, nerves, and epithelial surfaces in the bladder and lower urogenital tract, so their mediators can rapidly influence macrophages and dendritic cells. Histamine, TNF, tryptase, prostaglandins, leukotrienes, IL-6, and other mediators can increase vascular permeability and promote recruitment or activation of myeloid cells. Histamine receptors and protease-activated receptors on macrophages and dendritic cells can alter cytokine production, antigen presentation, and tissue-remodeling programs.

This is useful during infection or tissue injury because mast cells can accelerate local blood flow, leukocyte entry, and antimicrobial inflammation. Macrophages then provide phagocytosis, cytokines, efferocytosis, and repair, while dendritic cells help initiate adaptive immunity. The same network can also produce symptoms. Histamine, tryptase, prostaglandins, ATP-related signaling, and nerve growth factors can sensitize pelvic

sensory nerves, contributing to urgency, frequency, pain, or burning. Increased vascular permeability can cause edema and further amplify inflammatory signaling.

If the stimulus persists, mast-cell and macrophage cytokines can form a feed-forward loop that weakens epithelial barrier function and maintains neuroinflammation. Conversely, regulatory macrophage programs, IL-10, TGF-beta, and resolution mediators can dampen mast-cell-driven inflammation. Direct evidence for each pathway varies by condition, but this neuro-immune-vascular interaction is a plausible framework for why the same mast-cell response can contribute to both host defense and chronic inflammatory symptoms.

Q07

Question

During acute intestinal mucosal inflammation and subsequent resolution, how do neutrophils and macrophages influence each other through recruitment signals, cytokines, efferocytosis, lipid mediators, and tissue-repair programs?

Response

In acute intestinal inflammation, macrophages and epithelial cells initiate neutrophil recruitment through CXCL chemokines, IL-1, TNF, complement fragments, and lipid mediators. Neutrophils then kill microbes but also release ROS, proteases, inflammatory cytokines, and damage-associated signals that can further activate macrophages. Monocytes are recruited through signals such as CCL2 and differentiate into inflammatory macrophages, creating a positive feedback loop when the insult is not controlled.

Resolution is an active phase rather than a passive decline. Neutrophils undergo apoptosis, and macrophage efferocytosis is a major turning point. Engulfment of apoptotic neutrophils suppresses inflammatory cytokine production and promotes IL-10, TGF-beta, and repair-associated programs. There is also a lipid-mediator “class switch”: early leukotriene and prostaglandin signals are progressively replaced by lipoxins, resolvins, protectins, and maresins that reduce further neutrophil recruitment and improve clearance.

Repair-oriented macrophages stimulate epithelial restitution, angiogenesis, extracellular-matrix remodeling, and growth-factor production. However, these states are not a simple M1/M2 binary; intestinal macrophages occupy a continuum that depends on microbial load, hypoxia, and cytokines. Failure of efferocytosis or persistent bacterial products keeps macrophages inflammatory and can convert a normally self-limited neutrophil response into chronic mucosal damage. Clinically, successful resolution requires both effective pathogen control and timely transition from recruitment to clearance and repair.

Q08

Question

How can eosinophil-derived mediators alter macrophage polarization and dendritic-cell activation in the intestinal mucosa, and how could these interactions shift the balance among tolerance, tissue repair, and inflammation?

Response

Eosinophils can influence intestinal macrophages and dendritic cells through cytokines, lipid mediators, granule proteins, and growth factors. IL-4 and IL-13 can favor macrophage programs associated with wound repair, extracellular-matrix handling, and reduced classical inflammatory signaling. Eosinophils may also produce TGF-beta, IL-10-related signals, and lipid mediators that support restoration of tissue integrity. In some settings, however, eosinophil peroxidase, cationic granule proteins, leukotrienes, and danger signals promote macrophage activation and tissue injury.

Dendritic cells can be affected in both directions. A relatively quiet eosinophil environment may support tolerogenic antigen presentation and maintenance of responses to food or commensal antigens. During active inflammation, eosinophil-derived mediators can increase dendritic-cell maturation, migration, or cytokine production and thereby promote T-cell activation. Direct eosinophil antigen presentation has been described experimentally but is unlikely to be the dominant pathway in human intestine.

The net effect depends on timing and magnitude. Short-lived eosinophil activation may help contain injury and support repair, whereas persistent recruitment and degranulation can shift macrophages and dendritic cells toward an inflammatory circuit. The strongest mechanistic data come from animal and ex-vivo studies, so it is safer to view eosinophils as context-dependent modulators rather than assigning them a fixed pro- or anti-inflammatory role.

Q09

Question

During neutrophilic airway inflammation, how do neutrophils and macrophages influence each other through cytokines, lipid mediators, neutrophil extracellular traps, efferocytosis, and metabolic signals, and how can this crosstalk determine persistence versus resolution?

Response

In neutrophilic airway inflammation, macrophages release TNF, IL-1, CXCL8, GM-CSF, and lipid mediators that recruit and prolong the survival of neutrophils. Neutrophils in turn release proteases, ROS, alarmins, leukotriene B4, and extracellular traps that can further activate macrophages. NET-associated DNA and histones are antimicrobial, but they can damage epithelium and stimulate inflammasome-related or other innate signaling when they persist.

Efferocytosis is a key checkpoint. When macrophages efficiently ingest apoptotic neutrophils through receptors such as MerTK-related pathways, they reduce inflammatory cytokine output and increase IL-10, TGF-beta, lipid-resolving programs, and tissue repair. Defective efferocytosis leaves secondary necrotic neutrophils and extracellular proteases in the airway, which reinforces inflammation.

Metabolism also shapes this cross-talk. Hypoxia, lactate, succinate, mitochondrial stress, and HIF-1-linked glycolytic programs can maintain inflammatory macrophage and neutrophil functions. In contrast, recovery of mitochondrial metabolism and lipid-handling pathways supports resolution in macrophages. Persistence therefore occurs when recruitment, NET formation, metabolic stress, and poor clearance reinforce each other. Resolution occurs when microbial burden falls, chemokine production declines, neutrophils die in a controlled manner, NETs are dismantled, and macrophages switch from inflammatory signaling to efferocytosis and repair.

Q10

Question

During allergic airway inflammation, how do mast cells and eosinophils, with basophils where relevant, interact with dendritic cells and macrophages to amplify or restrain type 2 immunity?

Response

In allergic airway inflammation, mast cells, eosinophils, and sometimes basophils amplify type 2 immunity by interacting with dendritic cells, macrophages, and epithelium. Allergen-specific IgE cross-linking of Fc-epsilon-RI on mast cells triggers histamine, tryptase, prostaglandin D2, leukotrienes, TNF, and type 2 cytokines. These mediators increase vascular permeability, bronchoconstriction, and recruitment of eosinophils and dendritic cells. Basophils can provide an early source of IL-4 in selected settings, although their relative importance varies.

Dendritic cells take up allergen and drive Th2 responses, which provide IL-4, IL-5, and IL-13. IL-5 sustains eosinophils, while IL-4/IL-13 influence macrophages, mucus production, and tissue remodeling. Eosinophils release granule proteins and lipid mediators that can injure epithelium and further activate antigen-presenting cells. Macrophages can either amplify the response through inflammatory cytokines or adopt repair-oriented programs that clear debris and help restore the epithelial barrier.

Restraint comes from regulatory cytokines such as IL-10 and TGF-beta, inhibitory receptor signaling, efferocytosis, and pro-resolving lipids. Clinically, chronic disease reflects repeated failure to terminate this network: persistent mast-cell activation, eosinophil survival, ongoing antigen presentation, and remodeling can maintain symptoms even after the initial allergen exposure has ended.

Q11

Question

During acute bacterial urinary tract infection, how do recruited neutrophils and monocyte-derived macrophages cooperate or compete in pathogen clearance, cytokine production, efferocytosis, and mucosal repair?

Response

In acute bacterial urinary tract infection, neutrophils are the fastest high-capacity effector cells. They are recruited by urothelial and myeloid chemokines, cross the bladder epithelium, and kill bacteria by phagocytosis, oxidants, granule enzymes, and extracellular trapping. Monocyte-derived macrophages arrive somewhat later and have a broader role: they ingest bacteria and damaged cells, produce cytokines, clear apoptotic neutrophils, and coordinate mucosal repair.

The two populations cooperate when macrophage-derived chemokines recruit neutrophils and when macrophage efferocytosis removes neutrophils after bacterial control. They can also compete. Both consume microbes and inflammatory signals, and excessive TNF/IL-1 production from macrophages can intensify neutrophil recruitment. Conversely, heavy neutrophil degranulation can damage urothelium and release DAMPs that keep macrophages activated. If bacterial clearance is incomplete, this feedback can prolong tissue injury without improving sterilization.

The transition to repair depends on falling bacterial burden, neutrophil apoptosis, efficient efferocytosis, and reduced chemokine production. Macrophages then shift toward IL-10, TGF-beta, growth factors, matrix remodeling, and epithelial restitution. Therefore, optimal defense is sequential rather than competitive: rapid neutrophil killing first, followed by macrophage-dominated clearance and repair. The exact timing varies with pathogen strain, host sex, prior infection, and the depth of bacterial invasion.

Q12

Question

In chronic bladder mucosal inflammation, how might mast-cell–macrophage crosstalk alter sensory signaling, vascular permeability, epithelial barrier function, and the persistence or resolution of inflammation?

Response

In chronic bladder inflammation, mast-cell and macrophage cross-talk can connect immune activation to pain, vascular leakage, and barrier dysfunction. Mast-cell histamine, tryptase, prostaglandins, leukotrienes, TNF, and nerve growth factors can activate or sensitize sensory nerves. Tryptase acting through protease-activated receptors and histamine signaling can lower the threshold for pain and urgency. Macrophage-derived IL-1, TNF, IL-6, prostaglandins, and reactive mediators can reinforce the same neural pathways.

These mediators also affect the vasculature. Histamine and inflammatory cytokines increase endothelial permeability, allowing plasma proteins and leukocytes to enter the tissue. At the urothelium, cytokines, proteases, and oxidants can weaken tight-junction proteins and the surface barrier, increasing exposure of subepithelial nerves and immune cells to urinary solutes. A damaged epithelium then releases ATP and danger signals that reactivate mast cells and macrophages.

Whether inflammation persists or resolves depends on the macrophage response. Inflammatory macrophage programs can maintain TNF/IL-1 signaling, whereas efferocytosis and IL-10/TGF-beta-dominant programs support epithelial repair and reduce mast-cell activation. Persistent repair signaling can also become maladaptive and contribute to fibrosis. Human evidence is heterogeneous across chronic cystitis syndromes, so these pathways are best viewed as interacting mechanisms rather than a single proven cause of symptoms.

Q13

Question

In ulcerative colitis, how do neutrophil recruitment, neutrophil extracellular trap formation, neutrophil metabolism, and neutrophil–macrophage crosstalk contribute to mucosal injury versus inflammatory resolution?

Response

In ulcerative colitis, neutrophil recruitment is a defining feature of active mucosal inflammation. Epithelial and myeloid production of CXCL8-related chemokines, complement signals, and adhesion molecules drives neutrophils into crypts, where crypt abscesses can form. Neutrophils are useful because they contain bacteria and limit translocation, but their ROS, elastase, myeloperoxidase products, and other proteases can directly injure epithelium when recruitment is intense or prolonged.

NET formation adds another layer. NET DNA and antimicrobial proteins can trap microbes, yet histones, proteases, and persistent extracellular DNA can damage epithelial cells and stimulate innate immune pathways. Activated neutrophils rely heavily on glycolysis and can function in hypoxic inflamed mucosa; prolonged metabolic stress may favor survival and inflammatory activity rather than orderly apoptosis.

Macrophage cross-talk helps determine outcome. Early macrophage TNF, IL-1, and chemokines sustain neutrophil influx. Later, macrophage efferocytosis of apoptotic neutrophils promotes IL-10, TGF-beta, pro-resolving lipids, and epithelial repair. If efferocytosis is inefficient or microbial products remain, the circuit stays inflammatory. Thus, neutrophils are not simply harmful in ulcerative colitis: too little recruitment can impair bacterial containment, whereas uncontrolled recruitment, NET persistence, and defective clearance contribute to mucosal ulceration. This is why selective control of recruitment or NET-associated injury is more attractive than broad neutrophil depletion.

Q14

Question

In Crohn's disease, how can dysregulated interactions among neutrophils, monocyte-derived macrophages, and dendritic cells alter microbial handling, antigen presentation, inflammatory persistence, and epithelial repair?

Response

Crohn's disease combines defective microbial handling with excessive and persistent immune activation. Neutrophils are recruited to sites where the epithelial barrier is breached, but their function can be inconsistent: some patients show delayed or ineffective early innate responses, while established lesions contain highly activated neutrophils that release ROS and proteases. Genetic pathways involving NOD2, autophagy, and epithelial defense can alter how bacteria are sensed and cleared.

Recruited monocytes can differentiate into inflammatory macrophages that produce TNF, IL-1, IL-6, and IL-23. If bacterial material persists, these macrophages remain activated rather than transitioning to efferocytosis and repair. Dendritic cells sample luminal and tissue antigens and can reinforce Th1/Th17 responses through IL-12/IL-23-related pathways. This creates a loop in which microbial products, macrophage cytokines, T-cell responses, and neutrophil recruitment maintain one another.

Repair is also affected. Excess TNF, proteases, ROS, and matrix-remodeling enzymes delay epithelial restitution, while chronic macrophage and stromal activation can promote fibrosis. At the same time, macrophage IL-10, TGF-beta, growth factors, and neutrophil clearance are needed for healing. Therefore, the problem is not one "bad" cell type but loss of coordination: inadequate early containment can coexist with excessive later inflammation. The relative contribution of each pathway varies among patients, which helps explain the clinical heterogeneity of Crohn's disease.

Q15

Question

In severe neutrophilic asthma, how do neutrophils, macrophages, and dendritic cells form self-sustaining inflammatory circuits, and what roles may neutrophil extracellular traps, inflammasome signaling, microbial signals, and corticosteroid resistance play?

Response

Severe neutrophilic asthma can become self-sustaining when epithelial cells, neutrophils, macrophages, dendritic cells, and Th17-like responses reinforce each other. Microbial products, epithelial injury, and IL-1/TNF signaling drive CXCL8 and G-CSF, recruiting and prolonging neutrophil survival. Macrophages can add IL-1 beta and other inflammasome-related cytokines, while dendritic-cell activation can promote IL-17-producing T cells. IL-17 then stimulates more epithelial and stromal neutrophil chemokines.

NETs may strengthen this loop. Extracellular DNA, histones, neutrophil elastase, and myeloperoxidase products can injure airway epithelium and activate innate sensors. NLRP3 inflammasome signaling is biologically plausible and has supportive experimental data, but its importance differs among patients. Chronic bacterial colonization or airway dysbiosis can provide repeated TLR stimulation and maintain the circuit.

Corticosteroid resistance is multifactorial. IL-17-dominant inflammation, oxidative stress, altered p38/MAPK signaling, reduced steroid receptor function, and low HDAC2 activity have all been implicated. Corticosteroids can also delay neutrophil apoptosis, which may partly explain why eosinophilic inflammation often responds better than neutrophilic disease. Clinically, the best-supported concept is a heterogeneous, non-type-2 asthma phenotype with persistent innate inflammation; NET and inflammasome-targeted approaches remain more translational than established standard therapy.

Q16

Question

In eosinophilic asthma, how do eosinophils, mast cells, and where relevant basophils interact with macrophages, dendritic cells, and airway epithelium to initiate, amplify, and resolve type 2 inflammation?

Response

Eosinophilic asthma usually begins with epithelial sensing of allergens, viruses, or pollutants. Airway epithelial cells release TSLP, IL-33, and IL-25, which activate dendritic cells and type 2 innate pathways. Dendritic cells drive Th2 responses, while ILC2 cells provide additional IL-5 and IL-13. IL-5 promotes eosinophil production and survival; eotaxins recruit eosinophils into the airway. Mast cells are activated through allergen-specific IgE and release histamine, leukotrienes, prostaglandin D2, and cytokines. Basophils can provide IL-4 in some settings.

The amplification phase is multicellular. Eosinophil granule proteins, lipid mediators, and cytokines injure epithelium and enhance bronchial hyperresponsiveness. IL-4/IL-13 act on macrophages and epithelium, promoting mucus production, chemokines, and repair/remodeling programs. Macrophages clear debris but may also contribute growth factors and fibrosis when type 2 signaling is chronic. Dendritic cells maintain antigen presentation and memory Th2 responses.

Resolution requires removal of the trigger, eosinophil apoptosis or egress, macrophage efferocytosis, restoration of epithelial barrier function, and a shift toward regulatory cytokines and pro-resolving lipids. In practice, corticosteroids and type 2 biologics work by interrupting different parts of this network. Persistent epithelial alarmin production, mast-cell activation, or structural remodeling can allow symptoms to continue even when eosinophil numbers fall.

Q17

Question

In recurrent urinary tract infection, how can prior infection reshape neutrophil and monocyte or macrophage responses at the bladder mucosa through trained immunity, tolerance, or tissue remodeling, and how strong is the evidence for each mechanism?

Response

Prior urinary infection can plausibly reshape later neutrophil and monocyte/macrophage responses through three mechanisms, but the evidence is uneven. Trained immunity refers to epigenetic and metabolic changes that make innate cells or their progenitors respond more strongly on re-

exposure. This is well established as a general immunologic principle, and mouse infection models support lasting innate reprogramming, but direct proof that trained bladder myeloid cells are a dominant cause of recurrent UTI in humans is still limited.

Tolerance is the opposite pattern: repeated TLR or inflammatory stimulation can reduce later TNF/IL-1 production and blunt chemotaxis or bactericidal responses. Endotoxin tolerance is mechanistically well established, but whether it explains clinically important recurrent UTI in otherwise healthy patients is uncertain. It may coexist with trained features in different cell subsets.

Tissue remodeling has stronger direct relevance to the bladder. Prior infection can alter urothelial differentiation, epithelial barrier properties, extracellular matrix, nerve signaling, and local immune-cell niches. Experimental work suggests that infection history can create long-lived epithelial or stromal "memory" that changes subsequent inflammation. These changes could either improve rapid defense or create a niche favoring bacterial persistence and excessive inflammation. Overall, tissue/epithelial remodeling is supported most directly in UTI models; myeloid training and tolerance are biologically plausible but need better longitudinal human studies with cell-specific epigenetic and functional measurements.

Q18

Question

In bladder cancer, how do tumor-associated neutrophils and macrophages interact within the mucosal tumor microenvironment to influence antigen presentation, T-cell activity, angiogenesis, invasion, and response to immunotherapy?

Response

In bladder cancer, tumor-associated macrophages and neutrophils are highly plastic and can either support or oppose antitumor immunity. Tumor cells and stroma release CSF-1, CCL2, CXCL8, G-CSF, complement signals, and other mediators that recruit monocytes and neutrophils. In many advanced tumors, macrophages adopt immunosuppressive programs characterized by IL-10, TGF-beta, arginase activity, PD-L1, VEGF, and matrix-remodeling enzymes. Neutrophils can add ROS, arginase, MMP-9, VEGF, and NET-related signals.

Together, these cells can reduce effective antigen presentation and suppress T-cell function by nutrient depletion, inhibitory ligands, cytokines, and physical remodeling of the tumor. VEGF and proteases promote angiogenesis, while MMPs and NET-associated factors can facilitate invasion. However, this is not universal: activated macrophages can present antigen and kill tumor cells, and neutrophils can be antitumor under some inflammatory conditions.

Immunotherapy response depends on this balance. A strongly suppressive myeloid microenvironment can limit T-cell entry or function and contribute to resistance to checkpoint blockade. Bladder cancer also illustrates the opposite possibility: intravesical BCG recruits and activates neutrophils, monocytes, macrophages, and dendritic cells, showing that local innate activation can support antitumor immunity. The key therapeutic goal is therefore reprogramming rather than indiscriminate depletion, because the same myeloid populations are also important for urinary mucosal defense against infection.

Q19

Question

Which mechanistically plausible strategies could suppress pathogenic neutrophil recruitment or neutrophil-extracellular-trap-associated injury in ulcerative colitis while preserving antimicrobial defense and macrophage-mediated repair? Explain predicted benefits, trade-offs, and evidence gaps.

Response

A reasonable strategy in ulcerative colitis is to reduce the most tissue-damaging neutrophil signals without eliminating neutrophils completely. One approach is partial blockade of the CXCL8/CXCR1-CXCR2 recruitment axis. This could reduce excessive crypt infiltration and protease burden, but too much inhibition may impair bacterial containment and increase infection risk. Local or short-course delivery would theoretically preserve more systemic defense than continuous systemic blockade.

NET-associated injury can be targeted downstream. DNase can dismantle extracellular DNA scaffolds, while elastase or myeloperoxidase inhibition could reduce toxic NET cargo. PAD4 inhibition may reduce NET formation, but human efficacy and infection safety remain uncertain. An important trade-off is that breaking down NET DNA does not automatically neutralize histones and proteases, and NETs can be useful for trapping microbes.

A complementary approach is to accelerate resolution rather than only suppress recruitment. Enhancing macrophage efferocytosis, MerTK-related clearance pathways, IL-10 signaling, or pro-resolving lipid mediators could shorten neutrophil residence time while preserving the initial antibacterial burst. The major evidence gap is selectivity: most candidate pathways also participate in normal host defense. Future trials would need biomarkers that identify patients with excessive neutrophil/NET activity and should measure infection rates, mucosal healing, and macrophage repair function, not only neutrophil counts.

Q20

Question

How could metabolic or epigenetic reprogramming of intestinal macrophages and granulocytes be used to restore mucosal homeostasis in inflammatory bowel disease, and what mechanisms could cause loss of efficacy or unintended immunosuppression?

Response

Metabolic and epigenetic reprogramming is attractive in inflammatory bowel disease because intestinal myeloid cells are highly state-dependent. Inflammatory macrophages often rely on glycolysis, HIF-1-linked pathways, succinate signaling, and inflammasome activity, whereas repair-oriented macrophages use more mitochondrial oxidative metabolism, lipid handling, and regulatory pathways such as AMPK or PPAR signaling. Neutrophils are strongly glycolytic, and their metabolic state influences survival, ROS production, and NET formation.

Potential strategies include promoting AMPK/PPAR activity, using itaconate-like anti-inflammatory pathways, modulating mTOR, altering short-chain-fatty-acid signaling, or targeting epigenetic enzymes such as BET proteins or selected histone modifiers. In principle, these interventions could reduce inflammatory cytokines and NETs while improving macrophage efferocytosis and epithelial repair. However, most are not specific to intestinal myeloid cells.

Loss of efficacy can occur because inflammatory pathways are redundant and because circulating monocytes continually replace tissue cells. The inflammatory microbiome, hypoxia, or cytokine environment may simply reprogram newly arriving cells back into a pathogenic state. Unintended

immunosuppression is another major risk: suppressing glycolysis, inflammasomes, or trained immune programs too broadly can weaken antibacterial and antifungal defense. The translational challenge is therefore local, cell-selective, and reversible reprogramming rather than permanent systemic suppression. At present, much of this concept remains preclinical or mechanistically inferred from human tissue studies.

Q21

Question

How might biologics that alter type 2 cytokine signaling reshape the eosinophil–mast-cell–macrophage–dendritic-cell network in airway mucosa, beyond simply reducing eosinophil counts?

Response

Biologics that alter type 2 signaling reshape the whole airway myeloid network, not just the eosinophil count. Blocking IL-5 or IL-5 receptor signaling reduces eosinophil maturation, survival, and tissue persistence, which secondarily lowers eosinophil-derived leukotrienes, granule proteins, and cytokines. This reduces epithelial injury and removes signals that can activate macrophages and dendritic cells.

Blocking IL-4/IL-13 signaling changes a broader set of cells. Macrophages receive less IL-4R-alpha signaling, so alternative activation, mucus-associated repair programs, and some remodeling pathways are reduced. Airway epithelium produces fewer type 2 chemokines, and dendritic-cell/Th2 feedback can weaken. Anti-IgE therapy reduces Fc-epsilon-RI signaling on mast cells and basophils, decreasing immediate mediator release and therefore reducing downstream recruitment and antigen-presentation signals. Upstream alarmin blockade, such as TSLP-directed therapy, can reduce dendritic-cell activation and multiple downstream type 2 pathways at once.

These network effects help explain why clinical benefit may exceed the change in blood eosinophil count. They also explain discordance: a patient may have fewer eosinophils but persistent mast-cell activation, structural airway remodeling, or non-type-2 inflammation. Some treatments can transiently change blood eosinophil trafficking without worsening airway disease. Clinically, response is best understood as rewiring of cytokine communication among epithelium, granulocytes, macrophages, dendritic cells, and lymphocytes rather than simple cell depletion.

Q22

Question

What myeloid-directed strategies could interrupt neutrophil–macrophage inflammatory circuits in neutrophilic asthma while preserving antibacterial defense, and which proposed mechanisms are clinically established versus preclinical?

Response

For neutrophilic asthma, the ideal myeloid-directed treatment would reduce pathologic recruitment and activation while leaving enough neutrophil function for bacterial defense. CXCR2 antagonism is a logical strategy because it reduces neutrophil chemotaxis, and clinical studies have shown that airway neutrophil counts can fall. However, clinical benefit has been inconsistent and concerns remain about infection and systemic neutropenia; it is not an established standard therapy.

Other approaches are less mature. Blocking IL-1 or NLRP3 inflammasome signaling could reduce macrophage-to-neutrophil amplification, while inhibiting GM-CSF or selected TLR pathways might shorten neutrophil survival. NET-directed approaches include DNase, PAD4 inhibition, and neutrophil-elastase inhibition. Complement or C5a signaling and metabolic targets are also plausible. Most of these remain preclinical or early translational for asthma, and broad inhibition risks weakening antibacterial defense.

Macrolides are the main clinically used example of a treatment that can help some patients with severe non-type-2 or neutrophilic asthma, but their mechanism is pleiotropic: antimicrobial effects, changes in airway microbiota, and immunomodulation all contribute. They are not a selective neutrophil-macrophage circuit blocker. Therefore, there is currently no clearly established myeloid-specific therapy for neutrophilic asthma comparable to type 2 biologics. The most promising future approach is likely biomarker-guided, partial, and compartment-specific suppression rather than systemic neutrophil depletion.

Q23

Question

Could trained-immunity or mucosal-vaccine approaches beneficially reprogram monocytes or macrophages and neutrophils to reduce recurrent urinary tract infection? Explain the proposed mechanisms, potential tissue-specific risks, and current evidence limitations.

Response

Trained-immunity and mucosal-vaccine approaches could theoretically reduce recurrent UTI by improving the speed and quality of innate responses at the bladder mucosa. A mucosal vaccine may generate pathogen-specific IgA/IgG and T-cell memory, but it may also alter macrophage and neutrophil responses indirectly through cytokines and local stromal signals. Trained immunity could enhance monocyte/macrophage antimicrobial activity through epigenetic and metabolic changes in myeloid cells or bone-marrow progenitors, leading to faster cytokine production and neutrophil recruitment on re-exposure.

The challenge is tissue specificity. An excessively trained response could cause stronger neutrophilic cystitis, epithelial injury, pain, or fibrosis. Repeated stimulation could also induce tolerance instead of training and blunt useful cytokine responses. Broad systemic training might increase inflammatory responses outside the urinary tract. A successful approach would ideally create a controlled local memory that improves early bacterial clearance without prolonging inflammation.

Current evidence is promising but incomplete. Several vaccine or bacterial-extract strategies have reported reductions in recurrent UTI in selected populations, but products, routes, endpoints, and study quality vary. It is usually unclear whether benefit is due to adaptive antibodies/T cells, trained myeloid immunity, nonspecific adjuvant effects, or behavioral and microbiome factors. Direct human evidence showing durable epigenetic reprogramming of bladder monocytes/macrophages or neutrophil progenitors remains limited. Mechanistic trials with mucosal sampling are needed before trained immunity can be considered a proven clinical strategy.

Q24

Question

How could selective reprogramming of tumor-associated macrophages and neutrophils improve antitumor immunity in bladder cancer while minimizing disruption of protective urinary mucosal immunity? Compare mechanistic approaches and key translational barriers.

Response

Selective reprogramming of tumor-associated macrophages and neutrophils in bladder cancer is more attractive than broad depletion because these cells are also needed for urinary tract defense. For macrophages, potential approaches include blocking CSF-1/CSF-1R or CCL2-dependent recruitment, inhibiting PI3K-gamma/TREM2-like suppressive programs, or stimulating phagocytosis and antigen presentation through CD47-SIRP-alpha, TLR, or STING pathways. The goal is to shift macrophages from IL-10/TGF-beta/VEGF-dominant states toward tumor killing and T-cell support.

For neutrophils, CXCL8/CXCR2 or C5a-axis inhibition may reduce recruitment of suppressive tumor-associated neutrophils. NET-directed strategies, neutrophil-elastase inhibition, or metabolic modulation could reduce invasion and T-cell suppression while preserving basic phagocytic capacity. Local intravesical delivery is particularly appealing because it may reprogram the tumor microenvironment while limiting systemic impairment of neutrophil and macrophage function. BCG provides a clinical proof of principle that local innate activation can be therapeutically useful in the bladder.

Major barriers are myeloid heterogeneity and rapid plasticity. The same marker can identify different functional states, and blocking one recruitment pathway may cause compensation through another. Additional problems include infection risk, lack of validated biomarkers, variable drug penetration into tumor tissue, and uncertainty about timing with checkpoint inhibitors or chemotherapy. Translation will likely require spatial profiling and response biomarkers so that only the suppressive myeloid compartment is targeted while protective mucosal immunity is preserved.

Japanese (JP)

Model B | 日本語 | 24 Questions

モデル B

骨髄系－粘膜免疫 | Q01–Q24

Q01

質問

健常な腸管粘膜において、好中球は常在マクロファージおよび樹状細胞と相互作用しながら、どのようにバリア防御に寄与しますか。また、好中球による組織障害を通常どのような機序が抑制していますか。

回答

健常腸管では、好中球は固有層に少数しか存在せず、上皮バリアが破綻して微生物が組織側へ侵入した時に速やかに動員されます。上皮細胞、常在マクロファージ、間質細胞が CXCL1/CXCL2/CXCL8 などの走化性シグナルを出し、好中球は貪食、酸化バースト、抗菌顆粒タンパク、必要に応じて NET 形成によって病原体を制御します。マクロファージは初期の感知と動員に加え、後期には老化･アポトーシス好中球の除去を担います。樹状細胞は主として抗原サンプリングと適応免疫への橋渡しを行います。

健常粘膜で重要なのは、反応が時間的･空間的に限定されることです。粘液層、タイトジャンクション、抗菌ペプチド、速やかな上皮修復により、持続的な好中球動員が不要になります。腸管マクロファージは無害な腔内刺激に対して比較的低反応で、IL-10 などの制御性シグナルを産生します。好中球は役割を終えるとアポトーシスに入り、マクロファージのエフェロサイトーシスによって IL-10、TGF-β、炎症収束性脂質メディエーター、組織修復へ移行します。

さらに、抗プロテアーゼ、抗酸化系、細胞外 DNA を処理する DNase、走化性勾配の消失が組織障害を抑えます。これらが破綻すると、殺菌に有効なプロテアーゼ、ROS、NET 関連ヒストンが陰窩上皮を傷害し、慢性炎症を増幅します。

Q02

質問

健常な腸管粘膜において、好酸球はどのように恒常性の維持に寄与し、マクロファージまたは樹状細胞と情報交換しますか。また、これらの役割は炎症時の好酸球機能とどのように異なりますか。

回答

好酸球は正常腸管固有層の構成細胞で、とくに小腸で比較的多くみられます。恒常状態では IL-5 と eotaxin/CCR3 系により維持され、バリア監視、免疫グロブリン関連応答、組織リモデリングに関与すると考えられています。炎症時の爆発的脱顆粒とは異なり、平常時にはサイトカイン、脂質メディエーター、増殖因子を比較的穏やかに放出します。好酸球由来 IL-4/IL-13 は一部のマクロファージを修復寄りに誘導し得ますが、種差や腸管部位による違いがあります。樹状細胞との直接的な相互作用は抗原提示や局所寛容に関与する可能性がありますが、ヒトでの証拠は限定的です。

炎症時には好酸球の動員と生存が増え、major basic protein、好酸球ペルオキシダーゼ、カチオン性タンパク、ロイコトリエン、炎症性サイトカインを放出します。これらは上皮障害、血管透過性亢進、感覚神経刺激、マクロファージや樹状細胞の活性化変化をもたらします。一方で TGF-βや増殖因子を介した修復作用も残ります。

したがって腸管好酸球を一律に病原的とみなすのは適切ではありません。恒常性維持に寄与する面と、過剰･持続活性化によって炎症や線維化を促す面の両方があり、詳細な細胞間回路はヒトではなお状況依存的です。

Q03
質問
健常な気道粘膜および急性の微生物刺激時に、好中球は肺胞または間質マクロファージ、樹状細胞とどのように協調して宿主防御を担いながら、上皮への二次的損傷を抑えますか。

回答
健常肺では肺胞マクロファージが吸入粒子を処理し、不必要な炎症を抑えることで好中球動員を低く保っています。急性の細菌•真菌刺激では、肺胞および間質マクロファージがパターン認識受容体を介して微生物を感知し、TNF、IL-1、CXCL1/CXCL2/CXCL8 などを放出して好中球を速やかに呼び込みます。樹状細胞は抗原を取り込み、その後の T 細胞応答を形成するとともに、局所の抗菌反応を補助します。

好中球は貪食、酸化バースト、抗菌プロテアーゼ、状況に応じた NET 形成によって迅速に病原体を排除します。初期にはマクロファージと好中球が相互増幅しますが、これが長く続くと上皮障害につながります。

炎症収束には積極的な制御が必要です。アポトーシス好中球をマクロファージがエフェロサイトーシスすると、IL-10、TGF-β、リポキシンやレゾルビンなどの収束性脂質メディエーター、上皮修復が促進されます。抗酸化系、抗プロテアーゼ、粘液クリアランス、DNase も ROS、エラスターゼ、NET 関連傷害を抑えます。臨床的には「十分な動員」だけでなく「適時の除去」が重要です。

Q04
質問
恒常状態および 2 型炎症時の気道粘膜において、好酸球はマクロファージや樹状細胞とどのように相互作用しますか。

回答
恒常状態の気道では好酸球は比較的少なく、肺胞マクロファージと樹状細胞は低炎症環境を維持しています。上皮バリアも吸入抗原と免疫細胞の接触を制限します。好酸球は少量のサイトカインや増殖因子を供給しますが、正常気道での恒常性維持機能は腸管ほど明確ではありません。

2 型炎症では上皮から IL-33、TSLP、IL-25 などのアラーミンが放出され、樹状細胞や 2 型自然リンパ系が活性化します。樹状細胞は Th2 分化を促し、IL-4、IL-5、IL-13 環境を形成します。IL-5 は好酸球の生存を延長し、eotaxin/CCR3 系が組織への動員を促します。活性化好酸球はロイコトリエン、顆粒タンパク、サイトカイン、増殖因子を放出し、樹状細胞活性化や IL-4/IL-13 応答性マクロファージの修復プログラムに影響します。

このネットワークは二面的です。マクロファージの修復機能は上皮回復に有用ですが、持続する 2 型シグナルは粘液産生、気道過敏性、リモデリングを促します。好酸球顆粒タンパクは上皮を直接傷害します。ヒトでの直接的な好酸球—樹状細胞相互作用の重要性は、より広いサイトカインネットワークほど確立していません。

Q05
質問
尿路粘膜における初期の抗菌防御で、好中球は単球またはマクロファージ、樹状細胞とどのように協調しますか。また、過剰な組織障害を防ぐ機序は何ですか。

回答
急性細菌性尿路感染の初期には、尿路上皮と常在骨髄系細胞が TLR などのパターン認識経路で細菌を感知し、IL-6、CXCL8 などを産生します。これにより好中球が強く動員され、上皮を通過して尿腔側へ移動します。好中球は細菌負荷の高い場所で貪食、ROS、抗菌タンパク、状況に応じた NET 形成により殺菌します。

単球•マクロファージは走化性シグナルを出して初期動員を支え、細菌や組織デブリを貪食し、後期にはアポトーシス好中球を除去します。樹状細胞は抗原提示を通じて適応免疫につなげます。一方、マクロファージと好中球の TNF、IL-1 などが過剰になると、殺菌効果以上に組織炎症が強くなることがあります。

組織障害を防ぐには、短時間で細菌量を下げ、その後すぐに炎症を停止することが重要です。好中球アポトーシス、マクロファージのエフェロサイトーシス、IL-10、TGF-β、炎症収束性脂質、尿路上皮再生がバリア回復を促します。好中球が長く残ると、プロテアーゼ、ROS、NET 成分が尿路上皮を傷害し症状を悪化させます。

Q06
質問
膀胱または下部泌尿生殖器粘膜において、肥満細胞由来メディエーターはマクロファージや樹状細胞の機能にどのような影響を与えますか。また、このネットワークは宿主防御と炎症症状の双方にどのように関与し得ますか。

回答
肥満細胞は膀胱•下部泌尿生殖器の血管、神経、上皮の近くに存在するため、放出メディエーターがマクロファージや樹状細胞へすぐ影響します。ヒスタミン、TNF、トリプターゼ、プロスタグランジン、ロイコトリエン、IL-6 などは血管透過性を高め、骨髄系細胞の動員•活性化を促します。マクロファージや樹状細胞のヒスタミン受容体、プロテアーゼ活性化受容体を介して、サイトカイン産生、抗原提示、組織リモデリングも変化します。

感染や損傷時には、局所血流と白血球流入を早めることで宿主防御に役立ちます。マクロファージは貪食、サイトカイン、エフェロサイトーシス、修復を担い、樹状細胞は適応免疫を誘導します。しかし同じ回路が症状も生みます。ヒスタミン、トリプターゼ、プロスタグランジン、ATP 関連シグナル、神経成長因子などが骨盤感覚神経を感作し、尿意切迫、頻尿、疼痛、灼熱感を引き起こし得ます。

刺激が持続すると肥満細胞とマクロファージが正のフィードバックを形成し、上皮バリア低下と神経炎症が続きます。逆に IL-10、TGF-β、修復型マクロファージはこの回路を抑えます。ヒトでの証拠は疾患ごとに異なるため、神経･免疫･血管が連動する機序として理解するのが妥当です。

Q07
質問
急性の腸管粘膜炎症とその後の炎症収束過程において、好中球とマクロファージは、遊走･動員シグナル、サイトカイン、エフェロサイトーシス、脂質メディエーター、組織修復プログラムを介してどのように相互作用しますか。

回答
急性腸管炎症では、マクロファージと上皮細胞が CXCL ケモカイン、IL-1、TNF、補体断片、脂質メディエーターを介して好中球を動員します。好中球は病原体を殺す一方、ROS、プロテアーゼ、炎症性サイトカイン、DAMP を放出し、マクロファージをさらに活性化します。CCL2 などにより単球が流入し、炎症性マクロファージへ分化すると、原因が残っている場合は正のフィードバックが形成されます。

炎症収束は受動的な低下ではなく、能動的な過程です。好中球がアポトーシスに入り、マクロファージがエフェロサイトーシスを行うことが大きな転換点になります。その後、炎症性サイトカインが減少し、IL-10、TGF-β、修復プログラムが増えます。脂質メディエーターも、初期のロイコトリエンやプロスタグランジン優位から、リポキシン、レゾルビン、プロテクチン、マレシンなどへ切り替わります。

修復型マクロファージは上皮再生、血管新生、マトリックス再構築、増殖因子産生を促します。ただし実際の状態は単純な M1/M2 二分ではありません。エフェロサイトーシス不全や持続する微生物刺激があると、本来一過性の好中球反応が慢性粘膜障害へ移行します。

Q08
質問
好酸球由来メディエーターは、腸管粘膜のマクロファージの機能状態や樹状細胞の活性化をどのように変化させますか。また、これらの相互作用は免疫寛容、組織修復、炎症のバランスをどのように変え得ますか。

回答
好酸球はサイトカイン、脂質メディエーター、顆粒タンパク、増殖因子を介して腸管マクロファージと樹状細胞の状態を変えます。IL-4、IL-13 は一部のマクロファージを創傷修復や細胞外マトリックス処理に向かわせ、古典的炎症シグナルを弱める可能性があります。TGF-βや収束性脂質メディエーターも組織修復に寄与し得ます。一方、好酸球ペルオキシダーゼ、カチオン性顆粒タンパク、ロイコトリエン、DAMP はマクロファージを炎症方向に活性化し組織障害を起こします。

樹状細胞への影響も双方向です。穏やかな環境では食物や共生菌抗原に対する寛容性抗原提示を支える可能性がありますが、活動性炎症では好酸球由来メディエーターが樹状細胞の成熟、移動、サイトカイン産生を高め、T 細胞活性化を促します。好酸球自身の抗原提示能も実験的には報告されていますが、ヒト腸管で主経路とは考えにくいです。

最終的な結果は刺激の強さと持続時間で決まります。短期の活性化は修復を助け得ますが、持続的な動員と脱顆粒は寛容—修復のバランスを炎症側へ傾けます。ヒトではなお状況依存性が大きい領域です。

Q09
質問
好中球性気道炎症において、好中球とマクロファージは、サイトカイン、脂質メディエーター、好中球細胞外トラップ、エフェロサイトーシス、代謝シグナルを介してどのように相互作用しますか。また、そのクロストークは炎症の持続と収束をどのように左右しますか。

回答
好中球性気道炎症では、マクロファージが TNF、IL-1、CXCL8、GM-CSF、脂質メディエーターを放出し、好中球の動員と生存を促します。好中球はプロテアーゼ、ROS、アラーミン、LTB4、NET を放出してマクロファージを再活性化します。NET の DNA やヒストンは抗菌的ですが、長く残ると上皮を傷害し、インフラマソームなどの自然免疫経路を刺激します。

エフェロサイトーシスは重要な分岐点です。マクロファージが MerTK 関連経路などでアポトーシス好中球を効率よく取り込むと、炎症性サイトカインが減り、IL-10、TGF-β、炎症収束性脂質、組織修復が増えます。逆に除去が不十分だと好中球は二次性壊死に進み、細胞内酵素が気道へ漏れ出して炎症を長引かせます。

代謝もこの相互作用を規定します。低酸素、乳酸、コハク酸、ミトコンドリアストレス、HIF-1 依存性解糖は炎症状態を維持しやすく、ミトコンドリア代謝と脂質処理の回復はマクロファージの収束･修復に有利です。したがって持続は「再動員･NET･代謝ストレス･除去不全」の連鎖で、収束は病原体減少、走化性低下、NET 処理、エフェロサイトーシス成功によって決まります。

Q10
質問
アレルギー性気道炎症において、肥満細胞と好酸球、さらに該当する場合には好塩基球は、樹状細胞およびマクロファージとどのように相互作用して 2 型免疫を増幅または抑制しますか。

回答
アレルギー性気道炎症では、肥満細胞、好酸球、必要に応じて好塩基球が樹状細胞･マクロファージと連携し 2 型免疫を増幅します。アレルゲン特異的 IgE が肥満細胞 FcεRI を架橋すると、ヒスタミン、トリプターゼ、PGD2、ロイコトリエン、TNF、2 型サイト

カインが放出され、血管透過性、気管支収縮、好酸球･樹状細胞の流入を促します。好塩基球は一部の状況で早期 IL-4 源となります。

樹状細胞はアレルゲンを取り込み Th2 応答を誘導し、IL-4、IL-5、IL-13 環境を作ります。IL-5 は好酸球を維持し、IL-4/IL-13 はマクロファージ、粘液産生、組織リモデリングへ作用します。好酸球顆粒タンパクや脂質メディエーターは上皮を傷害し、抗原提示細胞をさらに活性化し得ます。マクロファージは炎症性サイトカインで増幅する場合と、デブリ除去･修復で抑える場合があります。

抑制には IL-10、TGF-β、抑制性受容体、エフェロサイトーシス、炎症収束性脂質が関与します。慢性喘息ではこれらの終結機構が不十分となり、肥満細胞活性化、好酸球生存、抗原提示、気道リモデリングが相互に持続します。

Q11
質問
急性細菌性尿路感染において、動員された好中球と単球由来マクロファージは、病原体排除、サイトカイン産生、エフェロサイトーシス、粘膜修復の過程でどのように協調または競合しますか。

回答
急性細菌性尿路感染では、好中球が最も速く高い殺菌能を発揮します。尿路上皮や骨髄系細胞のケモカインで動員され、膀胱上皮を越えて尿腔へ移動し、貪食、ROS、顆粒酵素、細胞外トラップで細菌を排除します。単球由来マクロファージはやや遅れて増え、細菌だけでなく組織デブリやアポトーシス好中球も処理し、粘膜修復を調整します。

両者は、マクロファージ由来ケモカインで好中球を呼び込み、その後エフェロサイトーシスで反応を終える点で協調します。一方、過剰な TNF/IL-1 が好中球動員をさらに増やす場合や、好中球の強い脱顆粒が尿路上皮を壊し DAMP を放出してマクロファージを再活性化する場合には競合的･悪循環的になります。細菌が残れば炎症だけが強くなり、殺菌があまり改善しないこともあります。

修復には細菌負荷の低下、好中球アポトーシス、効率的エフェロサイトーシス、ケモカイン減少が必要です。その後マクロファージが IL-10、TGF-β、増殖因子、マトリックス再構築を担います。したがって最適な反応は「初期好中球―後期マクロファージ」という時間的役割分担です。

Q12
質問
慢性膀胱粘膜炎症において、肥満細胞とマクロファージのクロストークは、感覚神経シグナル、血管透過性、上皮バリア機能、そして炎症の持続または収束をどのように変化させ得ますか。

回答
慢性膀胱炎症では、肥満細胞とマクロファージのクロストークが免疫反応を疼痛、血管漏出、バリア障害へつなげます。肥満細胞のヒスタミン、トリプターゼ、プロスタグランジン、ロイコトリエン、TNF、神経成長因子は感覚神経を活性化･感作します。プロテアーゼ活性化受容体やヒスタミン受容体の刺激により、疼痛や尿意切迫の閾値が下がります。マクロファージ由来 IL-1、TNF、IL-6、プロスタグランジンも同じ神経回路を増幅します。

血管ではヒスタミンと炎症性サイトカインが内皮透過性を高め、血漿タンパクや白血球の組織流入を増やします。尿路上皮ではサイトカイン、プロテアーゼ、ROS がタイトジャンクションや表面バリアを弱め、尿中成分が粘膜下神経･免疫細胞に到達しやすくなります。障害上皮から ATP や DAMP が出ると、肥満細胞とマクロファージが再刺激されます。

炎症が収束するかは、マクロファージが TNF/IL-1 優位からエフェロサイトーシス、IL-10/TGF-β、上皮修復へ切り替わるかに左右されます。修復が過剰に持続すれば線維化につながる可能性もあります。ヒト慢性膀胱炎症候群は異質で、単一機序では説明できません。

Q13
質問
潰瘍性大腸炎において、好中球の動員、好中球細胞外トラップ形成、好中球代謝、ならびに好中球–マクロファージ間クロストークは、粘膜障害と炎症収束にそれぞれどのように寄与しますか。

回答
潰瘍性大腸炎では、好中球動員は活動性粘膜炎症の代表的所見です。上皮･骨髄系細胞由来 CXCL8 関連ケモカイン、補体、接着分子が好中球を陰窩へ誘導し、陰窩膿瘍を形成します。好中球は細菌移行を抑える点では有益ですが、ROS、エラスターゼ、ミエロペルオキシダーゼ産物、各種プロテアーゼが過剰になると上皮を直接傷害します。

NET も二面的です。細胞外 DNA と抗菌タンパクは微生物を捕捉しますが、ヒストン、プロテアーゼ、残存 DNA は上皮障害と自然免疫活性化を促します。好中球は解糖依存性が高く、低酸素粘膜でも活動できますが、長期の代謝ストレスは炎症状態を維持させる可能性があります。

マクロファージとのクロストークが最終転帰を決めます。初期 TNF、IL-1、ケモカインは好中球流入を維持し、後期のエフェロサイトーシス成功は IL-10、TGF-β、炎症収束性脂質、上皮修復を促します。除去不全や微生物刺激の持続があると回路は閉じません。したがって好中球を完全に除くより、過剰動員や NET 毒性を選択的に抑える方が理論的に望ましいです。

Q14

質問

クローン病において、好中球、単球由来マクロファージ、樹状細胞の相互作用の異常は、微生物処理、抗原提示、炎症の持続、上皮修復をどのように変化させますか。

回答

クローン病では、微生物処理不全と過剰で持続的な免疫活性化が同時に起こります。上皮バリア破綻部には好中球が動員されますが、患者によっては初期自然免疫の遅延･低効率がみられる一方、成立した病変では高度に活性化した好中球が ROS やプロテアーゼを放出します。NOD2、自食作用関連経路、上皮防御の異常は細菌感知と除去を変化させます。

流入単球は炎症性マクロファージへ分化し、TNF、IL-1、IL-6、IL-23 を産生します。細菌成分が残ると、エフェロサイトーシスや修復へ移行できず炎症が続きます。樹状細胞は腔内･組織抗原を処理し、IL-12/IL-23 系を通じて Th1/Th17 応答を強めます。微生物刺激、マクロファージサイトカイン、T 細胞反応、好中球動員が互いに維持されます。

過剰 TNF、ROS、プロテアーゼ、マトリックス分解酵素は上皮修復を遅らせ、慢性的なマクロファージ･間質細胞活性化は線維化につながります。一方、IL-10、TGF-β、増殖因子、好中球除去は正常治癒に必須です。つまり一つの細胞が悪いのではなく、細胞間の時間的協調が崩れていることが本質です。

Q15

質問

重症好中球性喘息において、好中球、マクロファージ、樹状細胞はどのように自己持続的な炎症回路を形成しますか。また、好中球細胞外トラップ、インフラマソームシグナル、微生物由来シグナル、ステロイド抵抗性はどのような役割を果たし得ますか。

回答

重症好中球性喘息では、上皮、好中球、マクロファージ、樹状細胞、IL-17 系 T 細胞が自己増幅回路を作り得ます。微生物由来シグナル、上皮障害、IL-1、TNF が CXCL8 や G-CSF を誘導し、好中球の動員と生存を延長します。マクロファージは IL-1βなどインフラマソーム関連サイトカインを加え、樹状細胞は IL-17 産生 T 細胞を促します。IL-17 は再び上皮･間質から好中球ケモカインを増やします。

NET はこの回路の増幅因子になり得ます。細胞外 DNA、ヒストン、好中球エラスターゼ、MPO 産物は上皮を傷害し、自然免疫センサーを刺激します。NLRP3 インフラマソームは機序的に妥当で実験的支持もありますが、全患者で同程度に重要とは限りません。慢性細菌定着や気道ディスバイオーシスは反復する TLR 刺激源になります。

ステロイド抵抗性は多因子で、IL-17 優位炎症、酸化ストレス、p38/MAPK 異常、糖質コルチコイド受容体機能低下、HDAC2 低下などが関与します。ステロイドは好中球アポトーシスを遅らせることもあります。臨床的に確立しているのは非 2 型の異質性であり、NET やインフラマソーム標的はまだ標準治療ではありません。

Q16

質問

好酸球性喘息において、好酸球、肥満細胞、さらに該当する場合には好塩基球は、マクロファージ、樹状細胞、気道上皮とどのように相互作用して 2 型炎症を開始･増幅･収束させますか。

回答

好酸球性喘息は、気道上皮がアレルゲン、ウイルス、汚染物質を感知するところから始まることが多いです。上皮は TSLP、IL-33、IL-25 を放出し、樹状細胞と 2 型自然免疫を活性化します。樹状細胞は Th2 応答を誘導し、ILC2 も IL-5、IL-13 を供給します。IL-5 は好酸球産生と生存を促し、eotaxin が気道への流入を誘導します。肥満細胞は特異的 IgE で活性化され、ヒスタミン、ロイコトリエン、PGD2、サイトカインを放出します。状況により好塩基球も IL-4 源となります。

増幅期には好酸球顆粒タンパク、脂質メディエーター、サイトカインが上皮傷害と気道過敏性を高めます。IL-4/IL-13 はマクロファージと上皮に作用し、粘液、ケモカイン、修復･リモデリングを促します。マクロファージはデブリを除去しますが、慢性 2 型刺激下では増殖因子や線維化に関与することもあります。樹状細胞は抗原提示と記憶 Th2 応答を維持します。

収束には誘因除去、好酸球アポトーシスまたは組織からの離脱、マクロファージのエフェロサイトーシス、上皮バリア回復、制御性サイトカイン･収束性脂質の増加が必要です。好酸球が減っても、肥満細胞や構造的リモデリングが残れば症状は続き得ます。

Q17

質問

反復性尿路感染において、過去の感染は、訓練免疫、免疫寛容、または組織リモデリングを介して膀胱粘膜の好中球および単球 / マクロファージ応答をどのように再構成し得ますか。また、それぞれの機序を支持するエビデンスはどの程度確立されていますか。

回答

過去の尿路感染は、訓練免疫、免疫寛容、組織リモデリングを介して次回の好中球･単球/マクロファージ応答を変え得ますが、エビデンスの強さは同じではありません。訓練免疫は、自然免疫細胞や前駆細胞にエピジェネティック･代謝変化が残り、再刺激時により速く強く反応する現象です。一般免疫学としてはよく確立し、動物感染モデルも長期の自然免疫再プログラムを支持しますが、ヒト反復性 UTI で「膀胱骨髄系訓練」が主要因と直接示したデータはまだ少ないです。

免疫寛容は逆で、反復する TLR 刺激により TNF、IL-1 などの反応が低下し、走化性や殺菌能が鈍ることがあります。エンドトキシン寛容そのものは確立した現象ですが、一般的な反復性 UTI での臨床的重要性は不明です。同一個体でも細胞集団ごとに訓練と寛容が併存し得ます。

組織リモデリングは膀胱でより直接的です。感染歴は尿路上皮分化、バリア、細胞外マトリックス、神経、局所免疫ニッチを変えます。実験的には上皮･間質に長期の“記憶”が残ることが示唆されます。現時点では組織/上皮リモデリングの UTI モデル証拠が比較的強く、骨髄系訓練･寛容は縦断的ヒト研究が必要です。

Q18

質問

膀胱がんにおいて、腫瘍関連好中球と腫瘍関連マクロファージは粘膜腫瘍微小環境内でどのように相互作用し、抗原提示、T 細胞活性、血管新生、浸潤、免疫療法への反応に影響しますか。

回答

膀胱がんの腫瘍関連マクロファージと好中球は高い可塑性を持ち、抗腫瘍にも腫瘍促進にも働きます。腫瘍と間質は CSF-1、CCL2、CXCL8、G-CSF、補体関連シグナルなどを出して単球･好中球を動員します。進行腫瘍ではマクロファージが IL-10、TGF-β、アルギナーゼ、PD-L1、VEGF、マトリックス分解酵素に富む免疫抑制状態をとることが多く、好中球も ROS、アルギナーゼ、MMP-9、VEGF、NET 関連因子を加えます。

両者は栄養枯渇、抑制性リガンド、サイトカイン、腫瘍間質リモデリングによって T 細胞機能と有効な抗原提示を低下させます。VEGF とプロテアーゼは血管新生を促し、MMP や NET は浸潤を助けます。ただし常に腫瘍促進的とは限らず、適切に活性化されたマクロファージは抗原提示と腫瘍殺傷を行い、好中球も抗腫瘍的に働く場合があります。

免疫療法への反応はこのバランスに左右されます。抑制的骨髄系微小環境は T 細胞流入･機能を妨げます。一方、膀胱内 BCG は好中球、単球/マクロファージ、樹状細胞を局所活性化し、自然免疫が抗腫瘍に利用できることを示します。したがって目標は単純な除去ではなく再プログラムです。

Q19

質問

潰瘍性大腸炎において、病原性の好中球動員または好中球細胞外トラップ関連障害を抑えつつ、抗微生物防御とマクロファージによる修復を可能な限り温存するには、どのような機序的に妥当な戦略が考えられますか。予想される利点、トレードオフ、エビデンスの不足を説明してください。

回答

潰瘍性大腸炎では、好中球そのものを消すのではなく、組織傷害性の高い反応だけを弱める戦略が合理的です。第一に CXCL8/CXCR1-CXCR2 動員軸を部分的に抑えると、陰窩への過剰流入とプロテアーゼ負荷を減らせます。ただし強すぎる抑制は細菌封じ込めを損ない感染リスクを上げるため、局所投与や短期投与の方が理論上安全です。

NET 関連傷害には、DNase で DNA 骨格を分解する方法、好中球エラスターゼや MPO を抑える方法、PAD4 阻害で NET 形成を減らす方法があります。ただしヒトでの有効性と感染安全性はまだ十分ではありません。DNA を分解してもヒストンやプロテアーゼが自動的に無毒化されるわけではなく、NET 自体が一部の感染では有益である点がトレードオフです。

別の方向は炎症収束を早めることです。マクロファージのエフェロサイトーシス、MerTK 関連経路、IL-10、炎症収束性脂質メディエーターを強めれば、初期の抗菌バーストを残しながら好中球滞在時間を短縮できる可能性があります。今後は感染率、粘膜治癒、NET 指標、マクロファージ修復能を同時に評価する必要があります。

Q20

質問

炎症性腸疾患において、腸管マクロファージと顆粒球の代謝的またはエピジェネティックなリプログラミングを粘膜恒常性の回復にどのように利用できるでしょうか。また、効果消失や意図しない免疫抑制をもたらし得る機序は何ですか。

回答

炎症性腸疾患で代謝･エピジェネティック再プログラムが注目されるのは、骨髄系細胞の機能が代謝状態に強く依存するためです。炎症性マクロファージは解糖、HIF-1 系、コハク酸、インフラマソームに傾き、修復型マクロファージはミトコンドリア酸化代謝、脂質処理、AMPK/PPAR 系をより利用します。好中球はもともと解糖依存性が高く、その代謝は生存、ROS、NET 形成に影響します。

候補としては AMPK/PPAR 活性化、itaconate 様抗炎症経路、mTOR 調節、短鎖脂肪酸シグナル、BET タンパクや特定ヒストン修飾酵素の標的化などがあります。理論上は炎症性サイトカインと NET を減らし、マクロファージのエフェロサイトーシスと上皮修復を改善できます。ただし腸管骨髄系細胞だけに選択的な標的は少ないです。

効果が失われる理由には経路の冗長性、循環単球の継続流入、炎症性微生物叢･低酸素･サイトカイン環境による再教育があります。さらに解糖やインフラマソーム、訓練免疫を広く抑えると細菌･真菌防御まで落ちます。したがって必要なのは局所的、細胞選択的、可逆的な再プログラムであり、多くはまだ前臨床段階です。

Q21

質問

2 型サイトカインシグナルを変化させる生物学的製剤は、単に好酸球数を減らすだけでなく、気道粘膜の好酸球–肥満細胞–マクロファージ–樹状細胞ネットワークをどのように再構成し得ますか。

回答

2 型サイトカインを標的とする生物学的製剤は、好酸球数だけでなく気道の骨髄系ネットワーク全体を変えます。IL-5/IL-5 受容体系を遮断すると、好酸球の成熟、生存、組織滞留が減り、ロイコトリエン、顆粒タンパク、サイトカインも減少します。その結果、上皮傷害やマクロファージ･樹状細胞への二次刺激が弱まります。

IL-4/IL-13系を遮断するとより広い影響が出ます。マクロファージのIL-4Rαシグナルが減るため、代替活性化、粘液関連修復、リモデリングの一部が弱まります。上皮の2型ケモカイン産生も減り、樹状細胞—Th2フィードバックが弱くなります。抗IgE療法は肥満細胞・好塩基球のFcεRI活性化を抑え、即時メディエーターとその後の骨髄系動員を減らします。TSLPなど上流アラーミンの遮断は樹状細胞活性化と複数の下流2型経路を同時に弱めます。

このため、臨床効果は血中好酸球減少以上に大きい場合があります。逆に好酸球が減っても、肥満細胞活性化、構造的リモデリング、非2型炎症が残れば症状は持続します。つまり「細胞数の減少」より「ネットワーク配線の変更」と考える方が実態に近いです。

Q22

質問

好中球性喘息において、抗菌防御を維持しながら好中球–マクロファージ炎症回路を遮断するために、どのような骨髄系細胞標的戦略が考えられますか。また、提案されている機序のうち、臨床的に確立しているものと前臨床段階にあるものを区別してください。

回答

好中球性喘息では、病的な動員・活性化を抑えつつ、細菌防御に必要な好中球機能を残す必要があります。CXCR2拮抗は最も直接的で、気道好中球を減らせることは臨床研究で示されていますが、症状や転帰の改善は一貫せず、感染や全身性好中球減少の懸念もあるため標準治療ではありません。

IL-1やNLRP3インフラマソームの阻害はマクロファージから好中球への増幅を抑える可能性があり、GM-CSFや一部TLR経路の抑制は好中球生存を短くする可能性があります。NETを標的とするDNase、PAD4阻害、好中球エラスターゼ阻害、さらに補体/C5aや代謝標的も機序的には妥当です。しかし喘息では多くが前臨床または初期トランスレーショナル段階で、広範な抑制は抗菌防御低下につながります。

臨床的にはマクロライドが一部の重症非2型/好中球性喘息で有効ですが、抗菌作用、気道微生物叢変化、免疫調節が混在しており、選択的な好中球—マクロファージ回路阻害ではありません。現時点で2型生物学的製剤に相当する確立した骨髄系特異治療はなく、将来はバイオマーカーに基づく部分的・局所的抑制が現実的です。

Q23

質問

訓練免疫または粘膜ワクチンのアプローチによって、単球 / マクロファージと好中球を有益にリプログラムし、反復性尿路感染を減らすことは可能でしょうか。想定される機序、組織特異的な潜在的リスク、現時点のエビデンスの限界を説明してください。

回答

訓練免疫や粘膜ワクチンは、膀胱粘膜での初期応答を速く質の高いものにすることで反復性UTIを減らす可能性があります。粘膜ワクチンは病原体特異的IgA/IgGやT細胞記憶を作るだけでなく、局所サイトカイン・間質シグナルを介してマクロファージと好中球応答を間接的に変える可能性があります。訓練免疫では単球/マクロファージや骨髄前駆細胞にエピジェネティック・代謝変化が入り、再暴露時により速くサイトカインを産生し好中球を動員できると考えられます。

問題は組織特異性です。訓練が強すぎると好中球性膀胱炎、上皮障害、疼痛、線維化を悪化させる可能性があります。反復刺激が訓練ではなく寛容を誘導し、必要なサイトカイン反応を弱めることもあります。全身性の訓練は尿路以外の炎症を増やし得ます。

現在のエビデンスは有望ですが不完全です。一部のワクチン・細菌抽出物で再発減少が報告されていますが、製剤、投与経路、評価項目、試験品質は不均一です。効果が抗体/T細胞、骨髄系訓練、非特異的アジュバント効果、微生物叢変化のどれによるかも明確ではありません。ヒト膀胱骨髄系細胞の持続的エピジェネティック再プログラムを直接示すデータはまだ限られます。

Q24

質問

腫瘍関連マクロファージと好中球を選択的にリプログラムすることで、保護的な尿路粘膜免疫への影響を最小限にしつつ、膀胱がんの抗腫瘍免疫をどのように高められるでしょうか。機序的アプローチと主要なトランスレーショナル上の障壁を比較してください。

回答

膀胱がんでは、腫瘍関連マクロファージと好中球を広く除去するより選択的に再プログラムする方が望ましいです。これらは尿路感染防御にも必要だからです。マクロファージではCSF-1/CSF-1RやCCL2依存性動員の阻害、PI3Kγ・TREM2様の抑制プログラムの解除、CD47-SIRPα、TLR、STING経路を利用した貪食・抗原提示増強が候補です。目標はIL-10/TGF-β/VEGF優位の状態から、腫瘍殺傷とT細胞支援へ移すことです。

好中球ではCXCL8/CXCR2やC5a軸を抑えて免疫抑制性TANの流入を減らし、NET、好中球エラスターゼ、代謝経路を標的にして浸潤・T細胞抑制を減らす方法が考えられます。膀胱内投与は、全身の好中球・マクロファージ機能を比較的温存しながら腫瘍局所を変えられる点で魅力があります。BCGは局所自然免疫活性化が治療に利用できることを示す臨床的な概念実証です。

主な壁は骨髄系細胞の異質性と可塑性、経路の代償、感染リスク、信頼できるバイオマーカー不足、腫瘍深部への薬剤到達、免疫チェックポイント阻害薬や化学療法との最適な順序です。将来は空間プロファイリングと機能バイオマーカーで、抑制的な骨髄系サブセットだけを選んで介入する必要があります。

Simplified Chinese (SC)

Model B | 简体中文 | 24 Questions

模型 B

髓系－黏膜免疫 / Q01–Q24

Q01

问题

在健康肠黏膜中，中性粒细胞如何在与驻留巨噬细胞和树突状细胞相互作用的同时参与屏障防御？哪些机制通常限制中性粒细胞介导的组织损伤？

回答

健康肠黏膜内的中性粒细胞数量通常很低，真正发生上皮屏障破损或微生物越界时才会迅速募集。上皮细胞、驻留巨噬细胞以及基质细胞可产生 CXCL1、CXCL2、CXCL8 等趋化信号。募集来的中性粒细胞通过吞噬、氧爆发、抗菌颗粒蛋白以及在高病原负荷时形成胞外诱捕网来控制细菌。驻留巨噬细胞既参与早期感知和募集，也负责后期清除衰老或凋亡的中性粒细胞；树突状细胞则更偏向抗原采样和后续适应性免疫。

健康状态的关键不是“没有中性粒细胞”，而是反应被限制在合适的时间和空间。完整黏液层、紧密连接、抗菌肽和快速上皮修复减少持续招募的需要。肠道巨噬细胞对无害腔内刺激相对低反应，并可产生 IL-10 等调节信号。中性粒细胞完成任务后进入凋亡，巨噬细胞通过凋亡细胞清除转向 IL-10、TGF-β、促消退脂质介质和组织修复程序。

此外，抗蛋白酶、抗氧化系统、清除胞外 DNA 的 DNase 以及趋化梯度的终止都可限制损伤。若这些“刹车”失效，中性粒细胞用于杀菌的蛋白酶、氧化物和 NET 相关组蛋白也会损伤隐窝上皮并推动慢性炎症。

Q02

问题

在健康肠黏膜中，嗜酸性粒细胞如何参与维持稳态并与巨噬细胞或树突状细胞进行通讯？这些作用与炎症状态下嗜酸性粒细胞的功能有何不同？

回答

嗜酸性粒细胞本身就是正常肠固有层的一部分，在小肠尤其明显。稳态下，它们主要受 IL-5 和 eotaxin/CCR3 轴维持，可参与屏障监视、免疫球蛋白相关反应和组织重塑。与炎症时剧烈脱颗粒不同，稳态嗜酸性粒细胞可较温和地释放细胞因子、脂质介质和生长因子。其 IL-4、IL-13 等信号可使部分巨噬细胞偏向修复型功能，但这种作用在人和不同肠段中并不完全一致。对树突状细胞的影响可能涉及抗原呈递和局部耐受，但人体直接证据相对有限。

进入炎症状态后，嗜酸性粒细胞募集增多、存活延长，并释放主要碱性蛋白、嗜酸性粒细胞过氧化物酶、阳离子蛋白、白三烯以及炎性细胞因子。这些介质可损伤上皮、增加血管通透性、刺激感觉神经，并改变巨噬细胞和树突状细胞的激活状态。同时，它们仍可能通过 TGF-β和生长因子参与修复。

因此，临床上不宜把肠道嗜酸性粒细胞简单视为“有害细胞”。生理状态下可能有稳态作用，过度或持续激活时则可把同一网络推向炎症和纤维化。很多精细的细胞间机制在动物模型中证据更强，在人肠道中仍具有明显的情境依赖性。

Q03

问题

在健康呼吸道黏膜以及急性微生物攻击期间，中性粒细胞如何与肺泡或间质巨噬细胞及树突状细胞协同，在发挥宿主防御作用的同时限制对上皮的旁观者损伤？

回答

健康肺内，肺泡巨噬细胞会清除吸入颗粒并抑制不必要的炎症，从而使中性粒细胞募集保持在低水平。急性细菌或真菌刺激时，肺泡和间质巨噬细胞通过模式识别受体感知病原，释放 TNF、IL-1、CXCL1/CXCL2/CXCL8 等介质，迅速招募中性粒细胞。树突状细胞负责抗原采样并塑造后续 T 细胞反应，也可通过细胞因子增强局部抗感染反应。

中性粒细胞依靠吞噬、氧爆发、抗菌蛋白酶和在部分情境下形成 NET 来快速杀菌。早期巨噬细胞与中性粒细胞之间可互相放大：巨噬细胞趋化因子招募中性粒细胞，而中性粒细胞释放的危险信号又能进一步激活巨噬细胞。这在高病原负荷时有利，但持续过久会转为组织损伤。

炎症消退需要主动“刹车”。中性粒细胞凋亡后被巨噬细胞清除，可促进 IL-10、TGF-β、脂氧素/消退素等促消退介质和上皮修复。气道抗氧化系统、抗蛋白酶、黏液清除和 DNase 可限制氧化物、弹性蛋白酶和 NET 相关损伤。临床上，关键是“及时募集、及时清除”，而不是单纯追求更多中性粒细胞。

Q04

问题

在稳态条件和 2 型炎症期间，嗜酸性粒细胞如何在气道黏膜中与巨噬细胞和树突状细胞相互作用？

回答

稳态气道中的嗜酸性粒细胞数量较少，肺泡巨噬细胞和树突状细胞总体维持低炎症环境，上皮屏障也减少吸入抗原与免疫细胞的直接接触。此时嗜酸性粒细胞可提供少量细胞因子和生长因子，但其稳态作用通常不像肠道那样突出。

2 型炎症时，上皮细胞释放 IL-33、TSLP、IL-25 等警报素，激活树突状细胞和 2 型先天淋巴样细胞。树突状细胞促进 Th2 分化，形成 IL-4、IL-5 和 IL-13 环境；IL-5 延长嗜酸性粒细胞存活，eotaxin 通过 CCR3 促进其募集。活化嗜酸性粒细胞释放白三烯、颗粒蛋白、细胞因子和生长因子，可进一步影响树突状细胞，并使巨噬细胞偏向 IL-4/IL-13 反应性修复程序。

这类互作既有保护性也有致病性。巨噬细胞的修复程序有助于清除碎片和恢复上皮，但持续的 2 型信号也可促进黏液分泌、气道高反应性和重塑；嗜酸性粒细胞颗粒蛋白还能直接损伤上皮。因此，急性反应可在同一网络中被终止，而反复刺激时则可能演变为慢性哮喘。

Q05

问题

在尿路黏膜中，早期抗菌防御期间，中性粒细胞如何与单核细胞或巨噬细胞及树突状细胞协同？哪些机制可防止过度组织损伤？

回答

急性细菌性尿路感染早期，尿路上皮和驻留髓系细胞通过 TLR 等模式识别通路感知细菌，迅速产生 IL-6、CXCL8 等趋化信号，形成明显的中性粒细胞募集。中性粒细胞穿过尿路上皮进入尿腔，在细菌负荷最高的位置进行吞噬、氧爆发、释放抗菌蛋白，并在部分情况下形成胞外诱捕网。

单核细胞和巨噬细胞通过释放趋化因子支持早期募集，同时吞噬细菌和组织碎片，后期还负责清除凋亡中性粒细胞。树突状细胞参与抗原呈递并把先天免疫与适应性免疫连接起来。若巨噬细胞或中性粒细胞产生过多 TNF、IL-1 等炎症介质，可能只增加组织炎症而不再改善细菌清除。

限制组织损伤的核心是“快速杀菌后快速关闭”。中性粒细胞凋亡、巨噬细胞凋亡细胞清除、IL-10、TGF-β、促消退脂质介质以及尿路上皮再生共同帮助恢复屏障。若中性粒细胞持续存在，其蛋白酶、氧化物和 NET 成分可损伤尿路上皮并加重尿频、尿痛等症状。

Q06

问题

在膀胱或下泌尿生殖道黏膜中，肥大细胞释放的介质如何影响巨噬细胞和树突状细胞的功能？这一网络如何同时参与宿主防御和炎症相关症状？

回答

肥大细胞位于膀胱和下泌尿生殖道的血管、神经和上皮附近，因此其介质能很快影响巨噬细胞和树突状细胞。组胺、TNF、胰蛋白酶样蛋白酶、前列腺素、白三烯、IL-6 等可增加血管通透性，并促进髓系细胞募集和激活。巨噬细胞、树突状细胞上的组胺受体和蛋白酶激活受体还能改变其细胞因子产生、抗原呈递和组织重塑功能。

感染或组织损伤时，这种反应有利于提高局部血流和白细胞进入，巨噬细胞进一步完成吞噬、细胞因子产生、凋亡细胞清除和修复，树突状细胞则推动适应性免疫。但同一网络也会产生症状。组胺、胰蛋白酶样蛋白酶、前列腺素、ATP 相关信号和神经营养因子可使盆腔感觉神经敏化，出现尿急、尿频、疼痛或烧灼感；血管渗漏还会形成水肿并进一步放大炎症。

若刺激持续，肥大细胞和巨噬细胞之间可形成正反馈，削弱上皮屏障并维持神经炎症。相反，IL-10、TGF-β及修复型巨噬细胞可抑制这一回路。不同膀胱炎综合征的人体证据并不完全一致，因此更适合把它理解为神经—免疫—血管共同参与的机制框架。

Q07

问题

在急性肠黏膜炎症及其后的炎症消退阶段，中性粒细胞与巨噬细胞如何通过募集信号、细胞因子、凋亡细胞清除、脂质介质和组织修复程序相互影响？

回答

急性肠黏膜炎症时，巨噬细胞和上皮细胞通过 CXCL 趋化因子、IL-1、TNF、补体片段和脂质介质募集中性粒细胞。中性粒细胞杀菌的同时会释放 ROS、蛋白酶、炎性细胞因子和损伤相关信号，进一步激活巨噬细胞。CCL2 等信号还可募集单核细胞并分化为炎性巨噬细胞；如果病原或损伤没有被控制，就会形成正反馈。

炎症消退不是“自然停止”，而是主动程序。中性粒细胞进入凋亡后，巨噬细胞对其进行凋亡细胞清除，这是关键转折点。清除后，巨噬细胞降低炎性细胞因子，增加 IL-10、TGF-β和修复相关程序。同时发生脂质介质“类别转换”：早期白三烯、前列腺素为主，随后更多出现脂氧素、消退素、保护素和 maresin，减少进一步募集并促进清除。

修复型巨噬细胞促进上皮复原、血管生成、基质重塑和生长因子产生。但这些状态并不是简单的 M1/M2 二分，而是受微生物负荷、缺氧和局部细胞因子共同决定。凋亡细胞清除失败或微生物刺激持续，就会把本来短暂的中性粒细胞反应转成慢性黏膜损伤。

Q08

问题

嗜酸性粒细胞来源的介质如何改变肠黏膜巨噬细胞的功能状态和树突状细胞的激活？这些相互作用如何推动免疫耐受、组织修复与炎症之间的平衡发生变化？

回答

嗜酸性粒细胞可通过细胞因子、脂质介质、颗粒蛋白和生长因子改变肠黏膜中的巨噬细胞和树突状细胞。IL-4、IL-13 可使部分巨噬细胞偏向伤口修复、细胞外基质处理和较低的经典炎症反应；嗜酸性粒细胞还可能通过 TGF-β、调节性脂质介质等促进组织恢复。另一方面，嗜酸性粒细胞过氧化物酶、阳离子颗粒蛋白、白三烯和危险信号也可增强巨噬细胞炎症并造成组织损伤。

对树突状细胞的影响同样具有双向性。相对温和的嗜酸性粒细胞环境可能有利于耐受性抗原呈递，维持对食物或共生菌抗原的低反应；活动性炎症时，嗜酸性粒细胞介质可促进树突状细胞成熟、迁移或细胞因子释放，从而增强 T 细胞激活。实验中虽有嗜酸性粒细胞参与抗原呈递的报道，但在人肠道中不太可能是主导途径。

因此，最终结果取决于刺激强度和持续时间。短暂激活可参与修复，长期募集和脱颗粒则更容易把耐受—修复平衡推向炎症。人类直接证据仍不如动物和离体研究充分。

Q09
问题
在中性粒细胞性气道炎症中，中性粒细胞与巨噬细胞如何通过细胞因子、脂质介质、中性粒细胞胞外诱捕网、凋亡细胞清除和代谢信号相互影响？这种串扰如何决定炎症持续还是消退？

回答

中性粒细胞性气道炎症中，巨噬细胞可释放 TNF、IL-1、CXCL8、GM-CSF 以及脂质介质，促进中性粒细胞募集并延长其存活。中性粒细胞反过来释放蛋白酶、ROS、警报素、LTB4 和 NET，进一步激活巨噬细胞。NET 中的 DNA 和组蛋白具有抗菌作用，但持续存在时可损伤上皮，并刺激炎症小体或其他先天免疫通路。

凋亡细胞清除是决定走向的重要节点。巨噬细胞通过 MerTK 等相关受体高效吞噬凋亡中性粒细胞后，会降低炎性细胞因子，并增加 IL-10、TGF-β、促消退脂质介质和组织修复。若清除受损，中性粒细胞可发生继发坏死，蛋白酶和胞内成分进入气道，反而延长炎症。

代谢也参与调节。缺氧、乳酸、琥珀酸、线粒体应激和 HIF-1 相关糖酵解程序可维持炎性巨噬细胞和中性粒细胞状态；而线粒体代谢和脂质处理恢复则更有利于巨噬细胞进入修复。换句话说，持续取决于"继续招募+NET+代谢应激+清除失败"的闭环，而消退依赖病原下降、趋化减弱、NET 清除和巨噬细胞向修复转换。

Q10
问题
在过敏性气道炎症中，肥大细胞和嗜酸性粒细胞（在相关情境下包括嗜碱性粒细胞）如何与树突状细胞和巨噬细胞相互作用，从而放大或限制 2 型免疫反应？

回答

过敏性气道炎症中，肥大细胞、嗜酸性粒细胞以及部分情境下的嗜碱性粒细胞，与树突状细胞和巨噬细胞共同放大 2 型免疫。过敏原特异性 IgE 交联肥大细胞 FcεRI 后，可释放组胺、胰蛋白酶样蛋白酶、PGD2、白三烯、TNF 和 2 型细胞因子，引起血管通透性增加、支气管收缩，并促进嗜酸性粒细胞和树突状细胞进入。嗜碱性粒细胞在某些情况下可提供早期 IL-4，但其重要性因疾病阶段而异。

树突状细胞摄取过敏原并诱导 Th2 反应，产生 IL-4、IL-5、IL-13。IL-5 维持嗜酸性粒细胞，IL-4/IL-13 则影响巨噬细胞、黏液分泌和组织重塑。嗜酸性粒细胞释放颗粒蛋白和脂质介质，既可损伤上皮，也可进一步增强抗原呈递细胞的激活。巨噬细胞既可能通过炎症介质放大反应，也可能清除碎片并参与屏障修复。

限制机制包括 IL-10、TGF-β、抑制性受体信号、凋亡细胞清除和促消退脂质介质。慢性哮喘常反映这些终止机制不足，使肥大细胞激活、嗜酸性粒细胞存活、持续抗原呈递和气道重塑彼此维持。

Q11
问题
在急性细菌性尿路感染期间，募集到局部的中性粒细胞和单核细胞来源巨噬细胞如何在病原体清除、细胞因子产生、凋亡细胞清除和黏膜修复过程中协同或竞争？

回答

急性细菌性尿路感染中，中性粒细胞是最快、杀菌能力最强的一线效应细胞。它们受到尿路上皮和髓系细胞趋化因子募集，穿过膀胱上皮后进入尿腔，通过吞噬、氧化物、颗粒酶和胞外诱捕机制清除细菌。单核细胞来源巨噬细胞通常稍后增加，功能更"综合"：既吞噬细菌，也清除组织碎片和凋亡中性粒细胞，并参与修复。

二者协同时，巨噬细胞通过趋化信号帮助中性粒细胞到位，后期再通过凋亡细胞清除结束反应。它们也可能发生功能竞争。巨噬细胞过多产生 TNF、IL-1 会继续拉高中性粒细胞募集；而中性粒细胞过度脱颗粒可破坏尿路上皮并释放 DAMP，使巨噬细胞持续激活。如果细菌没有真正清除，就容易形成"炎症更多、杀菌改善不明显"的状态。

修复阶段需要细菌负荷下降、中性粒细胞凋亡、巨噬细胞高效清除以及趋化因子减少。随后巨噬细胞增加 IL-10、TGF-β、生长因子和基质重塑，促进上皮复原。因此更合适的理解是"先由中性粒细胞快速杀菌，后由巨噬细胞清场和修复"，而不是简单比较谁更重要。

Q12
问题
在慢性膀胱黏膜炎症中，肥大细胞与巨噬细胞之间的串扰可能如何改变感觉神经信号、血管通透性、上皮屏障功能以及炎症的持续或消退？

回答

慢性膀胱炎症中，肥大细胞—巨噬细胞串扰可把免疫反应直接连接到疼痛、血管渗漏和屏障损伤。肥大细胞释放的组胺、胰蛋白酶样蛋白酶、前列腺素、白三烯、TNF 和神经生长因子可激活或敏化感觉神经；蛋白酶激活受体和组胺受体信号可降低疼痛和尿急阈值。巨噬细胞来源 IL-1、TNF、IL-6 和前列腺素也会加强这一神经敏化。

这些介质同时作用于血管和上皮。组胺和炎性细胞因子提高内皮通透性，使血浆蛋白和更多白细胞进入组织；蛋白酶、氧化物及细胞因子还可削弱尿路上皮紧密连接和表面屏障，使尿液中的刺激性物质更容易接触黏膜下神经和免疫细胞。受损上皮释放 ATP 和危险信号后，又会重新刺激肥大细胞和巨噬细胞。

炎症是持续还是消退，很大程度取决于巨噬细胞是否从 TNF/IL-1 主导的炎性状态转向凋亡细胞清除、IL-10/TGF-β和上皮修复程序。修复若长期过度也可能导致纤维化。不同慢性膀胱炎综合征证据异质性较大，因此不能用单一机制解释所有患者。

Q13

问题

在溃疡性结肠炎中，中性粒细胞募集、中性粒细胞胞外诱捕网形成、中性粒细胞代谢以及中性粒细胞-巨噬细胞串扰如何分别促进黏膜损伤或炎症消退？

回答

溃疡性结肠炎活动期最典型的组织学特征之一就是中性粒细胞大量募集。上皮和髓系细胞产生 CXCL8 相关趋化因子，配合补体和黏附分子，使中性粒细胞进入隐窝并形成隐窝脓肿。中性粒细胞可限制细菌移位，但其 ROS、弹性蛋白酶、髓过氧化物酶产物和其他蛋白酶在反应过强或持续时会直接损伤上皮。

NET 具有双重作用。胞外 DNA 和抗菌蛋白可以捕获微生物，但 NET 中的组蛋白、蛋白酶和持续存在的 DNA 也会损伤上皮并激活先天免疫。中性粒细胞高度依赖糖酵解，因此能在缺氧黏膜中继续发挥功能；但持续代谢应激也可能使其更长时间保持炎性状态。

巨噬细胞决定后期走向。早期 TNF、IL-1 和趋化因子继续驱动中性粒细胞进入；后期若巨噬细胞能高效清除凋亡中性粒细胞，就会增加 IL-10、TGF-β、促消退脂质介质并促进上皮修复。若清除失败或微生物刺激持续，炎症回路就不会关闭。因此治疗上更理想的是抑制过度募集或 NET 相关毒性，而不是完全清除中性粒细胞。

Q14

问题

在克罗恩病中，中性粒细胞、单核细胞来源巨噬细胞和树突状细胞之间失调的相互作用如何改变微生物处理、抗原呈递、炎症持续以及上皮修复？

回答

克罗恩病同时包含“微生物处理不足”和“炎症反应过度”两方面问题。屏障破损后，中性粒细胞会被募集，但不同患者早期反应并不一致：部分患者可能存在先天免疫反应延迟或效率下降，而已经形成的病灶中又可见高度活化的中性粒细胞释放 ROS 和蛋白酶。NOD2、自噬相关通路和上皮防御缺陷都可能影响细菌感知与清除。

募集的单核细胞可分化为炎性巨噬细胞，产生 TNF、IL-1、IL-6 和 IL-23。若细菌成分持续存在，这些巨噬细胞就难以转入凋亡细胞清除和修复。树突状细胞处理腔内和组织抗原，并通过 IL-12/IL-23 相关通路加强 Th1/Th17 反应。由此形成微生物刺激、巨噬细胞因子、T 细胞反应和中性粒细胞募集相互维持的环路。

修复同样受损。过量 TNF、ROS、蛋白酶和基质重塑酶延迟上皮复原，长期巨噬细胞—基质细胞激活还可推动纤维化。另一方面，IL-10、TGF-β、生长因子和中性粒细胞清除又是正常愈合所必需。因此克罗恩病并非某一种髓系细胞“太多”，而是多细胞协同失调。

Q15

问题

在重度中性粒细胞性哮喘中，中性粒细胞、巨噬细胞和树突状细胞如何形成自我维持的炎症回路？中性粒细胞胞外诱捕网、炎症小体信号、微生物信号以及糖皮质激素抵抗可能分别发挥什么作用？

回答

重度中性粒细胞性哮喘可形成自我维持的炎症回路。微生物信号、上皮损伤、IL-1 和 TNF 使上皮及基质细胞产生 CXCL8 和 G-CSF，持续招募并延长中性粒细胞存活。巨噬细胞可通过 IL-1β等炎症小体相关因子进一步放大反应；树突状细胞则可促进 IL-17 相关 T 细胞反应。IL-17 又会刺激更多中性粒细胞趋化因子，形成闭环。

NET 可能是其中的放大器。胞外 DNA、组蛋白、中性粒细胞弹性蛋白酶和髓过氧化物酶产物可损伤气道上皮并激活先天免疫感受器。NLRP3 炎症小体在机制上很合理，也有实验支持，但并非所有患者都同样依赖这一通路。慢性细菌定植或气道菌群失衡可不断提供 TLR 刺激，使回路难以关闭。

糖皮质激素抵抗通常是多因素的，包括 IL-17 主导炎症、氧化应激、p38/MAPK 异常、糖皮质激素受体功能下降以及 HDAC2 活性降低。糖皮质激素还可能延缓中性粒细胞凋亡。临床上目前更确定的是“非 2 型、先天免疫持续激活”的异质性表型，而 NET 或炎症小体靶向仍更多处于转化研究阶段。

Q16

问题

在嗜酸性粒细胞性哮喘中，嗜酸性粒细胞、肥大细胞以及在相关情境下的嗜碱性粒细胞，如何与巨噬细胞、树突状细胞和气道上皮相互作用，从而启动、放大并最终促使 2 型炎症消退？

回答

嗜酸性粒细胞性哮喘通常从气道上皮对过敏原、病毒或污染物的感知开始。上皮释放 TSLP、IL-33、IL-25，激活树突状细胞和 2 型先天免疫通路。树突状细胞诱导 Th2 反应，ILC2 也产生 IL-5 和 IL-13。IL-5 促进嗜酸性粒细胞生成和存活，eotaxin 使其进入气道。肥大细胞经特异性 IgE 激活后释放组胺、白三烯、PGD2 和细胞因子；部分情境下嗜碱性粒细胞可提供 IL-4。

放大阶段是多细胞共同完成的。嗜酸性粒细胞颗粒蛋白、脂质介质和细胞因子损伤上皮并增加气道高反应性；IL-4/IL-13 作用于巨噬细胞和上皮，促进黏液、趋化因子以及修复/重塑程序。巨噬细胞清除碎片，但长期 2 型信号也可能促进生长因子和纤维化。树突状细胞维持抗原呈递和记忆性 Th2 反应。

炎症消退需要去除触发因素、嗜酸性粒细胞凋亡或离开组织、巨噬细胞凋亡细胞清除、上皮屏障恢复以及调节性细胞因子和促消退脂质介质增加。临床上即使嗜酸性粒细胞下降，若上皮警报素、肥大细胞激活或结构性重塑仍在，症状仍可持续。

Q17

问题

在复发性尿路感染中，既往感染如何通过训练性免疫、免疫耐受或组织重塑改变膀胱黏膜中的中性粒细胞和单核细胞/巨噬细胞反应？目前对这些机制各自的证据强度如何？

回答

既往尿路感染可以通过训练性免疫、免疫耐受和组织重塑改变后续髓系反应，但三者证据强度并不相同。训练性免疫是指先天免疫细胞或其祖细胞发生表观遗传和代谢改变，再次遇到刺激时反应更快、更强。作为一般免疫学现象，其证据很充分，动物感染模型也支持长期先天免疫重编程；但"膀胱局部髓系训练是人类复发性 UTI 的主要机制"目前直接证据仍有限。

免疫耐受则相反。反复 TLR 或炎症刺激可降低后续 TNF、IL-1 等反应，甚至削弱趋化和杀菌能力。内毒素耐受的机制本身较明确，但其在一般复发性 UTI 患者中的临床重要性尚未确定，而且同一患者不同细胞亚群可能同时存在训练和耐受特征。

组织重塑与膀胱更直接相关。既往感染可改变尿路上皮分化、屏障性质、细胞外基质、感觉神经以及局部免疫细胞生态位。实验研究提示感染史可留下长期的上皮或基质"记忆"，既可能让再次感染时防御更快，也可能形成细菌持续存在或炎症过强的环境。总体而言，组织/上皮重塑的 UTI 模型证据更直接；髓系训练和耐受还需要更好的纵向人体研究。

Q18

问题

在膀胱癌中，肿瘤相关中性粒细胞和肿瘤相关巨噬细胞如何在黏膜肿瘤微环境中相互作用，并影响抗原呈递、T 细胞活性、血管生成、肿瘤侵袭以及免疫治疗反应？

回答

膀胱癌中的肿瘤相关巨噬细胞和中性粒细胞具有明显可塑性，既可抑癌也可促癌。肿瘤细胞和基质可分泌 CSF-1、CCL2、CXCL8、G-CSF 和补体相关信号，募集单核细胞和中性粒细胞。在许多进展期肿瘤中，巨噬细胞偏向 IL-10、TGF-β、精氨酸酶、PD-L1、VEGF 和基质重塑酶占优势的免疫抑制状态；中性粒细胞则可提供 ROS、精氨酸酶、MMP-9、VEGF 和 NET 相关信号。

二者可通过营养物质消耗、抑制性配体、细胞因子和肿瘤基质重塑削弱 T 细胞功能，并使有效抗原呈递下降。VEGF 和蛋白酶促进血管生成，MMP 和 NET 相关因子还可支持肿瘤侵袭。但这种作用并非固定不变：在适当刺激下，巨噬细胞可以恢复抗原呈递和杀伤功能，中性粒细胞也可能具有抗肿瘤作用。

免疫治疗是否有效，很大程度取决于这种髓系平衡。强烈的免疫抑制性髓系微环境会限制 T 细胞进入或功能，导致检查点抑制剂耐药。另一方面，膀胱灌注 BCG 能明显招募和激活中性粒细胞、单核/巨噬细胞和树突状细胞，说明局部先天免疫激活也可产生抗肿瘤效果。因此更合理的策略是重编程，而不是简单耗竭。

Q19

问题

在溃疡性结肠炎中，哪些具有机制学合理性的策略可能抑制致病性中性粒细胞募集或中性粒细胞胞外诱捕网相关损伤，同时尽量保留抗微生物防御和巨噬细胞介导的修复？请说明预期获益、潜在权衡及证据缺口。

回答

溃疡性结肠炎中更合理的思路是降低最具组织毒性的中性粒细胞反应，同时保留基本抗菌功能。第一类策略是部分阻断 CXCL8/CXCR1-CXCR2 募集轴，可减少隐窝内过度聚集和蛋白酶负荷。但抑制过强会影响细菌限制并增加感染风险，因此局部给药或短疗程理论上比长期全身阻断更安全。

第二类策略是处理 NET 相关损伤。DNase 可拆解胞外 DNA 骨架；中性粒细胞弹性蛋白酶或髓过氧化物酶抑制剂可减少 NET 毒性成分；PAD4 抑制可减少 NET 形成，但人体疗效和感染安全性尚不确定。一个重要权衡是，分解 DNA 并不等于同时中和组蛋白和蛋白酶，而且 NET 在某些感染情境下确有捕获微生物作用。

第三类思路不是"少招募"，而是"更快消退"。增强巨噬细胞凋亡细胞清除、MerTK 相关通路、IL-10 或促消退脂质介质，有望保留早期杀菌爆发，同时缩短中性粒细胞停留时间。当前最大的证据缺口是选择性不足。未来研究应同时观察感染率、黏膜愈合、NET 指标和巨噬细胞修复功能，而不能只看中性粒细胞数量。

Q20

问题

如何利用肠道巨噬细胞和粒细胞的代谢或表观遗传重编程来恢复炎症性肠病中的黏膜稳态？哪些机制可能导致疗效丧失或非预期的免疫抑制？

回答

炎症性肠病中利用代谢或表观遗传重编程的逻辑，是髓系细胞功能高度依赖其代谢状态。炎性巨噬细胞常表现为糖酵解增强、HIF-1 相关通路、琥珀酸信号和炎症小体活化；修复型巨噬细胞则更多依赖线粒体氧化代谢、脂质处理以及 AMPK、PPAR 等调节通路。中性粒细胞本身高度依赖糖酵解，其代谢状态也会影响存活、ROS 和 NET 形成。

潜在策略包括增强 AMPK/PPAR、利用 itaconate 样抗炎通路、调节 mTOR、改变短链脂肪酸信号，或靶向 BET 蛋白和特定组蛋白修饰酶。理论上可降低炎性细胞因子和 NET，同时改善巨噬细胞凋亡细胞清除和上皮修复。但这些靶点通常并不局限于肠道髓系细胞。

疗效丧失可能来自炎症通路冗余以及不断有新的循环单核细胞进入组织；微生物组、缺氧和局部细胞因子还会把新细胞重新推回炎性状态。过度抑制糖酵解、炎症小体或训练性免疫也可能削弱细菌和真菌防御。因此真正需要的是局部、细胞选择性、可逆的重编程，而不是长期全身免疫压制。现阶段多数方案仍属临床前或基于人组织研究的机制推断。

Q21

问题

能够改变 2 型细胞因子信号的生物制剂，除降低嗜酸性粒细胞数量外，还可能如何重塑气道黏膜中的嗜酸性粒细胞-肥大细胞-巨噬细胞-树突状细胞网络？

回答

改变 2 型细胞因子信号的生物制剂会重塑整个气道髓系网络，而不仅仅是降低嗜酸性粒细胞计数。阻断 IL-5 或 IL-5 受体可减少嗜酸性粒细胞成熟、存活和组织滞留，继而降低白三烯、颗粒蛋白和相关细胞因子，减少上皮损伤，也减少对巨噬细胞和树突状细胞的继发刺激。

阻断 IL-4/IL-13 的影响更广。巨噬细胞接受的 IL-4Rα信号下降后，部分替代性激活、黏液相关修复和重塑程序会减弱；气道上皮产生的 2 型趋化因子减少，树突状细胞—Th2 反馈也会减弱。抗 IgE 治疗降低肥大细胞和嗜碱性粒细胞 FcεRI 相关激活，因此减少即时介质释放和后续髓系募集。上游 TSLP 阻断则可同时削弱树突状细胞激活及多个下游 2 型通路。

这也解释了为什么临床获益有时明显大于血嗜酸性粒细胞的变化。反过来，患者即使嗜酸性粒细胞明显下降，若肥大细胞持续激活、气道结构重塑或非 2 型炎症仍在，症状仍可存在。因此更准确的理解是“细胞网络重新接线”，而不是单纯“清除嗜酸性粒细胞”。

Q22

问题

在中性粒细胞性哮喘中，哪些针对髓系细胞的策略可能中断中性粒细胞-巨噬细胞炎症回路，同时保留抗细菌防御？这些策略所依据的机制中，哪些已有临床证据，哪些仍主要处于临床前阶段？

回答

中性粒细胞性哮喘的理想靶向方案，应降低病理性募集和激活，同时保留足够的抗菌能力。CXCR2 拮抗是最直接的思路之一，可减少中性粒细胞趋化，临床研究也证明气道中性粒细胞可以下降，但症状和结局改善并不稳定，而且存在感染或系统性中性粒细胞减少的顾虑，因此并未成为标准治疗。

其他方案成熟度更低。阻断 IL-1 或 NLRP3 炎症小体可能减少巨噬细胞对中性粒细胞的放大；抑制 GM-CSF 或部分 TLR 通路可能缩短中性粒细胞存活。NET 方向包括 DNase、PAD4 抑制和中性粒细胞弹性蛋白酶抑制；补体/C5a 以及代谢靶点也具有机制合理性。多数在哮喘中仍属于临床前或早期转化阶段，广泛抑制都会带来抗细菌防御下降的风险。

目前临床上有较明确证据的是，大环内酯类可使部分重度非 2 型/中性粒细胞性哮喘患者获益，但其机制很复杂，包含抗菌、改变气道菌群和免疫调节，并不是选择性的“中性粒细胞—巨噬细胞回路阻断剂”。因此现阶段尚无类似 2 型生物制剂那样成熟的髓系特异治疗。

Q23

问题

训练性免疫或黏膜疫苗策略是否可能通过有益地重编程单核细胞/巨噬细胞和中性粒细胞来减少复发性尿路感染？请解释其拟议机制、潜在的组织特异性风险以及当前证据的局限。

回答

训练性免疫和黏膜疫苗理论上都可能通过提高膀胱黏膜的早期免疫质量来减少复发性尿路感染。黏膜疫苗可以产生病原特异性 IgA/IgG 和 T 细胞记忆，也可能通过局部细胞因子和基质信号间接改变巨噬细胞与中性粒细胞。训练性免疫则可能让单核/巨噬细胞或骨髓祖细胞发生表观遗传、代谢重编程，再次暴露时更快产生细胞因子并更有效招募中性粒细胞。

主要风险是组织特异性不足。若“训练”过强，可能导致更剧烈的中性粒细胞性膀胱炎、上皮损伤、疼痛甚至纤维化；反复刺激也可能走向免疫耐受，反而削弱有用的细胞因子反应。若是全身性训练，还可能增加尿路之外的炎症反应。因此理想方案应形成受控的局部免疫记忆，提高早期清菌，但不延长炎症。

目前证据有希望但并不完整。一些疫苗或细菌提取物策略在特定人群中显示复发率下降，但产品、给药途径、终点和研究质量差异较大。多数研究也无法区分获益究竟来自抗体/T 细胞、髓系训练、非特异佐剂效应还是微生物组变化。直接证明人膀胱单核/巨噬细胞或中性粒细胞祖细胞发生持久表观遗传重编程的证据仍有限。

Q24

问题

如何通过选择性重编程肿瘤相关巨噬细胞和中性粒细胞来增强膀胱癌的抗肿瘤免疫，同时尽量减少对保护性尿路黏膜免疫的破坏？请比较不同机制策略及其主要转化障碍。

回答

膀胱癌中，与其广泛耗竭肿瘤相关巨噬细胞和中性粒细胞，更合理的是选择性重编程，因为这些细胞同时承担尿路抗感染防御。巨噬细胞方面，可考虑抑制 CSF-1/CSF-1R 或 CCL2 募集，阻断 PI3Kγ、TREM2 样免疫抑制程序，或通过 CD47-SIRPα、TLR、STING 等增强吞噬和抗原呈递，目标是把 IL-10/TGF-β/VEGF 占优势的状态转向更支持 T 细胞和肿瘤杀伤的状态。

中性粒细胞方面，CXCL8/CXCR2 或 C5a 轴可用于减少免疫抑制性肿瘤相关中性粒细胞募集；NET、弹性蛋白酶和代谢靶向可能减少侵袭和 T 细胞抑制，同时尽量保留基本吞噬能力。膀胱灌注具有特殊优势，因为局部给药有望重塑肿瘤微环境，而不明显影响全身中性粒细胞和巨噬细胞。BCG 已经提供了“局部先天免疫可用于抗肿瘤”的临床概念验证。

主要转化障碍包括髓系细胞异质性和快速可塑性、通路间代偿、感染风险、缺乏可靠生物标志物、药物进入肿瘤深部的限制，以及与检查点抑制剂或化疗如何排序。未来更可能依赖空间组学和功能标志物，精准识别需要重编程的抑制性髓系亚群。

Traditional Chinese (TC)

Model B | 繁體中文 | 24 Questions

模型 B

髓系－黏膜免疫 / Q01–Q24

Q01

問題

在健康腸黏膜中，中性粒細胞如何在與駐留巨噬細胞和樹突狀細胞相互作用的同時參與屏障防禦？哪些機制通常限制中性粒細胞介導的組織損傷？

回答

健康腸黏膜內的中性粒細胞數量通常很低，真正發生上皮屏障破損或微生物越界時才會迅速募集。上皮細胞、駐留巨噬細胞以及基質細胞可產生 CXCL1、CXCL2、CXCL8 等趨化訊號。募集來的中性粒細胞透過吞噬、氧爆發、抗菌顆粒蛋白以及在高病原負荷時形成胞外誘捕網來控制細菌。駐留巨噬細胞既參與早期感知和募集，也負責後期清除衰老或凋亡的中性粒細胞；樹突狀細胞則更偏向抗原採樣和後續適應性免疫。

健康狀態的關鍵不是“沒有中性粒細胞”，而是反應被限制在合適的時間和空間。完整黏液層、緊密連接、抗菌肽和快速上皮修復減少持續募集的需要。腸道巨噬細胞對無害腔內刺激相對低反應，並可產生 IL-10 等調節訊號。中性粒細胞完成任務後進入凋亡，巨噬細胞透過凋亡細胞清除轉向 IL-10、TGF-β、促消退脂質介質和組織修復程序。

此外，抗蛋白酶、抗氧化系統、清除胞外 DNA 的 DNase 以及趨化梯度的終止都可限制損傷。若這些“剎車”失效，中性粒細胞用於殺菌的蛋白酶、氧化物和 NET 相關組蛋白也會損傷隱窩上皮並推動慢性發炎。

Q02

問題

在健康腸黏膜中，嗜酸性粒細胞如何參與維持穩態並與巨噬細胞或樹突狀細胞進行通訊？這些作用與發炎狀態下嗜酸性粒細胞的功能有何不同？

回答

嗜酸性粒細胞本身就是正常腸固有層的一部分，在小腸尤其明顯。穩態下，它們主要受 IL-5 和 eotaxin/CCR3 軸維持，可參與屏障監視、免疫球蛋白相關反應和組織重塑。與發炎時劇烈脫顆粒不同，穩態嗜酸性粒細胞可較溫和地釋放細胞激素、脂質介質和生長因子。其 IL-4、IL-13 等訊號可使部分巨噬細胞偏向修復型功能，但這種作用在人和不同腸段中並不完全一致。對樹突狀細胞的影響可能涉及抗原呈遞和局部耐受，但人體直接證據相對有限。

進入發炎狀態後，嗜酸性粒細胞募集增多、存活延長，並釋放主要鹼性蛋白、嗜酸性粒細胞過氧化物酶、陽離子蛋白、白三烯以及促發炎細胞激素。這些介質可損傷上皮、增加血管通透性、刺激感覺神經，並改變巨噬細胞和樹突狀細胞的活化狀態。同時，它們仍可能透過 TGF-β和生長因子參與修復。

因此，臨床上不宜把腸道嗜酸性粒細胞簡單視為“有害細胞”。生理狀態下可能有穩態作用，過度或持續活化時則可把同一網路推向發炎和纖維化。很多精細的細胞間機制在動物模型中證據更強，在人腸道中仍具有明顯的情境依賴性。

Q03

問題

在健康呼吸道黏膜以及急性微生物攻擊期間，中性粒細胞如何與肺泡或間質巨噬細胞及樹突狀細胞協同，在發揮宿主防禦作用的同時限制對上皮的附帶損傷？

回答

健康肺內，肺泡巨噬細胞會清除吸入顆粒並抑制不必要的發炎，從而使中性粒細胞募集保持在低水平。急性細菌或真菌刺激時，肺泡和間質巨噬細胞透過模式識別受體感知病原，釋放 TNF、IL-1、CXCL1/CXCL2/CXCL8 等介質，迅速募集中性粒細胞。樹突狀細胞負責抗原採樣並塑造後續 T 細胞反應，也可透過細胞激素增強局部抗感染反應。

中性粒細胞依靠吞噬、氧爆發、抗菌蛋白酶和在部分情境下形成 NET 來快速殺菌。早期巨噬細胞與中性粒細胞之間可互相放大：巨噬細胞趨化因子募集中性粒細胞，而中性粒細胞釋放的危險訊號又能進一步活化巨噬細胞。這在高病原負荷時有利，但持續過久會轉為組織損傷。

發炎消退需要主動“剎車”。中性粒細胞凋亡後被巨噬細胞清除，可促進 IL-10、TGF-β、脂氧素/消退素等促消退介質和上皮修復。氣道抗氧化系統、抗蛋白酶、黏液清除和 DNase 可限制氧化物、彈性蛋白酶和 NET 相關損傷。臨床上，關鍵是“及時募集、及時清除”，而不是單純追求更多中性粒細胞。

Q04

問題

在穩態條件和第 2 型發炎期間，嗜酸性粒細胞如何在氣道黏膜中與巨噬細胞和樹突狀細胞相互作用？

回答

穩態氣道中的嗜酸性粒細胞數量較少，肺泡巨噬細胞和樹突狀細胞總體維持低發炎環境，上皮屏障也減少吸入抗原與免疫細胞的直接接觸。此時嗜酸性粒細胞可提供少量細胞激素和生長因子，但其穩態作用通常不像腸道那樣突出。

2 型發炎時，上皮細胞釋放 IL-33、TSLP、IL-25 等警報素，活化樹突狀細胞和 2 型先天淋巴樣細胞。樹突狀細胞促進 Th2 分化，形成 IL-4、IL-5 和 IL-13 環境；IL-5 延長嗜酸性粒細胞存活，eotaxin 透過 CCR3 促進其募集。活化嗜酸性粒細胞釋放白三烯、顆粒蛋白、細胞激素和生長因子，可進一步影響樹突狀細胞，並使巨噬細胞偏向 IL-4/IL-13 反應性修復程序。

這類互作既有保護性也有致病性。巨噬細胞的修復程序有助於清除碎片和恢復上皮，但持續的 2 型訊號也可促進黏液分泌、氣道高反應性和重塑；嗜酸性粒細胞顆粒蛋白還能直接損傷上皮。因此，急性反應可在同一網路中被終止，而反復刺激時則可能演變為慢性氣喘。

Q05

問題

在尿路黏膜中，早期抗菌防禦期間，中性粒細胞如何與單核細胞或巨噬細胞及樹突狀細胞協同？哪些機制可防止過度組織損傷？

回答

急性細菌性尿路感染早期，尿路上皮和駐留髓系細胞透過 TLR 等模式識別通路感知細菌，迅速產生 IL-6、CXCL8 等趨化訊號，形成明顯的中性粒細胞募集。中性粒細胞穿過尿路上皮進入尿腔，在細菌負荷最高的位置進行吞噬、氧爆發、釋放抗菌蛋白，並在部分情況下形成胞外誘捕網。

單核細胞和巨噬細胞透過釋放趨化因子支持早期募集，同時吞噬細菌和組織碎片，後期還負責清除凋亡中性粒細胞。樹突狀細胞參與抗原呈遞並把先天免疫與適應性免疫連接起來。若巨噬細胞或中性粒細胞產生過多 TNF、IL-1 等發炎介質，可能只增加組織發炎而不再改善細菌清除。

限制組織損傷的核心是"快速殺菌後快速關閉"。中性粒細胞凋亡、巨噬細胞凋亡細胞清除、IL-10、TGF-β、促消退脂質介質以及尿路上皮再生共同幫助恢復屏障。若中性粒細胞持續存在，其蛋白酶、氧化物和 NET 成分可損傷尿路上皮並加重尿頻、尿痛等症狀。

Q06

問題

在膀胱或下泌尿生殖道黏膜中，肥大細胞釋放的介質如何影響巨噬細胞和樹突狀細胞的功能？這一網路如何同時參與宿主防禦和發炎相關症狀？

回答

肥大細胞位於膀胱和下泌尿生殖道的血管、神經和上皮附近，因此其介質能很快影響巨噬細胞和樹突狀細胞。組胺、TNF、胰蛋白酶樣蛋白酶、前列腺素、白三烯、IL-6 等可增加血管通透性，並促進髓系細胞募集和活化。巨噬細胞、樹突狀細胞上的組胺受體和蛋白酶活化受體還能改變其細胞激素產生、抗原呈遞和組織重塑功能。

感染或組織損傷時，這種反應有利於提高局部血流和白細胞進入，巨噬細胞進一步完成吞噬、細胞激素產生、凋亡細胞清除和修復，樹突狀細胞則推動適應性免疫。但同一網路也會產生症狀。組胺、胰蛋白酶樣蛋白酶、前列腺素、ATP 相關訊號和神經營養因子可使盆腔感覺神經敏化，出現尿急、尿頻、疼痛或燒灼感；血管滲漏還會形成水腫並進一步放大發炎。

若刺激持續，肥大細胞和巨噬細胞之間可形成正反饋，削弱上皮屏障並維持神經發炎。相反，IL-10、TGF-β及修復型巨噬細胞可抑制這一迴路。不同膀胱炎綜合徵的人體證據並不完全一致，因此更適合把它理解為神經—免疫—血管共同參與的機制框架。

Q07

問題

在急性腸黏膜發炎及其後的發炎消退階段，中性粒細胞與巨噬細胞如何透過募集訊號、細胞激素、凋亡細胞清除、脂質介質和組織修復程序相互影響？

回答

急性腸黏膜發炎時，巨噬細胞和上皮細胞透過 CXCL 趨化因子、IL-1、TNF、補體片段和脂質介質募集中性粒細胞。中性粒細胞殺菌的同時會釋放 ROS、蛋白酶、促發炎細胞激素和損傷相關訊號，進一步活化巨噬細胞。CCL2 等訊號還可募集單核細胞並分化為炎性巨噬細胞；如果病原或損傷沒有被控制，就會形成正反饋。

發炎消退不是"自然停止"，而是主動程序。中性粒細胞進入凋亡後，巨噬細胞對其進行凋亡細胞清除，這是關鍵轉折點。清除後，巨噬細胞降低促發炎細胞激素，增加 IL-10、TGF-β和修復相關程序。同時發生脂質介質"類別轉換"：早期白三烯、前列腺素為主，隨後更多出現脂氧素、消退素、保護素和 maresin，減少進一步募集並促進清除。

修復型巨噬細胞促進上皮復原、血管新生、基質重塑和生長因子產生。但這些狀態並不是簡單的 M1/M2 二分，而是受微生物負荷、缺氧和局部細胞激素共同決定。凋亡細胞清除失敗或微生物刺激持續，就會把本來短暫的中性粒細胞反應轉成慢性黏膜損傷。

Q08

問題

嗜酸性粒細胞來源的介質如何改變腸黏膜巨噬細胞的功能狀態和樹突狀細胞的活化？這些相互作用如何推動免疫耐受、組織修復與發炎之間的平衡發生變化？

回答

嗜酸性粒細胞可透過細胞激素、脂質介質、顆粒蛋白和生長因子改變腸黏膜中的巨噬細胞和樹突狀細胞。IL-4、IL-13 可使部分巨噬細胞偏向傷口修復、細胞外基質處理和較低的經典發炎反應；嗜酸性粒細胞還可能透過 TGF-β、調節性脂質介質等促進組織恢復。另一方面，嗜酸性粒細胞過氧化物酶、陽離子顆粒蛋白、白三烯和危險訊號也可增強巨噬細胞發炎並造成組織損傷。

對樹突狀細胞的影響同樣具有雙向性。相對溫和的嗜酸性粒細胞環境可能有利於耐受性抗原呈遞，維持對食物或共生菌抗原的低反應；活動性發炎時，嗜酸性粒細胞介質可促進樹突狀細胞成熟、遷移或細胞激素釋放，從而增強 T 細胞活化。實驗中雖有嗜酸性粒細胞參與抗原呈遞的報道，但在人腸道中不太可能是主導途徑。

因此，最終結果取決於刺激強度和持續時間。短暫活化可參與修復，長期募集和脫顆粒則更容易把耐受—修復平衡推向發炎。人類直接證據仍不如動物和離體研究充分。

Q09

問題

在中性粒細胞性氣道發炎中，中性粒細胞與巨噬細胞如何透過細胞激素、脂質介質、中性粒細胞胞外誘捕網、凋亡細胞清除和代謝訊號相互影響？這種串擾如何決定發炎持續還是消退？

回答

中性粒細胞性氣道發炎中，巨噬細胞可釋放 TNF、IL-1、CXCL8、GM-CSF 以及脂質介質，促進中性粒細胞募集並延長其存活。中性粒細胞反過來釋放蛋白酶、ROS、警報素、LTB4 和 NET，進一步活化巨噬細胞。NET 中的 DNA 和組蛋白具有抗菌作用，但持續存在時可損傷上皮，並刺激發炎小體或其他先天免疫通路。

凋亡細胞清除是決定走向的重要節點。巨噬細胞透過 MerTK 等相關受體高效吞噬凋亡中性粒細胞後，會降低促發炎細胞激素，並增加 IL-10、TGF-β、促消退脂質介質和組織修復。若清除受損，中性粒細胞可發生繼發壞死，蛋白酶和胞內成分進入氣道，反而延長發炎。

代謝也參與調節。缺氧、乳酸、琥珀酸、線粒體應激和 HIF-1 相關糖酵解程序可維持炎性巨噬細胞和中性粒細胞狀態；而線粒體代謝和脂質處理恢復則更有利於巨噬細胞進入修復。換句話說，持續取決於"繼續募集+NET+代謝應激+清除失敗"的閉環，而消退依賴病原下降、趨化減弱、NET 清除和巨噬細胞向修復轉換。

Q10

問題

在過敏性氣道發炎中，肥大細胞和嗜酸性粒細胞（在相關情境下包括嗜鹼性粒細胞）如何與樹突狀細胞和巨噬細胞相互作用，從而放大或限制第 2 型免疫反應？

回答

過敏性氣道發炎中，肥大細胞、嗜酸性粒細胞以及部分情境下的嗜鹼性粒細胞，與樹突狀細胞和巨噬細胞共同放大 2 型免疫。過敏原特異性 IgE 交聯肥大細胞 FcεRI 後，可釋放組胺、胰蛋白酶樣蛋白酶、PGD2、白三烯、TNF 和 2 型細胞激素，引起血管通透性增加、支氣管收縮，並促進嗜酸性粒細胞和樹突狀細胞進入。嗜鹼性粒細胞在某些情況下可提供早期 IL-4，但其重要性因疾病階段而異。

樹突狀細胞攝取過敏原並誘導 Th2 反應，產生 IL-4、IL-5、IL-13。IL-5 維持嗜酸性粒細胞，IL-4/IL-13 則影響巨噬細胞、黏液分泌和組織重塑。嗜酸性粒細胞釋放顆粒蛋白和脂質介質，既可損傷上皮，也可進一步增強抗原呈遞細胞的活化。巨噬細胞既可能透過發炎介質放大反應，也可能清除碎片並參與屏障修復。

限制機制包括 IL-10、TGF-β、抑制性受體訊號、凋亡細胞清除和促消退脂質介質。慢性氣喘常反映這些終止機制不足，使肥大細胞活化、嗜酸性粒細胞存活、持續抗原呈遞和氣道重塑彼此維持。

Q11

問題

在急性細菌性尿路感染期間，募集到局部的中性粒細胞和單核細胞來源巨噬細胞如何在病原體清除、細胞激素產生、凋亡細胞清除和黏膜修復過程中協同或競爭？

回答

急性細菌性尿路感染中，中性粒細胞是最快、殺菌能力最強的一線效應細胞。它們受到尿路上皮和髓系細胞趨化因子募集，穿過膀胱上皮後進入尿腔，透過吞噬、氧化物、顆粒酶和胞外誘捕機制清除細菌。單核細胞來源巨噬細胞通常稍後增加，功能更"綜合"：既吞噬細菌，也清除組織碎片和凋亡中性粒細胞，並參與修復。

二者協同時，巨噬細胞透過趨化訊號幫助中性粒細胞到位，後期再透過凋亡細胞清除結束反應。它們也可能發生功能競爭。巨噬細胞過多產生 TNF、IL-1 會繼續拉高中性粒細胞募集；而中性粒細胞過度脫顆粒可破壞尿路上皮並釋放 DAMP，使巨噬細胞持續活化。如果細菌沒有真正清除，就容易形成"發炎更多、殺菌改善不明顯"的狀態。

修復階段需要細菌負荷下降、中性粒細胞凋亡、巨噬細胞高效清除以及趨化因子減少。隨後巨噬細胞增加 IL-10、TGF-β、生長因子和基質重塑，促進上皮復原。因此更合適的理解是"先由中性粒細胞快速殺菌，後由巨噬細胞清場和修復"，而不是簡單比較誰更重要。

Q12

問題

在慢性膀胱黏膜發炎中，肥大細胞與巨噬細胞之間的串擾可能如何改變感覺神經訊號、血管通透性、上皮屏障功能以及發炎的持續或消退？

回答

慢性膀胱發炎中，肥大細胞—巨噬細胞串擾可把免疫反應直接連接到疼痛、血管滲漏和屏障損傷。肥大細胞釋放的組胺、胰蛋白酶樣蛋白酶、前列腺素、白三烯、TNF 和神經生長因子可活化或敏化感覺神經；蛋白酶活化受體和組胺受體訊號可降低疼痛和尿急閾值。巨噬細胞來源 IL-1、TNF、IL-6 和前列腺素也會加強這一神經敏化。

這些介質同時作用於血管和上皮。組胺和促發炎細胞激素提高內皮通透性，使血漿蛋白和更多白細胞進入組織；蛋白酶、氧化物及細胞激素還可削弱尿路上皮緊密連接和表面屏障，使尿液中的刺激性物質更容易接觸黏膜下神經和免疫細胞。受損上皮釋放 ATP 和危險訊號後，又會重新刺激肥大細胞和巨噬細胞。

發炎是持續還是消退，很大程度取決於巨噬細胞是否從 TNF/IL-1 主導的炎性狀態轉向凋亡細胞清除、IL-10/TGF-β和上皮修復程序。修復若長期過度也可能導致纖維化。不同慢性膀胱炎綜合徵證據異質性較大，因此不能用單一機制解釋所有患者。

Q13
問題
在潰瘍性結腸炎中，中性粒細胞募集、中性粒細胞胞外誘捕網形成、中性粒細胞代謝以及中性粒細胞-巨噬細胞串擾如何分別促進黏膜損傷或發炎消退？

回答

潰瘍性結腸炎活動期最典型的組織學特徵之一就是中性粒細胞大量募集。上皮和髓系細胞產生 CXCL8 相關趨化因子，配合補體和黏附分子，使中性粒細胞進入隱窩並形成隱窩膿腫。中性粒細胞可限制細菌移位，但其 ROS、彈性蛋白酶、髓過氧化物酶產物和其他蛋白酶在反應過強或持續時會直接損傷上皮。

NET 具有雙重作用。胞外 DNA 和抗菌蛋白可以捕獲微生物，但 NET 中的組蛋白、蛋白酶和持續存在的 DNA 也會損傷上皮並活化先天免疫。中性粒細胞高度依賴糖酵解，因此能在缺氧黏膜中繼續發揮功能；但持續代謝應激也可能使其更長時間保持炎性狀態。

巨噬細胞決定後期走向。早期 TNF、IL-1 和趨化因子繼續驅動中性粒細胞進入；後期若巨噬細胞能高效清除凋亡中性粒細胞，就會增加 IL-10、TGF-β、促消退脂質介質並促進上皮修復。若清除失敗或微生物刺激持續，發炎迴路就不會關閉。因此治療上更理想的是抑制過度募集或 NET 相關毒性，而不是完全清除中性粒細胞。

Q14
問題
在克隆氏症中，中性粒細胞、單核細胞來源巨噬細胞和樹突狀細胞之間失調的相互作用如何改變微生物處理、抗原呈遞、發炎持續以及上皮修復？

回答

克隆氏症同時包含“微生物處理不足”和“發炎反應過度”兩方面問題。屏障破損後，中性粒細胞會被募集，但不同患者早期反應並不一致：部分患者可能存在先天免疫反應延遲或效率下降，而已經形成的病灶中又可見高度活化的中性粒細胞釋放 ROS 和蛋白酶。NOD2、自噬相關通路和上皮防禦缺陷都可能影響細菌感知與清除。

募集的單核細胞可分化為炎性巨噬細胞，產生 TNF、IL-1、IL-6 和 IL-23。若細菌成分持續存在，這些巨噬細胞就難以轉入凋亡細胞清除和修復。樹突狀細胞處理腔內和組織抗原，並透過 IL-12/IL-23 相關通路加強 Th1/Th17 反應。由此形成微生物刺激、巨噬細胞激素、T 細胞反應和中性粒細胞募集相互維持的環路。

修復同樣受損。過量 TNF、ROS、蛋白酶和基質重塑酶延遲上皮復原，長期巨噬細胞—基質細胞活化還可推動纖維化。另一方面，IL-10、TGF-β、生長因子和中性粒細胞清除又是正常愈合所必需。因此克隆氏症並非某一種髓系細胞“太多”，而是多細胞協同失調。

Q15
問題
在重度中性粒細胞性氣喘中，中性粒細胞、巨噬細胞和樹突狀細胞如何形成自我維持的發炎迴路？中性粒細胞胞外誘捕網、發炎小體訊號、微生物訊號以及糖皮質激素抗性可能分別發揮什麼作用？

回答

重度中性粒細胞性氣喘可形成自我維持的發炎迴路。微生物訊號、上皮損傷、IL-1 和 TNF 使上皮及基質細胞產生 CXCL8 和 G-CSF，持續募集並延長中性粒細胞存活。巨噬細胞可透過 IL-1β等發炎小體相關因子進一步放大反應；樹突狀細胞則可促進 IL-17 相關 T 細胞反應。IL-17 又會刺激更多中性粒細胞趨化因子，形成閉環。

NET 可能是其中的放大器。胞外 DNA、組蛋白、中性粒細胞彈性蛋白酶和髓過氧化物酶產物可損傷氣道上皮並活化先天免疫感受器。NLRP3 發炎小體在機制上很合理，也有實驗支持，但並非所有患者都同樣依賴這一通路。慢性細菌定植或氣道菌群失衡可不斷提供 TLR 刺激，使迴路難以關閉。

糖皮質激素抵抗通常是多因素的，包括 IL-17 主導發炎、氧化應激、p38/MAPK 異常、糖皮質激素受體功能下降以及 HDAC2 活性降低。糖皮質激素還可能延緩中性粒細胞凋亡。臨床上目前更確定的是“非 2 型、先天免疫持續活化”的異質性表型，而 NET 或發炎小體靶向仍更多處於轉化研究階段。

Q16
問題
在嗜酸性粒細胞性氣喘中，嗜酸性粒細胞、肥大細胞以及在相關情境下的嗜鹼性粒細胞，如何與巨噬細胞、樹突狀細胞和氣道上皮相互作用，從而啟動、放大並最終促使第 2 型發炎消退？

回答

嗜酸性粒細胞性氣喘通常從氣道上皮對過敏原、病毒或污染物的感知開始。上皮釋放 TSLP、IL-33、IL-25，活化樹突狀細胞和 2 型先天免疫通路。樹突狀細胞誘導 Th2 反應，ILC2 也產生 IL-5 和 IL-13。IL-5 促進嗜酸性粒細胞生成和存活，eotaxin 使其進入氣道。肥大細胞經特異性 IgE 活化後釋放組胺、白三烯、PGD2 和細胞激素；部分情境下嗜鹼性粒細胞可提供 IL-4。

放大階段是多細胞共同完成的。嗜酸性粒細胞顆粒蛋白、脂質介質和細胞激素損傷上皮並增加氣道高反應性；IL-4/IL-13 作用於巨噬細胞和上皮，促進黏液、趨化因子以及修復/重塑程序。巨噬細胞清除碎片，但長期 2 型訊號也可能促進生長因子和纖維化。樹突狀細胞維持抗原呈遞和記憶性 Th2 反應。

發炎消退需要去除觸發因素、嗜酸性粒細胞凋亡或離開組織、巨噬細胞凋亡細胞清除、上皮屏障恢復以及調節性細胞激素和促消退脂質介質增加。臨床上即使嗜酸性粒細胞下降，若上皮警報素、肥大細胞活化或結構性重塑仍在，症狀仍可持續。

Q17

問題

在復發性尿路感染中，既往感染如何透過訓練性免疫、免疫耐受或組織重塑改變膀胱黏膜中的中性粒細胞和單核細胞/巨噬細胞反應？目前對這些機制各自的證據強度如何？

回答

既往尿路感染可以透過訓練性免疫、免疫耐受和組織重塑改變後續髓系反應，但三者證據強度並不相同。訓練性免疫是指先天免疫細胞或其祖細胞發生表觀遺傳和代謝改變，再次遇到刺激時反應更快、更強。作為一般免疫學現象，其證據很充分，動物感染模型也支持長期先天免疫重編程；但“膀胱局部髓系訓練是人類復發性 UTI 的主要機制”目前直接證據仍有限。

免疫耐受則相反。反復 TLR 或發炎刺激可降低後續 TNF、IL-1 等反應，甚至削弱趨化和殺菌能力。內毒素耐受的機制本身較明確，但其在一般復發性 UTI 患者中的臨床重要性尚未確定，而且同一患者不同細胞亞群可能同時存在訓練和耐受特徵。

組織重塑與膀胱更直接相關。既往感染可改變尿路上皮分化、屏障性質、細胞外基質、感覺神經以及局部免疫細胞生態位。實驗研究提示感染史可留下長期的上皮或基質“記憶”，既可能讓再次感染時防禦更快，也可能形成細菌持續存在或發炎過強的環境。總體而言，組織/上皮重塑的 UTI 模型證據更直接；髓系訓練和耐受還需要更好的縱向人體研究。

Q18

問題

在膀胱癌中，腫瘤相關中性粒細胞和腫瘤相關巨噬細胞如何在黏膜腫瘤微環境中相互作用，並影響抗原呈遞、T 細胞活性、血管新生、腫瘤侵襲以及免疫治療反應？

回答

膀胱癌中的腫瘤相關巨噬細胞和中性粒細胞具有明顯可塑性，既可抑癌也可促癌。腫瘤細胞和基質可分泌 CSF-1、CCL2、CXCL8、G-CSF 和補體相關訊號，募集單核細胞和中性粒細胞。在許多進展期腫瘤中，巨噬細胞偏向 IL-10、TGF-β、精氨酸酶、PD-L1、VEGF 和基質重塑酶佔優勢的免疫抑制狀態；中性粒細胞則可提供 ROS、精氨酸酶、MMP-9、VEGF 和 NET 相關訊號。

二者可透過營養物質消耗、抑制性配體、細胞激素和腫瘤基質重塑削弱 T 細胞功能，並使有效抗原呈遞下降。VEGF 和蛋白酶促進血管新生，MMP 和 NET 相關因子還可支持腫瘤侵襲。但這種作用並非固定不變：在適當刺激下，巨噬細胞可以恢復抗原呈遞和殺傷功能，中性粒細胞也可能具有抗腫瘤作用。

免疫治療是否有效，很大程度取決於這種髓系平衡。強烈的免疫抑制性髓系微環境會限制 T 細胞進入或功能，導致檢查點抑制劑耐藥。另一方面，膀胱灌注 BCG 能明顯募集和活化中性粒細胞、單核/巨噬細胞和樹突狀細胞，說明局部先天免疫活化也可產生抗腫瘤效果。因此更合理的策略是重編程，而不是簡單耗竭。

Q19

問題

在潰瘍性結腸炎中，哪些具有機制學合理性的策略可能抑制致病性中性粒細胞募集或中性粒細胞胞外誘捕網相關損傷，同時盡量保留抗微生物防禦和巨噬細胞介導的修復？請說明預期獲益、潛在權衡及證據缺口。

回答

潰瘍性結腸炎中更合理的思路是降低最具組織毒性的中性粒細胞反應，同時保留基本抗菌功能。第一類策略是部分阻斷 CXCL8/CXCR1-CXCR2 募集軸，可減少隱窩內過度聚集和蛋白酶負荷。但抑制過強會影響細菌限制並增加感染風險，因此局部給藥或短療程理論上比長期全身阻斷更安全。

第二類策略是處理 NET 相關損傷。DNase 可拆解胞外 DNA 骨架；中性粒細胞彈性蛋白酶或髓過氧化物酶抑制劑可減少 NET 毒性成分；PAD4 抑制可減少 NET 形成，但人體療效和感染安全性尚不確定。一個重要權衡是，分解 DNA 並不等於同時中和組蛋白和蛋白酶，而且 NET 在某些感染情境下確有捕獲微生物作用。

第三類思路不是“少募集”，而是“更快消退”。增強巨噬細胞凋亡細胞清除、MerTK 相關通路、IL-10 或促消退脂質介質，有望保留早期殺菌爆發，同時縮短中性粒細胞停留時間。當前最大的證據缺口是選擇性不足。未來研究應同時觀察感染率、黏膜愈合、NET 指標和巨噬細胞修復功能，而不能只看中性粒細胞數量。

Q20

問題

如何利用腸道巨噬細胞和粒細胞的代謝或表觀遺傳重編程來恢復發炎性腸病中的黏膜穩態？哪些機制可能導致療效喪失或非預期的免疫抑制？

回答

發炎性腸病中利用代謝或表觀遺傳重編程的邏輯，是髓系細胞功能高度依賴其代謝狀態。炎性巨噬細胞常表現為糖酵解增強、HIF-1 相關通路、琥珀酸訊號和發炎小體活化；修復型巨噬細胞則更多依賴線粒體氧化代謝、脂質處理以及 AMPK、PPAR 等調節通路。中性粒細胞本身高度依賴糖酵解，其代謝狀態也會影響存活、ROS 和 NET 形成。

潛在策略包括增強 AMPK/PPAR、利用 itaconate 樣抗炎通路、調節 mTOR、改變短鏈脂肪酸訊號，或靶向 BET 蛋白和特定組蛋白修飾酶。理論上可降低促發炎細胞激素和 NET，同時改善巨噬細胞凋亡細胞清除和上皮修復。但這些靶點通常並不局限於腸道髓系細胞。

療效喪失可能來自發炎通路冗余以及不斷有新的循環單核細胞進入組織；微生物組、缺氧和局部細胞激素還會把新細胞重新推回炎性狀態。過度抑制糖酵解、發炎小體或訓練性免疫也可能削弱細菌和真菌防禦。因此真正需要的是局部、細胞選擇性、可逆的重編程，而不是長期全身免疫壓制。現階段多數方案仍屬臨床前或基於人組織研究的機制推斷。

Q21

問題

能夠改變第 2 型細胞激素訊號的生物製劑，除降低嗜酸性粒細胞數量外，還可能如何重塑氣道黏膜中的嗜酸性粒細胞-肥大細胞-巨噬細胞-樹突狀細胞網路？

回答

改變 2 型細胞激素訊號的生物制劑會重塑整個氣道髓系網路，而不僅僅是降低嗜酸性粒細胞計數。阻斷 IL-5 或 IL-5 受體可減少嗜酸性粒細胞成熟、存活和組織滯留，繼而降低白三烯、顆粒蛋白和相關細胞激素，減少上皮損傷，也減少對巨噬細胞和樹突狀細胞的繼發刺激。

阻斷 IL-4/IL-13 的影響更廣。巨噬細胞接受的 IL-4Rα訊號下降後，部分替代性活化、黏液相關修復和重塑程序會減弱；氣道上皮產生的 2 型趨化因子減少，樹突狀細胞—Th2 反饋也會減弱。抗 IgE 治療降低肥大細胞和嗜鹼性粒細胞 FcεRI 相關活化，因此減少即時介質釋放和後續髓系募集。上游 TSLP 阻斷則可同時削弱樹突狀細胞活化及多個下游 2 型通路。

這也解釋了為甚麼臨床獲益有時明顯大於血嗜酸性粒細胞的變化。反過來，患者即使嗜酸性粒細胞明顯下降，若肥大細胞持續活化、氣道結構重塑或非 2 型發炎仍在，症狀仍可存在。因此更準確的理解是“細胞網路重新接線”，而不是單純“清除嗜酸性粒細胞”。

Q22

問題

在中性粒細胞性氣喘中，哪些針對髓系細胞的策略可能中斷中性粒細胞-巨噬細胞發炎迴路，同時保留抗細菌防禦？這些策略所依據的機制中，哪些已有臨床證據，哪些仍主要處於臨床前階段？

回答

中性粒細胞性氣喘的理想靶向方案，應降低病理性募集和活化，同時保留足夠的抗菌能力。CXCR2 拮抗是最直接的思路之一，可減少中性粒細胞趨化，臨床研究也證明氣道中性粒細胞可以下降，但症狀和結局改善並不穩定，而且存在感染或系統性中性粒細胞減少的顧慮，因此並未成為標準治療。

其他方案成熟度更低。阻斷 IL-1 或 NLRP3 發炎小體可能減少巨噬細胞對中性粒細胞的放大；抑制 GM-CSF 或部分 TLR 通路可能縮短中性粒細胞存活。NET 方向包括 DNase、PAD4 抑制和中性粒細胞彈性蛋白酶抑制；補體/C5a 以及代謝靶點也具有機制合理性。多數在氣喘中仍屬於臨床前或早期轉化階段，廣泛抑制都會帶來抗細菌防禦下降的風險。

目前臨床上有較明確證據的是，大環內酯類可使部分重度非 2 型/中性粒細胞性氣喘患者獲益，但其機制很複雜，包含抗菌、改變氣道菌群和免疫調節，並不是選擇性的“中性粒細胞—巨噬細胞迴路阻斷劑”。因此現階段尚無類似 2 型生物制劑那樣成熟的髓系特異治療。

Q23

問題

訓練性免疫或黏膜疫苗策略是否可能透過有益地重編程單核細胞/巨噬細胞和中性粒細胞來減少復發性尿路感染？請解釋其擬議機制、潛在的組織特異性風險以及目前證據的侷限。

回答

訓練性免疫和黏膜疫苗理論上都可能透過提高膀胱黏膜的早期免疫品質來減少復發性尿路感染。黏膜疫苗可以產生病原特異性 IgA/IgG 和 T 細胞記憶，也可能透過局部細胞激素和基質訊號間接改變巨噬細胞與中性粒細胞。訓練性免疫則可能讓單核/巨噬細胞或骨髓祖細胞發生表觀遺傳、代謝重編程，再次暴露時更快產生細胞激素並更有效募集中性粒細胞。

主要風險是組織特異性不足。若“訓練”過強，可能導致更劇烈的中性粒細胞性膀胱炎、上皮損傷、疼痛甚至纖維化；反復刺激也可能走向免疫耐受，反而削弱有用的細胞激素反應。若是全身性訓練，還可能增加尿路之外的發炎反應。因此理想方案應形成受控的局部免疫記憶，提高早期清菌，但不延長發炎。

目前證據有希望但並不完整。一些疫苗或細菌提取物策略在特定人群中顯示復發率下降，但產品、給藥途徑、終點和研究品質差異較大。多數研究也無法區分獲益究竟來自抗體/T 細胞、髓系訓練、非特異佐劑效應還是微生物組變化。直接證明人膀胱單核/巨噬細胞或中性粒細胞祖細胞發生持久表觀遺傳重編程的證據仍有限。

Q24

問題

如何透過選擇性重編程腫瘤相關巨噬細胞和中性粒細胞來增強膀胱癌的抗腫瘤免疫，同時盡量減少對保護性尿路黏膜免疫的破壞？請比較不同機制策略及其主要轉譯障礙。

回答

膀胱癌中，與其廣泛耗竭腫瘤相關巨噬細胞和中性粒細胞，更合理的是選擇性重編程，因為這些細胞同時承擔尿路抗感染防禦。巨噬細胞方面，可考慮抑制 CSF-1/CSF-1R 或 CCL2 募集，阻斷 PI3Kγ、TREM2 樣免疫抑制程序，或透過 CD47-SIRPα、TLR、STING 等增強吞噬和抗原呈遞，目標是把 IL-10/TGF-β/VEGF 佔優勢的狀態轉向更支持 T 細胞和腫瘤殺傷的狀態。

中性粒細胞方面，CXCL8/CXCR2 或 C5a 軸可用於減少免疫抑制性腫瘤相關中性粒細胞募集；NET、彈性蛋白酶和代謝靶向可能減少侵襲和 T 細胞抑制，同時盡量保留基本吞噬能力。膀胱灌注具有特殊優勢，因為局部給藥有望重塑腫瘤微環境，而不明顯影響全身中性粒細胞和巨噬細胞。BCG 已經提供了“局部先天免疫可用於抗腫瘤”的臨床概念驗證。

主要轉化障礙包括髓系細胞異質性和快速可塑性、通路間代償、感染風險、缺乏可靠生物標誌物、藥物到達腫瘤深部的限制，以及與檢查點抑制劑或化療如何排序。未來更可能依賴空間組學和功能標誌物，精準識別需要重編程的抑制性髓系亞群。

III. Model C

English (EN)

Anonymous Model C | English

Anonymous Model C

English

24 standardized questions

Q01

Question

At the healthy intestinal mucosa, how do neutrophils contribute to barrier defense while interacting with resident macrophages and dendritic cells, and what mechanisms normally limit neutrophil-driven tissue injury?

Response

In a healthy intestine, neutrophils are not abundant residents of the lamina propria; their value lies in being rapidly recruitable when microbes cross the mucus or epithelial barrier. Resident macrophages and epithelial cells sense breach-associated microbial products through pattern-recognition pathways and generate short-range recruitment signals such as CXCL1/2 in mice and CXCL8 in humans, together with IL-1, TNF, complement fragments and lipid chemoattractants. Neutrophils then arrest on activated endothelium, migrate toward the epithelial lesion, and kill extracellular or recently translocated microbes by phagocytosis, NADPH-oxidase-derived reactive oxygen species, antimicrobial granule proteins and, when strongly stimulated, extracellular-trap formation. By rapidly reducing the microbial burden, they can prevent a focal barrier defect from becoming a disseminated mucosal infection.

Resident intestinal macrophages help determine whether this response remains protective. In the steady state they are unusually hyporesponsive to commensal stimulation, yet retain strong phagocytic capacity. They can clear microbes and dying neutrophils without producing the same magnitude of inflammatory cytokines as newly recruited monocytes. Efferocytosis of apoptotic neutrophils through receptors such as MerTK and related engulfment systems shifts macrophages toward IL-10-, TGF-β- and pro-resolving-lipid production, thereby converting an acute neutrophil-dominated reaction into a repair program. Dendritic cells have a different emphasis: they sample antigen and, when danger is sufficient, migrate and initiate adaptive responses. Under homeostatic conditions, however, mucosal dendritic-cell programs favor controlled antigen presentation and tolerance rather than indiscriminate neutrophil recruitment.

Several layers normally restrain neutrophil-mediated injury. The mucus layer, epithelial antimicrobial peptides and intact tight junctions reduce the need for cellular inflammation in the first place. Chemokine expression is spatially and temporally restricted, while IL-10, TGF-β, adenosine signaling, annexin A1 and specialized pro-resolving lipid mediators can reduce further recruitment and activation. Neutrophils are also programmed to undergo apoptosis after a short functional interval; efficient macrophage efferocytosis prevents secondary necrosis and the release of proteases, histones and oxidants. Tissue antiproteases and antioxidant systems further buffer elastase, MPO-derived oxidants and other toxic products.

Thus, healthy mucosal defense depends on a division of labor: neutrophils provide fast, high-intensity antimicrobial activity, macrophages contain and terminate that activity while clearing debris, and dendritic cells link local danger sensing to appropriately scaled adaptive immunity. Injury emerges when recruitment is prolonged, neutrophil death is delayed, extracellular traps and proteases accumulate, or efferocytosis fails—features more typical of active intestinal inflammation than of the healthy mucosa.

Q02

Question

At the healthy intestinal mucosa, how do eosinophils contribute to homeostasis and communicate with macrophages or dendritic cells, and how do these roles differ from eosinophil functions during inflammation?

Response

Eosinophils are a conspicuous granulocyte population in the healthy intestinal lamina propria, so their role is broader than simply executing allergic or antiparasitic inflammation. Their tissue accumulation is supported by constitutive eotaxin-family chemokines, especially CCL11, and by local survival cues. In homeostasis, intestinal eosinophils can contribute to barrier maintenance, regulation of the local microbiota and support of mucosal antibody responses. Experimental studies have implicated eosinophil-derived factors such as IL-6, APRIL-family signals and TGF-β-related pathways in plasma-cell and IgA biology, although the importance of individual mediators varies by species and anatomical site. They can also produce growth and lipid mediators that influence epithelial turnover, extracellular-matrix remodeling and vascular tone.

Their communication with macrophages is often indirect but functionally important. Eosinophil-derived IL-4 and IL-13 can bias macrophages toward repair-associated programs, including enhanced scavenging, matrix remodeling and growth-factor production. Conversely, resident macrophages shape eosinophil survival by controlling local cytokines, clearing apoptotic cells and limiting excessive alarmin production. Dendritic cells can be influenced by eosinophil cytokines and granule-derived ribonucleases; eosinophil-derived neurotoxin, for example, can act as an alarmin under inflammatory conditions and enhance dendritic-cell activation. Eosinophils themselves can express antigen-presentation machinery, but a major antigen-presenting role in normal human intestine is much less firmly established than the canonical role of professional dendritic cells.

The same cell type behaves differently when the tissue enters an inflammatory state. Epithelial alarmins such as IL-33, IL-25 and TSLP, together with IL-5 from ILC2 and Th2 cells and increased eotaxin production, expand and activate eosinophils. Activated eosinophils release major basic protein, eosinophil peroxidase, cationic proteins, reactive oxygen species, cysteinyl leukotrienes and inflammatory cytokines. These products can damage epithelium, increase permeability, activate nerves and amplify dendritic-cell and macrophage inflammatory programs. Persistent IL-4/IL-13 and TGF-β signaling can also push repair toward pathological remodeling or fibrosis rather than restitution.

Accordingly, homeostatic eosinophil activity is best viewed as low-level, spatially controlled trophic and immune-regulatory signaling, whereas inflammatory eosinophilia is characterized by increased cell number, survival and degranulation, stronger type 2 cytokine loops and greater potential for collateral tissue damage. The boundary is not absolute: eosinophils can be protective during infection and reparative after injury, and some purported homeostatic functions remain more secure in animal models than in humans.

Q03

Question

At the healthy respiratory mucosa and during an acute microbial challenge, how do neutrophils coordinate with alveolar or interstitial macrophages and dendritic cells to provide host defense while limiting collateral epithelial injury?

Response

At the healthy respiratory mucosa, alveolar macrophages provide the dominant first-line cellular surveillance in the distal airspaces, while interstitial macrophages and dendritic cells occupy complementary tissue niches. They continuously remove inhaled particles and microbes with relatively restrained inflammatory output. When a genuine microbial threat is detected through TLRs, NOD-like receptors and other sensing systems, macrophages, epithelial cells and recruited myeloid cells produce IL-1, TNF, CXCL chemokines, GM-CSF and lipid chemoattractants. These signals create a rapid but localized wave of neutrophil recruitment. Neutrophils then phagocytose organisms, generate an NADPH-oxidase respiratory burst, deliver MPO and granule proteases into phagolysosomes, and can form extracellular traps when the burden or microbial size makes extracellular containment useful.

Macrophages and neutrophils therefore operate sequentially and reciprocally. Macrophage-derived chemokines recruit neutrophils, while neutrophil products can amplify macrophage activation through microbial debris, IL-1-family signals, oxidized lipids and damage-associated molecules. This amplification is useful early because it accelerates sterilization, but it becomes dangerous if it continues after the microbial load has fallen. Dendritic cells sample antigen and danger signals, then migrate to draining lymph nodes to generate pathogen-specific T-cell responses. During an uncomplicated acute infection, this adaptive arm reinforces microbial clearance without needing to sustain maximal neutrophilic inflammation indefinitely.

Limiting epithelial injury requires active resolution rather than simple disappearance of the initiating pathogen. Neutrophils normally undergo apoptosis and are engulfed by alveolar or interstitial macrophages. Efferocytosis suppresses further NF-κB-driven cytokine production and promotes IL-10, TGF-β, annexin A1 and specialized pro-resolving mediators such as lipoxins and resolvins. Macrophages also clear extracellular debris and proteases. Airway epithelial antiproteases, including secretory leukocyte protease inhibitor and α1-antitrypsin in airway lining fluid, restrain neutrophil elastase, while antioxidant systems buffer oxidant injury. Extracellular DNases help dismantle excess DNA-rich traps.

The protective-pathogenic balance is therefore determined by timing and compartmentalization. A short neutrophil burst can be indispensable in bacterial or fungal infection, whereas persistent CXCL8/GM-CSF signaling, delayed neutrophil apoptosis, defective macrophage efferocytosis or excessive NET/protease release can damage cilia, tight junctions and alveolar epithelium. Severe pneumonia, COPD and some forms of asthma illustrate what happens when these normally self-limited circuits become sustained.

Q04

Question

How do eosinophils interact with macrophages and dendritic cells in the airway mucosa under homeostatic conditions and during type 2 inflammation?

Response

Under homeostatic airway conditions, eosinophils are relatively sparse compared with their abundance during type 2 inflammation. Alveolar macrophages and airway dendritic cells are exposed continuously to inhaled antigens, but in the absence of epithelial danger signals they generally maintain a restrained state. Macrophages remove particles and apoptotic cells, while conventional dendritic cells sample antigen without necessarily providing the strong costimulation needed for a Th2 response. Low-level IL-10, TGF-β and epithelial barrier signals help keep eosinophil recruitment and activation limited.

Type 2 inflammation changes this network at its upstream control points. Injured or allergen-stimulated epithelium releases TSLP, IL-33 and IL-25. These signals activate dendritic cells and ILC2s; dendritic cells upregulate molecules such as OX40L and migrate to lymph nodes, where they promote allergen-specific Th2 differentiation. ILC2 and Th2 cells provide IL-5, which expands and prolongs eosinophil survival, while IL-4 and IL-13 induce epithelial and stromal eotaxins such as CCL11, CCL24 and, especially in humans, CCL26. The result is selective eosinophil accumulation in the airway wall and lumen.

Once recruited, eosinophils feed back on macrophages and dendritic cells. IL-4 and IL-13 from eosinophils can reinforce repair-associated or alternatively activated macrophage programs, but in chronic disease those same programs may support mucus production, extracellular-matrix deposition and fibrosis. Eosinophil granule proteins, leukotrienes, reactive oxygen species and cytokines can injure epithelium and provide additional danger signals to dendritic cells. Eosinophil-derived ribonucleases such as eosinophil-derived neurotoxin can act as alarmins in experimental systems and enhance dendritic-cell activation. Conversely, macrophages can limit the reaction by engulfing apoptotic eosinophils and producing IL-10 and pro-resolving mediators; failure of this clearance favors secondary necrosis and continued inflammation.

Thus, the same cell-cell contacts can be protective or pathogenic depending on intensity and duration. Brief eosinophil-macrophage signaling may aid repair after epithelial injury, whereas sustained IL-5/eotaxin recruitment, repeated degranulation and persistent IL-4/IL-13 signaling convert the circuit into chronic type 2 inflammation. The broad architecture of this pathway is well established in asthma and allergic airway disease, but the exact contribution of direct eosinophil-to-dendritic-cell signaling in humans is more context dependent than the central role of epithelial alarmins and professional dendritic cells.

Q05

Question

In the urinary tract mucosa, how do neutrophils coordinate with monocytes or macrophages and dendritic cells during early antibacterial defense, and what mechanisms prevent excessive tissue injury?

Response

Early antibacterial defense in the urinary tract is initiated largely by the urothelium and resident innate immune cells. Uropathogenic bacteria contacting the bladder or urinary mucosa activate pattern-recognition pathways, including TLR-dependent sensing, leading to production of IL-1-family cytokines, TNF and neutrophil-attracting chemokines. Resident macrophages amplify this alarm and help organize vascular recruitment, while CCR2-dependent monocyte influx can replenish or expand the macrophage compartment during a substantial infection. Neutrophils respond rapidly, traverse the submucosa and urothelium, and enter the urinary space, where they phagocytose bacteria and use ROS, antimicrobial granules and, under strong stimulation, extracellular traps.

Macrophages and monocyte-derived macrophages complement rather than simply duplicate neutrophil function. They can phagocytose organisms and cellular debris, sustain cytokine and chemokine production when the infection is not immediately cleared, and later remove dying neutrophils. Dendritic cells sample microbial antigen and contribute to adaptive priming, although their role in the first hours of an uncomplicated urinary infection is generally less dominant than that of epithelial cells, macrophages and neutrophils. In recurrent or complicated infection, dendritic-cell-mediated T- and B-cell responses become more relevant to the quality of subsequent protection.

The same mechanisms that kill bacteria can injure urothelium. Neutrophil elastase, MPO-derived oxidants, ROS and NET-associated histones can disrupt epithelial junctions and expose deeper tissue. Several brakes normally limit this. Bacterial clearance quickly removes the recruitment stimulus; neutrophils then undergo apoptosis and are engulfed by macrophages. Efferocytosis shifts macrophages toward IL-10-, TGF-β- and repair-associated programs and reduces production of further neutrophil chemokines. Local prostaglandins and specialized pro-resolving lipid mediators can also suppress continued recruitment. Antioxidant and antiprotease systems, together with rapid urothelial regeneration, reduce collateral damage.

There is therefore a trade-off between speed and restraint. Too little neutrophil recruitment allows bacterial expansion and ascending infection, whereas persistent recruitment can produce barrier breakdown and symptomatic inflammation even after bacterial numbers fall. Much of the detailed cellular circuitry is strongly supported by experimental urinary-tract-infection models; the exact relative contributions of individual chemokines, macrophage subsets and NETs in human bladder infection are more heterogeneous and should not be treated as universally fixed.

Q06

Question

In the bladder or lower urogenital mucosa, how can mast-cell mediators influence macrophage and dendritic-cell functions, and how might this network contribute to both host defense and inflammatory symptoms?

Response

Mast cells are positioned close to vessels, nerves and epithelial surfaces in the bladder and lower urogenital tract, allowing a small number of activated cells to influence a much larger myeloid network. Microbial products, IgE-dependent signals, complement, epithelial alarmins and neuropeptides can trigger release of preformed histamine, tryptase, chymase and TNF, followed by newly synthesized prostaglandin D2, cysteinyl leukotrienes, cytokines and growth factors. These mediators rapidly increase blood flow and vascular permeability, alter epithelial signaling and recruit additional leukocytes.

Macrophages and dendritic cells express receptors for many mast-cell products. TNF and GM-CSF can enhance myeloid activation and survival, histamine can either augment or restrain cytokine production depending on the receptor subtype and cellular state, and tryptase can signal through protease-activated receptors such as PAR2. IL-4 and IL-13 can push macrophages toward repair-associated programs, while stronger TNF/IL-1-rich environments favor inflammatory cytokine production. Dendritic cells exposed to mast-cell TNF, histamine or alarmin-rich tissue can mature, migrate and present microbial or environmental antigen more efficiently. In infection, this can be beneficial by accelerating neutrophil recruitment and adaptive priming.

The same network can generate symptoms even when its antimicrobial value is limited. Histamine, tryptase, prostaglandins and nerve-growth-related signals sensitize pelvic afferent nerves and can lower activation thresholds in TRPV1- and purinergic pathways. Vascular leak produces edema, while proteases, TNF and ROS from recruited myeloid cells can weaken urothelial junctions. A more permeable epithelium exposes sensory terminals to urinary solutes and ATP, creating a feed-forward loop of urgency, pain and neurogenic inflammation. Macrophage-derived IL-1β, TNF and prostaglandins can further sensitize nerves and can, in turn, promote mast-cell activation through local cytokine and growth-factor circuits.

Resolution requires that this positive feedback be interrupted. Macrophage efferocytosis, IL-10, TGF-β and pro-resolving lipids can reduce leukocyte recruitment and support epithelial repair; restored barrier integrity then reduces ongoing mast-cell and sensory stimulation. In chronic bladder pain syndromes and other sterile inflammatory states, mast-cell accumulation or activation is reported in subsets of patients, but it is not a universal explanation for disease. Thus, mast-cell-macrophage-dendritic-cell crosstalk is a mechanistically plausible bridge between host defense and symptoms, but its quantitative importance is highly dependent on the initiating cause and patient subgroup.

Q07

Question

During acute intestinal mucosal inflammation and subsequent resolution, how do neutrophils and macrophages influence each other through recruitment signals, cytokines, efferocytosis, lipid mediators, and tissue-repair programs?

Response

During acute intestinal mucosal inflammation, neutrophils and macrophages form a rapidly amplifying circuit. Barrier breach activates epithelial cells and resident macrophages, producing IL-1, TNF, CXCL chemokines, C5a and lipid chemoattractants such as LTB4. These signals recruit neutrophils, which kill microbes but also release ROS, proteases, inflammatory cytokines and additional lipid mediators. Neutrophil-derived LTB4 and damage-associated molecules can recruit or activate more neutrophils and monocytes. CCR2-dependent monocytes entering the tissue differentiate into inflammatory macrophage states that reinforce TNF, IL-1β, IL-6 and chemokine production when microbial or damage signals persist.

Resolution begins when the inflammatory signal changes quality, not merely when cytokine concentrations decline. Neutrophils undergo apoptosis and expose 'eat-me' signals such as phosphatidylserine. Macrophages recognize apoptotic neutrophils through receptors including MerTK, Axl, TIM-family receptors and integrin/bridging-molecule systems. Efferocytosis suppresses macrophage NF-κB-driven inflammatory output and induces IL-10, TGF-β and metabolic programs that favor debris clearance and tissue repair. Because efficient engulfment prevents secondary neutrophil necrosis, it also prevents the late release of histones, elastase, MPO and other highly damaging intracellular contents.

A lipid-mediator class switch is another major control point. Early prostaglandins and leukotrienes facilitate vascular activation and leukocyte recruitment, whereas lipoxins, resolvins, protectins and maresins promote cessation of neutrophil influx, enhance macrophage efferocytosis and support return to tissue homeostasis. Neutrophils can participate in this switch rather than being passive targets of it. Macrophages that have cleared apoptotic cells also produce growth and repair factors—including TGF-β, amphiregulin, VEGF-family signals and matrix-remodeling enzymes—while epithelial stem and stromal compartments rebuild the barrier.

The same pathways become pathogenic if the sequence is interrupted. Excessive neutrophil recruitment, NET formation or protease release expands epithelial damage and produces more DAMPs, while defective efferocytosis leaves necrotic material in the tissue. Conversely,

suppressing neutrophils too early can impair microbial clearance. Modern evidence therefore supports a dynamic continuum of macrophage states rather than a simple 'M1-to-M2' switch: successful resolution depends on coordinated microbial control, neutrophil apoptosis, efferocytic reprogramming and pro-resolving lipid signaling occurring in the correct temporal order.

Q08

Question

How can eosinophil-derived mediators alter macrophage polarization and dendritic-cell activation in the intestinal mucosa, and how could these interactions shift the balance among tolerance, tissue repair, and inflammation?

Response

Eosinophils can alter intestinal macrophage and dendritic-cell behavior through cytokines, lipid mediators and granule-associated proteins, but the outcome depends strongly on whether the eosinophil is participating in homeostasis, repair or overt inflammation. At relatively low activation levels, eosinophil-derived IL-4 and IL-13 can promote macrophage programs associated with scavenging, matrix remodeling and tissue repair. Eosinophils can also produce TGF-β, lipid mediators and growth-related factors that influence stromal and epithelial cells. In some experimental settings, these signals support barrier restitution and local antibody/IgA-associated homeostasis.

Dendritic cells are particularly sensitive to the inflammatory form of eosinophil signaling. Eosinophil granule ribonucleases, including eosinophil-derived neurotoxin, can behave as alarmins and promote dendritic-cell activation in experimental systems. Leukotrienes, cytokines, ROS and epithelial damage caused by granule proteins can increase costimulatory signals and antigen presentation indirectly by creating a danger-rich microenvironment. IL-4 can also condition dendritic-cell function toward type 2-associated priming. Eosinophils themselves may express MHC class II and costimulatory molecules under inflammatory conditions, but they are not considered a substitute for conventional dendritic cells, and the physiological importance of direct eosinophil antigen presentation in human intestine remains uncertain.

These interactions can therefore move the mucosa in three directions. Toward tolerance, restrained eosinophils may support resident macrophage and dendritic-cell programs that limit responses to food and commensals while maintaining IgA and barrier integrity. Toward repair, IL-4/IL-13 and TGF-β can help macrophages clear debris and stimulate tissue reconstruction. Toward inflammation, high IL-5/eotaxin-driven eosinophilia, degranulation, cysteinyl leukotrienes, ROS and alarmin release can activate dendritic cells, recruit additional effector cells and convert macrophage repair responses into chronic remodeling or fibrosis.

The key variable is therefore not a fixed 'eosinophil polarization' but the combination of tissue cues, activation state and duration. Strong epithelial IL-33, IL-25 or TSLP signaling favors inflammatory type 2 amplification; efficient apoptosis and macrophage clearance favor resolution. Some homeostatic eosinophil functions are well demonstrated in mice but less firmly quantified in humans, so direct extrapolation of individual mediators should be cautious even though the overall principle of eosinophil-myeloid crosstalk is well supported.

Q09

Question

During neutrophilic airway inflammation, how do neutrophils and macrophages influence each other through cytokines, lipid mediators, neutrophil extracellular traps, efferocytosis, and metabolic signals, and how can this crosstalk determine persistence versus resolution?

Response

Neutrophilic airway inflammation becomes persistent when macrophages and neutrophils repeatedly re-create the signals that recruited them. Activated airway macrophages, epithelial cells and dendritic cells produce IL-1β, TNF, CXCL8/CXCL-family chemokines and GM-CSF, which recruit neutrophils and prolong their survival. Neutrophils then release ROS, elastase, MPO, cytokines and LTB4, all of which can enhance macrophage activation or generate additional epithelial DAMPs. LTB4 is particularly effective at propagating neutrophil recruitment, while GM-CSF and inflammatory cytokines delay apoptosis, extending the life of cells that would otherwise be rapidly cleared.

NETs can intensify this circuit. Extracellular DNA decorated with histones, elastase and MPO traps microbes, but abundant or poorly degraded NET material injures epithelium and can activate macrophage pattern-recognition and inflammasome pathways. DNA and oxidized proteins provide danger signals, while NET-associated proteases remain catalytically active outside the cell. Macrophages exposed to these products can produce additional IL-1β and chemokines, creating a loop in which NET formation increases recruitment of the cells capable of making more NETs. The precise relative importance of TLR9, cGAS-STING and NLRP3 signaling differs by disease model and should not be assumed to be identical in asthma, COPD and infection.

Metabolism helps determine whether the loop persists. Activated inflammatory macrophages often increase glycolysis and accumulate metabolites such as succinate that can support HIF-1α- and IL-1β-linked programs, whereas neutrophils rely heavily on glycolysis and the pentose-phosphate pathway to sustain antimicrobial ROS production. Hypoxia, extracellular ATP, lactate and adenosine can therefore alter survival and cytokine output. These changes are adaptive during infection but can become maladaptive in chronically inflamed, poorly ventilated airways.

Resolution requires neutrophil apoptosis followed by macrophage efferocytosis. Engulfment through MerTK and related pathways promotes IL-10, TGF-β, lipid-mediator switching and a metabolic transition toward repair. Lipoxins and resolvins reduce further recruitment and enhance clearance. If efferocytosis is defective—as reported in several chronic airway diseases—apoptotic cells progress to secondary necrosis, releasing additional proteases and DAMPs. Thus, persistence reflects coupled failure of recruitment shutoff, NET/debris removal, metabolic de-escalation and efferocytosis; successful resolution requires all four to move in the same direction.

Q10

Question

During allergic airway inflammation, how do mast cells and eosinophils, with basophils where relevant, interact with dendritic cells and macrophages to amplify or restrain type 2 immunity?

Response

Allergic airway inflammation is built around reciprocal amplification among epithelial alarmins, dendritic cells and type 2 effector cells. Allergen exposure or epithelial stress releases TSLP, IL-33 and IL-25, which activate dendritic cells and ILC2s. Conventional dendritic cells upregulate costimulatory programs such as OX40L, migrate to lymph nodes and promote allergen-specific Th2 responses. Th2 cells and ILC2s then produce IL-4, IL-5 and IL-13, establishing the cytokine environment in which mast cells, eosinophils and, in some settings, basophils become major amplifiers.

Mast cells rapidly respond to IgE/FcεRI crosslinking with histamine, tryptase, prostaglandin D2, cysteinyl leukotrienes, TNF and type 2 cytokines. PGD2 acting through CRTH2 and cysteinyl leukotrienes recruit and activate eosinophils, ILC2s and Th2 cells. IL-5 prolongs eosinophil survival, while IL-4/IL-13 induce epithelial eotaxins and adhesion programs. Eosinophils then release leukotrienes, cytokines and granule proteins, injuring epithelium and generating more alarmin signals. Basophils can provide a rapid source of IL-4 and can reinforce Th2 differentiation, but evidence that they replace conventional dendritic cells as principal antigen-presenting cells in human allergic airways is weak and context dependent.

Macrophages can either reinforce or restrain this network. IL-4Rα signaling induces repair-associated macrophage states that can help restore epithelium but can also contribute to mucus hypersecretion, extracellular-matrix deposition and chronic remodeling. Macrophages may produce CCL17/CCL22 and other mediators that support type 2 cell recruitment. In contrast, efficient clearance of apoptotic eosinophils and other cells promotes IL-10, TGF-β and specialized pro-resolving mediators, reducing further recruitment. Dendritic cells can likewise acquire more regulatory phenotypes when epithelial danger decreases and anti-inflammatory signals dominate.

The balance between amplification and restraint therefore depends on whether the circuit remains antigen/alarmin driven. Persistent allergen exposure, barrier dysfunction, IgE signaling and IL-5/eotaxin recruitment favor self-sustaining type 2 inflammation; antigen removal, eosinophil apoptosis, macrophage efferocytosis and restoration of epithelial integrity favor resolution. This explains why therapies that interrupt upstream IgE or type 2 cytokines can alter the whole cellular network rather than merely depleting one granulocyte population.

Q11

Question

During acute bacterial urinary tract infection, how do recruited neutrophils and monocyte-derived macrophages cooperate or compete in pathogen clearance, cytokine production, efferocytosis, and mucosal repair?

Response

In acute bacterial urinary tract infection, neutrophils and monocyte-derived macrophages are recruited on different time scales and therefore contribute complementary functions. Urothelial and resident-myeloid sensing of bacteria generates CXCL chemokines, IL-1, TNF and other inflammatory signals. Neutrophils arrive rapidly, migrate across the urothelium and often dominate the urinary cellular infiltrate. Their main advantage is speed: they phagocytose opsonized bacteria, generate NADPH-oxidase-dependent ROS, release antimicrobial granules and can form extracellular traps when organisms are abundant or difficult to contain.

CCR2-dependent monocytes generally accumulate later and differentiate into inflammatory macrophage states. They can phagocytose bacteria, but they are especially important for sustaining or reshaping the cytokine environment, removing dead cells and transitioning the tissue toward repair. Early macrophage-derived TNF, IL-1β and chemokines support neutrophil recruitment; after the bacterial burden falls, the same macrophage compartment can reduce these signals and increase IL-10, TGF-β and repair factors. Efferocytosis of apoptotic neutrophils is a central switch because it simultaneously removes a source of potentially toxic proteases and reprograms macrophages away from continued recruitment.

Cooperation is therefore most effective when neutrophils provide rapid sterilizing pressure and macrophages provide persistence, quality control and cleanup. Competition can occur, however, for opsonized bacteria, complement components and local metabolic resources. In some settings, macrophages may also internalize bacteria that are not immediately killed, potentially creating a transient intracellular niche rather than improving clearance. Conversely, exuberant neutrophil activity can kill or disable neighboring macrophages through oxidants and proteases and can damage the urothelium sufficiently to expose new bacterial attachment sites.

Mucosal repair requires a timely change in this relationship. Macrophage efferocytosis, matrix remodeling, growth-factor production and coordination with regenerating urothelial cells restore barrier function, while neutrophil recruitment should decline as chemokine production wanes. If neutrophils are suppressed too early, bacterial clearance suffers; if they persist after the infection is controlled, NETs, elastase, MPO and ROS can prolong symptoms and tissue injury. Experimental models strongly support this temporal division of labor, but the exact proportions of recruited macrophage subsets and the frequency with which they provide bacterial reservoirs in human UTI remain variable.

Q12

Question

In chronic bladder mucosal inflammation, how might mast-cell–macrophage crosstalk alter sensory signaling, vascular permeability, epithelial barrier function, and the persistence or resolution of inflammation?

Response

Mast cells and macrophages can form a neuroimmune-vascular feedback loop in chronically inflamed bladder mucosa. Mast cells lie close to afferent nerves and microvessels. When activated by IgE-independent danger signals, neuropeptides, complement or epithelial mediators, they release histamine, tryptase, prostaglandins, leukotrienes, TNF and growth factors. Histamine and proteases increase endothelial permeability and can directly sensitize sensory terminals, while tryptase acting through PAR2 and histamine acting through H1/H4 receptors can lower activation thresholds in pain and urgency pathways. Mast-cell-derived nerve growth factor can further promote sensory hyperexcitability.

Macrophages amplify these effects when they adopt a sustained inflammatory state. IL-1β, TNF, prostaglandin E2 and ROS can sensitize nociceptive neurons and maintain endothelial activation. Neurogenic mediators released from activated nerves—such as substance P and CGRP—can in turn affect mast cells, vessels and macrophages, creating a feed-forward loop. Cytokines and growth factors including CCL2/CCR2- and CSF1-related signals can continuously recruit or sustain monocyte/macrophage populations, while stem-cell-factor/c-Kit signaling can support local mast-cell survival.

Barrier dysfunction links the sensory and inflammatory arms. Mast-cell proteases, macrophage cytokines and recruited-neutrophil oxidants can disrupt urothelial tight-junction proteins and increase paracellular permeability. Damaged urothelial cells release ATP and other danger signals, exposing subepithelial afferents to urinary solutes and activating purinergic receptors. The resulting sensory firing further promotes local mediator release. Increased vascular leak adds edema and raises local tissue pressure, potentially worsening urgency and pain even in the absence of high microbial burden.

Resolution requires macrophages to switch from inflammatory cytokine production to efferocytosis, IL-10/TGF-β signaling, pro-resolving lipid production and support of epithelial regeneration. Restored urothelial integrity reduces access of urinary irritants to nerves and removes a major driver of mast-cell activation. If TGF-β-rich repair becomes excessive, however, fibrosis and altered compliance can themselves perpetuate symptoms. These mechanisms are biologically plausible and supported in experimental systems, but chronic bladder pain syndromes are heterogeneous: mast-cell abundance and activation vary substantially among patients, so this axis should be regarded as one possible disease-maintaining circuit rather than a universal explanation.

Q13

Question

In ulcerative colitis, how do neutrophil recruitment, neutrophil extracellular trap formation, neutrophil metabolism, and neutrophil–macrophage crosstalk contribute to mucosal injury versus inflammatory resolution?

Response

In ulcerative colitis, neutrophils are both essential antimicrobial effectors and major contributors to visible mucosal pathology. Inflamed colonic epithelium, stromal cells and monocyte-derived macrophages produce CXCL8 and related CXCR1/2 ligands, IL-1, TNF and colony-stimulating factors that drive intense neutrophil recruitment. Neutrophils migrate across the epithelium into crypts, producing the characteristic cryptitis and crypt abscesses of active disease. Their phagocytosis and ROS production help contain bacteria that cross a compromised barrier, but extracellular elastase, MPO-derived oxidants, MMPs and ROS can enlarge epithelial erosions and expose additional microbial products.

NET formation adds a second mechanism of injury. DNA-histone scaffolds can trap microbes, yet persistent NETs retain elastase and MPO, damage epithelial membranes, increase thrombogenic and inflammatory signals and provide DAMPs to macrophages. NET-derived DNA and proteins can activate nucleic-acid sensing and inflammasome-linked pathways, although the importance of specific sensors such as TLR9, cGAS-STING or NLRP3 differs among models. Human studies commonly associate NET markers with active disease, but the degree to which NETs are a primary causal driver rather than a marker of severe neutrophilic inflammation remains less certain.

Neutrophil metabolism helps sustain this phenotype. These cells rely heavily on glycolysis and the pentose-phosphate pathway, which supplies NADPH for the oxidative burst. The hypoxic, metabolically stressed inflamed mucosa can stabilize HIF-linked survival programs and delay apoptosis. Lactate, succinate and extracellular ATP produced by inflamed tissue can also influence macrophage and neutrophil activation. Thus, metabolic adaptation that is useful for antimicrobial function can prolong survival and inflammatory output when the stimulus becomes chronic.

Resolution depends on macrophages changing the meaning of neutrophil presence from 'danger' to 'cleanup.' Engulfment of apoptotic neutrophils through MerTK and related efferocytic pathways suppresses inflammatory cytokines and promotes IL-10, TGF-β, lipoxins, resolvins and tissue-repair programs. Failed efferocytosis permits secondary necrosis and further DAMP release. The therapeutic challenge is therefore not to eliminate neutrophils indiscriminately, but to reduce excessive recruitment and extracellular toxicity while preserving phagocytic killing and accelerating apoptosis, efferocytosis and barrier repair.

Q14

Question

In Crohn’s disease, how can dysregulated interactions among neutrophils, monocyte-derived macrophages, and dendritic cells alter microbial handling, antigen presentation, inflammatory persistence, and epithelial repair?

Response

Crohn's disease can be viewed partly as a failure to complete the normal sequence from microbial sensing to killing, antigen handling and repair. Barrier defects allow luminal organisms and microbial products to reach the lamina propria. Neutrophils are recruited to contain this breach, but abnormalities in microbial handling can make that response either insufficient or chronically repeated. Susceptibility pathways involving NOD2, ATG16L1 and related autophagy and bacterial-sensing machinery illustrate how impaired intracellular handling can allow microbial material to persist and repeatedly stimulate myeloid cells.

Recruited CCR2-positive monocytes differentiate into macrophages that, in active Crohn's disease, may fail to acquire the hyporesponsive phenotype typical of healthy intestinal resident macrophages. Instead they continue to produce TNF, IL-1β, IL-6, IL-23 and chemokines that recruit neutrophils and support Th17/Th1-associated responses. Neutrophil ROS, proteases and NETs can control microbes but also enlarge epithelial injury and release additional DAMPs. In granulomatous disease, macrophage persistence and incomplete processing of microbial or particulate material can further sustain local cytokine production.

Dendritic cells integrate this innate inflammation with adaptive immunity. Enhanced exposure to microbial antigen, together with IL-12/IL-23-family signals and strong costimulation, promotes inflammatory T-cell differentiation rather than tolerance. Activated T cells then return to the tissue and supply IFN-γ, IL-17-family and other mediators that reinforce macrophage and epithelial chemokine production. This creates a circuit in which defective microbial clearance increases antigen presentation, and the resulting adaptive inflammation further damages the barrier, allowing more microbial access.

Successful repair requires macrophage efferocytosis and growth-factor programs, epithelial stem-cell restitution and controlled extracellular-matrix remodeling. Persistent TNF/IL-1 signaling, protease activity and hypoxia can interfere with these processes, while excessive TGF-β-rich repair can promote fibrosis and stricturing. Anti-TNF efficacy supports a causal role for inflammatory cytokine circuits, but Crohn's disease is heterogeneous: the balance among defective innate killing, hyperactive monocyte/macrophage inflammation, dendritic-cell antigen presentation and fibrotic repair differs among patients and disease locations. A simple 'overactive immunity' model therefore misses the important coexistence of impaired microbial handling and excessive secondary inflammation.

Q15

Question

In severe neutrophilic asthma, how do neutrophils, macrophages, and dendritic cells form self-sustaining inflammatory circuits, and what roles may neutrophil extracellular traps, inflammasome signaling, microbial signals, and corticosteroid resistance play?

Response

Severe neutrophilic asthma can be sustained by a myeloid-epithelial-Th17 circuit rather than the classic eosinophilic type 2 pathway. Airway epithelial cells and macrophages exposed to pollutants, infection, DAMPs or dysbiotic microbial products activate TLR/NOD and NF-κB pathways and release IL-1β, TNF, CXCL8 and GM-CSF. Dendritic cells and macrophages can promote IL-6/IL-23-dependent Th17 responses; IL-17 then drives epithelial CXCL8 and G-CSF production, recruiting and prolonging neutrophil survival. Neutrophil elastase, ROS and additional DAMPs feed back on epithelium and macrophages, creating a self-reinforcing loop.

NETs can strengthen this circuit. Extracellular DNA, histones, MPO and elastase increase mucus viscosity, injure epithelium and provide signals that activate macrophage and dendritic-cell innate sensors. In several models, NET components and extracellular ATP promote NLRP3-inflammasome activation and IL-1β release, which in turn favors neutrophilic and Th17 inflammation. NLRP3 involvement is biologically

plausible and supported in subsets of severe asthma, but it is not uniform across all patients; 'neutrophilic asthma' itself contains infectious, smoking-related, obesity-associated and treatment-modified endotypes.

Microbial signals can provide a persistent trigger. Altered airway bacterial communities, chronic colonization or repeated infections supply TLR ligands that maintain CXCL8/IL-1 pathways. This may help explain why macrolides benefit some patients beyond their direct antibacterial effects, although antimicrobial resistance and microbiome disruption are important trade-offs. Persistent infection must also be distinguished from sterile inflammation because suppressing neutrophils in an infection-driven endotype could be harmful.

Corticosteroid resistance is multifactorial. Neutrophilic/Th17 inflammation is generally less steroid-responsive than eosinophilic type 2 disease; oxidative stress and p38-MAPK-related changes can impair glucocorticoid receptor function, and corticosteroids can delay neutrophil apoptosis even while suppressing some cytokines. Reduced HDAC2 activity has been implicated in oxidative-stress-related steroid resistance, although the strength of this mechanism varies between asthma and COPD. Thus, severe neutrophilic asthma persists when recruitment signals, NET/DAMP production, inflammasome activity, microbial stimulation and impaired resolution converge. Effective intervention must break the circuit without removing the antibacterial functions that are particularly important in this phenotype.

Q16

Question

In eosinophilic asthma, how do eosinophils, mast cells, and where relevant basophils interact with macrophages, dendritic cells, and airway epithelium to initiate, amplify, and resolve type 2 inflammation?

Response

Eosinophilic asthma begins when the airway epithelium converts environmental exposure into a type 2 alarm. Allergens, viruses, proteases and pollutants can induce epithelial IL-33, TSLP and IL-25. These alarmins activate ILC2s and condition conventional dendritic cells, which migrate to lymph nodes and prime allergen-specific Th2 cells. Th2 cells and ILC2s release IL-5 to expand and sustain eosinophils, while IL-4 and IL-13 drive epithelial eotaxins, mucus production and additional type 2 chemokines. Mast cells and basophils are recruited into the same circuit through IgE and cytokine-dependent pathways.

Amplification occurs when these effector cells begin to signal to one another. IgE-crosslinked mast cells rapidly release histamine, prostaglandin D2 and cysteinyl leukotrienes, which cause bronchoconstriction and recruit CRTH2-positive eosinophils, ILC2s and Th2 cells. Basophils supply IL-4 and can reinforce type 2 polarization, although conventional dendritic cells remain the dominant professional antigen-presenting cells. Eosinophils release leukotrienes, IL-4/IL-13, granule proteins and ROS, causing epithelial injury and more alarmin release. IL-4Rα signaling in macrophages promotes repair-associated states that can clear debris but, when persistent, may support mucus hypersecretion and tissue remodeling.

The epithelium is not a passive target. IL-13 induces goblet-cell metaplasia and mucus genes such as MUC5AC, while epithelial eotaxins determine tissue eosinophil localization. Damaged epithelium sustains TSLP/IL-33 output and therefore keeps dendritic cells and ILC2s activated. Chronic eosinophil- and mast-cell-derived TGF-β, proteases and lipid mediators can promote smooth-muscle changes and subepithelial fibrosis, converting episodic inflammation into structural airway remodeling.

Resolution requires withdrawal of antigen/alarmin signaling, eosinophil apoptosis and efficient macrophage efferocytosis. Engulfment of apoptotic eosinophils and neutrophils favors IL-10, TGF-β in a reparative context, and specialized pro-resolving lipids, while regulatory T cells and less activated dendritic cells reduce renewed Th2 priming. Because each node is connected, blocking a single upstream cytokine can change mast-cell activation, eosinophil trafficking, macrophage state and epithelial behavior simultaneously. The broad pathway is well established; the exact contribution of basophils and direct eosinophil antigen presentation is more context dependent.

Q17

Question

In recurrent urinary tract infection, how can prior infection reshape neutrophil and monocyte or macrophage responses at the bladder mucosa through trained immunity, tolerance, or tissue remodeling, and how strong is the evidence for each mechanism?

Response

Prior urinary infection can plausibly alter later neutrophil and monocyte/macrophage responses through three distinct processes: trained innate immunity, tolerance and structural tissue remodeling. These mechanisms should not be conflated, because they predict different secondary responses. Trained immunity involves durable epigenetic and metabolic changes—often increased accessibility of inflammatory or antimicrobial genes, enhanced glycolysis and altered mevalonate or mitochondrial pathways—in long-lived myeloid progenitors or tissue macrophage populations. A subsequent challenge can then produce faster cytokine release, phagocytosis and neutrophil recruitment. Because mature neutrophils are short-lived, persistent 'neutrophil memory' would more likely be imposed at the bone-marrow progenitor level or by a remodeled bladder environment than retained by the original neutrophils themselves.

Tolerance predicts the opposite phenotype. Repeated TLR/LPS exposure can epigenetically suppress subsets of TNF-, IL-1- and chemokine genes while preserving or even enhancing selected antimicrobial and repair pathways. In the bladder, such a state could reduce damaging secondary inflammation but might also permit recurrent bacterial growth if neutrophil recruitment becomes too weak. Direct demonstration of classical endotoxin-tolerance programs in human recurrent UTI myeloid cells is limited, so this remains mechanistically plausible rather than established as a dominant clinical mechanism.

Tissue remodeling currently has particularly strong experimental support. Prior infection can alter urothelial differentiation, barrier properties, stromal chemokine production and local immune-cell niches; intracellular bacterial reservoirs or persistent antigen can also change the baseline inflammatory set point. These changes can make a second infection either more inflammatory or more resistant, depending on the model and prior injury severity. Macrophage numbers, localization and activation thresholds may change even if each cell has not undergone autonomous 'training.'

The evidence hierarchy is therefore uneven. Infection-induced structural and epithelial remodeling is well documented in animal models and is biologically consistent with recurrent human disease. Trained-immunity-like reprogramming of monocytes/macrophages is strongly established in other infectious settings and increasingly plausible in the urinary tract, but direct human bladder evidence remains sparse. Neutrophil-intrinsic long-term memory is least convincing because of cell lifespan. Tolerance is also plausible from general innate-immune biology but not yet clearly quantified in recurrent UTI. Human longitudinal studies that combine tissue sampling, single-cell epigenomics and functional secondary-challenge assays are needed to distinguish these mechanisms.

Q18

Question

In bladder cancer, how do tumor-associated neutrophils and macrophages interact within the mucosal tumor microenvironment to influence antigen presentation, T-cell activity, angiogenesis, invasion, and response to immunotherapy?

Response

Tumor-associated macrophages and neutrophils in bladder cancer can form a mutually reinforcing immunosuppressive and tissue-remodeling network. Tumor cells and stromal cells produce CCL2/CSF1-family signals that recruit and sustain macrophages, and CXCL8/CXCR2 ligands, G-CSF and other chemokines that recruit neutrophils. In a tumor-conditioned environment, macrophages can reduce IL-12 and effective antigen presentation while increasing IL-10, TGF-β, arginase activity and PD-L1. Neutrophils can likewise acquire suppressive functions through arginase, ROS, proteases and NET formation. These states are heterogeneous and should not be reduced to rigid 'M1/M2' or 'N1/N2' categories.

Together, the two populations can suppress T-cell activity at several levels. Macrophage IL-10/TGF-β and PD-L1 blunt T-cell activation, while neutrophil ROS and arginine depletion impair T-cell receptor signaling and proliferation. Both cell types can recruit regulatory lymphocytes or other suppressive myeloid cells. Poor dendritic-cell maturation and antigen presentation further reduce productive priming. Conversely, inflammatory macrophages with intact MHC-II, costimulation and IL-12, and appropriately activated neutrophils with cytotoxic antimicrobial machinery, can support antitumor immunity; the tumor microenvironment shifts the balance between these opposing programs.

Angiogenesis and invasion are also cooperative outputs. TAM-derived VEGF, MMPs and TGF-β promote endothelial growth, matrix remodeling and epithelial-mesenchymal-like tumor programs. Neutrophil MMP9, elastase, VEGF-related signals and NETs can open extracellular-matrix paths, trap circulating tumor cells and facilitate invasion or metastasis in experimental models. Hypoxia reinforces both macrophage and neutrophil suppressive programs through HIF-dependent pathways.

These interactions influence therapy response. High systemic neutrophil-to-lymphocyte ratios and myeloid-rich tumors often correlate with worse outcome, but correlation does not prove a single causal pathway. Myeloid PD-L1, poor antigen presentation and T-cell exclusion can reduce checkpoint-blockade efficacy. At the same time, intravesical BCG demonstrates that bladder myeloid cells can be harnessed therapeutically: BCG induces intense local neutrophil and macrophage activation that contributes to antitumor immunity. Therefore, indiscriminate depletion could remove useful host-defense and BCG-responsive functions. The therapeutic objective is selective reprogramming—reducing suppressive, angiogenic and NET-rich states while preserving phagocytosis, antigen presentation and local antimicrobial capacity.

Q19

Question

Which mechanistically plausible strategies could suppress pathogenic neutrophil recruitment or neutrophil-extracellular-trap-associated injury in ulcerative colitis while preserving antimicrobial defense and macrophage-mediated repair? Explain predicted benefits, trade-offs, and evidence gaps.

Response

A useful strategy in ulcerative colitis would reduce the extracellular and tissue-destructive arm of neutrophil biology more than the phagocytic antimicrobial arm. One approach is partial or local blockade of the CXCL8-CXCR1/2 recruitment axis. This could reduce crypt accumulation, ROS and protease exposure, but systemic or profound CXCR2 inhibition risks impaired bacterial defense and has not become an established UC therapy. Local colonic delivery, biomarker-guided dosing or short treatment windows are conceptually attractive because they may preserve circulating neutrophil competence.

NET-focused approaches are potentially more selective. PAD4 inhibition can reduce one form of chromatin decondensation and NETosis in several models, but NET formation is not universally PAD4-dependent and PAD4 also has functions outside neutrophils. Extracellular DNase can dismantle DNA scaffolds, yet digestion alone may release still-active elastase, MPO and histones; combining DNase with protease or histone-neutralizing strategies may be safer in principle. MPO or neutrophil-elastase inhibition could reduce oxidative and proteolytic injury while leaving phagocytosis relatively intact, but complete inhibition would still weaken microbial killing.

A third strategy is to accelerate physiological resolution rather than block entry. Annexin A1, lipoxin/resolvin pathways and macrophage efferocytosis through MerTK-related systems can stop further neutrophil recruitment, shorten neutrophil lifespan in tissue and enhance clearance of apoptotic cells. This has the theoretical advantage of preserving the early antimicrobial burst while preventing the late toxic phase. Enhancing macrophage repair programs could also restore epithelial integrity and thereby remove the microbial stimulus that keeps neutrophils recruited. Excessive pro-repair signaling, however, could promote fibrosis or suppress needed antimicrobial cytokines.

Upstream anti-inflammatory therapies used in IBD can indirectly reduce neutrophil recruitment by lowering TNF, IL-23-associated and epithelial chemokine signaling, but they are not selective neutrophil-sparing solutions. The major evidence gap is that most NET-, CXCR2- and pro-resolving strategies remain preclinical or have not shown clear clinical benefit in UC. Future trials need functional endpoints beyond neutrophil counts: mucosal bacterial control, NET burden, macrophage efferocytosis, epithelial healing and infection rates should all be measured. The likely safest design is local and state-specific modulation rather than systemic neutrophil depletion.

Q20

Question

How could metabolic or epigenetic reprogramming of intestinal macrophages and granulocytes be used to restore mucosal homeostasis in inflammatory bowel disease, and what mechanisms could cause loss of efficacy or unintended immunosuppression?

Response

Metabolic and epigenetic reprogramming offers a way to change what intestinal myeloid cells do rather than simply remove them. In inflammatory macrophages, increased glycolysis, succinate-HIF-1α signaling, mTOR activity and inflammatory lipid metabolism can reinforce IL-1β, TNF and chemokine production. Interventions that activate AMPK, improve mitochondrial oxidative metabolism, enhance Nrf2/itaconate-related antioxidant programs or reduce excessive glycolytic flux could shift macrophages toward efferocytosis and repair. Epigenetically, BET proteins, histone acetylation/deacetylation and methylation pathways regulate accessibility of inflammatory genes and can, in principle, erase maladaptive trained-immunity-like states.

Granulocytes require a different degree of caution. Neutrophils rely heavily on glycolysis and the pentose-phosphate pathway for migration, survival and NADPH-oxidase-dependent microbial killing. Broad inhibition of glycolysis or NADPH generation might reduce ROS-mediated

mucosal injury but could simultaneously cripple antibacterial defense. A better goal would be to suppress prolonged survival, excessive NET formation or extracellular degranulation while preserving phagolysosomal killing. Targeting HIF-linked persistence, selected lipid pathways or NET-associated chromatin programs is mechanistically plausible but remains largely preclinical in IBD.

Loss of efficacy can arise from myeloid plasticity and constant cell replacement. Intestinal monocytes are continuously recruited from blood, so a locally reprogrammed macrophage population can be diluted by new inflammatory cells. Cytokines such as TNF, GM-CSF, IL-1 and IL-23 can override a metabolic intervention, while microbiota-derived metabolites can push the same pathway in the opposite direction. Redundant metabolic routes, disease heterogeneity and drug penetration into inflamed or fibrotic tissue also allow escape.

Unintended immunosuppression is the major translational risk. Excessive inhibition of glycolysis, mTOR, ROS or chromatin accessibility can reduce phagocytosis, inflammasome function, cytokine-mediated recruitment and antigen presentation needed for infection control. Overdriving repair-associated macrophage programs can increase TGF-β and fibrosis or reduce surveillance against dysplasia. Systemic epigenetic drugs may also affect lymphocytes and epithelial stem cells. Consequently, the most credible route is cell- and tissue-selective delivery with reversible dosing, combined with biomarkers of macrophage state, neutrophil antimicrobial function and mucosal infection. Most of these approaches are still mechanistic or preclinical rather than established IBD treatments.

Q21

Question

How might biologics that alter type 2 cytokine signaling reshape the eosinophil–mast-cell–macrophage–dendritic-cell network in airway mucosa, beyond simply reducing eosinophil counts?

Response

Type 2 biologics reshape airway immunity at multiple cellular levels because IL-5, IL-4/IL-13 and upstream epithelial cytokines are network signals, not merely eosinophil growth factors. Anti-IL-5 or anti-IL-5R therapy reduces eosinophil production, survival and tissue persistence, but it also removes eosinophil-derived leukotrienes, granule proteins, TGF-β and type 2 cytokines that would otherwise activate epithelium, macrophages and dendritic cells. Benralizumab, through IL-5Rα-directed antibody-dependent cytotoxicity, can additionally reduce basophils, changing an early IL-4 source as well as eosinophil numbers.

Blocking IL-4Rα has broader consequences because it interrupts both IL-4 and IL-13 signaling. Airway epithelium produces less eotaxin, mucus and nitric-oxide-associated type 2 output; B cells receive less IgE class-switching support; macrophages are less strongly driven into IL-4/IL-13-conditioned repair/remodeling states; and dendritic-cell trafficking or chemokine environments can change. A clinically notable consequence is that tissue eosinophil trafficking may fall before circulating eosinophil production does, producing transient blood eosinophilia in some patients. That observation itself illustrates why cell counts in blood do not fully represent what has happened to the mucosal network.

Upstream blockade can be broader still. TSLP inhibition can reduce dendritic-cell activation, ILC2 stimulation and the epithelial permissive signals that support mast cells and basophils, thereby lowering both type 2 and some non-type-2 inflammatory outputs. IL-33/ST2-directed approaches are also mechanistically capable of reducing ILC2, mast-cell and eosinophil activation, although their clinical roles have been less established than those of approved IL-5-, IL-4Rα- and TSLP-directed therapies.

These network effects may improve epithelial barrier function and reduce the need for macrophage-driven cleanup, but they can also alter repair. Removing chronic IL-13 and eosinophil granule injury is beneficial, whereas excessive suppression of type 2 repair pathways could theoretically change responses to helminths or tissue damage. Mast cells may persist despite successful eosinophil depletion because their survival is not primarily IL-5 dependent. Therefore, treatment response depends on which node is blocked: anti-IL-5 mainly collapses the eosinophil arm, IL-4Rα blockade alters several stromal and myeloid programs, and upstream alarmin blockade can reconfigure the entire dendritic-cell–ILC2–granulocyte network.

Q22

Question

What myeloid-directed strategies could interrupt neutrophil–macrophage inflammatory circuits in neutrophilic asthma while preserving antibacterial defense, and which proposed mechanisms are clinically established versus preclinical?

Response

The central therapeutic problem in neutrophilic asthma is that the same neutrophil-macrophage circuit that damages airways also protects against bacteria. A rational approach is therefore partial, local or state-specific interruption rather than systemic myeloid depletion. CXCR2 antagonism can markedly reduce airway neutrophil recruitment by blocking responses to CXCL8-family ligands. Clinical studies have shown target engagement and lower sputum neutrophils, but consistent improvements in asthma outcomes have been limited, and infection risk remains a concern. Thus CXCR2 blockade is mechanistically validated but not an established standard treatment for neutrophilic asthma.

Macrolides, especially long-term azithromycin in selected patients with persistent or severe asthma, have the strongest clinical evidence for a treatment with relevant myeloid effects. Their benefit is not simply antibacterial: they can reduce neutrophil chemotaxis, macrophage cytokine production and some inflammasome-related signaling. However, the precise mechanism responsible for fewer exacerbations is unresolved, and antimicrobial resistance, QT effects and microbiome disruption prevent viewing macrolides as a clean macrophage-neutrophil reprogramming therapy. Upstream biologics such as anti-TSLP are clinically established for severe asthma across a broad phenotype range, but they are not specifically myeloid-directed.

Several more selective ideas remain mainly preclinical or early translational. NLRP3 or IL-1-pathway modulation could reduce macrophage-neutrophil amplification, but broad IL-1 suppression may weaken host defense. NET-targeted DNase, PAD4 inhibition, MPO/elastase inhibitors and histone-neutralizing strategies aim to reduce extracellular toxicity while retaining phagocytosis; convincing asthma efficacy in humans is not yet established. Pro-resolving mediators, MerTK-enhancing approaches and macrophage efferocytosis therapies are attractive because they would accelerate neutrophil clearance instead of preventing the initial antibacterial response, but they remain experimental.

The most promising design principles are inhaled delivery, biomarker selection and preservation of early killing functions. Patients with chronic bacterial colonization should be distinguished from those with sterile IL-1/Th17-driven inflammation. Clinical endpoints should include exacerbations and lung function together with bacterial burden, sputum NETs, efferocytosis and infection frequency. At present, no therapy is clinically established specifically because it interrupts the neutrophil-macrophage circuit while demonstrably preserving antibacterial defense; that remains the key translational gap.

Q23

Question

Could trained-immunity or mucosal-vaccine approaches beneficially reprogram monocytes or macrophages and neutrophils to reduce recurrent urinary tract infection? Explain the proposed mechanisms, potential tissue-specific risks, and current evidence limitations.

Response

Trained-immunity and mucosal-vaccine strategies could, in principle, reduce recurrent UTI by raising the baseline readiness of bladder innate immunity without maintaining chronic inflammation. Trained immunity is most plausibly induced in long-lived monocyte/macrophage progenitors or tissue macrophages through pattern-recognition pathways such as NOD- or C-type-lectin signaling, followed by durable changes in histone accessibility, glycolysis and mevalonate/mitochondrial metabolism. On re-exposure, these cells could produce chemokines faster, phagocytose bacteria more efficiently and recruit neutrophils earlier. Mature neutrophils are too short-lived to retain long-term cell-autonomous memory, but bone-marrow progenitor reprogramming or a trained tissue environment could generate functionally 'primed' neutrophils during later infection.

A mucosal vaccine adds adaptive specificity. Antigen presentation by dendritic cells can generate urinary-tract-homing T cells, antibody and mucosal IgA/IgG responses, while vaccine adjuvants may simultaneously train innate cells. Oral or sublingual bacterial-lysate approaches and experimental multivalent vaccines have shown encouraging reductions in recurrence in some studies, but their immune mechanisms are difficult to separate into adaptive immunity, trained innate immunity and nonspecific exposure effects.

The tissue-specific risks are substantial. A bladder that is too readily 'trained' may respond to low-level bacterial exposure with excessive CXCL8, neutrophil ROS, NETs and pain-producing inflammation. Repeated innate stimulation could also produce tolerance rather than training, blunting TNF/IL-1 responses and permitting bacterial persistence. Remodeling of urothelial stem-cell or stromal programs might create durable barrier improvement, but maladaptive remodeling could increase fibrosis, sensory hypersensitivity or susceptibility to intracellular bacterial reservoirs. Cross-reactive adaptive responses and microbiome disruption are additional concerns.

Current evidence therefore supports the concept more strongly than a defined myeloid mechanism in humans. Recurrent-UTI immunoprophylaxis has clinical signals, but study designs, products, regulatory status and endpoints are heterogeneous. Direct demonstrations of trained macrophage chromatin states in human bladder tissue, progenitor-level neutrophil reprogramming and causal links to fewer recurrences are sparse. The decisive studies would combine randomized clinical outcomes with bladder or urinary single-cell profiling, epigenetic assays and ex vivo secondary-challenge tests to show that protection truly reflects beneficial myeloid reprogramming rather than only conventional adaptive immunity.

Q24

Question

How could selective reprogramming of tumor-associated macrophages and neutrophils improve antitumor immunity in bladder cancer while minimizing disruption of protective urinary mucosal immunity? Compare mechanistic approaches and key translational barriers.

Response

Selective myeloid reprogramming in bladder cancer should aim to convert tumor-associated macrophages and neutrophils from suppressive, angiogenic and tissue-remodeling states into cells that support antigen presentation and T-cell killing, while leaving ordinary urinary antimicrobial defense intact. For macrophages, one approach is functional activation rather than depletion: CD40, TLR or STING agonism, inhibition of PI3Kγ-like suppressive signaling, or blockade of phagocytosis checkpoints such as CD47-SIRPα can increase tumor-cell uptake, MHC-II/costimulatory activity and IL-12-type inflammatory programs. CSF1R blockade can reduce suppressive TAM numbers, but broad depletion risks loss of tissue repair and antimicrobial phagocytosis.

Neutrophils can be targeted at recruitment or effector state. Partial CXCR2/CXCL8-axis inhibition may reduce the influx of tumor-promoting neutrophils and myeloid suppressor cells. TGF-β-pathway modulation may favor a more inflammatory antitumor neutrophil state, while PAD4/NET-, MPO- or elastase-directed strategies could reduce NET-dependent invasion and vascular effects. The problem is that CXCR2 signaling, ROS and granule enzymes are also central to bacterial defense. Therefore, tumor-localized or intermittent inhibition is preferable to chronic systemic neutrophil suppression.

Bladder cancer offers a practical route to selectivity: intravesical delivery. Local agonists, nanoparticles or tumor-microenvironment-responsive formulations can expose tumor-infiltrating myeloid cells to high concentrations while limiting systemic effects. BCG already provides proof of principle that intense local innate activation—including neutrophils and macrophages—can be therapeutically useful. That fact argues strongly against indiscriminate myeloid depletion. Combination with checkpoint blockade may be most effective when reprogramming improves antigen presentation and reduces T-cell exclusion before PD-1/PD-L1 inhibition.

The main translational barriers are myeloid plasticity, spatial heterogeneity and lack of reliable biomarkers distinguishing protective from tumor-promoting cells. Mouse TAM/TAN markers do not map cleanly to human bladder tumors; the same macrophage or neutrophil can change state over hours. Local drug penetration into bulky or necrotic tumors, compensatory recruitment of other suppressive myeloid cells, infection risk, impaired mucosal repair and interactions with BCG are additional concerns. The most credible strategy is therefore spatially restricted, reversible reprogramming guided by tumor and urinary biomarkers, rather than permanent depletion of an entire myeloid lineage.

Japanese (JP)

Anonymous Model C | 日本語

Anonymous Model C

日本語

24 standardized questions

Q01

質問

健常な腸管粘膜において、好中球は常在マクロファージおよび樹状細胞と相互作用しながら、どのようにバリア防御に寄与しますか。また、好中球による組織障害を通常どのような機序が抑制していますか。

回答

健常な腸管粘膜では、好中球は固有層に大量に常在する細胞ではなく、バリアが局所的に破綻したときに迅速に動員されることに意義があります。上皮細胞や常在マクロファージが微生物由来分子や組織損傷をパターン認識受容体で感知すると、CXCL1/2、ヒトでは CXCL8 を代表とするケモカイン、IL-1、TNF、補体断片、脂質性走化因子などが産生されます。好中球はこれらの勾配に沿って血管外へ移動し、貪食、NADPH オキシダーゼ依存性の活性酸素、MPO、顆粒内抗菌分子を用いて侵入微生物を排除します。強い刺激では NET 形成も起こり得ます。局所の微生物量を短時間で下げ、限局したバリア欠損が広範な感染へ進むのを防ぐことが主な防御的意義です。

常在マクロファージは、この高出力の反応を必要な範囲にとどめる重要な制御役です。健常腸管のマクロファージは、共生菌由来刺激に対する炎症性サイトカイン産生が比較的抑えられている一方、貪食能は保たれています。微生物に加え、役割を終えてアポトーシスに入った好中球を MerTK などの経路を介してエフェロサイトーシスすると、NF-κB 型の炎症出力が低下し、IL-10、TGF-β、炎症収束性脂質メディエーターが増加します。樹状細胞は抗原採取と適応免疫への橋渡しを担いますが、恒常状態では強い共刺激を伴わない制御的な抗原提示が優位で、無制限な好中球動員を起こしにくい環境が維持されます。

組織障害を抑える仕組みは多層的です。粘液、抗菌ペプチド、タイトジャンクションがまず微生物の接触自体を減らし、ケモカイン産生は時間的･空間的に限定されます。IL-10、TGF-β、アデノシン、Annexin A1、リポキシンやレゾルビンなどは追加の動員と活性化を抑えます。好中球は本来短寿命で、刺激が低下するとアポトーシスし、マクロファージに速やかに除去されます。これにより二次性壊死によるエラスターゼ、ヒストン、MPO、酸化物の放出が防がれます。組織の抗プロテアーゼ･抗酸化系もさらに毒性を緩衝します。

したがって、健常粘膜では、好中球が迅速な抗菌作用、マクロファージが反応の封じ込めと終了、樹状細胞が必要に応じた適応免疫の起動という分業を行っています。長引く動員、アポトーシス遅延、NET やプロテアーゼの蓄積、エフェロサイトーシス不全が生じたときに初めて、好中球は防御因子から主要な組織障害因子へ転じます。

Q02

質問

健常な腸管粘膜において、好酸球はどのように恒常性の維持に寄与し、マクロファージまたは樹状細胞と情報交換しますか。また、これらの役割は炎症時の好酸球機能とどのように異なりますか。

回答

好酸球は健常腸管の固有層にも比較的多く存在するため、その役割はアレルギーや寄生虫防御だけでは説明できません。恒常状態では CCL11 などの eotaxin 系ケモカインが組織分布を支え、局所の生存因子のもとで比較的低い活性化状態が保たれます。実験研究では、好酸球が上皮維持、腸内細菌叢の調節、粘膜抗体応答に関わることが示されており、IL-6、APRIL 関連シグナル、TGF-β などを介した形質細胞や IgA 応答への関与も提案されています。また、脂質メディエーターや増殖関連因子を通じて血管、細胞外基質、上皮更新に影響し得ます。ただし、個々の恒常性機能の強さには動物種や腸管部位による差があります。

マクロファージとの情報交換は、直接接触より可溶性因子を介することが多いと考えられます。好酸球由来の IL-4、IL-13 は、マクロファージに貪食、マトリックス再構築、組織修復に関連する機能状態を誘導し得ます。一方、常在マクロファージは局所サイトカインを調整し、アポトーシス細胞を除去し、上皮由来アラーミンの過剰な持続を抑えることで好酸球活性化を制限します。樹状細胞は、炎症時には好酸球由来サイトカインや顆粒分子の影響を受け、たとえば eosinophil-derived neurotoxin は実験系でアラーミンとして樹状細胞活性化を増強します。好酸球自身も条件次第で MHC-II などを発現しますが、健常ヒト腸管で主要な抗原提示細胞として働くという証拠は限定的です。

炎症になると同じ細胞の出力が質的に変化します。上皮由来 IL-33、IL-25、TSLP、さらに ILC2 や Th2 細胞由来 IL-5 と eotaxin の増加により、好酸球数、寿命、脱顆粒能が上昇します。活性化好酸球は major basic protein、eosinophil peroxidase、陽性荷電顆粒蛋白、活性酸素、システイニルロイコトリエン、炎症性サイトカインを放出し、上皮障害、透過性亢進、神経刺激、マクロファージ･樹状細胞の追加活性化を引き起こします。IL-4/IL-13 や TGF-β が長期間持続すると、本来の修復反応が異常な線維化へ移行することもあります。

したがって、恒常状態では低強度で局在した栄養･調節作用が中心であるのに対し、炎症時には細胞数増加、長寿命化、顆粒毒性、2 型サイトカイン回路の増幅が前景化します。ただし両者は完全な二分ではなく、感染後の修復などで好酸球が有益に働く場合もあり、最終的な作用は組織環境と刺激の強さ･持続時間で決まります。

Q03

質問

健常な気道粘膜および急性の微生物刺激時に、好中球は肺胞または間質マクロファージ、樹状細胞とどのように協調して宿主防御を担いながら、上皮への二次的損傷を抑えますか。

回答

健常な呼吸器粘膜では、遠位気腔の第一線の監視役は肺胞マクロファージであり、間質マクロファージと樹状細胞が異なる組織ニッチを補完します。平時には吸入粒子や少量の微生物を除去しながら、過剰な炎症を起こさないよう抑制された状態にあります。明確な感染が TLR や NOD 様受容体などで認識されると、マクロファージ、上皮細胞、その他の局所細胞が IL-1、TNF、CXCL 系ケモカイン、GM-CSF、脂質性走化因子を産生し、好中球を短時間に感染部位へ集積させます。好中球は貪食、NADPH オキシダーゼによる呼吸バースト、MPO、顆粒蛋白を用いて殺菌し、高い微生物負荷では NET 形成も利用し得ます。

この協調は時間依存的です。初期にはマクロファージが好中球を呼び込み、続いて好中球によって処理された微生物断片、IL-1 系シグナル、酸化脂質、DAMP がマクロファージを再刺激します。感染が制御されていない間は有用な増幅ですが、病原体量が低下した後も続けば上皮への副次的損傷になります。樹状細胞は抗原を取り込み、所属リンパ節へ移動して病原体特異的 T 細胞応答を形成するため、後期の防御を最大強度の好中球炎症だけに依存させずに済みます。

上皮障害を抑えるには能動的な炎症収束が必要です。役割を終えた好中球はアポトーシスし、肺胞･間質マクロファージにエフェロサイトーシスされます。これによりマクロファージの NF-κB 型炎症出力が低下し、IL-10、TGF-β、Annexin A1、リポキシンやレゾルビンなどが増えます。気道表面液中の α1-antitrypsin や SLPI などは好中球エラスターゼを抑え、抗酸化系は MPO/ROS による障害を緩衝し、DNase は過剰な DNA-rich NET の除去に寄与します。

したがって、重要なのは好中球の有無ではなく、動員が短時間で局所化され、終了後に確実に除去されるかです。急性細菌･真菌感染では好中球は不可欠ですが、重症肺炎、COPD、ある種の喘息では CXCL8/GM-CSF の持続、アポトーシス遅延、マクロファージのエフェロサイトーシス不全、NET･プロテアーゼ過剰が、線毛、タイトジャンクション、肺胞上皮の障害へつながります。

Q04

質問

恒常状態および 2 型炎症時の気道粘膜において、好酸球はマクロファージや樹状細胞とどのように相互作用しますか。

回答

恒常状態の気道では好酸球は比較的少なく、肺胞マクロファージと樹状細胞は吸入抗原に継続的に接していても、上皮から強い危険シグナルが出ない限り低反応･低共刺激の状態を保ちます。マクロファージは粒子やアポトーシス細胞の除去、樹状細胞は抗原サンプリングを担い、IL-10、TGF-β、健全な上皮バリアが好酸球の過剰な動員と活性化を抑えています。

2 型炎症ではまず上流のアラームが変化します。アレルゲンや上皮障害により TSLP、IL-33、IL-25 が放出され、ILC2 と樹状細胞が活性化されます。樹状細胞は OX40L などの共刺激プログラムを高め、リンパ節で Th2 分化を促進します。ILC2/Th2 細胞由来の IL-5 は好酸球の生存を延長し、IL-4/IL-13 は上皮や間質細胞に CCL11、CCL24、ヒトでは特に CCL26 などの eotaxin を誘導して、好酸球を選択的に気道へ集積させます。

集積した好酸球はマクロファージと樹状細胞へフィードバックします。IL-4/IL-13 はマクロファージに修復関連機能を誘導し、短期的にはバリア再建に役立ちますが、持続すると粘液過剰、細胞外基質沈着、線維化に寄与し得ます。好酸球顆粒蛋白、ロイコトリエン、活性酸素、サイトカインは上皮を障害し、新たな危険シグナルを生み、樹状細胞活性化を増強します。eosinophil-derived neurotoxin などの顆粒由来リボヌクレアーゼが実験系で樹状細胞を刺激することも報告されています。逆にマクロファージがアポトーシス好酸球を効率よく除去すれば、IL-10 と炎症収束性メディエーターが増えます。

したがって、恒常状態と 2 型炎症の違いは細胞の種類というより、同じネットワークにかかるシグナルの強度と持続時間です。短期間の好酸球—マクロファージ相互作用は修復に有益ですが、IL-5/eotaxin による持続動員、反復脱顆粒、長期の IL-4/IL-13 は慢性炎症とリモデリングへ変換します。上皮アラーミンと樹状細胞の中心的役割は比較的確立していますが、ヒトでの直接的な好酸球—樹状細胞相互作用の定量的寄与はより状況依存的です。

Q05

質問

尿路粘膜における初期の抗菌防御で、好中球は単球またはマクロファージ、樹状細胞とどのように協調しますか。また、過剰な組織障害を防ぐ機序は何ですか。

回答

尿路粘膜の早期抗菌防御は、主として尿路上皮と局所の自然免疫細胞から始まります。尿路病原菌が膀胱や下部尿路の上皮に接触すると、TLR などのパターン認識経路を介して IL-1、TNF、好中球走化性ケモカインが誘導されます。常在マクロファージはこの警報を増幅し、局所血管の活性化を助け、感染が強い場合には CCR2 依存性単球が流入してマクロファージ集団を補います。好中球は数時間以内に大量に動員され、上皮を通過して尿腔側まで移動し、貪食、ROS、抗菌顆粒、強い刺激下では NET によって細菌量を急速に低下させます。

単球･マクロファージは好中球と同じ仕事をするだけではありません。細菌と組織残骸を貪食し、感染が続く間は適度なサイトカイン･ケモカイン産生を維持し、その後はアポトーシス好中球を除去して炎症終了へ導きます。樹状細胞は抗原サンプリングと適応免疫の起動を担いますが、単純な急性 UTI の最初の数時間では、上皮—マクロファージ—好中球軸ほど支配的ではありません。再発性･複雑性 UTI では、T 細胞や B 細胞応答の質に対する樹状細胞の意義が大きくなります。

抗菌機構そのものが尿路上皮障害の原因にもなります。好中球エラスターゼ、MPO 由来酸化物、ROS、NET 関連ヒストンはタイトジャンクションを傷害し、深部組織を露出させます。通常は細菌が除去されると走化シグナルが速やかに低下し、好中球はアポト

ーシスしてマクロファージにエフェロサイトーシスされます。これによりマクロファージは追加の好中球募集シグナルを減らし、IL-10、TGF-β、修復プログラムを増加させます。局所プロスタグランジン、炎症収束性脂質、抗酸化･抗プロテアーゼ系、さらに尿路上皮の高い再生能も副次的損傷を抑えます。

したがって、尿路防御は速度と制御の均衡です。好中球動員が弱すぎれば細菌増殖や上行感染を許し、病原体が減った後も動員が続けばバリア障害と症候性炎症が残ります。細かな分子回路の多くは UTI 動物モデルで強く支持されていますが、ヒトでのマクロファージ亜集団や NET、個々のケモカインの寄与はより不均一です。

Q06

質問

膀胱または下部泌尿生殖器粘膜において、肥満細胞由来メディエーターはマクロファージや樹状細胞の機能にどのような影響を与えますか。また、このネットワークは宿主防御と炎症症状の双方にどのように関与し得ますか。

回答

肥満細胞は膀胱や下部泌尿生殖器粘膜の血管、神経、上皮の近くに位置するため、少数の細胞の活性化でも周囲の髄系ネットワークを大きく変化させます。微生物産物、補体、IgE/FcεRI、上皮アラーミン、神経ペプチドなどにより活性化されると、ヒスタミン、tryptase、chymase、TNF を速やかに放出し、続いて PGD2、システイニルロイコトリエン、各種サイトカイン、増殖因子を産生します。これにより血流と血管透過性が上昇し、上皮シグナルが変化し、他の白血球が組織へ入りやすくなります。

マクロファージと樹状細胞は多くの肥満細胞メディエーターに対する受容体を持ちます。TNF や GM-CSF は髄系細胞の活性化･生存を増強し、ヒスタミンは受容体サブタイプと細胞状態によって炎症性サイトカインを増強も抑制もします。tryptase などのプロテアーゼは PAR2 を介して上皮や髄系細胞に作用します。IL-4/IL-13 はマクロファージを修復関連状態へ寄せ、TNF/IL-1 が優位ならより炎症性の反応が持続します。感染時には、これらの作用が好中球動員を加速し、樹状細胞の成熟･移動･抗原提示を高めるため、宿主防御に有利になり得ます。

同じ回路は炎症症状も生みます。ヒスタミン、tryptase、プロスタグランジン、NGF 関連シグナルは膀胱知覚神経を感作し、TRPV1 や P2X 系の興奮閾値を下げます。血管漏出は浮腫を生じ、プロテアーゼ、TNF、他の髄系細胞由来 ROS は尿路上皮バリアを弱めます。バリアが破綻すると尿中溶質や ATP が粘膜下神経終末へ届きやすくなり、尿意切迫、疼痛、神経原性炎症の正のフィードバックが形成されます。マクロファージ由来 IL-1β、TNF、PGE2 も神経をさらに感作します。

炎症収束には、マクロファージのエフェロサイトーシス、IL-10、TGF-β、炎症収束性脂質メディエーター、上皮再生が必要です。バリアが回復すれば感覚神経への刺激が減り、肥満細胞活性化も低下します。ただし慢性膀胱痛症候群などは非常に不均一で、肥満細胞増加がすべての患者に共通するわけではありません。この回路は宿主防御と症状をつなぐ有力な機序ですが、万能な単一病因ではありません。

Q07

質問

急性の腸管粘膜炎症とその後の炎症収束過程において、好中球とマクロファージは、遊走･動員シグナル、サイトカイン、エフェロサイトーシス、脂質メディエーター、組織修復プログラムを介してどのように相互作用しますか。

回答

急性腸管粘膜炎症の初期には、好中球とマクロファージが急速な増幅回路を形成します。バリア破綻により上皮細胞と常在マクロファージが IL-1、TNF、CXCL 系ケモカイン、C5a、LTB4 などを産生し、まず好中球を病変へ呼び込みます。好中球は微生物を殺す一方、ROS、プロテアーゼ、炎症性サイトカイン、LTB4 を放出し、さらに好中球や単球を誘導します。CCR2 依存性単球が流入して炎症性マクロファージ状態へ分化すると、危険シグナルが続く間は TNF、IL-1β、IL-6、ケモカイン産生が維持されます。

炎症収束は単なるサイトカイン量の低下ではなく、シグナルの質的転換です。役割を終えた好中球はアポトーシスし、ホスファチジルセリンなどの「eat-me」シグナルを提示します。マクロファージは MerTK、Axl、TIM 系受容体、インテグリンと架橋分子などを介してエフェロサイトーシスを行います。アポトーシス好中球を取り込むと、NF-κB 型の炎症性出力が抑えられ、IL-10、TGF-β、組織修復に適した代謝プログラムが誘導されます。さらに、二次性壊死によるヒストン、エラスターゼ、MPO などの大量放出を防ぐことができます。

脂質メディエーターにも「クラススイッチ」が起こります。初期のプロスタグランジンやロイコトリエンは血管活性化･白血球動員を助けますが、収束期にはリポキシン、レゾルビン、プロテクチン、マレシンが新規好中球流入を抑え、マクロファージのエフェロサイトーシスを高めます。好中球自身もこの脂質変換に関与し得ます。死細胞を除去したマクロファージは TGF-β、amphiregulin、VEGF 系因子、基質再構築酵素などを産生し、上皮幹･前駆細胞や間質細胞とともにバリア再建を進めます。

この順序が崩れると炎症は持続します。過剰な NET、プロテアーゼ、ROS は上皮障害を拡大し、DAMP が新たな髄系細胞を呼び込みます。エフェロサイトーシス不全では死細胞が壊死し、炎症性内容物が再放出されます。一方で好中球を早すぎる段階で抑えれば微生物排除が不十分になります。したがって、単純な M1/M2 二分ではなく、殺菌、アポトーシス、エフェロサイトーシス、脂質メディエータースイッチ、修復が正しい時間順序で連結されることが重要です。

Q08

質問

好酸球由来メディエーターは、腸管粘膜のマクロファージの機能状態や樹状細胞の活性化をどのように変化させますか。また、これらの相互作用は免疫寛容、組織修復、炎症のバランスをどのように変え得ますか。

回答

好酸球由来のサイトカイン、脂質メディエーター、顆粒蛋白は、腸管マクロファージと樹状細胞の機能状態を変化させ得ますが、その結果は恒常性、修復、強い炎症のどの状況にあるかで大きく異なります。比較的低い活性化状態では、好酸球由来 IL-4/IL-13 が

マクロファージの貪食、マトリックス再構築、修復関連機能を促進し得ます。TGF-β、脂質メディエーター、増殖関連因子を介して上皮や間質へ作用し、実験系ではバリア回復や IgA 関連恒常性と結びつく場合があります。

樹状細胞に対しては、炎症時の好酸球シグナルがより強く現れます。eosinophil-derived neurotoxin などの顆粒由来リボヌクレアーゼは実験系でアラーミンとして樹状細胞活性化を高め、ロイコトリエン、ROS、炎症性サイトカイン、顆粒蛋白による上皮障害も間接的に危険シグナルの多い環境を作ります。IL-4 は樹状細胞の 2 型免疫寄りの機能を条件づけることがあります。好酸球自身も炎症条件下で MHC-II や共刺激分子を発現し得ますが、ヒト腸管で主要な抗原提示細胞として働くという証拠は、古典的樹状細胞ほど強くありません。

この相互作用は、粘膜を寛容、修復、炎症の三方向へ動かし得ます。制御された好酸球は、食物抗原や共生菌に対する低反応性、IgA、バリア維持を支える可能性があります。損傷後には IL-4/IL-13、TGF-β がマクロファージの残骸処理と再建を助けます。一方、IL-5/eotaxin により好酸球が大量集積し、脱顆粒、システイニルロイコトリエン、ROS、アラーミン放出が続けば、樹状細胞と他のエフェクター細胞がさらに活性化され、修復反応が慢性炎症や線維化へ傾きます。

したがって、固定した「好酸球の型」よりも、組織シグナルの組み合わせ、活性化強度、持続時間が重要です。IL-33、IL-25、TSLP の持続は 2 型炎症増幅に傾き、好酸球のアポトーシスとマクロファージによる除去は収束を促します。恒常性における個々の好酸球機能の一部はマウスで強く示されていますが、ヒトでの定量的寄与には慎重な解釈が必要です。

Q09

質問

好中球性気道炎症において、好中球とマクロファージは、サイトカイン、脂質メディエーター、好中球細胞外トラップ、エフェロサイトーシス、代謝シグナルを介してどのように相互作用しますか。また、そのクロストークは炎症の持続と収束をどのように左右しますか。

回答

好中球性気道炎症が遷延するのは、マクロファージと好中球が互いの動員･生存シグナルを繰り返し作り出すためです。活性化した気道マクロファージ、上皮細胞、樹状細胞は IL-1β、TNF、CXCL8/CXCL 系ケモカイン、GM-CSF を産生し、好中球を呼び込みながら寿命も延長します。好中球は ROS、エラスターゼ、MPO、炎症性サイトカイン、LTB4 を放出し、マクロファージ活性化と上皮 DAMP 産生をさらに促進します。特に LTB4 は好中球同士の連鎖的動員を増幅し、GM-CSF は本来早期に起こるアポトーシスを遅らせます。

NET はこの回路を強く増幅します。DNA 網上にヒストン、エラスターゼ、MPO が保持され、微生物捕捉には有用ですが、過剰または除去不全の NET は上皮を直接障害し、マクロファージへ核酸･酸化蛋白の危険シグナルを与えます。TLR9、cGAS-STING、NLRP3 などが関与し得ますが、喘息、COPD、感染症で相対的な寄与は同一ではありません。マクロファージからさらに IL-1β とケモカインが出れば、「NET 増加→好中球増加→さらに NET」という正のフィードバックになります。

代謝状態も持続性を左右します。炎症性マクロファージは糖解系を高め、succinate/HIF-1α と IL-1β に結びつく代謝状態をとることがあります。好中球は移動や NADPH 依存性 ROS 産生のため糖解系とペントースリン酸経路に強く依存します。低酸素、細胞外 ATP、乳酸、アデノシンは寿命とサイトカイン出力を変えます。急性感染では有利な適応でも、低酸素と粘液貯留が続く慢性気道では炎症維持因子になり得ます。

収束の中心は好中球アポトーシスとマクロファージによるエフェロサイトーシスです。MerTK などを介した貪食後、マクロファージは IL-10、TGF-β、炎症収束性脂質を増やし、修復へ代謝転換します。リポキシンやレゾルビンは新たな好中球動員も抑えます。エフェロサイトーシスが障害されると二次性壊死が起こり、プロテアーゼと DAMP が再放出されます。したがって持続炎症は、動員停止、NET/残骸除去、代謝の沈静化、エフェロサイトーシスの複数段階が同時に失敗した状態と考えるのが適切です。

Q10

質問

アレルギー性気道炎症において、肥満細胞と好酸球、さらに該当する場合には好塩基球は、樹状細胞およびマクロファージとどのように相互作用して 2 型免疫を増幅または抑制しますか。

回答

アレルギー性気道炎症は、上皮アラーミン、樹状細胞、2 型エフェクター細胞の相互増幅で成立します。アレルゲンや上皮ストレスにより TSLP、IL-33、IL-25 が放出され、ILC2 と樹状細胞が活性化されます。古典的樹状細胞は OX40L などの共刺激プログラムを高め、リンパ節でアレルゲン特異的 Th2 応答を誘導します。Th2 と ILC2 は IL-4、IL-5、IL-13 を供給し、肥満細胞、好酸球、状況によって好塩基球が強い増幅因子として機能できる環境を作ります。

IgE/FcεRI が架橋された肥満細胞は、ヒスタミン、tryptase、PGD2、システイニルロイコトリエン、TNF、2 型サイトカインを迅速に放出します。PGD2-CRTH2 系とロイコトリエンは好酸球、ILC2、Th2 をさらに呼び込みます。IL-5 は好酸球生存を延長し、IL-4/IL-13 は上皮の eotaxin 産生を増やします。好酸球はロイコトリエン、サイトカイン、顆粒蛋白、ROS を放出して上皮を傷害し、新たなアラーミン産生を促します。好塩基球は早期 IL-4 の供給源となり得ますが、ヒト気道で古典的樹状細胞に代わる主要抗原提示細胞とみなす根拠は限定的です。

マクロファージは増幅にも抑制にも働きます。IL-4Rα シグナルは修復関連機能を誘導し、短期的には上皮回復を助けますが、慢性的には粘液過剰、基質沈着、リモデリングへ寄与します。CCL17/CCL22 などを介して 2 型細胞の集積を支える場合もあります。一方、アポトーシスした好酸球を効率よく除去すると IL-10、TGF-β、炎症収束性脂質が増え、追加動員が抑えられます。上皮の危険シグナルが低下すれば樹状細胞も低共刺激状態へ戻りやすくなります。

したがって、増幅か抑制かを決めるのは、抗原･アラーミン駆動が継続するかどうかです。持続的なアレルゲン曝露、バリア障害、IgE 活性化、IL-5/eotaxin は自己維持的 2 型炎症を促し、抗原低下、好酸球アポトーシス、マクロファージのエフェロサイトーシス、

上皮修復は収束を促します。このため IgE や主要 2 型サイトカインの阻害は、一種類の顆粒球だけでなくネットワーク全体を変化させます。

Q11
質問
急性細菌性尿路感染において、動員された好中球と単球由来マクロファージは、病原体排除、サイトカイン産生、エフェロサイトーシス、粘膜修復の過程でどのように協調または競合しますか。

回答
急性細菌性尿路感染では、好中球と単球由来マクロファージは異なる時間スケールで動員されるため、機能は補完的です。尿路上皮と常在髄系細胞が細菌を認識すると CXCL 系ケモカイン、IL-1、TNF などが産生され、好中球が最も早く大量に流入します。好中球は尿路上皮を通過して尿中にも現れ、オプソニン化細菌の貪食、NADPH オキシダーゼ依存性 ROS、抗菌顆粒、必要に応じた NET により短時間で強い殺菌圧をかけます。

CCR2 依存性単球は一般にやや遅れて流入し、炎症性マクロファージ状態へ分化します。これらも細菌を貪食しますが、特に重要なのはサイトカイン環境の維持･再構成、死細胞除去、修復への移行です。感染初期には TNF、IL-1β、ケモカインで好中球動員を支え、細菌量が低下すると同じマクロファージ集団がエフェロサイトーシスを行い、IL-10、TGF-β、修復因子を増加させます。アポトーシス好中球の除去は単なる清掃ではなく、炎症から修復へのスイッチです。

最も効率的な協調は、好中球が迅速な殺菌、マクロファージが持続的な監視･残骸処理･収束を担う場合です。ただし、オプソニン化細菌、補体、局所酸素や栄養をめぐって競合する可能性があります。マクロファージが細菌を取り込んでも完全に殺せない場合、一時的な細胞内生存部位になる可能性もあります。逆に、過剰な好中球 ROS やプロテアーゼは周囲のマクロファージを傷害し、尿路上皮を破綻させ、新たな細菌付着面を作り得ます。

粘膜修復には関係性の時間的転換が必要です。走化シグナルが低下し、好中球が退出し、マクロファージがエフェロサイトーシス、基質再構築、上皮再生支援を行います。好中球を早く抑えすぎれば殺菌不十分となり、逆に病原体が制御された後まで残れば NET、エラスターゼ、MPO、ROS が症状と損傷を長引かせます。この時間的分業は実験モデルで強く支持されますが、ヒト UTI における各マクロファージ亜集団や細胞内細菌リザーバーの重要性は症例により異なります。

Q12
質問
慢性膀胱粘膜炎症において、肥満細胞とマクロファージのクロストークは、感覚神経シグナル、血管透過性、上皮バリア機能、そして炎症の持続または収束をどのように変化させ得ますか。

回答
慢性膀胱粘膜炎症では、肥満細胞とマクロファージが神経･血管･上皮をつなぐ正のフィードバック回路を形成し得ます。肥満細胞は知覚神経と微小血管の近くにあり、神経ペプチド、補体、上皮由来シグナルなどで活性化されると、ヒスタミン、tryptase、プロスタグランジン、ロイコトリエン、TNF、増殖因子を放出します。ヒスタミンとプロテアーゼは血管透過性を高めるだけでなく、H1/H4 受容体や PAR2 を介して知覚神経の閾値を下げます。NGF 関連シグナルも疼痛や尿意切迫の過敏化に寄与します。

マクロファージが炎症状態を維持すると、この神経感作はさらに増幅されます。IL-1β、TNF、PGE2、ROS は侵害受容神経を感作し、血管内皮活性化を持続させます。活性化神経は substance P や CGRP を放出し、肥満細胞、血管、マクロファージへ逆方向に作用します。CCL2/CCR2、CSF1 関連シグナルは単球･マクロファージを持続的に補充･維持し、SCF/c-Kit などは局所肥満細胞の生存を支えるため、明確な感染がなくても炎症が自己維持される可能性があります。

上皮バリア障害が神経と炎症を結びつけます。肥満細胞プロテアーゼ、マクロファージサイトカイン、他の白血球由来 ROS がタイトジャンクションを障害すると、尿中成分が粘膜下へ到達しやすくなります。傷害上皮は ATP と DAMP を放出し、P2X などのプリン作動性受容体を介して知覚終末を刺激します。血管漏出による浮腫も局所圧を高め、疼痛･尿意切迫を悪化させます。こうして「バリア障害—神経過敏—肥満細胞/マクロファージ活性化」のループが形成されます。

収束には、マクロファージがエフェロサイトーシス、IL-10、TGF-β、炎症収束性脂質メディエーター、上皮再生へ移行する必要があります。バリアが回復すると尿中刺激物から神経が隔離され、肥満細胞刺激も低下します。ただし TGF-β 型修復が過剰なら線維化や膀胱コンプライアンス低下が新たな症状源になります。慢性膀胱痛症候群は不均一で、肥満細胞が全症例の中心機序というわけではありません。

Q13
質問
潰瘍性大腸炎において、好中球の動員、好中球細胞外トラップ形成、好中球代謝、ならびに好中球–マクロファージ間クロストークは、粘膜障害と炎症収束にそれぞれどのように寄与しますか。

回答
潰瘍性大腸炎では、好中球は抗微生物防御に必要である一方、活動性粘膜障害の主要な実行細胞でもあります。炎症上皮、間質細胞、単球由来マクロファージは CXCL8 をはじめとする CXCR1/2 リガンド、IL-1、TNF、コロニー刺激因子を産生し、大量の好中球を結腸粘膜へ動員します。好中球が上皮を通過して陰窩へ集積することで、陰窩炎や陰窩膿瘍が形成されます。貪食と ROS は破綻したバリアを越えた細菌の制御に役立ちますが、細胞外エラスターゼ、MPO 由来酸化物、MMP、ROS は上皮びらんを拡大します。

NET は別の障害経路を加えます。DNA･ヒストン骨格は微生物捕捉に有用ですが、持続する NET はエラスターゼと MPO を保持し、上皮膜を傷害し、炎症･凝固シグナルを強め、マクロファージに DAMP を供給します。TLR9、cGAS-STING、NLRP3 などの核酸感知･inflammasome 経路がモデルによって関与しますが、ヒト UC でどの経路が支配的かは確定していません。臨床検体では NET

指標と活動性の相関がしばしば認められますが、NET が主要な因果ドライバーか、強い好中球炎症のマーカーかは完全には分離されていません。

好中球代謝も持続性に関係します。好中球は糖解系とペントースリン酸経路に強く依存し、後者は NADPH を供給して酸化バーストを支えます。炎症粘膜の低酸素環境は HIF 関連生存シグナルを安定化させ、アポトーシスを遅らせ得ます。乳酸、succinate、細胞外 ATP なども好中球とマクロファージ双方の活性を変えます。急性防御のための代謝適応が、慢性病変では長寿命化と毒性出力維持につながります。

炎症収束にはマクロファージのエフェロサイトーシスが不可欠です。MerTK などでアポトーシス好中球を取り込むと、IL-10、TGF-β、リポキシン、レゾルビン、修復プログラムが増えます。除去に失敗すると二次性壊死と DAMP 放出が続きます。したがって治療上の理想は好中球を全面的に排除することではなく、過剰な動員と細胞外毒性を抑えながら貪食殺菌を保ち、アポトーシス、エフェロサイトーシス、バリア再建を加速することです。

Q14

質問

クローン病において、好中球、単球由来マクロファージ、樹状細胞の相互作用の異常は、微生物処理、抗原提示、炎症の持続、上皮修復をどのように変化させますか。

回答

クローン病では、通常なら「微生物認識—排除—抗原処理—炎症終了—上皮修復」まで完結するはずの流れが途中で止まると考えると理解しやすくなります。バリア障害により腸内微生物が固有層へ侵入し、好中球が動員されますが、NOD2、ATG16L1 など微生物認識、自食作用、細胞内処理に関わる感受性経路の異常があると、微生物成分が十分に処理されず、髄系細胞刺激が持続し得ます。

CCR2 依存性単球は持続的に組織へ流入し、健常腸管の常在マクロファージに特徴的な「高い貪食能と低い炎症性サイトカイン出力」を獲得できず、TNF、IL-1β、IL-6、IL-23、ケモカインを産生し続けることがあります。好中球の ROS、プロテアーゼ、NET は微生物制御に寄与しつつ、上皮障害と DAMP 放出を増幅します。肉芽腫形成では、処理しにくい微生物･粒子を抱えたマクロファージの長期残存も局所炎症維持に寄与し得ます。

樹状細胞はこの自然免疫炎症を適応免疫へ接続します。持続する微生物抗原、IL-12/IL-23 系シグナル、強い共刺激は、粘膜寛容より Th1/Th17 関連応答を促します。活性化 T 細胞が腸管へ戻り、IFN-γ、IL-17 などを産生すると、マクロファージと上皮のケモカイン産生がさらに増加します。その結果、「微生物処理不全→抗原曝露増加→適応免疫炎症増強→バリア障害→さらに微生物侵入」という循環が成立します。

修復にはマクロファージのエフェロサイトーシス、amphiregulin や TGF-β などの増殖･修復シグナル、上皮幹細胞の再生、制御された細胞外基質再構築が必要です。持続する TNF/IL-1、プロテアーゼ、低酸素はこれを阻害し、逆に TGF-β 型修復が過剰なら狭窄･線維化が進みます。抗 TNF 療法の有効性は炎症性髄系サイトカインの因果的役割を支持しますが、クローン病は不均一で、自然免疫の殺菌不全、単球/マクロファージ炎症、樹状細胞抗原提示、線維化の比重は患者ごとに異なります。

Q15

質問

重症好中球性喘息において、好中球、マクロファージ、樹状細胞はどのように自己持続的な炎症回路を形成しますか。また、好中球細胞外トラップ、インフラマソームシグナル、微生物由来シグナル、ステロイド抵抗性はどのような役割を果たし得ますか。

回答

重症好中球性喘息では、典型的な好酸球性 2 型回路よりも、「上皮—マクロファージ/樹状細胞—Th17—好中球」の自己持続回路が前景化することがあります。汚染物質、感染、DAMP、気道細菌叢由来シグナルが TLR/NOD、NF-κB を活性化し、上皮とマクロファージから IL-1β、TNF、CXCL8、GM-CSF が産生されます。樹状細胞･マクロファージは IL-6/IL-23 環境を通じて Th17 を支持し、IL-17 は上皮の CXCL8、G-CSF を増加させ、好中球の動員と生存を強化します。好中球エラスターゼ、ROS、DAMP が再び上皮とマクロファージを刺激するため、回路が閉じます。

NET はこの回路をさらに強化します。細胞外 DNA、ヒストン、MPO、エラスターゼは粘液粘稠度を高め、上皮を傷害し、マクロファージや樹状細胞の自然免疫センサーを刺激します。複数のモデルでは NET 成分や細胞外 ATP が NLRP3 inflammasome と IL-1β を促進し、好中球･Th17 炎症をさらに増幅します。ただし NLRP3 の重要性は全患者で均一ではなく、「好中球性喘息」には感染関連、喫煙･汚染関連、肥満関連、治療によって形成された複数のエンドタイプが含まれます。

微生物シグナルが持続性の外因性ドライバーになる場合もあります。慢性細菌定着、反復感染、細菌叢変化は TLR リガンドを継続的に供給し、IL-1/CXCL8 軸を維持します。マクロライドが一部の患者で有効なのは抗菌作用に加えて好中球走化やマクロファージ炎症出力を下げるためかもしれませんが、耐性化や細菌叢撹乱という代償があります。感染優位の患者で過剰に好中球を抑えることは危険です。

ステロイド抵抗性も多因子性です。好中球/Th17 炎症は一般に好酸球性 2 型炎症よりステロイド反応性が低く、酸化ストレスや p38 MAPK 系はグルココルチコイド受容体機能を損なう可能性があります。ステロイド自体が好中球アポトーシスを遅らせることもあります。HDAC2 低下も候補機序ですが、喘息での寄与は一様ではありません。したがって、NET、inflammasome、微生物刺激、動員、収束不全を同時に評価し、抗菌防御を保ちながら回路を切る必要があります。

Q16

質問

好酸球性喘息において、好酸球、肥満細胞、さらに該当する場合には好塩基球は、マクロファージ、樹状細胞、気道上皮とどのように相互作用して 2 型炎症を開始･増幅･収束させますか。

回答

好酸球性喘息の開始点は、多くの場合気道上皮です。アレルゲン、ウイルス、プロテアーゼ、汚染物質が IL-33、TSLP、IL-25 の放出を誘導し、ILC2 と古典的樹状細胞を活性化します。樹状細胞はリンパ節へ移動してアレルゲン特異的 Th2 を誘導し、Th2 と ILC2 は IL-5 により好酸球を増殖･維持します。IL-4/IL-13 は上皮の eotaxin、粘液、2 型関連ケモカインを増加させ、肥満細胞と好塩基球も IgE およびサイトカイン依存性経路で同じネットワークに入ります。

増幅期には各細胞が相互にシグナルを供給します。IgE で架橋された肥満細胞はヒスタミン、PGD2、システイニルロイコトリエンを放出し、気管支収縮を起こすと同時に、CRTH2 などを介して好酸球、ILC2、Th2 をさらに集めます。好塩基球は迅速な IL-4 供給源となり得ますが、主要な専門抗原提示細胞は樹状細胞です。好酸球はロイコトリエン、IL-4/IL-13、顆粒蛋白、ROS を放出し、上皮傷害と追加アラーミンを生みます。IL-4Rα シグナルを受けたマクロファージは修復･再構築に関連する状態へ移行します。

上皮は単なる標的ではありません。IL-13 は杯細胞化生と MUC5AC などの粘液遺伝子を誘導し、上皮由来 eotaxin は好酸球の局在を規定します。損傷が続けば TSLP/IL-33 が樹状細胞と ILC2 を持続的に刺激します。慢性期には好酸球･肥満細胞由来 TGF-β、プロテアーゼ、脂質メディエーターが平滑筋変化や上皮下線維化を促し、反復性炎症が構造的リモデリングへ転化します。

収束には、抗原･アラーミン刺激の低下、好酸球アポトーシス、マクロファージによる効率的エフェロサイトーシスが必要です。これにより IL-10 と炎症収束性脂質が増え、制御性 T 細胞と低活性樹状細胞が新たな Th2 誘導を抑えます。各ノードが密接に結びつくため、上流サイトカインを一つ遮断するだけでも、好酸球移動、肥満細胞活性、マクロファージ状態、上皮機能が同時に変化します。

Q17

質問

反復性尿路感染において、過去の感染は、訓練免疫、免疫寛容、または組織リモデリングを介して膀胱粘膜の好中球および単球 / マクロファージ応答をどのように再構成し得ますか。また、それぞれの機序を支持するエビデンスはどの程度確立されていますか。

回答

既往の尿路感染は、訓練免疫、免疫寛容、組織リモデリングという異なる三つの機序を通じて、次回感染時の好中球･単球/マクロファージ応答を変え得ます。これらは同一ではなく、二次応答に対して反対の予測を与える場合もあります。訓練免疫では、長寿命の骨髄系前駆細胞や組織マクロファージに、炎症･抗菌遺伝子のクロマチン開放性、糖解系、メバロン酸/ミトコンドリア代謝などの持続的変化が生じ、再刺激時にケモカイン産生、貪食、好中球動員が速くなると考えられます。成熟好中球は短寿命なので、長期記憶は同じ好中球自身よりも前駆細胞や膀胱環境によって再現される可能性が高いです。

免疫寛容は逆方向です。反復 TLR/LPS 刺激は TNF、IL-1、ケモカイン遺伝子の一部を低反応化させる一方、特定の抗菌･修復機能を残すことがあります。膀胱では二次感染時の組織障害を軽減する可能性がありますが、好中球動員が弱くなりすぎれば細菌再増殖を許します。古典的 endotoxin tolerance は他組織でよく確立していますが、再発性 UTI 患者の膀胱髄系細胞で主要機序として直接証明されたわけではありません。

組織リモデリングには比較的強い実験的根拠があります。感染既往により尿路上皮分化、バリア性、間質ケモカイン、局所免疫細胞ニッチが変化し、持続抗原や細胞内細菌リザーバーも基礎炎症閾値を変えます。そのため二次感染がより激しくなる場合も、逆に迅速に制御される場合もあります。個々のマクロファージが自律的に「訓練」されていなくても、細胞数、局在、活性化閾値の変化だけで応答は大きく再構成されます。

エビデンスの強さは不均一です。感染後の上皮･組織リモデリングは動物モデルで比較的確立し、ヒト再発性疾患とも整合します。単球/マクロファージの訓練免疫は他感染で強く確立し、尿路でも十分に考えられますが、ヒト膀胱での直接証拠は少ないです。成熟好中球自身の長期記憶は最も説得力が弱く、寛容も機序的には妥当ながら再発性 UTI での定量的証拠は不足しています。縦断的組織解析、単一細胞エピゲノム、再刺激機能試験が必要です。

Q18

質問

膀胱がんにおいて、腫瘍関連好中球と腫瘍関連マクロファージは粘膜腫瘍微小環境内でどのように相互作用し、抗原提示、T 細胞活性、血管新生、浸潤、免疫療法への反応に影響しますか。

回答

膀胱がんの腫瘍関連マクロファージ（TAM）と腫瘍関連好中球（TAN）は、免疫抑制と組織リモデリングを相互に強化するネットワークを形成し得ます。腫瘍･間質細胞は CCL2、CSF1 系でマクロファージを、CXCL8-CXCR2、G-CSF などで好中球を動員します。腫瘍環境で再プログラムされたマクロファージは IL-12 や有効な抗原提示を低下させ、IL-10、TGF-β、ARG1、PD-L1 を高めることがあります。好中球も ARG1、ROS、プロテアーゼ、NET を介して T 細胞抑制機能を獲得し得ます。これらは固定的な M1/M2、N1/N2 という二分類より連続した機能状態として捉える方が適切です。

両者は複数の段階で T 細胞を抑制します。TAM の IL-10/TGF-β と PD-L1 は T 細胞活性化を低下させ、TAN の ROS とアルギニン枯渇は TCR シグナルや増殖を障害します。さらに Treg や他の抑制性髄系細胞の集積を支え、樹状細胞の成熟･抗原提示が弱ければ、初期の抗腫瘍 T 細胞プライミング自体が不十分になります。一方、MHC-II、共刺激、IL-12 を保つ炎症性マクロファージや適切に活性化された好中球は抗腫瘍免疫を支援できるため、腫瘍微小環境がどちらの状態を選択するかが重要です。

血管新生と浸潤も共同出力です。TAM の VEGF、MMP、TGF-β は血管形成と基質再構築を進め、TAN の MMP9、エラスターゼ、VEGF 関連因子、NET は実験系で浸潤経路形成や循環腫瘍細胞の捕捉を助けます。低酸素は HIF 系を介して両細胞の免疫抑制･血管新生状態をさらに強めます。

治療反応にも影響します。高い好中球/リンパ球比や髄系細胞豊富な腫瘍はしばしば不良予後と相関しますが、単一経路の因果証明ではありません。髄系 PD-L1、抗原提示不全、T 細胞排除はチェックポイント阻害の効果を下げ得ます。ただし膀胱内 BCG は、局所の好中球･マクロファージ活性化自体が抗腫瘍に利用できることを示しています。したがって、理想は髄系細胞の一律除去ではなく、免疫抑制･血管新生･NET 優位状態を選択的に再プログラムし、貪食、抗原提示、尿路抗菌防御を残すことです。

Q19

質問

潰瘍性大腸炎において、病原性の好中球動員または好中球細胞外トラップ関連障害を抑えつつ、抗微生物防御とマクロファージによる修復を可能な限り温存するには、どのような機序的に妥当な戦略が考えられますか。予想される利点、トレードオフ、エビデンスの不足を説明してください。

回答

潰瘍性大腸炎では、好中球の貪食性抗菌作用より、細胞外で組織を傷害する機能を選択的に弱めることが理想です。一つの方法は CXCL8-CXCR1/2 動員軸を部分的または局所的に阻害することです。陰窩への好中球流入、ROS、プロテアーゼ負荷を減らせますが、全身性･強力な CXCR2 阻害は細菌感染時の防御を損なう可能性があります。現時点で UC の確立した標準治療ではなく、結腸局所投与、短期間投与、高 NET/好中球型患者の選択などが理論的に望まれます。

NET 関連障害を狙う方法はさらに選択的です。PAD4 阻害は一部の NETosis に必要なクロマチン脱凝縮を抑えますが、すべての NET が PAD4 依存ではなく、PAD4 には他細胞での機能もあります。DNase は DNA 骨格を分解できますが、エラスターゼ、MPO、ヒストンの活性が残れば毒性物質を組織液へ拡散させる可能性があります。そのため DNase とプロテアーゼ阻害、ヒストン中和、MPO 阻害の併用は機序的により合理的です。ただし過剰な酵素阻害は抗菌力も弱めます。

別の方向は流入を止めるより、正常な炎症収束を加速することです。Annexin A1、リポキシン、レゾルビン系は追加好中球動員を抑え、マクロファージのエフェロサイトーシスを高めます。MerTK 系を介してアポトーシス好中球の除去を促進すれば、感染初期の殺菌は残しつつ、後期の組織毒性を短縮できる可能性があります。マクロファージによる修復と上皮バリア回復を高めることも、持続的な微生物刺激を減らす点で有利ですが、過剰な修復型シグナルは線維化や免疫抑制を起こし得ます。

既存 IBD 治療は TNF、IL-23 関連炎症や上皮ケモカインを下げることで間接的に好中球流入を減らしますが、「好中球の抗菌機能を温存する選択的治療」ではありません。NET、CXCR2、炎症収束促進を直接狙う方法の多くは前臨床または明確な UC 臨床効果が未確立です。将来の試験では好中球数だけでなく、細菌制御、NET 負荷、エフェロサイトーシス、上皮治癒、感染率を同時に評価する必要があります。

Q20

質問

炎症性腸疾患において、腸管マクロファージと顆粒球の代謝的またはエピジェネティックなリプログラミングを粘膜恒常性の回復にどのように利用できるでしょうか。また、効果消失や意図しない免疫抑制をもたらし得る機序は何ですか。

回答

代謝･エピジェネティック再プログラミングの利点は、髄系細胞そのものを除去せず、機能状態を変えられる点です。炎症性腸管マクロファージでは糖解系、mTOR、succinate-HIF-1α などが IL-1β、TNF、ケモカイン産生を支えることがあります。AMPK 活性化、ミトコンドリア酸化代謝の改善、Nrf2/itaconate 関連抗酸化経路の増強、過剰な糖解系の抑制などにより、エフェロサイトーシスと修復へ傾けることが考えられます。BET 蛋白、ヒストンのアセチル化/脱アセチル化、メチル化などを調節すれば、炎症遺伝子のクロマチン可及性や訓練免疫様の持続記憶を変えられる可能性があります。

顆粒球ではより慎重な設計が必要です。好中球は移動、生存、NADPH オキシダーゼ依存性殺菌のため糖解系とペントースリン酸経路に強く依存します。糖解系や NADPH 産生を広く抑えれば ROS による粘膜障害は減っても、細菌防御を同時に損ないます。したがって、貪食胞内殺菌を保ったまま、長すぎる生存、過剰 NET、細胞外脱顆粒を選択的に抑える方が合理的です。HIF 関連持続、特定脂質代謝、NET のクロマチン制御などは候補ですが、IBD では大部分が前臨床段階です。

効果が失われる理由も多いです。腸管には血中単球が継続的に流入するため、局所で再プログラムされたマクロファージは新しい炎症細胞で置き換えられます。TNF、GM-CSF、IL-1、IL-23 などの強いサイトカインは代謝介入を上書きし、腸内細菌由来代謝物は同じ経路を逆方向へ押すことがあります。代謝経路の冗長性、疾患不均一性、炎症･線維化組織への薬剤到達不良も逃避機構になります。

意図しない免疫抑制が最大の問題です。糖解系、mTOR、ROS、クロマチン可及性を過剰に抑えると、貪食、inflammasome、サイトカイン依存性動員、抗原提示まで低下します。TGF-β 型修復を過度に促すと線維化や腫瘍監視低下の懸念もあります。全身性エピジェネティック薬はリンパ球や上皮幹細胞にも作用します。したがって、最も現実的なのは細胞･組織選択的送達、可逆的投与、マクロファージ状態と好中球抗菌機能を同時に監視する戦略です。

Q21
質問
2 型サイトカインシグナルを変化させる生物学的製剤は、単に好酸球数を減らすだけでなく、気道粘膜の好酸球–肥満細胞–マクロファージ–樹状細胞ネットワークをどのように再構成し得ますか。

回答
2 型サイトカインを標的とする生物学的製剤は、単に好酸球数を下げる以上に気道ネットワークを変化させます。IL-5 または IL-5R を阻害すると、好酸球の産生、生存、組織滞留が低下するだけでなく、好酸球由来ロイコトリエン、顆粒蛋白、TGF-β、IL-4/IL-13 などの二次シグナルも減ります。その結果、上皮障害、マクロファージのリモデリング、樹状細胞を刺激する危険環境も弱まります。Benralizumab は IL-5Rα を介した抗体依存性細胞傷害により好塩基球も減少させ得るため、早期 IL-4 供給源の一部も変化します。

IL-4Rα 阻害は IL-4 と IL-13 の両方を遮断するため、さらに広い効果を持ちます。上皮の eotaxin、粘液、2 型炎症関連 NO 出力が低下し、B 細胞の IgE クラススイッチ支援が減り、マクロファージは IL-4/IL-13 で条件づけられた修復･リモデリング状態へ強く偏りにくくなります。樹状細胞周囲のケモカイン環境も変化します。治療初期に末梢血好酸球が一時的に増える場合があるのは、骨髄産生低下より組織移行抑制が先行するためであり、血中細胞数だけでは粘膜ネットワークを評価できないことを示します。

より上流の TSLP 阻害では、樹状細胞活性化、ILC2 刺激、肥満細胞･好塩基球を支える上皮信号が同時に弱まり、好酸球以外の炎症出力も変化します。IL-33/ST2 系も ILC2、肥満細胞、好酸球へ幅広く作用するため有力な機序標的ですが、臨床的な確立度は IL-5、IL-4Rα、TSLP 標的療法ほど高くありません。

これらの変化は上皮バリアを改善し、マクロファージが処理すべき炎症残骸を減らしますが、正常な組織修復や抗寄生虫免疫にも影響し得ます。肥満細胞の生存は主に IL-5 依存ではないため、好酸球を大幅に減らしても肥満細胞ネットワークが残ることがあります。つまり、抗 IL-5 は主に好酸球枝を縮小し、IL-4Rα 阻害は上皮･B 細胞･髄系細胞を広く変え、上流アラーミン阻害は樹状細胞—ILC2—顆粒球ネットワーク全体を再構成します。

Q22
質問
好中球性喘息において、抗菌防御を維持しながら好中球–マクロファージ炎症回路を遮断するために、どのような骨髄系細胞標的戦略が考えられますか。また、提案されている機序のうち、臨床的に確立しているものと前臨床段階にあるものを区別してください。

回答
好中球性喘息で最も難しいのは、気道を傷害する好中球—マクロファージ回路が同時に細菌防御にも必要なことです。そのため、全身性の髄系細胞除去より、部分的･局所的･状態依存的な介入が合理的です。CXCR2 拮抗は CXCL8 系シグナルを遮断し、気道好中球を明確に減らせます。臨床試験でも喀痰好中球低下という標的到達は示されていますが、喘息の臨床転帰改善は一貫せず、感染リスクも懸念されます。したがって機序はヒトで検証されていますが、好中球性喘息の標準治療として確立しているわけではありません。

長期マクロライド、とくに選択された持続性･重症喘息患者での azithromycin は、髄系機能に関係する治療の中では比較的強い臨床エビデンスを持ちます。抗菌作用だけでなく、好中球走化、マクロファージサイトカイン、一部の inflammasome 関連出力を低下させ得ます。ただし増悪抑制のどの程度が抗菌作用と免疫調節作用に由来するかは不明で、耐性化、QT、細菌叢変化が問題です。抗 TSLP などの上流生物学的製剤は重症喘息で臨床的に確立していますが、髄系細胞特異的ではありません。

より選択的な方法の多くは前臨床または初期臨床です。NLRP3/IL-1 経路阻害はマクロファージ—好中球増幅を抑える可能性がありますが、IL-1 を広く抑えると感染防御が低下します。DNase、PAD4 阻害、MPO/エラスターゼ阻害、ヒストン中和などの NET 標的法は細胞外毒性を減らしつつ貪食を残すことを狙いますが、ヒト好中球性喘息で確立した有効性はありません。MerTK を介したエフェロサイトーシス促進やレゾルビンなどの炎症収束促進は、初期抗菌反応を残しながら好中球退出を早める点で魅力的ですが、まだ実験的です。

将来は吸入局所投与、生物学的マーカーによる患者選択、抗菌能の同時監視が重要です。慢性細菌定着型と、感染を伴わない IL-1/Th17 優位型では同じ強度の好中球抑制を行うべきではありません。現時点では、「好中球—マクロファージ回路を特異的に遮断し、かつ抗細菌防御を保つことが実証された」標準治療は存在せず、そこが主要なトランスレーショナル課題です。

Q23
質問
訓練免疫または粘膜ワクチンのアプローチによって、単球 / マクロファージと好中球を有益にリプログラムし、反復性尿路感染を減らすことは可能でしょうか。想定される機序、組織特異的な潜在的リスク、現時点のエビデンスの限界を説明してください。

回答
訓練免疫または粘膜ワクチンは、膀胱を慢性炎症状態にするのではなく、次回感染時の初動を速めることで反復性 UTI を減らせる可能性があります。訓練免疫は、長寿命の単球/マクロファージ前駆細胞や組織マクロファージで起こると考えるのが最も妥当です。NOD や C 型レクチンなどのパターン認識刺激により、ヒストン修飾とクロマチン可及性、糖解系、メバロン酸･ミトコンドリア代謝が持続的に変化し、再感染時にケモカイン産生、貪食、好中球動員が速くなる可能性があります。成熟好中球は短寿命なので、長期的な「好中球訓練」は骨髄前駆細胞や膀胱組織環境を介して再現されると考える方が自然です。

粘膜ワクチンはここに適応免疫の特異性を加えます。樹状細胞による抗原提示から尿路へホーミングする T 細胞、抗体、IgA/IgG 応答が形成され、ワクチンのアジュバントが同時に自然免疫訓練を起こす可能性もあります。経口･舌下の細菌溶解物や多価ワクチン

は一部研究で再発減少シグナルを示していますが、保護が適応免疫、訓練免疫、非特異的反復刺激のどれにどれだけ依存するかは十分に分離されていません。

組織特異的リスクは重要です。膀胱が過度に訓練されれば、低レベルの細菌曝露でも CXCL8、好中球 ROS、NET、疼痛性炎症が過剰になる可能性があります。反復刺激は逆に免疫寛容を誘導し、TNF/IL-1 応答を低下させて細菌持続を許すこともあります。尿路上皮幹細胞や間質の長期リモデリングはバリアを強化する可能性もあれば、線維化、知覚過敏、細胞内細菌リザーバーに有利な環境を作る可能性もあります。交差反応性や細菌叢変化も監視対象です。

現在のエビデンスは「概念の妥当性」には比較的強いものの、ヒト膀胱の髄系再プログラミングを直接証明する段階には達していません。再発性 UTI の免疫予防には臨床的な有望シグナルがありますが、製剤、試験デザイン、規制状況、評価項目が不均一です。ヒト膀胱マクロファージの保護的エピジェネティック状態、骨髄レベルの好中球前駆細胞再プログラミング、それらと再発減少の因果関係を示す研究は乏しく、単一細胞解析と再刺激機能試験を組み込んだ臨床研究が必要です。

Q24

質問

腫瘍関連マクロファージと好中球を選択的にリプログラムすることで、保護的な尿路粘膜免疫への影響を最小限にしつつ、膀胱がんの抗腫瘍免疫をどのように高められるでしょうか。機序的アプローチと主要なトランスレーショナル上の障壁を比較してください。

回答

膀胱がんで TAM と TAN を選択的に再プログラムする目的は、髄系細胞を一律に除去することではなく、免疫抑制･血管新生･浸潤促進状態から、抗原提示と T 細胞殺傷を支援する状態へ移行させつつ、通常の尿路抗菌防御を残すことです。マクロファージでは、単純な枯渇より機能活性化が有力です。CD40、TLR、STING の刺激、PI3Kγ 様の抑制シグナル阻害、CD47-SIRPα 貪食チェックポイント遮断などにより、腫瘍細胞貪食、MHC-II/共刺激、IL-12 型反応を高められる可能性があります。CSF1R 阻害は抑制性 TAM を減らせますが、広い枯渇は組織修復と抗菌貪食も失わせます。

好中球は動員とエフェクター状態の両方を標的にできます。CXCL8-CXCR2 軸を部分的に抑えれば、腫瘍促進性好中球や髄系抑制細胞の流入を減らせます。TGF-β 経路調節はより炎症性･抗腫瘍性の状態へ傾ける可能性があり、PAD4/NET、MPO、エラスターゼ標的法は NET 依存性浸潤や血管作用を抑えることを狙います。しかし CXCR2、ROS、顆粒酵素は細菌防御の中心でもあるため、長期全身阻害は危険です。

膀胱がんには選択性を高める実用上の利点があります。それが膀胱内投与です。局所免疫作動薬、ナノ粒子、腫瘍微小環境応答型製剤を用いれば、腫瘍浸潤髄系細胞へ高濃度を届けつつ全身作用を抑えられます。BCG は、好中球とマクロファージを含む強い局所自然免疫活性化が抗腫瘍に利用できることをすでに示す概念実証です。したがって無差別な髄系枯渇は合理的ではありません。再プログラミングで抗原提示を改善し T 細胞排除を減らした上で、PD-1/PD-L1 阻害と組み合わせる戦略は機序的に魅力があります。

主要な障壁は髄系細胞の高い可塑性、空間的不均一性、安定した選択マーカーの欠如です。マウスの TAM/TAN マーカーはヒト膀胱がんへそのまま対応せず、同じ細胞も短時間で状態を変えます。壊死性･大型腫瘍への局所薬剤浸透、他の抑制性髄系細胞の代償動員、尿路感染、粘膜修復障害、BCG との相互作用も課題です。最も現実的なのは、腫瘍･尿中バイオマーカーで誘導される局所的、可逆的な機能再プログラミングであり、髄系系譜全体の恒久的枯渇ではありません。

Simplified Chinese (SC)

Anonymous Model C | 简体中文

Anonymous Model C

简体中文

24 standardized questions

Q01

问题

在健康肠黏膜中，中性粒细胞如何在与驻留巨噬细胞和树突状细胞相互作用的同时参与屏障防御？哪些机制通常限制中性粒细胞介导的组织损伤？

回答

健康肠黏膜中的中性粒细胞并不是大量常驻细胞，其价值主要在于“可迅速调动”。当黏液层或上皮屏障出现局灶破口、细菌产物进入固有层时，上皮细胞和驻留巨噬细胞通过模式识别受体感知危险，释放 CXCL1/2、在人类中以 CXCL8 为代表的趋化因子，并伴随 IL-1、TNF、补体片段和脂质趋化信号。中性粒细胞随后从血管快速迁入病灶，依靠吞噬、NADPH 氧化酶产生的活性氧、髓过氧化物酶和颗粒抗菌蛋白杀灭越过屏障的微生物；在强烈刺激时还可形成胞外诱捕网。其保护作用的核心是尽快降低局部微生物负荷，阻止一个小的屏障缺损演变成广泛感染。

驻留巨噬细胞决定了这种高强度效应是否能及时“刹车”。稳态肠巨噬细胞对共生菌刺激相对低反应，却保留很强的吞噬能力；它们既能清除微生物，也能吞噬进入凋亡的中性粒细胞。通过 MerTK 等凋亡细胞清除通路进行 efferocytosis 后，巨噬细胞通常减少 NF-κB 驱动的促炎细胞因子，增加 IL-10、TGF-β 和促炎症消退脂质介质，从而把局灶的急性杀菌反应转换为清除碎片和组织修复。树突状细胞则更偏向抗原采样和适应性免疫启动；在没有持续危险信号时，黏膜树突状细胞更倾向于受控的抗原呈递和耐受，而不是不断放大中性粒细胞募集。

限制组织损伤依赖多层制动。黏液、抗菌肽和完整紧密连接首先减少“必须动用中性粒细胞”的机会；趋化因子表达被限制在局部和短时间内。IL-10、TGF-β、腺苷信号、Annexin A1 以及脂氧素、resolvin 等促消退介质可降低持续募集和活化。中性粒细胞本身具有较短寿

命，感染负荷下降后进入凋亡并被巨噬细胞及时清除，可避免继发性坏死释放弹性蛋白酶、组蛋白、MPO 和大量氧化物。上皮和组织液中的抗蛋白酶及抗氧化系统又进一步缓冲这些毒性分子。

因此，健康状态下是明确的分工合作：中性粒细胞负责快速而强力的局部杀菌，巨噬细胞负责控制强度、吞噬死亡细胞并推动修复，树突状细胞负责把真正需要升级的危险信号连接到适应性免疫。只有当募集长期不关闭、凋亡延迟、NET 和蛋白酶大量累积或 efferocytosis 失败时，中性粒细胞才从屏障防御者转变为明显的组织损伤来源。

Q02

问题

在健康肠黏膜中，嗜酸性粒细胞如何参与维持稳态并与巨噬细胞或树突状细胞进行通讯？这些作用与炎症状态下嗜酸性粒细胞的功能有何不同？

回答

嗜酸性粒细胞在健康肠固有层中本来就相当丰富，因此其功能不能简单等同于“过敏效应细胞”。稳态下，CCL11 等 eotaxin 家族趋化因子帮助维持其组织分布，局部生存信号使其处于相对受控的活化水平。动物和部分人类研究提示，肠嗜酸性粒细胞可参与维持上皮完整性、调节微生物群，并通过 IL-6、APRIL 相关信号、TGF-β 等途径影响浆细胞和 IgA 反应；它们还可释放脂质介质和生长相关因子，参与血管、基质和上皮更新。不过，不同物种和肠段之间差异明显，某些“稳态功能”在小鼠中的证据强于人体。

嗜酸性粒细胞与巨噬细胞之间常通过可溶性介质间接交流。低强度的 IL-4、IL-13 可推动巨噬细胞进入更偏清除、基质重塑和修复的功能状态；反过来，驻留巨噬细胞通过控制局部细胞因子、吞噬凋亡细胞和降低组织危险信号来限制嗜酸性粒细胞持续活化。对树突状细胞而言，嗜酸性粒细胞来源的细胞因子和颗粒蛋白在炎症时影响更突出，例如 eosinophil-derived neurotoxin 可在实验系统中作为 alarmin 增强树突状细胞活化。嗜酸性粒细胞虽然在特定条件下能表达 MHC-II 和共刺激分子，但其在健康人肠道中作为主要抗原呈递细胞的地位远没有经典树突状细胞那么确定。

进入炎症状态后，网络会发生质变。IL-33、IL-25、TSLP 等上皮报警素，加上 ILC2/Th2 来源的 IL-5 和增强的 eotaxin 信号，会提高嗜酸性粒细胞数量、寿命和脱颗粒倾向。活化嗜酸性粒细胞释放主要碱性蛋白、嗜酸性粒细胞过氧化物酶、阳离子蛋白、活性氧、半胱氨酰白三烯以及多种炎症因子，可直接破坏上皮、提高通透性，并进一步激活巨噬细胞和树突状细胞。若 IL-4/IL-13、TGF-β 持续存在，原本有利于修复的程序还可能转为异常基质沉积和纤维化。

所以，稳态嗜酸性粒细胞更接近“低强度、局部化的营养/调节型信号源”，而炎症中的嗜酸性粒细胞则表现为数量扩大、寿命延长、颗粒毒性和 2 型细胞因子回路增强。两者并非绝对二分：感染后的嗜酸性粒细胞也可有保护和修复作用；真正决定结果的是组织背景、活化强度和持续时间。

Q03

问题

在健康呼吸道黏膜以及急性微生物攻击期间，中性粒细胞如何与肺泡或间质巨噬细胞及树突状细胞协同，在发挥宿主防御作用的同时限制对上皮的旁观者损伤？

回答

健康呼吸道中，肺泡巨噬细胞是远端气腔最重要的第一线细胞哨兵，间质巨噬细胞和树突状细胞则占据不同的组织生态位。它们平时持续吞噬吸入颗粒和少量微生物，却尽量避免引发大规模炎症。一旦 TLR、NOD 样受体等真正识别到感染，巨噬细胞、上皮细胞和其他局部细胞会产生 IL-1、TNF、CXCL 趋化因子、GM-CSF 和脂质趋化信号，使中性粒细胞在短时间内大量进入感染部位。中性粒细胞通过吞噬、NADPH 氧化酶呼吸爆发、MPO 和颗粒蛋白杀菌；病原负荷很高或不易吞噬时，还可使用胞外诱捕网进行限制。

这种协同具有明显的时序性。早期是巨噬细胞“呼叫”中性粒细胞；随后中性粒细胞杀菌后产生的微生物碎片、IL-1 家族信号、氧化脂质以及组织损伤相关分子又会反向影响巨噬细胞。如果感染尚未受控，这种放大是有利的；若病原已明显下降仍继续放大，就会转为旁观者损伤。树突状细胞在此过程中采集抗原并迁移到引流淋巴结，启动病原特异性 T 细胞反应，使后续清除不必长期依赖最大强度的中性粒细胞炎症。

保护上皮的关键是主动消退。中性粒细胞完成任务后进入凋亡，由肺泡或间质巨噬细胞进行 efferocytosis。吞噬凋亡细胞会抑制巨噬细胞继续产生强烈 NF-κB 型炎症因子，并增加 IL-10、TGF-β、Annexin A1 以及脂氧素和 resolvin 等促消退介质。与此同时，气道表面液中的 α1-抗胰蛋白酶、SLPI 等抗蛋白酶限制中性粒细胞弹性蛋白酶，抗氧化系统降低 MPO/ROS 的毒性，DNase 可帮助清除过量 DNA-rich NET。

因此，决定结果的不是“有无中性粒细胞”，而是募集是否短暂、效应是否局部、清除是否及时。急性细菌或真菌感染中，中性粒细胞往往不可替代；而在重症肺炎、COPD 或部分哮喘中，持续的 CXCL8/GM-CSF、延迟凋亡、巨噬细胞 efferocytosis 障碍以及 NET/蛋白酶过量，会把原本的抗感染协同转变为纤毛、紧密连接和肺泡上皮损伤。

Q04

问题

在稳态条件和 2 型炎症期间，嗜酸性粒细胞如何在气道黏膜中与巨噬细胞和树突状细胞相互作用？

回答

稳态气道中的嗜酸性粒细胞数量较少，肺泡巨噬细胞和树突状细胞虽然持续接触吸入抗原，但通常处于受抑制、低共刺激的状态。巨噬细胞主要清除颗粒和凋亡细胞，树突状细胞进行抗原采样；只要上皮没有释放强烈危险信号，低水平 IL-10、TGF-β 和完整屏障就会限制嗜酸性粒细胞募集和活化。

2 型炎症首先改变的是上游“报警系统”。过敏原、蛋白酶或上皮损伤诱导 TSLP、IL-33、IL-25，进而激活 ILC2 并使树突状细胞上调 OX40L 等共刺激程序、迁移至淋巴结，促进 Th2 分化。ILC2/Th2 产生 IL-5，延长嗜酸性粒细胞生存；IL-4 和 IL-13 则促使上皮和基质细胞产生 CCL11、CCL24 以及在人类气道中尤其重要的 CCL26，从而形成选择性的嗜酸性粒细胞募集。

进入气道后，嗜酸性粒细胞会反向塑造巨噬细胞和树突状细胞。IL-4/IL-13 可推动巨噬细胞进入偏修复的功能状态，短期有利于恢复屏障；但持续存在时，同一类信号也会促进黏液分泌、基质沉积和气道重塑。嗜酸性粒细胞颗粒蛋白、白三烯、活性氧及细胞因子可损伤上皮，制造新的危险信号，从而增强树突状细胞活化。某些颗粒核糖核酸酶，如 eosinophil-derived neurotoxin，在实验中还能直接增强树突状细胞免疫刺激性。相反，巨噬细胞吞噬凋亡嗜酸性粒细胞后可增加 IL-10 和促消退介质，阻断持续募集。

因此，稳态和 2 型炎症之间不是完全不同的细胞，而是同一网络被置于不同强度和持续时间的信号环境。短暂的嗜酸性粒细胞—巨噬细胞交流可支持修复；持续的 IL-5/eotaxin、反复脱颗粒和长期 IL-4/IL-13 则把它变成慢性炎症和重塑回路。上皮报警素和经典树突状细胞在这一通路中的核心地位较明确，而"嗜酸性粒细胞直接作为重要抗原呈递者"的人体证据相对更具情境依赖性。

Q05

问题

在尿路黏膜中，早期抗菌防御期间，中性粒细胞如何与单核细胞或巨噬细胞及树突状细胞协同？哪些机制可防止过度组织损伤？

回答

尿路早期抗菌防御首先由尿路上皮和局部先天免疫细胞启动。尿路致病菌接触膀胱或下尿路黏膜后，通过 TLR 等模式识别通路诱导 IL-1、TNF 和中性粒细胞趋化因子。驻留巨噬细胞放大这一警报并帮助激活局部血管；感染较明显时，CCR2 依赖的单核细胞也会进入组织并补充巨噬细胞群。中性粒细胞在数小时内大量募集，穿过黏膜甚至进入尿腔，通过吞噬、ROS、抗菌颗粒以及强刺激下的 NET 快速压低细菌负荷。

单核细胞/巨噬细胞与中性粒细胞的功能并不重复。巨噬细胞可以吞噬细菌和细胞碎片，并在感染未完全控制时维持适度的细胞因子和趋化因子输出；随后又承担清除凋亡中性粒细胞、终止炎症的工作。树突状细胞则负责抗原采样和适应性免疫启动，在单次、早期的非复杂性 UTI 中通常不如上皮—巨噬细胞—中性粒细胞轴突出，但在复发或复杂感染中，其对 T、B 细胞反应的影响会更重要。

抗菌效应本身也可能破坏尿路上皮。弹性蛋白酶、MPO 产生的氧化物、ROS 和 NET 相关组蛋白都可损伤紧密连接并暴露深层组织。正常情况下，细菌被快速清除后趋化信号随即下降，中性粒细胞进入凋亡并由巨噬细胞 efferocytosis。这个过程会使巨噬细胞减少继续招募中性粒细胞的信号，增加 IL-10、TGF-β 和修复相关程序。前列腺素、促消退脂质介质、局部抗氧化/抗蛋白酶系统以及尿路上皮较强的再生能力共同限制旁观者损伤。

这套系统的本质是速度与克制之间的平衡。中性粒细胞不足会导致细菌扩增甚至上行感染；如果募集持续到病原负荷已很低，则可出现屏障破坏和症状性炎症。很多精细的细胞互作来自动物 UTI 模型，人体中不同巨噬细胞亚群、具体趋化因子及 NET 的相对贡献更为异质，不能把单一实验机制视为所有患者都固定存在。

Q06

问题

在膀胱或下泌尿生殖道黏膜中，肥大细胞释放的介质如何影响巨噬细胞和树突状细胞的功能？这一网络如何同时参与宿主防御和炎症相关症状？

回答

肥大细胞位于膀胱和下泌尿生殖道的血管、神经和上皮附近，因此少量细胞被激活就能迅速改变整个局部髓系网络。微生物产物、补体、IgE/FcεRI 信号、上皮报警素以及神经肽都可诱导其释放预存的组胺、胰蛋白酶样蛋白酶、糜蛋白酶和 TNF，随后再合成 PGD2、半胱氨酰白三烯、细胞因子和生长因子。结果是血管扩张、通透性上升、上皮信号改变，并为其他白细胞进入组织创造条件。

巨噬细胞和树突状细胞表达多类相应受体。TNF、GM-CSF 可增强髓系细胞活化和存活；组胺对巨噬细胞/树突状细胞的作用取决于 H 受体亚型和细胞状态，既可能增强也可能抑制某些炎症因子；肥大细胞蛋白酶可通过 PAR2 等受体影响髓系细胞及上皮。IL-4/IL-13 倾向于推动巨噬细胞进入修复型功能状态，而 TNF/IL-1 丰富的环境则更容易维持炎症。对感染而言，这些变化能加速中性粒细胞募集，并提高树突状细胞成熟、迁移和抗原呈递，从而有利于宿主防御。

同一网络也能制造明显症状。组胺、tryptase、前列腺素以及 NGF 相关信号可敏化膀胱感觉神经，降低 TRPV1、P2X 等通路的激活阈值；血管渗漏引起水肿，蛋白酶、TNF 以及其他髓系细胞产生的 ROS 可破坏尿路上皮屏障。屏障变"漏"后，尿液中的溶质和 ATP 更容易接触黏膜下感觉末梢，于是形成尿急、疼痛与神经源性炎症的正反馈。巨噬细胞产生的 IL-1β、TNF、PGE2 又可继续敏化神经并促进局部肥大细胞反应。

当巨噬细胞转向 efferocytosis、IL-10、TGF-β 和促消退脂质介质，并支持上皮修复时，这一循环可以终止。需要强调的是，慢性膀胱疼痛综合征等疾病高度异质：部分患者确有肥大细胞增加或活化，但并非所有病例都由肥大细胞主导。因此，这一串扰网络是连接抗感染与炎症症状的合理机制框架，而不是对所有慢性膀胱炎症的单一解释。

Q07

问题

在急性肠黏膜炎症及其后的炎症消退阶段，中性粒细胞与巨噬细胞如何通过募集信号、细胞因子、凋亡细胞清除、脂质介质和组织修复程序相互影响？

回答

急性肠黏膜炎症早期，中性粒细胞和巨噬细胞构成一个迅速放大的回路。屏障破坏后，上皮和驻留巨噬细胞释放 IL-1、TNF、CXCL 趋化因子、C5a 和 LTB4 等脂质趋化信号，首先把中性粒细胞招入病灶。中性粒细胞控制微生物的同时释放 ROS、蛋白酶、炎症因子和 LTB4；这些物质又可吸引更多中性粒细胞，并产生 DAMP。与此同时，CCR2 依赖的单核细胞进入黏膜并分化为炎症性巨噬细胞，在危险信号持续存在时继续产生 TNF、IL-1β、IL-6 和趋化因子。

真正的炎症消退不是"所有信号一起变小"，而是信号性质发生转换。完成任务的中性粒细胞进入凋亡，暴露磷脂酰丝氨酸等"eat-me"信号。巨噬细胞通过 MerTK、Axl、TIM 家族以及整合素/桥接分子等系统进行 efferocytosis。吞噬凋亡中性粒细胞后，巨噬细胞抑制持续的 NF-κB 型炎症输出，增加 IL-10、TGF-β，并启动清除碎片和修复所需的代谢程序。更重要的是，及时吞噬可阻止中性粒细胞发生继发性坏死，避免组蛋白、弹性蛋白酶、MPO 等高毒性内容物晚期大量外泄。

脂质介质也发生“类别切换”。早期前列腺素和白三烯促进血管活化和白细胞募集；进入消退期后，脂氧素、resolvin、protectin、maresin 等特殊促消退介质抑制新的中性粒细胞进入，并增强巨噬细胞 efferocytosis。中性粒细胞本身也可能参与这种脂质介质转换，而不是单纯被动退出。吞噬凋亡细胞后的巨噬细胞可产生 TGF-β、amphiregulin、VEGF 家族信号和基质重塑酶，与上皮干/祖细胞和基质细胞共同完成屏障修复。

如果任何一步失灵，保护性炎症就会转为病理性炎症：NET、蛋白酶和 ROS 造成更多上皮破坏，新的 DAMP 又重新招募髓系细胞；efferocytosis 失败则使死亡细胞坏死并持续刺激组织。反过来，过早压制中性粒细胞又会削弱微生物清除。因而，比简单的“M1→M2”二分更准确的描述是：多个巨噬细胞功能状态随时间连续转换，只有杀菌、凋亡、efferocytosis、脂质介质切换和修复按正确顺序衔接，炎症才真正消退。

Q08

问题

嗜酸性粒细胞来源的介质如何改变肠黏膜巨噬细胞的功能状态和树突状细胞的激活？这些相互作用如何推动免疫耐受、组织修复与炎症之间的平衡发生变化？

回答

嗜酸性粒细胞可通过细胞因子、脂质介质和颗粒蛋白改变肠黏膜巨噬细胞与树突状细胞的状态，但结果高度依赖于其所处的是稳态、修复还是强烈炎症环境。相对温和时，嗜酸性粒细胞来源的 IL-4、IL-13 可促使巨噬细胞增强清除、基质重塑和修复相关功能；TGF-β、脂质介质和生长因子还可影响上皮、血管和基质。在一些实验模型中，这种信号与屏障恢复和 IgA 相关稳态相联系。

对树突状细胞来说，炎症状态下的嗜酸性粒细胞影响更强。嗜酸性粒细胞来源的颗粒核糖核酸酶，例如 eosinophil-derived neurotoxin，在实验系统中可作为 alarmin 增强树突状细胞活化；白三烯、ROS、炎症细胞因子以及颗粒蛋白造成的上皮损伤，也会间接创造一个“危险信号丰富”的环境，提高共刺激和抗原呈递。IL-4 还能影响树突状细胞偏向 2 型免疫的功能。嗜酸性粒细胞在炎症时可以表达 MHC-II 和共刺激分子，但其是否在人体肠道中承担重要的直接抗原呈递作用，证据明显弱于经典树突状细胞。

因此，这一串扰可把黏膜平衡推向三个方向。若信号受控，嗜酸性粒细胞可帮助巨噬细胞和树突状细胞维持对食物/共生菌的低反应状态，并支持 IgA 和屏障；若组织处于损伤恢复期，IL-4/IL-13、TGF-β 可促进清除碎片和组织重建；若 IL-5/eotaxin 造成大量嗜酸性粒细胞积聚并发生脱颗粒，则颗粒毒性、半胱氨酰白三烯、ROS 和 alarmin 会激活更多树突状细胞和效应细胞，使“修复”转为持续炎症甚至纤维化。

决定方向的关键不是固定的“嗜酸性粒细胞表型标签”，而是组织信号组合、活化强度和时间。IL-33、IL-25、TSLP 持续上升通常有利于 2 型炎症放大；嗜酸性粒细胞及时凋亡并被巨噬细胞吞噬，则有利于消退。部分稳态功能在小鼠中的证据较强，在人类中的定量贡献仍需谨慎外推。

Q09

问题

在中性粒细胞性气道炎症中，中性粒细胞与巨噬细胞如何通过细胞因子、脂质介质、中性粒细胞胞外诱捕网、凋亡细胞清除和代谢信号相互影响？这种串扰如何决定炎症持续还是消退？

回答

中性粒细胞性气道炎症之所以会持续，常常是因为巨噬细胞和中性粒细胞反复制造彼此需要的募集与生存信号。活化的气道巨噬细胞、上皮和树突状细胞产生 IL-1β、TNF、CXCL8/CXCL 家族趋化因子和 GM-CSF，招募中性粒细胞并延长其寿命。中性粒细胞随后释放 ROS、弹性蛋白酶、MPO、炎症因子和 LTB4，反过来增强巨噬细胞活化并造成上皮 DAMP。LTB4 特别擅长放大中性粒细胞之间的“接力式”募集，而 GM-CSF 等信号会推迟本应较快发生的凋亡。

NET 是这一回路的重要放大器。DNA 网架上携带组蛋白、弹性蛋白酶和 MPO，可捕获微生物；但如果 NET 过量或清除不良，其组蛋白和蛋白酶可直接伤害上皮，并向巨噬细胞提供核酸和氧化蛋白危险信号。不同模型中 TLR9、cGAS-STING、NLRP3 等通路均可能参与，但在哮喘、COPD 与感染中的相对权重并不相同。巨噬细胞由此产生更多 IL-1β 和趋化因子，就会形成“NET 越多—募集越多—NET 更容易继续形成”的正反馈。

代谢状态进一步决定炎症能否退出。炎症性巨噬细胞常增强糖酵解，并出现 succinate/HIF-1α 与 IL-1β 相联系的代谢程序；中性粒细胞则高度依赖糖酵解和磷酸戊糖途径来维持迁移和 NADPH 氧化爆发。低氧、细胞外 ATP、乳酸和腺苷都能改变细胞寿命和细胞因子输出。感染急性期这些适应是有利的，但在长期低氧、黏液潴留的气道中可转为维持炎症的因素。

消退的核心是中性粒细胞凋亡后被巨噬细胞进行 efferocytosis。MerTK 等通路启动后，巨噬细胞增加 IL-10、TGF-β 和促消退脂质介质，并向修复型代谢状态转换；脂氧素和 resolvin 同时减少新的募集。若 efferocytosis 障碍，凋亡细胞继发坏死，蛋白酶和 DAMP 再次外泄。因此，持续炎症通常不是单一因子造成，而是“募集关闭失败、NET/碎片清除失败、代谢降档失败、efferocytosis 失败”同时存在；真正的消退要求这些环节协同逆转。

Q10

问题

在过敏性气道炎症中，肥大细胞和嗜酸性粒细胞（在相关情境下包括嗜碱性粒细胞）如何与树突状细胞和巨噬细胞相互作用，从而放大或限制 2 型免疫反应？

回答

过敏性气道炎症的核心是上皮报警素、树突状细胞和 2 型效应细胞之间的互相放大。过敏原或上皮损伤释放 TSLP、IL-33、IL-25，激活 ILC2 并使经典树突状细胞上调 OX40L 等共刺激程序，随后迁移到淋巴结诱导过敏原特异性 Th2。Th2 和 ILC2 产生 IL-4、IL-5、IL-13，为肥大细胞、嗜酸性粒细胞以及部分情境下的嗜碱性粒细胞构建持续的 2 型细胞因子环境。

肥大细胞在 IgE/FcεRI 交联后快速释放组胺、tryptase、PGD2、半胱氨酰白三烯、TNF 和 2 型细胞因子。PGD2 经 CRTH2、白三烯经相应受体可同时吸引和激活嗜酸性粒细胞、ILC2 和 Th2。IL-5 延长嗜酸性粒细胞寿命，IL-4/IL-13 促进上皮产生 eotaxin。嗜酸性粒细胞

继而释放白三烯、细胞因子、颗粒蛋白和 ROS，损伤上皮并制造更多 alarmin。嗜碱性粒细胞可提供较早的 IL-4 并强化 Th2 环境，但把它们视为人体气道中取代经典树突状细胞的主要抗原呈递者，证据不足。

巨噬细胞可以把这条回路推向两种方向。IL-4Rα 信号使其进入偏修复的功能状态，有利于清除碎片和恢复屏障；若信号持续，则也可促进黏液、基质沉积和气道重塑，并通过 CCL17/CCL22 等途径支持 2 型细胞聚集。相反，巨噬细胞高效吞噬凋亡嗜酸性粒细胞后，会增加 IL-10、TGF-β 和促消退脂质介质，降低进一步募集。随着上皮危险信号下降，树突状细胞也更容易回到低共刺激、偏调节的状态。

所以，放大还是限制取决于回路是否继续由抗原和报警素驱动。持续过敏原暴露、屏障损伤、IgE 激活和 IL-5/eotaxin 轴会让炎症自我维持；抗原减少、嗜酸性粒细胞凋亡、巨噬细胞 efferocytosis 和上皮修复则推动消退。这也解释了为什么阻断 IgE 或关键 2 型细胞因子的治疗常常重塑整个细胞网络，而不仅仅是改变某一种粒细胞数量。

Q11

问题

在急性细菌性尿路感染期间，募集到局部的中性粒细胞和单核细胞来源巨噬细胞如何在病原体清除、细胞因子产生、凋亡细胞清除和黏膜修复过程中协同或竞争？

回答

急性细菌性 UTI 中，中性粒细胞和单核细胞来源巨噬细胞按不同时间尺度进入病灶，因此更像“前锋与后勤/清场”的关系。尿路上皮和驻留髓系细胞感知细菌后释放 CXCL 趋化因子、IL-1、TNF 等信号，中性粒细胞最先大量到达，穿越尿路上皮后常成为尿液中最主要的炎症细胞。其优势是速度：快速吞噬调理后的细菌，使用 NADPH 氧化酶 ROS 和抗菌颗粒，并在高负荷刺激时形成 NET。

CCR2 依赖的单核细胞一般稍晚进入，并分化成炎症性巨噬细胞。它们也能吞噬细菌，但更重要的是持续或重新塑造细胞因子环境、吞噬死亡细胞，并在感染后期推动组织修复。早期巨噬细胞产生 TNF、IL-1β 和趋化因子支持中性粒细胞募集；病原负荷下降后，同一巨噬细胞群可通过 efferocytosis 减少这些信号并增加 IL-10、TGF-β 和修复因子。因此，吞噬凋亡中性粒细胞既是“垃圾清除”，也是把炎症程序切换为修复程序的信号事件。

协同最理想的状态是：中性粒细胞提供快速的灭菌压力，巨噬细胞提供更持久的吞噬、监控和收尾。二者也可能发生竞争，例如争夺被调理的细菌、补体组分以及局部氧和营养物；某些情境中巨噬细胞吞入但未完全杀死细菌，反而形成短暂的胞内存活位点。另一方向上，过强的中性粒细胞 ROS、蛋白酶可损伤周围巨噬细胞和尿路上皮，甚至暴露新的细菌黏附位点。

黏膜真正恢复依赖于这种关系及时改变：趋化信号下降，中性粒细胞退出，巨噬细胞完成 efferocytosis、基质重塑并与再生中的尿路上皮协同。如果中性粒细胞被过早抑制，灭菌不足；如果病原已控制后仍持续存在，NET、弹性蛋白酶、MPO 和 ROS 会维持症状和损伤。动物模型对这种时序分工支持较强，但人体中各单核/巨噬细胞亚群的比例，以及其是否形成细菌暂存库，仍具有明显个体差异。

Q12

问题

在慢性膀胱黏膜炎症中，肥大细胞与巨噬细胞之间的串扰可能如何改变感觉神经信号、血管通透性、上皮屏障功能以及炎症的持续或消退？

回答

慢性膀胱黏膜炎症中，肥大细胞和巨噬细胞可以形成一个连接神经、血管和上皮的正反馈环。肥大细胞紧邻感觉神经和微血管，受到神经肽、补体、上皮信号或其他危险刺激后释放组胺、tryptase、前列腺素、白三烯、TNF 和生长因子。组胺与蛋白酶既提高血管通透性，也能直接降低感觉神经的兴奋阈值；tryptase-PAR2、组胺 H1/H4 以及 NGF 相关信号可增强疼痛、尿急等感觉通路。

当巨噬细胞维持在炎症状态时，这种神经敏化会进一步放大。IL-1β、TNF、PGE2 和 ROS 可使伤害性感受神经更易兴奋，并维持血管内皮活化。被激活的神经又释放 substance P、CGRP 等神经源性介质，反过来影响肥大细胞、血管和巨噬细胞。CCL2/CCR2、CSF1 相关信号可持续补充或维持单核/巨噬细胞，而 SCF/c-Kit 等通路帮助肥大细胞局部存活，于是炎症即使没有强烈感染刺激也可能继续。

上皮屏障把神经和炎症两部分连在一起。肥大细胞蛋白酶、巨噬细胞炎症因子以及其他白细胞产生的 ROS 可破坏尿路上皮紧密连接，提高旁细胞通透性。受损上皮释放 ATP 和 DAMP，使黏膜下感觉末梢更容易接触尿液中的刺激物并激活 P2X 等受体。血管渗漏和水肿又增加局部压力，进一步加重尿急、疼痛。由此形成“屏障更差—神经更敏感—肥大细胞/巨噬细胞更活跃”的循环。

消退依赖巨噬细胞转向 efferocytosis、IL-10、TGF-β 和促消退脂质介质，并支持尿路上皮再生。屏障一旦恢复，尿液刺激物与感觉神经隔离，肥大细胞的持续激活也会下降。但如果 TGF-β 型修复过度，则纤维化和膀胱顺应性下降本身又可能产生症状。值得注意的是，慢性膀胱疼痛/间质性膀胱炎高度异质，肥大细胞并非所有患者的主导机制，因此应把这条轴视为一种可维持疾病的机制，而不是普遍适用的单因解释。

Q13

问题

在溃疡性结肠炎中，中性粒细胞募集、中性粒细胞胞外诱捕网形成、中性粒细胞代谢以及中性粒细胞-巨噬细胞串扰如何分别促进黏膜损伤或炎症消退？

回答

溃疡性结肠炎中，中性粒细胞既是必要的抗微生物效应细胞，也是活动期黏膜损伤最直观的参与者之一。炎症上皮、基质细胞和单核来源巨噬细胞产生 CXCL8 及其他 CXCR1/2 配体、IL-1、TNF 和集落刺激因子，驱动大量中性粒细胞进入结肠黏膜并穿过上皮形成隐窝炎和隐窝脓肿。吞噬和 ROS 有助于控制突破受损屏障的细菌，但细胞外弹性蛋白酶、MPO 氧化产物、MMP 和 ROS 又可扩大糜烂，形成更多微生物进入通道。

NET 进一步增加外源性毒性。DNA-组蛋白网架能捕获微生物，但持续存在的 NET 同时携带弹性蛋白酶和 MPO，可直接伤害上皮、增强凝血和炎症，并作为 DAMP 激活巨噬细胞。核酸感知和 inflammasome 相关通路可能参与，包括不同模型中报道的 TLR9、cGAS-

STING、NLRP3 等，但其在人体 UC 中的相对重要性并未完全统一。临床样本中 NET 标志物与疾病活动度常有关联，但“NET 是主要因果驱动”还是“严重中性粒细胞炎症的伴随标志”，仍需更强的干预性证据区分。

代谢状态能延长这种效应。中性粒细胞高度依赖糖酵解和磷酸戊糖途径，后者提供 NADPH 支撑氧化爆发；炎症黏膜本身低氧，HIF 相关生存程序可能延缓凋亡。乳酸、琥珀酸、细胞外 ATP 等炎症代谢物又可同时影响中性粒细胞和巨噬细胞。原本为急性杀菌服务的代谢适应，在慢性环境中就可能变成“让细胞继续活、继续输出毒性”的机制。

相反，炎症消退依赖巨噬细胞把中性粒细胞从“危险信号”转换为“待清除对象”。MerTK 等通路介导的 efferocytosis 会降低促炎因子并增加 IL-10、TGF-β、脂氧素、resolvin 以及组织修复程序；若清除失败，凋亡细胞继发坏死，DAMP 再次释放。因而治疗上的理想目标不是全面消灭中性粒细胞，而是减少过度募集和细胞外毒性，同时保留吞噬杀菌，并加速凋亡、efferocytosis 和屏障重建。

Q14

问题

在克罗恩病中，中性粒细胞、单核细胞来源巨噬细胞和树突状细胞之间失调的相互作用如何改变微生物处理、抗原呈递、炎症持续以及上皮修复？

回答

克罗恩病中的髓系异常可以理解为：正常应当完成的“微生物感知—清除—抗原处理—炎症终止—上皮修复”链条没有顺利结束。屏障缺损使肠腔微生物持续进入固有层，中性粒细胞被招募进行清除；但 NOD2、ATG16L1 等与微生物识别、自噬和胞内处理有关的易感通路异常，可使微生物成分不易被彻底处理，于是髓系细胞长期暴露于刺激。

CCR2 依赖的单核细胞不断进入组织，并可能无法像健康肠驻留巨噬细胞那样获得“高吞噬、低炎症反应”的稳态特征，而是持续产生 TNF、IL-1β、IL-6、IL-23 和趋化因子。中性粒细胞的 ROS、蛋白酶和 NET 在控制微生物的同时扩大上皮损伤、释放 DAMP。肉芽肿性病变中，巨噬细胞长期存在并持续处理难以清除的微生物或颗粒，也可成为炎症维持的一部分。

树突状细胞把这种持续的先天刺激连接到适应性免疫。大量微生物抗原、IL-12/IL-23 家族信号和较强共刺激更容易促进 Th1/Th17 相关反应，而不是黏膜耐受。活化 T 细胞返回组织后产生 IFN-γ、IL-17 等介质，又刺激巨噬细胞和上皮产生更多趋化因子。于是形成“清除不彻底→抗原暴露更多→适应性炎症更强→屏障更破→微生物进入更多”的闭环。

上皮修复则需要巨噬细胞 efferocytosis、amphiregulin/TGF-β 等生长与修复信号、上皮干细胞再生和受控的基质重塑。持续 TNF/IL-1、蛋白酶和低氧会妨碍这些过程；反过来，过度 TGF-β 型修复又可导致狭窄和纤维化。抗 TNF 的临床有效性支持炎症性髓系细胞因子在病程中具有因果意义，但克罗恩病高度异质：不同患者中“先天杀菌缺陷、单核/巨噬细胞过度炎症、树突状细胞抗原呈递和纤维化修复”的权重并不相同，因此不能只用“免疫过强”概括。

Q15

问题

在重度中性粒细胞性哮喘中，中性粒细胞、巨噬细胞和树突状细胞如何形成自我维持的炎症回路？中性粒细胞胞外诱捕网、炎症小体信号、微生物信号以及糖皮质激素抵抗可能分别发挥什么作用？

回答

重度中性粒细胞性哮喘常由“上皮—巨噬细胞/树突状细胞—Th17—中性粒细胞”回路维持，而不是经典的嗜酸性 2 型炎症。污染物、感染、DAMP 或菌群失衡产生的微生物信号可激活 TLR/NOD、NF-κB 通路，使上皮和巨噬细胞释放 IL-1β、TNF、CXCL8、GM-CSF。树突状细胞和巨噬细胞又可通过 IL-6/IL-23 环境支持 Th17；IL-17 反过来诱导上皮产生 CXCL8、G-CSF，进一步募集并延长中性粒细胞寿命。中性粒细胞的弹性蛋白酶、ROS 和 DAMP 又重新刺激上皮和巨噬细胞，形成自维持回路。

NET 可以把这一回路再放大一层。细胞外 DNA、组蛋白、MPO 和弹性蛋白酶增加黏液黏稠度、损伤上皮，并向巨噬细胞和树突状细胞提供先天免疫刺激。在多种模型中，NET 成分和细胞外 ATP 可促进 NLRP3 inflammasome 和 IL-1β，从而进一步支持中性粒细胞/Th17 炎症。但 NLRP3 并非所有重症哮喘患者都同等重要；所谓“中性粒细胞性哮喘”本身包括感染相关、吸烟/污染相关、肥胖相关及治疗塑形后的多个内型。

微生物信号可成为持续的外源驱动。慢性细菌定植、反复感染或气道菌群组成改变持续提供 TLR 配体，使 IL-1/CXCL8 轴难以关闭。这可能解释部分患者为何对大环内酯类治疗有反应，但其作用不一定仅来自抗菌，还可能包括降低中性粒细胞趋化和巨噬细胞炎症输出；同时必须考虑耐药和菌群扰动。若真正存在持续感染，过度抑制中性粒细胞反而可能危险。

糖皮质激素抵抗也具有多因素基础。中性粒细胞/Th17 炎症一般比嗜酸性 2 型炎症更不敏感；氧化应激、p38 MAPK 等可影响糖皮质激素受体功能，激素本身还可能延迟中性粒细胞凋亡。HDAC2 下降被提出为氧化应激相关耐药机制之一，但其在人类哮喘中的作用强度并非所有研究都一致。总体上，只有当募集、NET/DAMP、inflammasome、微生物刺激和消退失败同时被打断，才可能真正终止这一自我维持网络，同时还要尽量保留抗细菌防御。

Q16

问题

在嗜酸性粒细胞性哮喘中，嗜酸性粒细胞、肥大细胞以及在相关情境下的嗜碱性粒细胞，如何与巨噬细胞、树突状细胞和气道上皮相互作用，从而启动、放大并最终促使 2 型炎症消退？

回答

嗜酸性粒细胞性哮喘的启动通常发生在气道上皮。过敏原、病毒、蛋白酶和污染物使上皮释放 IL-33、TSLP、IL-25，激活 ILC2 并条件化经典树突状细胞。树突状细胞迁移到淋巴结后诱导过敏原特异性 Th2；Th2 与 ILC2 产生 IL-5 维持嗜酸性粒细胞，同时 IL-4/IL-13 促使上皮产生 eotaxin、黏液和其他 2 型趋化信号。肥大细胞和嗜碱性粒细胞则通过 IgE 和细胞因子通路进入同一网络。

放大阶段，各细胞开始互相喂养信号。IgE 交联的肥大细胞释放组胺、PGD2 和半胱氨酰白三烯，既引起支气管收缩，也通过 CRTH2 等通路吸引嗜酸性粒细胞、ILC2 和 Th2。嗜碱性粒细胞可快速提供 IL-4，增强 2 型偏向，但经典树突状细胞仍是主要专业抗原呈递细

胞。嗜酸性粒细胞释放白三烯、IL-4/IL-13、颗粒蛋白和 ROS，造成上皮损伤并诱导更多报警素；IL-4Rα 信号同时使巨噬细胞进入修复/重塑型功能状态。

气道上皮本身是回路的重要主动节点。IL-13 诱导杯状细胞化生和 MUC5AC 等黏液基因，上皮 eotaxin 决定嗜酸性粒细胞在组织中的定位。只要损伤持续，TSLP/IL-33 就继续激活树突状细胞和 ILC2。慢性状态下，嗜酸性粒细胞和肥大细胞来源的 TGF-β、蛋白酶、脂质介质可促进平滑肌改变和基底膜下纤维化，使反复炎症转化为结构性气道重塑。

消退需要上游抗原/报警素信号下降、嗜酸性粒细胞进入凋亡并被巨噬细胞高效 efferocytosis。此时巨噬细胞增加 IL-10 和促消退脂质介质，调节性 T 细胞以及低活化树突状细胞减少新的 Th2 启动。由于各节点紧密连接，阻断一个上游细胞因子往往同时改变嗜酸性粒细胞迁移、肥大细胞活化、巨噬细胞状态和上皮行为。核心通路较明确，而嗜碱性粒细胞的必要性及嗜酸性粒细胞直接抗原呈递在人体中的贡献更具情境依赖性。

Q17

问题

在复发性尿路感染中，既往感染如何通过训练性免疫、免疫耐受或组织重塑改变膀胱黏膜中的中性粒细胞和单核细胞/巨噬细胞反应？目前对这些机制各自的证据强度如何？

回答

既往 UTI 可能通过三类不同机制改变下一次感染时的中性粒细胞和单核/巨噬细胞反应：训练性免疫、免疫耐受和组织重塑。三者不能混为一谈，因为它们对二次反应的预测甚至可能相反。训练性免疫强调长期存在的代谢和表观遗传重编程，例如炎症/抗菌基因染色质开放度增加、糖酵解增强以及甲羟戊酸或线粒体代谢变化，使后续刺激时巨噬细胞更快产生趋化因子、更强吞噬并更早募集中性粒细胞。成熟中性粒细胞寿命很短，因此真正持续数月的“中性粒细胞记忆”更可能来自骨髓祖细胞重编程或膀胱微环境改变，而不是同一批成熟细胞自身保存记忆。

免疫耐受则可能产生相反结果。反复 TLR/LPS 刺激可使部分 TNF、IL-1 和趋化因子基因变得低反应，同时保留某些抗菌或修复通路。对膀胱来说，这也许减少二次感染时的组织损伤，却可能因中性粒细胞募集不足而给细菌再次扩增机会。经典 endotoxin tolerance 在其他系统证据充分，但在复发性 UTI 患者膀胱髓系细胞中的直接证明仍有限，因此应视为合理推断而非已确认的主导机制。

组织重塑目前具有更强的实验支持。既往感染可改变尿路上皮分化、屏障性质、基质细胞趋化因子表达以及局部免疫细胞生态位；持续抗原或胞内细菌库也能改变组织的基线炎症阈值。因此，第二次感染可能被“预先敏化”而更剧烈，也可能因为屏障和局部免疫环境已强化而更快清除。即使单个巨噬细胞没有发生自主训练，细胞数量、定位和刺激阈值的改变也足以重塑总体反应。

证据强度因此不均衡：感染诱导的上皮/组织重塑在动物模型中较为可靠，也符合人类复发病程；单核/巨噬细胞的训练性免疫在其他感染背景已被充分证明，在尿路中越来越具有可信度，但直接人体证据仍少；成熟中性粒细胞自身的长期记忆最不可信。耐受在机制上同样合理，却尚缺乏复发性 UTI 中的定量证据。真正区分三者需要纵向人体取样、单细胞表观组学和二次刺激功能实验。

Q18

问题

在膀胱癌中，肿瘤相关中性粒细胞和肿瘤相关巨噬细胞如何在黏膜肿瘤微环境中相互作用，并影响抗原呈递、T 细胞活性、血管生成、肿瘤侵袭以及免疫治疗反应？

回答

膀胱癌中的肿瘤相关巨噬细胞（TAM）和肿瘤相关中性粒细胞（TAN）可形成互相强化的免疫抑制和组织重塑网络。肿瘤/基质细胞通过 CCL2、CSF1 等信号招募并维持巨噬细胞，通过 CXCL8-CXCR2、G-CSF 等吸引中性粒细胞。被肿瘤环境重编程后，巨噬细胞可降低 IL-12 和有效抗原呈递，提高 IL-10、TGF-β、ARG1 和 PD-L1；中性粒细胞也可通过 ARG1、ROS、蛋白酶和 NET 获得 T 细胞抑制能力。这里更适合用连续功能状态描述，而不是把所有细胞硬分成“M1/M2”或“N1/N2”。

二者可从多个层面压制 T 细胞。TAM 的 IL-10/TGF-β、PD-L1 降低 T 细胞活化；TAN 的 ROS 和精氨酸耗竭损害 TCR 信号和增殖；两类细胞还可帮助募集 Treg 或其他抑制性髓系细胞。若树突状细胞成熟和抗原呈递同时受抑制，则初始抗肿瘤 T 细胞启动也会变差。反过来，具有较高 MHC-II、共刺激和 IL-12 的巨噬细胞以及处于适当炎症状态的中性粒细胞也能支持抗肿瘤免疫，因此关键在于肿瘤微环境把它们推向哪一端。

血管生成与侵袭同样是协同输出。TAM 产生 VEGF、MMP、TGF-β，促进血管和基质重塑；TAN 的 MMP9、弹性蛋白酶、VEGF 相关信号和 NET 可在实验中帮助打开组织通路、捕获循环肿瘤细胞并促进侵袭。低氧通过 HIF 等机制进一步强化两类髓系细胞的免疫抑制和促血管生成状态。

这也影响免疫治疗。高外周中性粒细胞/淋巴细胞比值或髓系细胞丰富常与较差结局相关，但这种关联并不能证明单一因果通路。髓系 PD-L1、抗原呈递不足和 T 细胞排斥都可能降低检查点抑制剂效果。另一方面，膀胱内 BCG 恰好证明局部强烈的中性粒细胞和巨噬细胞活化也可以成为抗肿瘤机制的一部分。因此，治疗目标不应是简单清空髓系细胞，而应选择性减少免疫抑制、促血管生成和 NET-rich 状态，同时保留吞噬、抗原呈递以及尿路局部抗菌能力。

Q19

问题

在溃疡性结肠炎中，哪些具有机制学合理性的策略可能抑制致病性中性粒细胞募集或中性粒细胞胞外诱捕网相关损伤，同时尽量保留抗微生物防御和巨噬细胞介导的修复？请说明预期获益、潜在权衡及证据缺口。

回答

在 UC 中，理想策略应更多压制中性粒细胞的“细胞外组织毒性”而不是吞噬杀菌本身。第一类思路是部分阻断 CXCL8-CXCR1/2 募集轴，减少中性粒细胞进入隐窝，从而降低 ROS、蛋白酶和 NET 负担。但如果全身、强力抑制 CXCR2，会削弱细菌感染时的募集能力；目前这类药物也并未成为 UC 的确立治疗。结肠局部给药、短疗程或只用于具有高 NET/高中性粒细胞生物标志的患者，理论上更能保留全身抗菌能力。

第二类是直接限制 NET 相关损伤。PAD4 抑制可减少部分 NETosis 所需的染色质去凝聚，但并非所有 NET 都依赖 PAD4，而且 PAD4 也并非只在中性粒细胞中有作用。DNase 可切断 DNA 网架，却可能把仍有活性的弹性蛋白酶、MPO 和组蛋白释放到组织液中，因此单纯"把网剪碎"未必足够；DNase 与抗蛋白酶、组蛋白中和或 MPO 抑制联用在机制上更合理。弹性蛋白酶/MPO 抑制可减少组织毒性，但抑制过强同样会牺牲微生物杀伤。

第三类策略不是阻止中性粒细胞来，而是加速正常消退。Annexin A1、脂氧素/resolvin 等通路可减少后续募集，同时提高巨噬细胞 efferocytosis；MerTK 相关清除通路增强后，可保留感染早期的中性粒细胞杀菌，却缩短后期组织中毒性阶段。促进巨噬细胞修复和上皮屏障恢复，也能从源头减少持续驱动中性粒细胞的微生物刺激。不过，如果把"修复型"信号推得过强，也可能出现免疫抑制或纤维化。

现有 IBD 治疗通过降低 TNF、IL-23 相关炎症和上皮趋化因子，常可间接减少中性粒细胞，但并非真正的"选择性保留抗菌功能"方案。最大的证据缺口是：NET、CXCR2 和促消退靶点多数仍停留在临床前或尚无明确 UC 临床获益。未来试验不能只看中性粒细胞数量，还应同时评估黏膜细菌控制、NET 负荷、巨噬细胞 efferocytosis、上皮愈合和感染率。最可行的方向可能是局部、可逆、按炎症状态精准调节，而不是系统性清除中性粒细胞。

Q20

问题

如何利用肠道巨噬细胞和粒细胞的代谢或表观遗传重编程来恢复炎症性肠病中的黏膜稳态？哪些机制可能导致疗效丧失或非预期的免疫抑制？

回答

代谢或表观遗传重编程的优势在于"改变髓系细胞做什么"，而不是直接删除整个细胞群。炎症性肠巨噬细胞常增强糖酵解、mTOR 和 succinate-HIF-1α 相关程序，从而支持 IL-1β、TNF 和趋化因子输出。理论上，可通过激活 AMPK、改善线粒体氧化代谢、增强 Nrf2/itaconate 抗氧化轴或降低过度糖酵解，把巨噬细胞推向更强 efferocytosis 和修复状态。表观遗传层面，BET 蛋白、组蛋白乙酰化/去乙酰化和甲基化可决定炎症基因的可及性，因此也可能用来清除"训练性炎症"留下的持续转录记忆。

但粒细胞需要更谨慎。中性粒细胞高度依赖糖酵解和磷酸戊糖途径完成迁移、存活以及 NADPH 氧化酶依赖的杀菌。全局抑制糖酵解或 NADPH 生成虽然可能减少 ROS 损伤，却可能同时毁掉抗细菌能力。更合理的目标是选择性限制过度 NET、胞外脱颗粒或异常延长的寿命，而保留吞噬体内的杀菌。HIF 相关持续生存、特定脂质代谢和 NET 染色质程序都是候选，但在 IBD 中大多仍属机制性或临床前研究。

疗效丧失有多种来源。肠单核细胞持续从血液补充，新来的炎症细胞会稀释已经被重编程的局部巨噬细胞；TNF、GM-CSF、IL-1、IL-23 等强细胞因子也可覆盖代谢干预的效果。微生物群代谢物可能把相同通路推向相反方向，代谢冗余、疾病异质性以及药物难以进入严重炎症/纤维化区域都会形成逃逸。

意外免疫抑制是最大风险。过度抑制糖酵解、mTOR、ROS 或染色质开放性，可同时降低吞噬、inflammasome、细胞因子募集和抗原呈递；过度强化 TGF-β 型修复又可能促进纤维化，并潜在降低对肿瘤前病变的免疫监视。系统性表观遗传药物还会影响淋巴细胞和上皮干细胞。因此，最有前景的是细胞/组织特异的递送、可逆剂量，并同步监测巨噬细胞状态、中性粒细胞杀菌能力和感染风险，而不是全身性地"重置"所有髓系细胞。

Q21

问题

能够改变 2 型细胞因子信号的生物制剂，除降低嗜酸性粒细胞数量外，还可能如何重塑气道黏膜中的嗜酸性粒细胞-肥大细胞-巨噬细胞-树突状细胞网络？

回答

改变 2 型细胞因子信号的生物制剂之所以能重塑整个气道网络，是因为 IL-5、IL-4/IL-13 以及更上游的上皮细胞因子并不只是控制嗜酸性粒细胞数量。抗 IL-5 或抗 IL-5R 治疗会减少嗜酸性粒细胞生成、存活和组织滞留，同时也减少其来源的白三烯、颗粒蛋白、TGF-β、IL-4/IL-13 等二级信号，因此上皮损伤、巨噬细胞重塑和树突状细胞受危险信号刺激都会下降。Benralizumab 通过 IL-5Rα 介导的细胞毒作用还可减少嗜碱性粒细胞，从而同时改变一个潜在的早期 IL-4 来源。

IL-4Rα 阻断的网络效应更广，因为同时阻断 IL-4 和 IL-13。气道上皮产生更少 eotaxin、黏液和 2 型炎症相关 NO 信号；B 细胞 IgE 类转换支持下降；巨噬细胞较少被持续推向 IL-4/IL-13 条件化的修复/重塑状态；树突状细胞周围的趋化因子和组织环境也会变化。临床上可出现短暂外周血嗜酸性粒细胞升高，因为组织迁入降低的速度可能快于骨髓产生下降，这正说明"血细胞计数"并不能完整代表黏膜网络发生了什么。

更上游的 TSLP 阻断可同时降低树突状细胞活化、ILC2 刺激以及支持肥大细胞/嗜碱性粒细胞的上皮信号，因此可能影响比单纯嗜酸性粒细胞更广的炎症维度。IL-33/ST2 轴从机制上也可影响 ILC2、肥大细胞和嗜酸性粒细胞，但其临床确立程度不如已经广泛应用的 IL-5、IL-4Rα 和 TSLP 靶向治疗。

这些变化可改善上皮屏障并减少巨噬细胞"清理炎症残骸"的负担，但也可能改变正常修复和抗寄生虫免疫。尤其是肥大细胞的生存主要不依赖 IL-5，因此即使嗜酸性粒细胞几乎被清除，肥大细胞仍可能存在。总体上，抗 IL-5 更偏向瓦解嗜酸性粒细胞支路，IL-4Rα 阻断同时重塑上皮、B 细胞和髓系细胞，而上游报警素阻断则有潜力重构树突状细胞—ILC2—粒细胞的整个黏膜网络。

Q22

问题

在中性粒细胞性哮喘中，哪些针对髓系细胞的策略可能中断中性粒细胞-巨噬细胞炎症回路，同时保留抗细菌防御？这些策略所依据的机制中，哪些已有临床证据，哪些仍主要处于临床前阶段？

回答

中性粒细胞性哮喘的治疗难点在于：造成气道损伤的中性粒细胞—巨噬细胞回路，也是抗细菌防御的一部分。因此更合理的原则是“部分、局部、按状态阻断”，而不是系统性清除髓系细胞。CXCR2 拮抗剂可阻断 CXCL8 家族趋化信号，明显减少气道中性粒细胞。临床研究已证明其能降低痰中性粒细胞，说明靶点确实被命中，但对哮喘临床结局的改善并不稳定，而且存在感染风险；因此它属于“机制得到人体验证、但并非标准治疗”。

长期大环内酯类，尤其是选定持续性/重症哮喘患者中的 azithromycin，具有较强的临床减轻急性加重证据，而且确实会影响髓系细胞：可降低部分中性粒细胞趋化、巨噬细胞细胞因子及 inflammasome 相关输出。但其获益究竟有多少来自抗菌、多少来自免疫调节并不完全清楚，同时会带来耐药、QT 风险和菌群改变，因此不能把它视为一个“纯粹、精准”的中性粒细胞—巨噬细胞重编程治疗。抗 TSLP 等上游生物制剂已用于重症哮喘，但它们并非髓系细胞特异靶向。

更选择性的方案大多仍属临床前或早期转化。NLRP3/IL-1 抑制可理论上削弱巨噬细胞与中性粒细胞互相放大的回路，但过强抑制 IL-1 会削弱感染防御。DNase、PAD4 抑制、MPO/弹性蛋白酶抑制以及组蛋白中和等 NET 靶向方法试图减少细胞外毒性、保留吞噬功能，但在人类中性粒细胞性哮喘尚缺乏确立疗效。促进 MerTK/efferocytosis、使用 resolvin 等促消退介质则更符合“让中性粒细胞完成早期杀菌后尽快退出”的思路，目前仍主要实验性。

未来最有可能成功的设计是吸入局部给药 + 生物标志物分型 + 同步监测抗菌能力。慢性细菌定植患者与无感染、主要由 IL-1/Th17 驱动的患者不能采用同一强度的中性粒细胞抑制。到目前为止，还没有一种治疗是因为“明确切断中性粒细胞—巨噬细胞回路且已证明保留抗细菌防御”而成为临床标准，这正是该领域最重要的转化缺口。

Q23

问题

训练性免疫或黏膜疫苗策略是否可能通过有益地重编程单核细胞/巨噬细胞和中性粒细胞来减少复发性尿路感染？请解释其拟议机制、潜在的组织特异性风险以及当前证据的局限。

回答

训练性免疫或黏膜疫苗有可能通过“提高下一次感染时的启动速度”减少复发性 UTI，而不是让膀胱长期处于炎症状态。训练性免疫更可能发生在寿命较长的单核/巨噬细胞祖细胞或组织巨噬细胞中：NOD、C 型凝集素等模式识别信号可诱导组蛋白可及性、糖酵解、甲羟戊酸和线粒体代谢的持久变化，使二次接触细菌时更快产生趋化因子、更有效吞噬并更早招募中性粒细胞。成熟中性粒细胞寿命短，所以长期“训练”更可能发生在骨髓祖细胞或膀胱组织环境，再让新生成的中性粒细胞表现出更易应答的状态。

黏膜疫苗则增加适应性特异性。树突状细胞呈递细菌抗原后，可诱导尿路相关的 T 细胞、抗体以及 IgA/IgG 反应；疫苗佐剂又可能同时训练先天免疫。口服或舌下的细菌裂解物、多价细菌疫苗在一些临床研究中显示复发减少信号，但实际保护究竟由适应性免疫、训练性免疫还是非特异性反复暴露共同造成，目前很难完全拆分。

组织特异性风险不可忽视。若膀胱被“训练”得过度敏感，低水平细菌暴露也可能引起过强 CXCL8、中性粒细胞 ROS、NET 和疼痛性炎症。反复先天刺激还可能产生免疫耐受而不是训练，使 TNF/IL-1 反应被压低，反而利于细菌持续。对尿路上皮干细胞和基质的长期重塑可能提高屏障能力，也可能带来纤维化、感觉神经高敏或有利于胞内细菌库持续。适应性免疫的交叉反应和微生物群改变同样需要监测。

目前的证据更支持“概念可行”，而不是已经证明一条明确的人体髓系重编程通路。复发性 UTI 的免疫预防确有临床信号，但产品、试验设计、监管状态和终点差异很大；直接证明人膀胱巨噬细胞存在保护性训练染色质状态、证明骨髓层面的中性粒细胞祖细胞重编程并与复发减少有因果关系的研究仍少。真正有说服力的研究应把随机临床结局与膀胱/尿液单细胞组学、表观遗传和二次刺激功能实验结合起来。

Q24

问题

如何通过选择性重编程肿瘤相关巨噬细胞和中性粒细胞来增强膀胱癌的抗肿瘤免疫，同时尽量减少对保护性尿路黏膜免疫的破坏？请比较不同机制策略及其主要转化障碍。

回答

膀胱癌中选择性重编程 TAM 和 TAN 的核心，不是把髓系细胞整体清除，而是把它们从免疫抑制、促血管生成、促侵袭状态推向支持抗原呈递和 T 细胞杀伤的状态，同时保留尿路平时所需的吞噬和抗菌能力。对巨噬细胞而言，可考虑功能激活而非纯耗竭：CD40、TLR 或 STING 激动，抑制 PI3Kγ 类免疫抑制信号，或阻断 CD47-SIRPα“别吃我”通路，都可提高肿瘤吞噬、MHC-II/共刺激和 IL-12 型反应。CSF1R 抑制可减少某些抑制性 TAM，但过度耗竭也会牺牲组织修复和抗感染吞噬。

中性粒细胞可从“募集”和“效应状态”两端干预。部分阻断 CXCL8-CXCR2 可减少肿瘤促进型中性粒细胞和髓系抑制细胞进入；TGF-β 轴调节有可能把 TAN 推向更炎症性、抗肿瘤的状态；PAD4/NET、MPO 或弹性蛋白酶靶向则试图减少 NET 促进侵袭和血管改变的作用。但 CXCR2、ROS 和颗粒酶也是细菌防御的重要组成，因此慢性全身抑制风险很高。

膀胱癌提供了一个非常有价值的选择性优势：可以膀胱内给药。局部免疫激动剂、纳米载体或肿瘤微环境响应制剂可使肿瘤髓系细胞获得高暴露，同时减少全身中性粒细胞和巨噬细胞受到影响。BCG 已经是一个现实的“概念验证”：它通过强烈局部先天免疫，包括中性粒细胞和巨噬细胞活化，产生抗肿瘤效应。这反而提示不能粗暴耗竭髓系细胞。若重编程能先改善抗原呈递、减少 T 细胞排斥，再与 PD-1/PD-L1 阻断联合，机制上更有吸引力。

主要转化障碍是髓系细胞高度可塑、空间异质且缺少稳定标志物。小鼠 TAM/TAN 标志并不能直接映射到人膀胱癌；同一细胞数小时内就可换状态。局部药物进入坏死/大体积肿瘤的能力、其他抑制性髓系细胞的代偿募集、尿路感染风险、黏膜修复受损以及与 BCG 的相互作用都需要解决。最可信的路线因此是：局部、可逆、由肿瘤和尿液生物标志物引导的功能重编程，而不是永久耗竭整个髓系谱系。

Traditional Chinese (TC)

Anonymous Model C | 繁體中文

Anonymous Model C

繁體中文

24 standardized questions

Q01

問題

在健康腸黏膜中，中性粒細胞如何在與駐留巨噬細胞和樹突狀細胞相互作用的同時參與屏障防禦？哪些機制通常限制中性粒細胞介導的組織損傷？

回答

健康腸黏膜中的中性粒細胞並不是大量常駐細胞，其價值主要在於“可迅速調動”。當黏液層或上皮屏障出現局灶破口、細菌產物進入固有層時，上皮細胞和駐留巨噬細胞通過模式識別受體感知危險，釋放 CXCL1/2、在人類中以 CXCL8 為代表的趨化因子，並伴隨 IL-1、TNF、補體片段和脂質趨化訊號。中性粒細胞隨後從血管快速遷入病灶，依靠吞噬、NADPH 氧化酶產生的活性氧、髓過氧化物酶和顆粒抗菌蛋白殺滅越過屏障的微生物；在強烈刺激時還可形成胞外誘捕網。其保護作用的核心是盡快降低局部微生物負荷，阻止一個小的屏障缺損演變成廣泛感染。

駐留巨噬細胞決定了這種高強度效應是否能及時“剎車”。穩態腸巨噬細胞對共生菌刺激相對低反應，卻保留很強的吞噬能力；它們既能清除微生物，也能吞噬進入凋亡的中性粒細胞。通過 MerTK 等凋亡細胞清除通路進行 efferocytosis 後，巨噬細胞通常減少 NF-κB 驅動的促炎細胞激素，增加 IL-10、TGF-β 和促發炎消退脂質介質，從而把局灶的急性殺菌反應轉換為清除碎片和組織修復。樹突狀細胞則更偏向抗原採樣和適應性免疫啓動；在沒有持續危險訊號時，黏膜樹突狀細胞更傾向於受控的抗原呈遞和耐受，而不是不斷放大中性粒細胞募集。

限制組織損傷依賴多層制動。黏液、抗菌肽和完整緊密連接首先減少“必須動用中性粒細胞”的機會；趨化因子表達被限制在局部和短時間內。IL-10、TGF-β、腺苷訊號、Annexin A1 以及脂氧素、resolvin 等促消退介質可降低持續募集和活化。中性粒細胞本身具有較短壽命，感染負荷下降後進入凋亡並被巨噬細胞及時清除，可避免繼發性壞死釋放彈性蛋白酶、組蛋白、MPO 和大量氧化物。上皮和組織液中的抗蛋白酶及抗氧化系統又進一步緩衝這些毒性分子。

因此，健康狀態下是明確的分工合作：中性粒細胞負責快速而強力的局部殺菌，巨噬細胞負責控制強度、吞噬死亡細胞並推動修復，樹突狀細胞負責把真正需要升級的危險訊號連接到適應性免疫。只有當募集長期不關閉、凋亡延遲、NET 和蛋白酶大量累積或 efferocytosis 失敗時，中性粒細胞才從屏障防禦者轉變為明顯的組織損傷來源。

Q02

問題

在健康腸黏膜中，嗜酸性粒細胞如何參與維持穩態並與巨噬細胞或樹突狀細胞進行通訊？這些作用與發炎狀態下嗜酸性粒細胞的功能有何不同？

回答

嗜酸性粒細胞在健康腸固有層中本來就相當豐富，因此其功能不能簡單等同於“過敏效應細胞”。穩態下，CCL11 等 eotaxin 家族趨化因子幫助維持其組織分布，局部生存訊號使其處於相對受控的活化水平。動物和部分人類研究提示，腸嗜酸性粒細胞可參與維持上皮完整性、調節微生物群，並通過 IL-6、APRIL 相關訊號、TGF-β 等途徑影響漿細胞和 IgA 反應；它們還可釋放脂質介質和生長相關因子，參與血管、基質和上皮更新。不過，不同物種和腸段之間差異明顯，某些“穩態功能”在小鼠中的證據強於人體。

嗜酸性粒細胞與巨噬細胞之間常通過可溶性介質間接交流。低強度的 IL-4、IL-13 可推動巨噬細胞進入更偏清除、基質重塑和修復的功能狀態；反過來，駐留巨噬細胞通過控制局部細胞激素、吞噬凋亡細胞和降低組織危險訊號來限制嗜酸性粒細胞持續活化。對樹突狀細胞而言，嗜酸性粒細胞來源的細胞激素和顆粒蛋白在發炎時影響更突出，例如 eosinophil-derived neurotoxin 可在實驗系統中作為 alarmin 增強樹突狀細胞活化。嗜酸性粒細胞雖然在特定條件下能表達 MHC-II 和共刺激分子，但其在健康人腸道中作為主要抗原呈遞細胞的地位遠沒有經典樹突狀細胞那麼確定。

進入發炎狀態後，網路會發生質變。IL-33、IL-25、TSLP 等上皮報警素，加上 ILC2/Th2 來源的 IL-5 和增強的 eotaxin 訊號，會提高嗜酸性粒細胞數量、壽命和脫顆粒傾向。活化嗜酸性粒細胞釋放主要鹼性蛋白、嗜酸性粒細胞過氧化物酶、陽離子蛋白、活性氧、半胱氨酰白三烯以及多種發炎因子，可直接破壞上皮、提高通透性，並進一步活化巨噬細胞和樹突狀細胞。若 IL-4/IL-13、TGF-β 持續存在，原本有利於修復的程序還可能轉為異常基質沈積和纖維化。

所以，穩態嗜酸性粒細胞更接近“低強度、局部化的營養/調節型訊號源”，而發炎中的嗜酸性粒細胞則表現為數量擴大、壽命延長、顆粒毒性和 2 型細胞激素迴路增強。兩者並非絕對二分：感染後的嗜酸性粒細胞也可有保護和修復作用；真正決定結果的是組織背景、活化強度和持續時間。

Q03

問題

在健康呼吸道黏膜以及急性微生物攻擊期間，中性粒細胞如何與肺泡或間質巨噬細胞及樹突狀細胞協同，在發揮宿主防禦作用的同時限制對上皮的附帶損傷？

回答

健康呼吸道中，肺泡巨噬細胞是遠端氣腔最重要的第一線細胞哨兵，間質巨噬細胞和樹突狀細胞則佔據不同的組織生態位。它們平時持續吞噬吸入顆粒和少量微生物，卻盡量避免引發大規模發炎。一旦 TLR、NOD 樣受體等真正識別到感染，巨噬細胞、上皮細胞和其他局部細胞會產生 IL-1、TNF、CXCL 趨化因子、GM-CSF 和脂質趨化訊號，使中性粒細胞在短時間內大量進入感染部位。中性粒細胞通過吞噬、NADPH 氧化酶呼吸爆發、MPO 和顆粒蛋白殺菌；病原負荷很高或不易吞噬時，還可使用胞外誘捕網進行限制。

這種協同具有明顯的時序性。早期是巨噬細胞“呼叫”中性粒細胞；隨後中性粒細胞殺菌後產生的微生物碎片、IL-1 家族訊號、氧化脂質以及組織損傷相關分子又會反向影響巨噬細胞。如果感染尚未受控，這種放大是有利的；若病原已明顯下降仍繼續放大，就會轉為旁觀者損傷。樹突狀細胞在此過程中採集抗原並遷移到引流淋巴結，啓動病原特異性 T 細胞反應，使後續清除不必長期依賴最大強度的中性粒細胞發炎。

保護上皮的關鍵是主動消退。中性粒細胞完成任務後進入凋亡，由肺泡或間質巨噬細胞進行 efferocytosis。吞噬凋亡細胞會抑制巨噬細胞繼續產生強烈 NF-κB 型發炎因子，並增加 IL-10、TGF-β、Annexin A1 以及脂氧素和 resolvin 等促消退介質。與此同時，氣道表面液中的 α1-抗胰蛋白酶、SLPI 等抗蛋白酶限制中性粒細胞彈性蛋白酶，抗氧化系統降低 MPO/ROS 的毒性，DNase 可幫助清除過量 DNA-rich NET。

因此，決定結果的不是“有無中性粒細胞”，而是募集是否短暫、效應是否局部、清除是否及時。急性細菌或真菌感染中，中性粒細胞往往不可替代；而在重症肺炎、COPD 或部分氣喘中，持續的 CXCL8/GM-CSF、延遲凋亡、巨噬細胞 efferocytosis 障礙以及 NET/蛋白酶過量，會把原本的抗感染協同轉變為纖毛、緊密連接和肺泡上皮損傷。

Q04

問題

在穩態條件和第 2 型發炎期間，嗜酸性粒細胞如何在氣道黏膜中與巨噬細胞和樹突狀細胞相互作用？

回答

穩態氣道中的嗜酸性粒細胞數量較少，肺泡巨噬細胞和樹突狀細胞雖然持續接觸吸入抗原，但通常處於受抑制、低共刺激的狀態。巨噬細胞主要清除顆粒和凋亡細胞，樹突狀細胞進行抗原採樣；只要上皮沒有釋放強烈危險訊號，低水平 IL-10、TGF-β 和完整屏障就會限制嗜酸性粒細胞募集和活化。

2 型發炎首先改變的是上游“報警系統”。過敏原、蛋白酶或上皮損傷誘導 TSLP、IL-33、IL-25，進而活化 ILC2 並使樹突狀細胞上調 OX40L 等共刺激程序、遷移至淋巴結，促進 Th2 分化。ILC2/Th2 產生 IL-5，延長嗜酸性粒細胞生存；IL-4 和 IL-13 則促使上皮和基質細胞產生 CCL11、CCL24 以及在人類氣道中尤其重要的 CCL26，從而形成選擇性的嗜酸性粒細胞募集。

進入氣道後，嗜酸性粒細胞會反向塑造巨噬細胞和樹突狀細胞。IL-4/IL-13 可推動巨噬細胞進入偏修復的功能狀態，短期有利於恢復屏障；但持續存在時，同一類訊號也會促進黏液分泌、基質沈積和氣道重塑。嗜酸性粒細胞顆粒蛋白、白三烯、活性氧及細胞激素可損傷上皮，製造新的危險訊號，從而增強樹突狀細胞活化。某些顆粒核糖核酸酶，如 eosinophil-derived neurotoxin，在實驗中還能直接增強樹突狀細胞免疫刺激性。相反，巨噬細胞吞噬凋亡嗜酸性粒細胞後可增加 IL-10 和促消退介質，阻斷持續募集。

因此，穩態和 2 型發炎之間不是完全不同的細胞，而是同一網路被置於不同強度和持續時間的訊號環境。短暫的嗜酸性粒細胞—巨噬細胞交流可支持修復；持續的 IL-5/eotaxin、反復脫顆粒和長期 IL-4/IL-13 則把它變成慢性發炎和重塑迴路。上皮報警素和經典樹突狀細胞在這一通路中的核心地位較明確，而“嗜酸性粒細胞直接作為重要抗原呈遞者”的人體證據相對更具情境依賴性。

Q05

問題

在尿路黏膜中，早期抗菌防禦期間，中性粒細胞如何與單核細胞或巨噬細胞及樹突狀細胞協同？哪些機制可防止過度組織損傷？

回答

尿路早期抗菌防禦首先由尿路上皮和局部先天免疫細胞啓動。尿路致病菌接觸膀胱或下尿路黏膜後，通過 TLR 等模式識別通路誘導 IL-1、TNF 和中性粒細胞趨化因子。駐留巨噬細胞放大這一警報並幫助活化局部血管；感染較明顯時，CCR2 依賴的單核細胞也會進入組織並補充巨噬細胞群。中性粒細胞在數小時內大量募集，穿過黏膜甚至進入尿腔，通過吞噬、ROS、抗菌顆粒以及強刺激下的 NET 快速壓低細菌負荷。

單核細胞/巨噬細胞與中性粒細胞的功能並不重復。巨噬細胞可以吞噬細菌和細胞碎片，並在感染未完全控制時維持適度的細胞激素和趨化因子輸出；隨後又承擔清除凋亡中性粒細胞、終止發炎的工作。樹突狀細胞則負責抗原採樣和適應性免疫啓動，在單次、早期的非複雜性 UTI 中通常不如上皮—巨噬細胞—中性粒細胞軸突出，但在復發或複雜感染中，其對 T、B 細胞反應的影響會更重要。

抗菌效應本身也可能破壞尿路上皮。彈性蛋白酶、MPO 產生的氧化物、ROS 和 NET 相關組蛋白都可損傷緊密連接並暴露深層組織。正常情況下，細菌被快速清除後趨化訊號隨即下降，中性粒細胞進入凋亡並由巨噬細胞 efferocytosis。這個過程會使巨噬細胞減少繼續招募中性粒細胞的訊號，增加 IL-10、TGF-β 和修復相關程序。前列腺素、促消退脂質介質、局部抗氧化/抗蛋白酶系統以及尿路上皮較強的再生能力共同限制旁觀者損傷。

這套系統的本質是速度與克制之間的平衡。中性粒細胞不足會導致細菌擴增甚至上行感染；如果募集持續到病原負荷已很低，則可出現屏障破壞和症狀性發炎。很多精細的細胞互作來自動物 UTI 模型，人體中不同巨噬細胞亞群、具體趨化因子及 NET 的相對貢獻更為異質，不能把單一實驗機制視為所有患者都固定存在。

Q06
問題
在膀胱或下泌尿生殖道黏膜中，肥大細胞釋放的介質如何影響巨噬細胞和樹突狀細胞的功能？這一網路如何同時參與宿主防禦和發炎相關症狀？

回答
肥大細胞位於膀胱和下泌尿生殖道的血管、神經和上皮附近，因此少量細胞被活化就能迅速改變整個局部髓系網路。微生物產物、補體、IgE/FcεRI 訊號、上皮報警素以及神經肽都可誘導其釋放預存的組胺、胰蛋白酶樣蛋白酶、糜蛋白酶和 TNF，隨後再合成 PGD2、半胱氨酰白三烯、細胞激素和生長因子。結果是血管擴張、通透性上升、上皮訊號改變，並為其他白細胞進入組織創造條件。

巨噬細胞和樹突狀細胞表達多類相應受體。TNF、GM-CSF 可增強髓系細胞活化和存活；組胺對巨噬細胞/樹突狀細胞的作用取決於 H 受體亞型和細胞狀態，既可能增強也可能抑制某些發炎因子；肥大細胞蛋白酶可通過 PAR2 等受體影響髓系細胞及上皮。IL-4/IL-13 傾向於推動巨噬細胞進入修復型功能狀態，而 TNF/IL-1 豐富的環境則更容易維持發炎。對感染而言，這些變化能加速中性粒細胞募集，並提高樹突狀細胞成熟、遷移和抗原呈遞，從而有利於宿主防禦。

同一網路也能製造明顯症狀。組胺、tryptase、前列腺素以及 NGF 相關訊號可敏化膀胱感覺神經，降低 TRPV1、P2X 等通路的活化閾值；血管滲漏引起水腫，蛋白酶、TNF 以及其他髓系細胞產生的 ROS 可破壞尿路上皮屏障。屏障變“漏”後，尿液中的溶質和 ATP 更容易接觸黏膜下感覺末梢，於是形成尿急、疼痛與神經源性發炎的正反饋。巨噬細胞產生的 IL-1β、TNF、PGE2 又可繼續敏化神經並促進局部肥大細胞反應。

當巨噬細胞轉向 efferocytosis、IL-10、TGF-β 和促消退脂質介質，並支持上皮修復時，這一循環可以終止。需要強調的是，慢性膀胱疼痛綜合徵等疾病高度異質：部分患者確有肥大細胞增加或活化，但並非所有病例都由肥大細胞主導。因此，這一串擾網路是連接抗感染與發炎症狀的合理機制框架，而不是對所有慢性膀胱發炎的單一解釋。

Q07
問題
在急性腸黏膜發炎及其後的發炎消退階段，中性粒細胞與巨噬細胞如何透過募集訊號、細胞激素、凋亡細胞清除、脂質介質和組織修復程序相互影響？

回答
急性腸黏膜發炎早期，中性粒細胞和巨噬細胞構成一個迅速放大的迴路。屏障破壞後，上皮和駐留巨噬細胞釋放 IL-1、TNF、CXCL 趨化因子、C5a 和 LTB4 等脂質趨化訊號，首先把中性粒細胞招入病灶。中性粒細胞控制微生物的同時釋放 ROS、蛋白酶、發炎因子和 LTB4；這些物質又可吸引更多中性粒細胞，並產生 DAMP。與此同時，CCR2 依賴的單核細胞進入黏膜並分化為發炎性巨噬細胞，在危險訊號持續存在時繼續產生 TNF、IL-1β、IL-6 和趨化因子。

真正的發炎消退不是“所有訊號一起變小”，而是訊號性質發生轉換。完成任務的中性粒細胞進入凋亡，暴露磷脂酰絲氨酸等“eat-me”訊號。巨噬細胞通過 MerTK、Axl、TIM 家族以及整合素/橋接分子等系統進行 efferocytosis。吞噬凋亡中性粒細胞後，巨噬細胞抑制持續的 NF-κB 型發炎輸出，增加 IL-10、TGF-β，並啓動清除碎片和修復所需的代謝程序。更重要的是，及時吞噬可阻止中性粒細胞發生繼發性壞死，避免組蛋白、彈性蛋白酶、MPO 等高毒性內容物晚期大量外洩。

脂質介質也發生“類別切換”。早期前列腺素和白三烯促進血管活化和白細胞募集；進入消退期後，脂氧素、resolvin、protectin、maresin 等特殊促消退介質抑制新的中性粒細胞進入，並增強巨噬細胞 efferocytosis。中性粒細胞本身也可能參與這種脂質介質轉換，而不是單純被動退出。吞噬凋亡細胞後的巨噬細胞可產生 TGF-β、amphiregulin、VEGF 家族訊號和基質重塑酶，與上皮乾/祖細胞和基質細胞共同完成屏障修復。

如果任何一步失靈，保護性發炎就會轉為病理性發炎：NET、蛋白酶和 ROS 造成更多上皮破壞，新的 DAMP 又重新招募髓系細胞；efferocytosis 失敗則使死亡細胞壞死並持續刺激組織。反過來，過早壓制中性粒細胞又會削弱微生物清除。因而，比簡單的“M1→M2”二分更準確的描述是：多個巨噬細胞功能狀態隨時間連續轉換，只有殺菌、凋亡、efferocytosis、脂質介質切換和修復按正確順序銜接，發炎才真正消退。

Q08
問題
嗜酸性粒細胞來源的介質如何改變腸黏膜巨噬細胞的功能狀態和樹突狀細胞的活化？這些相互作用如何推動免疫耐受、組織修復與發炎之間的平衡發生變化？

回答
嗜酸性粒細胞可通過細胞激素、脂質介質和顆粒蛋白改變腸黏膜巨噬細胞與樹突狀細胞的狀態，但結果高度依賴於其所處的是穩態、修復還是強烈發炎環境。相對溫和時，嗜酸性粒細胞來源的 IL-4、IL-13 可促使巨噬細胞增強清除、基質重塑和修復相關功能；TGF-β、脂質介質和生長因子還可影響上皮、血管和基質。在一些實驗模型中，這種訊號與屏障恢復和 IgA 相關穩態相聯繫。

對樹突狀細胞來說，發炎狀態下的嗜酸性粒細胞影響更強。嗜酸性粒細胞來源的顆粒核糖核酸酶，例如 eosinophil-derived neurotoxin，在實驗系統中可作為 alarmin 增強樹突狀細胞活化；白三烯、ROS、發炎細胞激素以及顆粒蛋白造成的上皮損傷，也會間接創造一個“危險訊號豐富”的環境，提高共刺激和抗原呈遞。IL-4 還能影響樹突狀細胞偏向 2 型免疫的功能。嗜酸性粒細胞在發炎時可以表達 MHC-II 和共刺激分子，但其是否在人體腸道中承擔重要的直接抗原呈遞作用，證據明顯弱於經典樹突狀細胞。

因此，這一串擾可把黏膜平衡推向三個方向。若訊號受控，嗜酸性粒細胞可幫助巨噬細胞和樹突狀細胞維持對食物/共生菌的低反應狀態，並支持 IgA 和屏障；若組織處於損傷恢復期，IL-4/IL-13、TGF-β 可促進清除碎片和組織重建；若 IL-5/eotaxin 造成大量嗜酸性粒細胞積聚併發生脫顆粒，則顆粒毒性、半胱氨酰白三烯、ROS 和 alarmin 會活化更多樹突狀細胞和效應細胞，使“修復”轉為持續發炎甚至纖維化。

決定方向的關鍵不是固定的“嗜酸性粒細胞表型標籤”，而是組織訊號組合、活化強度和時間。IL-33、IL-25、TSLP 持續上升通常有利於 2 型發炎放大；嗜酸性粒細胞及時凋亡並被巨噬細胞吞噬，則有利於消退。部分穩態功能在小鼠中的證據較強，在人類中的定量貢獻仍需謹慎外推。

Q09

問題

在中性粒細胞性氣道發炎中，中性粒細胞與巨噬細胞如何透過細胞激素、脂質介質、中性粒細胞胞外誘捕網、凋亡細胞清除和代謝訊號相互影響？這種串擾如何決定發炎持續還是消退？

回答

中性粒細胞性氣道發炎之所以會持續，常常是因為巨噬細胞和中性粒細胞反複製造彼此需要的募集與生存訊號。活化的氣道巨噬細胞、上皮和樹突狀細胞產生 IL-1β、TNF、CXCL8/CXCL 家族趨化因子和 GM-CSF，招募中性粒細胞並延長其壽命。中性粒細胞隨後釋放 ROS、彈性蛋白酶、MPO、發炎因子和 LTB4，反過來增強巨噬細胞活化並造成上皮 DAMP。LTB4 特別擅長放大中性粒細胞之間的“接力式”募集，而 GM-CSF 等訊號會推遲本應較快發生的凋亡。

NET 是這一迴路的重要放大器。DNA 網架上攜帶組蛋白、彈性蛋白酶和 MPO，可捕獲微生物；但如果 NET 過量或清除不良，其組蛋白和蛋白酶可直接傷害上皮，並向巨噬細胞提供核酸和氧化蛋白危險訊號。不同模型中 TLR9、cGAS-STING、NLRP3 等通路均可能參與，但在氣喘、COPD 與感染中的相對權重並不相同。巨噬細胞由此產生更多 IL-1β 和趨化因子，就會形成“NET 越多—募集越多—NET 更容易繼續形成”的正反饋。

代謝狀態進一步決定發炎能否退出。發炎性巨噬細胞常增強糖酵解，並出現 succinate/HIF-1α 與 IL-1β 相聯繫的代謝程序；中性粒細胞則高度依賴糖酵解和磷酸戊糖途徑來維持遷移和 NADPH 氧化爆發。低氧、細胞外 ATP、乳酸和腺苷都能改變細胞壽命和細胞激素輸出。感染急性期這些適應是有利的，但在長期低氧、黏液瀦留的氣道中可轉為維持發炎的因素。

消退的核心是中性粒細胞凋亡後被巨噬細胞進行 efferocytosis。MerTK 等通路啓動後，巨噬細胞增加 IL-10、TGF-β 和促消退脂質介質，並向修復型代謝狀態轉換；脂氧素和 resolvin 同時減少新的募集。若 efferocytosis 障礙，凋亡細胞繼發壞死，蛋白酶和 DAMP 再次外洩。因此，持續發炎通常不是單一因子造成，而是“募集關閉失敗、NET/碎片清除失敗、代謝降檔失敗、efferocytosis 失敗”同時存在；真正的消退要求這些環節協同逆轉。

Q10

問題

在過敏性氣道發炎中，肥大細胞和嗜酸性粒細胞（在相關情境下包括嗜鹼性粒細胞）如何與樹突狀細胞和巨噬細胞相互作用，從而放大或限制第 2 型免疫反應？

回答

過敏性氣道發炎的核心是上皮報警素、樹突狀細胞和 2 型效應細胞之間的互相放大。過敏原或上皮損傷釋放 TSLP、IL-33、IL-25，活化 ILC2 並使經典樹突狀細胞上調 OX40L 等共刺激程序，隨後遷移到淋巴結誘導過敏原特異性 Th2。Th2 和 ILC2 產生 IL-4、IL-5、IL-13，為肥大細胞、嗜酸性粒細胞以及部分情境下的嗜鹼性粒細胞構建持續的 2 型細胞激素環境。

肥大細胞在 IgE/FcεRI 交聯後快速釋放組胺、tryptase、PGD2、半胱氨酰白三烯、TNF 和 2 型細胞激素。PGD2 經 CRTH2、白三烯經相應受體可同時吸引和活化嗜酸性粒細胞、ILC2 和 Th2。IL-5 延長嗜酸性粒細胞壽命，IL-4/IL-13 促進上皮產生 eotaxin。嗜酸性粒細胞繼而釋放白三烯、細胞激素、顆粒蛋白和 ROS，損傷上皮並製造更多 alarmin。嗜鹼性粒細胞可提供較早的 IL-4 並強化 Th2 環境，但把它們視為人體氣道中取代經典樹突狀細胞的主要抗原呈遞者，證據不足。

巨噬細胞可以把這條迴路推向兩種方向。IL-4Rα 訊號使其進入偏修復的功能狀態，有利於清除碎片和恢復屏障；若訊號持續，則也可促進黏液、基質沈積和氣道重塑，並通過 CCL17/CCL22 等途徑支持 2 型細胞聚集。相反，巨噬細胞高效吞噬凋亡嗜酸性粒細胞後，會增加 IL-10、TGF-β 和促消退脂質介質，降低進一步募集。隨著上皮危險訊號下降，樹突狀細胞也更容易回到低共刺激、偏調節的狀態。

所以，放大還是限制取決於迴路是否繼續由抗原和報警素驅動。持續過敏原暴露、屏障損傷、IgE 活化和 IL-5/eotaxin 軸會讓發炎自我維持；抗原減少、嗜酸性粒細胞凋亡、巨噬細胞 efferocytosis 和上皮修復則推動消退。這也解釋了為甚麼阻斷 IgE 或關鍵 2 型細胞激素的治療常常重塑整個細胞網路，而不僅僅是改變某一種粒細胞數量。

Q11

問題

在急性細菌性尿路感染期間，募集到局部的中性粒細胞和單核細胞來源巨噬細胞如何在病原體清除、細胞激素產生、凋亡細胞清除和黏膜修復過程中協同或競爭？

回答

急性細菌性 UTI 中，中性粒細胞和單核細胞來源巨噬細胞按不同時間尺度進入病灶，因此更像“前鋒與後勤/清場”的關係。尿路上皮和駐留髓系細胞感知細菌後釋放 CXCL 趨化因子、IL-1、TNF 等訊號，中性粒細胞最先大量到達，穿越尿路上皮後常成為尿液中最主要的發炎細胞。其優勢是速度：快速吞噬調理後的細菌，使用 NADPH 氧化酶 ROS 和抗菌顆粒，並在高負荷刺激時形成 NET。

CCR2 依賴的單核細胞一般稍晚進入，並分化成發炎性巨噬細胞。它們也能吞噬細菌，但更重要的是持續或重新塑造細胞激素環境、吞噬死亡細胞，並在感染後期推動組織修復。早期巨噬細胞產生 TNF、IL-1β 和趨化因子支持中性粒細胞募集；病原負荷下降後，同一巨噬細胞群可通過 efferocytosis 減少這些訊號並增加 IL-10、TGF-β 和修復因子。因此，吞噬凋亡中性粒細胞既是“垃圾清除”，也是把發炎程序切換為修復程序的訊號事件。

協同最理想的狀態是：中性粒細胞提供快速的滅菌壓力，巨噬細胞提供更持久的吞噬、監控和收尾。二者也可能發生競爭，例如爭奪被調理的細菌、補體組分以及局部氧和營養物；某些情境中巨噬細胞吞入但未完全殺死細菌，反而形成短暫的胞內存活位點。另一方向上，過強的中性粒細胞 ROS、蛋白酶可損傷周圍巨噬細胞和尿路上皮，甚至暴露新的細菌黏附位點。

黏膜真正恢復依賴於這種關係及時改變：趨化訊號下降，中性粒細胞退出，巨噬細胞完成 efferocytosis、基質重塑並與再生中的尿路上皮協同。如果中性粒細胞被過早抑制，滅菌不足；如果病原已控制後仍持續存在，NET、彈性蛋白酶、MPO 和 ROS 會維持症狀和損傷。動物模型對這種時序分工支持較強，但人體中各單核/巨噬細胞亞群的比例，以及其是否形成細菌暫存庫，仍具有明顯個體差異。

Q12

問題

在慢性膀胱黏膜發炎中，肥大細胞與巨噬細胞之間的串擾可能如何改變感覺神經訊號、血管通透性、上皮屏障功能以及發炎的持續或消退？

回答

慢性膀胱黏膜發炎中，肥大細胞和巨噬細胞可以形成一個連接神經、血管和上皮的正反饋環。肥大細胞緊鄰感覺神經和微血管，受到神經肽、補體、上皮訊號或其他危險刺激後釋放組胺、tryptase、前列腺素、白三烯、TNF 和生長因子。組胺與蛋白酶既提高血管通透性，也能直接降低感覺神經的興奮閾值；tryptase-PAR2、組胺 H1/H4 以及 NGF 相關訊號可增強疼痛、尿急等感覺通路。

當巨噬細胞維持在發炎狀態時，這種神經敏化會進一步放大。IL-1β、TNF、PGE2 和 ROS 可使傷害性感受神經更易興奮，並維持血管內皮活化。被活化的神經又釋放 substance P、CGRP 等神經源性介質，反過來影響肥大細胞、血管和巨噬細胞。CCL2/CCR2、CSF1 相關訊號可持續補充或維持單核/巨噬細胞，而 SCF/c-Kit 等通路幫助肥大細胞局部存活，於是發炎即使沒有強烈感染刺激也可能繼續。

上皮屏障把神經和發炎兩部分連在一起。肥大細胞蛋白酶、巨噬細胞發炎因子以及其他白細胞產生的 ROS 可破壞尿路上皮緊密連接，提高旁細胞通透性。受損上皮釋放 ATP 和 DAMP，使黏膜下感覺末梢更容易接觸尿液中的刺激物並活化 P2X 等受體。血管滲漏和水腫又增加局部壓力，進一步加重尿急、疼痛。由此形成“屏障更差—神經更敏感—肥大細胞/巨噬細胞更活躍”的循環。

消退依賴巨噬細胞轉向 efferocytosis、IL-10、TGF-β 和促消退脂質介質，並支持尿路上皮再生。屏障一旦恢復，尿液刺激物與感覺神經隔離，肥大細胞的持續活化也會下降。但如果 TGF-β 型修復過度，則纖維化和膀胱順應性下降本身又可能產生症狀。值得注意的是，慢性膀胱疼痛/間質性膀胱炎高度異質，肥大細胞並非所有患者的主導機制，因此應把這條軸視為一種可維持疾病的機制，而不是普遍適用的單因解釋。

Q13

問題

在潰瘍性結腸炎中，中性粒細胞募集、中性粒細胞胞外誘捕網形成、中性粒細胞代謝以及中性粒細胞-巨噬細胞串擾如何分別促進黏膜損傷或發炎消退？

回答

潰瘍性結腸炎中，中性粒細胞既是必要的抗微生物效應細胞，也是活動期黏膜損傷最直觀的參與者之一。發炎上皮、基質細胞和單核來源巨噬細胞產生 CXCL8 及其他 CXCR1/2 配體、IL-1、TNF 和集落刺激因子，驅動大量中性粒細胞進入結腸黏膜並穿過上皮形成隱窩炎和隱窩膿腫。吞噬和 ROS 有助於控制突破受損屏障的細菌，但細胞外彈性蛋白酶、MPO 氧化產物、MMP 和 ROS 又可擴大糜爛，形成更多微生物進入通道。

NET 進一步增加外源性毒性。DNA-組蛋白網架能捕獲微生物，但持續存在的 NET 同時攜帶彈性蛋白酶和 MPO，可直接傷害上皮、增強凝血和發炎，並作為 DAMP 活化巨噬細胞。核酸感知和 inflammasome 相關通路可能參與，包括不同模型中報道的 TLR9、cGAS-STING、NLRP3 等，但其在人體 UC 中的相對重要性並未完全統一。臨床樣本中 NET 標誌物與疾病活動度常有關聯，但“NET 是主要因果驅動”還是“嚴重中性粒細胞發炎的伴隨標誌”，仍需更強的干預性證據區分。

代謝狀態能延長這種效應。中性粒細胞高度依賴糖酵解和磷酸戊糖途徑，後者提供 NADPH 支撐氧化爆發；發炎黏膜本身低氧，HIF 相關生存程序可能延緩凋亡。乳酸、琥珀酸、細胞外 ATP 等發炎代謝物又可同時影響中性粒細胞和巨噬細胞。原本為急性殺菌服務的代謝適應，在慢性環境中就可能變成“讓細胞繼續活、繼續輸出毒性”的機制。

相反，發炎消退依賴巨噬細胞把中性粒細胞從“危險訊號”轉換為“待清除對象”。MerTK 等通路介導的 efferocytosis 會降低促炎因子並增加 IL-10、TGF-β、脂氧素、resolvin 以及組織修復程序；若清除失敗，凋亡細胞繼發壞死，DAMP 再次釋放。因而治療上的理想目標不是全面消滅中性粒細胞，而是減少過度募集和細胞外毒性，同時保留吞噬殺菌，並加速凋亡、efferocytosis 和屏障重建。

Q14

問題

在克隆氏症中，中性粒細胞、單核細胞來源巨噬細胞和樹突狀細胞之間失調的相互作用如何改變微生物處理、抗原呈遞、發炎持續以及上皮修復？

回答

克隆氏症中的髓系異常可以理解為：正常應當完成的“微生物感知—清除—抗原處理—發炎終止—上皮修復”鏈條沒有順利結束。屏障缺損使腸腔微生物持續進入固有層，中性粒細胞被招募進行清除；但 NOD2、ATG16L1 等與微生物識別、自噬和胞內處理有關的易感通路異常，可使微生物成分不易被徹底處理，於是髓系細胞長期暴露於刺激。

CCR2 依賴的單核細胞不斷進入組織，並可能無法像健康腸駐留巨噬細胞那樣獲得“高吞噬、低發炎反應”的穩態特徵，而是持續產生 TNF、IL-1β、IL-6、IL-23 和趨化因子。中性粒細胞的 ROS、蛋白酶和 NET 在控制微生物的同時擴大上皮損傷、釋放 DAMP。肉芽腫性病變中，巨噬細胞長期存在並持續處理難以清除的微生物或顆粒，也可成為發炎維持的一部分。

樹突狀細胞把這種持續的先天刺激連接到適應性免疫。大量微生物抗原、IL-12/IL-23 家族訊號和較強共刺激更容易促進 Th1/Th17 相關反應，而不是黏膜耐受。活化 T 細胞返回組織後產生 IFN-γ、IL-17 等介質，又刺激巨噬細胞和上皮產生更多趨化因子。於是形成“清除不徹底→抗原暴露更多→適應性發炎更強→屏障更破→微生物進入更多”的閉環。

上皮修復則需要巨噬細胞 efferocytosis、amphiregulin/TGF-β 等生長與修復訊號、上皮乾細胞再生和受控的基質重塑。持續 TNF/IL-1、蛋白酶和低氧會妨礙這些過程；反過來，過度 TGF-β 型修復又可導致狹窄和纖維化。抗 TNF 的臨床有效性支持發炎性髓系細胞激素在

病程中具有因果意義，但克隆氏症高度異質：不同患者中“先天殺菌缺陷、單核/巨噬細胞過度發炎、樹突狀細胞抗原呈遞和纖維化修復”的權重並不相同，因此不能只用“免疫過強”概括。

Q15

問題

在重度中性粒細胞性氣喘中，中性粒細胞、巨噬細胞和樹突狀細胞如何形成自我維持的發炎迴路？中性粒細胞胞外誘捕網、發炎小體訊號、微生物訊號以及糖皮質激素抗性可能分別發揮什麼作用？

回答

重度中性粒細胞性氣喘常由“上皮—巨噬細胞/樹突狀細胞—Th17—中性粒細胞”迴路維持，而不是經典的嗜酸性 2 型發炎。污染物、感染、DAMP 或菌群失衡產生的微生物訊號可活化 TLR/NOD、NF-κB 通路，使上皮和巨噬細胞釋放 IL-1β、TNF、CXCL8、GM-CSF。樹突狀細胞和巨噬細胞又可通過 IL-6/IL-23 環境支持 Th17；IL-17 反過來誘導上皮產生 CXCL8、G-CSF，進一步募集並延長中性粒細胞壽命。中性粒細胞的彈性蛋白酶、ROS 和 DAMP 又重新刺激上皮和巨噬細胞，形成自維持迴路。

NET 可以把這一迴路再放大一層。細胞外 DNA、組蛋白、MPO 和彈性蛋白酶增加黏液黏稠度、損傷上皮，並向巨噬細胞和樹突狀細胞提供先天免疫刺激。在多種模型中，NET 成分和細胞外 ATP 可促進 NLRP3 inflammasome 和 IL-1β，從而進一步支持中性粒細胞/Th17 發炎。但 NLRP3 並非所有重症氣喘患者都同等重要；所謂“中性粒細胞性氣喘”本身包括感染相關、吸煙/污染相關、肥胖相關及治療塑形後的多個內型。

微生物訊號可成為持續的外源驅動。慢性細菌定植、反復感染或氣道菌群組成改變持續提供 TLR 配體，使 IL-1/CXCL8 軸難以關閉。這可能解釋部分患者為何對大環內酯類治療有反應，但其作用不一定僅來自抗菌，還可能包括降低中性粒細胞趨化和巨噬細胞發炎輸出；同時必須考慮耐藥和菌群擾動。若真正存在持續感染，過度抑制中性粒細胞反而可能危險。

糖皮質激素抗性也具有多因素基礎。中性粒細胞/Th17 發炎一般比嗜酸性 2 型發炎更不敏感；氧化應激、p38 MAPK 等可影響糖皮質激素受體功能，激素本身還可能延遲中性粒細胞凋亡。HDAC2 下降被提出為氧化應激相關耐藥機制之一，但其在人類氣喘中的作用強度並非所有研究都一致。總體上，只有當募集、NET/DAMP、inflammasome、微生物刺激和消退失敗同時被打斷，才可能真正終止這一自我維持網路，同時還要盡量保留抗細菌防禦。

Q16

問題

在嗜酸性粒細胞性氣喘中，嗜酸性粒細胞、肥大細胞以及在相關情境下的嗜鹼性粒細胞，如何與巨噬細胞、樹突狀細胞和氣道上皮相互作用，從而啟動、放大並最終促使第 2 型發炎消退？

回答

嗜酸性粒細胞性氣喘的啓動通常發生在氣道上皮。過敏原、病毒、蛋白酶和污染物使上皮釋放 IL-33、TSLP、IL-25，活化 ILC2 並條件化經典樹突狀細胞。樹突狀細胞遷移到淋巴結後誘導過敏原特異性 Th2；Th2 與 ILC2 產生 IL-5 維持嗜酸性粒細胞，同時 IL-4/IL-13 促使上皮產生 eotaxin、黏液和其他 2 型趨化訊號。肥大細胞和嗜鹼性粒細胞則通過 IgE 和細胞激素通路進入同一網路。

放大階段，各細胞開始互相餵養訊號。IgE 交聯的肥大細胞釋放組胺、PGD2 和半胱氨酰白三烯，既引起支氣管收縮，也通過 CRTH2 等通路吸引嗜酸性粒細胞、ILC2 和 Th2。嗜鹼性粒細胞可快速提供 IL-4，增強 2 型偏向，但經典樹突狀細胞仍是主要專業抗原呈遞細胞。嗜酸性粒細胞釋放白三烯、IL-4/IL-13、顆粒蛋白和 ROS，造成上皮損傷並誘導更多報警素；IL-4Rα 訊號同時使巨噬細胞進入修復/重塑型功能狀態。

氣道上皮本身是迴路的重要主動節點。IL-13 誘導杯狀細胞化生和 MUC5AC 等黏液基因，上皮 eotaxin 決定嗜酸性粒細胞在組織中的定位。只要損傷持續，TSLP/IL-33 就繼續活化樹突狀細胞和 ILC2。慢性狀態下，嗜酸性粒細胞和肥大細胞來源的 TGF-β、蛋白酶、脂質介質可促進平滑肌改變和基底膜下纖維化，使反復發炎轉化為結構性氣道重塑。

消退需要上游抗原/報警素訊號下降、嗜酸性粒細胞進入凋亡並被巨噬細胞高效 efferocytosis。此時巨噬細胞增加 IL-10 和促消退脂質介質，調節性 T 細胞以及低活化樹突狀細胞減少新的 Th2 啓動。由於各節點緊密連接，阻斷一個上游細胞激素往往同時改變嗜酸性粒細胞遷移、肥大細胞活化、巨噬細胞狀態和上皮行為。核心通路較明確，而嗜鹼性粒細胞的必要性及嗜酸性粒細胞直接抗原呈遞在人體中的貢獻更具情境依賴性。

Q17

問題

在復發性尿路感染中，既往感染如何透過訓練性免疫、免疫耐受或組織重塑改變膀胱黏膜中的中性粒細胞和單核細胞/巨噬細胞反應？目前對這些機制各自的證據強度如何？

回答

既往 UTI 可能通過三類不同機制改變下一次感染時的中性粒細胞和單核/巨噬細胞反應：訓練性免疫、免疫耐受和組織重塑。三者不能混為一談，因為它們對二次反應的預測甚至可能相反。訓練性免疫強調長期存在的代謝和表觀遺傳重編程，例如發炎/抗菌基因染色質開放度增加、糖酵解增強以及甲羥戊酸或線粒體代謝變化，使後續刺激時巨噬細胞更快產生趨化因子、更強吞噬並更早募集中性粒細胞。成熟中性粒細胞壽命很短，因此真正持續數月的“中性粒細胞記憶”更可能來自骨髓祖細胞重編程或膀胱微環境改變，而不是同一批成熟細胞自身保存記憶。

免疫耐受則可能產生相反結果。反復 TLR/LPS 刺激可使部分 TNF、IL-1 和趨化因子基因變得低反應，同時保留某些抗菌或修復通路。對膀胱來說，這也許減少二次感染時的組織損傷，卻可能因中性粒細胞募集不足而給細菌再次擴增機會。經典 endotoxin tolerance 在其他系統證據充分，但在復發性 UTI 患者膀胱髓系細胞中的直接證明仍有限，因此應視為合理推斷而非已確認的主導機制。

組織重塑目前具有更強的實驗支持。既往感染可改變尿路上皮分化、屏障性質、基質細胞趨化因子表達以及局部免疫細胞生態位；持續抗原或胞內細菌庫也能改變組織的基線發炎閾值。因此，第二次感染可能被“預先敏化”而更劇烈，也可能因為屏障和局部免疫環境已強化而更快清除。即使單個巨噬細胞沒有發生自主訓練，細胞數量、定位和刺激閾值的改變也足以重塑總體反應。

證據強度因此不均衡：感染誘導的上皮/組織重塑在動物模型中較為可靠，也符合人類復發病程；單核/巨噬細胞的訓練性免疫在其他感染背景已被充分證明，在尿路中越來越具有可信度，但直接人體證據仍少；成熟中性粒細胞自身的長期記憶最不可信。耐受在機制上同樣合理，卻尚缺乏復發性 UTI 中的定量證據。真正區分三者需要縱向人體取樣、單細胞表觀組學和二次刺激功能實驗。

Q18

問題

在膀胱癌中，腫瘤相關中性粒細胞和腫瘤相關巨噬細胞如何在黏膜腫瘤微環境中相互作用，並影響抗原呈遞、T 細胞活性、血管新生、腫瘤侵襲以及免疫治療反應？

回答

膀胱癌中的腫瘤相關巨噬細胞（TAM）和腫瘤相關中性粒細胞（TAN）可形成互相強化的免疫抑制和組織重塑網路。腫瘤/基質細胞通過 CCL2、CSF1 等訊號招募並維持巨噬細胞，通過 CXCL8-CXCR2、G-CSF 等吸引中性粒細胞。被腫瘤環境重編程後，巨噬細胞可降低 IL-12 和有效抗原呈遞，提高 IL-10、TGF-β、ARG1 和 PD-L1；中性粒細胞也可通過 ARG1、ROS、蛋白酶和 NET 獲得 T 細胞抑制能力。這裡更適合用連續功能狀態描述，而不是把所有細胞硬分成“M1/M2”或“N1/N2”。

二者可從多個層面壓制 T 細胞。TAM 的 IL-10/TGF-β、PD-L1 降低 T 細胞活化；TAN 的 ROS 和精氨酸耗竭損害 TCR 訊號和增殖；兩類細胞還可幫助募集 Treg 或其他抑制性髓系細胞。若樹突狀細胞成熟和抗原呈遞同時受抑制，則初始抗腫瘤 T 細胞啓動也會變差。反過來，具有較高 MHC-II、共刺激和 IL-12 的巨噬細胞以及處於適當發炎狀態的中性粒細胞也能支持抗腫瘤免疫，因此關鍵在於腫瘤微環境把它們推向哪一端。

血管新生與侵襲同樣是協同輸出。TAM 產生 VEGF、MMP、TGF-β，促進血管和基質重塑；TAN 的 MMP9、彈性蛋白酶、VEGF 相關訊號和 NET 可在實驗中幫助打開組織通路、捕獲循環腫瘤細胞並促進侵襲。低氧通過 HIF 等機制進一步強化兩類髓系細胞的免疫抑制和促血管新生狀態。

這也影響免疫治療。高外周中性粒細胞/淋巴細胞比值或髓系細胞豐富常與較差結局相關，但這種關聯並不能證明單一因果通路。髓系 PD-L1、抗原呈遞不足和 T 細胞排斥都可能降低檢查點抑制劑效果。另一方面，膀胱內 BCG 恰好證明局部強烈的中性粒細胞和巨噬細胞活化也可以成為抗腫瘤機制的一部分。因此，治療目標不應是簡單清空髓系細胞，而應選擇性減少免疫抑制、促血管新生和 NET-rich 狀態，同時保留吞噬、抗原呈遞以及尿路局部抗菌能力。

Q19

問題

在潰瘍性結腸炎中，哪些具有機制學合理性的策略可能抑制致病性中性粒細胞募集或中性粒細胞胞外誘捕網相關損傷，同時盡量保留抗微生物防禦和巨噬細胞介導的修復？請說明預期獲益、潛在權衡及證據缺口。

回答

在 UC 中，理想策略應更多壓制中性粒細胞的“細胞外組織毒性”而不是吞噬殺菌本身。第一類思路是部分阻斷 CXCL8-CXCR1/2 募集軸，減少中性粒細胞進入隱窩，從而降低 ROS、蛋白酶和 NET 負擔。但如果全身、強力抑制 CXCR2，會削弱細菌感染時的募集能力；目前這類藥物也並未成為 UC 的確立治療。結腸局部給藥、短療程或只用於具有高 NET/高中性粒細胞生物標誌的患者，理論上更能保留全身抗菌能力。

第二類是直接限制 NET 相關損傷。PAD4 抑制可減少部分 NETosis 所需的染色質去凝聚，但並非所有 NET 都依賴 PAD4，而且 PAD4 也並非只在中性粒細胞中有作用。DNase 可切斷 DNA 網架，卻可能把仍有活性的彈性蛋白酶、MPO 和組蛋白釋放到組織液中，因此單純“把網剪碎”未必足夠；DNase 與抗蛋白酶、組蛋白中和或 MPO 抑制聯用在機制上更合理。彈性蛋白酶/MPO 抑制可減少組織毒性，但抑制過強同樣會犧牲微生物殺傷。

第三類策略不是阻止中性粒細胞來，而是加速正常消退。Annexin A1、脂氧素/resolvin 等通路可減少後續募集，同時提高巨噬細胞 efferocytosis；MerTK 相關清除通路增強後，可保留感染早期的中性粒細胞殺菌，卻縮短後期組織中毒性階段。促進巨噬細胞修復和上皮屏障恢復，也能從源頭減少持續驅動中性粒細胞的微生物刺激。不過，如果把“修復型”訊號推得過強，也可能出現免疫抑制或纖維化。

現有 IBD 治療通過降低 TNF、IL-23 相關發炎和上皮趨化因子，常可間接減少中性粒細胞，但並非真正的“選擇性保留抗菌功能”方案。最大的證據缺口是：NET、CXCR2 和促消退靶點多數仍停留在臨床前或尚無明確 UC 臨床獲益。未來試驗不能只看中性粒細胞數量，還應同時評估黏膜細菌控制、NET 負荷、巨噬細胞 efferocytosis、上皮愈合和感染率。最可行的方向可能是局部、可逆、按發炎狀態精准調節，而不是系統性清除中性粒細胞。

Q20

問題

如何利用腸道巨噬細胞和粒細胞的代謝或表觀遺傳重編程來恢復發炎性腸病中的黏膜穩態？哪些機制可能導致療效喪失或非預期的免疫抑制？

回答

代謝或表觀遺傳重編程的優勢在於“改變髓系細胞做甚麼”，而不是直接刪除整個細胞群。發炎性腸巨噬細胞常增強糖酵解、mTOR 和 succinate-HIF-1α 相關程序，從而支持 IL-1β、TNF 和趨化因子輸出。理論上，可通過活化 AMPK、改善線粒體氧化代謝、增強 Nrf2/itaconate 抗氧化軸或降低過度糖酵解，把巨噬細胞推向更強 efferocytosis 和修復狀態。表觀遺傳層面，BET 蛋白、組蛋白乙酰化/去乙酰化和甲基化可決定發炎基因的可及性，因此也可能用來清除“訓練性發炎”留下的持續轉錄記憶。

但粒細胞需要更謹慎。中性粒細胞高度依賴糖酵解和磷酸戊糖途徑完成遷移、存活以及 NADPH 氧化酶依賴的殺菌。全局抑制糖酵解或 NADPH 生成雖然可能減少 ROS 損傷，卻可能同時毀掉抗細菌能力。更合理的目標是選擇性限制過度 NET、胞外脫顆粒或異常延長的壽命，而保留吞噬體內的殺菌。HIF 相關持續生存、特定脂質代謝和 NET 染色質程序都是候選，但在 IBD 中大多仍屬機制性或臨床前研究。

療效喪失有多種來源。腸單核細胞持續從血液補充，新來的發炎細胞會稀釋已經被重編程的局部巨噬細胞；TNF、GM-CSF、IL-1、IL-23 等強細胞激素也可覆蓋代謝干預的效果。微生物群代謝物可能把相同通路推向相反方向，代謝冗余、疾病異質性以及藥物難以進入嚴重發炎/纖維化區域都會形成逃逸。

意外免疫抑制是最大風險。過度抑制糖酵解、mTOR、ROS 或染色質開放性，可同時降低吞噬、inflammasome、細胞激素募集和抗原呈遞；過度強化 TGF-β 型修復又可能促進纖維化，並潛在降低對腫瘤前病變的免疫監視。系統性表觀遺傳藥物還會影響淋巴細胞和上皮乾細胞。因此，最有前景的是細胞/組織特異的遞送、可逆劑量，並同步監測巨噬細胞狀態、中性粒細胞殺菌能力和感染風險，而不是全身性地"重置"所有髓系細胞。

Q21

問題

能夠改變第 2 型細胞激素訊號的生物製劑，除降低嗜酸性粒細胞數量外，還可能如何重塑氣道黏膜中的嗜酸性粒細胞-肥大細胞-巨噬細胞-樹突狀細胞網路？

回答

改變 2 型細胞激素訊號的生物制劑之所以能重塑整個氣道網路，是因為 IL-5、IL-4/IL-13 以及更上游的上皮細胞激素並不只是控制嗜酸性粒細胞數量。抗 IL-5 或抗 IL-5R 治療會減少嗜酸性粒細胞生成、存活和組織滯留，同時也減少其來源的白三烯、顆粒蛋白、TGF-β、IL-4/IL-13 等二級訊號，因此上皮損傷、巨噬細胞重塑和樹突狀細胞受危險訊號刺激都會下降。Benralizumab 通過 IL-5Rα 介導的細胞毒作用還可減少嗜鹼性粒細胞，從而同時改變一個潛在的早期 IL-4 來源。

IL-4Rα 阻斷的網路效應更廣，因為同時阻斷 IL-4 和 IL-13。氣道上皮產生更少 eotaxin、黏液和 2 型發炎相關 NO 訊號；B 細胞 IgE 類轉換支持下降；巨噬細胞較少被持續推向 IL-4/IL-13 條件化的修復/重塑狀態；樹突狀細胞周圍的趨化因子和組織環境也會變化。臨床上可出現短暫外周血嗜酸性粒細胞升高，因為組織遷入降低的速度可能快於骨髓產生下降，這正說明"血細胞計數"並不能完整代表黏膜網路發生了甚麼。

更上游的 TSLP 阻斷可同時降低樹突狀細胞活化、ILC2 刺激以及支持肥大細胞/嗜鹼性粒細胞的上皮訊號，因此可能影響比單純嗜酸性粒細胞更廣的發炎維度。IL-33/ST2 軸從機制上也可影響 ILC2、肥大細胞和嗜酸性粒細胞，但其臨床確立程度不如已經廣泛應用的 IL-5、IL-4Rα 和 TSLP 靶向治療。

這些變化可改善上皮屏障並減少巨噬細胞"清理發炎殘骸"的負擔，但也可能改變正常修復和抗寄生蟲免疫。尤其是肥大細胞的生存主要不依賴 IL-5，因此即使嗜酸性粒細胞幾乎被清除，肥大細胞仍可能存在。總體上，抗 IL-5 更偏向瓦解嗜酸性粒細胞支路，IL-4Rα 阻斷同時重塑上皮、B 細胞和髓系細胞，而上游報警素阻斷則有潛力重構樹突狀細胞—ILC2—粒細胞的整個黏膜網路。

Q22

問題

在中性粒細胞性氣喘中，哪些針對髓系細胞的策略可能中斷中性粒細胞-巨噬細胞發炎迴路，同時保留抗細菌防禦？這些策略所依據的機制中，哪些已有臨床證據，哪些仍主要處於臨床前階段？

回答

中性粒細胞性氣喘的治療難點在於：造成氣道損傷的中性粒細胞—巨噬細胞迴路，也是抗細菌防禦的一部分。因此更合理的原則是"部分、局部、按狀態阻斷"，而不是系統性清除髓系細胞。CXCR2 拮抗劑可阻斷 CXCL8 家族趨化訊號，明顯減少氣道中性粒細胞。臨床研究已證明其能降低痰中性粒細胞，說明靶點確實被命中，但對氣喘臨床結局的改善並不穩定，而且存在感染風險；因此它屬於"機制得到人體驗證、但並非標準治療"。

長期大環內酯類，尤其是選定持續性/重症氣喘患者中的 azithromycin，具有較強的臨床減輕急性加重證據，而且確實會影響髓系細胞：可降低部分中性粒細胞趨化、巨噬細胞細胞激素及 inflammasome 相關輸出。但其獲益究竟有多少來自抗菌、多少來自免疫調節並不完全清楚，同時會帶來耐藥、QT 風險和菌群改變，因此不能把它視為一個"純粹、精准"的中性粒細胞—巨噬細胞重編程治療。抗 TSLP 等上游生物制劑已用於重症氣喘，但它們並非髓系細胞特異靶向。

更選擇性的方案大多仍屬臨床前或早期轉化。NLRP3/IL-1 抑制可理論上削弱巨噬細胞與中性粒細胞互相放大的迴路，但過強抑制 IL-1 會削弱感染防禦。DNase、PAD4 抑制、MPO/彈性蛋白酶抑制以及組蛋白中和等 NET 靶向方法試圖減少細胞外毒性、保留吞噬功能，但在人類中性粒細胞性氣喘尚缺乏確立療效。促進 MerTK/efferocytosis、使用 resolvin 等促消退介質則更符合"讓中性粒細胞完成早期殺菌後盡快退出"的思路，目前仍主要實驗性。

未來最有可能成功的設計是吸入局部給藥 + 生物標誌物分型 + 同步監測抗菌能力。慢性細菌定植患者與無感染、主要由 IL-1/Th17 驅動的患者不能採用同一強度的中性粒細胞抑制。到目前為止，還沒有一種治療是因為"明確切斷中性粒細胞—巨噬細胞迴路且已證明保留抗細菌防禦"而成為臨床標準，這正是該領域最重要的轉化缺口。

Q23

問題

訓練性免疫或黏膜疫苗策略是否可能透過有益地重編程單核細胞/巨噬細胞和中性粒細胞來減少復發性尿路感染？請解釋其擬議機制、潛在的組織特異性風險以及目前證據的侷限。

回答

訓練性免疫或黏膜疫苗有可能通過"提高下一次感染時的啓動速度"減少復發性 UTI，而不是讓膀胱長期處於發炎狀態。訓練性免疫更可能發生在壽命較長的單核/巨噬細胞祖細胞或組織巨噬細胞中：NOD、C 型凝集素等模式識別訊號可誘導組蛋白可及性、糖酵解、甲羥戊酸和線粒體代謝的持久變化，使二次接觸細菌時更快產生趨化因子、更有效吞噬並更早招募中性粒細胞。成熟中性粒細胞壽命短，所以長期"訓練"更可能發生在骨髓祖細胞或膀胱組織環境，再讓新生成的中性粒細胞表現出更易應答的狀態。

黏膜疫苗則增加適應性特異性。樹突狀細胞呈遞細菌抗原後，可誘導尿路相關的 T 細胞、抗體以及 IgA/IgG 反應；疫苗佐劑又可能同時訓練先天免疫。口服或舌下的細菌裂解物、多價細菌疫苗在一些臨床研究中顯示復發減少訊號，但實際保護究竟由適應性免疫、訓練性免疫還是非特異性反復暴露共同造成，目前很難完全拆分。

組織特異性風險不可忽視。若膀胱被"訓練"得過度敏感，低水平細菌暴露也可能引起過強 CXCL8、中性粒細胞 ROS、NET 和疼痛性發炎。反復先天刺激還可能產生免疫耐受而不是訓練，使 TNF/IL-1 反應被壓低，反而利於細菌持續。對尿路上皮乾細胞和基質的長期重塑可能提高屏障能力，也可能帶來纖維化、感覺神經高敏或有利於胞內細菌庫持續。適應性免疫的交叉反應和微生物群改變同樣需要監測。

目前的證據更支持"概念可行"，而不是已經證明一條明確的人體髓系重編程通路。復發性 UTI 的免疫預防確有臨床訊號，但產品、試驗設計、監管狀態和終點差異很大；直接證明人膀胱巨噬細胞存在保護性訓練染色質狀態、證明骨髓層面的中性粒細胞祖細胞重編程並與復發減少有因果關係的研究仍少。真正有說服力的研究應把隨機臨床結局與膀胱/尿液單細胞組學、表觀遺傳和二次刺激功能實驗結合起來。

Q24
問題
如何透過選擇性重編程腫瘤相關巨噬細胞和中性粒細胞來增強膀胱癌的抗腫瘤免疫，同時盡量減少對保護性尿路黏膜免疫的破壞？請比較不同機制策略及其主要轉譯障礙。

回答
膀胱癌中選擇性重編程 TAM 和 TAN 的核心，不是把髓系細胞整體清除，而是把它們從免疫抑制、促血管新生、促侵襲狀態推向支持抗原呈遞和 T 細胞殺傷的狀態，同時保留尿路平時所需的吞噬和抗菌能力。對巨噬細胞而言，可考慮功能活化而非純耗竭：CD40、TLR 或 STING 激動，抑制 PI3Kγ 類免疫抑制訊號，或阻斷 CD47-SIRPα"別吃我"通路，都可提高腫瘤吞噬、MHC-II/共刺激和 IL-12 型反應。CSF1R 抑制可減少某些抑制性 TAM，但過度耗竭也會犧牲組織修復和抗感染吞噬。

中性粒細胞可從"募集"和"效應狀態"兩端干預。部分阻斷 CXCL8-CXCR2 可減少腫瘤促進型中性粒細胞和髓系抑制細胞進入；TGF-β 軸調節有可能把 TAN 推向更發炎性、抗腫瘤的狀態；PAD4/NET、MPO 或彈性蛋白酶靶向則試圖減少 NET 促進侵襲和血管改變的作用。但 CXCR2、ROS 和顆粒酶也是細菌防禦的重要組成，因此慢性全身抑制風險很高。

膀胱癌提供了一個非常有價值的選擇性優勢：可以膀胱內給藥。局部免疫激動劑、納米載體或腫瘤微環境響應制劑可使腫瘤髓系細胞獲得高暴露，同時減少全身中性粒細胞和巨噬細胞受到影響。BCG 已經是一個現實的"概念驗證"：它通過強烈局部先天免疫，包括中性粒細胞和巨噬細胞活化，產生抗腫瘤效應。這反而提示不能粗暴耗竭髓系細胞。若重編程能先改善抗原呈遞、減少 T 細胞排斥，再與 PD-1/PD-L1 阻斷聯合，機制上更有吸引力。

主要轉化障礙是髓系細胞高度可塑、空間異質且缺少穩定標誌物。小鼠 TAM/TAN 標誌並不能直接映射到人膀胱癌；同一細胞數小時內就可換狀態。局部藥物進入壞死/大體積腫瘤的能力、其他抑制性髓系細胞的代償募集、尿路感染風險、黏膜修復受損以及與 BCG 的相互作用都需要解決。最可信的路線因此是：局部、可逆、由腫瘤和尿液生物標誌物引導的功能重編程，而不是永久耗竭整個髓系譜系。

IV. Model D

English (EN)

Anonymous Model D | English
Anonymous Model D
English
24 standardized questions

Q01
Question
At the healthy intestinal mucosa, how do neutrophils contribute to barrier defense while interacting with resident macrophages and dendritic cells, and what mechanisms normally limit neutrophil-driven tissue injury?

Response
At steady state, neutrophils are not a dominant population in the intestinal lamina propria; their value lies in being rapidly deployable when epithelial integrity is threatened. Microbial products or epithelial stress induce chemokines such as CXCL1/CXCL2 in mice and CXCL8 in humans, together with complement and lipid chemoattractants, drawing neutrophils across the microvasculature and, when necessary, through the epithelium. Once recruited, they phagocytose bacteria and fungi, generate compartmentalized reactive oxygen species through NADPH oxidase, release antimicrobial granule proteins, and can form extracellular traps. These functions are most protective when they are spatially confined to the site of barrier breach.

Resident intestinal macrophages and dendritic cells shape this response. Homeostatic macrophages continuously clear microbes and apoptotic material yet are relatively hyporesponsive to commensal stimulation; they produce IL-10 and other regulatory signals that suppress unnecessary inflammatory amplification. When a breach occurs, macrophage- and epithelial-derived IL-1, TNF and chemokines support neutrophil recruitment, while dendritic cells sample antigen and determine whether adaptive priming is required. Neutrophils can in turn modify antigen availability, release alarmins and proteases, and influence macrophage/DC activation, but in health these interactions are short-lived rather than self-sustaining.

Tissue protection depends on an active shutdown program. Neutrophils undergo apoptosis after completing antimicrobial work and are engulfed by macrophages through efferocytosis pathways involving receptors such as MerTK and AXL. Efferocytosis suppresses further TNF/IL-1 production and favors IL-10, TGF-β and pro-repair programs. Specialized pro-resolving lipid mediators, adenosine, annexin A1, epithelial anti-proteases and mucus also limit protease- and oxidant-mediated injury. Tight control of recruitment and rapid clearance prevent persistent NETs, extracellular histones, elastase and myeloperoxidase from damaging crypt epithelium.

Thus, neutrophils are beneficial when recruitment is proportional, killing is localized, and macrophage-mediated clearance follows promptly. The same effector systems become pathogenic when recruitment persists, neutrophils undergo secondary necrosis, NET material accumulates, or macrophage efferocytosis fails. This balance is central to distinguishing physiologic barrier defense from the neutrophil-rich tissue injury seen in inflammatory bowel disease.

Q02

Question

At the healthy intestinal mucosa, how do eosinophils contribute to homeostasis and communicate with macrophages or dendritic cells, and how do these roles differ from eosinophil functions during inflammation?

Response

Intestinal eosinophils are unusual because substantial numbers are present in the lamina propria even without overt inflammation. Their maintenance depends in part on IL-5 and eotaxin-family chemokines, especially CCL11, but the steady-state population behaves differently from eosinophils recruited into an actively inflamed mucosa. In health, eosinophils participate in immune surveillance, epithelial and stromal homeostasis, extracellular-matrix turnover, and regulation of local humoral immunity. Experimental data support roles in plasma-cell survival and IgA-associated mucosal responses, although the magnitude and necessity of these functions vary by species and intestinal segment.

Communication with macrophages and dendritic cells is bidirectional. Eosinophil-derived IL-4 and IL-13 can bias macrophages toward tissue-repair and remodeling programs, while macrophage-derived chemokines and growth factors influence eosinophil recruitment and survival. Eosinophils also release lipid mediators, TGF-β and granule-associated molecules that can alter antigen-presenting-cell activation. Dendritic cells provide an additional bridge to adaptive immunity: depending on the context, eosinophil products may enhance antigen uptake and costimulation or help maintain a more regulatory local environment. It is more accurate to view these states as a spectrum than as a simple “M1/M2” switch.

During inflammation, the same cells can become strongly pathogenic. Increased IL-5, GM-CSF, CCL11/CCL24/CCL26 and epithelial alarmins prolong eosinophil survival and promote activation. Degranulation releases major basic protein, eosinophil cationic protein and eosinophil peroxidase; leukotrienes, cytokines and reactive oxygen species can amplify vascular leak, epithelial injury and neuromuscular symptoms. Eosinophils may also promote fibroblast activation and chronic remodeling.

The clinically relevant distinction is therefore not the presence of eosinophils alone but their activation state, localization and persistence. Homeostatic eosinophils generally operate within regulatory and repair networks with macrophages and dendritic cells. In inflammatory settings, sustained type 2 cytokine signaling, excessive degranulation and prolonged cell survival can convert those same networks into drivers of tissue injury. Human intestinal eosinophil biology remains context dependent, so mechanistic findings from mouse models should not automatically be interpreted as universal human functions.

Q03

Question

At the healthy respiratory mucosa and during an acute microbial challenge, how do neutrophils coordinate with alveolar or interstitial macrophages and dendritic cells to provide host defense while limiting collateral epithelial injury?

Response

In the healthy lung, alveolar macrophages are the principal myeloid sentinels at the air–liquid interface and actively suppress unnecessary inflammation caused by inhaled particles and commensal signals. Neutrophils are normally sparse in the alveolar space. During an acute bacterial or fungal challenge, pattern-recognition signaling in alveolar macrophages, interstitial macrophages and airway epithelium induces IL-1, TNF, CXCL1/CXCL2/CXCL5 and, in humans, CXCL8. These signals recruit neutrophils rapidly, while dendritic cells acquire microbial antigens and coordinate later T-cell responses.

Neutrophils then provide high-capacity killing by phagocytosis, oxidative burst, granule proteases and antimicrobial peptides; NET formation can contribute when organisms are difficult to phagocytose, although excessive NETosis is injurious. Macrophages complement this by phagocytosing organisms, producing cytokines, removing surfactant debris and coordinating monocyte recruitment. Dendritic cells integrate danger signals with antigen presentation. Neutrophil products can enhance DC maturation and antigen availability, whereas macrophages can either amplify recruitment early or suppress it once microbial control is achieved.

Collateral epithelial injury is limited by timing and compartmentalization. Surfactant proteins, epithelial antioxidants and anti-proteases restrain extracellular oxidants and proteases. Neutrophil apoptosis is followed by macrophage efferocytosis, which decreases IL-1/TNF signaling and promotes IL-10, TGF-β, lipoxins, resolvins and other pro-resolving programs. Clearance of NET DNA and histones, restoration of epithelial tight junctions and mucociliary function, and termination of chemokine gradients are equally important.

Clinically, the protective versus pathogenic distinction depends on whether this response resolves. A brisk but self-limited neutrophil influx is essential for bacterial defense. Persistent recruitment, necrotic neutrophil death, defective efferocytosis or continued microbial stimulation creates a feed-forward loop of protease release, oxidant stress, mucus dysfunction and barrier injury. That pattern is relevant to severe pneumonia, acute lung injury and chronic neutrophilic airway disease, whereas intact macrophage-mediated resolution preserves antimicrobial efficacy without prolonged epithelial damage.

Q04

Question

How do eosinophils interact with macrophages and dendritic cells in the airway mucosa under homeostatic conditions and during type 2 inflammation?

Response

Under homeostatic conditions, airway eosinophils are relatively limited in number and are constrained by local survival signals. Macrophages and dendritic cells maintain a tolerogenic or low-inflammatory environment toward innocuous inhaled antigens. Alveolar macrophages clear apoptotic cells and particulate material, while dendritic cells sample antigen without necessarily driving strong T-cell priming. Eosinophils can contribute low-level lipid mediators, cytokines and growth factors, but their homeostatic role in human airways is less firmly defined than their role in type 2 disease.

During type 2 inflammation, the network changes sharply. Injured or allergen-exposed epithelium releases TSLP, IL-33 and IL-25. These alarmins activate dendritic cells and type 2 innate lymphoid cells, promoting Th2/ILC2 production of IL-4, IL-5 and IL-13. IL-5 prolongs eosinophil

survival, while eotaxins recruit them to the airway wall. Activated eosinophils release major basic protein, eosinophil peroxidase, cysteinyl leukotrienes and cytokines; these products injure epithelium, alter smooth-muscle and vascular responses, and can further activate antigen-presenting cells.

Dendritic cells are central to persistence because they translate epithelial danger signals into adaptive type 2 memory, including costimulatory programs and migration to draining lymph nodes. Macrophages exposed to IL-4/IL-13 adopt a repair/remodeling phenotype characterized by altered phagocytosis, chemokine production and extracellular-matrix signaling. This state can be protective after acute injury but, when sustained, can contribute to mucus hypersecretion, fibrosis and airway remodeling. Eosinophil-derived IL-4/IL-13 and lipid mediators can reinforce this macrophage state.

Resolution requires withdrawal of epithelial alarmins, eosinophil apoptosis, efficient macrophage efferocytosis and reduction of IL-5/eotaxin signaling. IL-10, TGF-β and specialized pro-resolving lipid mediators can restrain further activation. Thus, the same cell–cell network may support repair after a transient insult or maintain chronic asthma when epithelial activation, type 2 cytokines and granulocyte survival remain persistently elevated.

Q05

Question

In the urinary tract mucosa, how do neutrophils coordinate with monocytes or macrophages and dendritic cells during early antibacterial defense, and what mechanisms prevent excessive tissue injury?

Response

Early antibacterial defense in the urinary tract is initiated largely by urothelial and resident myeloid sensing of bacterial products. In uncomplicated bacterial cystitis, urothelial TLR signaling and resident macrophage responses induce IL-6, TNF and neutrophil-attracting chemokines, including CXCL8 in humans. Complement fragments and lipid mediators further sharpen the gradient. Neutrophils rapidly enter the mucosa and lumen, where they phagocytose uropathogens, generate an oxidative burst, release antimicrobial peptides and proteases, and may form extracellular traps.

Resident macrophages and recruited monocytes provide complementary functions. They help sense infection, produce chemokines that recruit neutrophils, phagocytose bacteria and damaged cells, and later clear apoptotic neutrophils. Monocyte-derived macrophages can therefore shift from an inflammatory, bactericidal program toward an efferocytic and reparative state as bacterial burden falls. Dendritic cells sample microbial antigens and provide the link to adaptive responses, particularly when infection persists or recurs. Neutrophil killing also changes antigen availability and the inflammatory signals encountered by dendritic cells.

Excessive injury is prevented by limiting both the magnitude and duration of neutrophil activation. Urothelial shedding and urine flow physically remove infected cells and organisms, reducing the need for prolonged inflammatory killing. Apoptotic neutrophils are engulfed by macrophages, which promotes IL-10, TGF-β and pro-resolving lipid mediator production. Antioxidant defenses and protease inhibitors limit bystander damage to the urothelium, while chemokine withdrawal terminates additional recruitment.

Failure of these brakes produces a clinically important trade-off. Insufficient neutrophil recruitment permits ascending infection and tissue invasion; excessive or prolonged activation produces epithelial denudation, edema, bleeding and pain and may contribute to chronic remodeling. NETs and extracellular proteases can be antimicrobial but become harmful when they persist after bacterial control. Accordingly, an effective urinary mucosal response is sequential: rapid neutrophil-dominant containment, macrophage-mediated clearance of inflammatory debris, and prompt epithelial repair.

Q06

Question

In the bladder or lower urogenital mucosa, how can mast-cell mediators influence macrophage and dendritic-cell functions, and how might this network contribute to both host defense and inflammatory symptoms?

Response

Mast cells are strategically positioned near bladder and lower urogenital vessels, nerves and epithelial surfaces, so their mediators can rapidly change the behavior of macrophages and dendritic cells. Following Fc receptor engagement, microbial sensing or neuroimmune stimulation, mast cells can release preformed histamine and tryptase together with newly synthesized prostaglandins, leukotrienes, TNF, IL-6 and chemokines. These signals increase local vascular permeability and leukocyte entry and can enhance macrophage phagocytic or inflammatory activity. TNF and other mediators can also promote dendritic-cell maturation, migration and costimulatory capacity.

This network can be protective during infection. Increased vascular permeability improves delivery of plasma antimicrobial factors and recruited leukocytes; mast-cell TNF and chemokines can accelerate neutrophil and monocyte recruitment; macrophages then phagocytose microbes and coordinate cytokine production, while dendritic cells support antigen-specific immunity. The benefit is greatest when mast-cell activation is brief and linked to a true barrier threat.

The same mediators explain why the network can generate symptoms. Histamine and lipid mediators sensitize sensory nerves and alter vascular tone. Tryptase can signal through protease-activated receptors, and mast-cell–derived growth factors can interact with neurotrophin pathways. Macrophage-derived IL-1, TNF, prostaglandins and reactive oxygen species may further lower sensory thresholds. Repeated neuropeptide release can in turn activate mast cells, creating a neuroimmune feedback loop associated with urgency, frequency and pain. Increased permeability and inflammatory proteases may weaken epithelial tight-junction function and expose subepithelial nerves to urinary solutes.

Whether this becomes chronic depends on regulatory counterweights. IL-10-producing or efferocytic macrophages, inhibitory mast-cell receptors, mediator degradation and restoration of epithelial barrier integrity favor resolution. Persistent mast-cell activation, macrophage inflammatory polarization and ongoing neural input can instead maintain symptoms after the initial trigger has subsided. Human data in chronic bladder pain syndromes are heterogeneous, so mast-cell–macrophage crosstalk is biologically plausible and supported by experimental evidence, but it should not be treated as a single universal mechanism for all patients.

Q07

Question

During acute intestinal mucosal inflammation and subsequent resolution, how do neutrophils and macrophages influence each other through recruitment signals, cytokines, efferocytosis, lipid mediators, and tissue-repair programs?

Response

Acute intestinal inflammation begins with a recruitment phase in which epithelial cells, resident macrophages and stromal cells generate CXCL chemokines, IL-1, TNF, complement-derived signals and lipid chemoattractants. Neutrophils follow these gradients into injured mucosa, where bacterial phagocytosis, reactive oxygen species, granule proteins and sometimes NETs provide immediate containment. Neutrophils also release LTB4 and cytokine-like mediators that can amplify further leukocyte recruitment, so the early response is intrinsically self-reinforcing.

Macrophages initially support this antimicrobial phase but are also responsible for switching the tissue into resolution. As neutrophils age they expose “eat-me” signals such as phosphatidylserine. Efferocytosis through receptors including MerTK and related pathways reprograms macrophages away from high-output TNF/IL-1 production toward IL-10, TGF-β, growth factors and tissue-repair programs. This is not merely passive cleanup: engulfment of apoptotic neutrophils changes macrophage lipid metabolism and transcriptional state.

A mediator-class switch is important. Early prostaglandins and leukotrienes support vascular and cellular recruitment; later lipoxins, resolvins, protectins and maresin-family mediators limit additional neutrophil influx, promote nonphlogistic phagocytosis and enhance epithelial repair. Neutrophils themselves can contribute pro-resolving signals, including annexin A1, while macrophages support fibroblast, endothelial and epithelial restitution through growth-factor and matrix-remodeling pathways.

Resolution fails when neutrophils undergo secondary necrosis or excessive NET formation before they are cleared, because extracellular histones, DNA, elastase and myeloperoxidase become persistent damage-associated signals. Likewise, defective macrophage efferocytosis allows inflammatory cytokines and chemokines to remain high. Clinically, therefore, neutrophil–macrophage crosstalk is biphasic: early reciprocal activation is protective against translocated microbes, whereas timely apoptotic-cell clearance and pro-resolving lipid signaling are required to stop tissue injury and restore the mucosal barrier.

Q08

Question

How can eosinophil-derived mediators alter macrophage polarization and dendritic-cell activation in the intestinal mucosa, and how could these interactions shift the balance among tolerance, tissue repair, and inflammation?

Response

Eosinophil-derived signals can move intestinal macrophages and dendritic cells toward either regulatory/repair states or inflammatory activation, depending on the tissue context. IL-4 and IL-13 are the clearest examples: when present at moderate levels they can promote macrophage programs associated with efferocytosis, matrix remodeling and tissue repair. Eosinophils also produce TGF-β, lipid mediators and growth factors that may influence stromal and epithelial repair. However, the traditional “M1 versus M2” terminology is too coarse; intestinal macrophages occupy multiple states shaped by microbiota, epithelial signals and monocyte replacement.

Dendritic cells are affected by both cytokines and eosinophil granule products. Eosinophil-derived neurotoxin and other cationic proteins can act as danger-associated signals in some experimental settings and may increase dendritic-cell activation or antigen presentation. Cysteinyl leukotrienes and other lipids can modify migration and cytokine output. Conversely, in a low-inflammatory mucosal environment, eosinophils may participate in networks that support IgA responses and limit excessive immune activation, although the relevant pathways are less consistently demonstrated in humans.

The functional balance depends on activation intensity. Limited IL-4/IL-13 signaling coupled to efficient clearance of damaged cells can favor repair and restoration of barrier function. Sustained eosinophil survival through IL-5/GM-CSF, high eotaxin exposure and degranulation release major basic protein, eosinophil peroxidase and reactive oxygen species, which injure epithelium and generate additional danger signals. Activated macrophages and dendritic cells then produce cytokines that maintain eosinophil recruitment and adaptive immune activation.

Thus, eosinophils are not intrinsically tolerogenic or inflammatory. Their impact is determined by which mediators dominate, whether granule contents remain intracellular or are released into tissue, and whether macrophage/DC responses terminate after repair. Evidence for some homeostatic eosinophil functions is strong in animal models but less definitive in humans, so mechanistic interpretation should remain tissue- and disease-specific.

Q09

Question

During neutrophilic airway inflammation, how do neutrophils and macrophages influence each other through cytokines, lipid mediators, neutrophil extracellular traps, efferocytosis, and metabolic signals, and how can this crosstalk determine persistence versus resolution?

Response

In neutrophilic airway inflammation, neutrophils and macrophages can form either a self-limited antimicrobial partnership or a self-sustaining inflammatory circuit. Activated macrophages and airway epithelium produce IL-1β, TNF, GM-CSF and CXCL8-family chemokines that recruit and prolong neutrophil survival. Neutrophils reciprocate by releasing proteases, reactive oxygen species, leukotriene B4 and alarmins that further activate macrophages and neighboring epithelium. When microbial products remain present, TLR signaling and inflammasome activation can maintain this loop.

NETs are a major fork in the pathway. Extracellular DNA, histones, elastase and myeloperoxidase can trap microbes but also damage epithelium, increase mucus viscosity and provide danger signals to macrophages. Macrophages exposed to NET components may increase IL-1 family cytokines and inflammasome activity. By contrast, when neutrophils die by apoptosis rather than lytic pathways, macrophage efferocytosis suppresses inflammatory cytokine production and promotes IL-10, TGF-β and pro-resolving programs.

Metabolism also matters. Inflamed, hypoxic airways favor glycolysis in both neutrophils and inflammatory macrophages. Lactate, succinate, altered redox state and HIF signaling can reinforce inflammatory gene expression, although the effect of any single metabolite is highly context dependent. A successful resolution phase includes a shift toward oxidative and reparative macrophage programs and production of lipoxins, resolvins and related specialized pro-resolving mediators.

Persistence therefore reflects failure of several brakes at once: continued chemokine production, prolonged neutrophil survival, NET accumulation, defective efferocytosis and metabolic conditions that favor inflammatory signaling. Resolution occurs when microbial burden falls, chemokine gradients collapse, apoptotic neutrophils are cleared and mediator production switches from recruitment-promoting lipids toward pro-resolving lipids. In severe airway disease, impaired macrophage phagocytosis or recurrent infection can prevent this transition and help explain chronic sputum neutrophilia and ongoing epithelial injury.

Q10

Question

During allergic airway inflammation, how do mast cells and eosinophils, with basophils where relevant, interact with dendritic cells and macrophages to amplify or restrain type 2 immunity?

Response

Type 2 airway inflammation is sustained by reciprocal signaling among mast cells, eosinophils, basophils, dendritic cells and macrophages rather than by any one lineage. Allergen-induced epithelial alarmins such as TSLP, IL-33 and IL-25 condition dendritic cells and activate ILC2s. Dendritic cells then promote Th2 differentiation and memory, while IL-4, IL-5 and IL-13 provide the cytokine framework that recruits eosinophils, enhances IgE production and alters macrophage function.

Mast cells amplify the response when allergen cross-links FcεRI-bound IgE. Histamine, tryptase, prostaglandin D2 and cysteinyl leukotrienes rapidly increase bronchoconstriction, vascular leak and leukocyte recruitment; later TNF, IL-4, IL-5 and IL-13 reinforce dendritic-cell activation and eosinophil survival. Basophils can provide an additional early source of IL-4 and leukotrienes, especially in sensitization or tissue contexts where they are recruited. Eosinophils then release granule proteins, lipid mediators and cytokines that injure epithelium and perpetuate alarmin release.

Macrophages are functionally plastic within this circuit. IL-4/IL-13 signaling through IL-4Rα promotes repair-oriented and remodeling programs, altered chemokine production and increased scavenging; these can help restore tissue after an acute insult but can also support mucus production, fibrosis and persistent type 2 inflammation. Macrophages can restrain disease by clearing apoptotic eosinophils and producing IL-10 and pro-resolving mediators. Dendritic-cell activity is likewise limited when epithelial danger signals decline and regulatory signals dominate.

The balance between amplification and restraint therefore depends on duration and localization. Short-lived mast-cell activation and eosinophil recruitment can contribute to barrier defense and repair. Persistent IgE signaling, repeated epithelial injury, sustained IL-5/IL-13 exposure and inadequate efferocytosis create a positive-feedback network that maintains chronic asthma. Regulatory T-cell activity, IL-10, inhibitory Fc receptors and specialized pro-resolving lipids provide counter-regulatory pressure, but their effectiveness varies among patients and disease endotypes.

Q11

Question

During acute bacterial urinary tract infection, how do recruited neutrophils and monocyte-derived macrophages cooperate or compete in pathogen clearance, cytokine production, efferocytosis, and mucosal repair?

Response

During acute bacterial urinary tract infection, neutrophils usually provide the fastest high-intensity antimicrobial response. Urothelial and resident macrophage sensing of uropathogens generates CXCL8 and related chemokines, IL-1 and TNF, bringing neutrophils into the mucosa and urinary lumen. They phagocytose bacteria, generate reactive oxygen species and release granule antimicrobials; NET formation may contribute in some settings. This is highly effective but potentially damaging if recruitment and degranulation remain excessive.

Monocyte-derived macrophages arrive somewhat later and can both cooperate with and compete against neutrophils. Cooperation occurs when macrophages sustain early chemokine production, phagocytose organisms not cleared by neutrophils, process cellular debris and remove apoptotic neutrophils. Competition is mainly functional rather than literal: both populations consume inflammatory signals and microbes, but they may favor different cytokine programs. Persistent TNF/IL-1 production from either lineage can amplify injury, whereas macrophage IL-10 and efferocytosis favor shutdown.

Efferocytosis is the major transition point. Once neutrophils become apoptotic, macrophage uptake of these cells reduces additional inflammatory cytokine release and promotes repair-associated transcriptional programs. This supports restoration of urothelial integrity after exfoliation or focal epithelial loss. Growth-factor and matrix-remodeling signals from macrophages, together with urothelial progenitor responses, help rebuild the barrier.

Pathogen biology can complicate this sequence. Uropathogenic bacteria may persist in intracellular reservoirs or protected niches, so a macrophage-driven switch to resolution that occurs too early could permit persistence. Conversely, failure to switch leaves the bladder exposed to prolonged oxidants, proteases and inflammatory edema. The most effective response is therefore sequential rather than maximally inflammatory throughout: rapid neutrophil bacterial control, macrophage-supported clearance, efficient efferocytosis, then mucosal repair. The relative contribution of each cell type varies with bacterial strain, host genetics, prior infection and whether disease remains confined to the bladder or ascends toward the kidney.

Q12

Question

In chronic bladder mucosal inflammation, how might mast-cell–macrophage crosstalk alter sensory signaling, vascular permeability, epithelial barrier function, and the persistence or resolution of inflammation?

Response

In chronic bladder mucosal inflammation, mast cells and macrophages can couple immune activation to sensory dysfunction. Mast-cell histamine, tryptase, prostaglandins and leukotrienes increase vascular permeability and can directly sensitize afferent nerves. Tryptase can activate protease-activated receptors, while mast-cell mediators may enhance neurotrophin pathways such as nerve growth factor signaling. Activated sensory neurons release neuropeptides, including substance P and CGRP, which can feed back on mast cells and vascular cells, creating a self-reinforcing neuroimmune loop.

Macrophages can strengthen or dampen this loop. Inflammatory macrophage states produce IL-1β, TNF, prostaglandins, reactive oxygen species and chemokines, all of which can lower sensory thresholds and recruit additional leukocytes. They may also increase endothelial activation and

prolong vascular leak. In contrast, efferocytic or regulatory macrophage programs produce IL-10, TGF-β and pro-resolving mediators that suppress cytokine output and support tissue repair.

The epithelial barrier is central. Histamine, proteases and inflammatory cytokines can alter tight-junction proteins and urothelial permeability, allowing urinary solutes to reach subepithelial nerves and immune cells. Barrier disruption then creates more danger signaling, which further activates mast cells and macrophages. With prolonged disease, fibroblast activation, extracellular-matrix remodeling and altered innervation may make symptoms persist even after the initiating trigger has diminished.

Resolution requires simultaneous control of several compartments: reduction of mast-cell activation, macrophage transition toward efferocytosis and repair, restoration of urothelial integrity, and interruption of neuropeptide-driven feedback. This framework is mechanistically plausible and supported by experimental and histologic studies, but human chronic bladder pain syndromes are heterogeneous. Mast-cell numbers, activation markers and macrophage phenotypes do not show a uniform pattern in every patient, so the pathway should be considered one potentially important endotype rather than a universal explanation.

Q13

Question

In ulcerative colitis, how do neutrophil recruitment, neutrophil extracellular trap formation, neutrophil metabolism, and neutrophil–macrophage crosstalk contribute to mucosal injury versus inflammatory resolution?

Response

In ulcerative colitis, neutrophils are both indispensable defenders and major contributors to tissue injury. Chemokines such as CXCL8, together with IL-1, TNF, complement products and lipid chemoattractants, drive dense recruitment into colonic crypts; crypt abscesses are the histologic expression of this response. Neutrophils phagocytose translocated bacteria and limit invasion, but extracellular release of elastase, myeloperoxidase, reactive oxygen species and other granule contents damages epithelial cells and tight junctions when the response is prolonged.

NET formation can intensify this injury. NET DNA, histones and granule enzymes immobilize microbes but also act as cytotoxic and prothrombotic danger signals. Persistent NET material can activate macrophages and other innate cells, promote epithelial death and amplify local cytokine production. This does not mean all NET formation is pathogenic; its effect depends on burden, clearance and whether microbial containment still requires it.

Neutrophil metabolism also shapes function. Neutrophils rely heavily on glycolysis, allowing them to operate in the hypoxic inflamed mucosa. Increased glucose flux and pentose-phosphate activity support NADPH oxidase and antimicrobial activity, but an environment rich in hypoxia, lactate and inflammatory metabolites can favor prolonged activation. Macrophages exposed to neutrophil debris and microbial products may remain in IL-1/TNF-producing states rather than transitioning to repair.

Resolution depends on apoptotic neutrophil clearance. Macrophage efferocytosis suppresses inflammatory signaling and promotes IL-10, TGF-β and specialized pro-resolving lipid pathways. Repair programs then support epithelial restitution and barrier re-establishment. In active ulcerative colitis, ongoing microbial translocation, impaired epithelial healing and continued cytokine production can prevent this switch.

The clinical implication is a narrow therapeutic window: completely blocking neutrophils risks bacterial invasion, whereas failing to control recruitment, NET burden or protease release promotes mucosal injury. The preferred mechanistic strategy is therefore to reduce pathological persistence and extracellular effector release while preserving phagocytosis and macrophage-mediated resolution.

Q14

Question

In Crohn’s disease, how can dysregulated interactions among neutrophils, monocyte-derived macrophages, and dendritic cells alter microbial handling, antigen presentation, inflammatory persistence, and epithelial repair?

Response

Crohn’s disease can be understood in part as a failure to coordinate sequential myeloid functions after microbes cross a damaged intestinal barrier. Neutrophils are needed for rapid containment, but recruitment and killing may be temporally or spatially dysregulated. Some Crohn’s disease models emphasize inadequate early innate clearance followed by prolonged secondary inflammation; established disease often shows abundant neutrophils at active lesions. These observations are not contradictory because different disease stages and genetic backgrounds can produce different recruitment phenotypes.

Monocyte-derived macrophages are particularly important in chronicity. Unlike homeostatic intestinal macrophages, which are relatively tolerant of commensal stimulation, newly recruited monocytes in active disease can produce high levels of TNF, IL-1β, IL-6 and IL-23. Defects in microbial sensing and autophagy-associated pathways, including NOD2- and ATG16L1-related biology, can alter bacterial handling and intracellular stress responses. Persistence of microbial material then sustains macrophage and stromal cytokine production.

Dendritic cells translate this innate inflammation into adaptive persistence by presenting microbial antigens and producing cytokines that support Th1/Th17-type responses. IL-23 is especially important in the myeloid-to-lymphoid interface. Activated T cells then return cytokine signals that maintain macrophage activation and recruit additional granulocytes, generating a closed inflammatory circuit.

Epithelial repair is impaired when inflammatory myeloid products remain high. TNF, IL-1, oxidants, proteases and matrix-remodeling enzymes can delay barrier restitution; prolonged repair signaling may also contribute to fibrostenotic remodeling. At the same time, macrophages are required for debris clearance, angiogenesis and tissue reconstruction, so global myeloid suppression is undesirable.

Clinically, Crohn’s disease is heterogeneous. Genetic defects, microbiota composition, smoking, disease location and prior therapy all influence which myeloid defect dominates. The best-supported model is therefore not a single abnormal cell type but a breakdown in timing: defective or misdirected microbial clearance, persistent inflammatory monocyte/macrophage signaling, dendritic-cell-supported adaptive activation and incomplete epithelial repair.

Q15

Question

In severe neutrophilic asthma, how do neutrophils, macrophages, and dendritic cells form self-sustaining inflammatory circuits, and what roles may neutrophil extracellular traps, inflammasome signaling, microbial signals, and corticosteroid resistance play?

Response

Severe neutrophilic asthma can be maintained by a feed-forward circuit linking injured epithelium, macrophages, dendritic cells and neutrophils. Airway epithelial cells and macrophages exposed to microbes, pollutants or cell damage produce IL-1β, IL-6, TNF, GM-CSF and CXCL8-family chemokines. Dendritic cells can support Th17-skewed adaptive responses, and IL-17 further drives epithelial and stromal production of neutrophil-recruiting chemokines. Neutrophils then release proteases, oxidants and alarmins that create additional epithelial injury and perpetuate the signal.

NETs can strengthen this circuit by delivering extracellular DNA, histones, elastase and myeloperoxidase to the airway surface. These molecules can increase mucus viscosity, damage epithelium and activate pattern-recognition or inflammasome pathways in macrophages and epithelial cells. NLRP3–IL-1β signaling is mechanistically attractive in a subset of patients, although not all neutrophilic asthma is inflammasome driven.

Microbial signals are another source of persistence. Chronic colonization, altered airway microbiota or recurrent infection can maintain TLR signaling and neutrophil recruitment. The relationship is bidirectional because structural airway disease and impaired mucociliary clearance also make colonization more likely.

Corticosteroid resistance is multifactorial. Oxidative stress and p38 MAPK signaling can impair glucocorticoid receptor function; Th17-associated inflammation is often less steroid responsive than classic type 2 inflammation; and corticosteroids can delay neutrophil apoptosis, potentially sustaining cell numbers even while suppressing other inflammatory pathways. None of these mechanisms is universal, but together they explain why increasing steroid dose may yield limited benefit in some neutrophilic endotypes.

Persistence is most likely when NET burden is high, macrophage efferocytosis is poor and microbial or inflammasome signaling continues. Resolution requires collapse of CXCL8/IL-1-driven recruitment, apoptotic neutrophil clearance and restoration of epithelial integrity. Clinically, severe neutrophilic asthma should therefore be treated as a heterogeneous syndrome in which infection, bronchiectatic change, steroid exposure and non-type-2 inflammation may contribute in different proportions.

Q16

Question

In eosinophilic asthma, how do eosinophils, mast cells, and where relevant basophils interact with macrophages, dendritic cells, and airway epithelium to initiate, amplify, and resolve type 2 inflammation?

Response

Eosinophilic asthma begins at the epithelial–immune interface. Allergens, viruses and epithelial stress trigger TSLP, IL-33 and IL-25, which condition dendritic cells and activate ILC2s. Dendritic cells promote Th2 differentiation and memory, while Th2 cells and ILC2s produce IL-4, IL-5 and IL-13. IL-5 supports eosinophil maturation and survival; epithelial eotaxins recruit eosinophils to the airway; IL-4 and IL-13 promote IgE production, mucus metaplasia and broader type 2 tissue programs.

Mast cells amplify the response through FcεRI-bound IgE. Allergen exposure triggers histamine, tryptase, prostaglandin D2 and cysteinyl leukotrienes, producing bronchoconstriction, vascular leak and rapid recruitment. Basophils, when present in sufficient numbers, provide additional IL-4 and lipid mediators and may contribute to sensitization or exacerbation. Eosinophils then release major basic protein, eosinophil peroxidase, leukotrienes and cytokines that damage epithelium and maintain alarmin production.

Macrophages are not merely bystanders. IL-4/IL-13 signaling alters macrophage phagocytosis, chemokine output and matrix-remodeling behavior. These “type 2-conditioned” macrophage states can support wound repair but may also promote mucus production and fibrosis when stimulation is chronic. Macrophage clearance of apoptotic eosinophils is an important resolution mechanism. Dendritic-cell activity also declines when epithelial alarmins and antigen exposure fall.

Resolution therefore requires interruption at several levels: reduced epithelial danger signaling, withdrawal of IL-5-dependent eosinophil survival, cessation of IgE-driven mast-cell activation and efficient efferocytosis. IL-10, TGF-β and specialized pro-resolving lipid mediators can further suppress residual inflammation. Clinically effective type 2 biologics validate many elements of this network, but patients differ in which node dominates; some retain symptoms because structural remodeling, infection or non-type-2 inflammation persists even after eosinophilic inflammation is controlled.

Q17

Question

In recurrent urinary tract infection, how can prior infection reshape neutrophil and monocyte or macrophage responses at the bladder mucosa through trained immunity, tolerance, or tissue remodeling, and how strong is the evidence for each mechanism?

Response

Prior urinary tract infection can leave several forms of “memory” at the bladder mucosa, but the evidence is not equally strong for all proposed mechanisms. The best-established phenomenon is tissue remodeling. Infection can cause urothelial exfoliation, regenerative proliferation, altered stromal signaling and changes in local innervation. Uropathogenic bacteria may also persist in intracellular reservoirs. These structural and microbial changes can alter the threshold for subsequent neutrophil recruitment and macrophage activation even after clinical symptoms resolve.

Trained immunity is mechanistically plausible but less firmly established in human recurrent UTI. Repeated pattern-recognition signals can produce durable metabolic and epigenetic changes in bone-marrow progenitors and monocytes, including increased glycolytic readiness and activating chromatin marks at inflammatory genes. In principle, newly recruited monocytes or macrophages could then respond more rapidly to a second infection. Neutrophils are short lived, so durable “training” is more likely to arise at the progenitor level or through persistent tissue signals than from long-lived individual neutrophils. Animal studies support innate memory-like effects, but direct demonstration in human bladder myeloid cells remains limited.

The opposite program, tolerance, is also plausible. Repeated TLR stimulation can reduce subsequent TNF/IL-1 output while preserving or enhancing selected antimicrobial functions. This could limit bladder injury but may also blunt early warning signals and permit bacterial persistence. Whether recurrent UTI patients show maladaptive tolerance in vivo is still uncertain.

A clinically realistic model is that these mechanisms coexist. One patient may have an inflamed, hyperresponsive mucosa after prior infection; another may have weakened epithelial defense, persistent bacterial reservoirs or relatively tolerant myeloid signaling. Evidence for tissue remodeling and intracellular persistence is comparatively strong, evidence for altered innate responsiveness is moderate and mostly experimental, and evidence for a specific, durable neutrophil- or macrophage-trained state in human recurrent UTI remains preliminary. Longitudinal human studies with cell-specific epigenetic and functional measurements are needed before trained immunity or tolerance can be considered established drivers of recurrence.

Q18

Question

In bladder cancer, how do tumor-associated neutrophils and macrophages interact within the mucosal tumor microenvironment to influence antigen presentation, T-cell activity, angiogenesis, invasion, and response to immunotherapy?

Response

In bladder cancer, tumor-associated macrophages (TAMs) and tumor-associated neutrophils (TANs) can form a mutually reinforcing myeloid compartment that suppresses adaptive immunity and supports tumor growth. Tumor cells and stromal cells release CCL2, CSF1, CXCL8, G-CSF and related factors that recruit monocytes and neutrophils. Within the tumor, hypoxia, TGF-β, IL-10, prostaglandins and metabolic stress can shift these cells toward immunosuppressive or tissue-remodeling states.

TAMs may reduce effective antigen presentation through low costimulatory activity while expressing inhibitory ligands such as PD-L1 and producing IL-10, TGF-β, arginase-associated metabolites and reactive oxygen species. TANs can further suppress T-cell function through oxidants, proteases, arginine metabolism and, in some contexts, NET formation. Reciprocal cytokines and chemokines allow each population to reinforce recruitment and survival of the other. This myeloid milieu can exclude or disable cytotoxic T cells even when tumor antigens are present.

The same cells also promote angiogenesis and invasion. VEGF, matrix metalloproteinases, elastase and other proteases remodel extracellular matrix and blood vessels. NETs may facilitate tumor-cell adhesion or dissemination in experimental systems. Importantly, macrophage and neutrophil phenotypes are not fixed “M1/M2” or “N1/N2” categories; bladder tumors contain a continuum of states that vary with grade, hypoxia, prior therapy and infection.

Myeloid composition also influences immunotherapy. A strongly suppressive TAM/TAN program is plausibly associated with poor response to checkpoint blockade, whereas local immune stimulation can activate antitumor myeloid functions. Intravesical BCG is a clinically important example of deliberate mucosal innate activation: early neutrophil and macrophage recruitment helps create a proinflammatory environment that can support antitumor immunity, although excessive inflammation causes toxicity and not all tumors respond.

The therapeutic goal is therefore selective reprogramming rather than global depletion. Suppressive macrophage signaling, CXCR2-dependent neutrophil recruitment, TGF-β pathways, NET-associated injury and metabolic suppression are candidate nodes, but any intervention must preserve urinary antimicrobial defense. Human validation remains essential because the same myeloid cells can be protective during infection and antitumor when appropriately activated.

Q19

Question

Which mechanistically plausible strategies could suppress pathogenic neutrophil recruitment or neutrophil-extracellular-trap-associated injury in ulcerative colitis while preserving antimicrobial defense and macrophage-mediated repair? Explain predicted benefits, trade-offs, and evidence gaps.

Response

A rational strategy in ulcerative colitis is to target the pathological persistence and extracellular effector functions of neutrophils rather than eliminate neutrophils altogether. One approach is partial or local inhibition of CXCL8–CXCR1/2-dependent recruitment. This could reduce crypt accumulation, protease release and mucosal oxidant injury, but systemic blockade risks impaired bacterial containment. For that reason, mucosa-restricted delivery, biomarker selection and incomplete rather than absolute pathway inhibition are mechanistically preferable.

A second approach is to reduce NET-associated toxicity. PAD4 inhibition can limit chromatin decondensation in some NET programs, whereas extracellular DNase can accelerate NET clearance. Neutralizing extracellular histones, elastase or myeloperoxidase may preserve phagocytosis while reducing tissue-toxic components. The trade-off is that NETs can contribute to extracellular microbial control, and DNase can release active proteases from the DNA scaffold unless protease activity is simultaneously controlled. Not all NET formation is PAD4 dependent, which is another limitation.

A third strategy is to enhance resolution. Annexin A1 pathways, specialized pro-resolving mediators and macrophage efferocytosis can shorten neutrophil residence without broadly suppressing recruitment. Improving MerTK-dependent apoptotic-cell clearance, for example, would be expected to increase IL-10 and repair programs while reducing secondary necrosis. Barrier-directed therapy that reduces microbial translocation can also lower the upstream stimulus for neutrophil recruitment rather than targeting the cell directly.

The predicted benefit of these approaches is preservation of intracellular killing and macrophage-mediated repair while reducing extracellular protease, oxidant and NET injury. The main risks are bacterial infection, delayed clearance of translocated microbes and interference with wound debridement. Evidence gaps are substantial: many NET- or resolution-directed strategies remain preclinical, human mucosal pharmacodynamic markers are poorly standardized, and ulcerative colitis is biologically heterogeneous. The most promising translational design is therefore local, reversible and endotype-guided, with simultaneous monitoring of antimicrobial competence and epithelial healing.

Q20

Question

How could metabolic or epigenetic reprogramming of intestinal macrophages and granulocytes be used to restore mucosal homeostasis in inflammatory bowel disease, and what mechanisms could cause loss of efficacy or unintended immunosuppression?

Response

Metabolic and epigenetic reprogramming could theoretically restore intestinal homeostasis by changing the state of inflammatory myeloid cells without removing them. In macrophages, active inflammatory programs are often associated with glycolysis, HIF signaling, altered succinate/itaconate metabolism, mTOR activity and changes in NAD+/redox balance. Shifting selected cells toward AMPK-supported oxidative

metabolism, restoring mitochondrial function or modifying immunoregulatory metabolites may reduce IL-1β/TNF output while preserving phagocytosis and efferocytosis. These are network effects rather than a simple "M1-to-M2" conversion.

Epigenetic interventions offer a second layer. Persistent inflammatory monocytes can carry altered enhancer accessibility, histone acetylation/methylation and trained-immunity-like chromatin states. BET, HDAC, histone-methylation or DNA-methylation pathways are therefore potential targets. In principle, local or cell-selective modulation could reduce exaggerated cytokine transcription and allow macrophages to reacquire tolerogenic, efferocytic and repair functions.

Granulocytes require a different strategy. Neutrophils depend heavily on glycolysis and the pentose-phosphate pathway for migration, oxidative burst and NET formation. Strong metabolic inhibition could therefore reduce tissue damage but also directly compromise bacterial killing. More selective targets would aim to limit excessive NETosis, prolonged survival or extracellular protease activity while retaining phagocytosis and oxidative killing at sites of true infection.

Loss of efficacy can arise through metabolic plasticity, redundant cytokine pathways, continued microbial translocation and replenishment by newly recruited monocytes. Epigenetic states may also be reversible once treatment stops. Unintended immunosuppression is a major concern because the same pathways that drive inflammatory injury support antimicrobial defense, wound healing and vaccine responses. Systemic chromatin-modifying drugs also affect epithelial, lymphoid and hematopoietic progenitor compartments.

Translation therefore requires spatial and cellular selectivity. Mucosa-targeted delivery, short treatment windows and biomarkers of macrophage state, neutrophil function and barrier healing are more attractive than chronic systemic suppression. At present, the concept is mechanistically compelling, but most specific myeloid metabolic or epigenetic reprogramming strategies remain experimental rather than established IBD therapy.

Q21

Question

How might biologics that alter type 2 cytokine signaling reshape the eosinophil–mast-cell–macrophage–dendritic-cell network in airway mucosa, beyond simply reducing eosinophil counts?

Response

Biologics that alter type 2 cytokine signaling reshape the entire airway myeloid network because IL-4, IL-5, IL-13 and epithelial alarmins control cell state as well as cell number. Blocking IL-5 or IL-5R reduces eosinophil maturation, survival and tissue persistence, but it also decreases eosinophil-derived leukotrienes, granule proteins and cytokines that normally feed back on mast cells, macrophages, dendritic cells and epithelium. The downstream effect is therefore a quieter network, not simply a lower blood eosinophil count.

Blocking IL-4Rα interrupts both IL-4 and IL-13 signaling. This can reduce epithelial eotaxin production, mucus metaplasia and IgE-supporting signals while changing macrophage transcriptional programs that are driven by IL-4/IL-13. Type 2-conditioned macrophages may become less involved in mucus, fibrosis and remodeling. Dendritic-cell and T-cell communication is also altered indirectly because the epithelial cytokine environment and antigen exposure change.

Biologics targeting upstream alarmins, such as TSLP pathways, can act even earlier. Reduced epithelial-to-dendritic-cell signaling may decrease Th2 priming, ILC2 activation and subsequent eosinophil/mast-cell recruitment. Interfering with IL-33-related signaling would be expected to have similarly broad effects on ILC2s, mast cells and eosinophils, although the clinical maturity of individual pathways differs. Anti-IgE therapy primarily reduces FcεRI-driven mast-cell and basophil activation but secondarily lowers the inflammatory signals encountered by dendritic cells and macrophages.

These network changes can improve resolution because fewer activated eosinophils need to be cleared and macrophage efferocytosis is less burdened. However, biologic therapy does not fully erase structural remodeling, infection, fixed airflow limitation or non-type-2 inflammation. Redundant pathways can also compensate. Clinically, response is therefore best understood as network rebalancing: less epithelial alarmin amplification, less granulocyte survival and degranulation, altered macrophage remodeling programs and weaker dendritic-cell support for recurrent type 2 memory.

Q22

Question

What myeloid-directed strategies could interrupt neutrophil–macrophage inflammatory circuits in neutrophilic asthma while preserving antibacterial defense, and which proposed mechanisms are clinically established versus preclinical?

Response

The central therapeutic challenge in neutrophilic asthma is to interrupt the CXCL8/IL-1–neutrophil–macrophage circuit without creating a lung that cannot clear bacteria. At present, there is no broadly accepted, myeloid-specific standard therapy that reliably achieves this balance. Macrolides have the strongest clinical foothold as an adjunct in selected severe asthma patients. Their benefit may include reduced exacerbations and immunomodulatory effects on neutrophils, macrophages and epithelial cytokine production, but they are not a selective myeloid therapy and long-term use raises antimicrobial-resistance and microbiome concerns.

Direct CXCR2 antagonism is mechanistically attractive because it reduces neutrophil trafficking. Clinical trials have shown that sputum or blood neutrophil measures can fall, but symptom and exacerbation benefits have been inconsistent, and infection risk is a concern. Therefore, CXCR2 blockade is clinically tested but not established routine therapy. Broad anti-IL-1, anti-IL-17 or related cytokine approaches have similarly not produced a universally effective neutrophilic-asthma treatment.

Several strategies remain mainly preclinical or early translational. NLRP3 inhibition could reduce macrophage IL-1β production in inflammasome-high disease. PAD4 inhibition, DNase-based NET clearance, extracellular histone neutralization or neutrophil-protease inhibition could reduce NET-associated epithelial injury while preserving some phagocytic function. Enhancing macrophage efferocytosis or specialized pro-resolving lipid pathways may be particularly attractive because it promotes removal of spent neutrophils rather than preventing all recruitment.

Preserving antibacterial defense favors local, reversible and endotype-specific intervention. Patients with chronic bacterial colonization, bronchiectasis or frequent infections are poor candidates for strong systemic neutrophil suppression. Biomarkers such as sputum neutrophilia, NET burden, IL-1/inflammasome signatures and evidence of bacterial colonization may be needed to select patients.

The clinically established part of the field is therefore limited: macrolides are used in selected severe asthma, while most specifically myeloid-directed mechanisms are investigational. The translational goal is not "anti-neutrophil" therapy but restoration of normal kinetics—adequate recruitment during infection, followed by rapid apoptosis, efferocytosis and termination of inflammatory signaling.

Q23
Question
Could trained-immunity or mucosal-vaccine approaches beneficially reprogram monocytes or macrophages and neutrophils to reduce recurrent urinary tract infection? Explain the proposed mechanisms, potential tissue-specific risks, and current evidence limitations.

Response

Trained-immunity and mucosal-vaccine approaches could, in principle, reduce recurrent urinary tract infection by improving the speed and quality of local antimicrobial responses rather than maintaining chronic inflammation. A mucosal vaccine would ideally generate pathogen-specific antibody, tissue-resident lymphocyte responses and faster epithelial/myeloid activation at re-exposure. Enhanced opsonization could make neutrophil and macrophage phagocytosis more efficient, reducing the duration of high-intensity inflammation needed to control bacteria.

Trained immunity addresses the innate side. Pattern-recognition stimulation can epigenetically and metabolically reprogram bone-marrow progenitors and monocytes so that later challenge produces a faster or qualitatively different response. A useful program would increase phagocytosis, chemotaxis and antimicrobial effector readiness while avoiding excessive IL-1/TNF or NET release. Because mature neutrophils are short lived, durable neutrophil effects would probably arise from progenitor reprogramming or changes in the tissue environment rather than long-lived "memory neutrophils."

Tissue specificity is the major safety issue. A bladder mucosa that is chronically primed could develop excessive neutrophil recruitment, urothelial injury, pain, urgency or fibrosis. Repeated innate stimulation could also alter local microbiota or generate tolerance rather than training, reducing cytokine responsiveness at the wrong time. Systemic training could theoretically worsen inflammatory disease outside the urinary tract.

Current evidence is encouraging but incomplete. Experimental models demonstrate that innate immune states can be durably altered, and several vaccine or bacterial-lysate strategies have been explored clinically for recurrent UTI. However, product formulations, endpoints and study quality are heterogeneous, and no approach has established that benefit is specifically mediated by trained monocytes/macrophages or neutrophils. Direct human bladder data showing durable, beneficial myeloid epigenetic reprogramming are particularly sparse.

The most credible future strategy would combine antigen-specific mucosal protection with tightly controlled innate conditioning, using biomarkers to confirm enhanced bacterial clearance without chronic inflammation. Until such studies are available, trained immunity should be considered a plausible mechanism and therapeutic concept rather than a clinically proven explanation for prevention of recurrent UTI.

Q24
Question
How could selective reprogramming of tumor-associated macrophages and neutrophils improve antitumor immunity in bladder cancer while minimizing disruption of protective urinary mucosal immunity? Compare mechanistic approaches and key translational barriers.

Response

Selective myeloid reprogramming in bladder cancer should aim to convert tumor-associated macrophages and neutrophils from immunosuppressive, tissue-remodeling states into short-lived antitumor effectors while preserving their capacity to control urinary pathogens. The bladder is unusually suitable for this concept because intravesical delivery can expose the tumor and mucosa while limiting systemic myeloid suppression.

For macrophages, candidate approaches include blocking CSF1/CSF1R- or CCL2-dependent recruitment, inhibiting PI3Kγ-like suppressive signaling, activating innate pathways such as TLR or STING, and enhancing phagocytosis through the CD47–SIRPα checkpoint. The desired result is greater tumor-cell uptake, improved inflammatory antigen presentation and less IL-10/TGF-β/VEGF-driven suppression. However, macrophage depletion alone may remove cells needed for bacterial defense and wound repair, so functional reprogramming is preferable to indiscriminate elimination.

For neutrophils, CXCR2-axis inhibition could reduce recruitment of suppressive TAN states, while NET/PAD4, elastase or TGF-β-related interventions could decrease pro-invasive and immunosuppressive functions. The trade-off is immediate: neutrophils are critical for defense against ascending urinary infection. Local delivery or intermittent treatment around antitumor therapy is therefore more attractive than chronic systemic neutropenia.

These approaches may synergize with checkpoint blockade by improving T-cell entry and reducing myeloid suppression. Intravesical BCG already provides proof that local innate activation can generate clinically meaningful antitumor immunity, although BCG is not a selective macrophage/neutrophil reprogrammer and can cause substantial inflammatory toxicity.

The major translational barriers are myeloid plasticity, absence of stable human TAM/TAN markers, tumor heterogeneity, variable drug penetration, compensatory recruitment pathways and infection risk. Biomarkers must distinguish suppressive tumor myeloid cells from protective mucosal cells, which may be spatially adjacent. The most realistic strategy is therefore spatially restricted, reversible combination therapy: locally reprogram the tumor-associated compartment, monitor urinary infection risk and barrier injury, and pair myeloid modulation with therapies that activate tumor-specific T cells.

Japanese (JP)

Anonymous Model D | 日本語

Anonymous Model D
日本語
24 standardized questions

Q01
質問
健常な腸管粘膜において、好中球は常在マクロファージおよび樹状細胞と相互作用しながら、どのようにバリア防御に寄与しますか。また、好中球による組織障害を通常どのような機序が抑制していますか。

回答

健常腸管では、好中球は粘膜固有層に大量に常在する細胞ではなく、上皮バリアが破綻した際に迅速に動員される即応性エフェクターとして機能します。上皮細胞、間質細胞、常在マクロファージが CXCL1/2 や、ヒトでは CXCL8 を中心とするケモカイン、補

体由来シグナル、脂質メディエーターを産生すると、好中球は微小血管から病変部へ移行します。局所では貪食、NADPH オキシダーゼ依存性の酸化バースト、抗菌顆粒蛋白の放出、必要に応じた NET 形成によって侵入細菌や真菌を制御します。防御効果を保つためには、これらの強力な機能がバリア破綻部位に限局していることが重要です。

常在マクロファージはこの反応を“増幅する側”と“止める側”の両方から調節します。健常腸管マクロファージは微生物や死細胞を持続的に処理しつつ、共生菌刺激に対しては比較的低反応性で、IL-10 などを介して過剰な炎症を抑えています。樹状細胞は抗原をサンプリングし、感染が実質的な脅威となった場合に適応免疫を起動します。好中球由来のプロテアーゼや DAMPs はマクロファージや樹状細胞を刺激し得ますが、健常時にはこのクロストークは短時間で終息します。

組織障害の抑制には能動的な炎症収束が必要です。好中球は抗菌機能を終えるとアポトーシスに入り、MerTK や AXL などに関わる経路を介してマクロファージにエフェロサイトーシスされます。これにより TNF や IL-1 の産生が低下し、IL-10、TGF-β、修復関連プログラムが優位になります。さらにリポキシン、レゾルビンなどの特殊化炎症収束メディエーター、アデノシン、Annexin A1、上皮由来抗プロテアーゼや粘液が、ROS、エラスターゼ、MPO、NET 成分による二次的障害を抑えます。

したがって、好中球は「迅速な動員—局所殺菌—速やかなアポトーシス—マクロファージによる除去」という時間的秩序が保たれる限り防御的です。動員が持続し、NET が蓄積し、二次壊死やエフェロサイトーシス不全が起こると、同じエフェクター機構が炎症性腸疾患でみられる粘膜障害へ転化します。

Q02

質問

健常な腸管粘膜において、好酸球はどのように恒常性の維持に寄与し、マクロファージまたは樹状細胞と情報交換しますか。また、これらの役割は炎症時の好酸球機能とどのように異なりますか。

回答

腸管好酸球の特徴は、明らかな炎症がなくても粘膜固有層に比較的多く存在する点です。その維持には IL-5 や CCL11 などの eotaxin 系ケモカインが関与します。恒常状態では、好酸球は上皮･間質の恒常性、細胞外マトリックスの回転、免疫監視、局所液性免疫の調節に関与すると考えられています。動物モデルでは形質細胞の生存や IgA 関連応答への関与も示されていますが、ヒトでの必須性や腸管部位ごとの重要度はなお一定していません。

マクロファージや樹状細胞との情報交換は双方向性です。好酸球由来 IL-4/IL-13 は、マクロファージをエフェロサイトーシス、組織修復、マトリックス再構築に関連する状態へ傾け得ます。TGF-β、脂質メディエーター、増殖因子も組織修復に影響します。一方、マクロファージや間質細胞が産生するケモカインや生存因子は好酸球の局在と寿命を規定します。好酸球顆粒蛋白や脂質メディエーターは樹状細胞の成熟、移動、抗原提示を変化させる可能性があります。なお、これらを単純な「M1/M2」分類で説明するのは不十分です。

炎症時には同じ細胞が明確に病原的になり得ます。IL-5、GM-CSF、CCL11/24/26 の増加によって好酸球の生存と活性化が延長し、主要塩基性蛋白、好酸球ペルオキシダーゼ、陽イオン蛋白、ロイコトリエン、ROS の放出が上皮傷害や血管透過性亢進を引き起こします。持続すれば線維芽細胞活性化や組織リモデリングにもつながります。

臨床的には、好酸球数そのものより、活性化の程度、脱顆粒の有無、組織内局在、持続時間を見るべきです。恒常状態では修復･耐容ネットワークの一部として働き得ますが、持続する 2 型サイトカイン刺激と脱顆粒は同じネットワークを慢性炎症と組織障害へ転換させます。

Q03

質問

健常な気道粘膜および急性の微生物刺激時に、好中球は肺胞または間質マクロファージ、樹状細胞とどのように協調して宿主防御を担いながら、上皮への二次的損傷を抑えますか。

回答

健常肺では、肺胞マクロファージが気液界面における主要な骨髄系センチネルであり、吸入粒子や低レベルの微生物刺激に対する不必要な炎症を抑えています。急性の細菌･真菌刺激が生じると、肺胞 / 間質マクロファージと気道上皮は IL-1、TNF、CXCL1/2/5、ヒトでは CXCL8 などを産生し、好中球を速やかに動員します。同時に樹状細胞が微生物抗原を取り込み、後続する T 細胞応答へ橋渡しします。

好中球は貪食、酸化バースト、顆粒プロテアーゼ、抗菌ペプチドによって高強度の殺菌を担います。貪食しにくい病原体では NET 形成が補助的に働きますが、過剰な NETosis は上皮障害を招きます。マクロファージは病原体と細胞残骸の除去、サイトカイン産生、単球動員、サーファクタントの恒常性維持を担い、樹状細胞は危険シグナルの強さに応じて抗原提示を調節します。

二次的上皮障害を抑えるには、反応の時間と空間の制御が重要です。サーファクタント蛋白、抗酸化系、抗プロテアーゼは細胞外 ROS やエラスターゼの毒性を抑えます。好中球がアポトーシスに入ると、マクロファージによるエフェロサイトーシスが IL-1/TNF を低下させ、IL-10、TGF-β、リポキシン、レゾルビンなどの収束プログラムを促進します。NET 由来 DNA･ヒストンの除去、タイトジャンクション修復、粘液線毛クリアランスの回復も不可欠です。

臨床的に重要なのは、抗菌相から収束相へ移行できるかどうかです。短期間の好中球流入は肺防御に必須ですが、持続的なケモカイン、NET 蓄積、壊死性細胞死、エフェロサイトーシス不全があると、プロテアーゼと酸化ストレスを介した上皮障害が自己増幅し、重症肺炎や慢性好中球性気道炎症につながります。

Q04

質問

恒常状態および2型炎症時の気道粘膜において、好酸球はマクロファージや樹状細胞とどのように相互作用しますか。

回答

恒常状態の気道では好酸球数は少なく、肺胞マクロファージと樹状細胞が低炎症環境を維持しています。マクロファージは粒子やアポトーシス細胞を除去し、樹状細胞は抗原をサンプリングしていても、危険シグナルが乏しければ強いT細胞活性化を起こしません。ヒトの健常気道における好酸球の恒常性維持機能は、2型炎症時の役割ほど明確には定義されていません。

2型炎症が起こると、上皮由来TSLP、IL-33、IL-25がネットワークを大きく変化させます。これらのアラーミンは樹状細胞を活性化し、同時にILC2を刺激してIL-4、IL-5、IL-13優位の環境を形成します。IL-5は好酸球の生存を延長し、eotaxinは気道への集積を促進します。活性化好酸球は主要塩基性蛋白、好酸球ペルオキシダーゼ、システイニルロイコトリエン、各種サイトカインを放出し、上皮傷害、平滑筋反応、血管透過性を増強します。

樹状細胞はこの炎症を適応免疫の記憶へ変換する中心的役割を担います。IL-4/IL-13に曝露されたマクロファージは、貪食、ケモカイン産生、マトリックス再構築に関わる修復型の機能状態へ移行します。急性傷害後には有益ですが、刺激が長期化すると粘液過分泌、線維化、気道リモデリングに寄与します。好酸球由来IL-4/IL-13や脂質メディエーターはこの状態をさらに強化し得ます。

収束には、上皮アラーミンの低下、IL-5/eotaxinシグナルの減弱、好酸球アポトーシスとマクロファージによる除去が必要です。したがって同じ細胞ネットワークでも、短期的には修復を支え、持続的な上皮活性化と2型サイトカイン環境では慢性喘息を維持する方向へ働きます。

Q05

質問

尿路粘膜における初期の抗菌防御で、好中球は単球またはマクロファージ、樹状細胞とどのように協調しますか。また、過剰な組織障害を防ぐ機序は何ですか。

回答

尿路の初期抗菌防御は、尿路上皮と常在骨髄系細胞による細菌認識から始まります。膀胱上皮のTLR関連シグナルと常在マクロファージはIL-6、TNF、ヒトではCXCL8などの好中球遊走因子を産生し、補体や脂質メディエーターも動員を増強します。好中球は粘膜から尿腔へ移行し、貪食、酸化バースト、抗菌顆粒蛋白、抗菌ペプチド、状況によりNET形成を用いて尿路病原菌を迅速に減少させます。

常在マクロファージと動員単球は、初期には好中球動員と直接的な貪食を支え、感染制御後には炎症を終息させる側へ移行します。特にアポトーシス好中球のエフェロサイトーシスは、IL-10、TGF-β、炎症収束性脂質メディエーターを増加させ、上皮再生を支える重要な転換点です。樹状細胞は微生物抗原を取り込み、感染が持続･反復する場合に適応免疫を形成します。

過剰な組織障害を防ぐには、中性粒細胞の数と活性化時間を制限する必要があります。尿流と感染上皮の剥離は細菌を物理的に排出し、長時間の炎症を必要としない状況を作ります。局所の抗酸化機構や抗プロテアーゼも、ROS、エラスターゼ、NET成分による傍観者傷害を軽減します。

臨床的なトレードオフは明確です。好中球動員が不十分なら上行性感染の危険が増えますが、過剰なら上皮剥離、浮腫、出血、疼痛を悪化させます。理想的には「迅速な好中球依存性殺菌→マクロファージによる炎症残渣の除去→上皮修復」という順序で進むことが、抗菌力と組織保護を両立させます。

Q06

質問

膀胱または下部泌尿生殖器粘膜において、肥満細胞由来メディエーターはマクロファージや樹状細胞の機能にどのような影響を与えますか。また、このネットワークは宿主防御と炎症症状の双方にどのように関与し得ますか。

回答

肥満細胞は膀胱･下部泌尿生殖器の血管、感覚神経、上皮近傍に位置するため、宿主防御と症状形成の両方を結び付ける細胞です。FcεRI刺激、微生物認識、神経ペプチド刺激などにより、ヒスタミン、トリプターゼ、プロスタグランジン、ロイコトリエン、TNF、IL-6、各種ケモカインを放出します。これらは血管透過性を高め、白血球流入を促進し、マクロファージの炎症･貪食機能や樹状細胞の成熟･移動･共刺激能を変化させます。

感染時にはこの増幅は防御的です。血管透過性の上昇により血漿中の抗菌因子や循環白血球が局所へ到達しやすくなり、肥満細胞由来TNFやケモカインが好中球･単球動員を加速します。マクロファージは病原体と障害組織を処理し、樹状細胞は局所シグナルを抗原特異的免疫へつなげます。

一方、同じメディエーターが疼痛、頻尿、尿意切迫感を生み得ます。ヒスタミンや脂質メディエーターは感覚神経を感作し、トリプターゼはPAR経路を介して神経･上皮反応を変化させます。マクロファージ由来IL-1、TNF、プロスタグランジン、ROSも感覚閾値を下げます。さらに感覚神経から放出されるサブスタンスPやCGRPが肥満細胞を再刺激し、神経免疫フィードバックを形成します。上皮タイトジャンクションが障害されると尿中刺激物が粘膜下神経に到達しやすくなります。

収束には、IL-10を産生する / エフェロサイトーシスを行うマクロファージ、肥満細胞の抑制性シグナル、メディエーター分解、上皮バリア修復が必要です。慢性膀胱痛症候群はヒトで非常に不均一であり、このクロストークは重要な機序の一つですが、全症例を説明する単一機序ではありません。

Q07

質問

急性の腸管粘膜炎症とその後の炎症収束過程において、好中球とマクロファージは、遊走･動員シグナル、サイトカイン、エフェロサイトーシス、脂質メディエーター、組織修復プログラムを介してどのように相互作用しますか。

回答

急性腸管炎症では、まず上皮、常在マクロファージ、間質細胞が CXCL 系ケモカイン、IL-1、TNF、補体由来シグナル、脂質遊走因子を産生し、好中球を病変部へ動員します。好中球は貪食、ROS、顆粒蛋白、必要に応じた NET 形成で越境した微生物を抑えますが、LTB4 などを放出してさらに白血球動員を増幅するため、初期相は本質的に正のフィードバックを含みます。

マクロファージは初期抗菌反応を支える一方で、炎症を収束へ切り替える中心細胞でもあります。老化した好中球はホスファチジルセリンなどの“eat-me”シグナルを提示し、MerTK などを介してエフェロサイトーシスされます。この取り込みは単なる清掃ではなく、マクロファージの転写･脂質代謝を変え、TNF/IL-1 の高出力状態から IL-10、TGF-β、増殖因子、組織修復を優位とする状態へ移行させます。

脂質メディエーターも時間とともに切り替わります。初期のプロスタグランジンやロイコトリエンは血管反応と細胞動員を支えますが、後期にはリポキシン、レゾルビン、プロテクチン、マレシン系が追加の好中球流入を抑え、非炎症性貪食と上皮修復を促進します。好中球自身も Annexin A1 などの収束シグナルを供給し得ます。

収束が失敗するのは、好中球が除去前に二次壊死したり、NET が過剰に残存したり、マクロファージのエフェロサイトーシスが障害された場合です。その結果、DNA、ヒストン、エラスターゼ、MPO が持続的 DAMP となります。したがって好中球とマクロファージの関係は二相性であり、前半は共同抗菌、後半は共同収束へ切り替わることが粘膜回復の条件です。

Q08

質問

好酸球由来メディエーターは、腸管粘膜のマクロファージの機能状態や樹状細胞の活性化をどのように変化させますか。また、これらの相互作用は免疫寛容、組織修復、炎症のバランスをどのように変え得ますか。

回答

好酸球由来メディエーターは、腸管マクロファージと樹状細胞を修復、耐容、炎症のいずれの方向にも動かし得ます。IL-4/IL-13 は比較的確立した例で、適度な条件ではマクロファージのエフェロサイトーシス、組織修復、マトリックス再構築に関連するプログラムを促進します。好酸球由来 TGF-β、脂質メディエーター、増殖因子も上皮や間質の修復に影響します。ただし腸管マクロファージは連続的で多様な状態をとるため、単純な M1/M2 分類では不十分です。

樹状細胞も好酸球の顆粒蛋白や脂質メディエーターの影響を受けます。実験系では好酸球由来ニューロトキシンなどの陽イオン蛋白が DAMP 様に働き、樹状細胞成熟や抗原提示を高めることがあります。システイニルロイコトリエンは移動やサイトカイン産生を変化させ得ます。一方、低炎症環境では好酸球が IgA 関連応答や局所恒常性を支える可能性も示されていますが、ヒトでの証拠は炎症作用ほど一貫していません。

方向性を決めるのは刺激の強さと持続時間です。適度な IL-4/IL-13 シグナルと効率的な細胞残骸除去があれば修復に傾きます。反対に IL-5/GM-CSF で好酸球寿命が延長し、脱顆粒が持続すると、主要塩基性蛋白、好酸球ペルオキシダーゼ、ROS が上皮を傷害し、新たな危険シグナルを生みます。マクロファージと樹状細胞はその DAMPs と微生物刺激に反応して炎症を維持します。

したがって、好酸球は本質的に“寛容”でも“炎症性”でもなく、どのメディエーターが優位か、脱顆粒がどの程度か、修復終了後に抗原提示細胞が沈静化できるかによって最終的な機能が決まります。

Q09

質問

好中球性気道炎症において、好中球とマクロファージは、サイトカイン、脂質メディエーター、好中球細胞外トラップ、エフェロサイトーシス、代謝シグナルを介してどのように相互作用しますか。また、そのクロストークは炎症の持続と収束をどのように左右しますか。

回答

好中球性気道炎症では、好中球とマクロファージは一過性の抗菌協調系にも、自己維持性の炎症回路にもなり得ます。気道上皮と活性化マクロファージは IL-1β、TNF、GM-CSF、CXCL8 などを産生し、好中球の動員と生存を延長します。好中球はエラスターゼ、ROS、LTB4、各種 DAMPs を放出し、マクロファージと上皮を再刺激します。微生物由来シグナルが持続すれば、TLR やインフラマソーム経路がこの正のフィードバックをさらに固定化します。

NET は病態の分岐点です。NET 中の DNA、ヒストン、エラスターゼ、MPO は微生物を捕捉する一方、上皮傷害、喀痰粘稠化、マクロファージの危険シグナル認識を増強します。NET 成分に曝露されたマクロファージでは IL-1 系やインフラマソーム活性が上昇し得ます。これに対し、好中球が溶解性細胞死ではなくアポトーシスに入り、マクロファージにエフェロサイトーシスされると、TNF/IL-1 が低下し IL-10、TGF-β、炎症収束プログラムが誘導されます。

代謝環境も重要です。低酸素･炎症性気道では好中球と炎症性マクロファージが糖解系に依存しやすく、乳酸、コハク酸、酸化還元状態、HIF シグナルが炎症性転写を支える場合があります。収束相では、マクロファージがより酸化的で修復･エフェロサイトーシスに適した状態へ移行し、リポキシンやレゾルビンなどの特殊化炎症収束メディエーターが増加します。

したがって炎症の持続は、ケモカイン産生の持続、好中球寿命延長、NET 蓄積、エフェロサイトーシス不全、炎症性代謝環境が重なった結果です。微生物負荷が低下し、遊走勾配が消失し、アポトーシス好中球が除去されて脂質メディエーターが収束型へ切り替わると、初めて炎症は終息へ向かいます。

Q10

質問

アレルギー性気道炎症において、肥満細胞と好酸球、さらに該当する場合には好塩基球は、樹状細胞およびマクロファージとどのように相互作用して 2 型免疫を増幅または抑制しますか。

回答

アレルギー性気道炎症は、肥満細胞、好酸球、好塩基球、樹状細胞、マクロファージの相互増幅によって維持されます。アレルゲン刺激や上皮傷害により TSLP、IL-33、IL-25 が放出されると、樹状細胞と ILC2 が活性化し、樹状細胞は Th2 分化と記憶形成を促進します。その結果、IL-4、IL-5、IL-13 を中心とする 2 型サイトカイン環境が形成されます。

肥満細胞は FcεRI 結合 IgE がアレルゲンで架橋されると、ヒスタミン、トリプターゼ、PGD2、システイニルロイコトリエンを速やかに放出し、気管支収縮、血管透過性亢進、顆粒球動員を起こします。続く TNF、IL-4、IL-5、IL-13 産生は樹状細胞活性化と好酸球生存をさらに強めます。好塩基球は一部の患者や感作段階で追加の IL-4 や脂質メディエーター源となり得ます。組織内好酸球は顆粒蛋白、ロイコトリエン、サイトカインによって上皮を傷害し、アラーミン放出を再誘導します。

マクロファージは IL-4/IL-13 によって貪食、ケモカイン産生、マトリックス再構築に関わる機能状態へ変化します。急性傷害後には修復に有利ですが、慢性化すると粘液過分泌、線維化、気道リモデリングを助長し得ます。一方、アポトーシス好酸球の除去、IL-10、炎症収束性脂質メディエーター産生を通じて炎症を抑える役割もあります。

増幅か収束かを決めるのは刺激の持続です。IgE 刺激、上皮傷害、IL-5/IL-13 が持続し、エフェロサイトーシスが不十分なら慢性の正のフィードバックが成立します。反対に上皮危険シグナルが低下し、顆粒球が除去され、樹状細胞とマクロファージの活性化が減弱すれば 2 型炎症は収束へ向かいます。

Q11

質問

急性細菌性尿路感染において、動員された好中球と単球由来マクロファージは、病原体排除、サイトカイン産生、エフェロサイトーシス、粘膜修復の過程でどのように協調または競合しますか。

回答

急性細菌性尿路感染では、好中球が最も速く高強度の抗菌反応を担います。尿路上皮と常在マクロファージが病原菌を認識すると CXCL8、IL-1、TNF などが産生され、好中球が膀胱粘膜と尿腔へ動員されます。好中球は貪食、酸化バースト、顆粒抗菌蛋白、状況によっては NET 形成を用いて細菌量を迅速に低下させます。ただしこの反応が長引くと上皮障害の原因にもなります。

単球由来マクロファージはやや遅れて集積し、感染の“攻撃相”から“清掃･修復相”への切り替えに重要です。初期にはケモカイン産生、病原体貪食、壊死組織除去によって好中球と協調します。両者の競合は、同じ病原体や炎症シグナルを利用しつつ異なるサイトカイン状態を形成するという機能的競合として理解する方が適切です。TNF/IL-1 が両細胞から持続すれば組織傷害が増幅し、マクロファージが IL-10 産生とエフェロサイトーシスへ移行すれば収束が進みます。

アポトーシス好中球の除去は重要な転換点です。マクロファージはこれを取り込むことで修復関連プログラムを誘導し、剥離･損傷した尿路上皮の再生を支えます。ただし尿路病原性大腸菌などは細胞内リザーバーを形成し得るため、炎症を早く止め過ぎると菌の残存を許す可能性があります。

理想的な応答は、常に最大の炎症を維持することではありません。「好中球による迅速な菌量低下→単球 / マクロファージによる補助的除菌と残骸処理→エフェロサイトーシス→粘膜修復」という時間的順序が、感染制御と組織保護の両方に必要です。

Q12

質問

慢性膀胱粘膜炎症において、肥満細胞とマクロファージのクロストークは、感覚神経シグナル、血管透過性、上皮バリア機能、そして炎症の持続または収束をどのように変化させ得ますか。

回答

慢性膀胱粘膜炎症では、肥満細胞とマクロファージが免疫炎症を感覚神経異常へ結び付けることがあります。肥満細胞由来ヒスタミン、トリプターゼ、プロスタグランジン、ロイコトリエンは血管透過性を高め、膀胱求心性神経を直接感作します。トリプターゼは PAR 経路を介して神経･上皮反応を変化させ、神経成長因子などの神経栄養シグナルも増強し得ます。

マクロファージはこの回路を増幅も抑制もします。炎症性状態では IL-1β、TNF、プロスタグランジン、ROS、ケモカインを産生し、感覚閾値低下、内皮活性化、追加白血球動員を促します。これに対しエフェロサイトーシスを行う調節性マクロファージは IL-10、TGF-β、炎症収束メディエーターを産生して回路を抑えます。さらに感覚神経から放出されるサブスタンス P や CGRP が肥満細胞と血管細胞を再刺激し、神経免疫フィードバックを形成します。

上皮バリア障害も重要です。ヒスタミン、プロテアーゼ、炎症性サイトカインはタイトジャンクションを変化させ、尿中の刺激性溶質が粘膜下神経や免疫細胞に到達しやすくなります。慢性化すると線維芽細胞活性化、細胞外マトリックス再構築、神経支配の変化が残り、初期刺激が弱まっても症状が持続する可能性があります。

収束には、肥満細胞活性の低下、マクロファージのエフェロサイトーシス･修復状態への転換、尿路上皮バリアの再建、神経ペプチドによる正のフィードバック遮断が同時に必要です。ヒトの慢性膀胱痛･炎症症候群は不均一であるため、この機序は重要な一群を説明し得ますが、全患者に共通する単一病態とは考えるべきではありません。

Q13

質問

潰瘍性大腸炎において、好中球の動員、好中球細胞外トラップ形成、好中球代謝、ならびに好中球–マクロファージ間クロストークは、粘膜障害と炎症収束にそれぞれどのように寄与しますか。

回答

潰瘍性大腸炎では、好中球は不可欠な抗菌細胞である一方、粘膜傷害の主要因にもなります。CXCL8、IL-1、TNF、補体、脂質遊走因子が大量の好中球を大腸陰窩へ誘導し、陰窩膿瘍を形成します。好中球は貪食と酸化バーストによって細菌の組織侵入を抑えますが、反応が長期化するとエラスターゼ、MPO、ROS、その他の顆粒内容物が細胞外へ漏出し、上皮細胞とタイトジャンクションを損傷します。

NET 形成はこの傷害を増幅し得ます。NET 中の DNA、ヒストン、顆粒酵素は病原体を捕捉する一方、細胞毒性と炎症惹起性を持ち、マクロファージなどの自然免疫細胞を再刺激します。場合によっては微小血栓形成にも関与し得ます。ただし NET 自体が常に病原的なのではなく、その量、残存時間、微生物制御の必要性によって意味が変わります。

好中球は主に解糖系に依存するため、低酸素の炎症粘膜でも移動と殺菌を維持できます。解糖系とペントースリン酸経路は NADPH オキシダーゼ活性を支えますが、持続する低酸素、乳酸、炎症性代謝物は過剰活性化を維持する方向にも働きます。好中球残骸や微生物産物に曝露されたマクロファージが IL-1/TNF 高産生状態に留まると、修復への切り替えが遅れます。

収束には、アポトーシス好中球のマクロファージによるエフェロサイトーシスが不可欠です。これにより IL-10、TGF-β、収束性脂質メディエーターが増え、上皮再生とバリア回復が促されます。したがって治療目標は好中球を完全に除去することではなく、病的な持続、NET 蓄積、細胞外プロテアーゼ放出を抑えつつ、貪食性抗菌能とマクロファージ依存性修復を保つことです。

Q14

質問

クローン病において、好中球、単球由来マクロファージ、樹状細胞の相互作用の異常は、微生物処理、抗原提示、炎症の持続、上皮修復をどのように変化させますか。

回答

クローン病では、単一の骨髄系細胞が単純に“過剰”または“不足”しているというより、微生物処理、炎症増幅、組織修復の時間的協調が破綻しています。好中球はバリア破綻直後の微生物封じ込めに必要ですが、患者や病期によって動員不足、局在異常、その後の過剰持続がみられ得ます。初期の自然免疫クリアランス不全を重視するモデルと、活動性病変で多数の好中球がみられる所見は、時間軸を考えれば両立します。

単球由来マクロファージは慢性化に特に重要です。健常腸管の常在マクロファージは共生菌刺激に比較的寛容ですが、活動期に新たに流入する単球は TNF、IL-1β、IL-6、IL-23 を高産生します。NOD2 や ATG16L1 に関連する微生物認識･オートファジー生物学の異常は、細菌処理、細胞内ストレス、炎症終結を乱し得ます。残存する微生物成分がマクロファージと間質細胞を持続刺激します。

樹状細胞はこの自然免疫炎症を適応免疫へ延長します。微生物抗原を提示し、Th1/Th17 型反応を支えるサイトカインを産生します。とくに IL-23 は骨髄系細胞と T 細胞をつなぐ重要な軸です。活性化 T 細胞からのフィードバックによりマクロファージ活性化と顆粒球動員がさらに維持されます。

TNF、IL-1、ROS、プロテアーゼ、マトリックス分解酵素が高い状態では上皮修復が遅れます。一方、慢性的な修復刺激は線維狭窄にもつながり得ます。マクロファージは残骸除去、血管新生、再構築にも必要であるため、全面的な骨髄系抑制は望ましくありません。臨床的には、クローン病を「初期微生物処理異常＋炎症性単球 / マクロファージの持続＋樹状細胞による適応免疫維持＋不完全な上皮修復」の組合せとして捉えるのが妥当です。

Q15

質問

重症好中球性喘息において、好中球、マクロファージ、樹状細胞はどのように自己持続的な炎症回路を形成しますか。また、好中球細胞外トラップ、インフラマソームシグナル、微生物由来シグナル、ステロイド抵抗性はどのような役割を果たし得ますか。

回答

重症好中球性喘息では、気道上皮、マクロファージ、樹状細胞、好中球が自己維持性の炎症回路を形成し得ます。微生物、汚染物質、細胞障害に曝露された上皮とマクロファージは IL-1β、IL-6、TNF、GM-CSF、CXCL8 を産生します。樹状細胞は Th17 偏倚を支え、IL-17 は上皮･間質細胞からの好中球遊走因子産生をさらに促進します。動員された好中球はプロテアーゼ、ROS、DAMPs を放出し、上皮障害を再び増幅します。

NET はこの回路を強化します。NET の細胞外 DNA、ヒストン、エラスターゼ、MPO は粘液粘度を上げ、上皮を障害し、マクロファージや上皮のパターン認識･インフラマソーム経路を活性化します。NLRP3–IL-1β軸は一部の患者で重要な可能性がありますが、すべての好中球性喘息が同じインフラマソーム機序で説明できるわけではありません。

微生物シグナルも持続化要因です。慢性定着、気道マイクロバイオームの変化、反復感染は TLR 刺激を持続させます。一方、構造的気道病変や粘液線毛クリアランス低下が細菌定着を助長するため、関係は双方向性です。

ステロイド抵抗性も多因子性です。酸化ストレスや p38 MAPK はグルココルチコイド受容体機能を低下させ得ます。Th17 型炎症は典型的 2 型炎症よりステロイド感受性が低いことがあり、さらにステロイドは好中球アポトーシスを遅延させるため、他の炎症成分が低下しても好中球数が残る場合があります。

したがって、持続化は NET 高負荷、エフェロサイトーシス不全、IL-1/CXCL8 シグナルの継続、微生物刺激の残存が重なった結果です。臨床では感染、気管支拡張性変化、ステロイド曝露、非 2 型炎症の寄与を患者ごとに評価する必要があります。

Q16

質問

好酸球性喘息において、好酸球、肥満細胞、さらに該当する場合には好塩基球は、マクロファージ、樹状細胞、気道上皮とどのように相互作用して 2 型炎症を開始･増幅･収束させますか。

回答

好酸球性喘息は気道上皮と免疫系の境界から始まります。アレルゲン、ウイルス、上皮ストレスにより TSLP、IL-33、IL-25 が放出され、樹状細胞が調節されると同時に ILC2 が活性化されます。樹状細胞は Th2 分化と記憶を促進し、Th2 と ILC2 は IL-4、IL-5、IL-13 を産生します。IL-5 は好酸球の成熟･生存を支え、eotaxin は気道への集積を促進し、IL-4/IL-13 は IgE 産生、粘液化生、2 型組織反応を拡大します。

肥満細胞は IgE/FcεRI 経路を介して増幅します。アレルゲンによる架橋でヒスタミン、トリプターゼ、PGD2、システイニルロイコトリエンを放出し、気管支収縮、血管透過性亢進、白血球動員を起こします。好塩基球は状況により IL-4 や脂質メディエーターの追加供給源となります。組織内好酸球は主要塩基性蛋白、好酸球ペルオキシダーゼ、ロイコトリエン、サイトカインを放出して上皮を傷害し、アラーミン放出を維持します。

マクロファージも重要です。IL-4/IL-13 は貪食、ケモカイン産生、マトリックス再構築を変化させ、短期には修復に寄与しますが、慢性刺激下では粘液、線維化、気道リモデリングを促進し得ます。アポトーシス好酸球のエフェロサイトーシスは収束に不可欠であり、上皮アラーミンや抗原曝露が低下すれば樹状細胞活性も減弱します。

収束には、上皮危険シグナルの低下、IL-5 依存性好酸球生存の終了、IgE 駆動性肥満細胞活性化の減少、効率的なエフェロサイトーシスが同時に必要です。2 型生物学的製剤の臨床効果はこのネットワークの妥当性を支持しますが、固定性リモデリング、感染、非 2 型炎症が残れば、好酸球炎症を抑えても症状が完全には消失しないことがあります。

Q17

質問

反復性尿路感染において、過去の感染は、訓練免疫、免疫寛容、または組織リモデリングを介して膀胱粘膜の好中球および単球 / マクロファージ応答をどのように再構成し得ますか。また、それぞれの機序を支持するエビデンスはどの程度確立されていますか。

回答

反復性尿路感染では、過去の感染が膀胱粘膜に複数の形で“記憶”を残し得ますが、訓練免疫、免疫寛容、組織リモデリングのエビデンス強度は同じではありません。比較的確立しているのは組織リモデリングです。感染は尿路上皮剥離、再生性増殖、間質シグナル、局所神経支配を変化させ、尿路病原菌は細胞内リザーバーとして残存することもあります。これらの構造的･微生物学的変化は症状消失後も次回感染時の好中球動員やマクロファージ活性化閾値を変え得ます。

訓練免疫は機序的には十分妥当ですが、ヒト反復性尿路感染での直接証拠は限定的です。反復するパターン認識受容体刺激は骨髄前駆細胞や単球に代謝･エピジェネティック変化を残し、解糖系の準備状態や炎症遺伝子周辺のクロマチン可及性を変え得ます。その結果、次回感染で新たに動員される単球 / マクロファージがより迅速に反応する可能性があります。成熟好中球は短命なので、持続的な“訓練”は個々の好中球より前駆細胞や組織環境の変化で説明する方が妥当です。

免疫寛容は逆方向の適応です。反復 TLR 刺激により TNF や IL-1 産生が低下し、組織傷害を抑える一方、初期警告反応が弱くなって細菌残存を許す可能性があります。ヒトでどの程度この耐容状態が再発に寄与するかは明確ではありません。

現実的には複数機序が併存すると考えるべきです。組織リモデリングと細胞内細菌残存の証拠は比較的強く、自然免疫応答閾値の変化は中等度で主に実験的、ヒト膀胱で安定した好中球･マクロファージ訓練状態が存在するという証拠はまだ予備的です。確立した病態機序とするには、縦断的かつ細胞特異的な機能･エピジェネティック研究が必要です。

Q18

質問

膀胱がんにおいて、腫瘍関連好中球と腫瘍関連マクロファージは粘膜腫瘍微小環境内でどのように相互作用し、抗原提示、T 細胞活性、血管新生、浸潤、免疫療法への反応に影響しますか。

回答

膀胱がんでは、腫瘍関連マクロファージ（TAM）と腫瘍関連好中球（TAN）が相互に増強する骨髄系免疫抑制環境を形成し得ます。腫瘍細胞と間質細胞は CCL2、CSF1、CXCL8、G-CSF などを分泌して単球と好中球を継続的に動員し、腫瘍内の低酸素、TGF-β、IL-10、プロスタグランジン、代謝ストレスがこれらを免疫抑制･組織再構築型の状態へ傾けます。

TAM は共刺激能を低下させ、PD-L1 などの抑制性分子を発現し、IL-10、TGF-β、アルギニン代謝関連因子、ROS などで T 細胞機能を抑制します。TAN も ROS、プロテアーゼ、アルギニン代謝、状況によっては NET 形成を介して T 細胞を抑えます。両者はケモカインや増殖因子を介して相互の動員･生存を支えるため、腫瘍抗原が存在しても細胞傷害性 T 細胞が腫瘍へ入りにくい、あるいは機能を失う状態が生じます。

血管新生と浸潤にも関与します。VEGF、MMP、エラスターゼなどが血管･細胞外マトリックスを再構築し、実験系では NET が腫瘍細胞接着や播種を促進する可能性も示されています。なお、実際の TAM/TAN は固定した M1/M2、N1/N2 の二分法ではなく、腫瘍の悪性度、低酸素、治療歴に応じた連続的状態をとります。

免疫療法への反応にも影響します。強い骨髄系抑制状態はチェックポイント阻害への抵抗性につながり得る一方、局所自然免疫活性化は抗腫瘍免疫を促進します。膀胱内 BCG 療法はその臨床的実例で、好中球･マクロファージを含む急性炎症を誘導して抗腫瘍環境を形成しますが、炎症毒性もあります。

したがって治療目標は全面的な骨髄系細胞除去ではなく、腫瘍内での選択的再プログラムです。尿路感染防御を担う同じ細胞を損なわない空間的･時間的選択性が重要です。

Q19
質問
潰瘍性大腸炎において、病原性の好中球動員または好中球細胞外トラップ関連障害を抑えつつ、抗微生物防御とマクロファージによる修復を可能な限り温存するには、どのような機序的に妥当な戦略が考えられますか。予想される利点、トレードオフ、エビデンスの不足を説明してください。

回答
潰瘍性大腸炎では、好中球を全面的に除去するより、病的な持続動員と細胞外毒性を選択的に抑える方が機序的に合理的です。第一に、CXCL8–CXCR1/2 軸を部分的または局所的に抑えることで、陰窩への過剰集積、プロテアーゼ、ROS 放出を減らす方法があります。ただし全身性に強く遮断すると細菌封じ込めが低下するため、粘膜局所投与、可逆的抑制、好中球高炎症型の患者選択が望まれます。

第二に NET 関連傷害を狙う方法があります。PAD4 阻害は一部の NET 形成プログラムを抑制し、DNase は細胞外 DNA の除去を促進します。細胞外ヒストン、エラスターゼ、MPO を中和すれば、貪食能を比較的温存しながら組織毒性を減らせる可能性があります。ただし NET は細胞外微生物制御にも寄与し、DNase で DNA 骨格を分解すると活性プロテアーゼが遊離する可能性があります。また NET 形成は必ずしも PAD4 依存性ではありません。

第三に、炎症収束を強化する戦略です。Annexin A1、リポキシン、レゾルビンなどの経路や、MerTK 関連エフェロサイトーシスを促進すれば、好中球を完全に遮断せずに組織滞在時間を短縮し、マクロファージの IL-10･修復プログラムを高められます。上皮バリアを回復し微生物移行を減らすことも、上流の好中球動員刺激を低下させます。

予想される利点は、細胞内殺菌とマクロファージ依存性修復を残しながら、NET、プロテアーゼ、酸化傷害を減らせることです。主なトレードオフは感染、微生物除去遅延、創傷デブリードマン低下です。多くの NET / 収束標的はなお前臨床段階で、ヒト粘膜の薬力学指標も標準化されていません。最も現実的なのは局所･短時間･エンドタイプ選択型の介入です。

Q20
質問
炎症性腸疾患において、腸管マクロファージと顆粒球の代謝的またはエピジェネティックなリプログラミングを粘膜恒常性の回復にどのように利用できるでしょうか。また、効果消失や意図しない免疫抑制をもたらし得る機序は何ですか。

回答
炎症性腸疾患における代謝･エピジェネティック再プログラムの狙いは、骨髄系細胞を除去するのではなく機能状態を変えることです。炎症性マクロファージでは解糖系、HIF、mTOR、コハク酸 / イタコン酸代謝、NAD+･酸化還元バランスの変化がみられます。AMPK に関連する酸化代謝を支え、ミトコンドリア機能や免疫代謝物を調節できれば、IL-1β や TNF を下げつつ貪食、エフェロサイトーシス、修復機能を温存できる可能性があります。これは単純な“M1 から M2 への転換”より複雑なネットワーク変化です。

エピジェネティックには、持続炎症性単球でエンハンサー可及性、ヒストンアセチル化 / メチル化、訓練免疫様クロマチン状態が変化し得ます。そのため BET、HDAC、ヒストン修飾、DNA メチル化関連経路は候補になります。局所･細胞選択的に操作できれば、過剰な炎症転写を抑え、より寛容･エフェロサイトーシス･修復寄りの状態を回復できる可能性があります。

顆粒球では安全域が狭い点が重要です。好中球は移動、酸化バースト、NET 形成に解糖系とペントースリン酸経路を強く依存するため、代謝を強く抑えると組織傷害だけでなく抗菌能も低下します。したがって、過剰 NETosis、異常な生存延長、細胞外プロテアーゼを選択的に抑える方が合理的です。

効果消失は代謝可塑性、代替サイトカイン、微生物移行の持続、新規単球の補充で起こり得ます。意図しない免疫抑制は、同じ経路が感染防御、創傷治癒、ワクチン応答にも必要なことから生じます。全身性エピジェネティック薬は上皮、リンパ球、造血前駆細胞にも影響します。したがって臨床応用には粘膜標的化、短期投与、細胞状態とバリア修復を測るバイオマーカーが必要で、現時点では多くが実験段階です。

Q21
質問
2 型サイトカインシグナルを変化させる生物学的製剤は、単に好酸球数を減らすだけでなく、気道粘膜の好酸球–肥満細胞–マクロファージ–樹状細胞ネットワークをどのように再構成し得ますか。

回答
2 型サイトカインを標的とする生物学的製剤は、好酸球数だけでなく気道全体の骨髄系ネットワークを再構成します。IL-5 または IL-5R を遮断すると、好酸球の成熟、生存、組織滞在が減少し、同時に好酸球由来ロイコトリエン、顆粒蛋白、サイトカインが減ります。その結果、肥満細胞、マクロファージ、樹状細胞、上皮が受ける二次刺激も低下します。

IL-4Rα遮断は IL-4 と IL-13 の両方を抑えるため、上皮 eotaxin、粘液化生、IgE を支える環境を低下させると同時に、IL-4/IL-13 で形成されていたマクロファージの転写プログラムを直接変えます。これにより粘液、線維化、リモデリングに関わるマクロファージ機能が弱まる可能性があります。上皮環境や抗原曝露が変わるため、樹状細胞と T 細胞の相互作用も二次的に変化します。

TSLP など上流アラーミンを標的とする治療はさらに早い段階で「上皮—樹状細胞—Th2/ILC2」軸を弱めるため、好酸球、肥満細胞、好塩基球の後続活性化を広く抑えます。IL-33 関連経路も同様に広いネットワーク効果が期待されます。抗 IgE 療法は主に FcεRI 依存性の肥満細胞･好塩基球活性化を低下させますが、その結果として樹状細胞･マクロファージが曝露される炎症環境も弱まります。

これらは炎症収束にも有利です。活性化好酸球の除去負荷が減り、上皮アラーミンの増幅も抑えられます。ただし固定性リモデリング、感染、非2型炎症は残り得ます。したがって臨床的には、単なる好酸球減少ではなく、顆粒球生存･脱顆粒、上皮アラーミン、マクロファージ再構築、樹状細胞による2型記憶支持を同時に弱める“ネットワーク再平衡”と捉えるのが適切です。

Q22

質問

好中球性喘息において、抗菌防御を維持しながら好中球–マクロファージ炎症回路を遮断するために、どのような骨髄系細胞標的戦略が考えられますか。また、提案されている機序のうち、臨床的に確立しているものと前臨床段階にあるものを区別してください。

回答

好中球性喘息では、CXCL8/IL-1—好中球—マクロファージ回路を遮断しながら細菌防御を保つことが治療上の核心です。現時点で、この条件を安定して満たす骨髄系特異的な標準治療は確立していません。マクロライドは一部の重症喘息で補助療法として比較的臨床的根拠があり、増悪抑制や好中球、マクロファージ、上皮のサイトカイン調節が関与すると考えられます。しかし骨髄系選択的ではなく、長期使用では抗菌薬耐性やマイクロバイオームへの影響が問題になります。

CXCR2拮抗は好中球遊走を直接減らすため機序的には魅力的です。臨床試験で喀痰･血中好中球の低下は示されていますが、症状や増悪への効果は一貫せず、感染リスクもあります。したがって“臨床で検証されたが標準治療として確立していない”段階です。IL-1、IL-17などを広く遮断する方法も、普遍的に有効な好中球性喘息治療には至っていません。

前臨床･初期臨床段階では、NLRP3阻害によるマクロファージIL-1β抑制、PAD4阻害やDNaseによるNET負荷低下、細胞外ヒストン中和、好中球プロテアーゼ阻害などが考えられます。さらにマクロファージのエフェロサイトーシスや特殊化炎症収束メディエーターを強化する戦略は、好中球動員そのものを完全に止めずに“使い終わった好中球を速やかに除去する”点で魅力があります。

抗菌防御を守るには、局所･可逆的･エンドタイプ選択型の介入が重要です。気管支拡張、慢性細菌定着、反復感染がある患者に強い全身性好中球抑制は適しません。臨床的に確立している部分は限定的で、多くの骨髄系標的機序はなお研究段階です。

Q23

質問

訓練免疫または粘膜ワクチンのアプローチによって、単球 / マクロファージと好中球を有益にリプログラムし、反復性尿路感染を減らすことは可能でしょうか。想定される機序、組織特異的な潜在的リスク、現時点のエビデンスの限界を説明してください。

回答

訓練免疫や粘膜ワクチンは、膀胱を慢性的に炎症状態へ置くのではなく、再曝露時の抗菌反応をより速く効率的にすることで反復性尿路感染を減らせる可能性があります。理想的な粘膜ワクチンは病原体特異的抗体、組織常在リンパ球、迅速な局所免疫起動を形成し、オプソニン化を通じて好中球とマクロファージの貪食効率を高めます。これにより高強度炎症が必要な時間を短縮できます。

訓練免疫は自然免疫側の再プログラムです。パターン認識受容体刺激により骨髄前駆細胞や単球に代謝･エピジェネティック変化が生じ、次回感染で貪食、遊走、抗菌エフェクターをより迅速に起動できる可能性があります。望ましいのは抗菌能を高めつつ、過剰なIL-1/TNFやNET形成を誘導しない状態です。成熟好中球は短命であるため、持続効果は前駆細胞や組織環境の再プログラムで説明する方が現実的です。

リスクは組織特異性にあります。過度に“プライム”された膀胱粘膜では、好中球過剰動員、上皮傷害、疼痛、尿意切迫、線維化が起こり得ます。反復自然免疫刺激はマイクロバイオームを変えたり、訓練ではなく耐容を誘導して本番の感染時に反応を弱めたりする可能性もあります。全身性訓練が起これば他臓器の炎症を悪化させる懸念もあります。

現時点のエビデンスは有望ですが不十分です。実験研究では自然免疫状態の持続的変化が示され、ワクチンや細菌由来製剤も臨床的に検討されていますが、製剤、評価項目、研究品質は均一ではありません。とくにヒトでの利益が単球 / マクロファージや好中球の訓練性再プログラムによって生じたと直接証明したデータは乏しいため、現段階では確立治療というより有望な研究概念です。

Q24

質問

腫瘍関連マクロファージと好中球を選択的にリプログラムすることで、保護的な尿路粘膜免疫への影響を最小限にしつつ、膀胱がんの抗腫瘍免疫をどのように高められるでしょうか。機序的アプローチと主要なトランスレーショナル上の障壁を比較してください。

回答

膀胱がんでの選択的骨髄系再プログラムは、TAMとTANを免疫抑制･血管新生･組織再構築型から、短時間･局所的な抗腫瘍エフェクターへ変えることを目標とします。同時に尿路感染に対する防御能は温存する必要があります。膀胱は膀胱内投与が可能であり、全身性骨髄系抑制を避けながら腫瘍と粘膜へ直接作用させられる点が大きな利点です。

マクロファージでは、CSF1/CSF1RやCCL2による募集の抑制、PI3Kγ様の免疫抑制シグナル阻害、TLR/STINGなど自然免疫経路の活性化、CD47–SIRPα遮断による腫瘍細胞貪食促進が考えられます。理想的には腫瘍抗原取り込みと炎症性抗原提示を高め、IL-10、TGF-β、VEGF優位の抑制環境を弱めます。抗菌･修復機能を考えると、全面的マクロファージ除去より機能再プログラムの方が望ましいです。

好中球ではCXCR2軸を抑えて免疫抑制性TANの動員を減らし、PAD4/NET、エラスターゼ、TGF-β関連経路を標的として浸潤促進･免疫抑制機能を減らす方法が考えられます。ただし好中球は尿路細菌防御の第一線であるため、慢性的な全身性抑制は感染リスクが高く、局所･間欠投与が合理的です。

これらはチェックポイント阻害薬と相乗し、T 細胞流入を改善する可能性があります。膀胱内 BCG 療法は局所自然免疫活性化が臨床的抗腫瘍効果を生むことの実例ですが、選択的 TAM/TAN 再プログラムではなく炎症毒性もあります。

主なトランスレーショナル障壁は、骨髄系細胞の高い可塑性、安定したヒト TAM/TAN マーカーの不足、腫瘍不均一性、代償性遊走経路、薬剤浸透、感染リスクです。最も現実的なのは局所･可逆･併用型の戦略で、腫瘍内抑制性骨髄系細胞と保護的尿路髄系細胞を空間的･機能的に区別するバイオマーカーが必要です。

Simplified Chinese (SC)

Anonymous Model D | 简体中文

Anonymous Model D
简体中文
24 standardized questions

Q01

问题

在健康肠黏膜中，中性粒细胞如何在与驻留巨噬细胞和树突状细胞相互作用的同时参与屏障防御？哪些机制通常限制中性粒细胞介导的组织损伤？

回答

健康肠黏膜中的中性粒细胞并不是长期占优势的常驻细胞，而是一个“按需快速动员”的效应群体。上皮屏障出现微小破损、细菌产物越过黏液层或局部模式识别受体被激活后，上皮细胞、基质细胞和驻留巨噬细胞可产生 CXCL1/CXCL2 以及人类中更典型的 CXCL8，并联合补体和脂质趋化信号募集粒细胞。到达局部后，中性粒细胞通过吞噬、NADPH 氧化酶依赖的氧爆发、抗菌颗粒释放及在特定情况下形成 NETs，对进入组织的细菌和真菌进行快速控制。关键并不在于“炎症强度越大越好”，而在于效应尽量局限于屏障破损处。

驻留巨噬细胞承担重要的调节作用。健康肠道巨噬细胞能够持续吞噬微生物和细胞碎片，却对共生菌刺激相对低反应，并通过 IL-10 等信号限制不必要的炎症放大。树突状细胞则负责抗原采样，并在真正存在感染风险时决定是否需要启动适应性免疫。中性粒细胞释放的蛋白酶、脂质介质和危险信号也会反过来影响巨噬细胞和树突状细胞，但在稳态下这种串扰通常是短暂而可终止的。

限制组织损伤依赖主动的炎症消退程序。完成杀菌后的中性粒细胞进入凋亡，由巨噬细胞通过 MerTK、AXL 等相关通路进行凋亡细胞清除。这个过程会抑制 TNF、IL-1 等促炎输出，并促进 IL-10、TGF-β及组织修复程序。脂氧素、消退素等促消退脂质介质、腺苷、Annexin A1、黏液层和上皮抗蛋白酶共同减少弹性蛋白酶、髓过氧化物酶、ROS 和 NET 成分的旁观者损伤。

因此，健康状态下的核心是“快速募集—局部杀菌—及时清除—屏障修复”。如果募集持续、NETs 堆积、中性粒细胞发生继发性坏死或巨噬细胞凋亡细胞清除受损，同一套防御机制就会转化为黏膜损伤机制，这也是炎症性肠病中中性粒细胞从保护性向致病性转变的重要生物学基础。

Q02

问题

在健康肠黏膜中，嗜酸性粒细胞如何参与维持稳态并与巨噬细胞或树突状细胞进行通讯？这些作用与炎症状态下嗜酸性粒细胞的功能有何不同？

回答

肠道嗜酸性粒细胞的特点是：即使没有明显炎症，固有层中也可存在相当数量的细胞。其维持与 IL-5、CCL11 等 eotaxin 家族趋化因子有关。稳态时，它们更像是黏膜免疫和组织生态中的调节者，参与上皮/基质稳态、细胞外基质周转、免疫监视以及局部体液免疫；动物研究还提示其可支持浆细胞和 IgA 相关反应，但这些作用在人类不同肠段中的重要性并不完全一致。

嗜酸性粒细胞与巨噬细胞、树突状细胞存在双向通讯。嗜酸性粒细胞来源的 IL-4、IL-13 可推动部分巨噬细胞进入更偏向吞噬、修复和基质重塑的功能状态；TGF-β、脂质介质和生长因子也可影响局部修复。反过来，巨噬细胞和基质来源的趋化因子、存活信号会调节嗜酸性粒细胞的募集和寿命。嗜酸性粒细胞的颗粒蛋白及脂质介质还可能改变树突状细胞的迁移、成熟和抗原呈递。临床上应避免把这些变化简单等同于“M1/M2”二分法，因为肠道巨噬细胞实际上呈连续谱状态。

炎症时，功能重点明显改变。IL-5、GM-CSF 和 CCL11/24/26 升高会延长嗜酸性粒细胞存活并增强脱颗粒。主要碱性蛋白、嗜酸性粒细胞过氧化物酶、阳离子蛋白、白三烯和 ROS 可直接损伤上皮、增加血管通透性并维持局部炎症，也可能促进成纤维细胞活化和慢性重塑。

因此，判断嗜酸性粒细胞是保护还是致病，不能只看数量，而要看激活程度、颗粒释放方式、组织位置及持续时间。稳态下它们更多嵌入耐受和修复网络；持续的 2 型细胞因子暴露和脱颗粒则可把同一网络推向慢性炎症和组织损伤。

Q03

问题

在健康呼吸道黏膜以及急性微生物攻击期间，中性粒细胞如何与肺泡或间质巨噬细胞及树突状细胞协同，在发挥宿主防御作用的同时限制对上皮的旁观者损伤？

回答

健康肺内，肺泡巨噬细胞是气液界面的主要髓系“哨兵”，其重要任务之一是对无害颗粒和低水平微生物刺激保持克制，从而避免不必要的肺泡炎症。急性细菌或真菌刺激出现后，肺泡/间质巨噬细胞及气道上皮通过 IL-1、TNF、CXCL1/2/5 以及人类 CXCL8 等信号迅速募集中性粒细胞；树突状细胞同时摄取抗原，为后续 T 细胞应答提供连接。

中性粒细胞通过吞噬、氧爆发、颗粒蛋白和抗菌肽进行高效杀菌，遇到难以吞噬的病原体时可形成 NETs。巨噬细胞则补充吞噬、细胞因子调度、肺泡碎片和表面活性物质清除，并决定是否继续募集单核细胞。树突状细胞根据危险信号强度调整成熟和抗原呈递。这个阶段三类细胞形成协同防御，但其理想状态应当是短暂、空间受限。

限制上皮旁观者损伤主要依靠“终止信号”。中性粒细胞完成任务后进入凋亡，由巨噬细胞进行凋亡细胞清除，从而降低 IL-1/TNF 输出并促进 IL-10、TGF-β以及脂氧素、消退素等促消退通路。肺泡表面活性物质、抗氧化体系和抗蛋白酶可中和外泄的 ROS 和弹性蛋白酶；NET DNA 和组蛋白也需要及时清除，随后恢复紧密连接和黏液纤毛清除功能。

从临床病理角度看，真正决定结局的是是否能从“杀菌期”顺利切换到“消退期”。适度、短暂的中性粒细胞流入对于肺部抗菌是必要的；持续趋化、NETs 积聚、坏死性细胞死亡或巨噬细胞凋亡细胞清除失败，则会形成蛋白酶—氧化应激—上皮损伤的正反馈，见于重症肺炎、急性肺损伤和部分慢性中性粒细胞性气道疾病。

Q04

问题

在稳态条件和 2 型炎症期间，嗜酸性粒细胞如何在气道黏膜中与巨噬细胞和树突状细胞相互作用？

回答

稳态气道中嗜酸性粒细胞数量较低，局部环境主要由肺泡巨噬细胞和树突状细胞维持低炎症状态。巨噬细胞负责清除颗粒及凋亡细胞，树突状细胞虽然持续进行抗原采样，但在缺乏危险信号时并不会强烈推动 T 细胞反应。人类健康气道中嗜酸性粒细胞的具体稳态功能尚不如其在 2 型炎症中的作用明确。

发生 2 型炎症时，气道上皮受到变应原、病毒或机械损伤后释放 TSLP、IL-33 和 IL-25。这些上皮警报素一方面激活树突状细胞，另一方面刺激 ILC2，继而形成 IL-4、IL-5、IL-13 为主的环境。IL-5 延长嗜酸性粒细胞存活，eotaxin 促进其进入气道。活化嗜酸性粒细胞释放主要碱性蛋白、嗜酸性粒细胞过氧化物酶、半胱氨酰白三烯及多种细胞因子，可损伤上皮、影响平滑肌和血管反应，并继续加强抗原呈递细胞的激活。

树突状细胞决定了反应是否形成持续的适应性记忆；IL-4/IL-13 则把巨噬细胞推向偏修复和重塑的功能谱。急性损伤后这种状态可以帮助清除细胞残骸、重建组织，但长期存在时会参与黏液高分泌、纤维化和气道重塑。嗜酸性粒细胞产生的 IL-4/IL-13 及脂质介质可进一步巩固这种巨噬细胞状态。

炎症消退需要上皮警报素下降、IL-5/eotaxin 信号减弱、嗜酸性粒细胞进入凋亡并被巨噬细胞清除，同时由 IL-10 和促消退脂质介质抑制剩余炎症。也就是说，同一套嗜酸性粒细胞—巨噬细胞—树突状细胞网络，在短暂刺激后可服务于修复，而在持续上皮激活和 2 型细胞因子驱动下则成为慢性哮喘的维持机制。

Q05

问题

在尿路黏膜中，早期抗菌防御期间，中性粒细胞如何与单核细胞或巨噬细胞及树突状细胞协同？哪些机制可防止过度组织损伤？

回答

尿路早期抗菌反应主要从尿路上皮和驻留髓系细胞对细菌产物的识别开始。膀胱上皮 TLR 相关信号及驻留巨噬细胞可迅速产生 IL-6、TNF 和 CXCL8 等中性粒细胞趋化因子，补体片段和脂质介质进一步增强募集。中性粒细胞进入黏膜和尿腔后，通过吞噬、氧爆发、抗菌肽/颗粒蛋白释放以及部分情境下的 NET 形成，迅速降低尿路致病菌负荷。

驻留巨噬细胞和后续募集的单核细胞承担两阶段作用：早期通过细胞因子和趋化因子支持中性粒细胞募集，并直接吞噬细菌；后期则清除坏死细胞和凋亡中性粒细胞，使局部环境从“杀菌模式”转向“修复模式”。树突状细胞摄取微生物抗原，并在感染持续或复发时促进适应性免疫。中性粒细胞杀菌也会改变抗原量和危险信号强度，从而间接影响树突状细胞功能。

防止过度损伤的关键，是限制中性粒细胞活化的持续时间。尿流和上皮脱落可物理性清除细菌及被感染细胞，减少长期高强度炎症的必要性。中性粒细胞凋亡后被巨噬细胞进行凋亡细胞清除，可增加 IL-10、TGF-β和促消退脂质介质，并促进上皮再生。局部抗氧化体系和抗蛋白酶也能限制 ROS、弹性蛋白酶和 NET 成分对尿路上皮的旁观者损伤。

临床上的核心权衡是：募集不足会增加上行感染风险，而募集过度则造成黏膜脱落、水肿、出血和疼痛。理想反应应按时间顺序完成——先以中性粒细胞快速控制细菌，再由巨噬细胞清除炎症残骸并启动上皮修复。

Q06

问题

在膀胱或下泌尿生殖道黏膜中，肥大细胞释放的介质如何影响巨噬细胞和树突状细胞的功能？这一网络如何同时参与宿主防御和炎症相关症状？

回答

肥大细胞位于膀胱和下泌尿生殖道的血管、感觉神经和上皮附近，因此很容易成为连接“感染防御”和“症状产生”的枢纽。受到 IgE/FcεRI、微生物模式识别或神经肽刺激后，肥大细胞可释放组胺、胰蛋白酶样蛋白酶、前列腺素、白三烯、TNF、IL-6 及多种趋化因子。这些介质增加血管通透性和白细胞进入，同时可增强巨噬细胞炎症反应，并促进树突状细胞成熟、迁移和共刺激能力。

在感染背景下，这种放大是有价值的。血管通透性增加有助于血浆抗菌成分和循环白细胞进入局部；肥大细胞来源的 TNF 和趋化因子可加快中性粒细胞、单核细胞募集；巨噬细胞负责吞噬病原体和坏死组织，树突状细胞则将局部感染信号连接到特异性免疫。

但同样的网络也可产生炎症症状。组胺和脂质介质可直接敏化感觉神经；肥大细胞蛋白酶通过 PAR 等受体改变神经和上皮反应。巨噬细胞产生的 IL-1、TNF、前列腺素和 ROS 进一步降低感觉阈值。感觉神经释放 P 物质、CGRP 等神经肽后，又可能反过来激活肥大细胞，形成神经—免疫正反馈。与此同时，蛋白酶和炎症因子削弱上皮紧密连接，使尿液中的刺激性溶质更容易接触黏膜下神经末梢。

能否消退取决于 IL-10 型/凋亡细胞清除型巨噬细胞、肥大细胞抑制性受体、介质降解和上皮屏障修复是否及时恢复。慢性膀胱疼痛综合征在人群中高度异质，因此肥大细胞—巨噬细胞串扰应理解为重要的机制亚型之一，而不是所有患者共同的单一病因。

Q07

问题

在急性肠黏膜炎症及其后的炎症消退阶段，中性粒细胞与巨噬细胞如何通过募集信号、细胞因子、凋亡细胞清除、脂质介质和组织修复程序相互影响？

回答

急性肠黏膜炎症首先进入募集和杀菌阶段。受损上皮、驻留巨噬细胞和基质细胞释放 CXCL 趋化因子、IL-1、TNF、补体信号及脂质趋化物，中性粒细胞沿梯度进入黏膜。它们通过吞噬、ROS、颗粒蛋白和部分情况下的 NETs 控制越过屏障的微生物；同时，中性粒细胞自身释放 LTB4 等介质，又可继续放大白细胞募集，因此早期反应具有明显的正反馈特征。

巨噬细胞既参与早期抗菌，也负责把炎症“刹车”。中性粒细胞完成任务后逐渐凋亡并暴露磷脂酰丝氨酸等“eat-me”信号，巨噬细胞通过 MerTK 等受体进行凋亡细胞清除。这个过程会重塑巨噬细胞的转录和脂质代谢状态，使 TNF/IL-1 输出下降，而 IL-10、TGF-β、修复因子和组织重建程序上升。因此，凋亡细胞清除并不是被动清垃圾，而是炎症由进攻期切换到消退期的关键开关。

脂质介质也发生“类别转换”：早期前列腺素、白三烯偏向募集和血管反应，后期脂氧素、消退素、保护素和 maresin 类介质则减少进一步中性粒细胞进入、促进非炎性吞噬并帮助上皮修复。中性粒细胞自身也可提供 Annexin A1 等促消退信号。

若中性粒细胞在被清除前发生继发坏死或大量 NET 形成，DNA、组蛋白、弹性蛋白酶和髓过氧化物酶会变成持续的危险信号；若巨噬细胞凋亡细胞清除能力不足，促炎细胞因子也难以下降。临床上，这种“先协同抗菌、后协同消退”的双相关系，决定了急性炎症能否恢复正常黏膜结构。

Q08

问题

嗜酸性粒细胞来源的介质如何改变肠黏膜巨噬细胞的功能状态和树突状细胞的激活？这些相互作用如何推动免疫耐受、组织修复与炎症之间的平衡发生变化？

回答

嗜酸性粒细胞来源的介质可以把肠道巨噬细胞和树突状细胞推向修复、耐受或炎症中的不同方向。IL-4 和 IL-13 是较明确的例子：在一定范围内可促进巨噬细胞的凋亡细胞清除、组织修复和基质重塑程序。嗜酸性粒细胞还可产生 TGF-β、脂质介质及生长因子，从而影响上皮和基质细胞。需要强调的是，肠道巨噬细胞的状态远比传统“M1/M2”二分复杂，其最终表型同时受微生物群、上皮信号和新募集单核细胞影响。

树突状细胞可受到嗜酸性粒细胞颗粒蛋白和脂质介质的调节。部分实验系统中，嗜酸性粒细胞来源神经毒素等阳离子蛋白可表现为危险信号，增强树突状细胞成熟或抗原呈递；半胱氨酰白三烯等则可改变树突状细胞迁移及细胞因子输出。另一方面，在低炎症环境中，嗜酸性粒细胞也可能参与支持 IgA 和局部免疫稳态的网络，但这些稳态作用在人类中的证据没有炎症效应那么一致。

决定方向的核心是激活强度和持续时间。适度 IL-4/IL-13 信号、伴随有效的细胞碎片清除，可促进屏障修复；如果 IL-5/GM-CSF 持续延长嗜酸性粒细胞寿命并出现明显脱颗粒，则主要碱性蛋白、过氧化物酶和 ROS 会造成上皮损伤，形成新的危险信号，继而让巨噬细胞和树突状细胞继续维持炎症。

因此，嗜酸性粒细胞本身既不是固定“耐受性”也不是固定“致炎性”。其作用取决于哪些介质占优势、是否发生广泛脱颗粒以及巨噬细胞/树突状细胞能否在修复完成后及时退出激活状态。

Q09

问题

在中性粒细胞性气道炎症中，中性粒细胞与巨噬细胞如何通过细胞因子、脂质介质、中性粒细胞胞外诱捕网、凋亡细胞清除和代谢信号相互影响？这种串扰如何决定炎症持续还是消退？

回答

在中性粒细胞性气道炎症中，中性粒细胞和巨噬细胞既可以形成短暂有效的抗菌协作，也可以形成自我维持的炎症回路。气道上皮和活化巨噬细胞释放 IL-1β、TNF、GM-CSF 和 CXCL8 等信号，持续募集并延长中性粒细胞寿命；中性粒细胞则通过弹性蛋白酶、ROS、LTB4 和危险相关分子反过来刺激巨噬细胞和上皮。当微生物信号长期存在时，TLR 和炎症小体通路会进一步固定这一正反馈。

NETs 是决定结局的重要分叉点。NET 中的 DNA、组蛋白、弹性蛋白酶和髓过氧化物酶可捕获病原体，但同时会损伤上皮、增加痰液黏稠度，并激活巨噬细胞的危险识别和 IL-1 通路。相反，如果中性粒细胞以凋亡方式结束其效应期并被巨噬细胞及时清除，巨噬细胞会减少促炎细胞因子输出，并增加 IL-10、TGF-β以及促消退相关程序。

代谢环境也会影响这种串扰。低氧、炎症性气道往往促使中性粒细胞和炎症型巨噬细胞更多依赖糖酵解；乳酸、琥珀酸、氧化还原状态和 HIF 信号可在特定情境下继续支持炎症转录。成功消退则伴随巨噬细胞向更偏氧化、修复和凋亡细胞清除的状态转换，并出现脂氧素、消退素等促消退脂质介质。

因此，炎症持续往往不是单一通路异常，而是多个“刹车”同时失效：趋化信号不降、中性粒细胞寿命延长、NETs 积聚、凋亡细胞清除不足以及代谢环境持续促炎。相反，当微生物负荷下降、趋化梯度消失、凋亡中性粒细胞被清除并完成脂质介质类别转换时，炎症才真正进入消退。

Q10

问题

在过敏性气道炎症中，肥大细胞和嗜酸性粒细胞（在相关情境下包括嗜碱性粒细胞）如何与树突状细胞和巨噬细胞相互作用，从而放大或限制 2 型免疫反应？

回答

过敏性气道炎症由肥大细胞、嗜酸性粒细胞、嗜碱性粒细胞、树突状细胞和巨噬细胞之间的双向信号共同维持。变应原损伤或刺激上皮后，TSLP、IL-33、IL-25 等警报素先激活树突状细胞和 ILC2；树突状细胞进一步促进 Th2 分化和记忆形成，建立以 IL-4、IL-5、IL-13 为核心的 2 型细胞因子环境。

肥大细胞在 IgE 通过 FcεRI 被交联后迅速释放组胺、胰蛋白酶样蛋白酶、PGD2 和半胱氨酰白三烯，引起支气管收缩、血管渗漏和粒细胞募集；随后释放的 TNF、IL-4、IL-5 和 IL-13 可继续加强树突状细胞活化和嗜酸性粒细胞存活。嗜碱性粒细胞在部分患者或特定阶段提供额外的 IL-4 和脂质介质。嗜酸性粒细胞进入组织后通过颗粒蛋白、白三烯和细胞因子损伤上皮，从而再次诱发警报素释放。

IL-4/IL-13 也会重塑巨噬细胞功能，使其更多参与吞噬、修复、趋化因子释放和基质重塑。短期内这有助于组织恢复；长期则可能促进黏液分泌、纤维化和气道重塑。巨噬细胞通过清除凋亡嗜酸性粒细胞、产生 IL-10 和促消退介质，又能成为网络的“刹车”。

最终是否放大取决于刺激是否持续。如果 IgE 信号、上皮损伤和 IL-5/IL-13 持续存在，而凋亡细胞清除和调节性信号不足，炎症就进入慢性正反馈。反之，随着上皮危险信号下降、粒细胞凋亡并被清除，树突状细胞和巨噬细胞的激活也会逐步回落，2 型炎症才有机会真正消退。

Q11

问题

在急性细菌性尿路感染期间，募集到局部的中性粒细胞和单核细胞来源巨噬细胞如何在病原体清除、细胞因子产生、凋亡细胞清除和黏膜修复过程中协同或竞争？

回答

急性细菌性尿路感染中，中性粒细胞通常承担最早、最强的抗菌任务。尿路上皮和驻留巨噬细胞识别尿路致病菌后产生 CXCL8、IL-1、TNF 等介质，中性粒细胞迅速进入膀胱黏膜及尿腔，通过吞噬、氧爆发、颗粒抗菌蛋白以及部分情况下的 NETs 降低细菌负荷。这个过程效率高，但若持续时间过长，也容易造成尿路上皮损伤。

单核细胞来源巨噬细胞通常较晚到达，却在感染由“攻击期”转向“清理期”时非常关键。它们可继续吞噬细菌、产生趋化因子并清除坏死组织，与中性粒细胞形成协同；所谓“竞争”更多体现为两类细胞可能占用相同炎症资源并倾向不同的细胞因子程序。若两者持续高水平产生 TNF/IL-1，会共同放大组织损伤；若巨噬细胞开始产生 IL-10 并进行凋亡细胞清除，则炎症会逐渐下降。

凋亡细胞清除是这一转换的核心。中性粒细胞完成任务后进入凋亡，被巨噬细胞吞噬后，巨噬细胞会转向修复和屏障重建相关程序，支持受损尿路上皮再生。与此同时，病原体生物学会影响这一过程：尿路致病性大肠杆菌等可形成胞内储存库或保护性微环境，如果过早“踩刹车”，可能允许细菌持续存在。

因此，最优反应不是始终保持最大炎症，而是具有清晰时序：中性粒细胞先快速压低细菌负荷，巨噬细胞随后补充清除和细胞因子调节，再通过凋亡细胞清除和上皮修复结束炎症。感染是否仅限于膀胱、是否上行至肾脏以及既往感染史都会改变两类细胞的相对作用。

Q12

问题

在慢性膀胱黏膜炎症中，肥大细胞与巨噬细胞之间的串扰可能如何改变感觉神经信号、血管通透性、上皮屏障功能以及炎症的持续或消退？

回答

慢性膀胱黏膜炎症中，肥大细胞和巨噬细胞可把免疫炎症与感觉神经异常直接连接起来。肥大细胞释放的组胺、胰蛋白酶样蛋白酶、前列腺素和白三烯可增加血管通透性，并直接敏化膀胱传入神经；蛋白酶还可通过 PAR 相关通路改变神经和上皮反应。神经生长因子等神经营养信号被增强后，感觉阈值进一步下降。

巨噬细胞的作用具有双向性。偏炎症状态的巨噬细胞产生 IL-1β、TNF、前列腺素、ROS 和趋化因子，会继续降低神经兴奋阈值、激活血管内皮并募集更多白细胞；相反，进行凋亡细胞清除或偏调节状态的巨噬细胞产生 IL-10、TGF-β和促消退介质，可帮助终止这一回路。感觉神经释放 P 物质、CGRP 等神经肽后，又可能重新刺激肥大细胞和血管细胞，形成“神经—肥大细胞—巨噬细胞”闭环。

上皮屏障是症状是否持续的另一个关键。组胺、蛋白酶和促炎细胞因子可破坏紧密连接，使尿液中的离子和小分子刺激物更容易接触黏膜下神经和免疫细胞。长期炎症还可引起成纤维细胞活化、基质重塑和神经支配改变，使症状在原始诱因减弱后仍然存在。

真正的炎症消退通常需要同时恢复多个层面：降低肥大细胞活化、让巨噬细胞转向清除和修复、重建上皮屏障，并切断神经肽反馈。人类慢性膀胱疼痛/炎症综合征高度异质，因此这一机制具有较强生物学合理性，但并不能解释所有患者。

Q13

问题

在溃疡性结肠炎中，中性粒细胞募集、中性粒细胞胞外诱捕网形成、中性粒细胞代谢以及中性粒细胞-巨噬细胞串扰如何分别促进黏膜损伤或炎症消退？

回答

在溃疡性结肠炎中，中性粒细胞既是必要的抗菌效应细胞，也是黏膜损伤的重要来源。CXCL8、IL-1、TNF、补体和脂质趋化物推动中性粒细胞大量进入结肠隐窝，形成典型的隐窝脓肿。它们通过吞噬和氧爆发限制细菌穿透，但如果反应持续，弹性蛋白酶、髓过氧化物酶、ROS 和其他颗粒蛋白外泄，就会破坏上皮细胞和紧密连接。

NETs 会进一步放大这种损伤。NET 中的 DNA、组蛋白和颗粒酶能够固定病原体，却也具有直接细胞毒性和促炎性，可激活巨噬细胞及其他先天免疫细胞，并在部分情况下增加微血栓风险。需要注意的是，NET 形成并非绝对有害，其作用取决于细菌负荷、NET 总量及清除速度。

中性粒细胞主要依赖糖酵解，因此在低氧的炎症性结肠黏膜中仍能维持迁移和杀菌。糖酵解及磷酸戊糖途径还为 NADPH 氧化酶提供底物，但持续的低氧、乳酸和炎症代谢环境可延长活化状态。巨噬细胞如果长期暴露于中性粒细胞碎片和微生物产物，也更容易停留在 IL-1/TNF 高表达状态，而不能顺利转向修复。

炎症消退依赖凋亡中性粒细胞被巨噬细胞及时清除。凋亡细胞清除可增加 IL-10、TGF-β和促消退脂质介质，帮助上皮再生并恢复屏障。临床上的治疗难点因此十分明确：完全抑制中性粒细胞会削弱抗菌防御，而不控制持续募集、NETs 和蛋白酶外泄又会加重黏膜损伤。理想目标是减少"病理性持续"，而不是简单去除中性粒细胞。

Q14

问题

在克罗恩病中，中性粒细胞、单核细胞来源巨噬细胞和树突状细胞之间失调的相互作用如何改变微生物处理、抗原呈递、炎症持续以及上皮修复？

回答

克罗恩病中的关键问题并不是某一种髓系细胞单独"过强"或"过弱"，而是微生物处理、炎症放大和组织修复的时序失调。中性粒细胞本应在屏障破裂后快速控制越界微生物，但不同患者和疾病阶段可能出现募集不足、定位错误或随后持续过度聚集。有些机制模型强调早期先天清除效率不足，而已经形成的活动性病灶又常见大量中性粒细胞，这两点在时间维度上可以同时成立。

单核细胞来源巨噬细胞是慢性炎症的重要维持者。与健康肠道中对共生菌相对耐受的驻留巨噬细胞不同，新募集单核细胞在活动期可大量产生 TNF、IL-1β、IL-6 和 IL-23。NOD2、ATG16L1 相关的微生物识别和自噬生物学异常，会影响细菌处理、细胞内应激和炎症终止。未被充分清除的微生物或其产物可持续刺激巨噬细胞和基质细胞。

树突状细胞则把先天炎症延伸为适应性免疫持续。它们呈递微生物抗原并产生支持 Th1/Th17 反应的细胞因子，其中 IL-23 是髓系细胞连接 T 细胞炎症的重要节点。随后 T 细胞又通过细胞因子反向维持巨噬细胞活化和粒细胞募集，形成闭合的炎症回路。

高水平 TNF、IL-1、ROS、蛋白酶和基质重塑酶会延迟上皮再生；而长期修复信号又可能促进纤维狭窄。由于巨噬细胞同时承担细胞碎片清除、血管生成和组织重建，全面髓系抑制并不可取。临床上更合理的理解是：克罗恩病存在明显异质性，核心病理是早期微生物处理异常、炎症单核/巨噬细胞持续化、树突状细胞支持的适应性炎症以及不完整的上皮修复共同叠加。

Q15

问题

在重度中性粒细胞性哮喘中，中性粒细胞、巨噬细胞和树突状细胞如何形成自我维持的炎症回路？中性粒细胞胞外诱捕网、炎症小体信号、微生物信号以及糖皮质激素抵抗可能分别发挥什么作用？

回答

重度中性粒细胞性哮喘可由上皮、巨噬细胞、树突状细胞和中性粒细胞形成自我维持的正反馈。上皮细胞和巨噬细胞受到微生物、污染物或细胞损伤刺激后产生 IL-1β、IL-6、TNF、GM-CSF 和 CXCL8；树突状细胞可支持偏 Th17 的适应性反应，IL-17 又进一步促使上皮和基质细胞释放中性粒细胞趋化因子。中性粒细胞随后释放蛋白酶、ROS 和危险信号，再次造成上皮损伤。

NETs 是放大该回路的重要介质。NET 中的胞外 DNA、组蛋白、弹性蛋白酶和髓过氧化物酶可增加黏液黏稠度、损伤上皮，并刺激巨噬细胞和上皮的模式识别及炎症小体通路。NLRP3–IL-1β轴在部分患者中具有较强机制学合理性，但并非所有中性粒细胞性哮喘都由同一炎症小体机制驱动。

微生物信号同样重要。慢性定植、气道微生态改变或反复感染会持续提供 TLR 信号；反过来，结构性气道病变和黏液纤毛清除下降又增加细菌定植机会。糖皮质激素抵抗则可能与氧化应激、p38 MAPK、糖皮质激素受体功能异常以及 Th17 炎症相对不敏感有关；此外，糖皮质激素可延迟中性粒细胞凋亡，使细胞数量并不一定随其他炎症成分同步下降。

因此，真正导致持续化的通常是多因素叠加：NET 负荷高、巨噬细胞凋亡细胞清除差、IL-1/CXCL8 信号未关闭、微生物刺激仍在。临床评估时应把感染、支气管扩张样改变、激素暴露及非 2 型炎症作为不同患者中权重不同的组成部分，而不是把"中性粒细胞高"视为单一疾病。

Q16

问题

在嗜酸性粒细胞性哮喘中，嗜酸性粒细胞、肥大细胞以及在相关情境下的嗜碱性粒细胞，如何与巨噬细胞、树突状细胞和气道上皮相互作用，从而启动、放大并最终促使 2 型炎症消退？

回答

嗜酸性粒细胞性哮喘从气道上皮—免疫界面启动。变应原、病毒或上皮应激促使 TSLP、IL-33 和 IL-25 释放，进而调节树突状细胞并激活 ILC2。树突状细胞推动 Th2 分化和记忆形成，Th2 与 ILC2 共同产生 IL-4、IL-5 和 IL-13。IL-5 促进嗜酸性粒细胞成熟和存活，eotaxin 把其募集到气道，IL-4/IL-13 则促进 IgE、黏液化生和更广泛的 2 型组织反应。

肥大细胞通过 IgE/FcεRI 进一步放大反应。变应原交联后释放组胺、胰蛋白酶样蛋白酶、PGD2 和半胱氨酰白三烯，引起支气管收缩、血管渗漏和快速白细胞募集。嗜碱性粒细胞在相关患者中可额外提供 IL-4 和脂质介质。进入组织的嗜酸性粒细胞释放主要碱性蛋白、嗜酸性粒细胞过氧化物酶、白三烯和多种细胞因子，进一步损伤上皮并维持警报素释放。

巨噬细胞不是旁观者。IL-4/IL-13 会改变其吞噬、趋化因子产生和基质重塑功能。短期可支持创伤修复，长期则可能促进黏液、纤维化和气道重塑。巨噬细胞清除凋亡嗜酸性粒细胞，是结束炎症的重要环节；随着上皮警报素和抗原暴露下降，树突状细胞活化也会减弱。

因此，炎症消退需要多个节点同时关闭：上皮警报素下降、IL-5 依赖的嗜酸性粒细胞存活终止、IgE 驱动的肥大细胞活化减少，并完成高效凋亡细胞清除。临床上，2 型生物制剂的疗效验证了该网络的多个环节，但若已经存在固定重塑、感染或非 2 型炎症，单纯降低嗜酸性粒细胞仍可能留下症状。

Q17

问题

在复发性尿路感染中，既往感染如何通过训练性免疫、免疫耐受或组织重塑改变膀胱黏膜中的中性粒细胞和单核细胞/巨噬细胞反应？目前对这些机制各自的证据强度如何？

回答

既往尿路感染确实可能给膀胱黏膜留下“记忆”，但训练性免疫、耐受和组织重塑三类机制的证据强度并不相同。相对最明确的是组织重塑：感染可造成尿路上皮脱落、再生性增殖、基质信号变化以及局部神经支配改变；部分尿路致病菌还可能形成胞内储存库。这些结构性和微生物学变化，即使在症状消退后，也能改变下一次感染时中性粒细胞募集和巨噬细胞激活的阈值。

训练性免疫在机制上很有吸引力，但在人类复发性尿路感染中的直接证据仍有限。反复模式识别受体刺激可以在骨髓祖细胞和单核细胞中形成较持久的代谢及表观遗传改变，例如增强糖酵解准备度、改变炎症基因相关染色质可及性。由此产生的新一代单核细胞/巨噬细胞理论上可在再次感染时更快反应。成熟中性粒细胞寿命较短，因此更持久的“训练”更可能发生于祖细胞层面或由长期组织信号维持，而不是来自单个中性粒细胞自身的长期记忆。

耐受则代表相反方向。反复 TLR 刺激可降低后续 TNF、IL-1 等输出，同时保留部分抗菌效应。这样可能减少膀胱损伤，却也可能使早期危险信号不足，增加细菌持续或复发的机会。人群中究竟有多少患者存在真正的“髓系耐受”，目前仍缺乏直接纵向证据。

因此，更现实的临床模型是多种机制并存。组织重塑及胞内细菌持续的证据相对较强；既往感染导致先天免疫反应阈值改变的证据中等且多来自动物或实验系统；针对人类膀胱中单核细胞/巨噬细胞或中性粒细胞存在稳定训练性状态的证据仍属早期。要把训练性免疫或耐受作为确定的复发机制，还需要纵向、细胞特异的功能和表观遗传研究。

Q18

问题

在膀胱癌中，肿瘤相关中性粒细胞和肿瘤相关巨噬细胞如何在黏膜肿瘤微环境中相互作用，并影响抗原呈递、T 细胞活性、血管生成、肿瘤侵袭以及免疫治疗反应？

回答

膀胱癌中的肿瘤相关巨噬细胞（TAM）和肿瘤相关中性粒细胞（TAN）可组成相互强化的髓系抑制网络。肿瘤细胞和基质细胞分泌 CCL2、CSF1、CXCL8、G-CSF 等因子，持续募集单核细胞和中性粒细胞；进入肿瘤后，缺氧、TGF-β、IL-10、前列腺素及代谢应激会把这些细胞推向更偏免疫抑制和组织重塑的状态。

TAM 可降低有效共刺激并表达 PD-L1，同时释放 IL-10、TGF-β、精氨酸代谢相关抑制物和 ROS，从而限制 T 细胞增殖和效应功能。TAN 则可通过 ROS、蛋白酶、精氨酸代谢以及部分情况下的 NETs 进一步抑制 T 细胞。两类细胞还可通过趋化因子和生长因子相互支持其募集和存活，因此即使肿瘤抗原存在，细胞毒性 T 细胞也可能难以进入或保持活性。

这套网络同时促进血管生成和侵袭。VEGF、MMP、弹性蛋白酶等可改变血管和细胞外基质；实验研究还提示 NETs 可能促进肿瘤细胞黏附和播散。需要避免把 TAM 简单分成“M1/M2”、TAN 分成“N1/N2”，因为真实膀胱癌中的髓系状态呈连续谱，并受分期、缺氧和既往治疗影响。

对免疫治疗而言，高度抑制性的 TAM/TAN 环境可能造成检查点抑制剂反应不佳；相反，局部先天免疫激活可促进抗肿瘤反应。膀胱灌注 BCG 就是具有临床意义的例子：它可引起中性粒细胞和巨噬细胞快速募集并建立炎性微环境，从而帮助抗肿瘤免疫，但过强炎症也会带来毒性。

因此，治疗目标更适合“选择性重编程”而非全面清除髓系细胞。若过度抑制 TAM/TAN，也可能削弱尿路抗菌和修复功能。

Q19

问题

在溃疡性结肠炎中，哪些具有机制学合理性的策略可能抑制致病性中性粒细胞募集或中性粒细胞胞外诱捕网相关损伤，同时尽量保留抗微生物防御和巨噬细胞介导的修复？请说明预期获益、潜在权衡及证据缺口。

回答

溃疡性结肠炎中，更合理的目标是减少中性粒细胞的“病理性持续”和胞外毒性效应，而不是把中性粒细胞整体清除。第一类策略是部分抑制 CXCL8–CXCR1/2 等募集轴，从而减少隐窝内持续聚集、蛋白酶和 ROS 外泄。主要风险是系统性阻断会削弱细菌控制，因此局部给药、可逆抑制以及根据中性粒细胞高炎症表型选择患者更符合机制逻辑。

第二类策略针对 NET 相关损伤。PAD4 抑制可减少部分 NET 形成程序；外源 DNase 可加速胞外 DNA 清除；中和胞外组蛋白、弹性蛋白酶或髓过氧化物酶，则有机会保留吞噬功能而降低组织毒性。但这些手段都有权衡：NETs 在某些情况下确实参与胞外抗菌，DNase 切断 DNA 支架后还可能释放仍有活性的蛋白酶；此外，并非所有 NET 形成都依赖 PAD4。

第三类策略不是“抑制”，而是增强炎症消退。Annexin A1、脂氧素/消退素等促消退通路，以及增强 MerTK 相关凋亡细胞清除，都可缩短中性粒细胞在组织内的停留时间，同时让巨噬细胞增加 IL-10 和修复信号。加强上皮屏障、减少微生物移位也能从上游降低中性粒细胞募集需求。

预期获益是保留细胞内杀菌和巨噬细胞修复，同时减少 NET、蛋白酶和氧化应激造成的黏膜损伤。主要风险则是感染、微生物清除延迟以及伤口清创不足。最大的证据缺口在于：多数 NET 或促消退策略仍主要处于临床前阶段，人类黏膜药效学指标缺乏标准化，而且 UC 本身高度异质。最理想的转化路线应当是局部、短效、可监测并按内型选择。

Q20

问题

如何利用肠道巨噬细胞和粒细胞的代谢或表观遗传重编程来恢复炎症性肠病中的黏膜稳态？哪些机制可能导致疗效丧失或非预期的免疫抑制？

回答

通过代谢或表观遗传重编程恢复炎症性肠病黏膜稳态，其核心思路不是消灭髓系细胞，而是改变其功能状态。炎症性巨噬细胞常伴随糖酵解增强、HIF 和 mTOR 活化、琥珀酸/衣康酸代谢改变以及 NAD+/氧化还原失衡。理论上，可通过增强 AMPK 相关氧化代谢、改善线粒体功能或调整免疫代谢物，降低 IL-1β、TNF 等输出，同时尽量保留吞噬、凋亡细胞清除和组织修复能力。这里应避免把过程简单描述成“M1 转 M2”，因为真实组织中的巨噬细胞状态更连续、更复杂。

表观遗传层面，持续炎症的单核细胞可具有增强子可及性、组蛋白乙酰化/甲基化及“训练性免疫样”染色质改变。因此，BET、HDAC、组蛋白甲基化或 DNA 甲基化相关通路都具有潜在靶向价值。如果能做到局部或细胞选择性调节，理论上可压低过度的炎症转录，同时恢复更偏耐受、吞噬和修复的程序。

粒细胞的风险更突出。中性粒细胞高度依赖糖酵解和磷酸戊糖途径来支持迁移、氧爆发和 NET 形成；过强代谢抑制可能减少黏膜损伤，却同时直接削弱抗菌。更可取的是限制过度 NETosis、延长寿命或胞外蛋白酶，而保留面对真实感染时的吞噬和杀菌。

疗效丧失可来自代谢可塑性、旁路细胞因子、持续微生物移位以及新募集单核细胞不断补充。非预期免疫抑制则来自同一通路在抗感染、伤口愈合和疫苗应答中的正常作用。系统性表观遗传药物还会影响上皮、淋巴细胞和造血祖细胞。因而真正可转化的方案需要黏膜定向、短疗程和可测量的细胞状态生物标志物；目前总体仍属机制上有吸引力、临床上尚未成熟的方向。

Q21

问题

能够改变 2 型细胞因子信号的生物制剂，除降低嗜酸性粒细胞数量外，还可能如何重塑气道黏膜中的嗜酸性粒细胞-肥大细胞-巨噬细胞-树突状细胞网络？

回答

改变 2 型细胞因子信号的生物制剂会重塑整个气道髓系网络，而不仅仅是“把嗜酸性粒细胞数目降下来”。阻断 IL-5 或 IL-5R 会减少嗜酸性粒细胞成熟、存活和组织驻留，同时也减少其释放的白三烯、颗粒蛋白和细胞因子，因此肥大细胞、巨噬细胞、树突状细胞和上皮受到的二级刺激都会下降。

阻断 IL-4Rα同时影响 IL-4 和 IL-13 信号，可降低上皮 eotaxin、黏液化生和 IgE 相关环境，也会直接改变原本被 IL-4/IL-13 塑造的巨噬细胞转录程序。这样可减少与黏液、纤维化和重塑相关的巨噬细胞功能。树突状细胞和 T 细胞之间的交流也会因上皮细胞因子环境和抗原暴露改变而间接受到影响。

针对更上游的 TSLP 等警报素，则可更早切断“上皮—树突状细胞—Th2/ILC2”的链条，因而同时降低嗜酸性粒细胞、肥大细胞和嗜碱性粒细胞的后续激活。IL-33 相关通路理论上也有类似网络效应。抗 IgE 治疗主要降低肥大细胞和嗜碱性粒细胞的 FcεRI 驱动活化，但其二级结果同样是让树突状细胞和巨噬细胞面对的炎症环境变得更弱。

这些变化还会改善炎症消退：需要巨噬细胞清除的活化嗜酸性粒细胞更少，持续上皮警报素也会下降。不过，生物制剂不能完全逆转固定气道重塑、感染、非 2 型炎症或结构性疾病。临床上更准确的理解是“网络重新平衡”——减少粒细胞存活和脱颗粒、降低上皮警报素放大、重塑巨噬细胞的修复/纤维化程序，并削弱树突状细胞对反复 2 型免疫记忆的支持。

Q22

问题

在中性粒细胞性哮喘中，哪些针对髓系细胞的策略可能中断中性粒细胞-巨噬细胞炎症回路，同时保留抗细菌防御？这些策略所依据的机制中，哪些已有临床证据，哪些仍主要处于临床前阶段？

回答

中性粒细胞性哮喘的治疗难点，是切断 CXCL8/IL-1—中性粒细胞—巨噬细胞炎症回路，同时不能把肺部抗菌能力一起关闭。就目前的临床证据而言，还没有一种已经成为常规标准、且高度髓系特异的治疗能够稳定做到这一点。大环内酯类在部分重症哮喘患者中具有较成熟的临床应用基础，其作用可能包括降低急性加重并调节中性粒细胞、巨噬细胞及上皮细胞因子，但它并不是选择性髓系药物，长期使用还涉及耐药和微生态风险。

CXCR2 拮抗剂在机制上很直接，因为可减少中性粒细胞进入气道。临床试验中确实可看到痰液或外周中性粒细胞下降，但症状和急性加重获益并不稳定，同时存在感染风险。因此，它属于“已有临床验证但尚未成为常规标准”的机制，而不是已确立治疗。广泛阻断 IL-1、IL-17 等通路也未形成普遍有效的中性粒细胞性哮喘方案。

更多策略目前主要处于临床前或早期转化阶段：NLRP3 抑制可针对炎症小体高表型患者；PAD4 抑制、DNase 清除 NET、胞外组蛋白中和或中性粒细胞蛋白酶抑制可减少 NET 相关上皮损伤；增强巨噬细胞凋亡细胞清除和促消退脂质通路则更像是“恢复正常终止程序”，而非阻断所有中性粒细胞募集。

保留抗菌能力要求局部、可逆、按内型治疗。存在支气管扩张、慢性细菌定植或反复肺部感染的患者尤其不适合强系统性中性粒细胞抑制。临床上真正成熟的部分仍有限：大环内酯可作为特定患者的辅助策略，而大多数专门髓系靶向机制仍属研究方向。

Q23

问题

训练性免疫或黏膜疫苗策略是否可能通过有益地重编程单核细胞/巨噬细胞和中性粒细胞来减少复发性尿路感染？请解释其拟议机制、潜在的组织特异性风险以及当前证据的局限。

回答

训练性免疫和黏膜疫苗理论上都可能降低复发性尿路感染，但更理想的目标不是让膀胱长期处于高炎症状态，而是让再次暴露病原体时的应答更快、更高效。黏膜疫苗可通过产生病原体特异性抗体、组织驻留淋巴细胞和更快速的局部免疫启动，提高调理吞噬效率，使中性粒细胞和巨噬细胞更快清除细菌，从而缩短需要高强度炎症的时间。

训练性免疫针对的是先天免疫。模式识别受体刺激可在骨髓祖细胞和单核细胞中形成代谢及表观遗传重编程，使再次感染时的吞噬、趋化和抗菌效应更迅速。理想状态是增强细菌清除而不伴随过度 IL-1/TNF 或 NET 释放。由于成熟中性粒细胞寿命短，长期效应更可能来自祖细胞重编程或组织微环境改变，而不是单个中性粒细胞保留"记忆"。

风险主要来自组织特异性。长期被"预激活"的膀胱黏膜可能出现过量中性粒细胞募集、上皮损伤、疼痛、尿急甚至纤维化；反复先天刺激还可能改变微生态，或者诱导耐受而不是训练，导致真正感染时反应反而不足。若训练效应是全身性的，还可能加重其他部位炎症。

目前证据具有可行性但不充分。动物和基础研究证明先天免疫状态可被较持久地重塑，也有多种疫苗或细菌裂解物方案用于复发性尿路感染的临床探索，但制剂、终点和研究质量差异较大。尤其缺乏直接证据证明人类获益确实来自单核细胞/巨噬细胞或中性粒细胞的训练性重编程。因此，现阶段更适合把它视为有前景的机制和研究方向，而不是已经确立的预防机制。

Q24

问题

如何通过选择性重编程肿瘤相关巨噬细胞和中性粒细胞来增强膀胱癌的抗肿瘤免疫，同时尽量减少对保护性尿路黏膜免疫的破坏？请比较不同机制策略及其主要转化障碍。

回答

膀胱癌中的髓系重编程应当把肿瘤相关巨噬细胞和中性粒细胞从免疫抑制、促血管生成和组织重塑状态，转换为短时、局部的抗肿瘤效应状态，同时尽量保留尿路抗菌功能。膀胱具有一个重要优势：可通过膀胱灌注实现局部给药，从而减少系统性髓系抑制。

针对巨噬细胞，可考虑抑制 CSF1/CSF1R 或 CCL2 募集轴、抑制 PI3Kγ样免疫抑制信号、激活 TLR/STING 等先天通路，以及阻断 CD47–SIRPα"别吃我"信号以增强肿瘤细胞吞噬。理想结果是提高肿瘤抗原摄取和炎性抗原呈递，同时减少 IL-10、TGF-β和 VEGF 主导的抑制环境。相比全面清除巨噬细胞，功能性重编程更符合尿路需要，因为巨噬细胞还承担抗感染和修复。

针对中性粒细胞，CXCR2 轴阻断可减少抑制性 TAN 募集；PAD4/NET、弹性蛋白酶或 TGF-β相关干预可降低促侵袭和免疫抑制作用。但中性粒细胞是尿路抗细菌防御的第一线，因此持续系统性抑制风险很高，更适合采用局部或间歇性方案。

这些手段可与免疫检查点抑制剂协同，通过改善 T 细胞进入和减少髓系抑制来增强疗效。膀胱灌注 BCG 已经证明"局部激活先天免疫"能够产生临床抗肿瘤效应，虽然它并不是选择性的 TAM/TAN 重编程，而且炎症毒性明显。

主要转化障碍包括髓系细胞高度可塑、缺乏稳定的人体 TAM/TAN 标志物、肿瘤异质性、补偿性募集通路、药物穿透以及感染风险。最现实的路径是局部、可逆、联合治疗，并用空间和功能生物标志物区分"肿瘤抑制性髓系细胞"与"保护性尿路髓系细胞"。

Traditional Chinese (TC)

Anonymous Model D | 繁體中文

Anonymous Model D

繁體中文

24 standardized questions

Q01

問題

在健康腸黏膜中，中性粒細胞如何在與駐留巨噬細胞和樹突狀細胞相互作用的同時參與屏障防禦？哪些機制通常限制中性粒細胞介導的組織損傷？

回答

健康腸黏膜中的中性粒細胞並不是長期佔優勢的常駐細胞，而是一個"按需快速動員"的效應群體。上皮屏障出現微小破損、細菌產物越過黏液層或局部模式識別受體被活化後，上皮細胞、基質細胞和駐留巨噬細胞可產生 CXCL1/CXCL2 以及人類中更典型的 CXCL8，並聯合補體和脂質趨化訊號募集粒細胞。到達局部後，中性粒細胞透過吞噬、NADPH 氧化酶依賴的氧爆發、抗菌顆粒釋放及在特定情況下形成 NETs，對進入組織的細菌和真菌進行快速控制。關鍵並不在於"發炎強度越大越好"，而在於效應盡量局限於屏障破損處。

駐留巨噬細胞承擔重要的調節作用。健康腸道巨噬細胞能夠持續吞噬微生物和細胞碎片，卻對共生菌刺激相對低反應，並透過 IL-10 等訊號限制不必要的發炎放大。樹突狀細胞則負責抗原採樣，並在真正存在感染風險時決定是否需要啟動適應性免疫。中性粒細胞釋放的蛋白酶、脂質介質和危險訊號也會反過來影響巨噬細胞和樹突狀細胞，但在穩態下這種串擾通常是短暫而可終止的。

限制組織損傷依賴主動的發炎消退程序。完成殺菌後的中性粒細胞進入凋亡，由巨噬細胞透過 MerTK、AXL 等相關通路進行凋亡細胞清除。這個過程會抑制 TNF、IL-1 等促炎輸出，並促進 IL-10、TGF-β及組織修復程序。脂氧素、消退素等促消退脂質介質、腺苷、Annexin A1、黏液層和上皮抗蛋白酶共同減少彈性蛋白酶、髓過氧化物酶、ROS 和 NET 成分的附帶損傷。

因此，健康狀態下的核心是"快速募集—局部殺菌—及時清除—屏障修復"。如果募集持續、NETs 堆積、中性粒細胞發生繼發性壞死或巨噬細胞凋亡細胞清除受損，同一套防禦機制就會轉化為黏膜損傷機制，這也是發炎性腸病中中性粒細胞從保護性向致病性轉變的重要生物學基礎。

Q02
問題
在健康腸黏膜中，嗜酸性粒細胞如何參與維持穩態並與巨噬細胞或樹突狀細胞進行通訊？這些作用與發炎狀態下嗜酸性粒細胞的功能有何不同？

回答
腸道嗜酸性粒細胞的特點是：即使沒有明顯發炎，固有層中也可存在相當數量的細胞。其維持與 IL-5、CCL11 等 eotaxin 家族趨化因子有關。穩態時，它們更像是黏膜免疫和組織生態中的調節者，參與上皮/基質穩態、細胞外基質週轉、免疫監視以及局部體液免疫；動物研究還提示其可支持漿細胞和 IgA 相關反應，但這些作用在人類不同腸段中的重要性並不完全一致。

嗜酸性粒細胞與巨噬細胞、樹突狀細胞存在雙向通訊。嗜酸性粒細胞來源的 IL-4、IL-13 可推動部分巨噬細胞進入更偏向吞噬、修復和基質重塑的功能狀態；TGF-β、脂質介質和生長因子也可影響局部修復。反過來，巨噬細胞和基質來源的趨化因子、存活訊號會調節嗜酸性粒細胞的募集和壽命。嗜酸性粒細胞的顆粒蛋白及脂質介質還可能改變樹突狀細胞的遷移、成熟和抗原呈遞。臨床上應避免把這些變化簡單等同於“M1/M2”二分法，因為腸道巨噬細胞實際上呈連續譜狀態。

發炎時，功能重點明顯改變。IL-5、GM-CSF 和 CCL11/24/26 升高會延長嗜酸性粒細胞存活並增強脫顆粒。主要鹼性蛋白、嗜酸性粒細胞過氧化物酶、陽離子蛋白、白三烯和 ROS 可直接損傷上皮、增加血管通透性並維持局部發炎，也可能促進成纖維細胞活化和慢性重塑。

因此，判斷嗜酸性粒細胞是保護還是致病，不能只看數量，而要看活化程度、顆粒釋放方式、組織位置及持續時間。穩態下它們更多嵌入耐受和修復網路；持續的 2 型細胞激素暴露和脫顆粒則可把同一網路推向慢性發炎和組織損傷。

Q03
問題
在健康呼吸道黏膜以及急性微生物攻擊期間，中性粒細胞如何與肺泡或間質巨噬細胞及樹突狀細胞協同，在發揮宿主防禦作用的同時限制對上皮的附帶損傷？

回答
健康肺內，肺泡巨噬細胞是氣液界面的主要髓系“哨兵”，其重要任務之一是對無害顆粒和低強度微生物刺激保持克制，從而避免不必要的肺泡發炎。急性細菌或真菌刺激出現後，肺泡/間質巨噬細胞及氣道上皮透過 IL-1、TNF、CXCL1/2/5 以及人類 CXCL8 等訊號迅速募集中性粒細胞；樹突狀細胞同時攝取抗原，為後續 T 細胞應答提供連接。

中性粒細胞透過吞噬、氧爆發、顆粒蛋白和抗菌肽進行高效殺菌，遇到難以吞噬的病原體時可形成 NETs。巨噬細胞則補充吞噬、細胞激素調度、肺泡碎片和表面活性物質清除，並決定是否繼續募集單核細胞。樹突狀細胞根據危險訊號強度調整成熟和抗原呈遞。這個階段三類細胞形成協同防禦，但其理想狀態應當是短暫、空間受限。

限制上皮附帶損傷主要依靠“終止訊號”。中性粒細胞完成任務後進入凋亡，由巨噬細胞進行凋亡細胞清除，從而降低 IL-1/TNF 輸出並促進 IL-10、TGF-β以及脂氧素、消退素等促消退通路。肺泡表面活性物質、抗氧化體系和抗蛋白酶可中和外洩的 ROS 和彈性蛋白酶；NET DNA 和組蛋白也需要及時清除，隨後恢復緊密連接和黏液纖毛清除功能。

從臨床病理角度看，真正決定結局的是是否能從“殺菌期”順利切換到“消退期”。適度、短暫的中性粒細胞流入對於肺部抗菌是必要的；持續趨化、NETs 積聚、壞死性細胞死亡或巨噬細胞凋亡細胞清除失敗，則會形成蛋白酶—氧化應激—上皮損傷的正回饋，見於重症肺炎、急性肺損傷和部分慢性中性粒細胞性氣道疾病。

Q04
問題
在穩態條件和第 2 型發炎期間，嗜酸性粒細胞如何在氣道黏膜中與巨噬細胞和樹突狀細胞相互作用？

回答
穩態氣道中嗜酸性粒細胞數量較低，局部環境主要由肺泡巨噬細胞和樹突狀細胞維持低發炎狀態。巨噬細胞負責清除顆粒及凋亡細胞，樹突狀細胞雖然持續進行抗原採樣，但在缺乏危險訊號時並不會強烈推動 T 細胞反應。人類健康氣道中嗜酸性粒細胞的具體穩態功能尚不如其在 2 型發炎中的作用明確。

發生 2 型發炎時，氣道上皮受到變應原、病毒或機械損傷後釋放 TSLP、IL-33 和 IL-25。這些上皮警報素一方面活化樹突狀細胞，另一方面刺激 ILC2，繼而形成 IL-4、IL-5、IL-13 為主的環境。IL-5 延長嗜酸性粒細胞存活，eotaxin 促進其進入氣道。活化嗜酸性粒細胞釋放主要鹼性蛋白、嗜酸性粒細胞過氧化物酶、半胱氨酰白三烯及多種細胞激素，可損傷上皮、影響平滑肌和血管反應，並繼續加強抗原呈遞細胞的活化。

樹突狀細胞決定了反應是否形成持續的適應性記憶；IL-4/IL-13 則把巨噬細胞推向偏修復和重塑的功能譜。急性損傷後這種狀態可以幫助清除細胞殘骸、重建組織，但長期存在時會參與黏液高分泌、纖維化和氣道重塑。嗜酸性粒細胞產生的 IL-4/IL-13 及脂質介質可進一步鞏固這種巨噬細胞狀態。

發炎消退需要上皮警報素下降、IL-5/eotaxin 訊號減弱、嗜酸性粒細胞進入凋亡並被巨噬細胞清除，同時由 IL-10 和促消退脂質介質抑制剩餘發炎。也就是說，同一套嗜酸性粒細胞—巨噬細胞—樹突狀細胞網路，在短暫刺激後可服務於修復，而在持續上皮活化和 2 型細胞激素驅動下則成為慢性氣喘的維持機制。

Q05

問題

在尿路黏膜中，早期抗菌防禦期間，中性粒細胞如何與單核細胞或巨噬細胞及樹突狀細胞協同？哪些機制可防止過度組織損傷？

回答

尿路早期抗菌反應主要從尿路上皮和駐留髓系細胞對細菌產物的識別開始。膀胱上皮 TLR 相關訊號及駐留巨噬細胞可迅速產生 IL-6、TNF 和 CXCL8 等中性粒細胞趨化因子，補體片段和脂質介質進一步增強募集。中性粒細胞進入黏膜和尿腔後，透過吞噬、氧爆發、抗菌肽/顆粒蛋白釋放以及部分情境下的 NET 形成，迅速降低尿路致病菌負荷。

駐留巨噬細胞和後續募集的單核細胞承擔兩階段作用：早期透過細胞激素和趨化因子支持中性粒細胞募集，並直接吞噬細菌；後期則清除壞死細胞和凋亡中性粒細胞，使局部環境從"殺菌模式"轉向"修復模式"。樹突狀細胞攝取微生物抗原，並在感染持續或復發時促進適應性免疫。中性粒細胞殺菌也會改變抗原量和危險訊號強度，從而間接影響樹突狀細胞功能。

防止過度損傷的關鍵，是限制中性粒細胞活化的持續時間。尿流和上皮脫落可物理性清除細菌及被感染細胞，減少長期高強度發炎的必要性。中性粒細胞凋亡後被巨噬細胞進行凋亡細胞清除，可增加 IL-10、TGF-β和促消退脂質介質，並促進上皮再生。局部抗氧化體系和抗蛋白酶也能限制 ROS、彈性蛋白酶和 NET 成分對尿路上皮的附帶損傷。

臨床上的核心權衡是：募集不足會增加上行感染風險，而募集過度則造成黏膜脫落、水腫、出血和疼痛。理想反應應按時間順序完成——先以中性粒細胞快速控制細菌，再由巨噬細胞清除發炎殘骸並啟動上皮修復。

Q06

問題

在膀胱或下泌尿生殖道黏膜中，肥大細胞釋放的介質如何影響巨噬細胞和樹突狀細胞的功能？這一網路如何同時參與宿主防禦和發炎相關症狀？

回答

肥大細胞位於膀胱和下泌尿生殖道的血管、感覺神經和上皮附近，因此很容易成為連接"感染防禦"和"症狀產生"的樞紐。受到 IgE/FcεRI、微生物模式識別或神經肽刺激後，肥大細胞可釋放組胺、胰蛋白酶樣蛋白酶、前列腺素、白三烯、TNF、IL-6 及多種趨化因子。這些介質增加血管通透性和白細胞進入，同時可增強巨噬細胞發炎反應，並促進樹突狀細胞成熟、遷移和共刺激能力。

在感染背景下，這種放大是有價值的。血管通透性增加有助於血漿抗菌成分和循環白細胞進入局部；肥大細胞來源的 TNF 和趨化因子可加快中性粒細胞、單核細胞募集；巨噬細胞負責吞噬病原體和壞死組織，樹突狀細胞則將局部感染訊號連接到特異性免疫。

但同樣的網路也可產生發炎症狀。組胺和脂質介質可直接敏化感覺神經；肥大細胞蛋白酶透過 PAR 等受體改變神經和上皮反應。巨噬細胞產生的 IL-1、TNF、前列腺素和 ROS 進一步降低感覺閾值。感覺神經釋放 P 物質、CGRP 等神經肽後，又可能反過來活化肥大細胞，形成神經—免疫正回饋。與此同時，蛋白酶和發炎因子削弱上皮緊密連接，使尿液中的刺激性溶質更容易接觸黏膜下神經末梢。

能否消退取決於 IL-10 型/凋亡細胞清除型巨噬細胞、肥大細胞抑制性受體、介質降解和上皮屏障修復是否及時恢復。慢性膀胱疼痛綜合徵在人群中高度異質，因此肥大細胞—巨噬細胞串擾應理解為重要的機制亞型之一，而不是所有患者共同的單一病因。

Q07

問題

在急性腸黏膜發炎及其後的發炎消退階段，中性粒細胞與巨噬細胞如何透過募集訊號、細胞激素、凋亡細胞清除、脂質介質和組織修復程序相互影響？

回答

急性腸黏膜發炎首先進入募集和殺菌階段。受損上皮、駐留巨噬細胞和基質細胞釋放 CXCL 趨化因子、IL-1、TNF、補體訊號及脂質趨化物，中性粒細胞沿梯度進入黏膜。它們透過吞噬、ROS、顆粒蛋白和部分情況下的 NETs 控制越過屏障的微生物；同時，中性粒細胞自身釋放 LTB4 等介質，又可繼續放大白細胞募集，因此早期反應具有明顯的正回饋特徵。

巨噬細胞既參與早期抗菌，也負責把發炎"剎車"。中性粒細胞完成任務後逐漸凋亡並暴露磷脂醯絲氨酸等"eat-me"訊號，巨噬細胞透過 MerTK 等受體進行凋亡細胞清除。這個過程會重塑巨噬細胞的轉錄和脂質代謝狀態，使 TNF/IL-1 輸出下降，而 IL-10、TGF-β、修復因子和組織重建程序上升。因此，凋亡細胞清除並不是被動清垃圾，而是發炎由進攻期切換到消退期的關鍵開關。

脂質介質也發生"類別轉換"：早期前列腺素、白三烯偏向募集和血管反應，後期脂氧素、消退素、保護素和 maresin 類介質則減少進一步中性粒細胞進入、促進非炎性吞噬並幫助上皮修復。中性粒細胞自身也可提供 Annexin A1 等促消退訊號。

若中性粒細胞在被清除前發生繼發壞死或大量 NET 形成，DNA、組蛋白、彈性蛋白酶和髓過氧化物酶會變成持續的危險訊號；若巨噬細胞凋亡細胞清除能力不足，促發炎細胞激素也難以下降。臨床上，這種"先協同抗菌、後協同消退"的雙相關係，決定了急性發炎能否恢復正常黏膜結構。

Q08

問題

嗜酸性粒細胞來源的介質如何改變腸黏膜巨噬細胞的功能狀態和樹突狀細胞的活化？這些相互作用如何推動免疫耐受、組織修復與發炎之間的平衡發生變化？

回答

嗜酸性粒細胞來源的介質可以把腸道巨噬細胞和樹突狀細胞推向修復、耐受或發炎中的不同方向。IL-4 和 IL-13 是較明確的例子：在一定範圍內可促進巨噬細胞的凋亡細胞清除、組織修復和基質重塑程序。嗜酸性粒細胞還可產生 TGF-β、脂質介質及生長因子，從而影響上皮和基質細胞。需要強調的是，腸道巨噬細胞的狀態遠比傳統"M1/M2"二分複雜，其最終表型同時受微生物群、上皮訊號和新募集單核細胞影響。

樹突狀細胞可受到嗜酸性粒細胞顆粒蛋白和脂質介質的調節。部分實驗系統中，嗜酸性粒細胞來源神經毒素等陽離子蛋白可表現為危險訊號，增強樹突狀細胞成熟或抗原呈遞；半胱氨酰白三烯等則可改變樹突狀細胞遷移及細胞激素輸出。另一方面，在低發炎環境中，嗜酸性粒細胞也可能參與支持 IgA 和局部免疫穩態的網路，但這些穩態作用在人類中的證據沒有發炎效應那麼一致。

決定方向的核心是活化強度和持續時間。適度 IL-4/IL-13 訊號、伴隨有效的細胞碎片清除，可促進屏障修復；如果 IL-5/GM-CSF 持續延長嗜酸性粒細胞壽命並出現明顯脫顆粒，則主要鹼性蛋白、過氧化物酶和 ROS 會造成上皮損傷，形成新的危險訊號，繼而讓巨噬細胞和樹突狀細胞繼續維持發炎。

因此，嗜酸性粒細胞本身既不是固定“耐受性”也不是固定“致炎性”。其作用取決於哪些介質佔優勢、是否發生廣泛脫顆粒以及巨噬細胞/樹突狀細胞能否在修復完成後及時退出活化狀態。

Q09

問題

在中性粒細胞性氣道發炎中，中性粒細胞與巨噬細胞如何透過細胞激素、脂質介質、中性粒細胞胞外誘捕網、凋亡細胞清除和代謝訊號相互影響？這種串擾如何決定發炎持續還是消退？

回答

在中性粒細胞性氣道發炎中，中性粒細胞和巨噬細胞既可以形成短暫有效的抗菌協作，也可以形成自我維持的發炎迴路。氣道上皮和活化巨噬細胞釋放 IL-1β、TNF、GM-CSF 和 CXCL8 等訊號，持續募集並延長中性粒細胞壽命；中性粒細胞則透過彈性蛋白酶、ROS、LTB4 和危險相關分子反過來刺激巨噬細胞和上皮。當微生物訊號長期存在時，TLR 和發炎小體通路會進一步固定這一正回饋。

NETs 是決定結局的重要分叉點。NET 中的 DNA、組蛋白、彈性蛋白酶和髓過氧化物酶可捕獲病原體，但同時會損傷上皮、增加痰液黏稠度，並活化巨噬細胞的危險識別和 IL-1 通路。相反，如果中性粒細胞以凋亡方式結束其效應期並被巨噬細胞及時清除，巨噬細胞會減少促發炎細胞激素輸出，並增加 IL-10、TGF-β以及促消退相關程序。

代謝環境也會影響這種串擾。低氧、發炎性氣道往往促使中性粒細胞和發炎型巨噬細胞更多依賴糖酵解；乳酸、琥珀酸、氧化還原狀態和 HIF 訊號可在特定情境下繼續支持發炎轉錄。成功消退則伴隨巨噬細胞向更偏氧化、修復和凋亡細胞清除的狀態轉換，並出現脂氧素、消退素等促消退脂質介質。

因此，發炎持續往往不是單一通路異常，而是多個“剎車”同時失效：趨化訊號不降、中性粒細胞壽命延長、NETs 積聚、凋亡細胞清除不足以及代謝環境持續促炎。相反，當微生物負荷下降、趨化梯度消失、凋亡中性粒細胞被清除並完成脂質介質類別轉換時，發炎才真正進入消退。

Q10

問題

在過敏性氣道發炎中，肥大細胞和嗜酸性粒細胞（在相關情境下包括嗜鹼性粒細胞）如何與樹突狀細胞和巨噬細胞相互作用，從而放大或限制第 2 型免疫反應？

回答

過敏性氣道發炎由肥大細胞、嗜酸性粒細胞、嗜鹼性粒細胞、樹突狀細胞和巨噬細胞之間的雙向訊號共同維持。變應原損傷或刺激上皮後，TSLP、IL-33、IL-25 等警報素先活化樹突狀細胞和 ILC2；樹突狀細胞進一步促進 Th2 分化和記憶形成，建立以 IL-4、IL-5、IL-13 為核心的 2 型細胞激素環境。

肥大細胞在 IgE 透過 FcεRI 被交聯後迅速釋放組胺、胰蛋白酶樣蛋白酶、PGD2 和半胱氨酰白三烯，引起支氣管收縮、血管滲漏和粒細胞募集；隨後釋放的 TNF、IL-4、IL-5 和 IL-13 可繼續加強樹突狀細胞活化和嗜酸性粒細胞存活。嗜鹼性粒細胞在部分患者或特定階段提供額外的 IL-4 和脂質介質。嗜酸性粒細胞進入組織後透過顆粒蛋白、白三烯和細胞激素損傷上皮，從而再次誘發警報素釋放。

IL-4/IL-13 也會重塑巨噬細胞功能，使其更多參與吞噬、修復、趨化因子釋放和基質重塑。短期內這有助於組織恢復；長期則可能促進黏液分泌、纖維化和氣道重塑。巨噬細胞透過清除凋亡嗜酸性粒細胞、產生 IL-10 和促消退介質，又能成為網路的“剎車”。

最終是否放大取決於刺激是否持續。如果 IgE 訊號、上皮損傷和 IL-5/IL-13 持續存在，而凋亡細胞清除和調節性訊號不足，發炎就進入慢性正回饋。反之，隨著上皮危險訊號下降、粒細胞凋亡並被清除，樹突狀細胞和巨噬細胞的活化也會逐步回落，2 型發炎才有機會真正消退。

Q11

問題

在急性細菌性尿路感染期間，募集到局部的中性粒細胞和單核細胞來源巨噬細胞如何在病原體清除、細胞激素產生、凋亡細胞清除和黏膜修復過程中協同或競爭？

回答

急性細菌性尿路感染中，中性粒細胞通常承擔最早、最強的抗菌任務。尿路上皮和駐留巨噬細胞識別尿路致病菌後產生 CXCL8、IL-1、TNF 等介質，中性粒細胞迅速進入膀胱黏膜及尿腔，透過吞噬、氧爆發、顆粒抗菌蛋白以及部分情況下的 NETs 降低細菌負荷。這個過程效率高，但若持續時間過長，也容易造成尿路上皮損傷。

單核細胞來源巨噬細胞通常較晚到達，卻在感染由“攻擊期”轉向“清理期”時非常關鍵。它們可繼續吞噬細菌、產生趨化因子並清除壞死組織，與中性粒細胞形成協同；所謂“競爭”更多體現為兩類細胞可能佔用相同發炎資源並傾向不同的細胞激素程序。若兩者持續高強度產生 TNF/IL-1，會共同放大組織損傷；若巨噬細胞開始產生 IL-10 並進行凋亡細胞清除，則發炎會逐漸下降。

凋亡細胞清除是這一轉換的核心。中性粒細胞完成任務後進入凋亡，被巨噬細胞吞噬後，巨噬細胞會轉向修復和屏障重建相關程序，支持受損尿路上皮再生。與此同時，病原體生物學會影響這一過程：尿路致病性大腸桿菌等可形成胞內儲存庫或保護性微環境，如果過早“踩剎車”，可能允許細菌持續存在。

因此，最優反應不是始終保持最大發炎，而是具有清晰時序：中性粒細胞先快速壓低細菌負荷，巨噬細胞隨後補充清除和細胞激素調節，再透過凋亡細胞清除和上皮修復結束發炎。感染是否僅限於膀胱、是否上行至腎臟以及既往感染史都會改變兩類細胞的相對作用。

Q12

問題

在慢性膀胱黏膜發炎中，肥大細胞與巨噬細胞之間的串擾可能如何改變感覺神經訊號、血管通透性、上皮屏障功能以及發炎的持續或消退？

回答

慢性膀胱黏膜發炎中，肥大細胞和巨噬細胞可把免疫發炎與感覺神經異常直接連接起來。肥大細胞釋放的組胺、胰蛋白酶樣蛋白酶、前列腺素和白三烯可增加血管通透性，並直接敏化膀胱傳入神經；蛋白酶還可透過 PAR 相關通路改變神經和上皮反應。神經生長因子等神經營養訊號被增強後，感覺閾值進一步下降。

巨噬細胞的作用具有雙向性。偏發炎狀態的巨噬細胞產生 IL-1β、TNF、前列腺素、ROS 和趨化因子，會繼續降低神經興奮閾值、活化血管內皮並募集更多白細胞；相反，進行凋亡細胞清除或偏調節狀態的巨噬細胞產生 IL-10、TGF-β和促消退介質，可幫助終止這一迴路。感覺神經釋放 P 物質、CGRP 等神經肽後，又可能重新刺激肥大細胞和血管細胞，形成“神經—肥大細胞—巨噬細胞”閉環。

上皮屏障是症狀是否持續的另一個關鍵。組胺、蛋白酶和促發炎細胞激素可破壞緊密連接，使尿液中的離子和小分子刺激物更容易接觸黏膜下神經和免疫細胞。長期發炎還可引起成纖維細胞活化、基質重塑和神經支配改變，使症狀在原始誘因減弱後仍然存在。

真正的發炎消退通常需要同時恢復多個層面：降低肥大細胞活化、讓巨噬細胞轉向清除和修復、重建上皮屏障，並切斷神經肽回饋。人類慢性膀胱疼痛/發炎綜合徵高度異質，因此這一機制具有較強生物學合理性，但並不能解釋所有患者。

Q13

問題

在潰瘍性結腸炎中，中性粒細胞募集、中性粒細胞胞外誘捕網形成、中性粒細胞代謝以及中性粒細胞-巨噬細胞串擾如何分別促進黏膜損傷或發炎消退？

回答

在潰瘍性結腸炎中，中性粒細胞既是必要的抗菌效應細胞，也是黏膜損傷的重要來源。CXCL8、IL-1、TNF、補體和脂質趨化物推動中性粒細胞大量進入結腸隱窩，形成典型的隱窩膿腫。它們透過吞噬和氧爆發限制細菌穿透，但如果反應持續，彈性蛋白酶、髓過氧化物酶、ROS 和其他顆粒蛋白外洩，就會破壞上皮細胞和緊密連接。

NETs 會進一步放大這種損傷。NET 中的 DNA、組蛋白和顆粒酶能夠固定病原體，卻也具有直接細胞毒性和促炎性，可活化巨噬細胞及其他先天免疫細胞，並在部分情況下增加微血栓風險。需要注意的是，NET 形成並非絕對有害，其作用取決於細菌負荷、NET 總量及清除速度。

中性粒細胞主要依賴糖酵解，因此在低氧的發炎性結腸黏膜中仍能維持遷移和殺菌。糖酵解及磷酸戊糖途徑還為 NADPH 氧化酶提供底物，但持續的低氧、乳酸和發炎代謝環境可延長活化狀態。巨噬細胞如果長期暴露於中性粒細胞碎片和微生物產物，也更容易停留在 IL-1/TNF 高表現狀態，而不能順利轉向修復。

發炎消退依賴凋亡中性粒細胞被巨噬細胞及時清除。凋亡細胞清除可增加 IL-10、TGF-β和促消退脂質介質，幫助上皮再生並恢復屏障。臨床上的治療難點因此十分明確：完全抑制中性粒細胞會削弱抗菌防禦，而不控制持續募集、NETs 和蛋白酶外洩又會加重黏膜損傷。理想目標是減少“病理性持續”，而不是簡單去除中性粒細胞。

Q14

問題

在克隆氏症中，中性粒細胞、單核細胞來源巨噬細胞和樹突狀細胞之間失調的相互作用如何改變微生物處理、抗原呈遞、發炎持續以及上皮修復？

回答

克隆氏症中的關鍵問題並不是某一種髓系細胞單獨“過強”或“過弱”，而是微生物處理、發炎放大和組織修復的時序失調。中性粒細胞本應在屏障破裂後快速控制越界微生物，但不同患者和疾病階段可能出現募集不足、定位錯誤或隨後持續過度聚集。有些機制模型強調早期先天清除效率不足，而已經形成的活動性病灶又常見大量中性粒細胞，這兩點在時間維度上可以同時成立。

單核細胞來源巨噬細胞是慢性發炎的重要維持者。與健康腸道中對共生菌相對耐受的駐留巨噬細胞不同，新募集單核細胞在活動期可大量產生 TNF、IL-1β、IL-6 和 IL-23。NOD2、ATG16L1 相關的微生物識別和自噬生物學異常，會影響細菌處理、細胞內應激和發炎終止。未被充分清除的微生物或其產物可持續刺激巨噬細胞和基質細胞。

樹突狀細胞則把先天發炎延伸為適應性免疫持續。它們呈遞微生物抗原並產生支持 Th1/Th17 反應的細胞激素，其中 IL-23 是髓系細胞連接 T 細胞發炎的重要節點。隨後 T 細胞又透過細胞激素反向維持巨噬細胞活化和粒細胞募集，形成閉合的發炎迴路。

高強度 TNF、IL-1、ROS、蛋白酶和基質重塑酶會延遲上皮再生；而長期修復訊號又可能促進纖維狹窄。由於巨噬細胞同時承擔細胞碎片清除、血管新生和組織重建，全面髓系抑制並不可取。臨床上更合理的理解是：克隆氏症存在明顯異質性，核心病理是早期微生物處理異常、發炎單核/巨噬細胞持續化、樹突狀細胞支持的適應性發炎以及不完整的上皮修復共同疊加。

Q15

問題

在重度中性粒細胞性氣喘中，中性粒細胞、巨噬細胞和樹突狀細胞如何形成自我維持的發炎迴路？中性粒細胞胞外誘捕網、發炎小體訊號、微生物訊號以及糖皮質激素抗性可能分別發揮什麼作用？

回答

重度中性粒細胞性氣喘可由上皮、巨噬細胞、樹突狀細胞和中性粒細胞形成自我維持的正回饋。上皮細胞和巨噬細胞受到微生物、污染物或細胞損傷刺激後產生 IL-1β、IL-6、TNF、GM-CSF 和 CXCL8；樹突狀細胞可支持偏 Th17 的適應性反應，IL-17 又進一步促使上皮和基質細胞釋放中性粒細胞趨化因子。中性粒細胞隨後釋放蛋白酶、ROS 和危險訊號，再次造成上皮損傷。

NETs 是放大該迴路的重要介質。NET 中的胞外 DNA、組蛋白、彈性蛋白酶和髓過氧化物酶可增加黏液黏稠度、損傷上皮，並刺激巨噬細胞和上皮的模式識別及發炎小體通路。NLRP3–IL-1β軸在部分患者中具有較強機制學合理性，但並非所有中性粒細胞性氣喘都由同一發炎小體機制驅動。

微生物訊號同樣重要。慢性定植、氣道微生態改變或反復感染會持續提供 TLR 訊號；反過來，結構性氣道病變和黏液纖毛清除下降又增加細菌定植機會。糖皮質激素抵抗則可能與氧化應激、p38 MAPK、糖皮質激素受體功能異常以及 Th17 發炎相對不敏感有關；此外，糖皮質激素可延遲中性粒細胞凋亡，使細胞數量並不一定隨其他發炎成分同步下降。

因此，真正導致持續化的通常是多因素疊加：NET 負荷高、巨噬細胞凋亡細胞清除差、IL-1/CXCL8 訊號未關閉、微生物刺激仍在。臨床評估時應把感染、支氣管擴張樣改變、激素暴露及非 2 型發炎作為不同患者中權重不同的組成部分，而不是把“中性粒細胞高”視為單一疾病。

Q16

問題

在嗜酸性粒細胞性氣喘中，嗜酸性粒細胞、肥大細胞以及在相關情境下的嗜鹼性粒細胞，如何與巨噬細胞、樹突狀細胞和氣道上皮相互作用，從而啟動、放大並最終促使第 2 型發炎消退？

回答

嗜酸性粒細胞性氣喘從氣道上皮—免疫界面啟動。變應原、病毒或上皮應激促使 TSLP、IL-33 和 IL-25 釋放，進而調節樹突狀細胞並活化 ILC2。樹突狀細胞推動 Th2 分化和記憶形成，Th2 與 ILC2 共同產生 IL-4、IL-5 和 IL-13。IL-5 促進嗜酸性粒細胞成熟和存活，eotaxin 把其募集到氣道，IL-4/IL-13 則促進 IgE、黏液化生和更廣泛的 2 型組織反應。

肥大細胞透過 IgE/FcεRI 進一步放大反應。變應原交聯後釋放組胺、胰蛋白酶樣蛋白酶、PGD2 和半胱氨酰白三烯，引起支氣管收縮、血管滲漏和快速白細胞募集。嗜鹼性粒細胞在相關患者中可額外提供 IL-4 和脂質介質。進入組織的嗜酸性粒細胞釋放主要鹼性蛋白、嗜酸性粒細胞過氧化物酶、白三烯和多種細胞激素，進一步損傷上皮並維持警報素釋放。

巨噬細胞不是旁觀者。IL-4/IL-13 會改變其吞噬、趨化因子產生和基質重塑功能。短期可支持創傷修復，長期則可能促進黏液、纖維化和氣道重塑。巨噬細胞清除凋亡嗜酸性粒細胞，是結束發炎的重要環節；隨著上皮警報素和抗原暴露下降，樹突狀細胞活化也會減弱。

因此，發炎消退需要多個節點同時關閉：上皮警報素下降、IL-5 依賴的嗜酸性粒細胞存活終止、IgE 驅動的肥大細胞活化減少，並完成高效凋亡細胞清除。臨床上，2 型生物製劑的療效驗證了該網路的多個環節，但若已經存在固定重塑、感染或非 2 型發炎，單純降低嗜酸性粒細胞仍可能留下症狀。

Q17

問題

在復發性尿路感染中，既往感染如何透過訓練性免疫、免疫耐受或組織重塑改變膀胱黏膜中的中性粒細胞和單核細胞/巨噬細胞反應？目前對這些機制各自的證據強度如何？

回答

既往尿路感染確實可能給膀胱黏膜留下“記憶”，但訓練性免疫、耐受和組織重塑三類機制的證據強度並不相同。相對最明確的是組織重塑：感染可造成尿路上皮脫落、再生性增殖、基質訊號變化以及局部神經支配改變；部分尿路致病菌還可能形成胞內儲存庫。這些結構性和微生物學變化，即使在症狀消退後，也能改變下一次感染時中性粒細胞募集和巨噬細胞活化的閾值。

訓練性免疫在機制上很有吸引力，但在人類復發性尿路感染中的直接證據仍有限。反復模式識別受體刺激可以在骨髓祖細胞和單核細胞中形成較持久的代謝及表觀遺傳改變，例如增強糖酵解準備度、改變發炎基因相關染色質可及性。由此產生的新一代單核細胞/巨噬細胞理論上可在再次感染時更快反應。成熟中性粒細胞壽命較短，因此更持久的“訓練”更可能發生於祖細胞層面或由長期組織訊號維持，而不是來自單個中性粒細胞自身的長期記憶。

耐受則代表相反方向。反復 TLR 刺激可降低後續 TNF、IL-1 等輸出，同時保留部分抗菌效應。這樣可能減少膀胱損傷，卻也可能使早期危險訊號不足，增加細菌持續或復發的機會。人群中究竟有多少患者存在真正的“髓系耐受”，目前仍缺乏直接縱向證據。

因此，更現實的臨床模型是多種機制並存。組織重塑及胞內細菌持續的證據相對較強；既往感染導致先天免疫反應閾值改變的證據中等且多來自動物或實驗系統；針對人類膀胱中單核細胞/巨噬細胞或中性粒細胞存在穩定訓練性狀態的證據仍屬早期。要把訓練性免疫或耐受作為確定的復發機制，還需要縱向、細胞特異的功能和表觀遺傳研究。

Q18

問題

在膀胱癌中，腫瘤相關中性粒細胞和腫瘤相關巨噬細胞如何在黏膜腫瘤微環境中相互作用，並影響抗原呈遞、T 細胞活性、血管新生、腫瘤侵襲以及免疫治療反應？

回答

膀胱癌中的腫瘤相關巨噬細胞（TAM）和腫瘤相關中性粒細胞（TAN）可組成相互強化的髓系抑制網路。腫瘤細胞和基質細胞分泌 CCL2、CSF1、CXCL8、G-CSF 等因子，持續募集單核細胞和中性粒細胞；進入腫瘤後，缺氧、TGF-β、IL-10、前列腺素及代謝應激會把這些細胞推向更偏免疫抑制和組織重塑的狀態。

TAM 可降低有效共刺激並表現 PD-L1，同時釋放 IL-10、TGF-β、精氨酸代謝相關抑制物和 ROS，從而限制 T 細胞增殖和效應功能。TAN 則可透過 ROS、蛋白酶、精氨酸代謝以及部分情況下的 NETs 進一步抑制 T 細胞。兩類細胞還可透過趨化因子和生長因子相互支持其募集和存活，因此即使腫瘤抗原存在，細胞毒性 T 細胞也可能難以進入或保持活性。

這套網路同時促進血管新生和侵襲。VEGF、MMP、彈性蛋白酶等可改變血管和細胞外基質；實驗研究還提示 NETs 可能促進腫瘤細胞黏附和播散。需要避免把 TAM 簡單分成“M1/M2”、TAN 分成“N1/N2”，因為真實膀胱癌中的髓系狀態呈連續譜，並受分期、缺氧和既往治療影響。

對免疫治療而言，高度抑制性的 TAM/TAN 環境可能造成檢查點抑製劑反應不佳；相反，局部先天免疫活化可促進抗腫瘤反應。膀胱灌注 BCG 就是具有臨床意義的例子：它可引起中性粒細胞和巨噬細胞快速募集並建立炎性微環境，從而幫助抗腫瘤免疫，但過強發炎也會帶來毒性。

因此，治療目標更適合“選擇性重編程”而非全面清除髓系細胞。若過度抑制 TAM/TAN，也可能削弱尿路抗菌和修復功能。

Q19

問題

在潰瘍性結腸炎中，哪些具有機制學合理性的策略可能抑制致病性中性粒細胞募集或中性粒細胞胞外誘捕網相關損傷，同時盡量保留抗微生物防禦和巨噬細胞介導的修復？請說明預期獲益、潛在權衡及證據缺口。

回答

潰瘍性結腸炎中，更合理的目標是減少中性粒細胞的“病理性持續”和胞外毒性效應，而不是把中性粒細胞整體清除。第一類策略是部分抑制 CXCL8–CXCR1/2 等募集軸，從而減少隱窩內持續聚集、蛋白酶和 ROS 外洩。主要風險是系統性阻斷會削弱細菌控制，因此局部給藥、可逆抑制以及根據中性粒細胞高發炎表型選擇患者更符合機制邏輯。

第二類策略針對 NET 相關損傷。PAD4 抑制可減少部分 NET 形成程序；外源 DNase 可加速胞外 DNA 清除；中和胞外組蛋白、彈性蛋白酶或髓過氧化物酶，則有機會保留吞噬功能而降低組織毒性。但這些手段都有權衡：NETs 在某些情況下確實參與胞外抗菌，DNase 切斷 DNA 支架後還可能釋放仍有活性的蛋白酶；此外，並非所有 NET 形成都依賴 PAD4。

第三類策略不是“抑制”，而是增強發炎消退。Annexin A1、脂氧素/消退素等促消退通路，以及增強 MerTK 相關凋亡細胞清除，都可縮短中性粒細胞在組織內的停留時間，同時讓巨噬細胞增加 IL-10 和修復訊號。加強上皮屏障、減少微生物移位也能從上游降低中性粒細胞募集需求。

預期獲益是保留細胞內殺菌和巨噬細胞修復，同時減少 NET、蛋白酶和氧化應激造成的黏膜損傷。主要風險則是感染、微生物清除延遲以及傷口清創不足。最大的證據缺口在於：多數 NET 或促消退策略仍主要處於臨床前階段，人類黏膜藥效學指標缺乏標準化，而且 UC 本身高度異質。最理想的轉化路線應當是局部、短效、可監測並按內型選擇。

Q20

問題

如何利用腸道巨噬細胞和粒細胞的代謝或表觀遺傳重編程來恢復發炎性腸病中的黏膜穩態？哪些機制可能導致療效喪失或非預期的免疫抑制？

回答

透過代謝或表觀遺傳重編程恢復發炎性腸病黏膜穩態，其核心思路不是消滅髓系細胞，而是改變其功能狀態。發炎性巨噬細胞常伴隨糖酵解增強、HIF 和 mTOR 活化、琥珀酸/衣康酸代謝改變以及 NAD+/氧化還原失衡。理論上，可透過增強 AMPK 相關氧化代謝、改善線粒體功能或調整免疫代謝物，降低 IL-1β、TNF 等輸出，同時盡量保留吞噬、凋亡細胞清除和組織修復能力。這裡應避免把過程簡單描述成“M1 轉 M2”，因為真實組織中的巨噬細胞狀態更連續、更複雜。

表觀遺傳層面，持續發炎的單核細胞可具有增強子可及性、組蛋白乙酰化/甲基化及“訓練性免疫樣”染色質改變。因此，BET、HDAC、組蛋白甲基化或 DNA 甲基化相關通路都具有潛在靶向價值。如果能做到局部或細胞選擇性調節，理論上可壓低過度的發炎轉錄，同時恢復更偏耐受、吞噬和修復的程序。

粒細胞的風險更突出。中性粒細胞高度依賴糖酵解和磷酸戊糖途徑來支持遷移、氧爆發和 NET 形成；過強代謝抑制可能減少黏膜損傷，卻同時直接削弱抗菌。更可取的是限制過度 NETosis、延長壽命或胞外蛋白酶，而保留面對真實感染時的吞噬和殺菌。

療效喪失可來自代謝可塑性、旁路細胞激素、持續微生物移位以及新募集單核細胞不斷補充。非預期免疫抑制則來自同一通路在抗感染、傷口愈合和疫苗應答中的正常作用。系統性表觀遺傳藥物還會影響上皮、淋巴細胞和造血祖細胞。因而真正可轉化的方案需要黏膜定向、短療程和可測量的細胞狀態生物標誌物；目前總體仍屬機制上有吸引力、臨床上尚未成熟的方向。

Q21
問題
能夠改變第 2 型細胞激素訊號的生物製劑，除降低嗜酸性粒細胞數量外，還可能如何重塑氣道黏膜中的嗜酸性粒細胞-肥大細胞-巨噬細胞-樹突狀細胞網路？

回答
改變 2 型細胞激素訊號的生物製劑會重塑整個氣道髓系網路，而不僅僅是“把嗜酸性粒細胞數量降下來”。阻斷 IL-5 或 IL-5R 會減少嗜酸性粒細胞成熟、存活和組織駐留，同時也減少其釋放的白三烯、顆粒蛋白和細胞激素，因此肥大細胞、巨噬細胞、樹突狀細胞和上皮受到的二級刺激都會下降。

阻斷 IL-4Rα同時影響 IL-4 和 IL-13 訊號，可降低上皮 eotaxin、黏液化生和 IgE 相關環境，也會直接改變原本被 IL-4/IL-13 塑造的巨噬細胞轉錄程序。這樣可減少與黏液、纖維化和重塑相關的巨噬細胞功能。樹突狀細胞和 T 細胞之間的交流也會因上皮細胞激素環境和抗原暴露改變而間接受到影響。

針對更上游的 TSLP 等警報素，則可更早切斷“上皮—樹突狀細胞—Th2/ILC2”的鏈條，因而同時降低嗜酸性粒細胞、肥大細胞和嗜鹼性粒細胞的後續活化。IL-33 相關通路理論上也有類似網路效應。抗 IgE 治療主要降低肥大細胞和嗜鹼性粒細胞的 FcεRI 驅動活化，但其二級結果同樣是讓樹突狀細胞和巨噬細胞面對的發炎環境變得更弱。

這些變化還會改善發炎消退：需要巨噬細胞清除的活化嗜酸性粒細胞更少，持續上皮警報素也會下降。不過，生物製劑不能完全逆轉固定氣道重塑、感染、非 2 型發炎或結構性疾病。臨床上更準確的理解是“網路重新平衡”——減少粒細胞存活和脫顆粒、降低上皮警報素放大、重塑巨噬細胞的修復/纖維化程序，並削弱樹突狀細胞對反復 2 型免疫記憶的支持。

Q22
問題
在中性粒細胞性氣喘中，哪些針對髓系細胞的策略可能中斷中性粒細胞-巨噬細胞發炎迴路，同時保留抗細菌防禦？這些策略所依據的機制中，哪些已有臨床證據，哪些仍主要處於臨床前階段？

回答
中性粒細胞性氣喘的治療難點，是切斷 CXCL8/IL-1—中性粒細胞—巨噬細胞發炎迴路，同時不能把肺部抗菌能力一起關閉。就目前的臨床證據而言，還沒有一種已經成為常規標準、且高度髓系特異的治療能夠穩定做到這一點。大環內酯類在部分重症氣喘患者中具有較成熟的臨床應用基礎，其作用可能包括降低急性加重並調節中性粒細胞、巨噬細胞及上皮細胞激素，但它並不是選擇性髓系藥物，長期使用還涉及耐藥和微生態風險。

CXCR2 拮抗劑在機制上很直接，因為可減少中性粒細胞進入氣道。臨床試驗中確實可看到痰液或外周中性粒細胞下降，但症狀和急性加重獲益並不穩定，同時存在感染風險。因此，它屬於“已有臨床驗證但尚未成為常規標準”的機制，而不是已確立治療。廣泛阻斷 IL-1、IL-17 等通路也未形成普遍有效的中性粒細胞性氣喘方案。

更多策略目前主要處於臨床前或早期轉化階段：NLRP3 抑制可針對發炎小體高表型患者；PAD4 抑制、DNase 清除 NET、胞外組蛋白中和或中性粒細胞蛋白酶抑制可減少 NET 相關上皮損傷；增強巨噬細胞凋亡細胞清除和促消退脂質通路則更像是“恢復正常終止程序”，而非阻斷所有中性粒細胞募集。

保留抗菌能力要求局部、可逆、按內型治療。存在支氣管擴張、慢性細菌定植或反復肺部感染的患者尤其不適合強系統性中性粒細胞抑制。臨床上真正成熟的部分仍有限：大環內酯可作為特定患者的輔助策略，而大多數專門髓系靶向機制仍屬研究方向。

Q23
問題
訓練性免疫或黏膜疫苗策略是否可能透過有益地重編程單核細胞/巨噬細胞和中性粒細胞來減少復發性尿路感染？請解釋其擬議機制、潛在的組織特異性風險以及目前證據的侷限。

回答
訓練性免疫和黏膜疫苗理論上都可能降低復發性尿路感染，但更理想的目標不是讓膀胱長期處於高發炎狀態，而是讓再次暴露病原體時的應答更快、更高效。黏膜疫苗可透過產生病原體特異性抗體、組織駐留淋巴細胞和更快速的局部免疫啟動，提高調理吞噬效率，使中性粒細胞和巨噬細胞更快清除細菌，從而縮短需要高強度發炎的時間。

訓練性免疫針對的是先天免疫。模式識別受體刺激可在骨髓祖細胞和單核細胞中形成代謝及表觀遺傳重編程，使再次感染時的吞噬、趨化和抗菌效應更迅速。理想狀態是增強細菌清除而不伴隨過度 IL-1/TNF 或 NET 釋放。由於成熟中性粒細胞壽命短，長期效應更可能來自祖細胞重編程或組織微環境改變，而不是單個中性粒細胞保留“記憶”。

風險主要來自組織特異性。長期被“預活化”的膀胱黏膜可能出現過量中性粒細胞募集、上皮損傷、疼痛、尿急甚至纖維化；反復先天刺激還可能改變微生態，或者誘導耐受而不是訓練，導致真正感染時反應反而不足。若訓練效應是全身性的，還可能加重其他部位發炎。

目前證據具有可行性但不充分。動物和基礎研究證明先天免疫狀態可被較持久地重塑，也有多種疫苗或細菌裂解物方案用於復發性尿路感染的臨床探索，但製劑、終點和研究質量差異較大。尤其缺乏直接證據證明人類獲益確實來自單核細胞/巨噬細胞或中性粒細胞的訓練性重編程。因此，現階段更適合把它視為有前景的機制和研究方向，而不是已經確立的預防機制。

Q24

問題

如何透過選擇性重編程腫瘤相關巨噬細胞和中性粒細胞來增強膀胱癌的抗腫瘤免疫，同時盡量減少對保護性尿路黏膜免疫的破壞？請比較不同機制策略及其主要轉譯障礙。

回答

膀胱癌中的髓系重編程應當把腫瘤相關巨噬細胞和中性粒細胞從免疫抑制、促血管新生和組織重塑狀態，轉換為短時、局部的抗腫瘤效應狀態，同時盡量保留尿路抗菌功能。膀胱具有一個重要優勢：可透過膀胱灌注實現局部給藥，從而減少系統性髓系抑制。

針對巨噬細胞，可考慮抑制 CSF1/CSF1R 或 CCL2 募集軸、抑制 PI3Kγ樣免疫抑制訊號、活化 TLR/STING 等先天通路，以及阻斷 CD47–SIRPα"別吃我"訊號以增強腫瘤細胞吞噬。理想結果是提高腫瘤抗原攝取和炎性抗原呈遞，同時減少 IL-10、TGF-β和 VEGF 主導的抑制環境。相比全面清除巨噬細胞，功能性重編程更符合尿路需要，因為巨噬細胞還承擔抗感染和修復。

針對中性粒細胞，CXCR2 軸阻斷可減少抑制性 TAN 募集；PAD4/NET、彈性蛋白酶或 TGF-β相關干預可降低促侵襲和免疫抑製作用。但中性粒細胞是尿路抗細菌防禦的第一線，因此持續系統性抑制風險很高，更適合採用局部或間歇性方案。

這些手段可與免疫檢查點抑製劑協同，透過改善 T 細胞進入和減少髓系抑制來增強療效。膀胱灌注 BCG 已經證明"局部活化先天免疫"能夠產生臨床抗腫瘤效應，雖然它並不是選擇性的 TAM/TAN 重編程，而且發炎毒性明顯。

主要轉譯障礙包括髓系細胞高度可塑、缺乏穩定的人體 TAM/TAN 標誌物、腫瘤異質性、補償性募集通路、藥物穿透以及感染風險。最現實的路徑是局部、可逆、聯合治療，並用空間和功能生物標誌物區分"腫瘤抑制性髓系細胞"與"保護性尿路髓系細胞"。

V. Model E

English (EN)

Anonymous Model E | English | Informal Response Package

Anonymous Model E

English

Q01

Question

At the healthy intestinal mucosa, how do neutrophils contribute to barrier defense while interacting with resident macrophages and dendritic cells, and what mechanisms normally limit neutrophil-driven tissue injury?

Response

In health, neutrophils are not meant to accumulate continuously in the intestinal lamina propria. They are kept mainly in the circulation and are recruited rapidly when epithelial cells, resident macrophages, or other sentinel cells detect microbial penetration. Chemokines such as CXCL1/CXCL2 and, in humans, CXCL8, together with complement and lipid mediators, guide them to the breached surface. Once there, neutrophils phagocytose bacteria and fungi, generate reactive oxygen species, release antimicrobial granule proteins, and can form extracellular traps when stimulation is strong. Resident macrophages help by sensing microbes, producing recruitment signals, and then taking over clearance of debris. Dendritic cells sample luminal antigens and provide a bridge to adaptive immunity rather than serving as the main acute killing cell.

The same neutrophil weapons can damage epithelium, so several brakes normally operate. The mucus layer, epithelial tight junctions, antimicrobial peptides, and rapid epithelial turnover reduce how often neutrophils need to be recruited. Intestinal macrophages are relatively hyporesponsive to harmless commensal material and produce regulatory mediators such as IL-10. Neutrophils also have a short functional life; after microbial control they undergo apoptosis and are removed by macrophage efferocytosis, which shifts macrophages toward IL-10, TGF-β, and tissue-repair programs. Pro-resolving lipid mediators, local antioxidants, antiproteases, and drainage of inflammatory material further limit collateral injury.

Clinically, the key point is balance: a brief, localized neutrophil response is protective, whereas persistent recruitment, excessive protease/oxidant release, or poorly cleared extracellular traps can convert a defense program into mucosal injury.

Q02

Question

At the healthy intestinal mucosa, how do eosinophils contribute to homeostasis and communicate with macrophages or dendritic cells, and how do these roles differ from eosinophil functions during inflammation?

Response

Eosinophils are normal residents of the intestinal lamina propria, so their presence in the gut is not automatically a sign of disease. Under homeostatic conditions they are maintained by signals including IL-5 and eotaxins acting through CCR3, and they participate in barrier surveillance, local immune regulation, and tissue remodeling. They can support plasma-cell and IgA responses and can release cytokines and growth factors that influence epithelial and stromal cells. Their communication with macrophages and dendritic cells is usually low-grade: eosinophil-derived IL-4/IL-13 and lipid mediators can bias macrophages toward reparative functions, while myeloid-cell cytokines can in turn regulate eosinophil survival and activation. Effects on dendritic-cell maturation or antigen presentation are plausible but are more context dependent than the basic recruitment pathways.

During active inflammation, the same cells become much more injurious. Activated eosinophils release major basic protein, eosinophil cationic protein, eosinophil peroxidase, leukotrienes, cytokines, and reactive oxygen species. These products can increase epithelial permeability, amplify vascular and neural responses, and activate or recruit additional myeloid cells. In type 2-dominant inflammation, eosinophils may reinforce IL-4/IL-13-dependent macrophage programs; in mixed inflammatory states they can coexist with neutrophils and inflammatory monocytes.

Thus, homeostatic eosinophils are best viewed as tissue-adapted regulatory and repair-associated cells with antimicrobial potential, whereas inflammatory eosinophils show stronger degranulation and cytokine output. The boundary is not absolute, and human intestinal disease shows substantial heterogeneity.

Q03

Question

At the healthy respiratory mucosa and during an acute microbial challenge, how do neutrophils coordinate with alveolar or interstitial macrophages and dendritic cells to provide host defense while limiting collateral epithelial injury?

Response

At baseline, alveolar macrophages are the main phagocytic sentinels in the airspaces and normally keep inflammation quiet despite constant exposure to inhaled material. During an acute microbial challenge, pattern-recognition signaling in alveolar or interstitial macrophages and airway epithelium induces IL-1, TNF, CXCL chemokines, G-CSF, and related mediators. These signals recruit neutrophils from the blood. Neutrophils then provide rapid bacterial and fungal killing by phagocytosis, oxidant production, granule enzymes, and, when necessary, extracellular traps. Dendritic cells capture antigen and migrate to lymph nodes to initiate or shape adaptive responses; they also receive inflammatory signals from macrophages and neutrophils.

Protection depends on stopping this cascade once the pathogen burden falls. Neutrophils undergo apoptosis, and macrophage efferocytosis removes them without spilling granule contents. This process suppresses further IL-1/TNF signaling and promotes IL-10, TGF-β, and pro-resolving lipid mediators. Airway antiproteases such as α1-antitrypsin and secretory leukocyte protease inhibitor, extracellular antioxidants, DNases, mucociliary clearance, and epithelial repair programs limit damage from elastase, myeloperoxidase products, and extracellular DNA.

If microbial clearance is incomplete, or if efferocytosis and epithelial repair are defective, the same macrophage-neutrophil circuit can become self-sustaining. Clinically this is relevant in severe pneumonia, COPD exacerbations, and some forms of severe asthma, where persistent neutrophilia can protect against microbes yet also worsen epithelial injury and gas-exchange abnormalities.

Q04

Question

How do eosinophils interact with macrophages and dendritic cells in the airway mucosa under homeostatic conditions and during type 2 inflammation?

Response

In a healthy airway, eosinophils are relatively sparse and their interaction with macrophages and dendritic cells is limited. Resident macrophages mainly maintain an anti-inflammatory environment and clear inhaled particles, while dendritic cells sample antigen without strongly activating type 2 immunity. Small numbers of eosinophils can contribute to tissue surveillance and repair, but there is less evidence for a dominant homeostatic eosinophil-myeloid circuit in the airway than in the gut.

During type 2 inflammation, the network changes markedly. Damaged or allergen-exposed epithelium releases TSLP, IL-33, and IL-25, which activate dendritic cells and type 2 innate lymphoid pathways. Th2/ILC2-derived IL-5 promotes eosinophil production and survival, while IL-4/IL-13 induce eotaxins and mucus programs. Recruited eosinophils release leukotrienes, granule proteins, cytokines, and growth factors. Their IL-4/IL-13 can reinforce alternatively activated or repair-oriented macrophage states, while macrophages can produce chemokines such as CCL17/CCL22 and help shape the local Th2 environment. Dendritic cells amplify the process by presenting allergen and maintaining Th2 polarization.

Macrophages also participate in resolution by clearing apoptotic eosinophils and producing regulatory mediators. Therefore the same network can be protective when it supports controlled repair, but pathogenic when eosinophil degranulation, mucus production, bronchial hyperreactivity, and remodeling become persistent. The exact contribution of eosinophils to dendritic-cell function is context dependent and varies across asthma phenotypes.

Q05

Question

In the urinary tract mucosa, how do neutrophils coordinate with monocytes or macrophages and dendritic cells during early antibacterial defense, and what mechanisms prevent excessive tissue injury?

Response

Early antibacterial defense in the urinary tract begins with urothelial and myeloid recognition of bacterial products, especially during ascending infection. Urothelial cells, macrophages, and dendritic cells can produce IL-1, IL-6, CXCL chemokines and other signals that rapidly recruit neutrophils. Neutrophils are usually the main early effector cells: they migrate across the mucosa, phagocytose bacteria, generate reactive oxygen species, release antimicrobial granule proteins, and can form extracellular traps. Monocytes and macrophages support this response by producing cytokines, ingesting organisms and debris, and later clearing dying neutrophils. Dendritic cells are more important for antigen sampling and coordination with adaptive immunity than for bulk bacterial killing.

Because urine flow and epithelial shedding already help remove bacteria, an overly strong neutrophil response can be counterproductive. Proteases, oxidants, and extracellular-trap components can damage urothelial cells and increase permeability. Tissue injury is normally limited by rapid bacterial clearance, neutrophil apoptosis, macrophage efferocytosis, IL-10/TGF-β-dominant resolution signals, local antioxidant and antiprotease systems, and regeneration of the urothelium.

The clinical trade-off is straightforward: inadequate recruitment allows bacterial persistence or upper-tract spread, whereas prolonged neutrophilia can worsen dysuria, hematuria, and mucosal injury. Macrophages are therefore important not only for early defense but also for switching the bladder from an inflammatory to a repair phase.

Q06

Question

In the bladder or lower urogenital mucosa, how can mast-cell mediators influence macrophage and dendritic-cell functions, and how might this network contribute to both host defense and inflammatory symptoms?

Response

Mast cells in the bladder and lower urogenital mucosa can be activated by microbial products, IgE-dependent signals, tissue damage, complement, and neuropeptides. They release histamine, tryptase, TNF, prostaglandins, leukotrienes, and multiple cytokines. These mediators increase vascular

permeability and local blood flow, recruit leukocytes, and can activate sensory nerves. In an acute infection this may be useful because it accelerates delivery of neutrophils and monocytes to the mucosa.

Macrophages and dendritic cells respond to mast-cell products through cytokine receptors, histamine receptors, protease-activated pathways, and lipid-mediator receptors. TNF and some lipid mediators can promote dendritic-cell maturation and inflammatory macrophage activity, enhancing antigen presentation and antimicrobial responses. Conversely, macrophage cytokines and danger signals can further activate mast cells. Depending on the mediator mix, mast cells can also release regulatory products that limit or reshape inflammation, so the interaction is not uniformly pro-inflammatory.

The same network can produce symptoms. Histamine, tryptase, prostaglandins, leukotrienes, NGF-related signaling, and macrophage-derived IL-1/TNF/PGE2 can sensitize afferent nerves, causing urgency, pain, and frequency, while increased vascular and epithelial permeability exposes the tissue to additional irritants. In chronic conditions this positive feedback may sustain inflammation even when infection is absent. Evidence is strongest for the general neuroimmune and vascular mechanisms; the dominant mast-cell-macrophage pathway likely differs among infection, interstitial cystitis/bladder pain syndrome, and other urogenital inflammatory states.

Q07

Question

During acute intestinal mucosal inflammation and subsequent resolution, how do neutrophils and macrophages influence each other through recruitment signals, cytokines, efferocytosis, lipid mediators, and tissue-repair programs?

Response

Acute intestinal inflammation typically starts with rapid neutrophil recruitment driven by epithelial and myeloid chemokines, complement, and lipid mediators such as LTB4. Neutrophils kill microbes but also release IL-1-family signals, proteases, oxidants, and danger-associated material that can recruit CCR2-dependent monocytes. These monocytes differentiate into inflammatory macrophages that produce TNF, IL-1β and other cytokines, reinforcing neutrophil influx when microbial or tissue danger persists.

Resolution requires a coordinated switch rather than simple disappearance of cells. Neutrophils stop receiving survival signals, undergo apoptosis, and expose phosphatidylserine. Macrophages recognize and engulf them through efferocytosis pathways that include receptors such as MerTK. Efferocytosis suppresses further inflammatory cytokine production and promotes IL-10, TGF-β, growth factors, and repair programs. At the same time, lipid-mediator production can shift from leukotriene-dominant signaling toward lipoxins, resolvins, protectins, and maresins, which reduce further neutrophil recruitment and enhance non-inflammatory clearance.

Repair-oriented macrophages then support epithelial restitution, matrix remodeling, angiogenesis, and restoration of barrier function. This is not a simple M1-to-M2 switch; intestinal macrophage states are more diverse. If neutrophils fail to undergo apoptosis, macrophage efferocytosis is impaired, or microbes continue to stimulate the tissue, the circuit remains inflammatory. Thus neutrophils can initiate both tissue damage and the signals that, once properly cleared, instruct macrophages to resolve inflammation.

Q08

Question

How can eosinophil-derived mediators alter macrophage polarization and dendritic-cell activation in the intestinal mucosa, and how could these interactions shift the balance among tolerance, tissue repair, and inflammation?

Response

Eosinophils can influence intestinal macrophages through cytokines, lipid mediators, granule products, and growth factors. IL-4 and IL-13 released by activated eosinophils can favor macrophage programs associated with tissue repair, extracellular-matrix handling, and control of type 2 responses. TGF-β and other growth factors may support epithelial repair but, if sustained, can also contribute to fibrosis. Eosinophil lipid mediators can alter vascular and myeloid-cell activation, while granule proteins and oxidants can act as local danger signals when degranulation is intense.

Effects on dendritic cells are more variable. Eosinophil-derived cytokines can change dendritic-cell maturation, migration, and the cytokine environment in which antigen is presented. In a relatively quiet mucosa, this may help maintain controlled immunity to dietary and microbial antigens. During strong type 2 or mixed inflammation, the same interaction can favor greater T-cell activation and recruitment of additional granulocytes. Direct antigen-presenting activity by eosinophils has been demonstrated in some settings, but it is not generally considered their main role in human gut mucosa.

The balance therefore depends on activation state and timing. Moderate eosinophil signals can support barrier repair and regulatory macrophage functions; persistent IL-4/IL-13/TGF-β signaling may promote remodeling; and heavy degranulation can directly injure epithelium and activate dendritic cells. It is more accurate to describe macrophage states as a spectrum than to label them simply M1 or M2.

Q09

Question

During neutrophilic airway inflammation, how do neutrophils and macrophages influence each other through cytokines, lipid mediators, neutrophil extracellular traps, efferocytosis, and metabolic signals, and how can this crosstalk determine persistence versus resolution?

Response

In neutrophilic airway inflammation, macrophages and neutrophils can form a reinforcing loop. Activated macrophages and airway epithelium release IL-1β, TNF, GM-CSF, CXCL8 and related chemokines, while neutrophils contribute LTB4, proteases, oxidants, and additional cytokine signals. These mediators prolong neutrophil survival and recruit more cells. Extracellular traps can help contain microbes, but extracellular DNA, histones, elastase, and myeloperoxidase products can activate epithelial and macrophage danger-sensing pathways, including inflammasome-related signaling, and increase mucus and tissue injury.

Metabolism also matters. Neutrophils depend heavily on glycolysis, allowing them to function in hypoxic mucus and inflamed tissue. High lactate, succinate, low oxygen, and oxidative stress can alter macrophage HIF-1α and mitochondrial signaling and favor persistent inflammatory cytokine production. Conversely, macrophage metabolites with anti-inflammatory effects, together with IL-10 and pro-resolving lipids, can help terminate the response.

Resolution requires neutrophil apoptosis and effective macrophage efferocytosis. Engulfment of apoptotic neutrophils reduces pro-inflammatory cytokines and promotes TGF-β, IL-10 and lipid-mediator class switching. When efferocytosis is defective, NET burden is high, or bacterial/viral signals continue, inflammation persists. This pattern is relevant to COPD, bronchiectasis, severe infection, and neutrophilic asthma, although the relative contribution of each mechanism differs across diseases.

Q10

Question

During allergic airway inflammation, how do mast cells and eosinophils, with basophils where relevant, interact with dendritic cells and macrophages to amplify or restrain type 2 immunity?

Response

Allergic airway inflammation is organized around a type 2 network rather than one single cell. Allergen-exposed epithelium releases alarmins such as TSLP, IL-33 and IL-25. Dendritic cells then promote Th2 differentiation, while ILC2 cells provide additional IL-5 and IL-13. Mast cells, after IgE-dependent activation, rapidly release histamine, PGD2, leukotrienes and cytokines. Basophils can contribute early IL-4 and lipid mediators in some settings. These signals recruit and sustain eosinophils through IL-5 and eotaxin pathways.

Eosinophils amplify inflammation by releasing cysteinyl leukotrienes, granule proteins, cytokines and growth factors. Their IL-4/IL-13 can reinforce macrophage programs associated with type 2 immunity, mucus production and remodeling. Macrophages can in turn produce CCL17, CCL22 and other mediators that support Th2-cell trafficking. Dendritic cells are repeatedly exposed to mast-cell and eosinophil products, which can alter antigen uptake and costimulation and thereby maintain allergen-specific T-cell responses.

The network also contains brakes. Macrophage efferocytosis of dying eosinophils, IL-10/TGF-β, regulatory T cells, and pro-resolving lipid mediators can reduce further recruitment. If these pathways are insufficient, mast-cell activation, eosinophil degranulation, and macrophage remodeling signals become a positive-feedback circuit. Basophils are usually less abundant than mast cells and eosinophils, so their importance is more context dependent.

Q11

Question

During acute bacterial urinary tract infection, how do recruited neutrophils and monocyte-derived macrophages cooperate or compete in pathogen clearance, cytokine production, efferocytosis, and mucosal repair?

Response

During acute bacterial UTI, neutrophils arrive quickly and provide high-capacity bacterial killing, whereas recruited monocytes and monocyte-derived macrophages have a broader role that evolves over time. Both cell types phagocytose bacteria, but neutrophils are specialized for rapid oxidant production, degranulation, and short-lived antimicrobial activity. Monocytes/macrophages produce IL-1, TNF and chemokines that sustain recruitment, can kill bacteria directly, and process debris. In this sense they cooperate: macrophage-derived signals bring neutrophils in, and neutrophils reduce the bacterial burden that would otherwise keep macrophages activated.

There can also be competition or functional conflict. Large numbers of neutrophils consume oxygen and nutrients and release proteases or extracellular traps that injure urothelium. Macrophages exposed to the same damage signals may remain inflammatory rather than moving into a repair state. Conversely, macrophage efferocytosis of apoptotic neutrophils actively terminates the inflammatory phase, so premature or excessive suppression of neutrophils could impair clearance while delayed clearance of dead neutrophils worsens tissue injury.

After bacterial control, macrophages become especially important for resolution. They remove apoptotic cells, release IL-10, TGF-β and growth factors, and support urothelial regeneration. The balance between cooperation and competition therefore changes with time: early inflammation favors joint microbial killing; later, macrophage-mediated clearance of neutrophils and tissue repair should dominate.

Q12

Question

In chronic bladder mucosal inflammation, how might mast-cell–macrophage crosstalk alter sensory signaling, vascular permeability, epithelial barrier function, and the persistence or resolution of inflammation?

Response

In chronic bladder mucosal inflammation, mast cells and macrophages can reinforce both inflammatory and sensory pathways. Activated mast cells release histamine, tryptase, prostaglandins, leukotrienes, TNF and growth factors. Histamine and lipid mediators increase vascular permeability, while tryptase can activate protease-activated receptors. These signals also sensitize afferent nerves, including pathways involving TRPV1 and neuropeptides. Macrophages recruited or activated in the same tissue can produce IL-1β, TNF, chemokines, PGE2 and nerve-growth-related signals, further increasing pain and urgency.

Barrier dysfunction makes the loop easier to sustain. Increased vascular leakage brings plasma proteins and leukocytes into the mucosa, and damaged urothelium becomes more permeable to urinary solutes and microbial or damage-associated signals. Those signals stimulate both mast cells and macrophages, creating a positive-feedback circuit. Persistent macrophage inflammatory activation can delay epithelial repair, while mast-cell proteases can alter tight junctions and extracellular matrix.

Resolution is possible when the initiating trigger is removed and macrophages switch toward efferocytosis and repair, with more IL-10, TGF-β and pro-resolving lipid signaling. Reduced mast-cell activation and restoration of the urothelial barrier then lowers neural and vascular stimulation. In human chronic bladder pain syndromes, however, mast-cell numbers and activation are variable, so this mechanism is plausible and supported in subsets rather than being a universal explanation for all patients.

Q13

Question

In ulcerative colitis, how do neutrophil recruitment, neutrophil extracellular trap formation, neutrophil metabolism, and neutrophil–macrophage crosstalk contribute to mucosal injury versus inflammatory resolution?

Response

In ulcerative colitis, neutrophils are a hallmark of active disease, including cryptitis and crypt abscesses. They are recruited by epithelial and myeloid chemokines such as CXCL8, complement fragments, and lipid mediators. This is initially protective because neutrophils phagocytose

bacteria that cross a damaged barrier. The problem is persistence: elastase, myeloperoxidase products, reactive oxygen species, and other granule enzymes can injure epithelial cells and widen barrier defects.

NET formation adds another layer. NETs can trap microbes, but extracellular DNA, histones, proteases and oxidant enzymes can damage mucosa and provide danger signals to macrophages. Depending on context, NET material may activate TLR- and inflammasome-related pathways and sustain IL-1β/TNF production. Neutrophil metabolism is strongly glycolytic; hypoxia and high metabolic demand allow continued function in inflamed crypts but can also contribute to low tissue oxygen and altered macrophage metabolism.

Resolution depends on neutrophil apoptosis and macrophage efferocytosis. Efficient clearance shifts macrophages away from inflammatory cytokines and toward IL-10, TGF-β, pro-resolving lipids, and epithelial repair. Thus the same neutrophil response can be antimicrobial early and tissue-damaging later. The strongest clinical evidence is for association of mucosal neutrophilia with active disease; the precise causal importance of individual NET or metabolic pathways is still less firmly established in humans.

Q14

Question

In Crohn's disease, how can dysregulated interactions among neutrophils, monocyte-derived macrophages, and dendritic cells alter microbial handling, antigen presentation, inflammatory persistence, and epithelial repair?

Response

Crohn's disease can involve defective microbial containment together with an exaggerated secondary inflammatory response. Neutrophils are needed for rapid killing of bacteria that cross the mucosa, but recruitment and function may be poorly coordinated in some patients. If organisms or microbial products persist, CCR2-dependent monocytes enter the tissue and can differentiate into inflammatory macrophages that produce TNF, IL-1β, IL-6 and IL-23. These cytokines support Th1/Th17 responses and maintain further myeloid recruitment.

Dendritic cells sample microbial and dietary antigens and can promote adaptive immunity when exposed to inflammatory cytokines and danger signals. Genetic pathways relevant to Crohn's disease, including NOD2 and autophagy-related pathways such as ATG16L1, can alter microbial sensing or handling in several cell types. The result may be incomplete bacterial clearance but prolonged antigen presentation—a combination that favors chronic inflammation rather than effective defense.

The same cells also participate in repair. Macrophages can clear apoptotic neutrophils and produce growth factors and TGF-β, while dendritic cells can promote tolerance under non-inflammatory conditions. In Crohn's disease, persistent microbial stimulation, cytokine signaling, hypoxia and matrix damage can prevent this transition. Excessive repair signaling can also contribute to fibrosis and strictures. Therefore the pathology is not simply 'too many' neutrophils or macrophages, but a failure to coordinate microbial handling, antigen presentation, inflammatory shutdown, and epithelial restitution.

Q15

Question

In severe neutrophilic asthma, how do neutrophils, macrophages, and dendritic cells form self-sustaining inflammatory circuits, and what roles may neutrophil extracellular traps, inflammasome signaling, microbial signals, and corticosteroid resistance play?

Response

Severe neutrophilic asthma can be maintained by a loop linking airway epithelium, macrophages, dendritic cells, Th17-type responses, and neutrophils. Epithelial and macrophage IL-1β, TNF, IL-6, GM-CSF and CXCL8 promote neutrophil recruitment and survival. Dendritic cells exposed to microbial products and inflammatory cytokines can support IL-17-producing T-cell responses, which further increase epithelial neutrophil chemokines. Neutrophils then release elastase, reactive oxygen species and inflammatory mediators that damage epithelium and provide new danger signals.

NETs may intensify this circuit. Extracellular DNA, histones and neutrophil enzymes can stimulate epithelial cells and macrophages and may promote inflammasome activation, including NLRP3/IL-1β pathways. Chronic bacterial colonization, altered airway microbiota, or recurrent viral infection can keep pattern-recognition receptors activated, making the inflammation difficult to switch off.

Corticosteroid resistance is multifactorial. Oxidative stress, p38-MAPK-related signaling, altered glucocorticoid-receptor function, persistent IL-17/IL-1 pathways, and the relative steroid insensitivity and prolonged survival of neutrophils can all contribute. These mechanisms are plausible and supported to different degrees, but severe neutrophilic asthma is heterogeneous; not every patient has a NET- or inflammasome-dominant phenotype. Clinically, the important concept is that persistent microbial/danger signaling can make the neutrophil-macrophage-DC circuit self-sustaining even when classic type 2 inflammation is limited.

Q16

Question

In eosinophilic asthma, how do eosinophils, mast cells, and where relevant basophils interact with macrophages, dendritic cells, and airway epithelium to initiate, amplify, and resolve type 2 inflammation?

Response

Eosinophilic asthma usually begins at the airway epithelium. Allergens, viruses, pollutants, or mechanical stress can trigger TSLP, IL-33 and IL-25. These alarmins activate dendritic cells and ILC2 cells. Dendritic cells promote allergen-specific Th2 responses; Th2 and ILC2 cells produce IL-5, while IL-4 and IL-13 drive IgE production, mucus, and eotaxin expression. Mast cells activated through IgE rapidly release histamine, PGD2 and cysteinyl leukotrienes. Basophils can add IL-4 and other type 2 signals in some patients.

Eosinophils recruited by IL-5/eotaxin pathways release major basic protein, eosinophil peroxidase, leukotrienes, cytokines and TGF-β. These products can injure epithelium, increase bronchial reactivity and contribute to remodeling. IL-4/IL-13 also alter macrophage function toward type 2/repair-associated states; those macrophages can produce chemokines and growth factors that sustain the local network. Dendritic cells continue to present allergen and receive signals from mast cells and eosinophils, maintaining adaptive responses.

Resolution requires reduced epithelial alarmin release, loss of eosinophil survival signals, eosinophil apoptosis, and macrophage efferocytosis. IL-10, TGF-β and pro-resolving lipid mediators help shut down recruitment and support repair. If repair signals such as TGF-β remain excessive, however, airway remodeling may continue even after eosinophil counts fall. This helps explain why eosinophilic asthma is a network disorder rather than simply an eosinophil-count disorder.

Q17

Question

In recurrent urinary tract infection, how can prior infection reshape neutrophil and monocyte or macrophage responses at the bladder mucosa through trained immunity, tolerance, or tissue remodeling, and how strong is the evidence for each mechanism?

Response

Prior UTI can plausibly reprogram later innate responses through at least three mechanisms, but the evidence is not equally strong. First, trained immunity refers to epigenetic and metabolic changes in monocytes or their bone-marrow progenitors after an initial stimulus. A later infection can then trigger faster or stronger cytokine and antimicrobial responses. This concept is well established in innate-immunity research, but direct proof that it prevents recurrent human UTI by reprogramming bladder monocytes, macrophages, or neutrophil progenitors is still limited and largely preclinical.

Second, repeated stimulation can produce tolerance rather than training. Persistent or repeated TLR exposure can reduce later TNF/IL-1 responses while preserving selected antimicrobial functions. Such tolerance could limit bladder damage, but it might also permit bacterial persistence. UTI-specific evidence exists mainly from experimental systems rather than definitive human mechanistic studies.

Third, tissue remodeling is strongly plausible. Prior infection can alter urothelial differentiation, barrier integrity, local stromal and neural signaling, and the composition of resident immune cells. Intracellular bacterial reservoirs and altered epithelial repair can also change the threshold for recurrence. This tissue-memory concept has substantial experimental support, although the exact contribution of macrophage versus epithelial programming remains uncertain.

Neutrophils themselves are short-lived, so durable 'memory' is more likely to arise from progenitors, resident cells, or the remodeled tissue environment than from long-lived mature neutrophils. Overall, remodeling has the clearest tissue-specific rationale; trained immunity and tolerance are promising but not yet firmly established as dominant mechanisms in recurrent human UTI.

Q18

Question

In bladder cancer, how do tumor-associated neutrophils and macrophages interact within the mucosal tumor microenvironment to influence antigen presentation, T-cell activity, angiogenesis, invasion, and response to immunotherapy?

Response

In bladder cancer, tumor-associated macrophages (TAMs) and tumor-associated neutrophils (TANs) are highly plastic. Tumor and stromal cells can recruit them through CCL2/CSF1 and CXCL chemokine pathways and then reprogram them with hypoxia, lactate, TGF-β, IL-10 and other signals. Suppressive TAMs may reduce effective antigen presentation and T-cell activation through IL-10, TGF-β, PD-L1, arginase-related metabolism and adenosine pathways. TANs can acquire similar suppressive or myeloid-derived-suppressor-like features, using ROS, arginase, proteases and other mediators to impair T-cell function.

Both populations can promote angiogenesis and invasion. TAM-derived VEGF, matrix metalloproteinases and growth factors support new vessels and matrix remodeling. TAN proteases and NETs can also change the extracellular matrix and, in some contexts, facilitate tumor-cell invasion or dissemination. The two cell types reinforce each other through chemokines and inflammatory mediators, so blocking only one pathway may lead to compensation by the other.

For immunotherapy, a suppressive myeloid-rich microenvironment may reduce response to checkpoint blockade. On the other hand, bladder cancer also shows that myeloid activation can be therapeutically useful: intravesical BCG depends on a strong local innate inflammatory response involving neutrophils, macrophages and other cells before adaptive antitumor immunity develops. Therefore the goal is not global myeloid suppression but selective reprogramming. Human evidence supports associations between myeloid states and outcome, but exact TAN/TAM subsets and causal pathways remain heterogeneous across tumors.

Q19

Question

Which mechanistically plausible strategies could suppress pathogenic neutrophil recruitment or neutrophil-extracellular-trap-associated injury in ulcerative colitis while preserving antimicrobial defense and macrophage-mediated repair? Explain predicted benefits, trade-offs, and evidence gaps.

Response

A reasonable strategy in ulcerative colitis is to reduce the most injurious part of the neutrophil response rather than remove neutrophils completely. One approach is partial inhibition of chemokine-driven recruitment, for example CXCR2/CXCL8-related pathways. This could reduce crypt accumulation and protease/oxidant injury, but strong blockade may impair bacterial clearance and wound defense. Local or short-course inhibition would therefore be more attractive than prolonged systemic neutrophil suppression.

A second approach is to target NET-associated damage. DNase can dismantle the DNA scaffold, while PAD4, neutrophil elastase, or myeloperoxidase inhibition could reduce NET formation or toxicity. The benefit would be less extracellular histone/protease exposure and fewer danger signals to macrophages. The trade-off is that NETs sometimes contribute to microbial containment; DNase also removes DNA without necessarily neutralizing bound proteases or histones.

A third, potentially more physiologic strategy is to accelerate resolution. Pro-resolving lipid mediators or therapies that improve macrophage efferocytosis could shorten neutrophil residence while preserving early antimicrobial function. Enhancing epithelial barrier repair would reduce the stimulus for repeated recruitment.

Most of these neutrophil/NET-directed ideas remain mechanistically attractive rather than clinically established for UC. Major gaps include identifying which patients truly have NET-driven disease, defining a safe degree and timing of neutrophil inhibition, and proving that treatment reduces mucosal injury without increasing invasive infection or delaying macrophage-mediated repair.

Q20

Question

How could metabolic or epigenetic reprogramming of intestinal macrophages and granulocytes be used to restore mucosal homeostasis in inflammatory bowel disease, and what mechanisms could cause loss of efficacy or unintended immunosuppression?

Response

Metabolic and epigenetic reprogramming aims to change myeloid-cell behavior without necessarily depleting the cells. In inflammatory intestinal macrophages, high glycolytic flux, HIF-1α signaling, succinate, mTOR activity and inflammatory chromatin programs can sustain IL-1β/TNF production. Strategies that favor AMPK activity, mitochondrial oxidative metabolism, itaconate-like anti-inflammatory signaling, or selected epigenetic regulators could shift macrophages toward efferocytosis, IL-10 production and tissue repair. For neutrophils and other granulocytes, reducing excessive glycolysis, ROS generation or NETosis might limit injury while allowing basic phagocytosis to continue.

The main challenge is selectivity. The same metabolic pathways are needed for antimicrobial defense. Too much suppression of glycolysis, NADPH-oxidase function, inflammasome activity, or chromatin accessibility could weaken phagocytosis, oxidative killing, cytokine responses and vaccine-related immunity. Gut-targeted delivery, transient treatment, or receptor-based delivery to inflammatory myeloid subsets would therefore be preferable to systemic metabolic inhibition.

Loss of efficacy can occur because myeloid cells are continually replaced from bone marrow, inflammatory cytokines and microbial products can override the programmed state, and parallel pathways compensate. Epigenetic states are also plastic rather than permanently fixed. Unintended immunosuppression may present as infection, impaired wound healing, or failure to contain translocating bacteria. At present, this is mainly a translational strategy rather than a standard IBD treatment, and biomarkers are needed to identify which metabolic program is dominant in an individual patient.

Q21

Question

How might biologics that alter type 2 cytokine signaling reshape the eosinophil–mast-cell–macrophage–dendritic-cell network in airway mucosa, beyond simply reducing eosinophil counts?

Response

Type 2 biologics can remodel the airway myeloid network even when their most visible effect is a lower blood or sputum eosinophil count. Anti-IL-5 or anti-IL-5-receptor therapy reduces eosinophil maturation, survival and tissue recruitment, which also reduces eosinophil-derived leukotrienes, granule proteins, IL-4/IL-13, TGF-β and danger signals. That can indirectly lower macrophage and dendritic-cell activation and reduce the amount of damaged epithelium feeding the inflammatory loop.

Blocking IL-4/IL-13 signaling has broader network effects. It can reduce eotaxin production, mucus programs, IgE-related amplification, and IL-4/IL-13-driven macrophage polarization. Dendritic-cell/T-cell crosstalk may also become less type-2 biased. Upstream blockade of epithelial alarmin pathways such as TSLP, and in some settings IL-33-related pathways, can reduce activation of dendritic cells, ILC2 cells, mast cells and downstream granulocytes simultaneously.

Mast-cell numbers may not fall substantially with eosinophil-targeted treatment, but their activation threshold and surrounding mediator environment can change. Macrophage repair programs may also normalize as epithelial injury decreases. The trade-off is that type 2 cytokines participate in helminth defense and aspects of barrier repair, so complete pathway suppression is not biologically neutral. Clinically, response heterogeneity reflects where a patient's dominant inflammatory circuit sits: eosinophil-dependent, IL-4/IL-13-dominant, or upstream alarmin-driven.

Q22

Question

What myeloid-directed strategies could interrupt neutrophil–macrophage inflammatory circuits in neutrophilic asthma while preserving antibacterial defense, and which proposed mechanisms are clinically established versus preclinical?

Response

The safest concept in neutrophilic asthma is to interrupt excessive recruitment or activation while leaving enough neutrophil and macrophage function for bacterial defense. Macrolide therapy has the most established clinical role in selected patients with persistent severe asthma; its benefit is partly antimicrobial but also immunomodulatory, with effects on epithelial and myeloid cytokine production. The trade-offs are antimicrobial resistance, QT/drug interactions, and microbiome effects, so it is not a pure neutrophil-targeted treatment.

More direct approaches remain less established. CXCR2 antagonism can reduce neutrophil trafficking, but clinical benefit has been inconsistent and excessive blockade could increase infection risk. Targeting IL-1β/NLRP3, GM-CSF, LTB4 signaling, neutrophil elastase, NET formation/PAD4, or macrophage inflammatory metabolism has strong mechanistic or preclinical rationale, but these are not standard proven therapies for neutrophilic asthma. Treating chronic infection or bronchiectasis when present can also remove the microbial stimulus that sustains the circuit.

Preserving host defense probably requires partial, phenotype-guided and preferably airway-localized therapy rather than systemic neutrophil depletion. Biomarkers such as sputum neutrophils, infection status, inflammasome activity or NET signatures may eventually guide selection. At present, there is no widely established drug that specifically breaks the neutrophil-macrophage circuit while reliably preserving antibacterial function; most proposed molecular targets remain experimental or have limited clinical validation.

Q23

Question

Could trained-immunity or mucosal-vaccine approaches beneficially reprogram monocytes or macrophages and neutrophils to reduce recurrent urinary tract infection? Explain the proposed mechanisms, potential tissue-specific risks, and current evidence limitations.

Response

Trained-immunity and mucosal-vaccine approaches are plausible ways to reduce recurrent UTI, but the mechanisms should be separated. A vaccine that improves pathogen-specific mucosal antibodies and T-cell responses could lower the bacterial inoculum reaching the bladder and therefore reduce the need for repeated, damaging neutrophil bursts. A trained-immunity approach would instead use pattern-recognition signaling

to epigenetically and metabolically prime monocytes or bone-marrow progenitors so that later macrophage and neutrophil responses are faster or better coordinated.

Potential benefits include earlier bacterial containment, more effective phagocytosis, and a quicker transition to macrophage-mediated resolution. However, stronger innate memory is not automatically beneficial. In the bladder, excessive trained responses could increase neutrophil recruitment, urothelial injury, pain, fibrosis, or inflammatory sensitization. Conversely, inducing too much tolerance could blunt cytokine responses and permit ascending infection. Because mature neutrophils are short-lived, durable reprogramming would likely need to occur at the progenitor level or through tissue-resident macrophages and epithelial/stromal memory.

Current evidence is limited by small and heterogeneous clinical studies, different vaccine formulations and routes, variable bacterial species, and incomplete mechanistic sampling of the bladder mucosa. Some immunoprophylactic approaches appear clinically promising, but direct proof that benefit is mediated by trained monocytes/macrophages or neutrophils is still weak. A successful strategy will probably need pathogen specificity, tissue targeting, and biomarkers that distinguish protective training from harmful chronic inflammation.

Q24

Question

How could selective reprogramming of tumor-associated macrophages and neutrophils improve antitumor immunity in bladder cancer while minimizing disruption of protective urinary mucosal immunity? Compare mechanistic approaches and key translational barriers.

Response

For bladder cancer, selective reprogramming is more attractive than broad depletion because macrophages and neutrophils are also needed for urinary-mucosal defense. One macrophage approach is to reduce recruitment or survival of suppressive TAMs through CCL2/CCR2 or CSF1/CSF1R pathways. A second is functional reprogramming with CD40, TLR/STING, PI3Kγ, metabolic, or other innate-activation strategies so that macrophages improve antigen presentation and support T-cell killing. Reprogramming may preserve phagocytosis better than complete depletion, but excessive activation can cause cystitis or systemic inflammation.

For neutrophils, CXCR2-pathway blockade could reduce entry of tumor-promoting TANs, while TGF-β modulation, arginase/adenosine targeting, or inhibition of NET formation and neutrophil proteases could reduce T-cell suppression and invasion. The risk is impaired antibacterial defense and delayed healing. Local intravesical delivery is therefore particularly appealing in bladder cancer because it can expose the tumor while limiting systemic myeloid suppression. BCG already demonstrates the value of local innate activation, although it is not a selective TAM/TAN reprogramming therapy.

Key translational barriers are myeloid plasticity, lack of unique markers separating harmful tumor-associated cells from protective mucosal cells, rapid replacement from bone marrow, tumor-to-tumor heterogeneity, and compensatory recruitment pathways. Timing with checkpoint blockade or chemotherapy also matters. The most realistic strategy is likely biomarker-guided, locally delivered, and combination-based rather than permanent systemic suppression of a whole myeloid lineage.

Japanese (JP)

Anonymous Model E | 日本語 | Informal Response Package

Anonymous Model E

日本語

Q01

質問

健常な腸管粘膜において、好中球は常在マクロファージおよび樹状細胞と相互作用しながら、どのようにバリア防御に寄与しますか。また、好中球による組織障害を通常どのような機序が抑制していますか。

回答

健常な腸管粘膜では、好中球が固有層に大量に常在しているわけではありません。上皮バリアが破綻し、細菌が粘液層を越えたり、常在マクロファージなどのセンチネル細胞が危険シグナルを感知したときに、CXCL1/CXCL2、ヒトでは CXCL8、補体や脂質メディエーターによって速やかに動員されます。到達した好中球は貪食、活性酸素、顆粒蛋白、必要に応じた NET 形成で細菌や真菌を処理します。常在マクロファージは初期の動員シグナルを出す一方、病原体や細胞残骸の除去も担います。樹状細胞は急性の大量殺菌よりも、抗原取り込みと適応免疫への橋渡しが中心です。

好中球の武器は上皮も傷つけ得るため、健常粘膜には複数の制御機構があります。粘液、タイトジャンクション、抗菌ペプチド、上皮の速い更新がまず微生物侵入を減らします。腸管マクロファージは無害な共生菌に対して比較的低反応性で、IL-10 などの調節性因子を産生します。病原体負荷が低下すると好中球はアポトーシスに入り、マクロファージのエフェロサイトーシスによって除去されます。この過程は IL-10、TGF-β、組織修復、炎症収束性脂質メディエーターを促します。抗酸化系や抗プロテアーゼも副次的損傷を抑えます。

臨床的には、短時間で局所的な好中球反応は防御的ですが、動員が持続し、プロテアーゼ･酸化物･NET が過剰になると、同じ反応が粘膜障害へ転じると考えるのが実用的です。

Q02

質問

健常な腸管粘膜において、好酸球はどのように恒常性の維持に寄与し、マクロファージまたは樹状細胞と情報交換しますか。また、これらの役割は炎症時の好酸球機能とどのように異なりますか。

回答

好酸球は腸管固有層の正常な常在細胞なので、腸で好酸球が見られること自体は病的ではありません。恒常状態では IL-5 と、CCR3 を介する eotaxin 系シグナルによって生存･局在が維持され、バリア監視、局所免疫調節、組織更新に関与します。サイトカイン、脂質メディエーター、増殖因子を介して上皮や間質細胞、IgA 関連応答に影響することもあります。マクロファージとの間では、好酸

球由来 IL-4/IL-13 が一部の修復関連プログラムを促し、逆にマクロファージや樹状細胞由来因子が好酸球の生存や活性化を調節します。樹状細胞成熟や抗原提示への直接作用は、基本的な遊走機構ほど一貫しておらず、状況依存性が高い部分です。

炎症時には同じ細胞が強い組織障害性を示します。主要塩基性蛋白、好酸球カチオン蛋白、好酸球ペルオキシダーゼ、ロイコトリエン、サイトカイン、活性酸素が放出され、上皮透過性、血管･神経反応、他の骨髄系細胞の活性化を増強します。2 型炎症では IL-4/IL-13 依存性のマクロファージ機能を補強し、混合型炎症では好中球や炎症性単球と同時に存在することもあります。

したがって、恒常状態の好酸球は組織適応型の調節･修復細胞としての側面が強く、炎症時には脱顆粒とサイトカイン産生が前面に出ます。両者の境界は明確ではなく、ヒト腸疾患ではかなりの不均一性があります。

Q03

質問

健常な気道粘膜および急性の微生物刺激時に、好中球は肺胞または間質マクロファージ、樹状細胞とどのように協調して宿主防御を担いながら、上皮への二次的損傷を抑えますか。

回答

健常な気道では、肺胞マクロファージが肺胞腔の主要な貪食センチネルで、吸入物に常時さらされながら過剰な炎症を抑えています。急性の微生物刺激では、肺胞または間質マクロファージと気道上皮のパターン認識受容体が活性化し、IL-1、TNF、CXCL 系ケモカイン、G-CSF などを産生して好中球を動員します。好中球は貪食、酸化バースト、顆粒酵素、必要に応じた NET 形成で迅速な抗菌･抗真菌作用を示します。樹状細胞は抗原を取り込み、リンパ節へ移動して適応免疫を開始･調整します。

組織を守るには、病原体負荷が下がった後にこの反応を止めることが重要です。好中球はアポトーシスに入り、マクロファージがエフェロサイトーシスで除去します。これにより IL-1/TNF 系が低下し、IL-10、TGF-β、炎症収束性脂質メディエーターが増えます。α1 アンチトリプシンや分泌型白血球プロテアーゼ阻害因子、抗酸化系、DNase、粘液線毛クリアランス、上皮修復がエラスターゼ、MPO 産物、細胞外 DNA による損傷を抑えます。

病原体が残る、あるいはエフェロサイトーシスや上皮修復が不十分だと、防御回路は慢性炎症回路に変わります。重症肺炎、COPD 増悪、気管支拡張症、一部の重症喘息で、この「抗菌には必要だが上皮も傷つける」という二面性が臨床的に問題になります。

Q04

質問

恒常状態および 2 型炎症時の気道粘膜において、好酸球はマクロファージや樹状細胞とどのように相互作用しますか。

回答

恒常状態の気道では好酸球は比較的少なく、マクロファージや樹状細胞との相互作用も限定的です。肺胞マクロファージは主に粒子除去と炎症抑制を担い、樹状細胞は低レベルで抗原をサンプリングします。少数の好酸球が組織監視や修復に関与する可能性はありますが、健常気道では腸管ほど明確な好酸球中心の恒常性ネットワークは示されていません。

2 型炎症になると状況は大きく変わります。傷害･アレルゲン刺激を受けた上皮から TSLP、IL-33、IL-25 が放出され、樹状細胞や ILC2 が活性化します。Th2/ILC2 由来 IL-5 は好酸球の産生･生存を促し、IL-4/IL-13 は eotaxin、粘液産生、バリア変化を誘導します。動員された好酸球はロイコトリエン、顆粒蛋白、サイトカイン、増殖因子を放出し、IL-4/IL-13 はマクロファージの 2 型･修復関連状態を補強します。マクロファージは CCL17/CCL22 などを産生し、Th2 環境を維持し得ます。樹状細胞はアレルゲン提示を継続し、適応免疫を保ちます。

一方、マクロファージはアポトーシス好酸球を除去し、収束にも関与します。したがって短期の修復には役立つ反応でも、脱顆粒、粘液過剰、気道過敏、リモデリングが持続すれば病原的になります。樹状細胞への好酸球の直接作用は喘息表現型により差があります。

Q05

質問

尿路粘膜における初期の抗菌防御で、好中球は単球またはマクロファージ、樹状細胞とどのように協調しますか。また、過剰な組織障害を防ぐ機序は何ですか。

回答

尿路の初期抗菌防御では、尿路上皮と局所骨髄系細胞が細菌成分を感知し、IL-1、IL-6、CXCL 系ケモカインなどを産生して好中球を速やかに動員します。好中球は通常、最も早い主要エフェクターで、上皮を越えて移動し、貪食、活性酸素、抗菌顆粒蛋白、必要に応じた NET 形成によって細菌を排除します。単球･マクロファージも貪食とサイトカイン産生に加わり、後期には死んだ好中球を除去します。樹状細胞は大量の細菌殺傷より、抗原サンプリングと適応免疫の調整に重点があります。

尿流や上皮脱落そのものにも細菌排除作用があるため、好中球反応が強すぎると逆に傷害が増えます。プロテアーゼ、活性酸素、NET 成分は尿路上皮を傷つけ、透過性を高めます。通常は、速やかな細菌負荷低下、好中球アポトーシス、マクロファージのエフェロサイトーシス、IL-10/TGF-β系の収束シグナル、抗酸化･抗プロテアーゼ機構、尿路上皮再生によって障害が制限されます。

臨床的には、動員が不十分なら細菌持続や上部尿路への進展を許し、反対に好中球が長く残れば排尿痛、血尿、粘膜障害が悪化します。マクロファージは初期防御だけでなく、炎症から修復へ切り替える役割も重要です。

Q06

質問

膀胱または下部泌尿生殖器粘膜において、肥満細胞由来メディエーターはマクロファージや樹状細胞の機能にどのような影響を与えますか。また、このネットワークは宿主防御と炎症症状の双方にどのように関与し得ますか。

回答

膀胱や下部泌尿生殖器粘膜の肥満細胞は、微生物成分、IgE 依存性刺激、組織傷害、補体、神経ペプチドなどで活性化され、ヒスタミン、トリプターゼ、TNF、プロスタグランジン、ロイコトリエン、多様なサイトカインを放出します。急性感染では血管透過性と局所血流を高め、好中球や単球を呼び込みやすくするため、防御に役立つ面があります。

マクロファージと樹状細胞は、サイトカイン受容体、ヒスタミン受容体、プロテアーゼ活性化経路、脂質メディエーター受容体を介して肥満細胞由来シグナルを受けます。TNF や一部脂質メディエーターは樹状細胞成熟や炎症性マクロファージ機能を高め、抗原提示･抗菌反応を増強し得ます。逆に、マクロファージ由来 IL-1/TNF や傷害シグナルも肥満細胞を再活性化します。ただし肥満細胞は常に促炎性というわけではなく、条件により調節性メディエーターも産生します。

同じネットワークは症状の原因にもなります。ヒスタミン、トリプターゼ、プロスタグランジン、ロイコトリエン、NGF 関連シグナルは求心性感覚神経を過敏化し、尿意切迫、疼痛、頻尿を起こします。血管･上皮透過性上昇も尿中刺激物への曝露を増やします。慢性化すると感染がなくても正のフィードバックが残ることがありますが、感染と膀胱痛症候群などでは主な経路が同一とは限りません。

Q07

質問

急性の腸管粘膜炎症とその後の炎症収束過程において、好中球とマクロファージは、遊走･動員シグナル、サイトカイン、エフェロサイトーシス、脂質メディエーター、組織修復プログラムを介してどのように相互作用しますか。

回答

急性腸管炎症では、上皮･骨髄系細胞由来の CXCL ケモカイン、補体、LTB4 などが好中球を速やかに病変へ動員します。好中球は微生物を殺しますが、IL-1 系シグナル、プロテアーゼ、活性酸素、DAMP も放出し、CCR2 依存性単球の流入を増やします。単球は炎症性マクロファージへ分化し、TNF や IL-1βを産生します。微生物や傷害刺激が残ると、このマクロファージシグナルがさらに好中球動員を持続させます。

炎症収束には能動的な切り替えが必要です。病原体が減ると好中球は生存シグナルを失いアポトーシスに入り、表面にホスファチジルセリンを提示します。マクロファージは MerTK などを含む経路でエフェロサイトーシスを行います。これにより促炎性サイトカインが抑えられ、IL-10、TGF-β、増殖因子、組織修復プログラムが増えます。同時に脂質メディエーターもロイコトリエン優位からリポキシン、レゾルビン、プロテクチン、マレシンなどの収束系へ移行し得ます。

その後、修復型のマクロファージ状態が上皮再生、基質再構築、バリア回復を支えます。単純な M1 から M2 への切り替えではなく、実際の腸管マクロファージはより多様です。好中球アポトーシスやエフェロサイトーシスが障害されると、炎症は持続します。

Q08

質問

好酸球由来メディエーターは、腸管粘膜のマクロファージの機能状態や樹状細胞の活性化をどのように変化させますか。また、これらの相互作用は免疫寛容、組織修復、炎症のバランスをどのように変え得ますか。

回答

好酸球はサイトカイン、脂質メディエーター、顆粒蛋白、増殖因子を通じて腸管マクロファージ機能を変え得ます。好酸球由来 IL-4/IL-13 は、組織修復、細胞外基質処理、2 型応答に関連するマクロファージプログラムを促すことがあります。TGF-βなどは短期的には上皮修復を助けますが、持続すれば線維化にもつながり得ます。脂質メディエーターは血管･骨髄系細胞活性を変え、強い脱顆粒時には顆粒蛋白や酸化物そのものが DAMP 様刺激になります。

樹状細胞への影響はさらに状況依存性が高いと考えられます。好酸球由来サイトカインは樹状細胞の成熟、移動、抗原提示時のサイトカイン環境を変えます。静かな粘膜では食物･共生菌抗原への過剰反応を避ける方向に働く可能性がある一方、強い 2 型炎症や混合炎症では T 細胞活性化と追加の顆粒球動員を促し得ます。好酸球が抗原提示を行える場面はありますが、ヒト腸粘膜の主要な専門抗原提示細胞とは通常考えません。

結果は活性化の強さと時間に左右されます。中等度のシグナルは寛容と修復、持続する IL-4/IL-13/TGF-βはリモデリング、強い脱顆粒は上皮障害と樹状細胞活性化に傾きます。マクロファージは M1/M2 の二分法より連続的な機能状態として理解する方が実際的です。

Q09

質問

好中球性気道炎症において、好中球とマクロファージは、サイトカイン、脂質メディエーター、好中球細胞外トラップ、エフェロサイトーシス、代謝シグナルを介してどのように相互作用しますか。また、そのクロストークは炎症の持続と収束をどのように左右しますか。

回答

好中球性気道炎症では、マクロファージと好中球が正のフィードバックを形成します。マクロファージと気道上皮は IL-1β、TNF、GM-CSF、CXCL8 などを放出し、好中球の流入と生存を増やします。好中球は LTB4、プロテアーゼ、活性酸素、炎症メディエーターを返し、さらにマクロファージと上皮を刺激します。NET は微生物を封じ込める一方、細胞外 DNA、ヒストン、エラスターゼ、

MPO 産物が上皮やマクロファージの危険感知、インフラマソーム関連シグナルを刺激し、粘液産生と組織障害を増やすことがあります。

代謝環境も重要です。好中球は主に解糖系でエネルギーを得るため、低酸素の粘稠な分泌物中でも働けます。しかし乳酸、コハク酸、低酸素、酸化ストレスはマクロファージの HIF-1αやミトコンドリア機能を変化させ、IL-1βなどの産生を維持し得ます。反対に IL-10、炎症収束性脂質、抗炎症代謝プログラムは反応を終息させます。

収束には好中球アポトーシスとマクロファージのエフェロサイトーシスが必要です。これが成功すると促炎サイトカインが下がり、TGF-β、IL-10、脂質メディエーターの切り替えが進みます。エフェロサイトーシス不良、NET 過剰、持続感染があると炎症は慢性化しやすく、COPD、気管支拡張症、重症感染、好中球性喘息で重要になります。

Q10

質問

アレルギー性気道炎症において、肥満細胞と好酸球、さらに該当する場合には好塩基球は、樹状細胞およびマクロファージとどのように相互作用して 2 型免疫を増幅または抑制しますか。

回答

アレルギー性気道炎症は、単一細胞ではなく 2 型免疫ネットワークとして理解するのが適切です。アレルゲン刺激を受けた上皮から TSLP、IL-33、IL-25 が放出され、樹状細胞は Th2 分化を促し、ILC2 も IL-5/IL-13 を産生します。肥満細胞は IgE 依存性にヒスタミン、PGD2、ロイコトリエン、サイトカインを迅速に放出します。状況により好塩基球も早期 IL-4 や脂質メディエーターを補います。IL-5 と eotaxin 系が好酸球を動員･維持します。

好酸球はシステイニルロイコトリエン、顆粒蛋白、サイトカイン、増殖因子で炎症を増幅します。好酸球由来 IL-4/IL-13 はマクロファージの 2 型･修復関連状態を補強し、マクロファージは CCL17/CCL22 などを介して Th2 細胞の局在を支えます。樹状細胞は肥満細胞･好酸球由来メディエーターの影響を受けながら抗原提示を続け、アレルゲン特異的 T 細胞応答を維持します。

抑制側には、マクロファージによるアポトーシス好酸球の除去、IL-10/TGF-β、制御性 T 細胞、炎症収束性脂質があります。これらが弱いと、肥満細胞活性化、好酸球脱顆粒、マクロファージのリモデリングシグナルが正のフィードバックになります。好塩基球の寄与は一般に肥満細胞･好酸球より状況依存性が高いです。

Q11

質問

急性細菌性尿路感染において、動員された好中球と単球由来マクロファージは、病原体排除、サイトカイン産生、エフェロサイトーシス、粘膜修復の過程でどのように協調または競合しますか。

回答

急性細菌性尿路感染では、好中球が最も早く大量の殺菌を担い、動員単球･単球由来マクロファージは時間とともに役割を変えます。両者とも細菌を貪食しますが、好中球は酸化バースト、脱顆粒、短時間の強い抗菌作用に特化しています。単球･マクロファージは IL-1、TNF、ケモカインを産生して好中球を呼び込み、細菌や組織残骸も処理します。初期には、マクロファージが動員を支え、好中球が細菌量を素早く減らすという協力関係が中心です。

ただし反応が過剰になると競合･機能衝突が起こります。多数の好中球は酸素や栄養を消費し、プロテアーゼ、活性酸素、NET で尿路上皮を傷つけます。その DAMP がマクロファージを炎症性状態に留めることもあります。一方、マクロファージがアポトーシス好中球をエフェロサイトーシスすることは炎症終息の中心です。したがって好中球を早すぎる段階で抑えれば殺菌が落ち、死細胞除去が遅れれば組織障害が増えます。

細菌が制御された後は、マクロファージが IL-10、TGF-β、増殖因子を産生し、尿路上皮修復を支えます。実際には、感染初期は共同防御、後期はマクロファージ主導の清掃･修復という時間的役割分担で考えると理解しやすいです。

Q12

質問

慢性膀胱粘膜炎症において、肥満細胞とマクロファージのクロストークは、感覚神経シグナル、血管透過性、上皮バリア機能、そして炎症の持続または収束をどのように変化させ得ますか。

回答

慢性膀胱粘膜炎症では、肥満細胞とマクロファージが炎症だけでなく感覚神経･血管反応も相互に増幅します。肥満細胞はヒスタミン、トリプターゼ、プロスタグランジン、ロイコトリエン、TNF、増殖因子を放出します。ヒスタミンと脂質メディエーターは血管透過性を高め、トリプターゼは PAR 系受容体を活性化し、これらは TRPV1 などの求心性感覚経路を過敏化します。マクロファージも IL-1β、TNF、ケモカイン、PGE2、NGF 関連シグナルを産生し、疼痛･尿意切迫を強めます。

バリア障害はこの回路を維持しやすくします。血管漏出で血漿蛋白と炎症細胞が流入し、傷害された尿路上皮は尿中溶質や微生物/DAMP 刺激を受けやすくなります。これらの刺激が再び肥満細胞とマクロファージを活性化し、正のフィードバックを作ります。持続する炎症性マクロファージは上皮修復を遅らせ、肥満細胞プロテアーゼもタイトジャンクションや基質に影響し得ます。

原因刺激が除去され、マクロファージがエフェロサイトーシスと修復へ移行して IL-10、TGF-β、収束性脂質を増やすと、肥満細胞活性と神経･血管刺激も低下します。ただしヒトの慢性膀胱痛症候群では肥満細胞所見に個人差が大きく、この経路は全患者に共通する単一原因ではありません。

Q13

質問

潰瘍性大腸炎において、好中球の動員、好中球細胞外トラップ形成、好中球代謝、ならびに好中球–マクロファージ間クロストークは、粘膜障害と炎症収束にそれぞれどのように寄与しますか。

回答

潰瘍性大腸炎の活動期では、好中球が陰窩に入り陰窩炎･陰窩膿瘍を形成することが代表的所見です。CXCL8 などのケモカイン、補体、脂質メディエーターが動員を促します。バリアが壊れた初期には、侵入細菌を貪食するために必要な防御反応です。しかし流入が持続すると、エラスターゼ、MPO 産物、活性酸素、顆粒酵素が上皮を傷つけ、さらにバリア障害を拡大します。

NET も二面性があります。微生物を捕捉できる反面、細胞外 DNA、ヒストン、プロテアーゼ、酸化酵素が組織中に残ると細胞毒性を示し、TLR やインフラマソーム関連経路を介してマクロファージの IL-1β/TNF 産生を持続させる可能性があります。好中球は解糖系依存性が高いため低酸素の陰窩内でも活動できますが、大量の酸素消費と代謝産物は局所低酸素とマクロファージ代謝にも影響します。

炎症収束には好中球のアポトーシスとマクロファージのエフェロサイトーシスが必要です。効率よく除去されると IL-10、TGF-β、炎症収束性脂質、上皮修復が進みます。臨床的には粘膜好中球増加と活動性の関連は強い一方、特定の NET 経路や代謝経路がヒト病態のどの程度を直接駆動するかは、まだ同じレベルには確立していません。

Q14

質問

クローン病において、好中球、単球由来マクロファージ、樹状細胞の相互作用の異常は、微生物処理、抗原提示、炎症の持続、上皮修復をどのように変化させますか。

回答

クローン病では、微生物封じ込めの不十分さと、その後の過剰な炎症が同時に起こり得ます。好中球は本来、粘膜を越えた細菌を迅速に処理しますが、一部患者では動員時期や殺菌効率がうまく調整されない可能性があります。微生物やその産物が残ると、CCR2 依存性単球が組織に入り、TNF、IL-1β、IL-6、IL-23 を産生する炎症性マクロファージへ分化し、Th1/Th17 反応と追加の骨髄系細胞動員を維持します。

樹状細胞は微生物･食物抗原を取り込み、炎症性サイトカイン環境では共刺激と抗原提示を強めます。クローン病に関連する NOD2 や ATG16L1 などの経路は複数の細胞で微生物認識やオートファジー処理を変え得ます。その結果、細菌が十分除去されないまま抗原提示と炎症シグナルだけが長く続く、という不利な組み合わせが生じます。

一方、マクロファージはアポトーシス好中球を除去し、増殖因子や TGF-βを産生して修復にも関与します。樹状細胞も非炎症環境では寛容を支えます。持続する微生物刺激、サイトカイン、低酸素、基質障害がこの切り替えを阻害し、過剰な TGF-β系修復は線維化･狭窄に寄与し得ます。病態の核心は単一細胞数ではなく、微生物処理、抗原提示、炎症停止、上皮修復の協調不全です。

Q15

質問

重症好中球性喘息において、好中球、マクロファージ、樹状細胞はどのように自己持続的な炎症回路を形成しますか。また、好中球細胞外トラップ、インフラマソームシグナル、微生物由来シグナル、ステロイド抵抗性はどのような役割を果たし得ますか。

回答

重症好中球性喘息では、気道上皮、マクロファージ、樹状細胞、Th17 系反応、好中球が自己持続回路を作り得ます。上皮とマクロファージ由来 IL-1β、TNF、IL-6、GM-CSF、CXCL8 が好中球を動員し生存を延長します。樹状細胞は微生物産物や炎症性サイトカインを受けると IL-17 産生 T 細胞応答を支え、IL-17 は上皮の好中球ケモカイン産生をさらに増やします。好中球はエラスターゼ、活性酸素、炎症メディエーターを放出し、上皮障害と新たな DAMP を作ります。

NET はこのループを強化する可能性があります。細胞外 DNA、ヒストン、好中球酵素は上皮とマクロファージを刺激し、NLRP3/IL-1βを含むインフラマソーム関連シグナルを促進し得ます。慢性細菌定着、気道微生物叢の変化、反復ウイルス感染もパターン認識受容体刺激を持続させます。

ステロイド抵抗性は多因子性で、酸化ストレス、p38-MAPK 関連シグナル、グルココルチコイド受容体機能変化、IL-17/IL-1 系の持続、好中球自体の相対的なステロイド低感受性と長い生存などが関与し得ます。ただし全患者が NET 優位･インフラマソーム優位ではありません。臨床的には「好中球性喘息」という一群の中にも複数の機序があると考える必要があります。

Q16

質問

好酸球性喘息において、好酸球、肥満細胞、さらに該当する場合には好塩基球は、マクロファージ、樹状細胞、気道上皮とどのように相互作用して 2 型炎症を開始･増幅･収束させますか。

回答

好酸球性喘息では、気道上皮からの警報シグナルが出発点になることが多いです。アレルゲン、ウイルス、汚染物質、機械刺激で TSLP、IL-33、IL-25 が増え、樹状細胞はアレルゲン特異的 Th2 応答を促します。ILC2 も活性化され、Th2/ILC2 由来 IL-5 が好酸球を維持し、IL-4/IL-13 は IgE、粘液、eotaxin を誘導します。肥満細胞は IgE 刺激でヒスタミン、PGD2、システイニルロイコトリエンを即時放出し、患者によっては好塩基球も IL-4 を補います。

IL-5/eotaxin 系で動員された好酸球は主要塩基性蛋白、好酸球ペルオキシダーゼ、ロイコトリエン、サイトカイン、TGF-βを放出し、上皮障害、気道過敏、リモデリングを促します。IL-4/IL-13 はマクロファージを 2 型･修復関連状態へ変え、マクロファージ由来ケ

モカインや増殖因子が局所ネットワークを補強します。樹状細胞は抗原提示を継続し、反応を反復可能な適応免疫へ結びつけます。

収束には上皮アラーミン低下、IL-5 などの生存シグナル減少、好酸球アポトーシス、マクロファージのエフェロサイトーシスが必要です。IL-10、TGF-β、収束性脂質が修復を支えます。ただし TGF-βが長く高いと、好酸球数が下がってもリモデリングが残り得ます。

Q17

質問

反復性尿路感染において、過去の感染は、訓練免疫、免疫寛容、または組織リモデリングを介して膀胱粘膜の好中球および単球 / マクロファージ応答をどのように再構成し得ますか。また、それぞれの機序を支持するエビデンスはどの程度確立されていますか。

回答

既往の尿路感染は、訓練免疫、免疫寛容、組織リモデリングの少なくとも 3 つで次回応答を変え得ますが、エビデンスの強さは同じではありません。訓練免疫は、初回刺激が単球や骨髄前駆細胞にエピジェネティック･代謝的変化を残し、再感染時のサイトカイン･抗菌反応を速くする概念です。先天免疫一般では確立した概念ですが、ヒト反復性 UTI で膀胱単球/マクロファージや好中球前駆細胞の訓練が再発を防ぐという直接証拠はまだ限定的で、主に前臨床的です。

反復 TLR 刺激では逆に寛容が起こり、TNF や IL-1 の再刺激応答が弱くなりながら一部抗菌機能が残ることがあります。これは膀胱障害を減らす可能性がある一方、細菌持続を許す可能性もあります。UTI 特異的なヒト機序データは十分ではありません。

組織リモデリングは膀胱局所として最も分かりやすい機序です。既往感染は尿路上皮分化、バリア、間質･神経シグナル、常在免疫細胞構成を変え、細胞内細菌リザーバーや異常修復も再発閾値を変えます。成熟好中球は短命なので、長期記憶は前駆細胞、常在マクロファージ、あるいは組織環境に宿る可能性が高いです。総じてリモデリングの組織特異的根拠が比較的強く、訓練免疫･寛容は有望ですがヒト UTI の主因としては未確立です。

Q18

質問

膀胱がんにおいて、腫瘍関連好中球と腫瘍関連マクロファージは粘膜腫瘍微小環境内でどのように相互作用し、抗原提示、T 細胞活性、血管新生、浸潤、免疫療法への反応に影響しますか。

回答

膀胱がんの腫瘍関連マクロファージ（TAM）と腫瘍関連好中球（TAN）は非常に可塑的です。腫瘍･間質は CCL2/CSF1、CXCL 系シグナルでこれらを呼び込み、低酸素、乳酸、TGF-β、IL-10 などで機能を再設定します。免疫抑制性 TAM は IL-10、TGF-β、PD-L1、アルギニン代謝、アデノシン系を介して抗原提示や T 細胞機能を弱めます。TAN も一部では MDSC 様機能を獲得し、ROS、アルギナーゼ、プロテアーゼなどで T 細胞を抑制します。

両者は血管新生と浸潤にも関わります。TAM 由来 VEGF、MMP、増殖因子は血管と細胞外基質を変え、TAN のプロテアーゼや NET も基質を変化させ、状況によって腫瘍浸潤･播種を助けます。ケモカインや炎症メディエーターを介して相互に補強するため、一方だけを遮断するともう一方が代償する可能性があります。

免疫チェックポイント阻害では、骨髄系抑制が強い腫瘍は反応しにくい傾向があります。一方、膀胱内 BCG は局所の好中球･マクロファージを含む強い先天免疫活性化が抗腫瘍免疫に有用であることを示しています。したがって目標は骨髄系細胞の全面抑制ではなく選択的再プログラムです。ヒトでは TAM/TAN 状態と予後の関連は示されますが、具体的な亜集団と因果経路は腫瘍間で不均一です。

Q19

質問

潰瘍性大腸炎において、病原性の好中球動員または好中球細胞外トラップ関連障害を抑えつつ、抗微生物防御とマクロファージによる修復を可能な限り温存するには、どのような機序的に妥当な戦略が考えられますか。予想される利点、トレードオフ、エビデンスの不足を説明してください。

回答

潰瘍性大腸炎では、好中球を完全に除くより、最も組織障害性の高い部分だけを弱める考え方が合理的です。第一は CXCR2/CXCL8 系などの遊走シグナルを部分的に抑え、陰窩への過剰集積とプロテアーゼ･活性酸素傷害を減らす方法です。ただし強い遮断は細菌排除と創傷防御を損なうため、局所投与や短期投与が望ましい可能性があります。

第二は NET 関連障害を狙う方法です。DNase は DNA 骨格を分解し、PAD4、好中球エラスターゼ、MPO の阻害は NET 形成や毒性を減らす可能性があります。細胞外ヒストンやプロテアーゼ曝露、マクロファージへの DAMP 刺激を減らせる点が利点です。一方、NET は微生物封じ込めに寄与する場合があり、DNase で DNA を除いても結合したプロテアーゼ･ヒストンまで中和できるとは限りません。

第三は収束を早める戦略で、マクロファージのエフェロサイトーシスを高める、あるいは炎症収束性脂質を利用して、初期抗菌作用を残しつつ好中球滞在時間を短くする方法です。上皮バリア修復も再動員刺激を減らします。

これらの多くは UC で臨床的に確立した標準治療ではなく、機序的･前臨床的段階です。NET 優位患者の同定、安全な抑制度、感染リスク、マクロファージ修復を保てるかが主要なエビデンスギャップです。

Q20

質問

炎症性腸疾患において、腸管マクロファージと顆粒球の代謝的またはエピジェネティックなリプログラミングを粘膜恒常性の回復にどのように利用できるでしょうか。また、効果消失や意図しない免疫抑制をもたらし得る機序は何ですか。

回答

代謝･エピジェネティック再プログラムは、骨髄系細胞を減らすのではなく、持続炎症型から恒常性･修復型へ機能を変える考え方です。IBDの炎症性マクロファージでは、強い解糖、HIF-1α、コハク酸、mTOR、炎症性クロマチンプログラムがIL-1β/TNF産生を支えます。AMPKやミトコンドリア酸化、itaconate様の抗炎症代謝、特定のエピジェネティック制御を利用すれば、エフェロサイトーシス、IL-10、修復へ寄せられる可能性があります。好中球などでは、過剰な解糖、ROS、NETosisを抑えて組織傷害を減らすことが想定されます。

問題は選択性です。同じ代謝経路は抗菌にも必要です。解糖、NADPHオキシダーゼ、インフラマソーム、クロマチン開放を過度に抑えると、貪食、酸化殺菌、サイトカイン応答、感染防御が落ちます。そのため腸管局所、短期間、炎症性骨髄系サブセットに選択的な送達が全身抑制より望まれます。

効果が失われる理由として、骨髄から新しい細胞が常時補充されること、微生物･サイトカイン刺激がプログラムを上書きすること、平行経路が代償することが挙げられます。エピジェネティック状態も固定ではありません。意図しない免疫抑制は感染、創傷治癒不良、細菌移行の制御不全として現れ得ます。現時点では標準IBD治療というより、バイオマーカーと送達技術を要するトランスレーショナル戦略です。

Q21

質問

2型サイトカインシグナルを変化させる生物学的製剤は、単に好酸球数を減らすだけでなく、気道粘膜の好酸球–肥満細胞–マクロファージ–樹状細胞ネットワークをどのように再構成し得ますか。

回答

2型サイトカインを標的とする生物学的製剤は、単に好酸球数を減らすだけでなく、気道骨髄系ネットワーク全体を変えます。抗IL-5/抗IL-5受容体療法では好酸球の成熟、生存、組織動員が減り、同時にロイコトリエン、顆粒蛋白、IL-4/IL-13、TGF-β、DAMPの放出も減ります。その結果、マクロファージや樹状細胞への二次刺激、上皮傷害による炎症の再増幅も低下します。

IL-4/IL-13シグナル遮断はさらに広い作用を持ち、eotaxin、粘液、IgE関連増幅、IL-4/IL-13依存性マクロファージ機能を弱め、樹状細胞とT細胞のクロストークも2型方向へ偏りにくくします。より上流のTSLP、また状況に応じたIL-33関連経路の遮断では、樹状細胞、ILC2、肥満細胞、下流顆粒球を同時に抑えられる可能性があります。

好酸球標的療法で肥満細胞数そのものが大きく減らなくても、周囲のメディエーター環境と活性化閾値は変化します。上皮傷害が減ればマクロファージの修復機能も正常化し得ます。一方、2型サイトカインは寄生虫防御や一部のバリア修復にも関与するため、完全抑制は生物学的に中立ではありません。治療反応の差は、患者の主回路が好酸球、IL-4/13、上皮アラーミンのどこにあるかを反映します。

Q22

質問

好中球性喘息において、抗菌防御を維持しながら好中球–マクロファージ炎症回路を遮断するために、どのような骨髄系細胞標的戦略が考えられますか。また、提案されている機序のうち、臨床的に確立しているものと前臨床段階にあるものを区別してください。

回答

好中球性喘息では、抗菌防御を残しながら過剰な動員･活性化だけを切ることが目標です。臨床的に比較的確立しているのは、選択された持続性重症喘息患者でのマクロライド使用です。抗菌作用に加えて、上皮･骨髄系細胞のサイトカイン産生を調整する作用が考えられます。ただし純粋な好中球標的薬ではなく、耐性菌、QT、薬物相互作用、微生物叢への影響というトレードオフがあります。

より直接的な手段はまだ確立度が低いです。CXCR2阻害で好中球遊走を減らせますが、臨床利益は一貫せず、強い遮断は感染リスクを上げる可能性があります。IL-1β/NLRP3、GM-CSF、LTB4、好中球エラスターゼ、NET/PAD4、マクロファージ炎症代謝などは機序的･前臨床的根拠がありますが、好中球性喘息の標準治療ではありません。気管支拡張症や慢性感染が併存する場合は、感染源を治療して持続刺激を減らすことも重要です。

抗菌防御を保つには、全身性の好中球除去ではなく、部分的、表現型に基づく、可能なら気道局所の治療が理想です。現時点で、好中球–マクロファージ回路だけを選択的に遮断し、抗菌能を確実に温存できる広く確立した薬剤はありません。

Q23

質問

訓練免疫または粘膜ワクチンのアプローチによって、単球 / マクロファージと好中球を有益にリプログラムし、反復性尿路感染を減らすことは可能でしょうか。想定される機序、組織特異的な潜在的リスク、現時点のエビデンスの限界を説明してください。

回答

訓練免疫と粘膜ワクチンはいずれも反復性UTIを減らす可能性がありますが、主な機序は異なります。粘膜ワクチンは病原体特異的IgAなどの抗体やT細胞応答を高め、膀胱へ到達･侵入する菌量を減らすことで、毎回の強い好中球炎症を減らす考え方です。訓練免疫はパターン認識受容体刺激によって単球や骨髄前駆細胞にエピジェネティック･代謝的変化を残し、次回のマクロファージ･好中球応答を速く、より効率的にすることを狙います。

利点は早期の細菌封じ込め、貪食効率、マクロファージ主導の収束への移行を改善できる可能性です。しかし強い訓練が必ず有益とは限りません。膀胱で過剰な好中球動員、尿路上皮障害、疼痛、線維化を起こすリスクがあります。反対に寛容が強すぎればサイトカイン応答が鈍り、上行感染を許す可能性があります。成熟好中球は短命なので、持続的効果は前駆細胞、常在マクロファージ、上皮･間質の組織記憶に由来すると考える方が自然です。

現在の臨床エビデンスは研究規模、製剤、投与経路、菌種が不均一で、膀胱粘膜を直接解析した機序研究も少ないです。免疫予防法の一部には有望な臨床信号がありますが、「訓練された単球/マクロファージや好中球が効果の直接原因」とする証拠はまだ弱い段階です。

Q24
質問
腫瘍関連マクロファージと好中球を選択的にリプログラムすることで、保護的な尿路粘膜免疫への影響を最小限にしつつ、膀胱がんの抗腫瘍免疫をどのように高められるでしょうか。機序的アプローチと主要なトランスレーショナル上の障壁を比較してください。

回答
膀胱がんでは、マクロファージや好中球の全面除去より選択的再プログラムが望まれます。これらは正常尿路の抗菌防御にも必要だからです。TAM に対しては、CCL2/CCR2 や CSF1/CSF1R を介した免疫抑制性細胞の流入･生存を減らす方法と、CD40、TLR/STING、PI3Kγ、代謝系などを利用して抗原提示と T 細胞支援能力を回復させる方法があります。再プログラムは貪食機能を残しやすい一方、過剰活性化は膀胱炎や全身炎症を起こし得ます。

TAN では CXCR2 系を抑えて腫瘍促進型細胞の流入を減らす、TGF-β、アルギニン/アデノシン代謝、NET/PAD4、好中球プロテアーゼを標的にして T 細胞抑制と浸潤を減らす方法が考えられます。ただし抗菌防御と創傷治癒低下が問題です。膀胱内局所投与は腫瘍への曝露を高めながら全身骨髄系抑制を減らせる点で魅力的です。BCG は局所先天免疫活性化の有効性を示す実例ですが、選択的 TAM/TAN 再プログラム薬ではありません。

主なトランスレーショナル障壁は、骨髄系細胞の可塑性、悪い腫瘍関連細胞だけを識別する固有マーカーの不足、骨髄からの迅速な補充、腫瘍間不均一性、代償性経路です。現実的には、バイオマーカーで患者を選び、局所投与とチェックポイント阻害などを組み合わせる戦略が有望です。

Simplified Chinese (SC)

Anonymous Model E ｜ 简体中文 ｜ Informal Response Package
Anonymous Model E
简体中文

Q01
问题
在健康肠黏膜中，中性粒细胞如何在与驻留巨噬细胞和树突状细胞相互作用的同时参与屏障防御？哪些机制通常限制中性粒细胞介导的组织损伤？

回答
健康肠黏膜并不会长期聚集大量中性粒细胞。正常情况下，它们主要停留在血循环中；只有当上皮屏障出现微小破口、细菌穿越黏液层，或驻留巨噬细胞等哨兵细胞识别到危险信号时，才会被迅速募集。CXCL1/CXCL2、人体中的 CXCL8，以及补体和脂质介质共同引导中性粒细胞迁移。到达局部后，中性粒细胞通过吞噬、活性氧、颗粒酶和抗菌蛋白杀灭细菌或真菌，刺激较强时也可形成胞外诱捕网。驻留巨噬细胞一方面产生募集信号，另一方面负责吞噬病原体和后续清除细胞碎片；树突状细胞则更多承担抗原取样和连接适应性免疫的作用。

中性粒细胞的杀菌工具同样可能伤害上皮，因此健康黏膜存在多层“刹车”。黏液、紧密连接、抗菌肽和快速上皮更新先把微生物限制在腔面；肠道巨噬细胞对无害共生菌相对低反应，并持续产生 IL-10 等调节因子。病原负荷下降后，中性粒细胞进入凋亡，随后被巨噬细胞进行凋亡细胞清除，这会推动 IL-10、TGF-β和组织修复程序，同时减少新的中性粒细胞募集。促炎症消退脂质介质、抗氧化系统、抗蛋白酶以及局部引流也可降低弹性蛋白酶、MPO 产物和胞外 DNA 造成的旁观者损伤。

临床上最关键的是“短、准、能停”：短暂而局限的中性粒细胞反应是屏障防御的一部分；如果募集持续、NET 或蛋白酶过多、凋亡细胞清除失败，就会从保护转为黏膜损伤。

Q02
问题
在健康肠黏膜中，嗜酸性粒细胞如何参与维持稳态并与巨噬细胞或树突状细胞进行通讯？这些作用与炎症状态下嗜酸性粒细胞的功能有何不同？

回答
嗜酸性粒细胞本来就是肠固有层的常驻细胞，因此在肠道看到它们并不等同于炎症。稳态下，IL-5 以及通过 CCR3 起作用的 eotaxin 类趋化因子维持其存活和组织定位。它们参与屏障监视、局部免疫调节和组织更新，还可通过细胞因子、脂质介质和生长因子影响上皮、基质细胞及 IgA 相关反应。与巨噬细胞和树突状细胞的交流通常较温和：嗜酸性粒细胞来源的 IL-4/IL-13 可使部分巨噬细胞偏向修复和组织重塑功能，而巨噬细胞、树突状细胞释放的细胞因子又会影响嗜酸性粒细胞的存活和活化。其直接改变树突状细胞成熟或抗原呈递的程度，则更依赖具体环境。

炎症时情况明显不同。活化的嗜酸性粒细胞会释放主要碱性蛋白、嗜酸性粒细胞阳离子蛋白、嗜酸性粒细胞过氧化物酶、白三烯、细胞因子和活性氧。这些介质可增加上皮通透性、促进血管和神经反应，并进一步激活其他髓系细胞。在 2 型炎症中，它们还会加强 IL-4/IL-13 主导的巨噬细胞程序；在混合型炎症中，也可与中性粒细胞和炎性单核细胞同时出现。

因此，稳态嗜酸性粒细胞更接近“组织适应型的调节/修复细胞”；炎症嗜酸性粒细胞则表现为更强的脱颗粒和细胞因子输出。两者并非截然分开，人类肠道疾病中的表型差异很大。

Q03

问题

在健康呼吸道黏膜以及急性微生物攻击期间，中性粒细胞如何与肺泡或间质巨噬细胞及树突状细胞协同，在发挥宿主防御作用的同时限制对上皮的旁观者损伤？

回答

健康气道中，肺泡巨噬细胞是肺泡腔最主要的吞噬哨兵，平时需要在不断接触吸入颗粒的情况下维持低炎症状态。急性微生物刺激出现后，肺泡或间质巨噬细胞以及气道上皮的模式识别受体被激活，产生 IL-1、TNF、CXCL 类趋化因子、G-CSF 等，迅速招募中性粒细胞。中性粒细胞通过吞噬、氧化爆发、颗粒酶和必要时的 NET 形成进行快速抗菌/抗真菌防御。树突状细胞负责摄取抗原并迁移至淋巴结，决定后续适应性免疫的强度和方向。

真正保护肺组织的关键是“及时关停”。病原负荷下降后，中性粒细胞凋亡，随后被巨噬细胞清除；这一过程会降低 IL-1/TNF 样信号，并增强 IL-10、TGF-β和促炎症消退脂质介质。α1-抗胰蛋白酶、分泌型白细胞蛋白酶抑制剂等抗蛋白酶，局部抗氧化系统、DNase、黏液纤毛清除和上皮修复，可减少弹性蛋白酶、MPO 产物及胞外 DNA 对上皮的伤害。

如果病原没有被清除，或巨噬细胞的凋亡细胞清除能力下降，这个协同防御回路就可能变成持续炎症回路。临床上在重症肺炎、COPD 急性加重、支气管扩张和部分重症哮喘中都可看到这种“既抗菌、又可能伤肺”的双重性。

Q04

问题

在稳态条件和 2 型炎症期间，嗜酸性粒细胞如何在气道黏膜中与巨噬细胞和树突状细胞相互作用？

回答

稳态气道中的嗜酸性粒细胞数量通常不高，与巨噬细胞和树突状细胞的互作也比较有限。此时肺泡巨噬细胞主要负责清除颗粒和抑制不必要的炎症，树突状细胞持续进行低水平抗原取样。少量嗜酸性粒细胞可能参与组织监视和修复，但在健康气道中，并没有像肠道那样明确的、占主导地位的嗜酸性粒细胞稳态网络。

进入 2 型炎症后，网络迅速放大。受损或接触过敏原的上皮释放 TSLP、IL-33 和 IL-25，激活树突状细胞和 ILC2；Th2/ILC2 产生 IL-5 促进嗜酸性粒细胞生成和存活，IL-4/IL-13 诱导 eotaxin、黏液和屏障改变。募集的嗜酸性粒细胞释放白三烯、颗粒蛋白、细胞因子和生长因子，其 IL-4/IL-13 可加强偏修复/2 型的巨噬细胞状态；巨噬细胞又可产生 CCL17、CCL22 等，帮助维持 Th2 细胞环境。树突状细胞通过持续呈递过敏原，使这种反应获得适应性免疫“记忆”。

巨噬细胞也参与消退，例如清除凋亡嗜酸性粒细胞并产生调节介质。所以，这套网络在短期内可服务于修复，但若嗜酸性粒细胞持续脱颗粒、黏液分泌和气道高反应性持续存在，就会变成致病回路。不同哮喘表型中，嗜酸性粒细胞对树突状细胞的直接影响强弱并不一致。

Q05

问题

在尿路黏膜中，早期抗菌防御期间，中性粒细胞如何与单核细胞或巨噬细胞及树突状细胞协同？哪些机制可防止过度组织损伤？

回答

尿路早期抗菌防御首先依赖尿路上皮和局部髓系细胞对细菌成分的识别。上皮细胞、巨噬细胞和树突状细胞可释放 IL-1、IL-6 及 CXCL 类趋化因子，快速把中性粒细胞从血液招募到黏膜。中性粒细胞通常是最早、最强的效应细胞，通过跨上皮迁移、吞噬、活性氧、颗粒抗菌蛋白以及必要时的 NET 形成来清除细菌。单核细胞/巨噬细胞同时参与吞噬和细胞因子产生，并在稍后阶段负责清除死亡中性粒细胞；树突状细胞更偏向抗原取样和连接适应性免疫。

尿流冲刷和上皮脱落本身已经有助于清除细菌，因此如果中性粒细胞反应过强，反而会增加损伤。弹性蛋白酶等蛋白酶、活性氧和 NET 成分可破坏尿路上皮，使通透性增加。正常情况下，快速降低细菌负荷、中性粒细胞凋亡、巨噬细胞的凋亡细胞清除、IL-10/TGF-β主导的消退信号、局部抗氧化/抗蛋白酶系统以及尿路上皮再生共同限制组织损伤。

从临床角度看，募集不足可能导致细菌持续甚至上行感染；募集过久则会加重尿痛、血尿和黏膜损伤。巨噬细胞的价值不仅在“帮忙杀菌”，还在于及时把炎症切换到修复阶段。

Q06

问题

在膀胱或下泌尿生殖道黏膜中，肥大细胞释放的介质如何影响巨噬细胞和树突状细胞的功能？这一网络如何同时参与宿主防御和炎症相关症状？

回答

膀胱和下泌尿生殖道黏膜中的肥大细胞可被微生物成分、IgE 依赖信号、组织损伤、补体和神经肽激活，随后释放组胺、胰蛋白酶样蛋白酶、TNF、前列腺素、白三烯及多种细胞因子。急性阶段，这些介质提高血管通透性和局部血流，并促进中性粒细胞、单核细胞进入组织，因此对感染防御有一定帮助。

巨噬细胞和树突状细胞可通过细胞因子受体、组胺受体、蛋白酶激活受体及脂质介质受体感受肥大细胞信号。TNF 和部分脂质介质可增强树突状细胞成熟和炎症型巨噬细胞活性，帮助抗原呈递和抗菌；反过来，巨噬细胞产生的 IL-1、TNF 和危险信号也可进一步刺激肥大细胞。与此同时，肥大细胞并非只会“促炎”，某些情况下也能释放调节介质，使炎症重新分配或减弱。

症状往往来自同一套网络的另一面。组胺、胰蛋白酶、前列腺素、白三烯及 NGF 相关信号可敏化感觉神经，引起尿急、疼痛和尿频；血管和上皮通透性增加又让尿液中的刺激物更容易接触黏膜。若这种正反馈持续，就可能在没有活动性感染时仍维持症状。不同疾病——感染、间质性膀胱炎/膀胱疼痛综合征等——主导通路可能并不相同。

Q07

问题

在急性肠黏膜炎症及其后的炎症消退阶段，中性粒细胞与巨噬细胞如何通过募集信号、细胞因子、凋亡细胞清除、脂质介质和组织修复程序相互影响？

回答

急性肠黏膜炎症开始时，上皮和髓系细胞产生 CXCL 趋化因子，补体和 LTB4 等脂质介质共同驱动中性粒细胞快速进入病灶。中性粒细胞杀菌的同时也会释放 IL-1 相关信号、蛋白酶、活性氧和危险分子，进一步招募 CCR2 依赖的单核细胞。单核细胞进入组织后形成炎性巨噬细胞，产生 TNF、IL-1β等细胞因子；如果微生物和组织危险信号持续存在，就会继续推动中性粒细胞募集。

炎症消退不是“细胞自然消失”，而是主动转换。病原负荷下降后，中性粒细胞停止接受存活信号并进入凋亡，膜表面暴露磷脂酰丝氨酸。巨噬细胞通过 MerTK 等受体相关途径进行凋亡细胞清除。这个过程会抑制新的促炎细胞因子，同时增加 IL-10、TGF-β、生长因子和修复程序。脂质介质也可从以白三烯为主转向脂氧素、resolvin、protectin、maresin 等促炎症消退介质，从而减少新的中性粒细胞进入。

随后巨噬细胞支持上皮再生、基质重塑和屏障恢复。这里不宜简单概括为“M1 变 M2”，因为肠道巨噬细胞状态更复杂。如果中性粒细胞不易凋亡、巨噬细胞清除能力受损，或病原持续刺激，炎症就会延长。也就是说，中性粒细胞既能制造损伤，也能通过“被正确清除”向巨噬细胞发出进入修复期的信号。

Q08

问题

嗜酸性粒细胞来源的介质如何改变肠黏膜巨噬细胞的功能状态和树突状细胞的激活？这些相互作用如何推动免疫耐受、组织修复与炎症之间的平衡发生变化？

回答

嗜酸性粒细胞可以通过细胞因子、脂质介质、颗粒蛋白和生长因子改变肠黏膜巨噬细胞的功能。嗜酸性粒细胞产生的 IL-4 和 IL-13 可使部分巨噬细胞偏向组织修复、基质处理和 2 型免疫相关程序；TGF-β等生长因子在短期内有助于上皮修复，但如果持续过高，也可能推动纤维化。嗜酸性粒细胞的脂质介质可改变血管和髓系细胞活性；而强烈脱颗粒时，颗粒蛋白和氧化产物本身又可作为危险信号。

它们对树突状细胞的影响更依赖具体情境。嗜酸性粒细胞来源的细胞因子可改变树突状细胞成熟、迁移和抗原呈递时的细胞因子环境。在稳态或轻度刺激下，这种作用可能帮助维持对食物和共生菌的“可控反应”；在强烈 2 型或混合炎症中，则可能增加 T 细胞活化并促进更多粒细胞募集。嗜酸性粒细胞在某些实验条件下可呈递抗原，但在人类肠黏膜中通常不是主要的专业抗原呈递细胞。

因此，结果取决于激活强度和时序：适度信号偏向耐受和修复；持续 IL-4/IL-13/TGF-β可能造成重塑；大量脱颗粒则更容易造成上皮损伤和树突状细胞激活。描述巨噬细胞时采用连续功能谱，比简单的“M1/M2 二分法”更准确。

Q09

问题

在中性粒细胞性气道炎症中，中性粒细胞与巨噬细胞如何通过细胞因子、脂质介质、中性粒细胞胞外诱捕网、凋亡细胞清除和代谢信号相互影响？这种串扰如何决定炎症持续还是消退？

回答

中性粒细胞性气道炎症中，巨噬细胞和中性粒细胞很容易形成正反馈。巨噬细胞和气道上皮释放 IL-1β、TNF、GM-CSF、CXCL8 等，使中性粒细胞持续进入并延长存活；中性粒细胞则释放 LTB4、蛋白酶、活性氧和其他炎症介质，再次刺激巨噬细胞和上皮。NET 在控制微生物时有价值，但胞外 DNA、组蛋白、弹性蛋白酶和 MPO 产物也可激活危险感知和炎症小体相关通路，增加黏液分泌及上皮损伤。

代谢环境会决定这个回路是否容易“熄火”。中性粒细胞高度依赖糖酵解，因此即使在缺氧、黏稠的痰液中仍可工作；但高乳酸、琥珀酸、低氧和氧化应激也会改变巨噬细胞 HIF-1α及线粒体信号，使 IL-1β等促炎介质持续。相反，IL-10、促炎症消退脂质和部分抗炎代谢程序可推动巨噬细胞退出炎症状态。

真正的消退依赖中性粒细胞凋亡和巨噬细胞有效的凋亡细胞清除。清除后，巨噬细胞会减少促炎细胞因子并增加 TGF-β、IL-10 和脂质介质转换。若清除失败、NET 负荷过高或细菌/病毒信号持续，炎症就容易长期存在。COPD、支气管扩张、重症感染和部分中性粒细胞性哮喘都可出现这一模式，但各机制所占比重不同。

Q10
问题
在过敏性气道炎症中，肥大细胞和嗜酸性粒细胞（在相关情境下包括嗜碱性粒细胞）如何与树突状细胞和巨噬细胞相互作用，从而放大或限制 2 型免疫反应？

回答
过敏性气道炎症由多个细胞共同构成 2 型免疫网络。上皮接触过敏原后释放 TSLP、IL-33 和 IL-25，树突状细胞由此促进 Th2 分化，ILC2 也产生 IL-5 和 IL-13。肥大细胞经 IgE 交联后迅速释放组胺、PGD2、白三烯和细胞因子；某些情况下，嗜碱性粒细胞可提供较早的 IL-4 和脂质介质。IL-5 和 eotaxin 通路共同招募并维持嗜酸性粒细胞。

嗜酸性粒细胞通过半胱氨酰白三烯、颗粒蛋白、细胞因子和生长因子继续放大炎症。其 IL-4/IL-13 可加强巨噬细胞的 2 型/修复相关程序，巨噬细胞又能产生 CCL17、CCL22 等趋化因子，帮助 Th2 细胞维持在气道。树突状细胞不断接收肥大细胞和嗜酸性粒细胞的介质信号，同时持续呈递过敏原，因此使反应从一次性炎症变成可反复启动的适应性免疫反应。

限制这套网络主要依靠巨噬细胞清除凋亡嗜酸性粒细胞、IL-10/TGF-β、调节性 T 细胞和促炎症消退脂质介质。若这些“刹车”不足，肥大细胞激活、嗜酸性粒细胞脱颗粒和巨噬细胞重塑信号就形成持续正反馈。嗜碱性粒细胞数量通常更少，其重要性也更依赖具体表型。

Q11
问题
在急性细菌性尿路感染期间，募集到局部的中性粒细胞和单核细胞来源巨噬细胞如何在病原体清除、细胞因子产生、凋亡细胞清除和黏膜修复过程中协同或竞争？

回答
急性细菌性尿路感染中，中性粒细胞到达最快，负责高强度、短时的杀菌；募集的单核细胞及其分化形成的巨噬细胞则承担更广泛、随时间变化的任务。两者都能吞噬细菌，但中性粒细胞更擅长快速氧化爆发、脱颗粒和短寿命抗菌。单核/巨噬细胞产生 IL-1、TNF 和趋化因子，维持中性粒细胞募集，也可直接吞噬病原体和处理组织碎片。因此早期二者多是协同关系：巨噬细胞“叫人”，中性粒细胞迅速降低细菌负荷。

竞争或冲突主要发生在反应过强时。大量中性粒细胞消耗氧和营养，并释放蛋白酶、活性氧或 NET，可能造成尿路上皮损伤；这些损伤信号又使巨噬细胞继续保持炎症状态。反过来，巨噬细胞通过吞噬凋亡中性粒细胞主动结束炎症，所以如果过早抑制中性粒细胞会影响杀菌，而如果死亡中性粒细胞迟迟不能清除，又会加重组织损伤。

细菌控制后，巨噬细胞在消退和修复阶段更关键：清除凋亡细胞，释放 IL-10、TGF-β和生长因子，促进尿路上皮再生。换句话说，早期强调共同抗菌，后期则应转为“巨噬细胞清场和修复”。

Q12
问题
在慢性膀胱黏膜炎症中，肥大细胞与巨噬细胞之间的串扰可能如何改变感觉神经信号、血管通透性、上皮屏障功能以及炎症的持续或消退？

回答
慢性膀胱黏膜炎症中，肥大细胞和巨噬细胞可共同放大炎症、血管和感觉神经信号。肥大细胞释放组胺、胰蛋白酶样蛋白酶、前列腺素、白三烯、TNF 和生长因子；组胺和脂质介质增加血管通透性，蛋白酶可激活 PAR 类受体，同时这些介质可敏化 TRPV1 等感觉神经通路。局部巨噬细胞又可释放 IL-1β、TNF、趋化因子、PGE2 和 NGF 相关信号，使疼痛、尿急进一步增强。

屏障损伤会让回路更稳定。血管渗漏带来更多血浆蛋白和炎症细胞；受损尿路上皮对尿液中的溶质、微生物成分和损伤相关分子更敏感。上述信号再次刺激肥大细胞和巨噬细胞，形成正反馈。持续的巨噬细胞促炎状态会延缓上皮修复，肥大细胞蛋白酶也可能影响紧密连接和细胞外基质。

如果最初刺激被去除，巨噬细胞转向凋亡细胞清除和修复，并增加 IL-10、TGF-β和促炎症消退脂质，肥大细胞激活下降，屏障恢复，神经和血管刺激就可减弱。需要注意的是，在人类慢性膀胱疼痛相关疾病中，肥大细胞增多并非所有患者都存在，因此这一机制更适合解释部分表型，而不是统一解释全部病例。

Q13
问题
在溃疡性结肠炎中，中性粒细胞募集、中性粒细胞胞外诱捕网形成、中性粒细胞代谢以及中性粒细胞-巨噬细胞串扰如何分别促进黏膜损伤或炎症消退？

回答
溃疡性结肠炎活动期最典型的病理之一就是中性粒细胞进入隐窝，形成隐窝炎和隐窝脓肿。上皮和髓系细胞产生 CXCL8 等趋化因子，加上补体和脂质介质，使中性粒细胞大量募集。早期这有保护意义，因为屏障破损后需要迅速吞噬越过上皮的细菌；问题在于反应持续时，弹性蛋白酶、MPO 产物、活性氧等会直接损伤上皮，使屏障缺口继续扩大。

NET 形成同样具有双重性。NET 可限制微生物扩散，但胞外 DNA、组蛋白、蛋白酶和氧化酶暴露在组织中时会造成细胞毒性，并可能通过 TLR、炎症小体等危险感知通路刺激巨噬细胞持续产生 IL-1β和 TNF。中性粒细胞高度依赖糖酵解，因此在缺氧隐窝中仍能保持活性；与此同时，大量耗氧和代谢产物也会进一步改变局部低氧和巨噬细胞代谢状态。

炎症消退依赖中性粒细胞凋亡后被巨噬细胞及时清除。有效的凋亡细胞清除会降低促炎因子，增加 IL-10、TGF-β、促炎症消退脂质和上皮修复。临床上“黏膜中性粒细胞多”与活动性疾病关系很明确；至于某一种 NET 或代谢通路在人类患者中究竟占多大因果比例，目前证据还没有同样扎实。

Q14

问题

在克罗恩病中，中性粒细胞、单核细胞来源巨噬细胞和树突状细胞之间失调的相互作用如何改变微生物处理、抗原呈递、炎症持续以及上皮修复？

回答

克罗恩病可同时存在“微生物处理不够有效”和“后续炎症过强”两个问题。中性粒细胞本应快速清除穿过黏膜的细菌，但在部分患者中，募集的时序或杀菌效率可能不理想。病原体或其产物持续存在后，CCR2 依赖的单核细胞进入组织并形成炎性巨噬细胞，产生 TNF、IL-1β、IL-6、IL-23 等，支持 Th1/Th17 反应并继续招募髓系细胞。

树突状细胞持续摄取微生物和食物抗原，在炎症细胞因子环境下提高共刺激和抗原呈递。与克罗恩病有关的 NOD2、ATG16L1 等通路可影响不同细胞的微生物识别或自噬处理。这样就可能出现一个不利组合：细菌没有被迅速、彻底清除，但抗原呈递和炎症信号却长期存在，最终形成慢性免疫激活。

这些细胞同样具有修复作用。巨噬细胞可清除凋亡中性粒细胞，产生生长因子和 TGF-β；树突状细胞在非炎症环境下也可促进耐受。克罗恩病中，持续微生物刺激、细胞因子、缺氧和基质损伤会阻碍这种转换；而过强的修复/TGF-β信号又可能参与纤维化和狭窄。因此核心不是单纯“某类细胞太多”，而是微生物处理、抗原呈递、炎症关停和上皮修复之间失去协调。

Q15

问题

在重度中性粒细胞性哮喘中，中性粒细胞、巨噬细胞和树突状细胞如何形成自我维持的炎症回路？中性粒细胞胞外诱捕网、炎症小体信号、微生物信号以及糖皮质激素抵抗可能分别发挥什么作用？

回答

重度中性粒细胞性哮喘可由气道上皮、巨噬细胞、树突状细胞、Th17 样反应和中性粒细胞共同构成自我维持回路。上皮和巨噬细胞产生 IL-1β、TNF、IL-6、GM-CSF 和 CXCL8，促进中性粒细胞募集并延长其存活。树突状细胞接触微生物产物和炎症细胞因子后，可支持 IL-17 相关 T 细胞反应；IL-17 又刺激上皮产生更多中性粒细胞趋化因子。中性粒细胞释放弹性蛋白酶、活性氧和炎症介质，再次破坏上皮并制造新的危险信号。

NET 可能进一步放大回路。胞外 DNA、组蛋白和中性粒细胞酶可刺激上皮和巨噬细胞，并促进 NLRP3/IL-1β等炎症小体相关信号。慢性细菌定植、气道微生态改变或反复病毒感染，会持续激活模式识别受体，使炎症更难停止。

糖皮质激素抵抗通常不是单一机制造成的。氧化应激、p38-MAPK 相关信号、糖皮质激素受体功能改变、持续的 IL-17/IL-1 通路，以及中性粒细胞本身对激素较不敏感和存活延长，都可能参与。不同患者并非都以 NET 或炎症小体为主导，因此临床上需要表型分层，而不能把所有中性粒细胞性哮喘归为同一种机制。

Q16

问题

在嗜酸性粒细胞性哮喘中，嗜酸性粒细胞、肥大细胞以及在相关情境下的嗜碱性粒细胞，如何与巨噬细胞、树突状细胞和气道上皮相互作用，从而启动、放大并最终促使 2 型炎症消退？

回答

嗜酸性粒细胞性哮喘通常从气道上皮的“报警”开始。过敏原、病毒、污染物或机械刺激可诱导 TSLP、IL-33、IL-25。树突状细胞在这些信号下促进过敏原特异性 Th2 反应，ILC2 也被激活；Th2 和 ILC2 产生 IL-5，IL-4/IL-13 则促进 IgE、黏液和 eotaxin 表达。肥大细胞经 IgE 激活后快速释放组胺、PGD2 和半胱氨酰白三烯；部分患者中，嗜碱性粒细胞可补充 IL-4 等 2 型信号。

IL-5/eotaxin 通路招募的嗜酸性粒细胞释放主要碱性蛋白、嗜酸性粒细胞过氧化物酶、白三烯、细胞因子和 TGF-β，造成上皮损伤、气道高反应性和重塑。IL-4/IL-13 还会改变巨噬细胞功能，使其偏向 2 型/修复相关状态；这些巨噬细胞又可产生趋化因子和生长因子，使局部网络持续。树突状细胞持续呈递过敏原，因此把一次刺激转化为可重复的适应性免疫反应。

消退依赖上皮报警信号下降、IL-5 减少、嗜酸性粒细胞凋亡以及巨噬细胞凋亡细胞清除。IL-10、TGF-β和促炎症消退脂质有助于停止募集和修复组织。但如果 TGF-β等修复信号长期过强，即使嗜酸性粒细胞减少，气道重塑仍可能继续。这也是为什么治疗不能只看嗜酸性粒细胞计数。

Q17

问题

在复发性尿路感染中，既往感染如何通过训练性免疫、免疫耐受或组织重塑改变膀胱黏膜中的中性粒细胞和单核细胞/巨噬细胞反应？目前对这些机制各自的证据强度如何？

回答

既往尿路感染可能通过训练性免疫、耐受和组织重塑影响下一次感染，但三者证据强度不同。训练性免疫指初次刺激在单核细胞或骨髓祖细胞留下表观遗传和代谢改变，使再次感染时产生更快或更强的先天反应。这个概念在基础免疫学中相对成熟，但“它能否通过重编程膀胱单核/巨噬细胞或中性粒细胞祖细胞来预防人类复发性 UTI”，目前直接证据仍有限，主要来自临床前研究。

第二种是耐受。反复 TLR 刺激后，单核/巨噬细胞可降低后续 TNF、IL-1 等输出，同时保留部分抗菌功能。这样可能减少膀胱损伤，也可能因为反应过低而使细菌更容易持续。针对复发性 UTI 的人体机制证据仍不充分。

第三种是组织重塑，组织特异性依据更强。既往感染可改变尿路上皮分化、屏障完整性、局部基质和神经信号，以及驻留免疫细胞组成；细胞内细菌储存库和异常上皮修复也会改变复发阈值。成熟中性粒细胞寿命很短，所以长期“记忆”更可能来自骨髓祖细胞、驻留巨噬细胞或被重塑的组织环境。总体而言，组织重塑最符合膀胱局部的持续效应；训练性免疫和耐受很有吸引力，但尚不能视为已证实的主导机制。

Q18

问题

在膀胱癌中，肿瘤相关中性粒细胞和肿瘤相关巨噬细胞如何在黏膜肿瘤微环境中相互作用，并影响抗原呈递、T 细胞活性、血管生成、肿瘤侵袭以及免疫治疗反应？

回答

膀胱癌中的肿瘤相关巨噬细胞和肿瘤相关中性粒细胞都具有很强的可塑性。肿瘤和基质可通过 CCL2/CSF1、CXCL 类趋化信号把它们招募进来，再利用缺氧、乳酸、TGF-β、IL-10 等改变其功能。偏免疫抑制的巨噬细胞可通过 IL-10、TGF-β、PD-L1、精氨酸代谢和腺苷通路削弱抗原呈递和 T 细胞反应；部分肿瘤相关中性粒细胞也可表现出类似髓源抑制细胞的特征，用 ROS、精氨酸酶、蛋白酶等抑制 T 细胞。

两类细胞都可能促进血管生成和侵袭。巨噬细胞产生 VEGF、MMP 和多种生长因子，促进新生血管和基质重塑；中性粒细胞蛋白酶及 NET 也可改变细胞外基质，在某些环境中帮助肿瘤浸润和播散。二者通过趋化因子和炎症介质互相加强，所以单独阻断一条通路常可能出现代偿。

在免疫治疗方面，髓系抑制性微环境往往不利于免疫检查点抑制剂发挥作用。但膀胱癌也说明“髓系激活”并非一定有害：膀胱灌注 BCG 需要强烈的局部先天免疫反应，随后才形成有效的抗肿瘤适应性免疫。因此治疗目标更应是选择性重编程，而非一概清除。不同患者的 TAN/TAM 亚群差异很大，因果通路仍需进一步明确。

Q19

问题

在溃疡性结肠炎中，哪些具有机制学合理性的策略可能抑制致病性中性粒细胞募集或中性粒细胞胞外诱捕网相关损伤，同时尽量保留抗微生物防御和巨噬细胞介导的修复？请说明预期获益、潜在权衡及证据缺口。

回答

溃疡性结肠炎中更合理的思路是“削弱最伤组织的中性粒细胞功能”，而不是把中性粒细胞全部去掉。第一类策略是部分抑制趋化募集，例如 CXCR2/CXCL8 相关通路，从而减少隐窝内大量聚集及蛋白酶、活性氧损伤。潜在问题是阻断过强会影响细菌清除和创面防御，因此短疗程、局部治疗或只针对活动期可能比长期全身抑制更安全。

第二类是针对 NET 相关损伤。DNase 可破坏 NET 的 DNA 骨架；PAD4、弹性蛋白酶或 MPO 抑制有望减少 NET 形成或毒性。好处是减少组蛋白、蛋白酶和危险信号暴露给上皮与巨噬细胞。但 NET 在某些情况下确实参与微生物限制，而且 DNase 只清除 DNA，并不一定中和附着其上的蛋白酶和组蛋白。

第三类是促进炎症自然消退，例如增强巨噬细胞凋亡细胞清除或应用促炎症消退脂质介质，使早期抗菌功能保留、随后更快“收尾”；同时修复上皮屏障可减少新的中性粒细胞募集刺激。

这些策略大多仍处于机制或临床前/早期研究层面，并非 UC 常规成熟治疗。主要证据缺口包括：哪些患者真正属于 NET 驱动型、抑制多少才不会增加感染、以及能否在降低黏膜损伤的同时保留巨噬细胞修复功能。

Q20

问题

如何利用肠道巨噬细胞和粒细胞的代谢或表观遗传重编程来恢复炎症性肠病中的黏膜稳态？哪些机制可能导致疗效丧失或非预期的免疫抑制？

回答

代谢或表观遗传重编程的目标，不是单纯减少髓系细胞数量，而是把它们从持续炎症状态拉回稳态。在 IBD 炎性巨噬细胞中，强糖酵解、HIF-1α、琥珀酸、mTOR 和促炎染色质程序可支持 IL-1β、TNF 持续产生。理论上，增强 AMPK 和线粒体氧化、利用 itaconate 样抗炎代谢信号，或调节特定表观遗传酶，可使巨噬细胞更倾向凋亡细胞清除、IL-10 和修复。对中性粒细胞等粒细胞，则可考虑降低过度糖酵解、ROS 或 NET 形成，从而减少组织损伤。

最大的风险是这些通路同样参与抗菌。过度抑制糖酵解、NADPH 氧化酶、炎症小体或染色质开放状态，可能削弱吞噬、氧化杀菌、细胞因子反应和疫苗相关免疫。因此，更理想的是肠道局部递送、短期治疗，或通过受体把药物送到炎性髓系亚群，而不是全身长期抑制代谢。

疗效也可能很快丧失，因为髓系细胞持续由骨髓补充，细菌产物和细胞因子可覆盖原有程序，平行通路还能代偿。表观遗传状态本身也具有可塑性。非预期免疫抑制可能表现为感染增加、伤口愈合变差或不能控制穿过黏膜的细菌。目前这更多是转化研究方向，还不是标准 IBD 治疗，关键缺口是患者分层和可监测的代谢生物标志物。

Q21

问题

能够改变 2 型细胞因子信号的生物制剂，除降低嗜酸性粒细胞数量外，还可能如何重塑气道黏膜中的嗜酸性粒细胞-肥大细胞-巨噬细胞-树突状细胞网络？

回答

2 型细胞因子生物制剂对气道网络的影响不只是“把嗜酸性粒细胞降下来”。抗 IL-5 或抗 IL-5 受体治疗会减少嗜酸性粒细胞的生成、存活和组织募集，因此同时降低白三烯、颗粒蛋白、IL-4/IL-13、TGF-β和损伤相关信号。这会间接减轻巨噬细胞、树突状细胞受到的刺激，也减少上皮损伤反过来驱动炎症的机会。

阻断 IL-4/IL-13 的网络效应更广。它可减少 eotaxin、黏液、IgE 相关放大，以及 IL-4/IL-13 驱动的巨噬细胞功能改变；树突状细胞与 T 细胞的互作也会较少向 2 型免疫偏移。再往上游，阻断 TSLP，以及在相应药物和适应证下影响 IL-33 相关通路，可同时降低树突状细胞、ILC2、肥大细胞和下游粒细胞的激活。

针对嗜酸性粒细胞的药物未必明显减少肥大细胞数量，但可改变周围介质环境和其激活阈值。随着上皮损伤减少，巨噬细胞的修复程序也可能恢复正常。需要考虑的是，2 型细胞因子也参与寄生虫防御和部分屏障修复，所以完全抑制并非没有生物学代价。不同患者对不同生物制剂反应差异，往往反映其主要炎症节点位于嗜酸性粒细胞、IL-4/IL-13，还是更上游的上皮报警通路。

Q22

问题

在中性粒细胞性哮喘中，哪些针对髓系细胞的策略可能中断中性粒细胞-巨噬细胞炎症回路，同时保留抗细菌防御？这些策略所依据的机制中，哪些已有临床证据，哪些仍主要处于临床前阶段？

回答

中性粒细胞性哮喘的治疗目标应是打断“过度募集和持续激活”，同时保留足够的中性粒细胞和巨噬细胞抗菌功能。临床上证据相对成熟的是部分重症哮喘患者使用大环内酯类药物，其作用既有抗菌成分，也有对上皮和髓系细胞炎症信号的免疫调节作用。但它并不是纯粹的中性粒细胞靶向治疗，并存在耐药、QT 间期/药物相互作用和微生态改变等问题。

更直接的髓系靶点目前多数尚未成为成熟临床方案。CXCR2 拮抗可降低中性粒细胞迁移，但临床获益并不稳定，阻断过强还可能增加感染风险。IL-1β/NLRP3、GM-CSF、LTB4、弹性蛋白酶、NET/PAD4 以及巨噬细胞炎症代谢等靶点具有较强机制或临床前依据，但目前并非中性粒细胞性哮喘的标准疗法。若患者同时存在支气管扩张或慢性感染，积极控制微生物刺激本身也可间接中断回路。

要保留抗菌防御，更可行的设计是“部分、表型指导、尽量气道局部”的抑制，而不是全身清除中性粒细胞。未来可能结合痰中中性粒细胞、感染状态、炎症小体或 NET 标志物选药。现阶段还没有一个被广泛确立的药物，能专门切断中性粒细胞-巨噬细胞回路并可靠保留抗菌功能。

Q23

问题

训练性免疫或黏膜疫苗策略是否可能通过有益地重编程单核细胞/巨噬细胞和中性粒细胞来减少复发性尿路感染？请解释其拟议机制、潜在的组织特异性风险以及当前证据的局限。

回答

训练性免疫和黏膜疫苗都可能用于减少复发性 UTI，但机制并不相同。黏膜疫苗的主要目标是增强病原体特异性的黏膜抗体和 T 细胞反应，使细菌在到达或侵入膀胱前就被更有效限制，从而减少后续中性粒细胞“暴发式”炎症。训练性免疫则是通过模式识别受体刺激，在单核细胞或骨髓祖细胞留下表观遗传和代谢改变，使后续巨噬细胞和中性粒细胞反应更快、更协调。

潜在获益包括更早的细菌控制、更有效的吞噬，以及更快进入巨噬细胞主导的炎症消退阶段。但“更强的先天记忆”不一定更好：膀胱局部如果训练过度，可能加重中性粒细胞募集、尿路上皮损伤、疼痛或纤维化；如果诱导的是过度耐受，则可能使细胞因子反应不足，增加上行感染风险。成熟中性粒细胞寿命短，因此持久重编程更可能发生在骨髓祖细胞、驻留巨噬细胞或上皮/基质组织记忆层面。

目前证据受限于临床研究规模小、制剂和给药途径不同、致病菌谱差异，以及很少直接取样膀胱黏膜进行机制验证。某些免疫预防方法具有临床前景，但“疗效明确由训练后的单核/巨噬细胞或中性粒细胞介导”这一点仍缺乏直接证据。

Q24

问题

如何通过选择性重编程肿瘤相关巨噬细胞和中性粒细胞来增强膀胱癌的抗肿瘤免疫，同时尽量减少对保护性尿路黏膜免疫的破坏？请比较不同机制策略及其主要转化障碍。

回答

膀胱癌中，选择性重编程通常比把巨噬细胞或中性粒细胞整体清除更合理，因为这两类细胞还承担尿路黏膜抗菌防御。巨噬细胞方面，一类策略是减少免疫抑制性 TAM 的募集或存活，例如干预 CCL2/CCR2 或 CSF1/CSF1R；另一类是功能重编程，例如利用 CD40、TLR/STING、PI3Kγ或代谢相关策略，提高抗原呈递和支持 T 细胞杀伤的能力。重编程比全面清除更可能保留吞噬功能，但激活过强也可能造成膀胱炎或全身炎症。

中性粒细胞方面，可通过 CXCR2 相关通路减少肿瘤促进型 TAN 进入，或通过 TGF-β调节、精氨酸/腺苷通路、NET/PAD4 及中性粒细胞蛋白酶抑制，降低 T 细胞抑制和肿瘤侵袭。主要代价是抗菌能力和组织修复下降。因此膀胱内局部给药很有吸引力：既能充分暴露肿瘤，又可减少全身髓系抑制。BCG 灌注已经证明局部先天免疫激活可以产生抗肿瘤效应，但它并不是选择性 TAM/TAN 重编程药物。

转化障碍包括髓系细胞高度可塑、缺乏只标记“坏细胞”而不影响保护性黏膜细胞的特异标志物、骨髓持续补充、不同肿瘤差异大以及通路代偿。最现实的方向可能是生物标志物指导、局部给药并与免疫检查点抑制剂等联合，而不是长期全身抑制整个髓系谱系。

Traditional Chinese (TC)

Anonymous Model E ｜ 繁體中文 ｜ Informal Response Package

Anonymous Model E

繁體中文

Q01

問題

在健康腸黏膜中，中性粒細胞如何在與駐留巨噬細胞和樹突狀細胞相互作用的同時參與屏障防禦？哪些機制通常限制中性粒細胞介導的組織損傷？

回答

健康腸黏膜並不會長期聚集大量中性粒細胞。正常情況下，它們主要停留在血循環中；只有當上皮屏障出現微小破口、細菌穿越黏液層，或駐留巨噬細胞等哨兵細胞識別到危險訊號時，才會被迅速募集。CXCL1/CXCL2、人體中的 CXCL8，以及補體和脂質介質共同引導中性粒細胞遷移。到達局部後，中性粒細胞通過吞噬、活性氧、顆粒酶和抗菌蛋白殺滅細菌或真菌，刺激較強時也可形成胞外誘捕網。駐留巨噬細胞一方面產生募集訊號，另一方面負責吞噬病原體和後續清除細胞碎片；樹突狀細胞則更多承擔抗原取樣和連接適應性免疫的作用。

中性粒細胞的殺菌工具同樣可能傷害上皮，因此健康黏膜存在多層"剎車"。黏液、緊密連接、抗菌肽和快速上皮更新先把微生物限制在腔面；腸道巨噬細胞對無害共生菌相對低反應，並持續產生 IL-10 等調節因子。病原負荷下降後，中性粒細胞進入凋亡，隨後被巨噬細胞進行凋亡細胞清除，這會推動 IL-10、TGF-β和組織修復程序，同時減少新的中性粒細胞募集。促發炎消退脂質介質、抗氧化系統、抗蛋白酶以及局部引流也可降低彈性蛋白酶、MPO 產物和胞外 DNA 造成的附帶損傷。

臨床上最關鍵的是"短、準、能停"：短暫而局限的中性粒細胞反應是屏障防禦的一部分；如果募集持續、NET 或蛋白酶過多、凋亡細胞清除失敗，就會從保護轉為黏膜損傷。

Q02

問題

在健康腸黏膜中，嗜酸性粒細胞如何參與維持穩態並與巨噬細胞或樹突狀細胞進行通訊？這些作用與發炎狀態下嗜酸性粒細胞的功能有何不同？

回答

嗜酸性粒細胞本來就是腸固有層的常駐細胞，因此在腸道看到它們並不等同於發炎。穩態下，IL-5 以及通過 CCR3 起作用的 eotaxin 類趨化因子維持其存活和組織定位。它們參與屏障監視、局部免疫調節和組織更新，還可通過細胞激素、脂質介質和生長因子影響上皮、基質細胞及 IgA 相關反應。與巨噬細胞和樹突狀細胞的交流通常較溫和：嗜酸性粒細胞來源的 IL-4/IL-13 可使部分巨噬細胞偏向修復和組織重塑功能，而巨噬細胞、樹突狀細胞釋放的細胞激素又會影響嗜酸性粒細胞的存活和活化。其直接改變樹突狀細胞成熟或抗原呈遞的程度，則更依賴具體環境。

發炎時情況明顯不同。活化的嗜酸性粒細胞會釋放主要鹼性蛋白、嗜酸性粒細胞陽離子蛋白、嗜酸性粒細胞過氧化物酶、白三烯、細胞激素和活性氧。這些介質可增加上皮通透性、促進血管和神經反應，並進一步激活其他髓系細胞。在 2 型發炎中，它們還會加強 IL-4/IL-13 主導的巨噬細胞程序；在混合型發炎中，也可與中性粒細胞和炎性單核細胞同時出現。

因此，穩態嗜酸性粒細胞更接近"組織適應型的調節/修復細胞"；發炎嗜酸性粒細胞則表現為更強的脫顆粒和細胞激素輸出。兩者並非截然分開，人類腸道疾病中的表型差異很大。

Q03

問題

在健康呼吸道黏膜以及急性微生物攻擊期間，中性粒細胞如何與肺泡或間質巨噬細胞及樹突狀細胞協同，在發揮宿主防禦作用的同時限制對上皮的附帶損傷？

回答

健康氣道中，肺泡巨噬細胞是肺泡腔最主要的吞噬哨兵，平時需要在不斷接觸吸入顆粒的情況下維持低發炎狀態。急性微生物刺激出現後，肺泡或間質巨噬細胞以及氣道上皮的模式識別受體被激活，產生 IL-1、TNF、CXCL 類趨化因子、G-CSF 等，迅速招募中性粒細胞。中性粒細胞通過吞噬、氧化爆發、顆粒酶和必要時的 NET 形成進行快速抗菌/抗真菌防禦。樹突狀細胞負責攝取抗原並遷移至淋巴結，決定後續適應性免疫的強度和方向。

真正保護肺組織的關鍵是"及時關停"。病原負荷下降後，中性粒細胞凋亡，隨後被巨噬細胞清除；這一過程會降低 IL-1/TNF 樣訊號，並增強 IL-10、TGF-β和促發炎消退脂質介質。α1-抗胰蛋白酶、分泌型白細胞蛋白酶抑制劑等抗蛋白酶，局部抗氧化系統、DNase、黏液纖毛清除和上皮修復，可減少彈性蛋白酶、MPO 產物及胞外 DNA 對上皮的傷害。

如果病原沒有被清除，或巨噬細胞的凋亡細胞清除能力下降，這個協同防禦迴路就可能變成持續發炎迴路。臨床上在重症肺炎、COPD 急性加重、支氣管擴張和部分重症氣喘中都可看到這種"既抗菌、又可能傷肺"的雙重性。

Q04

問題

在穩態條件和第 2 型發炎期間，嗜酸性粒細胞如何在氣道黏膜中與巨噬細胞和樹突狀細胞相互作用？

回答

穩態氣道中的嗜酸性粒細胞數量通常不高，與巨噬細胞和樹突狀細胞的互作也比較有限。此時肺泡巨噬細胞主要負責清除顆粒和抑制不必要的發炎，樹突狀細胞持續進行低水平抗原取樣。少量嗜酸性粒細胞可能參與組織監視和修復，但在健康氣道中，並沒有像腸道那樣明確的、佔主導地位的嗜酸性粒細胞穩態網路。

進入 2 型發炎後，網路迅速放大。受損或接觸過敏原的上皮釋放 TSLP、IL-33 和 IL-25，激活樹突狀細胞和 ILC2；Th2/ILC2 產生 IL-5 促進嗜酸性粒細胞生成和存活，IL-4/IL-13 誘導 eotaxin、黏液和屏障改變。募集的嗜酸性粒細胞釋放白三烯、顆粒蛋白、細胞激素和生長因子，其 IL-4/IL-13 可加強偏修復/2 型的巨噬細胞狀態；巨噬細胞又可產生 CCL17、CCL22 等，幫助維持 Th2 細胞環境。樹突狀細胞通過持續呈遞過敏原，使這種反應獲得適應性免疫“記憶”。

巨噬細胞也參與消退，例如清除凋亡嗜酸性粒細胞並產生調節介質。所以，這套網路在短期內可服務於修復，但若嗜酸性粒細胞持續脫顆粒、黏液分泌和氣道高反應性持續存在，就會變成致病迴路。不同氣喘表型中，嗜酸性粒細胞對樹突狀細胞的直接影響強弱並不一致。

Q05

問題

在尿路黏膜中，早期抗菌防禦期間，中性粒細胞如何與單核細胞或巨噬細胞及樹突狀細胞協同？哪些機制可防止過度組織損傷？

回答

尿路早期抗菌防禦首先依賴尿路上皮和局部髓系細胞對細菌成分的識別。上皮細胞、巨噬細胞和樹突狀細胞可釋放 IL-1、IL-6 及 CXCL 類趨化因子，快速把中性粒細胞從血液招募到黏膜。中性粒細胞通常是最早、最強的效應細胞，通過跨上皮遷移、吞噬、活性氧、顆粒抗菌蛋白以及必要時的 NET 形成來清除細菌。單核細胞/巨噬細胞同時參與吞噬和細胞激素產生，並在稍後階段負責清除死亡中性粒細胞；樹突狀細胞更偏向抗原取樣和連接適應性免疫。

尿流衝刷和上皮脫落本身已經有助於清除細菌，因此如果中性粒細胞反應過強，反而會增加損傷。彈性蛋白酶等蛋白酶、活性氧和 NET 成分可破壞尿路上皮，使通透性增加。正常情況下，快速降低細菌負荷、中性粒細胞凋亡、巨噬細胞的凋亡細胞清除、IL-10/TGF-β主導的消退訊號、局部抗氧化/抗蛋白酶系統以及尿路上皮再生共同限制組織損傷。

從臨床角度看，募集不足可能導致細菌持續甚至上行感染；募集過久則會加重尿痛、血尿和黏膜損傷。巨噬細胞的價值不僅在“幫忙殺菌”，還在於及時把發炎切換到修復階段。

Q06

問題

在膀胱或下泌尿生殖道黏膜中，肥大細胞釋放的介質如何影響巨噬細胞和樹突狀細胞的功能？這一網路如何同時參與宿主防禦和發炎相關症狀？

回答

膀胱和下泌尿生殖道黏膜中的肥大細胞可被微生物成分、IgE 依賴訊號、組織損傷、補體和神經肽激活，隨後釋放組胺、胰蛋白酶樣蛋白酶、TNF、前列腺素、白三烯及多種細胞激素。急性階段，這些介質提高血管通透性和局部血流，並促進中性粒細胞、單核細胞進入組織，因此對感染防禦有一定幫助。

巨噬細胞和樹突狀細胞可通過細胞激素受體、組胺受體、蛋白酶激活受體及脂質介質受體感受肥大細胞訊號。TNF 和部分脂質介質可增強樹突狀細胞成熟和發炎型巨噬細胞活性，幫助抗原呈遞和抗菌；反過來，巨噬細胞產生的 IL-1、TNF 和危險訊號也可進一步刺激肥大細胞。與此同時，肥大細胞並非只會“促炎”，某些情況下也能釋放調節介質，使發炎重新分配或減弱。

症狀往往來自同一套網路的另一面。組胺、胰蛋白酶、前列腺素、白三烯及 NGF 相關訊號可敏化感覺神經，引起尿急、疼痛和尿頻；血管和上皮通透性增加又讓尿液中的刺激物更容易接觸黏膜。若這種正反饋持續，就可能在沒有活動性感染時仍維持症狀。不同疾病——感染、間質性膀胱炎/膀胱疼痛綜合徵等——主導通路可能並不相同。

Q07

問題

在急性腸黏膜發炎及其後的發炎消退階段，中性粒細胞與巨噬細胞如何透過募集訊號、細胞激素、凋亡細胞清除、脂質介質和組織修復程序相互影響？

回答

急性腸黏膜發炎開始時，上皮和髓系細胞產生 CXCL 趨化因子，補體和 LTB4 等脂質介質共同驅動中性粒細胞快速進入病灶。中性粒細胞殺菌的同時也會釋放 IL-1 相關訊號、蛋白酶、活性氧和危險分子，進一步招募 CCR2 依賴的單核細胞。單核細胞進入組織後形成炎性巨噬細胞，產生 TNF、IL-1β等細胞激素；如果微生物和組織危險訊號持續存在，就會繼續推動中性粒細胞募集。

發炎消退不是“細胞自然消失”，而是主動轉換。病原負荷下降後，中性粒細胞停止接受存活訊號並進入凋亡，膜表面暴露磷脂醯絲氨酸。巨噬細胞通過 MerTK 等受體相關途徑進行凋亡細胞清除。這個過程會抑制新的促炎細胞激素，同時增加 IL-10、TGF-β、生長因子和修復程序。脂質介質也可從以白三烯為主轉向脂氧素、resolvin、protectin、maresin 等促發炎消退介質，從而減少新的中性粒細胞進入。

隨後巨噬細胞支持上皮再生、基質重塑和屏障恢復。這裡不宜簡單概括為“M1 變 M2”，因為腸道巨噬細胞狀態更複雜。如果中性粒細胞不易凋亡、巨噬細胞清除能力受損，或病原持續刺激，發炎就會延長。也就是說，中性粒細胞既能製造損傷，也能通過“被正確清除”向巨噬細胞發出進入修復期的訊號。

Q08

問題

嗜酸性粒細胞來源的介質如何改變腸黏膜巨噬細胞的功能狀態和樹突狀細胞的活化？這些相互作用如何推動免疫耐受、組織修復與發炎之間的平衡發生變化？

回答

嗜酸性粒細胞可以通過細胞激素、脂質介質、顆粒蛋白和生長因子改變腸黏膜巨噬細胞的功能。嗜酸性粒細胞產生的 IL-4 和 IL-13 可使部分巨噬細胞偏向組織修復、基質處理和 2 型免疫相關程序；TGF-β等生長因子在短期內有助於上皮修復，但如果持續過高，也可能推動纖維化。嗜酸性粒細胞的脂質介質可改變血管和髓系細胞活性；而強烈脫顆粒時，顆粒蛋白和氧化產物本身又可作為危險訊號。

它們對樹突狀細胞的影響更依賴具體情境。嗜酸性粒細胞來源的細胞激素可改變樹突狀細胞成熟、遷移和抗原呈遞時的細胞激素環境。在穩態或輕度刺激下，這種作用可能幫助維持對食物和共生菌的“可控反應”；在強烈 2 型或混合發炎中，則可能增加 T 細胞活化並促進更多粒細胞募集。嗜酸性粒細胞在某些實驗條件下可呈遞抗原，但在人類腸黏膜中通常不是主要的專業抗原呈遞細胞。

因此，結果取決於激活強度和時序：適度訊號偏向耐受和修復；持續 IL-4/IL-13/TGF-β可能造成重塑；大量脫顆粒則更容易造成上皮損傷和樹突狀細胞激活。描述巨噬細胞時採用連續功能譜，比簡單的“M1/M2 二分法”更準確。

Q09

問題

在中性粒細胞性氣道發炎中，中性粒細胞與巨噬細胞如何透過細胞激素、脂質介質、中性粒細胞胞外誘捕網、凋亡細胞清除和代謝訊號相互影響？這種串擾如何決定發炎持續還是消退？

回答

中性粒細胞性氣道發炎中，巨噬細胞和中性粒細胞很容易形成正反饋。巨噬細胞和氣道上皮釋放 IL-1β、TNF、GM-CSF、CXCL8 等，使中性粒細胞持續進入並延長存活；中性粒細胞則釋放 LTB4、蛋白酶、活性氧和其他發炎介質，再次刺激巨噬細胞和上皮。NET 在控制微生物時有價值，但胞外 DNA、組蛋白、彈性蛋白酶和 MPO 產物也可激活危險感知和發炎小體相關通路，增加黏液分泌及上皮損傷。

代謝環境會決定這個迴路是否容易“熄火”。中性粒細胞高度依賴糖酵解，因此即使在缺氧、黏稠的痰液中仍可工作；但高乳酸、琥珀酸、低氧和氧化應激也會改變巨噬細胞 HIF-1α及線粒體訊號，使 IL-1β等促炎介質持續。相反，IL-10、促發炎消退脂質和部分抗炎代謝程序可推動巨噬細胞退出發炎狀態。

真正的消退依賴中性粒細胞凋亡和巨噬細胞有效的凋亡細胞清除。清除後，巨噬細胞會減少促炎細胞激素並增加 TGF-β、IL-10 和脂質介質轉換。若清除失敗、NET 負荷過高或細菌/病毒訊號持續，發炎就容易長期存在。COPD、支氣管擴張、重症感染和部分中性粒細胞性氣喘都可出現這一模式，但各機制所佔比重不同。

Q10

問題

在過敏性氣道發炎中，肥大細胞和嗜酸性粒細胞（在相關情境下包括嗜鹼性粒細胞）如何與樹突狀細胞和巨噬細胞相互作用，從而放大或限制第 2 型免疫反應？

回答

過敏性氣道發炎由多個細胞共同構成 2 型免疫網路。上皮接觸過敏原後釋放 TSLP、IL-33 和 IL-25，樹突狀細胞由此促進 Th2 分化，ILC2 也產生 IL-5 和 IL-13。肥大細胞經 IgE 交聯後迅速釋放組胺、PGD2、白三烯和細胞激素；某些情況下，嗜鹼性粒細胞可提供較早的 IL-4 和脂質介質。IL-5 和 eotaxin 通路共同招募並維持嗜酸性粒細胞。

嗜酸性粒細胞通過半胱氨酰白三烯、顆粒蛋白、細胞激素和生長因子繼續放大發炎。其 IL-4/IL-13 可加強巨噬細胞的 2 型/修復相關程序，巨噬細胞又能產生 CCL17、CCL22 等趨化因子，幫助 Th2 細胞維持在氣道。樹突狀細胞不斷接收肥大細胞和嗜酸性粒細胞的介質訊號，同時持續呈遞過敏原，因此使反應從一次性發炎變成可反復啓動的適應性免疫反應。

限制這套網路主要依靠巨噬細胞清除凋亡嗜酸性粒細胞、IL-10/TGF-β、調節性 T 細胞和促發炎消退脂質介質。若這些“剎車”不足，肥大細胞激活、嗜酸性粒細胞脫顆粒和巨噬細胞重塑訊號就形成持續正反饋。嗜鹼性粒細胞數量通常更少，其重要性也更依賴具體表型。

Q11

問題

在急性細菌性尿路感染期間，募集到局部的中性粒細胞和單核細胞來源巨噬細胞如何在病原體清除、細胞激素產生、凋亡細胞清除和黏膜修復過程中協同或競爭？

回答

急性細菌性尿路感染中，中性粒細胞到達最快，負責高強度、短時的殺菌；募集的單核細胞及其分化形成的巨噬細胞則承擔更廣泛、隨時間變化的任務。兩者都能吞噬細菌，但中性粒細胞更擅長快速氧化爆發、脫顆粒和短壽命抗菌。單核/巨噬細胞產生 IL-1、TNF 和趨化因子，維持中性粒細胞募集，也可直接吞噬病原體和處理組織碎片。因此早期二者多是協同關係：巨噬細胞“叫人”，中性粒細胞迅速降低細菌負荷。

競爭或衝突主要發生在反應過強時。大量中性粒細胞消耗氧和營養，並釋放蛋白酶、活性氧或 NET，可能造成尿路上皮損傷；這些損傷訊號又使巨噬細胞繼續保持發炎狀態。反過來，巨噬細胞通過吞噬凋亡中性粒細胞主動結束發炎，所以如果過早抑制中性粒細胞會影響殺菌，而如果死亡中性粒細胞遲遲不能清除，又會加重組織損傷。

細菌控制後，巨噬細胞在消退和修復階段更關鍵：清除凋亡細胞，釋放 IL-10、TGF-β和生長因子，促進尿路上皮再生。換句話說，早期強調共同抗菌，後期則應轉為“巨噬細胞清場和修復”。

Q12
問題
在慢性膀胱黏膜發炎中，肥大細胞與巨噬細胞之間的串擾可能如何改變感覺神經訊號、血管通透性、上皮屏障功能以及發炎的持續或消退？

回答
慢性膀胱黏膜發炎中，肥大細胞和巨噬細胞可共同放大發炎、血管和感覺神經訊號。肥大細胞釋放組胺、胰蛋白酶樣蛋白酶、前列腺素、白三烯、TNF 和生長因子；組胺和脂質介質增加血管通透性，蛋白酶可激活 PAR 類受體，同時這些介質可敏化 TRPV1 等感覺神經通路。局部巨噬細胞又可釋放 IL-1β、TNF、趨化因子、PGE2 和 NGF 相關訊號，使疼痛、尿急進一步增強。

屏障損傷會讓迴路更穩定。血管滲漏帶來更多血漿蛋白和發炎細胞；受損尿路上皮對尿液中的溶質、微生物成分和損傷相關分子更敏感。上述訊號再次刺激肥大細胞和巨噬細胞，形成正反饋。持續的巨噬細胞促炎狀態會延緩上皮修復，肥大細胞蛋白酶也可能影響緊密連接和細胞外基質。

如果最初刺激被去除，巨噬細胞轉向凋亡細胞清除和修復，並增加 IL-10、TGF-β和促發炎消退脂質，肥大細胞激活下降，屏障恢復，神經和血管刺激就可減弱。需要注意的是，在人類慢性膀胱疼痛相關疾病中，肥大細胞增多並非所有患者都存在，因此這一機制更適合解釋部分表型，而不是統一解釋全部病例。

Q13
問題
在潰瘍性結腸炎中，中性粒細胞募集、中性粒細胞胞外誘捕網形成、中性粒細胞代謝以及中性粒細胞-巨噬細胞串擾如何分別促進黏膜損傷或發炎消退？

回答
潰瘍性結腸炎活動期最典型的病理之一就是中性粒細胞進入隱窩，形成隱窩炎和隱窩膿腫。上皮和髓系細胞產生 CXCL8 等趨化因子，加上補體和脂質介質，使中性粒細胞大量募集。早期這有保護意義，因為屏障破損後需要迅速吞噬越過上皮的細菌；問題在於反應持續時，彈性蛋白酶、MPO 產物、活性氧等會直接損傷上皮，使屏障缺口繼續擴大。

NET 形成同樣具有雙重性。NET 可限制微生物擴散，但胞外 DNA、組蛋白、蛋白酶和氧化酶暴露在組織中時會造成細胞毒性，並可能通過 TLR、發炎小體等危險感知通路刺激巨噬細胞持續產生 IL-1β和 TNF。中性粒細胞高度依賴糖酵解，因此在缺氧隱窩中仍能保持活性；與此同時，大量耗氧和代謝產物也會進一步改變局部低氧和巨噬細胞代謝狀態。

發炎消退依賴中性粒細胞凋亡後被巨噬細胞及時清除。有效的凋亡細胞清除會降低促炎因子，增加 IL-10、TGF-β、促發炎消退脂質和上皮修復。臨床上"黏膜中性粒細胞多"與活動性疾病關係很明確；至於某一種 NET 或代謝通路在人類患者中究竟佔多大因果比例，目前證據還沒有同樣扎實。

Q14
問題
在克隆氏症中，中性粒細胞、單核細胞來源巨噬細胞和樹突狀細胞之間失調的相互作用如何改變微生物處理、抗原呈遞、發炎持續以及上皮修復？

回答
克隆氏症可同時存在"微生物處理不夠有效"和"後續發炎過強"兩個問題。中性粒細胞本應快速清除穿過黏膜的細菌，但在部分患者中，募集的時序或殺菌效率可能不理想。病原體或其產物持續存在後，CCR2 依賴的單核細胞進入組織並形成炎性巨噬細胞，產生 TNF、IL-1β、IL-6、IL-23 等，支持 Th1/Th17 反應並繼續招募髓系細胞。

樹突狀細胞持續攝取微生物和食物抗原，在發炎細胞激素環境下提高共刺激和抗原呈遞。與克隆氏症有關的 NOD2、ATG16L1 等通路可影響不同細胞的微生物識別或自噬處理。這樣就可能出現一個不利組合：細菌沒有被迅速、徹底清除，但抗原呈遞和發炎訊號卻長期存在，最終形成慢性免疫激活。

這些細胞同樣具有修復作用。巨噬細胞可清除凋亡中性粒細胞，產生生長因子和 TGF-β；樹突狀細胞在非發炎環境下也可促進耐受。克隆氏症中，持續微生物刺激、細胞激素、缺氧和基質損傷會阻礙這種轉換；而過強的修復/TGF-β訊號又可能參與纖維化和狹窄。因此核心不是單純"某類細胞太多"，而是微生物處理、抗原呈遞、發炎關停和上皮修復之間失去協調。

Q15
問題
在重度中性粒細胞性氣喘中，中性粒細胞、巨噬細胞和樹突狀細胞如何形成自我維持的發炎迴路？中性粒細胞胞外誘捕網、發炎小體訊號、微生物訊號以及糖皮質激素抗性可能分別發揮什麼作用？

回答
重度中性粒細胞性氣喘可由氣道上皮、巨噬細胞、樹突狀細胞、Th17 樣反應和中性粒細胞共同構成自我維持迴路。上皮和巨噬細胞產生 IL-1β、TNF、IL-6、GM-CSF 和 CXCL8，促進中性粒細胞募集並延長其存活。樹突狀細胞接觸微生物產物和發炎細胞激素後，可支持 IL-17 相關 T 細胞反應；IL-17 又刺激上皮產生更多中性粒細胞趨化因子。中性粒細胞釋放彈性蛋白酶、活性氧和發炎介質，再次破壞上皮並製造新的危險訊號。

NET 可能進一步放大迴路。胞外 DNA、組蛋白和中性粒細胞酶可刺激上皮和巨噬細胞，並促進 NLRP3/IL-1β等發炎小體相關訊號。慢性細菌定植、氣道微生態改變或反復病毒感染，會持續激活模式識別受體，使發炎更難停止。

糖皮質激素抗性通常不是單一機製造成的。氧化應激、p38-MAPK 相關訊號、糖皮質激素受體功能改變、持續的 IL-17/IL-1 通路，以及中性粒細胞本身對激素較不敏感和存活延長，都可能參與。不同患者並非都以 NET 或發炎小體為主導，因此臨床上需要表型分層，而不能把所有中性粒細胞性氣喘歸為同一種機制。

Q16
問題
在嗜酸性粒細胞性氣喘中，嗜酸性粒細胞、肥大細胞以及在相關情境下的嗜鹼性粒細胞，如何與巨噬細胞、樹突狀細胞和氣道上皮相互作用，從而啟動、放大並最終促使第 2 型發炎消退？

回答
嗜酸性粒細胞性氣喘通常從氣道上皮的"報警"開始。過敏原、病毒、污染物或機械刺激可誘導 TSLP、IL-33、IL-25。樹突狀細胞在這些訊號下促進過敏原特異性 Th2 反應，ILC2 也被激活；Th2 和 ILC2 產生 IL-5，IL-4/IL-13 則促進 IgE、黏液和 eotaxin 表達。肥大細胞經 IgE 激活後快速釋放組胺、PGD2 和半胱氨酰白三烯；部分患者中，嗜鹼性粒細胞可補充 IL-4 等 2 型訊號。

IL-5/eotaxin 通路招募的嗜酸性粒細胞釋放主要鹼性蛋白、嗜酸性粒細胞過氧化物酶、白三烯、細胞激素和 TGF-β，造成上皮損傷、氣道高反應性和重塑。IL-4/IL-13 還會改變巨噬細胞功能，使其偏向 2 型/修復相關狀態；這些巨噬細胞又可產生趨化因子和生長因子，使局部網路持續。樹突狀細胞持續呈遞過敏原，因此把一次刺激轉化為可重復的適應性免疫反應。

消退依賴上皮報警訊號下降、IL-5 減少、嗜酸性粒細胞凋亡以及巨噬細胞凋亡細胞清除。IL-10、TGF-β和促發炎消退脂質有助於停止募集和修復組織。但如果 TGF-β等修復訊號長期過強，即使嗜酸性粒細胞減少，氣道重塑仍可能繼續。這也是為甚麼治療不能只看嗜酸性粒細胞計數。

Q17
問題
在復發性尿路感染中，既往感染如何透過訓練性免疫、免疫耐受或組織重塑改變膀胱黏膜中的中性粒細胞和單核細胞/巨噬細胞反應？目前對這些機制各自的證據強度如何？

回答
既往尿路感染可能通過訓練性免疫、耐受和組織重塑影響下一次感染，但三者證據強度不同。訓練性免疫指初次刺激在單核細胞或骨髓祖細胞留下表觀遺傳和代謝改變，使再次感染時產生更快或更強的先天反應。這個概念在基礎免疫學中相對成熟，但"它能否通過重編程膀胱單核/巨噬細胞或中性粒細胞祖細胞來預防人類復發性 UTI"，目前直接證據仍有限，主要來自臨床前研究。

第二種是耐受。反復 TLR 刺激後，單核/巨噬細胞可降低後續 TNF、IL-1 等輸出，同時保留部分抗菌功能。這樣可能減少膀胱損傷，也可能因為反應過低而使細菌更容易持續。針對復發性 UTI 的人體機制證據仍不充分。

第三種是組織重塑，組織特異性依據更強。既往感染可改變尿路上皮分化、屏障完整性、局部基質和神經訊號，以及駐留免疫細胞組成；細胞內細菌儲存庫和異常上皮修復也會改變復發閾值。成熟中性粒細胞壽命很短，所以長期"記憶"更可能來自骨髓祖細胞、駐留巨噬細胞或被重塑的組織環境。總體而言，組織重塑最符合膀胱局部的持續效應；訓練性免疫和耐受很有吸引力，但尚不能視為已證實的主導機制。

Q18
問題
在膀胱癌中，腫瘤相關中性粒細胞和腫瘤相關巨噬細胞如何在黏膜腫瘤微環境中相互作用，並影響抗原呈遞、T 細胞活性、血管新生、腫瘤侵襲以及免疫治療反應？

回答
膀胱癌中的腫瘤相關巨噬細胞和腫瘤相關中性粒細胞都具有很強的可塑性。腫瘤和基質可通過 CCL2/CSF1、CXCL 類趨化訊號把它們招募進來，再利用缺氧、乳酸、TGF-β、IL-10 等改變其功能。偏免疫抑制的巨噬細胞可通過 IL-10、TGF-β、PD-L1、精氨酸代謝和腺苷通路削弱抗原呈遞和 T 細胞反應；部分腫瘤相關中性粒細胞也可表現出類似髓源抑制細胞的特徵，用 ROS、精氨酸酶、蛋白酶等抑制 T 細胞。

兩類細胞都可能促進血管生成和侵襲。巨噬細胞產生 VEGF、MMP 和多種生長因子，促進新生血管和基質重塑；中性粒細胞蛋白酶及 NET 也可改變細胞外基質，在某些環境中幫助腫瘤浸潤和播散。二者通過趨化因子和發炎介質互相加強，所以單獨阻斷一條通路常可能出現代償。

在免疫治療方面，髓系抑制性微環境往往不利於免疫檢查點抑制劑發揮作用。但膀胱癌也說明"髓系激活"並非一定有害：膀胱灌注 BCG 需要強烈的局部先天免疫反應，隨後才形成有效的抗腫瘤適應性免疫。因此治療目標更應是選擇性重編程，而非一概清除。不同患者的 TAN/TAM 亞群差異很大，因果通路仍需進一步明確。

Q19
問題
在潰瘍性結腸炎中，哪些具有機制學合理性的策略可能抑制致病性中性粒細胞募集或中性粒細胞胞外誘捕網相關損傷，同時盡量保留抗微生物防禦和巨噬細胞介導的修復？請說明預期獲益、潛在權衡及證據缺口。

回答
潰瘍性結腸炎中更合理的思路是"削弱最傷組織的中性粒細胞功能"，而不是把中性粒細胞全部去掉。第一類策略是部分抑制趨化募集，例如 CXCR2/CXCL8 相關通路，從而減少隱窩內大量聚集及蛋白酶、活性氧損傷。潛在問題是阻斷過強會影響細菌清除和創面防禦，因此短療程、局部治療或只針對活動期可能比長期全身抑制更安全。

第二類是針對 NET 相關損傷。DNase 可破壞 NET 的 DNA 骨架；PAD4、彈性蛋白酶或 MPO 抑制有望減少 NET 形成或毒性。好處是減少組蛋白、蛋白酶和危險訊號暴露給上皮與巨噬細胞。但 NET 在某些情況下確實參與微生物限制，而且 DNase 只清除 DNA，並不一定中和附著其上的蛋白酶和組蛋白。

第三類是促進發炎自然消退，例如增強巨噬細胞凋亡細胞清除或應用促發炎消退脂質介質，使早期抗菌功能保留、隨後更快"收尾"；同時修復上皮屏障可減少新的中性粒細胞募集刺激。

這些策略大多仍處於機制或臨床前/早期研究層面，並非 UC 常規成熟治療。主要證據缺口包括：哪些患者真正屬於 NET 驅動型、抑制多少才不會增加感染、以及能否在降低黏膜損傷的同時保留巨噬細胞修復功能。

Q20
問題
如何利用腸道巨噬細胞和粒細胞的代謝或表觀遺傳重編程來恢復發炎性腸病中的黏膜穩態？哪些機制可能導致療效喪失或非預期的免疫抑制？

回答
代謝或表觀遺傳重編程的目標，不是單純減少髓系細胞數量，而是把它們從持續發炎狀態拉回穩態。在 IBD 炎性巨噬細胞中，強糖酵解、HIF-1α、琥珀酸、mTOR 和促炎染色質程序可支持 IL-1β、TNF 持續產生。理論上，增強 AMPK 和線粒體氧化、利用 itaconate 樣抗炎代謝訊號，或調節特定表觀遺傳酶，可使巨噬細胞更傾向凋亡細胞清除、IL-10 和修復。對中性粒細胞等粒細胞，則可考慮降低過度糖酵解、ROS 或 NET 形成，從而減少組織損傷。

最大的風險是這些通路同樣參與抗菌。過度抑制糖酵解、NADPH 氧化酶、發炎小體或染色質開放狀態，可能削弱吞噬、氧化殺菌、細胞激素反應和疫苗相關免疫。因此，更理想的是腸道局部遞送、短期治療，或通過受體把藥物送到炎性髓系亞群，而不是全身長期抑制代謝。

療效也可能很快喪失，因為髓系細胞持續由骨髓補充，細菌產物和細胞激素可覆蓋原有程序，平行通路還能代償。表觀遺傳狀態本身也具有可塑性。非預期免疫抑制可能表現為感染增加、傷口愈合變差或不能控制穿過黏膜的細菌。目前這更多是轉化研究方向，還不是標準 IBD 治療，關鍵缺口是患者分層和可監測的代謝生物標誌物。

Q21
問題
能夠改變第 2 型細胞激素訊號的生物製劑，除降低嗜酸性粒細胞數量外，還可能如何重塑氣道黏膜中的嗜酸性粒細胞-肥大細胞-巨噬細胞-樹突狀細胞網路？

回答
2 型細胞激素生物制劑對氣道網路的影響不只是“把嗜酸性粒細胞降下來”。抗 IL-5 或抗 IL-5 受體治療會減少嗜酸性粒細胞的生成、存活和組織募集，因此同時降低白三烯、顆粒蛋白、IL-4/IL-13、TGF-β和損傷相關訊號。這會間接減輕巨噬細胞、樹突狀細胞受到的刺激，也減少上皮損傷反過來驅動發炎的機會。

阻斷 IL-4/IL-13 的網路效應更廣。它可減少 eotaxin、黏液、IgE 相關放大，以及 IL-4/IL-13 驅動的巨噬細胞功能改變；樹突狀細胞與 T 細胞的互作也會較少向 2 型免疫偏移。再往上游，阻斷 TSLP，以及在相應藥物和適應證下影響 IL-33 相關通路，可同時降低樹突狀細胞、ILC2、肥大細胞和下游粒細胞的激活。

針對嗜酸性粒細胞的藥物未必明顯減少肥大細胞數量，但可改變周圍介質環境和其激活閾值。隨著上皮損傷減少，巨噬細胞的修復程序也可能恢復正常。需要考慮的是，2 型細胞激素也參與寄生蟲防禦和部分屏障修復，所以完全抑制並非沒有生物學代價。不同患者對不同生物制劑反應差異，往往反映其主要發炎節點位於嗜酸性粒細胞、IL-4/IL-13，還是更上游的上皮報警通路。

Q22
問題
在中性粒細胞性氣喘中，哪些針對髓系細胞的策略可能中斷中性粒細胞-巨噬細胞發炎迴路，同時保留抗細菌防禦？這些策略所依據的機制中，哪些已有臨床證據，哪些仍主要處於臨床前階段？

回答
中性粒細胞性氣喘的治療目標應是打斷“過度募集和持續激活”，同時保留足夠的中性粒細胞和巨噬細胞抗菌功能。臨床上證據相對成熟的是部分重症氣喘患者使用大環內酯類藥物，其作用既有抗菌成分，也有對上皮和髓系細胞發炎訊號的免疫調節作用。但它並不是純粹的中性粒細胞靶向治療，並存在耐藥、QT 間期/藥物相互作用和微生態改變等問題。

更直接的髓系靶點目前多數尚未成為成熟臨床方案。CXCR2 拮抗可降低中性粒細胞遷移，但臨床獲益並不穩定，阻斷過強還可能增加感染風險。IL-1β/NLRP3、GM-CSF、LTB4、彈性蛋白酶、NET/PAD4 以及巨噬細胞發炎代謝等靶點具有較強機制或臨床前依據，但目前並非中性粒細胞性氣喘的標準療法。若患者同時存在支氣管擴張或慢性感染，積極控制微生物刺激本身也可間接中斷迴路。

要保留抗菌防禦，更可行的設計是“部分、表型指導、盡量氣道局部”的抑制，而不是全身清除中性粒細胞。未來可能結合痰中中性粒細胞、感染狀態、發炎小體或 NET 標誌物選藥。現階段還沒有一個被廣泛確立的藥物，能專門切斷中性粒細胞-巨噬細胞迴路並可靠保留抗菌功能。

Q23
問題
訓練性免疫或黏膜疫苗策略是否可能透過有益地重編程單核細胞/巨噬細胞和中性粒細胞來減少復發性尿路感染？請解釋其擬議機制、潛在的組織特異性風險以及目前證據的侷限。

回答
訓練性免疫和黏膜疫苗都可能用於減少復發性 UTI，但機制並不相同。黏膜疫苗的主要目標是增強病原體特異性的黏膜抗體和 T 細胞反應，使細菌在到達或侵入膀胱前就被更有效限制，從而減少後續中性粒細胞“暴發式”發炎。訓練性免疫則是通過模式識別受體刺激，在單核細胞或骨髓祖細胞留下表觀遺傳和代謝改變，使後續巨噬細胞和中性粒細胞反應更快、更協調。

潛在獲益包括更早的細菌控制、更有效的吞噬，以及更快進入巨噬細胞主導的發炎消退階段。但“更強的先天記憶”不一定更好：膀胱局部如果訓練過度，可能加重中性粒細胞募集、尿路上皮損傷、疼痛或纖維化；如果誘導的是過度耐受，則可能使細胞激素反應不足，增加上行感染風險。成熟中性粒細胞壽命短，因此持久重編程更可能發生在骨髓祖細胞、駐留巨噬細胞或上皮/基質組織記憶層面。

目前證據受限於臨床研究規模小、制劑和給藥途徑不同、致病菌譜差異，以及很少直接取樣膀胱黏膜進行機制驗證。某些免疫預防方法具有臨床前景，但"療效明確由訓練後的單核/巨噬細胞或中性粒細胞介導"這一點仍缺乏直接證據。

Q24

問題

如何透過選擇性重編程腫瘤相關巨噬細胞和中性粒細胞來增強膀胱癌的抗腫瘤免疫，同時盡量減少對保護性尿路黏膜免疫的破壞？請比較不同機制策略及其主要轉譯障礙。

回答

膀胱癌中，選擇性重編程通常比把巨噬細胞或中性粒細胞整體清除更合理，因為這兩類細胞還承擔尿路黏膜抗菌防禦。巨噬細胞方面，一類策略是減少免疫抑制性 TAM 的募集或存活，例如干預 CCL2/CCR2 或 CSF1/CSF1R；另一類是功能重編程，例如利用 CD40、TLR/STING、PI3Kγ或代謝相關策略，提高抗原呈遞和支持 T 細胞殺傷的能力。重編程比全面清除更可能保留吞噬功能，但激活過強也可能造成膀胱炎或全身發炎。

中性粒細胞方面，可通過 CXCR2 相關通路減少腫瘤促進型 TAN 進入，或通過 TGF-β調節、精氨酸/腺苷通路、NET/PAD4 及中性粒細胞蛋白酶抑制，降低 T 細胞抑制和腫瘤侵襲。主要代價是抗菌能力和組織修復下降。因此膀胱內局部給藥很有吸引力：既能充分暴露腫瘤，又可減少全身髓系抑制。BCG 灌注已經證明局部先天免疫激活可以產生抗腫瘤效應，但它並不是選擇性 TAM/TAN 重編程藥物。

轉譯障礙包括髓系細胞高度可塑、缺乏只標記"壞細胞"而不影響保護性黏膜細胞的特異標誌物、骨髓持續補充、不同腫瘤差異大以及通路代償。最現實的方向可能是生物標誌物指導、局部給藥並與免疫檢查點抑制劑等聯合，而不是長期全身抑制整個髓系譜系。
